# Evaluating the performance of ChatGPT-4 on the United Kingdom Medical Licensing Assessment

**DOI:** 10.3389/fmed.2023.1240915

**Published:** 2023-09-19

**Authors:** U Hin Lai, Keng Sam Wu, Ting-Yu Hsu, Jessie Kai Ching Kan

**Affiliations:** ^1^Sandwell and West Birmingham NHS Trust, West Bromwich, United Kingdom; ^2^Aston Medical School, Birmingham, United Kingdom; ^3^University Hospitals Birmingham NHS Trust, Birmingham, United Kingdom; ^4^Worcestershire Acute Hospitals NHS Trust, Worcester, United Kingdom

**Keywords:** examination, ChatGPT, assessment, United Kingdom Medical Licensing Assessment, medical education, medicine, Medical Licensing Examination

## Abstract

**Introduction:**

Recent developments in artificial intelligence large language models (LLMs), such as ChatGPT, have allowed for the understanding and generation of human-like text. Studies have found LLMs abilities to perform well in various examinations including law, business and medicine. This study aims to evaluate the performance of ChatGPT in the United Kingdom Medical Licensing Assessment (UKMLA).

**Methods:**

Two publicly available UKMLA papers consisting of 200 single-best-answer (SBA) questions were screened. Nine SBAs were omitted as they contained images that were not suitable for input. Each question was assigned a specialty based on the UKMLA content map published by the General Medical Council. A total of 191 SBAs were inputted in ChatGPT-4 through three attempts over the course of 3 weeks (once per week).

**Results:**

ChatGPT scored 74.9% (143/191), 78.0% (149/191) and 75.6% (145/191) on three attempts, respectively. The average of all three attempts was 76.3% (437/573) with a 95% confidence interval of (74.46% and 78.08%). ChatGPT answered 129 SBAs correctly and 32 SBAs incorrectly on all three attempts. On three attempts, ChatGPT performed well in mental health (8/9 SBAs), cancer (11/14 SBAs) and cardiovascular (10/13 SBAs). On three attempts, ChatGPT did not perform well in clinical haematology (3/7 SBAs), endocrine and metabolic (2/5 SBAs) and gastrointestinal including liver (3/10 SBAs). Regarding to response consistency, ChatGPT provided correct answers consistently in 67.5% (129/191) of SBAs but provided incorrect answers consistently in 12.6% (24/191) and inconsistent response in 19.9% (38/191) of SBAs, respectively.

**Discussion and conclusion:**

This study suggests ChatGPT performs well in the UKMLA. There may be a potential correlation between specialty performance. LLMs ability to correctly answer SBAs suggests that it could be utilised as a supplementary learning tool in medical education with appropriate medical educator supervision.

## Introduction

Artificial intelligence (AI) can be defined as “human intelligence exhibited by machine” or, more sophisticatedly in the field of AI research, “the study of intelligent agents, which are devices that perceive their environment and take actions to maximize their chance of success at some goal” (1). The initial idea of AI can be traced back to 1950 when Turing (2) proposed the question “Can machines think?” and his concept of the Imitation Game. The Turing test is a “method of inquiry in AI for determining whether or not a computer is capable of thinking like a human being” (3). Turing’s test has become an essential concept in AI philosophy and has been widely discussed and debated over the past several decades (4).

Seventy years after the initial proposal of the Imitation Game concept, the advance in computer chips and microprocessors and the development of deep neural network (DNN) enable computers to exhibit the characteristics of experiential learning by reassembling human intelligence (1, 5), in which the computers demonstrate the capacity to learn through refining their analysis with the use of computational algorithms. AI is widespread today, particularly in business and finance, supply chain management, and cybersecurity (6, 7). Its increasing applications in healthcare have also been evident, for example, histopathological and radiological imaging analysis (8, 9), AI-assisted endoscopy (10), and risk stratification of patients with carotid artery disease (9).

Given the growing phenomenon of AI in healthcare, medical educators should prepare for its potential impact on medical education to maximize learners’ learning opportunities. There have been suggestions that developing AI-driven intelligent tutoring systems can identify gaps in learners’ knowledge; facilitate learning with constructivist approaches; provide thoughtful feedback to students and teachers; and perform time-consuming tasks efficiently, such as recording attendance and grading assessments (11). Nevertheless, given that medicine is a high-stake profession in which training requires high-level accountability and transparency, developing an AI system with accurate and reliable medical knowledge is paramount.

ChatGPT is an AI chatbot developed based on large language models (LLMs). These machine-learning systems can learn autonomously after training on large quantities of unlabeled text, producing sophisticated and seemingly intelligent writing (12). Since the launching of the ChatGPT in November 2022, multiple pieces of literature (13–15) have demonstrated its capability of displaying comprehensible reasoning in professional examinations across different disciplinaries, including the United States Medical Licensing Examination (USMLE), the primary fellowship examination from The Royal College of Anesthetists and the New York State Bar Examination. There have been suggestions that ChatGPT can be used to improve the quality of medical education in several dimensions: automated scoring, teaching assistance, personalized learning, quick access to information, and generating case scenarios (16). Nevertheless, to our knowledge, there has yet to be a current study assessing the performance of ChatGPT in UK undergraduate medical examinations.

With the increasing use of tablet computing in medical education, medical students have found accessing broad medical knowledge easier in classrooms and clinical settings (17). The advent of ChatGPT, alongside the extensive use of technology, has allowed the synthesis of large bodies of medical knowledge to produce a personalized response to a question (18). Differential diagnosis generation is a particular use for junior medical students to help highlight “red flag” conditions that cannot be missed. It has also been proposed that ChatGPT can be used to prepare medical students for practical examinations that assess the clinical skills of medical students, also known as Objective Structured Clinical Examinations (OSCEs) (19). The increasing use of LLMs highlights the role of AI in medical education and the need for further assessment of the accuracy and consistency of this technology.

The United Kingdom Medical Licensing Assessment (UKMLA) is a newly derived national undergraduate medical exit examination. From 2024 onwards, all final-year medical students in the United Kingdom (UK) must pass the UKMLA before graduation (20). The UKMLA is divided into the Applied Knowledge Test (AKT) and the Clinical and Professional Skills Assessment (CPSA). The AKT is a multiple-choice exam consisting of 200 single-best-answer (SBA) questions. Candidates must choose the best answer out of the five, and there is no negative marking. The standard of the AKT is set by a national panel of experts from medical schools across the UK (21). Recently, a study reported that ChatGPT correctly answered 140/191 SBAs (73.3%) on the UKMLA (22).

In this study, we aim to evaluate the performance of ChatGPT in the AKT practice papers published by the Medical Schools Council and if answers generated by ChatGPT are consistent (23). This can serve as an indicator of the clinical knowledge that ChatGPT currently possesses and whether it is a reliable and accountable AI system to facilitate human learning in medical education.

## Methods

### Data collection

Publicly available UKMLA practice materials were utilized for this study (23). These include two AKT practice papers last updated in February 2023. Each practice paper consisted of 100 SBA questions with five choices. Two hundred SBAs were screened for suitability. Nine SBAs were excluded from the study as they included images that could not be input into ChatGPT.

In total, 191 SBAs were inputted individually into ChatGPT-4 May 24 Version 3.5 (OpenAI). ChatGPT answered each question with one of the five choices (e.g., A, B, C, D, E) alongside an explanation on why this is the correct answer. Each attempt was classified as a new attempt. Therefore, a “New Chat” was used for each attempt. ChatGPT had three attempts to answer the complete set of SBAs over 3 weeks (once per week). Three attempts were used to establish whether ChatGPT would generate consistent responses to the same question on each attempt.

### Data analysis

The answers generated by ChatGPT on each attempt at the UKMLA practice materials were recorded into a Microsoft Excel spreadsheet. The answers generated were then compared with the answer key provided by the Medical Schools Council to determine whether ChatGPT provided the correct response for each question. Furthermore, individual SBAs inputted into ChatGPT were assigned a specialty based on the UKMLA Content Map published by the General Medical Council (GMC) in September 2019 (24). This allows the evaluation of the performance of ChatGPT in questions across different specialties. In addition, the variability in the response of ChatGPT to the same question in different attempts was also recorded, which provides a measure of the consistency of the response generated by ChatGPT. The statistical analysis of the data was performed with Microsoft Excel formulas.

## Results

### Performance of ChatGPT on the UKMLA

**Figure 1:**
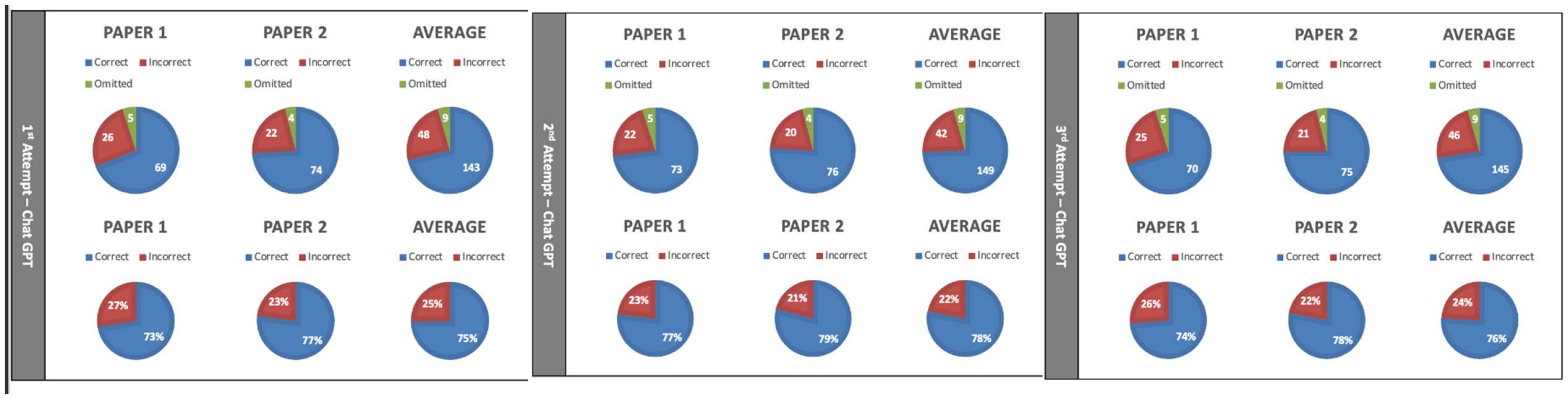
Results of ChatGPT on United Kingdom Medical Licensing Assessment (UKMLA) single-best-answer (SBAs) (*n* = 191) on each of three attempts.

One hundred ninety-one SBAs were inputted into ChatGPT. ChatGPT scored 74.9% (143/191), 78.0% (149/191), and 75.6% (145/191) on three attempts, respectively, (Figure 1). Averaging three attempts, ChatGPT scored 76.3% (437/573) with a 95% confidence interval of (74.46% and 78.08%).

Breakdown analysis of the AKT papers showed that the most tested specialties were obstetrics and gynecology (16 SBAs), acute and emergency (15 SBAs), cancer (14 SBAs), cardiovascular (13 SBAs), musculoskeletal (12 SBAs), infection (12 SBAs) and child health (12 SBAs) (Table 1). Of note, two specialties were only tested with one SBA; both of which ChatGPT generated a correct answer. In terms of the proportion of SBAs being answered correctly across different specialties, ChatGPT performed best in mental health (88.9%), cancer (78.6%), and cardiovascular (77.0%). Its performance in clinical hematology (28.6%), perioperative medicine and anesthesia (33.3%), and endocrine and metabolic (40.0%) were the worst in our study.

**Table 1:** Performance of ChatGPT in relation to specialties tested in the United Kingdom Medical Licensing Assessment (UKMLA).

Speciality	# Questions	# Correct	% Correct
Acute and emergency	15	8	53.33%
Cancer	14	11	78.57%
Cardiovascular	13	10	76.92%
Child health	12	8	66.67%
Clinical haematology	7	2	28.57%
Ear, nose and throat	3	2	66.67%
Endocrine and metabolic	5	2	40.00%
Gastrointestinal including liver	10	6	60.00%
General practice and primary healthcare	4	3	75.00%
Genetics and genomics	2	1	50.00%
Infection	12	9	75.00%
Medical ethics and law	1	1	100.00%
Medicine of older adult	10	5	50.00%
Mental health	9	8	88.89%
Musculoskeletal	12	9	75.00%
Neuroscience	10	8	80.00%
Obstetrics and gynaecology	16	11	68.75%
Ophthalmology	5	3	60.00%
Palliative and end of life care	5	3	60.00%
Perioperative medicine and anaesthesia	3	1	33.33%
Renal and urology	6	4	66.67%
Respiratory	8	7	87.50%
Sexual health	2	1	50.00%
Social and population health	6	5	83.33%
Surgery	1	1	100.00%

An analysis of SBAs where ChatGPT scored incorrectly on all three attempts was also conducted. Clinical hematology (3/7), endocrine and metabolic (2/5), and gastrointestinal, including liver (3/10) were the specialties in which ChatGPT had the highest tendency to consistently provide an incorrect response, accounting for the number of SBAs within a specialty (Table 2).

**Table 2:** Results where ChatGPT generated the incorrect answer on all three attempts.

Speciality	# Questions	# 3 Incorrect	% 3 Incorrect
Acute and emergency	15	4	26.67%
Cancer	14	2	14.29%
Cardiovascular	13	0	0.00%
Child health	12	2	16.67%
Clinical haematology	7	3	42.86%
Ear, nose and throat	3	0	0.00%
Endocrine and metabolic	5	2	40.00%
Gastrointestinal including liver	10	3	30.00%
General practice and primary healthcare	4	1	25.00%
Genetics and genomics	2	0	0.00%
Infection	12	2	16.67%
Medical ethics and law	1	0	0.00%
Medicine of older adult	10	3	30.00%
Mental health	9	0	0.00%
Musculoskeletal	12	1	8.33%
Neuroscience	10	1	10.00%
Obstetrics and gynaecology	16	4	25.00%
Ophthalmology	5	0	0.00%
Palliative and end of life care	5	0	0.00%
Perioperative medicine and anaesthesia	3	1	33.33%
Renal and urology	6	1	16.67%
Respiratory	8	1	12.50%
Sexual health	2	0	0.00%
Social and population health	6	1	16.67%
Surgery	1	0	0.00%

### Consistency of ChatGPT on UKMLA

ChatGPT consistently answered 67.5% (129/191) of SBAs correctly and 12.6% (24/191) SBAs incorrectly on all three attempts. Of note, ChatGPT provided different answers to the same question with the same phrasing in 19.9% (38/191) of SBAs.

Amongst the different specialties, accounting for the number of SBAs within different specialties, ChatGPT provided inconsistent responses most in the field of clinical hemaetology (4/7), genetics and genomes (1/2), ophthalmology (2/5) and medicine of older adults (4/10). In contrast, ChatGPT provided consistent responses in General Practice and Primary Healthcare (4/4) and Social and Population Health (6/6) (Table 3).

**Table 3:** Percentage of response consistency of ChatGPT in different specialties adjusted to the number of SBAs in different specialties.

Out of the 38 inconsistent responses			No. of SBAs in specific specialties	Adjusted to number of questions of individual specialities in UKMLA
Specialties	Number of responses	Percentage (raw)		
Neuroscience	1	2.6%	10	10.0%
Clinical haematology	4	10.5%	7	57.1%
Acute and emergency	4	10.5%	15	26.7%
Ophthalmology	2	5.3%	5	40.0%
Medicine of older adult	4	10.5%	10	40.0%
Cancer	1	2.6%	14	7.1%
Gastrointestinal including liver	2	5.3%	10	20.0%
Endocrine and metabolic	1	2.6%	5	20.0%
Cardiovascular	3	7.9%	13	23.1%
Infection	2	5.3%	12	16.7%
Sexual health	1	2.6%	2	50.0%
Musculoskeletal	3	7.9%	12	25.0%
Mental health	1	2.6%	9	11.1%
Obstetrics and gynaecology	1	2.6%	16	6.3%
Child health	2	5.3%	12	16.7%
Perioperative medicine and anaesthesia	1	2.6%	3	33.3%
Renal and urology	1	2.6%	6	16.7%
Palliative and end of life care	2	5.3%	5	40.0%
Genetics and genomics	1	2.6%	2	50.0%
Ear, nose and throat	1	2.6%	3	33.3%
Total	38	100.0%		

General practice and primary healthcare, social and population health, surgery and medical ethics and law has 100% consistency.

## Discussion

### Discussion on the performance of ChatGPT

Our study suggests ChatGPT performed reasonably well on the UKMLA. As the UKMLA is relatively new and will be fully implemented in early 2024, there needs to be publicly available data on pass marks set by subject matter experts through the modified-Angoff method to determine if ChatGPT “passed” the examination (23). It should be noted that post-graduate medical examinations have been well-established and have statistics relating to pass marks set by standard-setting committees.

Analysing ChatGPT based on specialty performance on the UKMLA demonstrated that it did not perform well on gastrointestinal and liver scoring only 30% (3/10) of SBAs correctly. Interestingly, ChatGPT scored poorly on the American College of Gastroenterology self-assessment suggesting a correlation between specialties and ChatGPT performance (25). From our study, ChatGPT performed less well in the fields of hematology, perioperative medicine, and anesthetics. To date, further studies on ChatGPT performance in these post-graduate specialties have not been conducted. ChatGPT also performed below standard in the field of Acute and Emergency Medicine, answering only 53.3% (8/15) of the questions correctly. Given recognition and management of life-threatening emergencies are the essential competencies of final-year medical students, the under-performance in this area indicates that ChatGPT may not be an appropriate learning tool for undergraduate learners in this specialty, or at least, it should not be used as a main resource of learning.

A study by Jang and Lukasiewicz (26) found that generated answers are inconsistent when input text is paraphrased. Currently, there are no known studies on the consistency of answers generated by ChatGPT from a medical education context. Notably, Al-Shakarachi and Haq (22) also conducted a study on ChatGPT performance in UKMLA practice papers and found it achieved 100% accuracy in emergency medicine, palliative care, and otolaryngology in UKMLA practice papers. Our study has found ChatGPT performed differently in these specialties with 8/15 (53.3%), 3/5 (60.0%), and 2/3 (66.8%) SBAs answered correctly, respectively. This highlights the inconsistency in the performance of ChatGPT that could affect its ability to act as a tool in medical education.

To understand the inconsistency of the answers provided by ChatGPT, we need to discuss how LLM functions. As an LLM, the fundamental principle of how ChatGPT operates is to predict the next most reasonable word from the existing one to create an adequate response utilizing the vast amount of text and information that is fed into its database (27). Using the database, the probability of reasonable words is compared, informing ChatGPT which words are most likely to formulate a satisfactory response. However, if the highest probability word is always used, the response generated would be repetitive within itself, and to avoid creating a paragraph that repeats the same sentence frequently, randomization is added to ChatGPT’s response. This creates a strong ability allowing ChatGPT to generate text from scratch, creating fascinating poems and stories that do not exist in its database, but consequently lead to inconsistency and inaccuracy, which is demonstrated in our study. As mentioned in Hamolak (28) ChatGPT tends to confabulate references to create a sense of plausibility. Moreover, the information that it was trained on also only dates up to September 2021 which also implies the information may be outdated. Nevertheless, ChatGPT is still early in its developmental stage, and in our study, ChatGPT has performed reasonably well and scored 76.3% in UKMLA. The role of ChatGPT in medical education is still widely debated, particularly given the high-stringency nature of the medical profession. However, with the unprecedented speed of advancement in AI, further improvement in the accuracy and reliability of ChatGPT is conceivable. This makes LLM software, such as ChatGPT, a potentially valuable tool in both medical education and clinical practice in the future.

### Comparative analysis of ChatGPT on examinations

The AKT for Membership of the Royal College of General Practitioners in the United Kingdom consists of 200 SBAs sat over 3 h and 10 min: akin to the UKMLA (29). On average, the pass mark has been set at around 70% (141/200) between April 2021 to April 2023 (30–36). Our result shows an average of 76.3% (437/573), suggesting that it could pass the final-year medical school examination. ChatGPT has been studied on various post-graduate medical examinations such as the Fellowship of the Royal College of Ophthalmologists (FRCOphth), the American Radiology Board examination, the Chinese National Medical Licensing Examination, the Taiwanese Pharmacist Licensing Examination and the American College of Gastroenterology self-assessment tests (25, 37–40). ChatGPT fared well on both the FRCOphth and the American Radiology Board Examination. It was unable to pass the Chinese National Medical Licensing Examination and the Taiwanese Licensing Examination. Additionally, it did not pass the American College of Gastroenterology self-assessment scoring 62.4% where the pass mark was set at 70%. It should be noted that these studies also used publicly available question banks where a pass mark was not standard set through the traditional modified-Angoff method.

On medical school entrance examinations, ChatGPT has performed accurately on the National Eligibility cum Entrance Test (NEET) in India (41). ChatGPT-4 scored 72.5%, 44.4%, and 50.5% in physics, chemistry, and biology on the NEET, respectively. The authors of the study suggest that a potential application of LLMs could be to act as a supplementary tool to aid students in preparing for pre-medical examinations and beyond. Additionally, it was also found that ChatGPT fares well in non-medical examinations such as the Test of Mathematics for University Admission (TMUA), Law National Aptitude Test (LNAT), and the Thinking Skills Assessment (TSA) (42); further highlighting the promising potential of the use of LLMs as a supplementary learning tool. However, another study that evaluated the performance of ChatGPT on the Japanese National Medical Practitioners Qualifying Examination (NMPQE) shows some concern about using LLMs as a supplementary learning tool (43). The NMPQE consists of 400 multiple-choice questions taken by medical students in Japan in their final year of medical school. Interestingly, the exam consists of 25+ “prohibited choices.” These prohibited choices are responses that are strictly avoided in medical practice in Japan, such as euthanasia, as it is illegal in Japan. A candidate for the NMPQE would fail the examination if they select more than three prohibited choices. These prohibited choices range from ethically, or legally, incorrect responses to clinically incorrect responses, such as offering oral hypoglycemics to pregnant women. The authors of the study found that ChatGPT-4 tends to select prohibited choices, such as offering euthanasia, in comparison to candidates. This highlights a potential limitation on the application of the use of LLMs as a learning tool for students in healthcare.

### Applications

Despite its suboptimal performance in certain specialties in the UKMLA, the overall high accuracy of ChatGPT on undergraduate medical examinations suggests that it could be utilized, with caution, as a supplementary tool to facilitate learning by UK medical educators and UK learners in both undergraduate and postgraduate medical education settings. It has been shown that ChatGPT can generate clinical scenarios that could be applied in medical education (16, 44). Whether these clinical scenarios are accurate and of sufficient quality has not been studied. Nevertheless, reviewing these generated scenarios by clinicians for medical education use within a respective school would aid in guaranteeing accuracy and quality. Students in medical school could use ChatGPT as an individualized “personal tutor.” ChatGPT can explain medical concepts, generate questions and give feedback to students. Another potential application of ChatGPT in undergraduate medical education is within problem-based learning (PBL).

PBL is widely adopted in medical schools across the world. Typically, it centers on a clinical case, or “problem” where a group of medical students will discuss and solve it under the supervision of a clinical tutor (45). Medical students have been shown to increase knowledge-base and improve on higher-level thinking using PBL (46). Disadvantages of PBL include significant time investment of both students and staff, financial costs, and lack of suitable staff to undertake the role of a clinical tutor, dependent on the university. ChatGPT could play a role in these PBL sessions to address disadvantages. Based on our study, ChatGPT answering around 25% of SBAs incorrectly on the UKMLA suggests that there are limitations to its use and that clinical tutors are still vital in promoting accuracy, quality, and higher-level thinking. It should be noted that ChatGPT in its current form does not access real-time information from the internet (18). Therefore, up-to-date medical practice cannot be utilized in medical education.

### Limitations

A significant limitation of our study was the small sample size of 191 SBAs from UKMLA practice materials, particularly in certain specialties such as medical ethics and law and surgery. Although our study demonstrated ChatGPT performance of 100% in these specialties, there was only one question relevant to ethics and law and surgery, respectively, in the practice materials. This result may not be reliable given the small sample size. Moreover, laws and ethics, and clinical guidance differ by country. The performance of ChatGPT in certain specialties may not be extrapolated to professional medical examinations in other countries. Furthermore, we cannot ascertain if SBAs in publicly available UKMLA practice materials have undergone a similar standard-setting process as the official UKMLA examination. The practice materials simulate the styles of SBAs of the official examination, but it may not be an accurate representation both in terms of the level of difficulty and the proportion of SBAs across different specialties. Further studies on ChatGPT’s performance in the official UKMLA examination with a larger sample of SBAs will be needed to address this. Given the SBAs in the official UKMLA will be undergoing the standard setting process, such as modified-Angoff, each SBAs can be assigned with an index of difficulty. It will be interesting in future studies to look at ChatGPT’s performance on SBAs with different ranges of index of difficulty. Finally, the current version of ChatGPT only allows for text-based input. As such, nine image-based SBAs were excluded from our study. This limitation may have affected the actual performance of ChatGPT.

## Conclusion

Our study has demonstrated the ability of ChatGPT to answer SBAs on the UKMLA. It also noted a potential correlation between different specialties and the performance of ChatGPT. We also noted the possibility of utilizing LLMs as a supplementary learning tool in medical education under the supervision of appropriately trained medical educators. Further avenues of research involving standard-set UKMLA papers, the medical specialty-dependent performance of ChatGPT, and the use of LLMs in medical education could be conducted in the future.

## Data availability statement

The original contributions presented in the study are included in the article/Supplementary material, further inquiries can be directed to the corresponding authors.

## Ethics statement

Ethical review and approval was not required for the study on human participants in accordance with the local legislation and institutional requirements. Written informed consent from the participants was not required to participate in this study in accordance with the national legislation and the institutional requirements.

## Author contributions

UL conceived this paper. UL and KW prepared the manuscript. T-YH prepared figures used in the manuscript. Data collection and analysis were done by UL, KW, T-YH, and JK. All authors contributed to the article and approved the submitted version.

## Conflict of interest

The authors declare that the research was conducted in the absence of any commercial or financial relationships that could be construed as a potential conflict of interest.

## Publisher’s note

All claims expressed in this article are solely those of the authors and do not necessarily represent those of their affiliated organizations, or those of the publisher, the editors and the reviewers. Any product that may be evaluated in this article, or claim that may be made by its manufacturer, is not guaranteed or endorsed by the publisher.

## References

[1] Bini SA. Artificial intelligence, machine learning, deep learning, and cognitive computing: what do these terms mean and how will they impact health care? J Arthroplast. (2018) 33:2358–61. doi: 10.1016/j.arth.2018.02.067, PMID: 29656964

[2] Turing AM. Computing machinery and intelligence. Mind. (1950) LIX:433–60. doi: 10.1093/mind/LIX.236.433

[3] St George B Gillis AS. Turing test. Available at: https://www.techtarget.com/searchenterpriseai/definition/Turing-test (Accessed June 14, 2023)

[4] Saygin AP Cicekli I Akman V. Turing test: 50 years later. Mind Mach. (2000) 10:463–518. doi: 10.1023/A:1011288000451

[5] Helm JM Swiergosz AM Haeberie HS Karnuta JM Schaffer JL Krebs VE. Machine learning and artificial intelligence: definitions, applications, and future directions. Curr Rev Musculoskelet Med. (2020) 13:69–76. doi: 10.1007/s12178-020-09600-8, PMID: 31983042 PMC7083992

[6] Collins C Dennehy D Conboy K Mikalef P. Artificial intelligence in information systems research: a systematic literature review and research agenda. Int J Inf Manag. (2021) 60:102383–17. doi: 10.1016/j.ijinfomgt.2021.102383

[7] Sarker IH. AI-based modeling: techniques, applications and research issues towards automation, intelligent and smart systems. SN Comput Sci. (2022) 3:158. doi: 10.1007/s42979-022-01043-x 35194580 PMC8830986

[8] Stifanic J Stifanic D Zulijani A Car Z. Application of AI in histopathological image analysis In: Filipovic N, editor. Applied artificial intelligence: medicine, biology, chemistry, financial, games, engineering. Cham: Springer (2023). 121–31.

[9] Blagojevic A Geroski T. A review of the application of artificial intelligence in medicine: from data to personalised models In: Filipovic N, editor. Applied artificial intelligence: medicine, biology, chemistry, financial, games, engineering. Cham: Springer (2023). 271–306.

[10] Nakase H Hirano T Wagatsuma K Ichimiya T Yamakawa T Yokoyama Y. Artificial intelligence-assisted endoscopy changes the definition of mucosal healing in ulcerative colitis. Dig Endosc. (2021) 33:903–11. doi: 10.1111/den.13825, PMID: 32909283 PMC8647580

[11] Masters K. Artificial intelligence in medical education. Med Teach. (2019) 41:976–80. doi: 10.1080/0142159X.2019.1595557 31007106

[12] van Dis EAM Bollen J Zuidema W van Rooij R Bockting CL. ChatGPT: five priorities for research. Nature. (2023) 614:224–6. doi: 10.1038/d41586-023-00288-7, PMID: 36737653

[13] Kung TH Cheatham M Medenilla A Sillos C De Leon L Elepano C. Performance of ChatGPT on USMLE: potential for AI-assisted medical education using large language models. PLoS Digit Health. (2023) 2:1–12. doi: 10.1371/journal.pdig.0000198 PMC9931230 36812645

[14] Bommarito M Katz DM. GPT takes the Bar Exam. *arXiv* (2022). Available at: 10.48550/arXiv.2212.14402. [Epub ahead of preprint]. (Accessed June 14, 2023)

[15] Aldridge MJ Penders R. Artificial intelligence and anaesthesia examinations: exploring ChatGPT as a prelude to the future. Br J Anaesth. (2023) 131:e36–7. doi: 10.1016/j.bja.2023.04.033, PMID: 37244834

[16] Kan RA Jawaid M Khan QR Sajjad M. ChatGPT—reshaping medical education and clinical management. Pak J Med Sci. (2023) 39:605–7. doi: 10.12669/pjms.39.2.7653 36950398 PMC10025693

[17] Leydon GB Schwartz ML. The use of mobile devices to enhance engagement and integration with curricular content. Yale J Bio Med. (2020) 93:453–60. 32874152 PMC7448388

[18] Tsang R. Practical applications of ChatGPT in undergraduate medical education. J Med Educ Curric Dev. (2023) 10:238212052311784. doi: 10.1177/23821205231178449 PMC10226299 37255525

[19] Soong TK Ho C-M. Artificial intelligence in medical OSCES: reflections and future developments. Adv Med Educ Pract. (2021) 12:167–73. doi: 10.2147/AMEP.S287926 33628074 PMC7899303

[20] General Medical Council. Medical Licensing Assessment. Available at: https://www.gmc-uk.org/education/medical-licensing-assessment (Accessed June 14, 2023)

[21] Medical Schools Council. The Applied Knowledge Test—FAQs for UK medical students. (2023). Available at: https://www.medschools.ac.uk/media/3015/the-akt-faqs-for-uk-medical-students-january-2023.pdf (Accessed June 14, 2023)

[22] Al-Shakarachi NJ Haq IU. ChatGPT performance in the UK medical licensing assessment: how to train the next generation? Mayo Clinic Proc. (2023) 1:309–10. doi: 10.1016/j.mcpdig.2023.06.004 PMC11975728 40206625

[23] Medical Schools Council. Medical Licensing Assessment—practice materials. (2023). Available at: https://www.medschools.ac.uk/studying-medicine/medical-licensing-assessment/practice-materials (Accessed June 14, 2023)

[24] The General Medical Council. MLA content map. Available at: https://www.gmc-uk.org/-/media/documents/mla-content-map-_pdf-85707770.pdf (Accessed June 14, 2023)

[25] Suchman K Garg S Trindade AJ. Chat Generative Pretrained Transformer fails the multiple-choice American College of Gastroenterology self-assessment test. Am J Gastroenterol. (2023). doi: 10.14309/ajg.0000000000002320, PMID: 37212584

[26] Jang M Lukasiewicz T. Consistency analysis of ChatGPT. *arXiv*. (2023). Available at: 10.48550/arXiv.2303.06273. [Epub ahead of preprint]

[27] Wolfram S. What is ChatGPT doing … and why does it work?. Available at: https://writings.stephenwolfram.com/2023/02/what-is-chatgpt-doing-and-why-does-it-work/

[28] Hamolak J. Opportunities and risks of ChatGPT in medicine, science and academic publishing: a modern promethean dilemma. Croat Med J. (2023) 64:1–3. doi: 10.3325/cmj.2023.64.1, PMID: 36864812 PMC10028563

[29] Royal College of General Practitioners. MRCGP: Applied Knowledge Test (AKT). Available at: https://www.rcgp.org.uk/mrcgp-exams/applied-knowledge-test (Accessed June 14, 2023)

[30] Royal College of General Practitioners. Feedback on the MRCGP Applied Knowledge Test (AKT) April 2021, AKT 42. Available at: https://www.rcgp.org.uk/getmedia/0c476e63-ccc7-4738-a2d7-7b75978564ec/akt-feedback-report-apr21.pdf (Accessed June 14, 2023)

[31] Royal College of General Practitioners. Feedback on the MRCGP Applied Knowledge Test (AKT) October 2021, AKT 43. Available at: https://www.rcgp.org.uk/getmedia/0f74e174-62b9-45f3-a060-306eaeba6563/AKT-feedback-report-October-2021.pdf (Accessed June 14, 2023)

[32] Royal College of General Practitioners. Feedback on the MRCGP Applied Knowledge Test (AKT) January 2022, AKT 44. Available at: https://www.rcgp.org.uk/getmedia/568d3ca9-2c3b-4be5-bf7b-26fca49cd171/AKT-44-feedback-draft_FINAL.pdf (Accessed June 14, 2023)

[33] Royal College of General Practitioners. Feedback on the MRCGP Applied Knowledge Test (AKT) April 2022, AKT 45. Available at: https://www.rcgp.org.uk/getmedia/4194f100-08f7-4310-ab95-cdfd4c7c03e6/April-2022-AKT-feedback-report.pdf (Accessed June 14, 2023)

[34] Royal College of General Practitioners. Feedback on the MRCGP Applied Knowledge Test (AKT) October 2022, AKT 46. Available at: https://www.rcgp.org.uk/getmedia/deeef4b0-38fc-4ade-b4a4-d5948b32074d/October-2022-AKT-Feedback-Report.pdf (Accessed June 14, 2023)

[35] Royal College of General Practitioners. Feedback on the MRCGP Applied Knowledge Test (AKT) January 2023, AKT 47. Available at: https://www.rcgp.org.uk/getmedia/5695c150-4460-4484-8fcb-259025cd05ea/january-2023-akt-feedback-report.pdf (Accessed June 14, 2023)

[36] Royal College of General Practitioners. Feedback on the MRCGP Applied Knowledge Test (AKT) April 2023, AKT 48. Available at: https://www.rcgp.org.uk/getmedia/df2c3ea2-695c-4c6c-aa89-8ad37ad6a0d5/Feedback-on-the-MRCGP-Applied-Knowledge-Test-(AKT)-April-2023.pdf (Accessed June 14, 2023)

[37] Raimondi R Tzoumas N Salisbury T Di Simplicio S Romano MR North East Trainee Research in Ophthalmology Network (NETRiON. Comparative analysis of large language models in the Royal College of Ophthalmologists fellowship exams. Eye. (2023). doi: 10.1038/s41433-023-02563-3, PMID: 37161074 PMC10686375

[38] Bhayana R Krishna S Bleakney RR. Performance of ChatGPT on a radiology board-style examination: insights into current strengths and limitations. Radiology. (2023) 307:1–8. doi: 10.1148/radiol.230582 37191485

[39] Wang YM Shen HW Chen TJ. Performance of ChatGPT on the pharmacist licensing examination in Taiwan. J Chin Med Assoc. (2023) 86:653–8. doi: 10.1097/JCMA.0000000000000942, PMID: 37227901 PMC12755457

[40] Wang X Gong Z Wang G Jia J Xu Y Zhao J. ChatGPT performs on the Chinese National Medical Licensing Examination. J Med Syst. (2023) 47:86. doi: 10.1007/s10916-023-01961-0, PMID: 37581690

[41] Farhat F Chaudry BM Nadeem M Sohail SS Madsen DO Evaluating AI models for the National Pre-Medical Exam in India: a head-to-head analysis of ChatGPT-3.5, GPT-4 and Bard. *JMIR Preprints*. Available at: https://preprints.jmir.org/preprint/51523. [Epub ahead of preprint] 10.2196/51523 PMC10918540 38381486

[42] Giannos P Delardas O. Performance of ChatGPT on UK standardized admission tests: insights from the BMAT, TMUA, LNAT and TSA examinations. JMIR Med Educ. (2023) 9:e47737. doi: 10.2196/47737, PMID: 37099373 PMC10173042

[43] Kasai J Kasai Y Sakaguchi K Yamada Y Radev D Evaluating GPT-4 and ChatGPT on Japanese Medical Licensing Examinations. *arXiv*. Available at: 10.48550/arXiv.2303.18027. [Epub ahead of preprint].

[44] Eysenbach G. The role of ChatGPT, generative language models, and artificial intelligence in medical education: a conversation with ChatGPT and a call for papers. JMIR Med Educ. (2023) 9:1–13. doi: 10.2196/46885 PMC10028514 36863937

[45] Jaffarey NA. Problem based learning. J Pak Med Assoc. (2001) 51:1–3. 11715885

[46] Abdelkarim A Schween D Timothy F. Advantages and disadvantages of problem-based learning from the professional perspective of medical and dental faculty. EC Dent Sci. (2018) 17:1–7.

