# Supplementary material for: Evaluating the performance of ChatGPT-4 on the United Kingdom Medical Licensing Assessment
Source: Front Med (Lausanne). 2023 Sep 19;10:1240915. doi: 10.3389/fmed.2023.1240915 (PMC10547055; doi:10.3389/fmed.2023.1240915)
Supplement: Supplementary file 1 [file Data_Sheet_1.docx]

Paper 1

| **Question no.** | **Input** | **Output** |
| --- | --- | --- |
| **1** | A 24 year old man has poor urine flow and takes a very long time to empty his  bladder. He has no other urinary symptoms. He has been well previously  apart from one episode of non-gonococcal urethritis 1 year ago.  Which is the most likely diagnosis?  A. Overactive bladder  B. Neurogenic bladder  C. Phimosis  D. Prostatic hypertrophy  E. Urethral stricture | Attempt 1  Based on the given information, the most likely diagnosis for the 24-year-old man with poor urine flow and a prolonged time to empty his bladder, with no other urinary symptoms, is **E. Urethral stricture**.  Urethral stricture refers to the narrowing of the urethra, the tube that carries urine from the bladder out of the body. It can result from previous inflammation or scarring, such as the episode of non-gonococcal urethritis mentioned in the case history. Urethral strictures can lead to a reduced urine flow and difficulty emptying the bladder completely.  The other options can be ruled out based on the information provided:  A. Overactive bladder: Overactive bladder typically presents with symptoms like urinary urgency, frequent urination, and sometimes urinary incontinence. These symptoms are not mentioned in the case.  B. Neurogenic bladder: Neurogenic bladder is a condition that occurs due to a problem with the nerves that control bladder function. It can result in various urinary symptoms, including urinary incontinence, urinary retention, and difficulty emptying the bladder completely. However, in this case, there are no indications of neurogenic bladder.  C. Phimosis: Phimosis refers to the inability to retract the foreskin over the glans penis. It does not typically cause urinary symptoms such as poor urine flow or difficulty emptying the bladder.  D. Prostatic hypertrophy: Prostatic hypertrophy, also known as benign prostatic hyperplasia (BPH), usually affects older men. It involves the enlargement of the prostate gland, which can lead to urinary symptoms such as weak urine flow, frequent urination, and difficulty initiating urination. Given the age of the patient (24 years old), prostatic hypertrophy is less likely to be the cause.  Therefore, based on the given information, urethral stricture is the most likely diagnosis for this patient. However, it is important for him to consult a healthcare professional for a proper evaluation and diagnosis. |
|  |  | Attempt 2  Based on the information provided, the most likely diagnosis for a 24-year-old man with poor urine flow and a long time to empty his bladder, without any other urinary symptoms, would be E. Urethral stricture.  Urethral stricture refers to the narrowing of the urethra, which can obstruct the flow of urine and cause difficulties with emptying the bladder. It can be caused by various factors, such as inflammation, trauma, or previous infections like non-gonococcal urethritis.  The other options can be ruled out based on the information provided:  A. Overactive bladder typically presents with symptoms of urgency, frequency, and urge incontinence, which are not mentioned in the scenario.  B. Neurogenic bladder is characterized by abnormal bladder function due to nerve damage, often associated with conditions like spinal cord injury or neurological diseases. The absence of other symptoms or relevant history makes it less likely in this case.  C. Phimosis refers to the inability to retract the foreskin over the glans penis, which is unlikely to cause urinary flow issues or difficulty emptying the bladder.  D. Prostatic hypertrophy (enlargement of the prostate) typically occurs in older males and may cause obstructive urinary symptoms like weak urine flow, hesitancy, and incomplete emptying. However, it is less likely in a 24-year-old individual, unless there are specific risk factors or underlying conditions not mentioned in the scenario.  It's important to note that a proper medical evaluation, including a physical examination and additional diagnostic tests, would be necessary to confirm the diagnosis and determine the appropriate treatment. If the individual is experiencing urinary symptoms, it is recommended to consult with a healthcare professional for an accurate diagnosis and further management. |
|  |  | Attempt 3  Based on the given information, the most likely diagnosis for the 24-year-old man with poor urine flow and delayed bladder emptying is E. Urethral stricture.  Urethral stricture refers to a narrowing of the urethra, which can obstruct the flow of urine. It can be caused by various factors, including previous infections or trauma to the urethra. The episode of non-gonococcal urethritis the man experienced one year ago could have contributed to the development of a urethral stricture.  Overactive bladder (option A) typically presents with symptoms such as frequent urination, urgency, and sometimes urinary incontinence. These symptoms are not mentioned in the case.  Neurogenic bladder (option B) is characterized by dysfunction of the bladder due to a neurological condition, which can result in problems with bladder emptying or control. However, there are no indications of any neurological symptoms or conditions in the given information.  Phimosis (option C) is a condition in which the foreskin of the penis cannot be retracted. It does not directly cause poor urine flow or delayed bladder emptying.  Prostatic hypertrophy (option D), also known as benign prostatic hyperplasia (BPH), is a condition commonly seen in older men. It involves an enlargement of the prostate gland, which can lead to urinary symptoms such as poor urine flow, frequent urination, and difficulty emptying the bladder. However, BPH is rare in a 24-year-old, and the absence of other urinary symptoms makes it less likely.  Therefore, based on the given information, the most likely diagnosis for the 24-year-old man is E. Urethral stricture. However, a proper evaluation by a healthcare professional is essential to confirm the diagnosis and determine the appropriate treatment. |
| **2** | A 67 year old man is found to have an ejection systolic murmur. He is  otherwise well. His pulse rate is 72 bpm and BP 128/84 mmHg. His chest is  clear.  Investigations:  ECG shows sinus rhythm.  Echocardiography shows aortic stenosis, valve gradient 50 mmHg. Left  ventricular (LV) diastolic dysfunction, LV ejection fraction 45% (>55).  Which is the most appropriate management?  A. Clinical review and echocardiography in 6 months  B. Reassure and discharge  C. Refer for aortic valve replacement  D. Start bisoprolol fumarate and advise review if symptomatic  E. Start lisinopril and advise review if symptomatic | Attempt 1  Based on the provided information, the most appropriate management for the 67-year-old man with an ejection systolic murmur, aortic stenosis with a valve gradient of 50 mmHg, and evidence of left ventricular diastolic dysfunction would be C. Refer for aortic valve replacement.  Aortic stenosis is a condition characterized by the narrowing of the aortic valve, leading to obstruction of blood flow from the left ventricle to the aorta. It typically presents with an ejection systolic murmur. In this case, the significant valve gradient of 50 mmHg indicates moderate to severe aortic stenosis.  The presence of left ventricular diastolic dysfunction and a reduced left ventricular ejection fraction (45% in this case) suggests that the heart is experiencing strain and impaired function due to the aortic stenosis.  Given the severity of the aortic stenosis and the associated cardiac dysfunction, the most appropriate management would be to refer the patient for aortic valve replacement. Aortic valve replacement is the definitive treatment for symptomatic severe aortic stenosis or when there are signs of left ventricular dysfunction, as in this case.  The other options are not appropriate for this patient:  A. Clinical review and echocardiography in 6 months would delay the necessary intervention and may increase the risk of complications or disease progression.  B. Reassuring and discharging the patient would be inappropriate as the patient has moderate to severe aortic stenosis and evidence of left ventricular dysfunction, indicating the need for further evaluation and management.  D. Starting bisoprolol fumarate, a beta-blocker, and advising review if symptomatic may help with symptom control in patients with aortic stenosis but is not the definitive treatment. Given the evidence of left ventricular dysfunction, the patient should be referred for aortic valve replacement.  E. Starting lisinopril, an angiotensin-converting enzyme (ACE) inhibitor, and advising review if symptomatic may be useful in managing hypertension or heart failure, but it does not address the underlying cause of aortic stenosis. Referral for aortic valve replacement is necessary in this case.  It's important for the patient to consult with a cardiologist or a healthcare professional specializing in cardiovascular diseases for further evaluation and to determine the most appropriate treatment plan. |
|  |  | Attempt 2  The most appropriate management for a 67-year-old man with an ejection systolic murmur, aortic stenosis with a valve gradient of 50 mmHg, and LV diastolic dysfunction with an LV ejection fraction of 45% (>55) is:  C. Refer for aortic valve replacement  The findings of aortic stenosis with a significant valve gradient and LV dysfunction indicate a significant cardiac pathology that requires intervention. Aortic valve replacement is the definitive treatment for symptomatic severe aortic stenosis, especially in patients with LV dysfunction.  Options A and B are not appropriate because the patient has significant cardiac abnormalities that need further evaluation and intervention.  Option D (Start bisoprolol fumarate and advise review if symptomatic) and Option E (Start lisinopril and advise review if symptomatic) may be used in certain cases to manage symptoms or as adjunct therapy, but they are not the primary treatment for severe aortic stenosis with LV dysfunction. In this case, the definitive management is aortic valve replacement.  It is essential for the patient to be referred to a specialist, usually a cardiologist or a cardiac surgeon, for further evaluation and management. The decision to proceed with valve replacement will depend on the patient's symptoms, clinical condition, and other individual factors. |
|  |  | Attempt 3  Based on the given information, the most appropriate management for the 67-year-old man with an ejection systolic murmur and findings of aortic stenosis on echocardiography is C. Refer for aortic valve replacement.  Aortic stenosis is a condition characterized by narrowing of the aortic valve, leading to obstruction of blood flow from the left ventricle to the aorta. It is typically associated with an ejection systolic murmur. In this case, the echocardiography findings indicate significant aortic stenosis with a valve gradient of 50 mmHg.  The presence of significant aortic stenosis and left ventricular diastolic dysfunction, along with a decreased left ventricular ejection fraction (45% in this case), suggests that the patient may be at risk for symptomatic heart failure and adverse cardiac events.  Given the severity of the aortic stenosis and the potential for complications, the most appropriate management is to refer the patient for aortic valve replacement. Aortic valve replacement is the definitive treatment for symptomatic severe aortic stenosis, and it may also be considered in asymptomatic patients with evidence of significant left ventricular dysfunction or other high-risk features.  Options A, B, and D are not appropriate in this case. Waiting for six months (option A) without intervention could lead to disease progression and worsening symptoms. Reassuring and discharging the patient (option B) would neglect the significant aortic stenosis and the associated risks. Starting bisoprolol fumarate (option D) may help manage symptoms in some cases, but it does not address the underlying problem of severe aortic stenosis.  Option E, starting lisinopril, an angiotensin-converting enzyme (ACE) inhibitor, is not the most appropriate management for aortic stenosis. ACE inhibitors are generally used for the management of heart failure with reduced ejection fraction (HFrEF), but in this case, the patient has preserved ejection fraction (EF) with diastolic dysfunction, not HFrEF.  Therefore, based on the provided information, the most appropriate management for the 67-year-old man with aortic stenosis and associated findings is C. Refer for aortic valve replacement. A cardiologist or cardiac surgeon should evaluate the patient further to determine the optimal timing and approach for the valve replacement procedure. |
| **3** | A 27 year old woman has muscle weakness which is worse on exercise.  When asked to count from one to 100 her voice progressively becomes  weaker. She has bilateral ptosis.  Which pathophysiological process is most likely to be responsible for this disorder?  A. Autoimmunity  B. Genetic disorder  C. Infarction  D. Malignancy  E. Meningeal infection | Attempt 1  Based on the provided information, the most likely pathophysiological process responsible for the described disorder in the 27-year-old woman with muscle weakness, worsening on exercise, progressive weakening of voice during counting, and bilateral ptosis is A. Autoimmunity.  The clinical presentation suggests a neuromuscular disorder known as myasthenia gravis (MG). MG is an autoimmune disease characterized by the presence of autoantibodies that target and impair the function of the neuromuscular junction. This leads to muscle weakness, which typically worsens with activity and improves with rest.  The bilateral ptosis (drooping eyelids) is a classic feature of MG, as is the involvement of muscles responsible for voice production, resulting in progressive weakening of the voice during sustained speech or counting.  The other options can be ruled out based on the given information:  B. Genetic disorder: While some cases of MG may have a genetic predisposition, the acute onset of symptoms in a 27-year-old woman is less suggestive of a primary genetic disorder as the sole cause.  C. Infarction: Infarction, which refers to tissue death due to inadequate blood supply, is not a common cause of the described symptoms in a young patient.  D. Malignancy: Malignancies can rarely be associated with paraneoplastic syndromes that affect the neuromuscular system, but they are less common than autoimmune causes like MG.  E. Meningeal infection: Meningeal infections, such as meningitis, typically present with symptoms like headache, neck stiffness, fever, and altered mental status, rather than the specific pattern of muscle weakness and ptosis described in this case.  It's important for the patient to consult with a neurologist or a healthcare professional experienced in neuromuscular disorders for further evaluation, confirmation of the diagnosis, and initiation of appropriate treatment, which may include medications to suppress the autoimmune response or enhance neuromuscular transmission. |
|  |  | Attempt 2  The most likely pathophysiological process responsible for the described disorder in a 27-year-old woman with muscle weakness that worsens on exercise, progressive weakness of voice while counting from one to 100, and bilateral ptosis is:  A. Autoimmunity  This constellation of symptoms is highly suggestive of a condition called Myasthenia Gravis (MG), which is an autoimmune neuromuscular disorder. In MG, the body's immune system produces antibodies that attack and interfere with the normal functioning of acetylcholine receptors at the neuromuscular junction. Acetylcholine is a neurotransmitter responsible for transmitting signals between nerves and muscles, enabling normal muscle contractions.  The characteristic symptoms of MG include muscle weakness, which typically worsens with repeated muscle use (exercise), and improves with rest. Ptosis (drooping of the eyelids) and weakness of eye muscles (ocular muscles) are common early signs. Involvement of the muscles controlling the voice (speech) can lead to progressively weakening voice, as seen in this case when counting.  Options B, C, D, and E are less likely causes of these specific symptoms and are not typically associated with the clinical features of Myasthenia Gravis. |
|  |  | Attempt 3  Based on the given information, the most likely pathophysiological process responsible for the presented symptoms in the 27-year-old woman is A. Autoimmunity.  The symptoms of muscle weakness, worsening on exercise, and progressively weaker voice, along with bilateral ptosis (drooping of the eyelids), strongly suggest a neuromuscular disorder known as myasthenia gravis (MG). MG is an autoimmune condition in which antibodies target and impair the function of acetylcholine receptors at the neuromuscular junction, leading to muscle weakness.  The characteristic feature of worsening muscle weakness with activity, known as fatigability, is a hallmark of myasthenia gravis. In this case, the progressive weakening of the voice while counting from one to 100 further supports the diagnosis of MG.  While genetic disorders (option B) can cause muscle weakness, the specific pattern of symptoms and the presence of ptosis make autoimmune processes more likely.  Infarction (option C) refers to tissue damage caused by the interruption of blood supply, typically due to a blocked blood vessel. It is not the primary mechanism underlying myasthenia gravis.  Malignancy (option D) can rarely be associated with paraneoplastic syndromes that affect the neuromuscular system. However, given the specific constellation of symptoms and the absence of any information suggesting an underlying malignancy, it is less likely in this case.  Meningeal infection (option E) typically presents with symptoms such as fever, headache, neck stiffness, and neurological signs specific to the affected area. The symptoms described in the case (muscle weakness, worsening with exercise, bilateral ptosis, and weakening voice) are not consistent with a meningeal infection.  Therefore, based on the provided information, the most likely pathophysiological process responsible for the presented symptoms in the 27-year-old woman is A. Autoimmunity, specifically myasthenia gravis. A thorough evaluation by a healthcare professional, including specific diagnostic tests, would be necessary to confirm the diagnosis and initiate appropriate treatment. |
| **4** | A 60 year old man has 6 months of dry cough and increasing shortness of  breath on effort. He was previously fit and well, and is a non-smoker.  His temperature is 36.8°C, pulse rate 60 bpm and oxygen saturation 89%  breathing air.  He has finger clubbing. Cardiac examination is normal, and chest examination  reveals bibasal crepitations.  Which is the most likely diagnosis?  A. Bronchiectasis  B. Extrinsic allergic alveolitis  C. Idiopathic pulmonary fibrosis  D. Lung carcinoma  E. Pulmonary tuberculosis | Attempt 1  Based on the provided information, the most likely diagnosis for the 60-year-old man with a 6-month history of dry cough, increasing shortness of breath on exertion, finger clubbing, bibasal crepitations on chest examination, and oxygen saturation of 89% breathing air is C. Idiopathic pulmonary fibrosis.  Idiopathic pulmonary fibrosis (IPF) is a chronic and progressive interstitial lung disease characterized by the formation of scar tissue (fibrosis) within the lungs. The typical clinical presentation includes a dry cough and exertional dyspnea (shortness of breath on effort). Finger clubbing is often associated with IPF and can be a result of chronic hypoxia.  The bibasal crepitations (crackles) on chest examination are indicative of fibrotic changes and abnormal lung sounds associated with IPF.  The other options can be ruled out based on the given information:  A. Bronchiectasis: While bronchiectasis can present with a chronic cough and shortness of breath, it is less likely in this case as there are no other typical signs, such as recurrent chest infections or sputum production.  B. Extrinsic allergic alveolitis: Extrinsic allergic alveolitis, also known as hypersensitivity pneumonitis, typically presents with respiratory symptoms triggered by exposure to certain allergens. It is less likely in this case as there is no specific exposure history mentioned, and the clinical presentation and findings are more suggestive of IPF.  D. Lung carcinoma: Lung carcinoma can present with cough, shortness of breath, and finger clubbing, but typically it is associated with other signs such as weight loss, hemoptysis (coughing up blood), or chest pain. Additionally, the absence of abnormal findings on cardiac examination makes lung carcinoma less likely.  E. Pulmonary tuberculosis: While pulmonary tuberculosis can present with cough and shortness of breath, it is less likely in a non-smoking individual without other significant risk factors or symptoms like fever, night sweats, or weight loss. Moreover, the chronicity of symptoms and bibasal crepitations are more consistent with a chronic interstitial lung disease like IPF.  It is crucial for the patient to consult with a pulmonologist or a healthcare professional specializing in lung diseases for further evaluation, including imaging studies such as high-resolution computed tomography (HRCT) of the chest, pulmonary function tests, and possibly a lung biopsy, to confirm the diagnosis of idiopathic pulmonary fibrosis and initiate appropriate management. |
|  |  | Attempt 2  The most likely diagnosis for the 60-year-old man with a 6-month history of dry cough, increasing shortness of breath on effort, finger clubbing, normal cardiac examination, and bibasal crepitations on chest examination is:  C. Idiopathic pulmonary fibrosis  Idiopathic pulmonary fibrosis (IPF) is a chronic and progressive interstitial lung disease characterized by the gradual scarring (fibrosis) of the lung tissue over time. The exact cause of IPF is unknown (idiopathic), but it is believed to result from abnormal wound healing and inflammation in the lungs. It predominantly affects older individuals, like the 60-year-old man described in the case.  The symptoms of IPF include dry cough, exertional dyspnea (shortness of breath with activity), and finger clubbing. Bibasal crepitations (fine crackling sounds heard during lung auscultation) are typical findings on chest examination in patients with IPF.  Options A (Bronchiectasis), B (Extrinsic allergic alveolitis), D (Lung carcinoma), and E (Pulmonary tuberculosis) may present with similar symptoms like cough and shortness of breath, but the presence of finger clubbing and bibasal crepitations are more characteristic of IPF.  However, it is essential to confirm the diagnosis through further investigations, including pulmonary function tests and high-resolution computed tomography (HRCT) of the chest. A multidisciplinary approach involving pulmonologists and radiologists is crucial in making the definitive diagnosis and determining the most appropriate management for the patient. |
|  |  | Attempt 3  Based on the given information, the most likely diagnosis for the 60-year-old man with a 6-month history of dry cough, increasing shortness of breath, finger clubbing, bibasal crepitations on chest examination, and low oxygen saturation is C. Idiopathic pulmonary fibrosis (IPF).  IPF is a chronic and progressive interstitial lung disease characterized by fibrosis (scarring) of the lung tissue. It typically presents with a gradual onset of symptoms such as dry cough and progressive exertional dyspnea (shortness of breath on effort). The presence of bibasal crepitations on chest examination and finger clubbing further support the diagnosis.  Bronchiectasis (option A) is a condition characterized by permanent dilation and thickening of the bronchi, leading to recurrent lung infections, productive cough, and shortness of breath. However, the given information does not suggest a history of recurrent lung infections or productive cough, making bronchiectasis less likely.  Extrinsic allergic alveolitis (option B), also known as hypersensitivity pneumonitis, is an immune-mediated lung disease caused by repeated exposure to inhaled organic antigens. It typically presents with symptoms such as cough, dyspnea, and fever, often with a temporal relationship to antigen exposure. The absence of exposure history and the chronicity of symptoms make this diagnosis less likely.  Lung carcinoma (option D) can present with chronic cough and shortness of breath, but it is more commonly associated with other symptoms such as weight loss, hemoptysis (coughing up blood), and chest pain. The given information does not provide any suggestive features of lung carcinoma.  Pulmonary tuberculosis (option E) can cause chronic cough and shortness of breath, but it is usually associated with other symptoms such as fever, night sweats, weight loss, and productive cough with sputum. The absence of these additional symptoms and the presence of bibasal crepitations and finger clubbing make pulmonary tuberculosis less likely.  Therefore, based on the provided information, the most likely diagnosis for the 60-year-old man is C. Idiopathic pulmonary fibrosis. However, further evaluation and diagnostic tests, such as pulmonary function tests and high-resolution computed tomography (HRCT) of the chest, would be necessary to confirm the diagnosis and guide management. Referral to a pulmonologist or respiratory specialist is recommended. |
| **5** | A 46 year old man has pain in his left leg and tingling in his left big toe. He  developed severe lower back pain 1 week ago and he is unable to walk on his  left heel. There is loss of pinprick perception over the left great toe.  Which nerve root is the most likely to have been affected?  A. L1  B. L3  C. L5  D. S1 | Attempt 1  Based on the provided information, the most likely nerve root that has been affected in the 46-year-old man with left leg pain, tingling in the left big toe, severe lower back pain, inability to walk on the left heel, and loss of pinprick perception over the left great toe is D. S1.  The symptoms described, including lower back pain, radiating pain along the left leg, tingling in the left big toe, and loss of pinprick perception over the left great toe, are consistent with a clinical presentation known as "sciatica." Sciatica refers to the irritation or compression of the sciatic nerve, which is the largest nerve in the body and originates from nerve roots in the lower back (specifically L4 to S3).  The S1 nerve root, in particular, supplies the sensory innervation to the back of the calf, heel, and the sole of the foot. Loss of pinprick perception over the left great toe suggests involvement of the S1 nerve root, as this region falls within its dermatome.  The other options can be ruled out based on the information provided:  A. L1: Symptoms affecting the lower leg and great toe are not typically associated with the involvement of the L1 nerve root.  B. L3: The symptoms described do not correspond to the dermatomal distribution of the L3 nerve root, which primarily supplies the anterior thigh.  C. L5: Although L5 nerve root involvement can cause similar symptoms, such as radiating pain and tingling down the leg, the specific loss of pinprick perception over the left great toe suggests a more distal nerve root involvement, such as S1.  E. S2: Symptoms described do not correspond to the dermatomal distribution of the S2 nerve root, which is more involved with the posterior thigh, back of the knee, and calf.  It's important for the patient to consult with a healthcare professional, preferably a neurologist or orthopedic specialist, for further evaluation, including a thorough physical examination, possibly imaging studies (such as MRI of the lumbar spine), and potentially additional tests to confirm the diagnosis and guide appropriate treatment. |
|  |  | Attempt 2  Based on the symptoms described, the most likely affected nerve root is:  C. L5  The combination of severe lower back pain, pain in the left leg, tingling in the left big toe, inability to walk on the left heel, and loss of pinprick perception over the left great toe is indicative of a condition called "L5 radiculopathy." Radiculopathy refers to compression or irritation of a spinal nerve root.  In this case, the L5 nerve root, which emerges from the spinal cord in the lower back (lumbar region), is likely affected. The symptoms of L5 radiculopathy typically include pain and sensory changes along the distribution of the L5 nerve, which includes the lateral leg and the top of the foot, particularly the great toe.  Options A (L1), B (L3), and D (S1) do not typically present with the specific combination of symptoms described in the case. Each nerve root is associated with its own distinct pattern of pain and sensory changes, and in this case, the involvement of L5 is most likely. However, a thorough neurological examination and possibly imaging studies (e.g., MRI) would be required to confirm the diagnosis and identify the exact cause of the nerve root compression or irritation. |
|  |  | Attempt 3  Based on the given information, the most likely nerve root affected in the 46-year-old man with pain in the left leg, tingling in the left big toe, severe lower back pain, inability to walk on the left heel, and loss of pinprick perception over the left great toe is D. S1.  The symptoms described suggest a clinical picture consistent with a radiculopathy, which is a condition characterized by compression or irritation of a spinal nerve root. The distribution of symptoms in the left leg, including the left big toe, lower back pain, and inability to walk on the left heel, is indicative of involvement of the S1 nerve root.  The S1 nerve root is responsible for innervating specific areas of the leg and foot, including the outer side of the foot and the little toe. The loss of pinprick perception over the left great toe further supports the involvement of the S1 nerve root.  Options A (L1), B (L3), and C (L5) are less likely to be affected based on the given symptoms. The clinical findings in this case, such as the distribution of symptoms and loss of pinprick perception over the left great toe, are more consistent with S1 nerve root involvement.  It's important to note that a comprehensive evaluation by a healthcare professional, such as a physician or neurologist, should be undertaken to confirm the diagnosis and determine the appropriate management for the patient. |
| **6** | A 65 year old man attends the anticoagulant clinic. He has had a metal mitral  valve replacement and atrial fibrillation. He takes warfarin 7 mg daily.  There are no signs of bleeding. His pulse rate is 70 bpm, irregularly irregular,  with a mechanical second heart sound. His INR is 5.1.  Which is the most appropriate next step in management?  A. Continue warfarin at lower dose  B. Continue warfarin at same dose  C. Give vitamin K intravenously  D. Give vitamin K orally  E. Withhold warfarin for 2 days then restart at lower dose | Attempt 1  Based on the provided information, the most appropriate next step in management for the 65-year-old man with a metal mitral valve replacement, atrial fibrillation, pulse rate of 70 bpm, irregularly irregular rhythm, mechanical second heart sound, and an INR of 5.1 is C. Give vitamin K intravenously.  The international normalized ratio (INR) is a measure of blood clotting time and is used to monitor the effectiveness of warfarin therapy. The target INR for patients with mechanical heart valves is typically higher than for other indications, usually around 2.5-3.5. An INR of 5.1 is significantly above the target range, indicating a higher risk of bleeding complications.  In this scenario, the most appropriate next step is to administer vitamin K intravenously. Intravenous administration of vitamin K will more rapidly lower the INR and reduce the risk of bleeding. The patient's high INR likely reflects excessive anticoagulation, which needs to be corrected promptly to minimize bleeding risk.  The other options are less appropriate:  A. Continuing warfarin at a lower dose would not be the best course of action in this case, as the INR is significantly elevated, and the patient is at a higher risk of bleeding. Simply lowering the warfarin dose may not effectively address the immediate need for INR correction.  B. Continuing warfarin at the same dose would not be appropriate since the INR is already elevated and requires intervention to reduce the risk of bleeding.  D. Giving vitamin K orally would be less effective than intravenous administration in rapidly reversing the anticoagulant effect of warfarin. Oral vitamin K has a delayed onset of action and is not the optimal choice in this situation.  E. Withholding warfarin for two days and then restarting at a lower dose may be an option in some cases of mildly elevated INR, but in this situation with a significantly elevated INR and higher bleeding risk, more immediate action is required.  It's important for the patient to consult with a healthcare professional, preferably the anticoagulation clinic or a specialist managing their anticoagulation therapy, for further guidance and monitoring of anticoagulation status. |
|  |  | Attempt 2  The most appropriate next step in management for this patient with an INR of 5.1 is:  E. Withhold warfarin for 2 days then restart at a lower dose.  In this scenario, the patient has an elevated INR of 5.1, which indicates that his blood is too thin, and there is an increased risk of bleeding. Given that the patient has a mechanical mitral valve replacement and atrial fibrillation, maintaining appropriate anticoagulation is essential to prevent thromboembolic events (blood clots) associated with the mechanical valve.  Withholding warfarin for 2 days allows the INR to naturally decrease, and then restarting at a lower dose may help achieve the target therapeutic range without exposing the patient to the potential risks associated with immediate reversal using vitamin K.  However, it is important to note that this approach carries a risk of prolonged exposure to an elevated INR, which may not be ideal for patients with mechanical heart valves. The decision to withhold and restart warfarin should be made with careful consideration of the patient's individual risks and benefits. Frequent monitoring and close follow-up are essential during this period to ensure that the INR remains within the desired therapeutic range and to prevent complications. The final decision should be made by the attending healthcare professionals based on the patient's clinical condition and risk profile. |
|  |  | Attempt 3  Based on the given information, the most appropriate next step in management for the 65-year-old man with a metal mitral valve replacement, atrial fibrillation, an irregularly irregular pulse rate, a mechanical second heart sound, and an INR of 5.1 is C. Give vitamin K intravenously.  The international normalized ratio (INR) is a measure of the blood's ability to clot, with a target range typically set for patients on anticoagulation therapy. In this case, the patient's INR of 5.1 indicates that his blood is more anticoagulated than desired, putting him at an increased risk of bleeding complications.  Given that the patient has a mechanical mitral valve and atrial fibrillation, it is crucial to maintain an appropriate level of anticoagulation to reduce the risk of thromboembolic events (blood clots). However, an INR of 5.1 is considered excessively high.  The most appropriate next step in this scenario is to give vitamin K intravenously to reverse the anticoagulant effects of warfarin. Intravenous vitamin K has a rapid onset of action and can help lower the INR more quickly compared to oral administration.  Options A and B, continuing warfarin at a lower or the same dose, would not be appropriate in this case as the patient's INR is already elevated, indicating excess anticoagulation. Continuing at the same dose or reducing the dose could further increase the risk of bleeding.  Option D, giving vitamin K orally, is generally reserved for cases where the INR is only slightly elevated and there is no active bleeding. In this case, with an INR of 5.1, intravenous administration is more suitable.  Option E, withholding warfarin for 2 days and then restarting at a lower dose, may be considered in some situations to reduce the anticoagulant effect. However, given the patient's mechanical mitral valve and atrial fibrillation, maintaining an appropriate level of anticoagulation is crucial, and the immediate effect of intravenous vitamin K is preferred.  Therefore, based on the provided information, the most appropriate next step in management for the 65-year-old man is C. Give vitamin K intravenously. The patient should be closely monitored for any signs of bleeding or further coagulation derangement, and adjustments to the anticoagulation regimen should be made in consultation with a healthcare professional. |
| **7** | A 52 year old man has three days of severe epigastric pain, radiating to his  back, but no chest pain. He has vomited several times. He was previously  well. He drinks approximately 60 units of alcohol a week and smokes 20  cigarettes per day.  There is epigastric tenderness but his abdomen is not distended, and bowel  sounds are present.  Which test would confirm the most likely diagnosis?  A. Abdominal X-ray  B. Gastroduodenoscopy  C. Serum alkaline phosphatase concentration  D. Serum amylase concentration  E. Ultrasound scan of abdomen | Attempt 1  Based on the provided information, the test that would confirm the most likely diagnosis in the 52-year-old man with severe epigastric pain radiating to the back, vomiting, alcohol consumption, and smoking history is D. Serum amylase concentration.  The clinical presentation described is highly suggestive of acute pancreatitis, which is inflammation of the pancreas. Severe epigastric pain radiating to the back, vomiting, and tenderness in the epigastric region are classic symptoms of acute pancreatitis.  Serum amylase concentration is a key laboratory test for the diagnosis of acute pancreatitis. Elevated levels of serum amylase are commonly seen in the early stages of acute pancreatitis and can help confirm the diagnosis.  The other options can be ruled out based on the given information:  A. Abdominal X-ray: An abdominal X-ray is not typically useful for confirming the diagnosis of acute pancreatitis. It may be performed in some cases to rule out other causes of abdominal pain or to assess for complications, but it would not confirm the diagnosis.  B. Gastroduodenoscopy: Gastroduodenoscopy (also known as upper endoscopy) is a procedure used to visualize the upper gastrointestinal tract, including the esophagus, stomach, and duodenum. While it may be performed in certain cases to evaluate the upper digestive tract, it is not the primary test for diagnosing acute pancreatitis.  C. Serum alkaline phosphatase concentration: Serum alkaline phosphatase is not specific to acute pancreatitis and is not the primary test used for its diagnosis. It may be elevated in various liver and bone conditions, among others.  E. Ultrasound scan of the abdomen: An ultrasound scan of the abdomen can be helpful in the evaluation of acute pancreatitis, especially in assessing for gallstones or complications such as fluid collections or pseudocysts. However, the primary initial test for confirming the diagnosis of acute pancreatitis is serum amylase concentration.  It's important for the patient to consult with a healthcare professional, preferably a gastroenterologist or a specialist experienced in pancreatitis, for further evaluation, management, and treatment of acute pancreatitis. |
|  |  | Attempt 2  The most likely diagnosis for the 52-year-old man with severe epigastric pain radiating to his back, vomiting, a history of heavy alcohol consumption, and smoking is acute pancreatitis. To confirm the diagnosis, the most appropriate test would be:  D. Serum amylase concentration  Acute pancreatitis is an inflammatory condition of the pancreas that can be caused by various factors, including heavy alcohol consumption and gallstones. Elevated levels of serum amylase and lipase, both pancreatic enzymes, are characteristic of acute pancreatitis.  Option A (Abdominal X-ray) is not the most appropriate test for diagnosing acute pancreatitis. An abdominal X-ray may be helpful in evaluating other conditions, but it does not directly confirm pancreatitis.  Option B (Gastroduodenoscopy) may be useful in assessing the upper gastrointestinal tract, but it is not the primary test to confirm acute pancreatitis.  Option C (Serum alkaline phosphatase concentration) is not specific for pancreatitis and is more often associated with liver conditions.  Option E (Ultrasound scan of the abdomen) can be useful in evaluating the pancreas and other abdominal organs. While it may show signs of pancreatitis, it may not be as sensitive or specific as serum amylase concentration for confirming the diagnosis.  In this case, the most appropriate initial test to confirm the most likely diagnosis of acute pancreatitis is the measurement of serum amylase concentration. Additional tests, such as serum lipase and imaging studies like ultrasound or CT scan, may be ordered to further assess the severity and potential complications of pancreatitis. The patient should also receive supportive care, including pain management, fluid resuscitation, and addressing the underlying risk factors such as alcohol consumption. |
|  |  | Attempt 3  Based on the given information, the most appropriate test to confirm the most likely diagnosis in the 52-year-old man with severe epigastric pain, radiating to the back, vomiting, and a history of heavy alcohol consumption and smoking is D. Serum amylase concentration.  The patient's symptoms, including severe epigastric pain radiating to the back, vomiting, and a history of heavy alcohol consumption, raise suspicion for acute pancreatitis. Acute pancreatitis is often characterized by sudden-onset severe abdominal pain, commonly located in the epigastric region and may radiate to the back. Vomiting is also a common symptom.  Serum amylase concentration is a specific laboratory test used in the diagnosis of acute pancreatitis. In cases of acute pancreatitis, serum amylase levels are typically elevated. However, it is important to note that serum amylase levels can also be elevated in other conditions, such as salivary gland disease and renal impairment. Therefore, additional clinical information and further investigations may be required to establish the definitive diagnosis.  Options A (abdominal X-ray), C (serum alkaline phosphatase concentration), and E (ultrasound scan of the abdomen) are not the most appropriate tests for confirming the diagnosis of acute pancreatitis. Abdominal X-rays are generally not useful for diagnosing pancreatitis, and serum alkaline phosphatase concentration is not a specific marker for pancreatitis. While an ultrasound scan of the abdomen (option E) can provide additional information and help evaluate the pancreas, it is not the initial test of choice to confirm the diagnosis of acute pancreatitis.  Gastroduodenoscopy (option B), also known as an upper endoscopy, is typically used to evaluate the upper gastrointestinal tract and is not the initial test of choice for diagnosing acute pancreatitis. It may be considered in specific cases if there is suspicion of other gastrointestinal pathology or to evaluate the extent of pancreatitis, but serum amylase concentration is a more specific and readily available test.  Therefore, based on the provided information, the most appropriate test to confirm the most likely diagnosis of acute pancreatitis in the 52-year-old man is D. Serum amylase concentration. Additional investigations and management would be determined based on the clinical findings and severity of the condition. |
| **8** | A 24 year old man attends the Emergency Department after 2 days of  vomiting. He has type 1 diabetes. He is drowsy but maintaining his airway. His  pulse rate is 100 bpm, BP 90/60 mmHg, respiratory rate 30 breaths per  minute and oxygen saturation 96% breathing air.  Investigations:  Blood capillary glucose 32 mmol/L  Blood capillary ketones 6.2 mmol/L (<0.6)  Venous pH 7.15 (7.35–7.45)  Which is the most appropriate initial treatment?  A. Intravenous 0.9% sodium chloride  B. Intravenous 1.26% sodium bicarbonate  C. Intravenous antibiotics  D. Intravenous insulin  E. Subcutaneous insulin | Attempt 1  Based on the provided information, the most appropriate initial treatment for the 24-year-old man with type 1 diabetes, drowsiness, elevated blood glucose, elevated ketones, and metabolic acidosis is D. Intravenous insulin.  The patient is presenting with diabetic ketoacidosis (DKA), a serious complication of uncontrolled diabetes characterized by hyperglycemia, ketosis, and metabolic acidosis. DKA can lead to severe dehydration, electrolyte imbalances, and can be life-threatening if left untreated.  The initial treatment for DKA involves addressing the underlying metabolic abnormalities. Intravenous insulin administration is essential to lower blood glucose levels and suppress ketone production. Insulin is typically administered intravenously as a continuous infusion to achieve a gradual reduction in blood glucose and ketone levels.  The other options can be ruled out based on the given information:  A. Intravenous 0.9% sodium chloride: Intravenous fluid resuscitation is an important component of DKA management to correct dehydration and electrolyte imbalances. However, intravenous fluid alone is not sufficient and should be combined with insulin therapy.  B. Intravenous 1.26% sodium bicarbonate: Sodium bicarbonate administration is generally reserved for severe cases of metabolic acidosis with pH <7.0. In this case, the patient's pH is 7.15, which does not indicate an immediate need for sodium bicarbonate therapy.  C. Intravenous antibiotics: The information provided does not suggest the need for intravenous antibiotics. Antibiotics are typically administered in DKA only if there is evidence of infection, which is not mentioned in the scenario.  E. Subcutaneous insulin: Subcutaneous insulin is not the appropriate initial treatment for DKA because it has slower absorption and onset of action compared to intravenous insulin. Intravenous insulin allows for more rapid and precise control of blood glucose levels.  It is important for the patient to be managed in a hospital setting, preferably in an intensive care unit or a specialized diabetes care unit, where close monitoring and further interventions can be provided, including intravenous fluids, electrolyte correction, and frequent monitoring of blood glucose, ketones, electrolytes, and acid-base status.  Prompt medical attention and treatment are necessary for DKA, and the patient's condition warrants urgent evaluation and management by healthcare professionals experienced in diabetic emergencies. |
|  |  | Attempt 2  The most appropriate initial treatment for the 24-year-old man with type 1 diabetes, drowsiness, high blood glucose, elevated ketones, and metabolic acidosis (low pH) is:  A. Intravenous 0.9% sodium chloride  The patient is presenting with diabetic ketoacidosis (DKA), which is a severe and life-threatening complication of uncontrolled diabetes. DKA is characterized by hyperglycemia, ketosis (elevated ketones), and metabolic acidosis due to insulin deficiency. The initial management of DKA involves fluid resuscitation with intravenous 0.9% sodium chloride to correct dehydration and restore blood pressure and perfusion.  Option B (Intravenous 1.26% sodium bicarbonate) is not the first-line treatment for DKA. Bicarbonate therapy is generally not recommended unless the pH is extremely low (<6.9), as rapid correction of acidosis can lead to adverse effects.  Option C (Intravenous antibiotics) is not indicated as DKA is not caused by infection.  Option D (Intravenous insulin) is a crucial part of DKA management, but it should be started after initial fluid resuscitation with saline. Insulin helps to reverse the ketosis and hyperglycemia.  Option E (Subcutaneous insulin) is not appropriate for the initial treatment of DKA, as it would be too slow to correct the severe metabolic derangements.  The priority in managing this patient is to address the dehydration and metabolic acidosis with fluid resuscitation using intravenous 0.9% sodium chloride. Insulin therapy should be initiated once the patient's fluid status has been stabilized. The patient will likely require hospital admission and close monitoring to correct the DKA and its underlying causes effectively. |
|  |  | Attempt 3  Based on the given information, the most appropriate initial treatment for the 24-year-old man with type 1 diabetes, drowsiness, vomiting, elevated blood glucose, ketones, and metabolic acidosis is D. Intravenous insulin.  The patient's presentation suggests diabetic ketoacidosis (DKA), a life-threatening complication of uncontrolled diabetes characterized by hyperglycemia, ketosis, and metabolic acidosis. DKA is commonly associated with type 1 diabetes and can occur due to insulin deficiency.  Intravenous insulin is the cornerstone of treatment for DKA. It helps lower blood glucose levels, suppresses ketone production, and corrects metabolic acidosis. Intravenous administration allows for precise control and adjustment of insulin infusion based on frequent monitoring of blood glucose and ketone levels.  Options A (intravenous 0.9% sodium chloride), B (intravenous 1.26% sodium bicarbonate), and C (intravenous antibiotics) are not the most appropriate initial treatments for DKA.  Intravenous 0.9% sodium chloride (option A) is often used as part of the treatment for DKA to correct dehydration and electrolyte imbalances. However, it does not address the underlying problem of insulin deficiency and metabolic acidosis.  Intravenous 1.26% sodium bicarbonate (option B) is generally reserved for severe cases of metabolic acidosis with pH below 7.0. In this case, the patient's pH is 7.15, which does not meet the criteria for administration of sodium bicarbonate.  Intravenous antibiotics (option C) are not indicated as the primary treatment for DKA. Antibiotics may be considered if there is a suspicion of infection contributing to the development of DKA, but it is not the initial treatment priority.  Subcutaneous insulin (option E) is not the most appropriate initial treatment for DKA because it may have delayed onset of action and unreliable absorption in the setting of vomiting and dehydration. Intravenous insulin is required to rapidly lower blood glucose and resolve metabolic acidosis.  Therefore, based on the provided information, the most appropriate initial treatment for the 24-year-old man with DKA is D. Intravenous insulin. Prompt medical attention is crucial in DKA cases, and the patient should be closely monitored and managed in an appropriate healthcare facility. |
| **9** | A 55 year old man is rescued from a collapsed building where he has been  trapped for 12 hours without water. His temperature is 35.6°C, pulse rate 100  bpm and BP 90/42 mmHg. His JVP is not visible. His abdomen is non tender.  Investigations:  Haemoglobin 168 g/L (130–175)  Sodium 148 mmol/L (135–146)  Potassium 6.0 mmol/L (3.5–5.3)  Urea 25.1 mmol/L (2.5–7.8)  Creatinine 184 μmol/L (60–120)  Creatine kinase 840 U/L (25–200)  Which is the most likely cause of this biochemical picture?  A. Bladder outflow obstruction  B. Direct renal trauma  C. Hypovolaemia  D. Rhabdomyolysis  E. Sepsis | Attempt 1  Based on the provided information, the most likely cause of the biochemical picture in the 55-year-old man who was trapped without water for 12 hours and presents with specific laboratory findings is C. Hypovolaemia.  The patient's presentation suggests hypovolemic shock, which occurs when there is inadequate circulating blood volume leading to impaired tissue perfusion. The following findings support this diagnosis:  - Low blood pressure (90/42 mmHg)  - Non-visible jugular venous pressure (JVP)  - Elevated hematocrit (hemoglobin 168 g/L), indicating hemoconcentration due to volume depletion  - Elevated urea (25.1 mmol/L) and creatinine (184 μmol/L), suggesting acute kidney injury due to reduced renal perfusion  The elevated potassium (6.0 mmol/L) may be a consequence of the release of intracellular potassium due to tissue ischemia and cellular damage associated with hypovolemic shock.  The other options can be ruled out based on the given information:  A. Bladder outflow obstruction: Bladder outflow obstruction would not typically cause this combination of laboratory findings.  B. Direct renal trauma: While direct renal trauma can cause kidney injury, the absence of trauma history and the presence of specific findings associated with hypovolemia make it less likely in this case.  D. Rhabdomyolysis: Rhabdomyolysis, which is the breakdown of skeletal muscle leading to the release of myoglobin and other intracellular contents, can result in elevated creatine kinase (CK) levels. However, the biochemical picture in this case is more consistent with hypovolemia rather than isolated rhabdomyolysis.  E. Sepsis: Sepsis is a systemic inflammatory response to infection and can cause multiple organ dysfunction. While it may result in kidney injury, the absence of signs of infection and the specific findings supporting hypovolemia make sepsis less likely in this scenario.  It is important for the patient to receive immediate resuscitation with intravenous fluids and further management in a hospital setting to address the hypovolemia and prevent complications associated with inadequate tissue perfusion.  Prompt medical attention and treatment are necessary, and the patient's condition warrants urgent evaluation and management by healthcare professionals experienced in managing shock and acute kidney injury. |
|  |  | Attempt 2  The most likely cause of this biochemical picture in the 55-year-old man who was trapped without water for 12 hours is:  C. Hypovolaemia  The patient's clinical presentation, including collapsed building incident, low body temperature (35.6°C), tachycardia (100 bpm), low blood pressure (90/42 mmHg), non-visible jugular venous pulse (JVP), and elevated urea and creatinine levels, is consistent with hypovolemia, which is severe fluid loss or decreased blood volume.  Hypovolemia can occur in situations of prolonged dehydration, such as being trapped without water for an extended period. The body tries to compensate for the low blood volume by increasing the heart rate and releasing antidiuretic hormone (ADH) to retain water. However, if not adequately treated, it can lead to severe metabolic derangements, including elevated urea and creatinine levels.  Option A (Bladder outflow obstruction) and Option B (Direct renal trauma) are less likely to cause this biochemical picture, as they do not typically lead to widespread fluid loss and dehydration.  Option D (Rhabdomyolysis) can cause elevated creatine kinase (CK) levels, but it does not explain the other abnormalities in this case, such as low body temperature, low blood pressure, and elevated urea.  Option E (Sepsis) can cause multiple organ dysfunction and deranged laboratory results, but it is less likely in this case, considering the patient's history of being trapped without water for 12 hours, which points more toward hypovolemia.  The most critical initial step in management is fluid resuscitation to correct the hypovolemia and restore organ perfusion. The patient may require intravenous fluids and careful monitoring to stabilize his hemodynamic status and improve the biochemical abnormalities. |
|  |  | Attempt 3  Based on the given information, the most likely cause of the biochemical picture in the 55-year-old man who was trapped without water for 12 hours, with a low body temperature, hypotension, non-visible jugular venous pressure (JVP), and abnormal laboratory values, is C. Hypovolaemia.  The patient's clinical presentation, including the history of being trapped without water for a prolonged period, low body temperature (35.6°C), hypotension (BP 90/42 mmHg), and non-visible JVP, suggests hypovolaemia. Hypovolaemia refers to a decreased blood volume and can lead to inadequate tissue perfusion and organ dysfunction.  The laboratory findings in this case further support the diagnosis of hypovolaemia. The elevated urea (25.1 mmol/L) and creatinine (184 μmol/L) levels indicate impaired kidney function due to reduced renal perfusion. The elevated potassium (6.0 mmol/L) and creatine kinase (840 U/L) levels are suggestive of tissue breakdown and release of intracellular contents, which can occur in conditions of reduced blood flow and prolonged ischemia.  Options A (bladder outflow obstruction), B (direct renal trauma), D (rhabdomyolysis), and E (sepsis) are less likely causes in this scenario. There is no indication or history of bladder outflow obstruction or direct renal trauma. While rhabdomyolysis can lead to elevated creatine kinase levels, it is unlikely to be the primary cause in this case without a history of significant muscle injury. Similarly, sepsis is less likely without any signs or symptoms suggestive of infection.  Therefore, based on the provided information, the most likely cause of the biochemical picture in the 55-year-old man is C. Hypovolaemia. Prompt fluid resuscitation and further management are crucial to restore adequate tissue perfusion and address the underlying cause of hypovolaemia. |
| **10** | A 24 year old woman has diarrhoea. She is HIV positive and has been  working in Namibia.  Investigation:  Faeces microscopy (following modified Ziehl–Neelsen stain): protozoa  Which is the most likely causative organism?  A. Acanthamoeba  B. Cryptosporidium parvum  C. Entamoeba coli  D. Plasmodium falciparum  E. Schistosoma mansoni | Attempt 1  Based on the provided information, the most likely causative organism for the woman's diarrhea, as detected on faeces microscopy following a modified Ziehl–Neelsen stain, is B. Cryptosporidium parvum.  Cryptosporidium parvum is a protozoan parasite that can cause gastrointestinal infections, particularly in individuals with weakened immune systems, such as those who are HIV positive. It is known to cause diarrheal illness, especially in immunocompromised individuals.  The other options can be ruled out based on the given information:  A. Acanthamoeba: Acanthamoeba is a protozoan organism that can cause various infections, including eye and central nervous system infections. It is not typically associated with gastrointestinal symptoms such as diarrhea.  C. Entamoeba coli: Entamoeba coli is a non-pathogenic amoeba commonly found in the human intestinal tract. It does not typically cause significant gastrointestinal symptoms or diarrhea.  D. Plasmodium falciparum: Plasmodium falciparum is a protozoan parasite responsible for causing malaria. Malaria is characterized by fever, chills, and other systemic symptoms, but it does not typically cause diarrhea.  E. Schistosoma mansoni: Schistosoma mansoni is a parasitic worm that causes schistosomiasis, a disease commonly associated with chronic inflammation of the liver, intestines, and other organs. While it can lead to gastrointestinal symptoms, including diarrhea, the detection of protozoa on microscopy suggests a different causative organism.  It's important for the patient to receive appropriate medical management and treatment for Cryptosporidium parvum infection. Antiparasitic medications may be prescribed, and supportive measures, such as fluid and electrolyte replacement, should be considered to manage the diarrhea and prevent dehydration.  The patient should be evaluated by a healthcare professional, preferably an infectious disease specialist or a healthcare provider experienced in managing HIV-related conditions, for further assessment, diagnosis confirmation, and appropriate treatment options. |
|  |  | Attempt 2  The most likely causative organism for the diarrhea in this 24-year-old HIV-positive woman who has been working in Namibia and has protozoa detected in her feces is:  B. Cryptosporidium parvum  Cryptosporidium parvum is a protozoan parasite that commonly causes diarrhea in immunocompromised individuals, such as those with HIV/AIDS. It is transmitted through contaminated food or water and is particularly prevalent in areas with poor sanitation.  Option A (Acanthamoeba) is a free-living amoeba that can cause infections, but it is not a common cause of diarrhea in this context.  Option C (Entamoeba coli) is a non-pathogenic amoeba and is not known to cause significant illness or diarrhea.  Option D (Plasmodium falciparum) is the causative organism of malaria, a mosquito-borne infectious disease, and it does not cause diarrhea.  Option E (Schistosoma mansoni) is a parasitic worm that causes schistosomiasis, a disease primarily affecting the liver and intestines. While schistosomiasis can cause abdominal symptoms, it is not commonly associated with diarrhea.  Given the patient's HIV-positive status and the detection of protozoa in her feces, Cryptosporidium parvum is the most likely causative organism responsible for her diarrhea. Treatment and management will likely involve addressing her immunocompromised state, supportive care, and specific treatment for Cryptosporidium infection. |
|  |  | Attempt 3  Based on the provided information, the most likely causative organism for the diarrhoea in the 24-year-old woman who is HIV positive and has been working in Namibia is B. Cryptosporidium parvum.  Cryptosporidium parvum is a protozoan parasite that can cause gastrointestinal infections, particularly in individuals with weakened immune systems, such as those with HIV/AIDS. It is a common cause of diarrhoea in HIV-positive individuals, especially in regions with poor sanitation and contaminated water sources, which may be relevant to the woman's history of working in Namibia.  The modified Ziehl-Neelsen stain is a laboratory technique used to detect acid-fast organisms, including Cryptosporidium parvum. The identification of protozoa on faecal microscopy with this stain suggests the presence of Cryptosporidium parvum as the causative organism.  Options A (Acanthamoeba), C (Entamoeba coli), D (Plasmodium falciparum), and E (Schistosoma mansoni) are less likely causative organisms in this scenario. Acanthamoeba is a rare cause of gastrointestinal infections and is not commonly associated with diarrhoea. Entamoeba coli is a non-pathogenic amoeba that is typically considered a commensal organism in the human gastrointestinal tract. Plasmodium falciparum is the causative organism of malaria and primarily affects the bloodstream, not the gastrointestinal tract. Schistosoma mansoni is a parasitic worm that causes schistosomiasis, a condition characterized by liver and intestinal involvement, but it does not typically present with diarrhoea as the main symptom.  Therefore, based on the provided information, the most likely causative organism for the woman's diarrhoea is B. Cryptosporidium parvum. Further evaluation, including appropriate management and treatment of the infection, should be conducted in consultation with a healthcare professional experienced in the management of HIV/AIDS-related conditions and tropical medicine. |
| **11** | A 67 year old man has difficulty chewing and speaking. He underwent carotid  surgery 2 days ago.  His tongue deviates to the right when he is asked to protrude it.  Which nerve has been damaged?  A. Left glossopharyngeal nerve  B. Left hypoglossal nerve  C. Left vagus nerve  D. Right glossopharyngeal nerve  E. Right hypoglossal nerve | Attempt 1  Based on the provided information, the nerve that is most likely to be damaged in the 67-year-old man with difficulty chewing, speaking, and deviation of the tongue to the right is B. Left hypoglossal nerve.  The hypoglossal nerve (cranial nerve XII) innervates the muscles responsible for the movement of the tongue. Damage to the hypoglossal nerve can result in weakness or paralysis of the muscles on one side of the tongue, leading to deviation of the tongue toward the affected side. In this case, the patient's tongue deviates to the right when protruded, indicating a left hypoglossal nerve injury.  The other options can be ruled out based on the given information:  A. Left glossopharyngeal nerve: The glossopharyngeal nerve (cranial nerve IX) is responsible for various functions related to the throat and tongue, including taste sensation and swallowing. It is not directly involved in tongue protrusion or the observed symptoms.  C. Left vagus nerve: The vagus nerve (cranial nerve X) is a mixed nerve responsible for various functions, including control of the muscles of the soft palate and pharynx, as well as parasympathetic innervation of the digestive system and other organs. It is not directly involved in tongue protrusion or the observed symptoms.  D. Right glossopharyngeal nerve: The glossopharyngeal nerve (cranial nerve IX) functions similarly to the left glossopharyngeal nerve and is not directly involved in tongue protrusion or the observed symptoms.  E. Right hypoglossal nerve: The right hypoglossal nerve is not likely to be damaged based on the symptoms described, as the deviation of the tongue to the right indicates an injury to the left side.  It is important for the patient to be evaluated by a healthcare professional, preferably a neurologist or a specialist experienced in cranial nerve disorders, for further assessment, confirmation of the diagnosis, and appropriate management of the hypoglossal nerve injury. |
|  |  | Attempt 2  The nerve that has most likely been damaged in this 67-year-old man with difficulty chewing and speaking and deviation of the tongue to the right when protruded after carotid surgery is:  B. Left hypoglossal nerve  The hypoglossal nerve (cranial nerve XII) is responsible for the motor innervation of the muscles of the tongue. Damage or injury to the left hypoglossal nerve can lead to weakness or paralysis of the left side of the tongue, causing it to deviate to the same side (left) when the patient attempts to protrude it.  Options A (Left glossopharyngeal nerve), C (Left vagus nerve), D (Right glossopharyngeal nerve), and E (Right hypoglossal nerve) are not the likely culprits in this case, as they are not responsible for the observed symptoms and findings.  The left hypoglossal nerve, which controls the muscles on the left side of the tongue, seems to be affected, leading to the tongue deviation and resulting in the patient's difficulty with chewing and speaking. The exact cause and extent of the nerve damage should be further evaluated and managed by the appropriate medical professionals to improve the patient's condition. |
|  |  | Attempt 3  Based on the provided information, the nerve that is most likely to have been damaged in the 67-year-old man who underwent carotid surgery and now presents with difficulty chewing, speaking, and deviation of the tongue to the right is B. Left hypoglossal nerve.  The hypoglossal nerve (cranial nerve XII) is responsible for the motor innervation of the muscles of the tongue. It controls the movements of the tongue, including protrusion, retraction, and side-to-side movements. Damage to the hypoglossal nerve can result in weakness or paralysis of the affected side of the tongue, leading to deviation towards the affected side when protruded.  In this case, since the tongue deviates to the right, it suggests damage to the left |
| **12** | An 18 year old woman has had 3 years of intermittent zig-zagging and  flashing lights in both eyes associated with headache. These episodes occur  2-3 times per month, last approximately half an hour, and are associated with  nausea and vomiting. Her vision is affected at the time of each episode but  returns to normal afterwards.  Which is the most likely diagnosis?  A. Acute glaucoma  B. Migraine  C. Occipital lobe epilepsy  D. Retinal detachment  E. Tension-type headache | Attempt 1  Based on the provided information, the most likely diagnosis for the 18-year-old woman experiencing intermittent zig-zagging and flashing lights in both eyes associated with headache is B. Migraine.  Migraine is a neurological condition characterized by recurrent episodes of moderate to severe headaches often associated with other symptoms such as visual disturbances, nausea, and vomiting. The specific visual disturbances described in this case, such as zig-zagging and flashing lights, are known as "migraine aura" and can occur before or during a migraine headache. The headache is typically unilateral, throbbing, and can be accompanied by sensitivity to light and sound.  The episodic nature of the symptoms (occurring 2-3 times per month), the duration of approximately half an hour, and the return to normal vision after each episode are consistent with the characteristics of a migraine attack.  The other options can be ruled out based on the given information:  A. Acute glaucoma: Acute glaucoma typically presents with sudden-onset severe eye pain, decreased vision, and halos around lights. It is not associated with zig-zagging and flashing lights, headache, or the described pattern of symptoms.  C. Occipital lobe epilepsy: Occipital lobe epilepsy can present with visual disturbances, but the duration of half an hour, the association with headache, and the presence of other migraine-like symptoms make migraine a more likely diagnosis in this case.  D. Retinal detachment: Retinal detachment typically presents with sudden-onset flashes of light or floaters, along with a curtain or shadow obscuring vision in one eye. It is unlikely to cause intermittent symptoms in both eyes as described.  E. Tension-type headache: Tension-type headaches are typically characterized by a bilateral, pressing or tightening headache without significant associated symptoms such as visual disturbances, nausea, or vomiting. The specific visual symptoms described in this case make migraine a more likely diagnosis.  It is important for the patient to undergo a thorough evaluation by a healthcare professional, preferably a neurologist or headache specialist, to confirm the diagnosis of migraine and discuss appropriate management strategies to help reduce the frequency and severity of her migraine attacks. |
|  |  | Attempt 2  The most likely diagnosis for the 18-year-old woman with 3 years of intermittent zig-zagging and flashing lights in both eyes associated with headache, occurring 2-3 times per month, lasting approximately half an hour, and associated with nausea and vomiting is:  B. Migraine  The symptoms described, including the visual disturbances (zig-zagging and flashing lights) known as an aura, headache, and associated nausea and vomiting, are classic features of a migraine with aura. Migraine is a neurological disorder characterized by recurrent episodes of moderate to severe headaches often accompanied by sensory disturbances, such as visual aura.  Acute glaucoma (Option A) typically presents with sudden-onset severe eye pain, decreased vision, and colored halos around lights, but it does not involve the described visual auras or the associated headache with nausea and vomiting.  Occipital lobe epilepsy (Option C) may cause visual disturbances, but it is not typically associated with headache or nausea and vomiting. Seizures from occipital lobe epilepsy usually last for a brief period (seconds to minutes) rather than half an hour.  Retinal detachment (Option D) presents with sudden-onset floaters, flashes of light, and a curtain-like shadow across the visual field. It is not typically associated with headache or a pattern of recurrent episodes.  Tension-type headache (Option E) may cause headache, but it does not typically involve visual disturbances like zig-zagging and flashing lights or the associated symptoms of nausea and vomiting.  Given the classic features of the episodes and the association of visual aura with headache and other symptoms, migraine is the most likely diagnosis. However, it is important for the patient to be evaluated by a healthcare professional to confirm the diagnosis and discuss appropriate management strategies. |
|  |  | Attempt 3  Based on the given information, the most likely diagnosis for the 18-year-old woman experiencing intermittent zig-zagging and flashing lights in both eyes associated with headaches is B. Migraine.  Migraine is a neurological condition characterized by recurrent episodes of headache, often accompanied by sensory disturbances called aura. Aura symptoms can include visual disturbances such as zig-zagging lines, flashing lights, and temporary vision loss. These symptoms typically precede or accompany the headache and can last anywhere from a few minutes to an hour.  The described episodes occurring 2-3 times per month, lasting approximately half an hour, and associated with nausea, vomiting, and visual disturbances are consistent with the typical features of a migraine with aura. The fact that the woman's vision returns to normal after each episode also supports the diagnosis of migraine.  Options A (Acute glaucoma), C (Occipital lobe epilepsy), D (Retinal detachment), and E (Tension-type headache) are less likely diagnoses based on the given information. Acute glaucoma typically presents with severe eye pain, decreased vision, and halos around lights, rather than intermittent zig-zagging and flashing lights associated with headaches. Occipital lobe epilepsy may present with visual disturbances, but the duration and characteristics of the episodes described are more consistent with migraine. Retinal detachment usually presents with sudden and painless vision |
| **13** | A 75 year old man has had 3 days of intermittent headaches, blurred vision  and vomiting. For the past 24 hours he has had a severe left sided headache  and eye pain, accompanied by blurred vision and vomiting. His left eye is red  and the left pupil is dilated.  Which investigation is most likely to confirm the diagnosis?  A. CT scan of head  B. Erythrocyte sedimentation rate  C. Fluorescein staining of the cornea  D. Measurement of intraocular pressure  E. MR scan of head | Attempt 1  Based on the provided information, the investigation that is most likely to confirm the diagnosis in the 75-year-old man with severe left-sided headache, eye pain, blurred vision, vomiting, redness in the left eye, and a dilated left pupil is A. CT scan of the head.  These symptoms are suggestive of a condition called acute angle-closure glaucoma, which is a medical emergency. In this condition, there is a sudden increase in intraocular pressure due to the blockage of the drainage angle in the eye, leading to a rapid onset of symptoms.  A CT scan of the head can help evaluate the structures of the eye, including the angle of the anterior chamber, to identify any anatomical abnormalities or signs of acute angle-closure glaucoma, such as ciliary body swelling or forward displacement of the lens-iris diaphragm.  The other options can be ruled out based on the given information:  B. Erythrocyte sedimentation rate: Erythrocyte sedimentation rate (ESR) is a non-specific marker of inflammation and is not specific for acute angle-closure glaucoma. It is typically used in the evaluation of inflammatory conditions.  C. Fluorescein staining of the cornea: Fluorescein staining of the cornea is used to assess corneal integrity and detect corneal abrasions or ulcers. It is not specific to acute angle-closure glaucoma.  D. Measurement of intraocular pressure: Measurement of intraocular pressure is an important diagnostic test for glaucoma, but in the case of acute angle-closure glaucoma, the intraocular pressure is typically significantly elevated. The clinical presentation described in the scenario, including a dilated pupil, redness in the left eye, and severe symptoms, suggests a more urgent need for imaging with a CT scan rather than a standalone measurement of intraocular pressure.  E. MR scan of the head: While an MRI scan of the head can provide detailed imaging of the structures of the eye and surrounding tissues, a CT scan is usually the preferred initial investigation in the case of acute angle-closure glaucoma due to its wider availability, faster imaging time, and ability to identify specific features of the condition.  It is critical for the patient to seek immediate medical attention and be evaluated by an ophthalmologist or an emergency healthcare professional to confirm the diagnosis and initiate appropriate management to relieve intraocular pressure and prevent further vision loss or complications associated with acute angle-closure glaucoma. |
|  |  | Attempt 2  The most likely investigation to confirm the diagnosis in the 75-year-old man with severe left-sided headache, eye pain, blurred vision, vomiting, red left eye, and a dilated left pupil is:  D. Measurement of intraocular pressure  Given the symptoms described, the most likely diagnosis is acute angle-closure glaucoma. This is a medical emergency caused by a sudden increase in intraocular pressure due to the blockage of the drainage angle in the eye, leading to optic nerve compression and potential vision loss.  Measurement of intraocular pressure (tonometry) is a crucial initial investigation in suspected cases of acute angle-closure glaucoma. It helps assess the level of pressure within the eye, which is typically significantly elevated in this condition.  While other investigations, such as a CT scan of the head (Option A) or an MR scan of the head (Option E), may be useful in evaluating the structures within the eye and the optic nerve, they may not be necessary initially for confirming the diagnosis of acute angle-closure glaucoma.  Option B (Erythrocyte sedimentation rate) is a marker of inflammation and is not specific to the diagnosis of acute angle-closure glaucoma.  Option C (Fluorescein staining of the cornea) is a test to assess corneal integrity and diagnose corneal abnormalities but is not specific to the diagnosis of acute angle-closure glaucoma.  In this scenario, measuring intraocular pressure is the most immediate and important investigation to determine the presence of acute angle-closure glaucoma. The patient should be urgently evaluated by an ophthalmologist or an eye care professional for appropriate management and treatment to reduce intraocular pressure and prevent further complications and vision loss. |
|  |  | Attempt 3  Based on the given information, the investigation most likely to confirm the diagnosis in the 75-year-old man with severe left-sided headache, eye pain, blurred vision, vomiting, red left eye, and dilated left pupil is A. CT scan of the head.  This presentation raises concern for a potentially serious condition known as acute angle-closure glaucoma, which is characterized by a sudden increase in intraocular pressure due to blockage of the drainage of aqueous humor in the eye. The symptoms described, including severe headache, eye pain, blurred vision, vomiting, redness of the eye, and a dilated pupil, are consistent with acute angle-closure glaucoma.  A CT scan of the head is the most appropriate investigation to confirm the diagnosis in this case. It can help evaluate the structures of the eye and detect any anatomical abnormalities or changes associated with acute angle-closure glaucoma.  Option B (Erythrocyte sedimentation rate) is not the most appropriate investigation for this presentation. Erythrocyte sedimentation rate is a non-specific marker of inflammation and would not directly confirm the diagnosis of acute angle-closure glaucoma.  Option C (Fluorescein staining of the cornea) is used to assess the integrity of the cornea and detect corneal abnormalities or injuries, but it would not be the primary investigation to confirm acute angle-closure glaucoma.  Option D (Measurement of intraocular pressure) is commonly performed in the evaluation of glaucoma, but in this case, given the severity of the symptoms and the potential urgency of the situation, a more comprehensive imaging study (such as a CT scan) would be needed.  Option E (MR scan of the head) could provide valuable information, but it is not the most appropriate initial investigation in this acute setting. CT scan is generally preferred due to its wider availability, faster imaging time, and better visualization of bony structures.  Therefore, based on the given information, the investigation most likely to confirm the diagnosis in the 75-year-old man is A. CT scan of the head. |
| **14** | A 72 year old woman has had 4 months of progressive difficulty walking. She  describes numbness and tingling in her feet and has fallen on several  occasions.  On examination of her lower limbs, she has normal tone, moderate weakness  of ankle dorsiflexion and plantar flexion, normal knee jerks, but absent ankle  jerks and extensor plantars. Romberg's test is positive. She has reduced  vibration sense, and joint position sense is impaired up to the ankle joints.  Temperature and pinprick sensations are normal.  Which investigation is most likely to confirm the diagnosis?  A. HbA 1c  B. Serum folate  C. Serum protein electrophoresis  D. Serum vitamin B 12  E. Serum vitamin D | Attempt 1  Based on the provided information, the investigation that is most likely to confirm the diagnosis in the 72-year-old woman with progressive difficulty walking, numbness and tingling in her feet, absent ankle jerks, impaired joint position sense, and positive Romberg's test is D. Serum vitamin B12.  The clinical presentation described is consistent with a peripheral neuropathy, specifically a sensory ataxic neuropathy. This type of neuropathy can be caused by various factors, but one of the common causes, particularly in older individuals, is vitamin B12 deficiency.  Vitamin B12 deficiency can result from malabsorption, dietary insufficiency, or other underlying conditions affecting vitamin B12 absorption or metabolism. The deficiency leads to damage to the peripheral nerves, resulting in symptoms such as numbness, tingling, impaired sensation, and weakness. The positive Romberg's test indicates impaired proprioception, which is a characteristic finding in sensory ataxic neuropathy.  Serum vitamin B12 level measurement is an appropriate investigation to confirm the diagnosis and determine if vitamin B12 deficiency is contributing to the patient's symptoms. A low vitamin B12 level would support the diagnosis and indicate the need for treatment with vitamin B12 supplementation.  The other options can be ruled out based on the given information:  A. HbA1c: HbA1c is a marker of long-term glucose control and is used to assess diabetes management. It is not specific to the symptoms described and is unlikely to confirm the diagnosis in this case.  B. Serum folate: Folate deficiency can also cause neurological symptoms, but the clinical presentation described is more consistent with a sensory ataxic neuropathy, which is commonly associated with vitamin B12 deficiency. Vitamin B12 deficiency should be evaluated first before considering folate deficiency.  C. Serum protein electrophoresis: Serum protein electrophoresis is used to evaluate the pattern and quantity of proteins in the blood and is typically ordered to investigate certain blood disorders or monitor response to treatment. It is not specific to the symptoms described and is unlikely to confirm the diagnosis in this case.  E. Serum vitamin D: Vitamin D deficiency can cause various symptoms, but the clinical presentation described, with predominantly sensory symptoms and absent ankle jerks, is not characteristic of vitamin D deficiency. Vitamin B12 deficiency is a more likely cause based on the given information.  It is important for the patient to undergo further evaluation, including serum vitamin B12 level measurement, and consult with a healthcare professional, preferably a neurologist or a specialist in peripheral neuropathy, for accurate diagnosis and appropriate management of the underlying condition. |
|  |  | Attempt 2  Option A, HbA1c, is the most appropriate investigation to confirm the diagnosis because it can help assess the patient's glycemic control over the past 2 to 3 months. Elevated HbA1c levels are indicative of poorly controlled diabetes, which is a known risk factor for peripheral neuropathy.  The rationale for choosing HbA1c as the preferred investigation is as follows:  1. Age and risk factors: The patient is 72 years old, and diabetes becomes more prevalent with age. Additionally, diabetes is a significant risk factor for peripheral neuropathy, and given the patient's age, it is essential to rule out or identify diabetes as a potential contributing factor.  2. Symptoms: While peripheral neuropathy can present with numbness, tingling, and weakness in the extremities, these symptoms are also commonly seen in diabetic peripheral neuropathy. Therefore, evaluating the patient's glycemic control is crucial in this context.  3. Screening for diabetes: The presence of peripheral neuropathy can be an early sign of undiagnosed diabetes. Measuring HbA1c can help identify patients with diabetes who might not be aware of their condition, as it reflects average blood glucose levels over the past few months.  4. Differential diagnosis: There could be various causes of peripheral neuropathy, including metabolic, autoimmune, and infectious etiologies. Measuring HbA1c can aid in narrowing down the potential causes and guiding further investigations.  5. Treatment implications: If the HbA1c levels are elevated, it would be important to address the underlying diabetes as part of the treatment plan for peripheral neuropathy. Proper glycemic control can help prevent further nerve damage and potentially improve the patient's symptoms.  In conclusion, given the patient's age, symptoms, and the importance of ruling out diabetes as a potential cause of peripheral neuropathy, measuring HbA1c is the most appropriate investigation in this case. |
|  |  | Attempt 3  Based on the provided information, the investigation most likely to confirm the diagnosis in the 72-year-old woman with progressive difficulty walking, numbness, tingling in her feet, weakness of ankle dorsiflexion and plantar flexion, absent ankle jerks, extensor plantars, positive Romberg's test, reduced vibration sense, and impaired joint position sense up to the ankle joints is D. Serum vitamin B12.  The clinical presentation described suggests a peripheral neuropathy, which can be caused by various factors. One of the common causes of peripheral neuropathy is vitamin B12 deficiency, especially in older adults. Vitamin B12 deficiency can result from malabsorption, dietary insufficiency, or other underlying medical conditions.  The woman's symptoms, including difficulty walking, numbness, tingling, muscle weakness, and impaired joint position sense, are consistent with a peripheral neuropathy related to vitamin B12 deficiency. Vitamin B12 is essential for proper neurological function, and its deficiency can lead to peripheral neuropathy, among other neurological manifestations.  Therefore, measuring the serum vitamin B12 level would be the most appropriate investigation to confirm the diagnosis in this case. A low serum vitamin B12 level would support the diagnosis of vitamin B12 deficiency as the underlying cause of the woman's symptoms.  Option A (HbA1c) is a blood test used to assess long-term blood sugar control and is not directly related to the symptoms and examination findings described in this case.  Option B (Serum folate) is less likely to be the cause of the woman's symptoms because folate deficiency typically presents with different neurological manifestations and does not typically cause the characteristic peripheral neuropathy seen in vitamin B12 deficiency.  Option C (Serum protein electrophoresis) is not the most appropriate investigation in this scenario. While it may help evaluate certain conditions associated with peripheral neuropathy, such as monoclonal gammopathy, it is not the initial investigation of choice.  Option E (Serum vitamin D) is not directly related to the symptoms and examination findings described in this case. Vitamin D deficiency primarily affects bone health and is not a common cause of peripheral neuropathy.  Therefore, based on the provided information, the investigation most likely to confirm the diagnosis is D. Serum vitamin B12. |
| **15** | N/A | Attempt 1 |
|  |  | Attempt 2 |
|  |  | Attempt 3 |
| **16** | A 29 year old woman has 2 days of marked loss of vision and acute pain in  her left eye. The pain is worse when she changes her gaze direction.  Her eyes appear normal on general inspection. Her vision is 'count fingers  only' in the affected eye. The swinging flashlight test shows that the left pupil  dilates when a bright light is moved from the right eye to the left eye. The optic  discs are normal on fundoscopy.  Which is the most likely diagnosis?  A. Acute closed angle glaucoma  B. Giant cell arteritis  C. Idiopathic intracranial hypertension  D. Migraine with aura  E. Retrobulbar optic neuritis | Attempt 1  Based on the provided information, the most likely diagnosis in the 29-year-old woman with marked loss of vision, acute pain in the left eye, worsened pain with gaze changes, and a relative afferent pupillary defect (RAPD) on the swinging flashlight test is E. Retrobulbar optic neuritis.  Retrobulbar optic neuritis is an inflammatory condition that affects the optic nerve behind the eye. It often presents with acute pain in or around the eye, accompanied by rapid loss of vision. Pain with eye movement, known as pain on gaze, is a characteristic feature of retrobulbar optic neuritis. The presence of a RAPD, where the affected eye dilates when a bright light is moved from the unaffected eye to the affected eye, is consistent with optic nerve dysfunction.  The absence of abnormal findings on general inspection of the eyes and normal optic discs on fundoscopy also support the diagnosis of retrobulbar optic neuritis. In this condition, the optic nerve inflammation occurs behind the eye, leading to normal-appearing optic discs on fundoscopy.  The other options can be ruled out based on the given information:  A. Acute closed-angle glaucoma: Acute closed-angle glaucoma typically presents with a sudden onset of severe eye pain, blurred vision, and a red, congested eye. It is often associated with a fixed, mid-dilated pupil, elevated intraocular pressure, and a shallow anterior chamber. These findings are not described in the case, making acute closed-angle glaucoma an unlikely diagnosis.  B. Giant cell arteritis: Giant cell arteritis (temporal arteritis) is an inflammatory condition affecting the blood vessels, particularly in the head and neck. It can cause vision loss, often in the setting of severe headache, jaw pain, and systemic symptoms. However, it is more common in older individuals, typically over the age of 50. Additionally, the swinging flashlight test is not typically abnormal in giant cell arteritis. Given the patient's age and clinical presentation, giant cell arteritis is less likely.  C. Idiopathic intracranial hypertension: Idiopathic intracranial hypertension (pseudotumor cerebri) is a condition characterized by increased intracranial pressure without an identifiable cause. It can cause headache, transient visual obscurations, and papilledema (swelling of the optic discs). In this case, the patient's optic discs are described as normal, making idiopathic intracranial hypertension less likely.  D. Migraine with aura: Migraine with aura is a neurological condition characterized by recurrent episodes of headache, often preceded by visual disturbances or other sensory symptoms (aura). However, the presence of acute pain in the eye, marked vision loss, and a RAPD are not typical features of migraine with aura. Therefore, this diagnosis is less likely.  It is important for the patient to seek immediate medical attention and be evaluated by an ophthalmologist or an emergency healthcare professional to confirm the diagnosis of retrobulbar optic neuritis and initiate appropriate management, which may include further investigations (such as MRI of the brain and orbits) and treatment with corticosteroids to reduce inflammation and promote recovery of vision. |
|  |  | Attempt 2  The most likely diagnosis in this case is:  E. Retrobulbar optic neuritis  The patient's symptoms of marked loss of vision and acute pain in the left eye, worsened by changes in gaze direction, are suggestive of optic neuritis. Optic neuritis is an inflammatory condition affecting the optic nerve and can cause vision loss and eye pain.  The swinging flashlight test shows an afferent pupillary defect (the left pupil dilates when light is moved from the right eye to the left eye). This finding indicates dysfunction of the left optic nerve, which is consistent with optic neuritis.  Acute closed-angle glaucoma (A) is a condition characterized by a sudden increase in intraocular pressure, causing severe eye pain, redness, and blurred vision. However, it typically presents with a fixed, mid-dilated pupil and does not cause a relative afferent pupillary defect (swinging flashlight test).  Giant cell arteritis (B) is an inflammatory condition affecting medium and large arteries, often involving the temporal artery. It can cause sudden vision loss, but it is more common in older individuals, especially over the age of 50. The patient in this case is 29 years old, making giant cell arteritis less likely.  Idiopathic intracranial hypertension (C) is a condition characterized by increased intracranial pressure without an identifiable cause. It can cause vision problems, including transient vision loss, but it typically presents with bilateral symptoms and may be associated with papilledema (swelling of the optic discs). In this case, the optic discs are described as normal on fundoscopy, making idiopathic intracranial hypertension less likely.  Migraine with aura (D) is a type of migraine headache that may be associated with visual disturbances (aura) before the headache phase. However, the acute loss of vision and severe eye pain in this case are not typical of migraine with aura.  Therefore, based on the clinical presentation and findings, retrobulbar optic neuritis (E) is the most likely diagnosis. Prompt evaluation and treatment with corticosteroids are often necessary to manage optic neuritis and preserve vision. |
|  |  | Attempt 3  Based on the provided information, the most likely diagnosis for the 29-year-old woman with marked loss of vision, acute pain in her left eye, pain worsening with changes in gaze direction, "count fingers only" vision in the affected eye, left pupil dilation in response to the swinging flashlight test, normal optic discs on fundoscopy, and normal general inspection of the eyes is E. Retrobulbar optic neuritis.  Retrobulbar optic neuritis is characterized by inflammation of the optic nerve behind the eyeball, typically resulting in acute pain, loss of vision, and impaired color vision. The pain may worsen with eye movement. Visual acuity is often significantly affected, as indicated by the patient's ability to only count fingers in the affected eye. The swinging flashlight test reveals a relative afferent pupillary defect (RAPD), where the affected pupil dilates when light is shone in that eye, indicating a dysfunction of the optic nerve. Fundoscopy reveals normal optic discs in retrobulbar optic neuritis because the inflammation is primarily affecting the portion of the optic nerve behind the eye.  Option A (Acute closed-angle glaucoma) is less likely because the patient's symptoms and examination findings are not consistent with the characteristic features of acute closed-angle glaucoma, such as a sudden increase in intraocular pressure, severe eye pain, decreased vision, and a mid-dilated and non-reactive pupil.  Option B (Giant cell arteritis) typically presents in older individuals and is associated with systemic symptoms such as headache, jaw claudication, and temporal artery tenderness. Fundoscopy may reveal ischemic optic neuropathy characterized by swollen optic discs. In this case, the patient is younger and lacks the systemic symptoms and fundoscopic findings suggestive of giant cell arteritis.  Option C (Idiopathic intracranial hypertension), also known as pseudotumor cerebri, typically presents with symptoms of increased intracranial pressure, such as headache, transient visual obscurations, and papilledema (swelling of the optic disc) on fundoscopy. The patient's presentation is more consistent with retrobulbar optic neuritis rather than idiopathic intracranial hypertension.  Option D (Migraine with aura) typically presents with visual disturbances, but the sudden loss of vision, |
| **17** | N/A | Attempt 1 |
|  |  | Attempt 2 |
|  |  | Attempt 3 |
| **18** | A 48 year old woman has rheumatoid arthritis. She takes regular paracetamol  and has no drug allergies. She is due to commence methotrexate weekly.  Which additional treatment should be prescribed?  A. Calcium carbonate  B. Folic acid  C. Pyridoxine hydrochloride  D. Thiamine  E. Vitamin D | Attempt 1  When initiating methotrexate therapy for rheumatoid arthritis, it is recommended to prescribe additional treatment with B. Folic acid.  Methotrexate is an immunosuppressive medication commonly used in the treatment of rheumatoid arthritis. It works by inhibiting the activity of certain enzymes involved in the immune system response. However, methotrexate can also interfere with the body's utilization of folate (a form of vitamin B9), leading to potential side effects such as mouth sores, gastrointestinal disturbances, and hematological abnormalities.  To mitigate these side effects and ensure optimal efficacy of methotrexate, folic acid supplementation is typically prescribed. Folic acid is a synthetic form of folate that helps replenish the body's folate levels, minimizing the adverse effects of methotrexate on folate metabolism.  The other options listed are not specifically indicated as additional treatment when starting methotrexate for rheumatoid arthritis:  A. Calcium carbonate: Calcium carbonate is commonly used as a calcium supplement and is not directly related to the use of methotrexate or rheumatoid arthritis treatment.  C. Pyridoxine hydrochloride: Pyridoxine hydrochloride, also known as vitamin B6, is not routinely prescribed as additional treatment when initiating methotrexate therapy for rheumatoid arthritis.  D. Thiamine: Thiamine, also known as vitamin B1, is not typically prescribed as additional treatment when starting methotrexate for rheumatoid arthritis.  E. Vitamin D: While vitamin D supplementation is often recommended for individuals with rheumatoid arthritis due to its potential benefits for bone health and immune function, it is not specifically indicated as additional treatment when initiating methotrexate therapy.  It is important for the patient to follow their healthcare provider's instructions regarding methotrexate dosing, folic acid supplementation, and regular monitoring to ensure optimal management of rheumatoid arthritis and minimize potential side effects. |
|  |  | Attempt 2  B. Folic acid  When starting methotrexate therapy for rheumatoid arthritis, it is common practice to prescribe folic acid as an additional treatment. Methotrexate is an immunosuppressive medication used to treat inflammatory conditions like rheumatoid arthritis. However, it can also interfere with folate metabolism, leading to potential side effects such as mouth sores, gastrointestinal upset, and liver enzyme abnormalities.  To reduce these side effects, folic acid is co-prescribed with methotrexate. Folic acid supplementation helps mitigate the adverse effects of methotrexate while maintaining its therapeutic benefits for rheumatoid arthritis. The usual dosing of folic acid is once weekly, except on the same day as the methotrexate dose, to avoid interference with its efficacy. This approach helps strike a balance between managing the side effects and achieving the desired therapeutic outcome with methotrexate treatment. |
|  |  | Attempt 3  When initiating treatment with methotrexate in a patient with rheumatoid arthritis, it is important to prescribe additional medications to mitigate potential side effects and optimize the effectiveness of the treatment. In this case, the most appropriate additional treatment to be prescribed is B. Folic acid.  Methotrexate is an immunosuppressive medication commonly used in the treatment of rheumatoid arthritis. However, it can deplete the body's folate levels, leading to side effects such as gastrointestinal symptoms, mouth ulcers, and hair loss. By prescribing folic acid, a B vitamin, alongside methotrexate, these side effects can be minimized or prevented.  Folic acid supplementation is typically started at a dose of 5 to 10 mg per week, given 24 to 48 hours after the methotrexate dose. This timing helps to reduce the interference with the effectiveness of methotrexate while providing the necessary folate support to minimize side effects.  Option A (Calcium carbonate) is not directly related to the initiation of methotrexate in this case. Calcium supplementation may be considered in patients at risk of osteoporosis or with calcium deficiency, but it is not specifically indicated as an additional treatment when starting methotrexate.  Option C (Pyridoxine hydrochloride) and option D (Thiamine) are not directly indicated as additional treatments when starting methotrexate in rheumatoid arthritis. These vitamins are not typically prescribed in the context of methotrexate therapy.  Option E (Vitamin D) may be considered as a separate supplementation for patients with vitamin D |
| **19** | A 74 year old woman has 6 months of progressive weakness of her right leg  and 3 months of a similar problem on the left, resulting in several falls. She  has also noticed difficulty using her hands and can no longer fasten the  buttons on her clothes.  There is wasting of both legs and the hands, particularly the thenar  eminences. There is fasciculation in her right quadriceps. Tone is increased in  both legs, with brisk reflexes.  Which is the most likely diagnosis?  A. Chronic inflammatory demyelinating polyneuropathy  B. Motor neurone disease  C. Multiple sclerosis  D. Myasthenia gravis  E. Polymyositis | Attempt 1  The most likely diagnosis in this case is B. Motor neurone disease.  Motor neurone disease (MND), also known as amyotrophic lateral sclerosis (ALS), is a progressive neurodegenerative disorder that affects the motor neurons in the brain and spinal cord. It leads to weakness, wasting, and fasciculations of the affected muscles.  In this case, the patient's clinical presentation of progressive weakness in both legs and hands, along with wasting and fasciculations, is consistent with the characteristic features of motor neurone disease. The involvement of both upper and lower motor neurons, as evidenced by increased tone and brisk reflexes, further supports this diagnosis.  The other options listed are less likely based on the given clinical features:  A. Chronic inflammatory demyelinating polyneuropathy (CIDP): CIDP is a chronic immune-mediated neuropathy that typically presents with symmetrical sensory and motor dysfunction in the limbs, but it is less likely to cause the combination of upper and lower motor neuron signs seen in this patient.  C. Multiple sclerosis (MS): MS is a demyelinating disorder that primarily affects the central nervous system. It typically presents with episodes of neurological deficits that come and go (relapses and remissions) and often involves a variety of symptoms, including visual disturbances, sensory abnormalities, and motor weakness. However, the progressive and symmetric nature of the weakness, along with the absence of other typical MS features, makes it less likely in this case.  D. Myasthenia gravis (MG): MG is an autoimmune disorder characterized by muscle weakness and fatigue, often involving the muscles of the eyes and face. While it can cause generalized weakness, it typically does not present with the wasting and fasciculations seen in this patient.  E. Polymyositis: Polymyositis is an inflammatory muscle disorder that primarily affects the skeletal muscles, causing muscle weakness and fatigue. It is less likely in this case because of the combination of upper and lower motor neuron signs and the involvement of both the hands and legs, which are not typical features of polymyositis.  Motor neurone disease is a progressive and debilitating condition, and further evaluation by a neurologist is necessary for confirmation of the diagnosis and to discuss appropriate management and supportive care options for the patient. |
|  |  | Attempt 2  The most likely diagnosis in this case is:  B. Motor neurone disease  The clinical presentation of progressive weakness of the legs and hands, along with wasting, fasciculations, brisk reflexes, and increased tone, is highly suggestive of motor neurone disease (MND), also known as amyotrophic lateral sclerosis (ALS).  Motor neurone disease is a neurodegenerative disorder that affects the motor neurones (nerve cells) in the brain and spinal cord, leading to progressive weakness and wasting of muscles. Fasciculations (involuntary muscle twitches) are a characteristic feature of MND. The involvement of both upper and lower motor neurones results in increased tone and brisk reflexes.  The other options are less likely given the specific clinical findings:  A. Chronic inflammatory demyelinating polyneuropathy (CIDP) is a chronic immune-mediated disorder affecting peripheral nerves. It typically presents with symmetrical weakness and sensory symptoms in the limbs but does not cause the upper motor neurone signs seen in this case.  C. Multiple sclerosis (MS) is a demyelinating disease of the central nervous system, which can cause various neurological symptoms, including weakness. However, the combination of upper and lower motor neurone signs is not typical of MS.  D. Myasthenia gravis (MG) is an autoimmune disorder affecting neuromuscular transmission. It primarily causes weakness and fatigue of voluntary muscles, especially those involved in eye movements and facial expressions. It does not typically cause wasting, fasciculations, or increased tone.  E. Polymyositis is an inflammatory myopathy that primarily affects the muscles, leading to weakness and inflammation. While it can cause proximal muscle weakness, it does not typically involve the lower motor neurones or cause fasciculations.  Given the combination of symptoms and signs described, motor neurone disease (ALS) is the most likely diagnosis in this case. Further neurological evaluation and investigations may be needed to confirm the diagnosis and rule out other possible causes. |
|  |  | Attempt 3  Based on the provided information, the most likely diagnosis for the 74-year-old woman with progressive weakness of her legs and hands, falls, wasting of both legs and hands (particularly the thenar eminences), fasciculations in her right quadriceps, increased tone in both legs, and brisk reflexes is B. Motor neurone disease.  Motor neurone disease (MND), also known as amyotrophic lateral sclerosis (ALS), is a progressive neurodegenerative disorder that affects the nerve cells responsible for controlling voluntary muscle movement. It typically presents with weakness and wasting of muscles, initially in the limbs but eventually involving the muscles of the hands, bulbar muscles, and respiratory muscles. Fasciculations (muscle twitches) are commonly observed. Increased tone (spasticity) and brisk reflexes are also characteristic features of upper motor neuron involvement seen in MND.  Option A (Chronic inflammatory demyelinating polyneuropathy) typically presents with symmetric weakness and sensory changes, but it does not typically cause fasciculations or upper motor neuron signs.  Option C (Multiple sclerosis) is characterized by episodes of neurological dysfunction affecting different areas |
| **20** | A 61 year old man has had 2 months of ankle swelling. He has hypertension and a 30 year history of seronegative polyarthritis. His medication includes ramipril, sulfasalzine, hydroxychloroquine sulfate and diclofenac. His BP is 156/90 mmHg. He has pitting oedema to mid thigh and signs of chronic deforming polyarthropathy in his hands, but no joint tenderness. His optic fundi show silver wiring and arteriovenous nipping. Urinalysis: protein 4+, no other abnormalities. Investigations: Sodium 133 mmol/L (135–146) Potassium 5.4 mmol/L (3.5–5.3) Urea 9.0 mmol/L (2.5–7.8) Creatinine 119 μmol/L (60–120) Albumin 21 g/L (35–50) CRP 43 mg/L (<5) Urinary protein:creatinine ratio 1100 mg/mmol (<30)  Which is the most appropriate initial treatment?  A. Candesartan cilexetil  B. Furosemide  C. Indapamide  D. Prednisolone  E. Prednisolone and cyclophosphamide | Attempt 1  Based on the information provided, the most appropriate initial treatment for this patient would be option D. Prednisolone.  The patient presents with several findings that suggest a systemic disease process involving multiple organ systems. The ankle swelling, pitting edema, and proteinuria are indicative of fluid retention and potential renal involvement. The elevated blood pressure, optic fundus changes (silver wiring and arteriovenous nipping), and low serum albumin are consistent with hypertensive retinopathy and hypoalbuminemia, which can be associated with renal disease.  Given the patient's history of seronegative polyarthritis, it is possible that the underlying cause of his symptoms is related to his autoimmune condition. Prednisolone, a corticosteroid, is commonly used in the treatment of inflammatory and autoimmune disorders. It can help reduce inflammation, alleviate symptoms, and potentially slow down the disease progression.  The other options listed are not the most appropriate initial treatment for this patient:  A. Candesartan cilexetil: This is an angiotensin receptor blocker (ARB) commonly used to treat hypertension. While it may help manage the patient's blood pressure, it does not address the underlying autoimmune condition or the fluid retention.  B. Furosemide: This is a loop diuretic that can help with fluid retention. However, using diuretics alone without addressing the underlying cause is unlikely to provide long-term benefit, and it does not treat the autoimmune condition.  C. Indapamide: This is a thiazide-like diuretic used to treat hypertension. Similar to furosemide, it may help with fluid retention, but it does not address the underlying cause.  E. Prednisolone and cyclophosphamide: Cyclophosphamide is an immunosuppressive medication used in certain autoimmune conditions, particularly those involving the kidneys. While it may be considered as a treatment option in some cases, initiating both prednisolone and cyclophosphamide together as the initial treatment is not typically the first-line approach. Prednisolone alone is often used initially to assess the response before considering additional immunosuppressive agents.  It is important for the patient to undergo further evaluation and monitoring to determine the underlying cause of his symptoms and to tailor the treatment accordingly. Consulting with a healthcare professional experienced in rheumatology and/or nephrology would be appropriate for comprehensive management. |
|  |  | Attempt 2  The most appropriate initial treatment for this patient is option D, Prednisolone.  Based on the patient's presentation and history, he has multiple signs and symptoms that suggest a likely diagnosis of Systemic Lupus Erythematosus (SLE) with associated kidney involvement (nephritis) and hypertensive changes in the eyes (silver wiring and arteriovenous nipping). The patient's long history of seronegative polyarthritis may also be a manifestation of the underlying autoimmune disorder.  Treatment with glucocorticoids, such as Prednisolone, is the initial standard of care for managing active SLE with organ involvement, including lupus nephritis. The dose of Prednisolone will depend on the severity of the disease and may be tapered down gradually once the disease is under control. Prednisolone helps suppress the inflammatory response and immune system activity, which can reduce symptoms and improve organ function.  Other treatment options (e.g., Candesartan cilexetil, Furosemide, Indapamide, Prednisolone and cyclophosphamide) may have roles in managing specific symptoms or complications associated with SLE, but Prednisolone is the most appropriate initial treatment for this patient's overall condition. The use of other immunosuppressive agents like cyclophosphamide would require more consideration and may be reserved for more severe cases or if Prednisolone alone is insufficient to control the disease.  As always, the management of a patient's medical condition should be carried out by a qualified healthcare professional who can consider the patient's full medical history and perform a comprehensive evaluation before making treatment decisions. |
|  |  | Attempt 3  Based on the provided information, the most appropriate initial treatment for the 61-year-old man with ankle swelling, hypertension, seronegative polyarthritis, pitting edema, signs of chronic deforming polyarthropathy, silver wiring and arteriovenous nipping on optic fundi, and laboratory findings indicative of nephrotic syndrome (elevated urinary protein:creatinine ratio, low serum albumin) is E. Prednisolone and cyclophosphamide.  The patient's clinical presentation and laboratory findings suggest the presence of systemic lupus erythematosus (SLE) with associated lupus nephritis. SLE is a chronic autoimmune disease that can affect multiple organ systems, including the kidneys. The combination of polyarthritis, seronegativity, optic fundi findings, and nephrotic syndrome in the setting of long-standing polyarthritis is highly suggestive of SLE.  The initial treatment for lupus nephritis involves the use of glucocorticoids (such as prednisolone) to suppress the immune response and reduce inflammation. Cyclophosphamide, a potent immunosuppressant, is often added to the treatment regimen to further control the autoimmune process and prevent further renal damage.  Option A (Candesartan cilexetil) is an angiotensin receptor blocker (ARB) used to manage hypertension, but it does not address the underlying autoimmune process or nephrotic syndrome in this case.  Option B (Furosemide) is a diuretic used to manage fluid overload, but it does not address the underlying autoimmune process or nephrotic syndrome. |
| **21** | A 75 year old woman has had 5 months of a 2 cm red plaque on her leg. Investigation: Skin biopsy: Bowen's disease  Which is the most appropriate topical treatment?  A. 5-fluorouracil (Efudix ® ) cream  B. Betamethasone valerate (Betnovate ® ) cream  C. Diclofenac (Solaraze ® ) gel  D. Isotretinoin gel  E. Salicylic acid gel | Attempt 1  Based on the diagnosis of Bowen's disease, the most appropriate topical treatment option would be option A. 5-fluorouracil (Efudix®) cream.  Bowen's disease, also known as squamous cell carcinoma in situ, is a precancerous skin condition characterized by the presence of abnormal cells in the outermost layer of the skin (epidermis). It typically presents as a well-defined red plaque or patch that may be scaly or crusted.  5-fluorouracil (5-FU) is a chemotherapy medication that can also be used topically for the treatment of precancerous and cancerous skin lesions. It works by inhibiting the growth of abnormal cells and inducing cell death. When applied to the affected area, 5-FU cream selectively targets and destroys the abnormal cells in Bowen's disease.  The other options listed are not the most appropriate topical treatment for Bowen's disease:  B. Betamethasone valerate (Betnovate®) cream: Betamethasone is a potent corticosteroid commonly used to treat various inflammatory skin conditions. However, it is not typically the first-line treatment for Bowen's disease, as it primarily targets inflammation rather than the abnormal cells themselves.  C. Diclofenac (Solaraze®) gel: Diclofenac is a nonsteroidal anti-inflammatory drug (NSAID) that is sometimes used topically for actinic keratoses, another precancerous skin condition. While it may have some utility in certain cases of Bowen's disease, it is generally not considered the first-line treatment.  D. Isotretinoin gel: Isotretinoin is a retinoid medication primarily used in the treatment of severe acne. Its use in Bowen's disease is not well-established, and it is not typically recommended as the first-line treatment.  E. Salicylic acid gel: Salicylic acid is a keratolytic agent commonly used to treat various skin conditions, such as acne and warts. While it may help with the scaling and thickening of the skin in Bowen's disease, it is not the primary treatment for the abnormal cells themselves.  It is important for the patient to consult with a dermatologist or healthcare professional experienced in dermatology for a thorough evaluation and to discuss the best treatment options based on the individual's specific condition and medical history. |
|  |  | Attempt 2  The most appropriate topical treatment for Bowen's disease, a type of intraepidermal squamous cell carcinoma, is A. 5-fluorouracil (Efudix®) cream.  Bowen's disease is a pre-cancerous skin condition characterized by red, scaly, and well-defined plaques. The main goal of treatment is to remove or destroy the abnormal cells on the skin's surface.  5-fluorouracil (5-FU) is a topical chemotherapy agent that interferes with the growth of abnormal cells. It is commonly used to treat Bowen's disease and other precancerous skin conditions. The cream is applied directly to the affected area, and it works by selectively destroying the abnormal cells, allowing healthy skin to regenerate.  Betamethasone valerate (Betnovate®) cream is a topical corticosteroid and is not the most appropriate treatment for Bowen's disease. It is mainly used to reduce inflammation and relieve symptoms associated with certain skin conditions like eczema and psoriasis.  Diclofenac (Solaraze®) gel is a non-steroidal anti-inflammatory drug (NSAID) and is typically used for the treatment of actinic keratoses, another type of precancerous skin lesion, and is not the first-line treatment for Bowen's disease.  Isotretinoin gel is used primarily for the treatment of acne and is not indicated for Bowen's disease.  Salicylic acid gel is commonly used for the treatment of acne, warts, and certain other skin conditions, but it is not the recommended treatment for Bowen's disease.  In summary, the most appropriate topical treatment for Bowen's disease is 5-fluorouracil (Efudix®) cream, as it is specifically designed to target and remove the abnormal cells associated with this condition. However, the final treatment decision should be made in consultation with a dermatologist or healthcare provider, taking into consideration the patient's overall health and medical history. |
|  |  | Attempt 3  The most appropriate topical treatment for Bowen's disease, a form of intraepidermal squamous cell carcinoma, is A. 5-fluorouracil (Efudix®) cream.  Bowen's disease is a precancerous skin condition characterized by the presence of squamous cell carcinoma in situ. Topical 5-fluorouracil cream is a commonly used treatment option for Bowen's disease. It is a chemotherapy medication that inhibits DNA synthesis and leads to the destruction of abnormal cells. It is applied topically to the affected area and works by selectively targeting and eliminating the abnormal cells in the epidermis.  Option B (Betamethasone valerate cream) is a topical corticosteroid that is typically used for inflammatory skin conditions and would not be the most appropriate treatment for Bowen's disease.  Option C (Diclofenac gel) is a nonsteroidal anti-inflammatory drug (NSAID) that is sometimes used topically for actinic keratoses, a precancerous skin condition. While similar in appearance to Bowen's disease, actinic keratoses are different entities, and diclofenac gel is not the most appropriate treatment for Bowen's disease.  Option D (Isotretinoin gel) is primarily used for the treatment of severe acne and is not typically indicated for Bowen's disease.  Option E (Salicylic acid gel) is commonly used for the treatment of various skin conditions, such as acne and warts, but it is not the most appropriate treatment for Bowen's disease.  It's important for the patient to consult with a dermatologist for a comprehensive evaluation and discussion of treatment options for Bowen's disease. |
| **22** | A 32 year old woman has had palpitations and hot flushes for 4 weeks. She has noticed a painless swelling in her neck over the same time and her weight has decreased by 2 kg. She gave birth 4 months ago after a normal pregnancy. She is not breastfeeding. Her pulse rate is 120 bpm and BP 140/90 mmHg. She is tremulous and restless. She has a large smooth non-tender goitre. Investigations: Free T4 35.6 pmol/L (9–25) Free T3 10.8 pmol/L (4.0–7.2) TSH <0.01 mU/L (0.3–4.2) Thyroid peroxidase antibodies >1600 IU/L (<50) Thyroid stimulating antibodies <1.0 IU/L (<1.75)  Which is the most appropriate initial treatment?  A. Carbimazole  B. Propranolol  C. Propylthiouracil  D. Thyroidectomy  E. Thyrotropin alfa | Attempt 1  Based on the clinical presentation and laboratory findings, the most appropriate initial treatment for this patient would be option C. Propylthiouracil.  The patient's symptoms, including palpitations, hot flushes, weight loss, tremors, and restlessness, along with the presence of a painless swelling in the neck (large smooth non-tender goiter), suggest hyperthyroidism. The laboratory results further support this diagnosis, with elevated levels of free T4, free T3, and suppressed TSH. The presence of thyroid peroxidase antibodies indicates an autoimmune cause, most likely Graves' disease.  Propylthiouracil is an antithyroid medication that works by inhibiting the production of thyroid hormones. It can be used as initial treatment to help manage the symptoms and normalize thyroid hormone levels. It is particularly suitable for pregnant or lactating women or those who cannot tolerate carbimazole due to side effects.  The other treatment options listed are as follows:  A. Carbimazole: Carbimazole is another antithyroid medication commonly used to treat hyperthyroidism. However, in this case, propylthiouracil is preferred as the initial treatment option.  B. Propranolol: Propranolol is a beta-blocker that can help alleviate the symptoms of hyperthyroidism, such as palpitations and tremors. While it provides symptomatic relief, it does not address the underlying cause and is not considered a definitive treatment for hyperthyroidism.  D. Thyroidectomy: Surgical removal of the thyroid gland (thyroidectomy) is a treatment option for hyperthyroidism, but it is typically reserved for cases that are unresponsive to or have contraindications to antithyroid medications, or in certain specific circumstances. It is not typically the initial treatment choice.  E. Thyrotropin alfa: Thyrotropin alfa, also known as recombinant human thyroid-stimulating hormone (rhTSH), is not the appropriate initial treatment for hyperthyroidism. It is primarily used in diagnostic testing or preparation for radioactive iodine therapy, but not as the primary treatment for hyperthyroidism itself.  It is important for the patient to be under the care of an endocrinologist or healthcare professional experienced in managing thyroid disorders to monitor her progress, adjust the treatment as needed, and provide appropriate follow-up care. |
|  |  | Attempt 2  The most appropriate initial treatment for this patient with thyrotoxicosis and a large smooth non-tender goitre is C. Propylthiouracil.  The clinical presentation, laboratory results, and the presence of thyroid peroxidase antibodies are consistent with Graves' disease, an autoimmune disorder causing hyperthyroidism. Propylthiouracil is an antithyroid medication that blocks the synthesis of thyroid hormones and reduces their levels in the bloodstream. It is a suitable choice for initial treatment, especially in cases where carbimazole may not be tolerated or is contraindicated.  Propranolol (B) can be used to manage the symptoms of thyrotoxicosis, such as rapid heart rate (tachycardia) and tremor, but it does not address the underlying thyroid hormone excess.  Carbimazole (A) is another antithyroid medication used to treat hyperthyroidism, and it is generally the first-line treatment in most cases. However, propylthiouracil may be preferred in specific situations.  Thyroidectomy (D) is a surgical option for hyperthyroidism, but it is typically reserved for cases where medical treatment has failed, or in certain situations where surgery is deemed more appropriate.  Thyrotropin alfa (E) is not used as a first-line treatment for hyperthyroidism. It is a synthetic form of thyroid-stimulating hormone (TSH) and is primarily used for diagnostic purposes, such as testing the responsiveness of the thyroid gland after radioiodine treatment.  In conclusion, given the patient's clinical presentation, laboratory results, and the diagnosis of Graves' disease, the most appropriate initial treatment is C. Propylthiouracil. However, the final treatment decision should be made in consultation with an endocrinologist, considering the patient's overall health and individual circumstances. |
|  |  | Attempt 3  The most appropriate initial treatment for this patient with hyperthyroidism and a large smooth non-tender goitre is C. Propylthiouracil.  The clinical presentation, including palpitations, hot flushes, weight loss, tremor, and a large smooth non-tender goitre, is suggestive of hyperthyroidism. The laboratory findings of elevated free T4, free T3, and suppressed TSH confirm the diagnosis.  Propylthiouracil (PTU) is an antithyroid medication that inhibits the production of thyroid hormones. It blocks the synthesis of thyroid hormones by interfering with the conversion of T4 to T3 in peripheral tissues and by inhibiting the activity of thyroid peroxidase, an enzyme involved in thyroid hormone synthesis. PTU is effective in controlling the symptoms of hyperthyroidism and can be used as initial treatment.  Carbimazole is another antithyroid medication that is commonly used in the treatment of hyperthyroidism. However, propylthiouracil is preferred in this scenario due to the patient's recent pregnancy. Carbimazole has the potential to cross the placenta and can pose a risk of fetal hypothyroidism. Therefore, propylthiouracil is the preferred choice in this case.  Propranolol, a beta-blocker, can provide symptomatic relief by controlling the heart rate and tremor associated with hyperthyroidism, but it does not address the underlying thyroid hormone excess and is not the most appropriate initial treatment.  Thyroidectomy (surgical removal of the thyroid gland) is an option for patients with hyperthyroidism who do not respond to or have contraindications to antithyroid medications, or in cases of large goitres causing compressive symptoms. However, it is not the most appropriate initial treatment in this patient.  Thyrotropin alfa (recombinant human thyroid-stimulating hormone) is not used as initial treatment for hyperthyroidism. It is typically used in diagnostic procedures, such as radioactive iodine uptake tests or thyroglobulin testing.  It is important for the patient to be managed by an endocrinologist or an appropriate healthcare professional for further evaluation, treatment, and monitoring of her hyperthyroidism. |
| **23** | An 80 year old man has an ulcer over the left heel and reduced mobility. He has a loss of appetite. He has type 2 diabetes mellitus and has previously had a myocardial infarction. The ulcer is 3 cm in diameter and deeply penetrating. Sensory testing shows reduced vibration sense but normal sensation to light touch. His Doppler ratio (ankle brachial pressure index) on the left is 0.68 and on the right is 0.98 (normal value 1.00).  Which is the most likely mechanism of his ulcer?  A. Arterial  B. Neuropathic  C. Nutritional  D. Vasculitic  E. Venous | Attempt 1  Based on the information provided, the most likely mechanism of the ulcer in this case is option A. Arterial.  The patient's clinical history, including reduced mobility, type 2 diabetes mellitus, previous myocardial infarction, and sensory testing showing reduced vibration sense (indicating peripheral neuropathy), suggests that he is at risk for peripheral arterial disease (PAD). The presence of an ulcer that is deeply penetrating and the abnormal Doppler ratio (ankle brachial pressure index) on the left side (0.68) further support an arterial etiology.  Arterial ulcers typically occur due to inadequate blood supply to the affected area, resulting in tissue ischemia and subsequent breakdown. Risk factors for arterial ulcers include atherosclerosis, diabetes mellitus, peripheral neuropathy, and previous vascular events like myocardial infarction.  The other options listed are as follows:  B. Neuropathic: While the patient has peripheral neuropathy as indicated by reduced vibration sense, the characteristics of the ulcer, such as being deeply penetrating and associated with reduced arterial blood supply, are more suggestive of an arterial mechanism rather than a pure neuropathic cause.  C. Nutritional: Nutritional ulcers, such as those caused by deficiencies in vitamins (e.g., vitamin C) or minerals, typically present differently and are not the most likely mechanism in this case.  D. Vasculitic: Vasculitic ulcers occur as a result of inflammation and damage to blood vessels. While they can cause non-healing ulcers, the clinical features and risk factors in this patient make an arterial mechanism more likely.  E. Venous: Venous ulcers typically occur in the lower extremities and are associated with venous insufficiency, which presents with symptoms like leg swelling, varicose veins, and aching pain. The characteristics of the ulcer and the abnormal Doppler ratio in this case are more consistent with an arterial etiology rather than venous insufficiency.  It is important for the patient to be evaluated and managed by a healthcare professional, such as a vascular specialist or wound care specialist, to address the underlying arterial insufficiency and optimize wound healing. |
|  |  | Attempt 2  The most likely mechanism of the ulcer in this 80-year-old man is A. Arterial.  The clinical presentation and findings point towards an arterial ulcer. The risk factors such as reduced mobility, type 2 diabetes mellitus, and a history of myocardial infarction suggest atherosclerosis, which can lead to arterial insufficiency and impaired blood flow to the lower extremities. The reduced ankle brachial pressure index (Doppler ratio) on the left (0.68) indicates decreased blood flow to the affected leg, supporting the arterial involvement.  Arterial ulcers typically occur in areas with reduced blood supply, such as the lower limbs, especially in patients with atherosclerosis, peripheral arterial disease (PAD), or other vascular issues. The ulcer is often deep and painful, with surrounding features such as cool skin, diminished or absent pulses, and delayed wound healing.  The other options are less likely in this case:  B. Neuropathic ulcers are commonly seen in patients with peripheral neuropathy, which often presents with sensory loss to light touch and preserved vibration sense. Neuropathic ulcers are typically painless and occur due to repetitive trauma in areas of sensory loss.  C. Nutritional ulcers are not likely to cause deep penetrating ulcers over the heel, and they are more commonly seen in areas exposed to pressure and friction, such as the sacrum.  D. Vasculitic ulcers result from inflammation of the blood vessels, but they are less common compared to arterial ulcers, and the clinical presentation in this case does not strongly suggest a vasculitic etiology.  E. Venous ulcers are associated with chronic venous insufficiency and are typically found around the lower legs and ankles. They usually have a superficial appearance with exudative features, but the clinical features described in the case point more towards arterial involvement.  Overall, the most likely mechanism of the ulcer in this patient is arterial insufficiency due to atherosclerosis and peripheral arterial disease. |
|  |  | Attempt 3  The most likely mechanism of the ulcer in this case is A. Arterial.  The patient's clinical history, including reduced mobility, type 2 diabetes mellitus, and a previous myocardial infarction, along with the characteristics of the ulcer (deeply penetrating, reduced sensation to vibration, and abnormal ankle brachial pressure index), suggest that the ulcer is likely due to arterial insufficiency.  Arterial insufficiency is commonly seen in individuals with peripheral arterial disease (PAD), which can be associated with atherosclerosis, diabetes mellitus, and other cardiovascular risk factors. Reduced blood flow to the lower extremities leads to tissue ischemia and impaired wound healing, making patients more susceptible to the development of ulcers, particularly over bony prominences or areas of pressure.  Neuropathic ulcers (Option B) are typically seen in individuals with peripheral neuropathy, such as in diabetic neuropathy. They often occur over weight-bearing areas and are associated with loss of protective sensation. However, in this case, the patient's reduced sensation is limited to vibration, and there is no mention of other signs of peripheral neuropathy.  Nutritional ulcers (Option C) are usually seen in individuals with malnutrition or specific vitamin deficiencies. They are typically shallow and present with surrounding skin changes. The clinical presentation in this case is not suggestive of a nutritional etiology.  Vasculitic ulcers (Option D) occur due to inflammation of blood vessels, often as a result of systemic vasculitis. This patient's history and clinical presentation are not consistent with a vasculitic process.  Venous ulcers (Option E) are associated with chronic venous insufficiency and are typically located around the medial or lateral malleoli. The patient's symptoms and characteristics of the ulcer, including reduced sensation to vibration and an abnormal ankle brachial pressure index, are not consistent with venous insufficiency.  It is important for the patient to be evaluated and managed by a healthcare professional experienced in wound |
| **24** | A 73 year old man is in hospital with a chest infection. He has several episodes of confusion, anxiety and aggression, during which he attempts to leave the hospital. He is convinced he is being 'spied on' by the doctors and nurses and insists that 'cameras have been installed in my room'. These episodes alternate with periods of marked lethargy, which become more pronounced towards the evening. His temperature is 37.8°C, pulse rate 100 bpm, BP 110/73 mmHg and respiratory rate 12 breaths per minute.  Which is the most likely diagnosis?  A. Alzheimer's dementia  B. Bipolar disorder  C. Delirium  D. Lewy body dementia  E. Schizophrenia | Attempt 1  Based on the given information, the most likely diagnosis for this patient is option C. Delirium.  Delirium is an acute and fluctuating change in mental status characterized by disturbances in attention, awareness, and cognition. It often occurs in the setting of an underlying medical condition, such as infection or medication side effects. The patient in this case has a chest infection, which could contribute to the development of delirium.  The key features that support the diagnosis of delirium in this patient are the acute onset of confusion, anxiety, aggression, and delusions (believing he is being spied on), as well as attempts to leave the hospital. The fluctuation between periods of confusion and lethargy, particularly worsening in the evening (sundowning), is also characteristic of delirium.  The other options listed are as follows:  A. Alzheimer's dementia: Alzheimer's dementia is a chronic and progressive neurodegenerative disorder characterized by gradual cognitive decline. While some behavioral disturbances can occur in Alzheimer's dementia, the acute and fluctuating nature of symptoms described in this case is not consistent with this diagnosis.  B. Bipolar disorder: Bipolar disorder is a mood disorder characterized by episodes of depression and mania. While bipolar disorder can present with mood swings, the presence of confusion, delusions, and fluctuations in mental status as described in this case are more indicative of delirium rather than bipolar disorder.  D. Lewy body dementia: Lewy body dementia is a neurodegenerative disorder characterized by cognitive decline, visual hallucinations, parkinsonism, and fluctuations in cognition. While some features overlap with the symptoms described in this case, such as fluctuations in mental status and visual hallucinations, the acute onset and presence of a chest infection are more suggestive of delirium.  E. Schizophrenia: Schizophrenia is a chronic psychiatric disorder characterized by the presence of positive symptoms (e.g., hallucinations, delusions) and negative symptoms (e.g., flattened affect, social withdrawal). The acute onset, fluctuations, and association with a chest infection are not typical of schizophrenia.  It is crucial to promptly identify and address the underlying cause of delirium, such as the chest infection in this patient. Managing the infection and providing supportive care, including maintaining a calm and safe environment, can help improve the symptoms of delirium. Additionally, the patient should be evaluated by a healthcare professional experienced in managing delirium to ensure appropriate management and monitoring. |
|  |  | Attempt 2  The most likely diagnosis for the 73-year-old man with a chest infection and alternating episodes of confusion, anxiety, aggression, and marked lethargy is C. Delirium.  Delirium is a common and acute neuropsychiatric syndrome characterized by disturbances in attention, awareness, and cognition. It often occurs in the setting of an underlying medical condition, such as an infection, and can be precipitated or worsened by hospitalization and medications.  The key features in this case that suggest delirium are:  1. Presence of an acute medical condition (chest infection).  2. Fluctuating course of symptoms, with periods of confusion, anxiety, and aggression alternating with marked lethargy.  3. Delusions and paranoia (e.g., believing he is being spied on and cameras are installed in his room).  4. Elevated temperature, which can be a sign of infection.  5. Age of the patient (73 years old), as delirium is more common in older individuals.  Delirium is often reversible once the underlying medical condition is addressed and appropriate treatment is provided. It requires urgent evaluation and management to prevent complications and ensure patient safety.  The other options are less likely in this case:  A. Alzheimer's dementia: Alzheimer's dementia is a chronic and progressive neurodegenerative disorder. While it can cause cognitive and behavioral changes, it typically has a more insidious onset and a gradual decline in cognitive function, which is different from the acute and fluctuating symptoms seen in delirium.  B. Bipolar disorder: Bipolar disorder is a mood disorder characterized by episodes of mania and depression. It does not typically present with acute confusion and delusional thinking like what is seen in this case.  D. Lewy body dementia: Lewy body dementia is characterized by cognitive decline, visual hallucinations, and parkinsonism. While delirium can occur in patients with Lewy body dementia, the presence of an acute medical condition (chest infection) makes delirium more likely.  E. Schizophrenia: Schizophrenia is a chronic psychiatric disorder characterized by positive symptoms (hallucinations, delusions) and negative symptoms (flat affect, social withdrawal). Delirium is an acute and reversible condition, whereas schizophrenia has a more chronic course.  Overall, given the clinical presentation and the patient's medical condition, delirium is the most likely diagnosis. |
|  |  | Attempt 3  The most likely diagnosis in this case is C. Delirium.  Delirium is an acute confusional state characterized by a disturbance in attention and awareness. It is often caused by an underlying medical condition, such as an infection or medication side effects, and is commonly seen in older adults, particularly those in a hospital setting.  The patient's symptoms of confusion, anxiety, aggression, delusional beliefs (thinking he is being spied on), and fluctuations in level of consciousness are consistent with delirium. The presence of a chest infection suggests that the delirium may be due to an underlying medical condition.  Alzheimer's dementia (Option A) is a chronic, progressive neurodegenerative disorder characterized by memory loss and cognitive decline. It does not typically present with acute episodes of confusion and delusions.  B |
| **25** | A 78 year old woman is admitted to the surgical unit with a suspected vesicocolic fistula. She has a history of hypertension, type 2 diabetes mellitus and angina. She is taking amlodipine, metformin, gliclazide, simvastatin and bisoprolol. Her serum creatinine is 120 μmol/L (60–120). The consultant surgeon requests a CT scan of abdomen with contrast.  Which medication should be stopped before her CT scan?  A. Amlodipine  B. Bisoprolol  C. Gliclazide  D. Metformin  E. Simvastatin | Attempt 1  The medication that should be stopped before the CT scan with contrast in this patient is option D. Metformin.  Metformin is an oral antidiabetic medication commonly used to treat type 2 diabetes. However, it can pose a risk of a rare but potentially serious side effect called lactic acidosis when administered in the setting of impaired kidney function or contrast administration. Since the patient's serum creatinine is at the upper limit of the normal range (120 μmol/L), it indicates mild renal impairment, and there is a concern for contrast-induced nephropathy.  Contrast-induced nephropathy is a form of acute kidney injury that can occur after the administration of contrast agents used in imaging procedures such as CT scans. The risk of developing this complication is higher in patients with pre-existing renal impairment. Since metformin is primarily excreted through the kidneys, it is necessary to temporarily discontinue it before the CT scan to minimize the risk of lactic acidosis.  The other options listed are as follows:  A. Amlodipine: Amlodipine is a calcium channel blocker used to treat hypertension and angina. It does not need to be stopped before the CT scan.  B. Bisoprolol: Bisoprolol is a beta-blocker commonly used in the management of hypertension and angina. It does not need to be stopped before the CT scan.  C. Gliclazide: Gliclazide is an oral antidiabetic medication. While it is important to monitor blood glucose levels during the fasting period before the CT scan, it does not need to be stopped.  E. Simvastatin: Simvastatin is a statin medication used to manage high cholesterol levels. It does not need to be stopped before the CT scan.  It is essential for the patient to follow the instructions provided by the healthcare team regarding the temporary discontinuation of metformin, as well as any other pre-scan preparations. The decision to stop and restart metformin should be made in consultation with the healthcare provider managing the patient's diabetes. |
|  |  | Attempt 2  Before a CT scan with contrast, it is important to consider the risk of contrast-induced nephropathy, especially in patients with pre-existing renal impairment. In this case, the patient's serum creatinine level is at the upper limit of normal (120 μmol/L), which suggests some degree of renal impairment. Among the medications listed, the one that poses the greatest risk for contrast-induced nephropathy is:  D. Metformin  Metformin is an oral antidiabetic medication commonly used in the treatment of type 2 diabetes. It is excreted primarily by the kidneys, and its use in patients with renal impairment can increase the risk of lactic acidosis, particularly in the setting of contrast administration. Contrast-induced nephropathy can further worsen renal function and increase the risk of lactic acidosis with metformin use.  Therefore, metformin should be stopped before the CT scan and withheld for at least 48 hours after the procedure, as long as the patient's renal function remains stable and there are no signs of contrast-induced nephropathy. Alternative measures for glycemic control should be considered during this period to manage the patient's diabetes.  The other medications listed (A. Amlodipine, B. Bisoprolol, C. Gliclazide, and E. Simvastatin) do not pose the same risk of contrast-induced nephropathy and do not need to be stopped before the CT scan. However, it is always important to review the patient's complete medication list and consider any other factors that may influence the use of contrast agents or the safety of other medications during the procedure. |
|  |  | Attempt 3  The medication that should be stopped before the CT scan with contrast is D. Metformin.  Metformin is an oral antidiabetic medication commonly used in the management of type 2 diabetes. However, it can increase the risk of a rare but serious side effect called lactic acidosis, particularly in patients with impaired renal function. The presence of a serum creatinine level at the upper limit of the normal range suggests some degree of renal impairment in this patient.  Contrast agents used in imaging studies, such as CT scans, can further stress the kidneys and potentially increase the risk of contrast-induced nephropathy. When metformin is combined with contrast agents, there is a potential for additive renal toxicity, which can increase the risk of lactic acidosis.  Therefore, it is generally recommended to temporarily stop metformin before a CT scan with contrast in patients with renal impairment to minimize the risk. The decision to discontinue metformin should be made in consultation with the patient's healthcare provider, who can provide specific guidance based on the patient's individual circumstances. In this case, the patient's serum creatinine level of 120 µmol/L suggests some degree of renal impairment, making it appropriate to stop metformin before the CT scan. |
| **26** | A 50 year old man has a 3 month history of right loin pain and weight loss. For the past 20 years, he has smoked ten cigarettes per day. His temperature is 37.4°C, pulse rate is 72 bpm and BP is 142/74 mmHg. Investigations: Haemoglobin 11.2 g/L (130–175) Platelets 340 × 109/L (150–400) White cell count 10.1 × 109/L (4.0–11.0) Urinalysis blood 3+  Which is the most likely diagnosis?  A. Benign prostatic hypertrophy  B. Pyelonephritis  C. Renal calculus  D. Renal cancer  E. Urinary tract infection | Attempt 1  Based on the information provided, the most likely diagnosis for this patient is option D. Renal cancer.  The patient's symptoms of right loin pain and weight loss, along with the presence of hematuria (blood in the urine), raise suspicion for renal cancer. Risk factors such as a history of smoking and the duration of symptoms further support this possibility.  The laboratory findings of a decreased hemoglobin level (11.2 g/L) may suggest anemia, which can be associated with renal cancer due to chronic blood loss in the urine. The platelet and white cell counts are within the normal range and do not provide specific clues for a particular diagnosis.  The other options listed are as follows:  A. Benign prostatic hypertrophy (BPH): BPH typically presents with lower urinary tract symptoms such as urinary frequency, urgency, hesitancy, and weak urine flow. It is less likely to cause loin pain and weight loss as described in this case.  B. Pyelonephritis: Pyelonephritis is a kidney infection that can cause fever, flank pain, and urinary symptoms. However, weight loss is less commonly associated with pyelonephritis, and the presence of hematuria raises concern for a more serious underlying condition such as renal cancer.  C. Renal calculus: Renal calculi, or kidney stones, can cause severe loin pain. However, weight loss is not typically associated with renal calculi, and the presence of persistent hematuria should prompt consideration of other causes.  E. Urinary tract infection (UTI): While UTIs can cause symptoms such as urinary frequency, urgency, and dysuria, the presence of loin pain, weight loss, and persistent hematuria make a UTI less likely as the sole explanation for the patient's presentation.  Given the concerning symptoms, it is important for the patient to undergo further evaluation, such as imaging studies (e.g., CT scan) and urological referral, to confirm the diagnosis of renal cancer and initiate appropriate management. |
|  |  | Attempt 2  Given the patient's age, history of smoking, and the presence of right loin pain and weight loss, the most likely diagnosis among the options provided is:  D. Renal cancer  Renal cancer, also known as renal cell carcinoma, is a malignancy that originates from the cells of the renal tubules in the kidneys. It commonly presents with symptoms like flank pain, weight loss, and sometimes hematuria (blood in the urine). The presence of blood (3+) in the urinalysis raises concern for a possible renal tumor.  While the other options (A. Benign prostatic hypertrophy, B. Pyelonephritis, C. Renal calculus, and E. Urinary tract infection) may cause similar symptoms, the combination of age, smoking history, and the presence of blood in the urine is most suggestive of renal cancer in this case.  It is important for the patient to undergo further evaluation, including imaging studies (such as CT scan or ultrasound) and possibly a biopsy to confirm the diagnosis of renal cancer and determine the extent of the disease. Early detection and appropriate management are crucial for better outcomes in cases of renal cancer. |
|  |  | Attempt 3  Based on the provided information, the most likely diagnosis in this case is D. Renal cancer.  Several factors point towards renal cancer as the likely diagnosis:  1. The presence of right loin pain: Loin pain can be a symptom of renal cancer, especially if it is persistent and associated with other concerning factors.  2. Weight loss: Unintentional weight loss is often associated with malignancies, including renal cancer.  3. Smoking history: Smoking is a well-established risk factor for the development of renal cancer.  4. Hematuria: The presence of blood (3+) in the urine can be a sign of renal cancer. Hematuria may be visible (macroscopic) or only detected on urine analysis (microscopic).  Although other conditions such as pyelonephritis, renal calculus, and urinary tract infection can also present with similar symptoms, the combination of right loin pain, weight loss, and hematuria raises a higher suspicion of renal cancer, especially given the patient's smoking history.  It's important to note that a definitive diagnosis can only be made through further investigations, such as imaging studies (e.g., CT scan, ultrasound) and possibly a biopsy. Therefore, prompt medical evaluation and follow-up with appropriate specialists are essential to confirm the diagnosis and initiate appropriate management. |
| **27** | A 74 year old man with type 2 diabetes has been increasingly unwell and experiencing progressive thirst and nausea for 2 weeks. Initially he described needing to pass urine more frequently than usual, but now he has not passed urine for 24 hours. He is dehydrated. Investigations: Sodium 149 mmol/L (135–146) Potassium 5.2 mmol/L (3.5–5.3) Chloride 101 mmol/L (95–106) Urea 15.4 mmol/L (2.5–7.8) Creatinine 208 μmol/L (60–120) Fasting glucose 41.7 mmol/L (3.0–6.0)  Which is the calculated serum osmolality?  A. 206.1 mmol/L  B. 255.2 mmol/L  C. 312.3 mmol/L  D. 355.1 mmol/L  E. Impossible to calculate, more information needed | Attempt 1  To calculate the serum osmolality, we can use the formula:  Serum osmolality (mOsm/kg) = 2 × ([Na] + [K]) + glucose + urea  In this case, the values provided are:  Sodium ([Na]) = 149 mmol/L  Potassium ([K]) = 5.2 mmol/L  Glucose = 41.7 mmol/L  Urea = 15.4 mmol/L  Plugging in these values into the formula, we get:  Serum osmolality = 2 × (149 + 5.2) + 41.7 + 15.4  Serum osmolality = 2 × 154.2 + 41.7 + 15.4  Serum osmolality = 308.4 + 41.7 + 15.4  Serum osmolality = 365.5 mmol/L  Therefore, the calculated serum osmolality is approximately 365.5 mmol/L.  The closest option to this value is option D. 355.1 mmol/L. |
|  |  | Attempt 2  Let's calculate the serum osmolality again using the correct formula:  Serum Osmolality (mOsm/kg) = 2(Na) + glucose/18 + BUN/2.8  Given values:  Sodium (Na) = 149 mmol/L  Glucose = 41.7 mmol/L  BUN = 15.4 mmol/L  Serum Osmolality = 2(149) + 41.7/18 + 15.4/2.8  Now, calculate:  Serum Osmolality ≈ 298 + 2.316 + 5.5 ≈ 305.816 mOsm/kg  The calculated serum osmolality is approximately 305.816 mOsm/kg.  Now, let's check which of the provided options is closest to the calculated value:  A. 206.1 mmol/L (This is too low and does not match the calculated value)  B. 255.2 mmol/L (This is too low and does not match the calculated value)  C. 312.3 mmol/L (This is too high and does not match the calculated value)  D. 355.1 mmol/L (This is too high and does not match the calculated value)  E. Impossible to calculate, more information needed (This is not correct; we were able to calculate the serum osmolality)  None of the provided options exactly match the calculated value, but the closest one is:  B. 255.2 mmol/L  However, the correct answer should be closer to 305.8 mOsm/kg, so none of the given options are entirely accurate. |
|  |  | Attempt 3  To calculate the serum osmolality, we can use the following formula:  Serum Osmolality (mOsm/kg) = 2(Na) + glucose/18 + urea/2.8  Plugging in the values provided:  Na = 149 mmol/L  Glucose = 41.7 mmol/L  Urea = 15.4 mmol/L  Serum Osmolality = 2(149) + 41.7/18 + 15.4/2.8  Calculating this equation:  Serum Osmolality = 298 + 2.317 + 5.5  Serum Osmolality = 305.817 mOsm/kg  Therefore, the calculated serum osmolality is approximately 305.8 mmol/L.  The correct answer is not among the options given, and it seems that the calculated osmolality was rounded incorrectly. |
| **28** | A 65 year old man receives a renal transplant. He is transferred back to the ward after four hours in recovery. His pulse is 106 bpm regular, BP 110/70 mmHg and respiratory rate 18 breaths per minute. His chest is clear on auscultation. His urine output has been 15–20 mL per hour while in recovery. Drain output has been 120 mL since surgery. Investigations: Haemoglobin 90 g/L (130–175) (preoperative level 103 g/L) Sodium 142 mmol/L (135–146) Potassium 5.8 mmol/L (3.5–5.3) Urea 31.9 mmol/L (2.5–7.8) Creatinine 590 μmol/L (60–120)  Which is the next most appropriate management step?  A. Blood transfusion  B. Fluid challenge  C. Furosemide  D. Haemofiltration  E. Insulin and dextrose infusion | Attempt 1  The next most appropriate management step for this patient is option D. Haemofiltration.  The patient's clinical presentation, including decreased urine output (<0.5 mL/kg/h), elevated creatinine (590 μmol/L), and deranged electrolyte levels (elevated potassium and urea), suggests acute kidney injury (AKI) following renal transplant. These findings indicate that the transplanted kidney is not functioning adequately to maintain normal kidney function.  Given the severity of the AKI and the associated electrolyte abnormalities, the most appropriate management step is to initiate renal replacement therapy (RRT) such as haemofiltration. Haemofiltration is a form of continuous renal replacement therapy (CRRT) that helps remove excess fluids, urea, and other waste products from the blood in patients with acute kidney injury.  The other options listed are as follows:  A. Blood transfusion: The patient's low hemoglobin level (90 g/L) may indicate anemia, but transfusion alone would not address the underlying cause of the acute kidney injury. The primary focus should be on managing the renal dysfunction.  B. Fluid challenge: The patient is already receiving adequate fluids and has a normal blood pressure. A fluid challenge may further worsen fluid overload and does not address the underlying cause of the acute kidney injury.  C. Furosemide: Furosemide is a diuretic that promotes urine output. However, in the context of acute kidney injury, the effectiveness of diuretics is often limited. Furthermore, the patient is already experiencing fluid and electrolyte imbalances, and furosemide may exacerbate these abnormalities.  E. Insulin and dextrose infusion: Insulin and dextrose infusion is typically used in the management of hyperkalemia, but it does not address the underlying cause of the acute kidney injury. In this case, the primary concern is the initiation of renal replacement therapy to provide adequate renal support.  It is important to involve a nephrologist or a specialist in the management of renal transplant patients for further evaluation and guidance in managing the patient's acute kidney injury. |
|  |  | Attempt 2  The patient has a low hemoglobin level (90 g/L) and elevated serum urea (31.9 mmol/L) and creatinine (590 μmol/L), which indicates acute kidney injury (AKI) following the renal transplant.  Given the low urine output (15-20 mL per hour) and high creatinine level, the most appropriate management step would be to initiate fluid challenge (Option B). A fluid challenge involves administering a bolus of intravenous fluids to improve kidney perfusion and urine output. This can help correct the intravascular volume depletion and improve kidney function in the immediate postoperative period.  Options A, D, and E are not the immediate management steps for improving kidney perfusion. Blood transfusion (Option A) may be considered later if the patient remains anemic. Haemofiltration (Option D) is a more advanced form of renal replacement therapy and is not the first-line treatment in this scenario. Insulin and dextrose infusion (Option E) may be used to treat hyperkalemia if present, but it does not address the underlying issue of kidney perfusion.  Option C (Furosemide) would be contraindicated as it may worsen the patient's kidney function in the setting of hypovolemia and decreased urine output.  Therefore, the next most appropriate management step is to initiate a fluid challenge (Option B) to improve kidney perfusion and urine output. |
|  |  | Attempt 3  The given scenario describes a patient who has undergone a renal transplant and is now experiencing decreased urine output (oliguria) and elevated creatinine levels, indicating impaired kidney function. This suggests the possibility of acute kidney injury (AKI) following the transplant.  In this situation, the next most appropriate management step would be a fluid challenge (Option B). A fluid challenge involves administering a bolus of intravenous fluids to assess the patient's response and improve renal perfusion. It aims to increase urine output and improve kidney function.  Administering a blood transfusion (Option A) may be considered if the patient's hemoglobin level drops significantly and they become symptomatic, but it is not the immediate management priority in this case.  Furosemide (Option C) is a diuretic that could be used to promote diuresis in certain situations, but it may not be effective in cases of AKI. It is not the primary management step in this scenario.  Haemofiltration (Option D) is a renal replacement therapy that may be considered if the patient's renal function does not improve with other interventions. However, it is not the initial step and is typically reserved for more severe cases of AKI.  Insulin and dextrose infusion (Option E) is not indicated in this scenario unless there is evidence of hyperkalemia requiring urgent management, which is not mentioned in the given information.  Therefore, the most appropriate next management step in this case is a fluid challenge (Option B). |
| **29** | A 70 year old man has a sharp stabbing pain in his jaw and cheek that lasts for seconds. He reports that the pain is triggered when brushing his teeth, cold wind and touching his face.  Which is the most appropriate treatment?  A. Carbamazepine  B. Indometacin  C. Morphine  D. Prednisolone  E. Pregabalin | Attempt 1  The most appropriate treatment for the described symptoms is option A. Carbamazepine.  The patient's symptoms of sharp stabbing pain in the jaw and cheek that is triggered by specific stimuli (such as brushing teeth, cold wind, and touching the face) are consistent with trigeminal neuralgia. Trigeminal neuralgia is characterized by sudden, severe facial pain that is often described as electric shock-like or stabbing in nature.  Carbamazepine is the first-line treatment for trigeminal neuralgia. It is an anticonvulsant medication that helps to relieve the neuropathic pain associated with this condition. Carbamazepine works by reducing the abnormal firing of nerve signals that contribute to the pain.  The other options listed are as follows:  B. Indometacin: Indometacin is a nonsteroidal anti-inflammatory drug (NSAID) that is primarily used for its anti-inflammatory properties. However, it is not typically effective in managing the neuropathic pain associated with trigeminal neuralgia.  C. Morphine: Morphine is a potent opioid analgesic that is commonly used for moderate to severe pain. However, it is not considered the first-line treatment for trigeminal neuralgia, and other medications, such as carbamazepine, are more effective for this specific condition.  D. Prednisolone: Prednisolone is a corticosteroid that is often used for its anti-inflammatory and immunosuppressive effects. However, in the case of trigeminal neuralgia, there is no clear evidence supporting the use of corticosteroids as the primary treatment.  E. Pregabalin: Pregabalin is an anticonvulsant medication that is commonly used for the management of neuropathic pain. While it may be effective for some types of neuropathic pain, carbamazepine is the preferred first-line treatment for trigeminal neuralgia.  It is important for the patient to be evaluated and prescribed the appropriate dosage of carbamazepine by a healthcare professional experienced in managing trigeminal neuralgia. Regular follow-up is also necessary to monitor the response to treatment and make any necessary adjustments. |
|  |  | Attempt 2  The symptoms described by the patient are characteristic of trigeminal neuralgia, which is a neuropathic pain condition involving the trigeminal nerve. The pain is sharp, stabbing, and triggered by certain actions like brushing teeth, cold wind, and touching the face.  The most appropriate treatment for trigeminal neuralgia is carbamazepine (Option A). Carbamazepine is an anticonvulsant drug that is effective in relieving the pain associated with trigeminal neuralgia. It helps in reducing the hyperactivity of the trigeminal nerve, which is responsible for the episodes of intense pain.  Options B (Indometacin) and C (Morphine) are not considered first-line treatments for trigeminal neuralgia. Indometacin is a nonsteroidal anti-inflammatory drug (NSAID) that is not typically used for this condition, and morphine is an opioid that is not effective in treating neuropathic pain like trigeminal neuralgia.  Option D (Prednisolone) is a corticosteroid, and while it may have some anti-inflammatory effects, it is not considered a first-line treatment for trigeminal neuralgia.  Option E (Pregabalin) is another anticonvulsant medication that is sometimes used as an alternative to carbamazepine for treating trigeminal neuralgia. However, carbamazepine is generally considered the first-line treatment due to its proven efficacy.  Therefore, the most appropriate treatment for the patient's trigeminal neuralgia is carbamazepine (Option A). |
|  |  | Attempt 3  The described symptoms are suggestive of trigeminal neuralgia, a condition characterized by sudden, severe facial pain that is typically triggered by specific activities or stimuli. The most appropriate treatment for trigeminal neuralgia is carbamazepine (Option A). Carbamazepine is an anticonvulsant medication that is effective in reducing the frequency and intensity of the pain episodes in trigeminal neuralgia. It is considered the first-line treatment for this condition.  Indometacin (Option B) is a nonsteroidal anti-inflammatory drug (NSAID) that may be used in some cases of facial pain, but it is not typically the treatment of choice for trigeminal neuralgia.  Morphine (Option C) is a potent opioid analgesic and is not typically used as a first-line treatment for trigeminal neuralgia. It may be considered in certain cases when other treatments have failed to provide relief.  Prednisolone (Option D) is a corticosteroid and may be used in certain types of facial pain conditions, but it is not the primary treatment for trigeminal neuralgia.  Pregabalin (Option E) is an anticonvulsant medication that has been used in the management of neuropathic pain, including trigeminal neuralgia. However, it is not considered the first-line treatment and is often reserved for cases that do not respond adequately to carbamazepine or have contr |
| **30** | A 64 year old man has developed a tremor in both arms over the last 6 months. It is worse on the right. He also reports difficulty sleeping due to restlessness. He appears emotionally flat and has a tremor at rest that is alleviated on movement.  Which neurotransmitter is most likely to be deficient?  A. Acetylcholine  B. Dopamine  C. Glycine  D. Norepinephrine (noradrenaline)  E. Serotonin | Attempt 1  The neurotransmitter most likely to be deficient in this case is option B. Dopamine.  The patient's symptoms of tremor, particularly at rest and worsened on the right side, along with difficulty sleeping due to restlessness, suggest a possible diagnosis of Parkinson's disease. Parkinson's disease is a neurodegenerative disorder characterized by the progressive loss of dopaminergic neurons in the substantia nigra of the brain.  Dopamine is an important neurotransmitter involved in the regulation of movement, mood, sleep, and motivation. The loss of dopamine-producing neurons in Parkinson's disease leads to a deficiency of dopamine in the brain, resulting in the characteristic motor symptoms such as tremors, rigidity, and bradykinesia (slowness of movement). The emotional flatness or decreased facial expression, known as hypomimia, is also associated with dopamine deficiency in Parkinson's disease.  The other options listed are as follows:  A. Acetylcholine: Acetylcholine is not typically deficient in Parkinson's disease. In fact, the imbalance between dopamine and acetylcholine, with relatively increased acetylcholine activity, is believed to contribute to the motor symptoms of Parkinson's disease.  C. Glycine: Glycine is an inhibitory neurotransmitter primarily involved in the spinal cord and brainstem. It is not directly related to the symptoms described in this case.  D. Norepinephrine (noradrenaline): Norepinephrine is a neurotransmitter involved in the regulation of arousal, attention, and mood. While norepinephrine dysfunction can occur in certain neurological and psychiatric conditions, it is not primarily associated with the motor symptoms seen in Parkinson's disease.  E. Serotonin: Serotonin is a neurotransmitter involved in the regulation of mood, appetite, and sleep, among other functions. While serotonin dysfunction can contribute to various mood and sleep disorders, it is not the primary neurotransmitter implicated in the motor symptoms of Parkinson's disease.  It is important for the patient to be evaluated by a healthcare professional, such as a neurologist, for a comprehensive assessment and proper diagnosis. Treatment options, including dopaminergic medications, can be considered to manage the symptoms and improve the patient's quality of life. |
|  |  | Attempt 2  Based on the symptoms described, the most likely neurotransmitter to be deficient in this case is B. Dopamine.  The combination of resting tremor, difficulty sleeping due to restlessness, and emotional flatness are classic symptoms of Parkinson's disease, a neurodegenerative disorder that primarily involves the loss of dopamine-producing neurons in the substantia nigra region of the brain. Dopamine is a neurotransmitter that plays a crucial role in regulating movement, mood, and sleep.  The tremor at rest that is alleviated on movement is a hallmark sign of Parkinson's disease and is caused by the degeneration of dopaminergic neurons, leading to an imbalance between dopamine and other neurotransmitters in the brain.  The other neurotransmitters listed (Acetylcholine, Glycine, Norepinephrine, and Serotonin) are not primarily associated with the symptoms and characteristics described in this case. |
|  |  | Attempt 3  The clinical presentation described suggests a parkinsonian syndrome, which is commonly associated with a deficiency of dopamine (Option B) in the brain. Parkinson's disease is the most common cause of parkinsonian syndrome, characterized by resting tremor, bradykinesia (slowness of movement), rigidity, and postural instability.  Dopamine is a neurotransmitter involved in various functions, including movement control. In Parkinson's disease, there is a degeneration of dopamine-producing cells in the substantia nigra, leading to a deficiency of dopamine in the basal ganglia, a region of the brain involved in motor control.  While other neurotransmitters, such as acetylcholine, norepinephrine, and serotonin, also play important roles in the central nervous system, dopamine deficiency is specifically associated with the motor symptoms seen in parkinsonian syndromes. |
| **31** | An 85 year old man is admitted from a nursing home with a spreading cellulitis originating from an ulcer over his right ankle. Cultures taken from the ulcer and blood have grown MRSA. He is mildly confused. His temperature is 39.5°C, pulse rate 96 bpm and BP 114/60 mmHg.  Which is the most appropriate initial antibiotic treatment?  A. Co-amoxiclav  B. Flucloxacillin  C. Meropenem  D. Piperacillin with tazobactam  E. Vancomycin | Attempt 1  Given the patient's history of cellulitis with confirmed MRSA infection, the most appropriate initial antibiotic treatment option would be option E. Vancomycin.  MRSA (Methicillin-Resistant Staphylococcus aureus) is a type of bacteria that is resistant to many commonly used antibiotics, including flucloxacillin and co-amoxiclav. Therefore, these options would not be effective against MRSA.  Vancomycin is an antibiotic that is effective against MRSA and is commonly used as the initial treatment for severe MRSA infections, such as cellulitis associated with systemic symptoms like fever and spreading infection. It is administered intravenously and has good activity against MRSA strains.  Options C and D (meropenem and piperacillin with tazobactam) are broad-spectrum antibiotics that may be effective against MRSA; however, they are not typically used as first-line treatment for MRSA infections like cellulitis. These antibiotics are usually reserved for more severe infections or when other treatment options are not available.  It is important to note that the choice of antibiotic may depend on local antibiotic resistance patterns and individual patient factors. Therefore, it is recommended to consult with an infectious disease specialist or follow local guidelines for the treatment of MRSA infections. |
|  |  | Attempt 2  Given the patient's clinical presentation of spreading cellulitis, fever, and positive MRSA cultures, the most appropriate initial antibiotic treatment would be E. Vancomycin.  Vancomycin is a potent antibiotic commonly used to treat infections caused by methicillin-resistant Staphylococcus aureus (MRSA), which is a type of bacteria resistant to many common antibiotics like penicillins and cephalosporins. The patient's condition, with spreading cellulitis and positive MRSA cultures, warrants the use of an antibiotic that is effective against MRSA.  Options A (Co-amoxiclav), B (Flucloxacillin), C (Meropenem), and D (Piperacillin with tazobactam) are not as appropriate in this case. Co-amoxiclav and Flucloxacillin do not cover MRSA, and Meropenem and Piperacillin with tazobactam are broad-spectrum antibiotics that are not typically the first-line choice for treating MRSA infections. Vancomycin is the more suitable choice based on the information provided. |
|  |  | Attempt 3  Given the presence of MRSA (methicillin-resistant Staphylococcus aureus) in the cultures and the severity of the cellulitis, the most appropriate initial antibiotic treatment would be Vancomycin (Option E). MRSA is resistant to many commonly used antibiotics, including flucloxacillin (Option B) and co-amoxiclav (Option A), which target methicillin-susceptible Staphylococcus aureus (MSSA).  Vancomycin is an effective antibiotic against MRSA and is commonly used for the treatment of severe infections caused by MRSA. It is usually administered intravenously. In this case, given the spreading cellulitis, high temperature, and the patient's mild confusion, intravenous therapy with Vancomycin would be the most appropriate choice.  Options C (Meropenem) and D (Piperacillin with tazobactam) are broad-spectrum antibiotics that are not specifically targeted against MRSA. They are more commonly used for treating infections caused by gram-negative bacteria. |
| **32** | A 75 year old man is found collapsed at home and is brought to the Emergency Department. He has right sided weakness and reduced consciousness. He has type 2 diabetes mellitus, atrial fibrillation and hypertension. He is taking warfarin. Investigations: INR 4.6 (<1.4) CT scan of the head shows a large intracranial haemorrhage. He is given intravenous vitamin K.  Which is the most appropriate next additional treatment?  A. Cryoprecipitate  B. Fresh frozen plasma  C. Fibrinogen concentrate  D. No additional treatment needed  E. Prothrombin complex concentrate | Attempt 1  The most appropriate next additional treatment in this scenario would be option E. Prothrombin complex concentrate.  The patient's presentation with a large intracranial hemorrhage and an elevated INR of 4.6 suggests over-anticoagulation with warfarin. Vitamin K is given to reverse the anticoagulant effects of warfarin by promoting the synthesis of clotting factors.  However, in the setting of an acute intracranial hemorrhage and high INR, there is a need for urgent and more rapid reversal of anticoagulation. Fresh frozen plasma (option B) and cryoprecipitate (option A) contain various clotting factors, but they have limitations in terms of volume and time needed for preparation, as well as the risk of volume overload and potential transmission of blood-borne infections.  Fibrinogen concentrate (option C) is used to address low fibrinogen levels, but it may not be the optimal choice as the primary treatment in this scenario.  Prothrombin complex concentrate (option E) is a concentrated form of clotting factors, including factors II, VII, IX, and X, which are deficient in warfarin-induced coagulopathy. It provides a more rapid and targeted reversal of anticoagulation. Therefore, in the case of an acute intracranial hemorrhage with over-anticoagulation, prothrombin complex concentrate is the preferred treatment option.  It is important to note that the decision on the choice and dose of reversal agents should be made in consultation with a hematologist or a specialist with expertise in anticoagulation management, taking into account individual patient factors and local guidelines. |
|  |  | Attempt 2 |
|  |  | Attempt 3  In this scenario, the patient has an intracranial hemorrhage and an elevated INR, indicating an over-anticoagulated state due to warfarin use. The most appropriate next additional treatment would be Prothrombin complex concentrate (Option E).  Prothrombin complex concentrate (PCC) contains factors II, VII, IX, and X, which are the clotting factors that are affected by warfarin. By administering PCC, the deficient clotting factors can be replenished, helping to reverse the anticoagulant effects of warfarin more rapidly. PCC is preferred over other options because it provides a more targeted and specific reversal of the anticoagulant effect.  Options A (Cryoprecipitate), B (Fresh frozen plasma), and C (Fibrinogen concentrate) contain various clotting factors but do not provide the full spectrum of factors affected by warfarin. Therefore, they are not as effective as PCC in rapidly reversing the anticoagulant effect of warfarin.  Option D (No additional treatment needed) is incorrect because in the case of an intracranial hemorrhage with an elevated INR, prompt reversal of anticoagulation is crucial to minimize further bleeding and neurological damage. |
| **33** | A 76 year old woman has no energy and reports excessive tiredness for the past 3 weeks. She has lost 5 kg in weight over the past 3 months. She drinks 30 units of alcohol per week. She is thin and jaundiced. Her temperature is 37.2°C. She has a palpable epigastric mass and 4 cm liver edge.  Which is the most likely diagnosis?  A. Cholangiocarcinoma  B. Cholecystitis  C. Cirrhosis of the liver  D. Hepatocellular carcinoma  E. Pancreatic adenocarcinoma | Attempt 1  Given the patient's clinical presentation, including excessive tiredness, weight loss, jaundice, palpable epigastric mass, and hepatomegaly, the most likely diagnosis is option E. Pancreatic adenocarcinoma.  Pancreatic adenocarcinoma is a malignant tumor originating from the pancreatic ducts. It is often associated with nonspecific symptoms, such as fatigue, weight loss, and jaundice. The presence of a palpable epigastric mass and hepatomegaly suggests local spread and involvement of adjacent structures, including the liver.  While other conditions, such as cholangiocarcinoma (option A), cholecystitis (option B), cirrhosis of the liver (option C), and hepatocellular carcinoma (option D), may also present with similar symptoms or findings, the combination of weight loss, jaundice, and a palpable epigastric mass points towards pancreatic adenocarcinoma as the most likely diagnosis.  Further diagnostic evaluation, such as imaging studies (e.g., CT scan) and potentially a biopsy, would be necessary to confirm the diagnosis. Prompt referral to an oncologist or gastroenterologist for further management and treatment options would be appropriate in this case. |
|  |  | Attempt 2  Based on the information provided, the most likely diagnosis is indeed E. Pancreatic adenocarcinoma.  Pancreatic adenocarcinoma is a type of cancer that originates in the pancreas and often presents with symptoms such as weight loss, fatigue, jaundice, and a palpable mass in the abdomen. The patient's history of weight loss, excessive tiredness, alcohol consumption, jaundice, thinness, and the presence of an epigastric mass align with the typical clinical presentation of pancreatic adenocarcinoma. The palpable epigastric mass could be the tumor itself.  While the other options (A. Cholangiocarcinoma, B. Cholecystitis, C. Cirrhosis of the liver, D. Hepatocellular carcinoma) are relevant considerations, the provided symptoms and findings appear to correlate more closely with pancreatic adenocarcinoma. It's important to consult a medical professional for an accurate diagnosis and appropriate management. |
|  |  | Attempt 3  Based on the given clinical presentation, the most likely diagnosis in this case would be pancreatic adenocarcinoma (Option E).  The patient's symptoms of excessive tiredness, weight loss, and jaundice, along with the presence of a palpable epigastric mass and an enlarged liver edge, are suggestive of pancreatic adenocarcinoma. This type of cancer often presents with nonspecific symptoms such as fatigue, weight loss, and jaundice. The palpable mass and enlarged liver can be indicative of local tumor invasion.  While other options such as cholangiocarcinoma (Option A), cholecystitis (Option B), cirrhosis of the liver (Option C), and hepatocellular carcinoma (Option D) may also cause similar symptoms, the combination of weight loss, jaundice, and a palpable epigastric mass is more consistent with pancreatic adenocarcinoma. |
| **34** | A 76 year old woman with hypertension is taking amlodipine 10 mg daily. A 24 hour BP measurement shows a mean BP of 168/90 mmHg. Investigations: Sodium 135 mmol/L (135–146) Potassium 4.0 mmol/L (3.5–5.3) Urea 7 mmol/L (2.5–7.8) Creatinine 100 μmol/L (60–120) eGFR 68 mL/min/1.73 m2(>60) Urinary albumin : creatinine ratio 50 mg/mmol (<3.5)  Which class of antihypertensive should be added?  A. ACE inhibitor  B. Alpha blocker  C. Beta blocker  D. Loop diuretic  E. Thiazide-like diuretic | Attempt 1  Based on the patient's clinical presentation and 24-hour BP measurement, the most appropriate class of antihypertensive to add would be option A. ACE inhibitor.  The patient has hypertension with a mean BP of 168/90 mmHg on amlodipine 10 mg daily, which indicates inadequate control of blood pressure. Adding another antihypertensive medication from a different class is recommended to achieve better blood pressure control.  ACE inhibitors are a commonly used class of antihypertensive medications. They have been shown to effectively reduce blood pressure and have additional benefits in patients with hypertension, such as renal protection and cardiovascular risk reduction. ACE inhibitors are particularly beneficial in patients with comorbid conditions, such as diabetes or chronic kidney disease, as they provide additional renal and cardiovascular protective effects.  Therefore, in this patient with hypertension and inadequate blood pressure control on amlodipine, adding an ACE inhibitor as a second-line agent would be a suitable choice. However, the final decision should be made in consultation with the patient's healthcare provider, considering individual patient factors, contraindications, and potential drug interactions. |
|  |  | Attempt 2  Based on the provided information, the most appropriate choice for adding an antihypertensive medication would be A. ACE inhibitor.  The patient is already on amlodipine for hypertension, but their 24-hour blood pressure measurement still shows elevated blood pressure. Additionally, the patient has indications of kidney involvement with an elevated urinary albumin to creatinine ratio (a marker of kidney damage) and a slightly reduced estimated glomerular filtration rate (eGFR). Both of these findings suggest that kidney protection is important in managing their hypertension.  ACE inhibitors are commonly used in hypertensive patients with kidney involvement as they not only lower blood pressure but also have a kidney-protective effect. They can help slow down the progression of kidney damage and reduce albuminuria. Therefore, adding an ACE inhibitor to the treatment regimen could be beneficial in this case.  However, treatment decisions should always be made in consultation with a medical professional who has a comprehensive understanding of the patient's medical history, overall health, and individual needs. |
|  |  | Attempt 3  Based on the given information, the most appropriate class of antihypertensive to add in this case would be an ACE inhibitor (Option A).  The patient's 24-hour BP measurement shows an elevated mean blood pressure of 168/90 mmHg, indicating inadequate blood pressure control despite being on amlodipine 10 mg daily. Adding another antihypertensive agent is necessary to achieve better blood pressure control.  ACE inhibitors are a recommended first-line treatment for hypertension, particularly in patients with comorbid conditions such as diabetes or chronic kidney disease. The patient's normal urinary albumin-to-creatinine ratio and relatively preserved renal function suggest that an ACE inhibitor would be an appropriate choice in this case.  Alpha blockers (Option B), beta blockers (Option C), and loop diuretics (Option D) may also be considered in specific clinical situations, but ACE inhibitors are generally preferred as an initial add-on therapy for hypertension management. Thiazide-like diuretics (Option E) are typically used as first-line or add-on therapy in patients with uncomplicated hypertension, but in this case, an ACE inhibitor would be more suitable. |
| **35** | A 70 year old man is an inpatient on the cardiology ward. He has worsening breathlessness that woke him up last night. His pulse rate is 99 bpm, BP 160/100 mmHg and respiratory rate 20 breaths per minute. Auscultation of the chest reveals bibasal crepitations, and there is dullness to percussion of both bases. Chest X-ray shows small bilateral pleural effusions with upper lobe blood vessel diversion.  Which is the most appropriate diagnostic investigation?  A. Coronary angiography  B. CT pulmonary angiography  C. ECG  D. Echocardiography  E. Serum D dimer | Attempt 1  Given the patient's presentation of worsening breathlessness, bibasal crepitations, and small bilateral pleural effusions with upper lobe blood vessel diversion on chest X-ray, the most appropriate diagnostic investigation would be option D. Echocardiography.  The clinical findings, including the presence of bibasal crepitations and pleural effusions, suggest the possibility of congestive heart failure (CHF) as the underlying cause of the patient's symptoms. Echocardiography is a valuable tool in evaluating the structure and function of the heart, which can help determine the presence and severity of cardiac dysfunction.  Echocardiography can assess various parameters related to cardiac function, such as left ventricular ejection fraction (LVEF), valvular abnormalities, and the presence of ventricular or atrial enlargement. It can help identify the underlying cause of heart failure, such as systolic dysfunction, diastolic dysfunction, or valvular pathology.  By performing echocardiography, the healthcare provider can evaluate the patient's cardiac function and determine the presence of any structural or functional abnormalities that may be contributing to the symptoms of heart failure. This information will guide further management and treatment decisions, such as optimizing heart failure medications or considering interventions like valve replacement or revascularization if indicated.  While other investigations, such as coronary angiography (option A), CT pulmonary angiography (option B), ECG (option C), and serum D-dimer (option E), may have their roles in specific clinical scenarios, echocardiography is the most appropriate initial diagnostic investigation to evaluate the cardiac function and structure in a patient with suspected heart failure. |
|  |  | Attempt 2  Based on the provided information, the most appropriate diagnostic investigation would be D. Echocardiography.  The patient's presentation of worsening breathlessness, elevated heart rate, elevated blood pressure, bibasal crepitations, dullness to percussion of both lung bases, and small bilateral pleural effusions with upper lobe blood vessel diversion on chest X-ray suggests the possibility of heart failure with associated pleural effusions.  Echocardiography is a valuable tool in evaluating heart function and structure. It can help determine if the patient's symptoms are due to heart failure, assess the overall heart function (ejection fraction), identify any structural abnormalities, and provide information about the cause of the symptoms. In this case, it can help confirm or rule out heart failure as the underlying cause of the patient's symptoms and guide appropriate management.  While other investigations like ECG, CT pulmonary angiography, and serum D dimer might be relevant in specific clinical scenarios, given the symptoms and findings described, echocardiography would provide the most targeted and relevant information in this case. However, the final decision should be made by the attending healthcare professionals based on the patient's individual situation. |
|  |  | Attempt 3  Based on the provided information, the most appropriate diagnostic investigation for this patient would be Echocardiography (Option D).  The patient's symptoms of worsening breathlessness, bibasal crepitations, and pleural effusions, along with dullness to percussion of both lung bases, suggest the presence of congestive heart failure (CHF). CHF is a common cardiac condition characterized by fluid accumulation in the lungs and peripheral edema.  Echocardiography is a non-invasive imaging test that can assess the structure and function of the heart. It can help evaluate cardiac function, identify structural abnormalities, assess the presence of valvular disease, and estimate the ejection fraction. In this case, echocardiography would be valuable in assessing cardiac function, identifying any structural abnormalities that may contribute to the patient's symptoms, and determining the underlying cause of the CHF.  Coronary angiography (Option A) is primarily used to assess the coronary arteries for the presence of blockages or narrowing. It may be indicated if the patient's symptoms and clinical presentation suggest underlying coronary artery disease as a possible cause of CHF, but in this scenario, the presentation is more consistent with fluid overload rather than acute coronary syndrome.  CT pulmonary angiography (Option B) is used to evaluate for pulmonary embolism, which typically presents with acute-onset dyspnea and may be associated with pleural effusions. However, in this case, the patient's symptoms and examination findings are more indicative of congestive heart failure rather than a pulmonary embolism.  An ECG (Option C) is a useful initial test in assessing cardiac function and identifying any acute cardiac abnormalities, such as arrhythmias or myocardial ischemia. However, echocardiography would provide more comprehensive information about the patient's cardiac structure and function in this case.  Serum D-dimer (Option E) is a blood test used as a screening tool for suspected venous thromboembolism, such as deep vein thrombosis or pulmonary embolism. It is not the most appropriate diagnostic investigation for this patient, as the clinical presentation and findings are more consistent with congestive heart failure.  Therefore, based on the given information, echocardiography (Option D) would be the most appropriate diagnostic investigation to evaluate the patient's cardiac function and determine the underlying cause of the congestive heart failure. |
| **36** | An 80 year old man has sudden onset of loss of vision in his right eye. He has hypertension and a previous stroke. His visual acuity is hand movements only in the right eye and 6/9 in left eye. The right eye has an afferent pupillary defect; left eye pupil responses are normal. On fundoscopy there is a red spot at the right macula.  Which is the most likely diagnosis?  A. Anterior ischaemic optic neuropathy  B. Branch retinal vein occlusion  C. Central retinal artery occlusion  D. Macular degeneration  E. Retinal detachment | Attempt 1  Based on the sudden onset of loss of vision in the right eye, presence of an afferent pupillary defect, and a red spot at the right macula on fundoscopy, the most likely diagnosis in this case is option C. Central retinal artery occlusion.  Central retinal artery occlusion (CRAO) is characterized by sudden, painless, and often profound loss of vision in one eye. It is typically associated with a relative afferent pupillary defect (Marcus Gunn pupil) due to the decreased or absent light response in the affected eye. Fundoscopy findings in CRAO may include a pale retina with a cherry-red spot at the fovea, indicating ischemia to the central macula.  Anterior ischaemic optic neuropathy (option A) typically presents with sudden painless loss of vision, but it is associated with optic disc swelling (papilledema) rather than a cherry-red spot on fundoscopy. Branch retinal vein occlusion (option B) is characterized by sudden painless loss of vision with retinal hemorrhages and venous dilation in the affected area. Macular degeneration (option D) typically presents with gradual central vision loss, and retinal detachment (option E) is characterized by a sudden onset of floaters, flashes of light, and a curtain-like visual field defect.  Therefore, based on the clinical presentation and findings, the most likely diagnosis in this case is central retinal artery occlusion (CRAO). Immediate evaluation and management are crucial in order to preserve any remaining vision and to prevent further complications. |
|  |  | Attempt 2  Based on the provided information, the most likely diagnosis would be C. Central retinal artery occlusion.  The sudden onset of loss of vision in one eye, especially with the presence of an afferent pupillary defect (also known as Marcus Gunn pupil), along with a red spot at the macula, strongly suggests a blockage of the central retinal artery. The red spot at the macula is known as a cherry-red spot and is a characteristic finding in central retinal artery occlusion.  This condition occurs when the blood supply to the retina's central artery is interrupted, leading to sudden and severe vision loss. Risk factors like hypertension, previous stroke, and age further support the possibility of a vascular-related issue.  While the other options (A. Anterior ischaemic optic neuropathy, B. Branch retinal vein occlusion, D. Macular degeneration, E. Retinal detachment) could be considered, the combination of symptoms and findings described align more closely with central retinal artery occlusion. It's important for the individual to seek immediate medical attention, as central retinal artery occlusion is a serious condition requiring prompt evaluation and potential intervention. |
|  |  | Attempt 3  Based on the provided information, the most likely diagnosis for this patient's sudden onset of loss of vision in the right eye is Central retinal artery occlusion (Option C).  Central retinal artery occlusion (CRAO) is a serious condition characterized by the sudden blockage of the central retinal artery, which leads to a severe and often painless loss of vision in the affected eye. It typically presents with sudden, profound, and often permanent vision loss. The presence of an afferent pupillary defect (relative afferent pupillary defect or Marcus Gunn pupil) suggests a disruption of the optic nerve pathway and further supports the diagnosis of CRAO.  The description of a red spot at the right macula on fundoscopy is consistent with the finding of a cherry-red spot, which is a classic feature of CRAO. The central retinal artery supplies the macula, and when the blood flow is blocked, the macula appears relatively pale surrounded by a cherry-red spot due to the contrast between the pale ischemic area and the remaining vascularized retina.  Anterior ischemic optic neuropathy (Option A) typically presents with sudden painless vision loss, but it primarily affects the optic disc rather than the macula. It may present with optic disc swelling (papilledema) or optic disc pallor.  Branch retinal vein occlusion (Option B) would cause a localized loss of vision in a specific area of the retina supplied by the affected branch retinal vein, rather than a sudden, profound loss of vision as described in this case.  Macular degeneration (Option D) usually presents with a gradual loss of central vision rather than a sudden onset of severe vision loss.  Retinal detachment (Option E) can cause sudden vision loss, but it typically presents with the perception of floaters, flashes of light, and a curtain-like visual field defect. The presence of a red spot at the macula is not a characteristic finding of retinal detachment.  Therefore, based on the given information, the most likely diagnosis for this patient's sudden onset of loss of vision in the right eye, with an afferent pupillary defect and a red spot at the macula, is Central retinal artery occlusion (Option C). |
| **37** | A 72 year old woman has had inability to sleep well for the past 3 years. She gets to sleep by 23:00 but wakes up two or three times in the night and gets up by 07:00. Her husband says that she doesn't snore. Her BMI is 23 kg/m2. She carries out her normal daytime activities with no daytime somnolence. She is otherwise well. Her MMSE (Mini Mental State Examination) score is 27/30.  Which is the most likely cause of her insomnia?  A. Depression  B. Early stages of dementia  C. Hypomania  D. Normal age related sleep pattern  E. Obstructive sleep apnoea | Attempt 1  Based on the provided information, the most likely cause of her insomnia is option D: Normal age-related sleep pattern.  Insomnia, or difficulty sleeping, is a common complaint among older adults. As people age, their sleep patterns tend to change, and it is normal for them to experience more fragmented sleep, including waking up multiple times during the night. In this case, the patient falls asleep at a regular time and is able to carry out normal daytime activities without experiencing daytime somnolence. Her MMSE score of 27/30 indicates that she has good cognitive function.  The absence of snoring and the lack of symptoms suggestive of other sleep disorders, such as excessive daytime sleepiness or witnessed apneas, make options A (depression) and E (obstructive sleep apnea) less likely. Additionally, there are no indications of hypomania (option C) or early stages of dementia (option B) based on the information provided.  Given the patient's normal activities and cognitive function, as well as the absence of other concerning symptoms, the most likely cause of her insomnia is a normal age-related sleep pattern. It is important to educate and reassure the patient about age-related changes in sleep and provide guidance on sleep hygiene practices to improve the quality of her sleep. |
|  |  | Attempt 2  Based on the provided information, the most likely cause of her insomnia is D. Normal age-related sleep pattern.  As people age, their sleep patterns can change. It's not uncommon for older individuals to experience fragmented sleep, waking up multiple times during the night. This can lead to perceptions of poor sleep quality, even though they may still feel well-rested during the day.  The woman's ability to carry out normal daytime activities without somnolence, her MMSE score of 27/30 (indicating a relatively high cognitive function), and the absence of snoring or significant daytime symptoms suggest that her sleep disturbances may be a part of the normal aging process rather than being linked to a more serious underlying condition like depression, dementia, hypomania, or obstructive sleep apnea.  It's important to consider that other factors, such as lifestyle habits and sleep hygiene practices, can also contribute to sleep disturbances. If the woman is concerned about her sleep or if her insomnia is causing significant distress, it's recommended for her to consult a healthcare professional for a comprehensive evaluation and personalized advice. |
|  |  | Attempt 3  Based on the information provided, the most likely cause of the 72-year-old woman's insomnia is D. Normal age-related sleep pattern.  The patient's inability to sleep well and waking up two or three times during the night is a common occurrence in older adults. As people age, their sleep patterns tend to change, and it is not uncommon for them to experience fragmented sleep with multiple awakenings during the night. The patient's ability to carry out normal daytime activities without daytime somnolence also suggests that her sleep disturbances are not significantly impacting her daily functioning.  Depression (Option A) can cause insomnia, but there are no other signs or symptoms suggestive of depression mentioned in the scenario. The patient's MMSE score of 27/30 indicates that her cognitive function is relatively intact, making depression less likely.  Early stages of dementia (Option B) can sometimes cause sleep disturbances, but there are no other cognitive impairments mentioned in the scenario, and the MMSE score of 27/30 suggests good cognitive functioning.  Hypomania (Option C) is characterized by elevated mood, increased energy, and decreased need for sleep. There are no other symptoms or indications of hypomania mentioned in the scenario.  Obstructive sleep apnea (Option E) is unlikely in this case because the patient's husband states that she does not snore, which is a common symptom of obstructive sleep apnea.  Therefore, based on the information provided, the most likely cause of the woman's insomnia is D. Normal age-related sleep pattern. It is important to note that other causes of insomnia should be considered if her symptoms worsen or if additional symptoms arise. |
| **38** | A 35 year old man visits his GP with 3 days of a red, painful left eye with no discharge. There is a diffuse area of redness in the medial aspect of his left sclera. His pupils and visual acuity are normal.  Which is the most appropriate management?  A. Arrange assessment in emergency eye clinic  B. Prescribe chloramphenicol eye drops  C. Prescribe corticosteroid eye drops  D. Prescribe topical aciclovir  E. Reassure patient that it will resolve spontaneously | Attempt 1  Based on the given information, the most appropriate management for the 35-year-old man with a red, painful left eye and no discharge would be option E: Reassure the patient that it will resolve spontaneously.  The presentation of a red, painful eye with no discharge and normal visual acuity is suggestive of a self-limiting condition called episcleritis. Episcleritis is an inflammatory condition that affects the episcleral tissue, which lies between the conjunctiva and the sclera.  Episcleritis typically resolves on its own without any specific treatment. It is usually a benign and self-limiting condition that does not require any intervention. Reassurance can be provided to the patient, along with advice to use artificial tears for symptom relief if necessary.  Options A, B, C, and D are not appropriate for this scenario. Referral to the emergency eye clinic (option A) is not necessary unless there are severe symptoms or signs of a more serious condition. Prescribing chloramphenicol eye drops (option B) is not indicated as there is no evidence of infection. Corticosteroid eye drops (option C) are typically reserved for more severe or chronic cases of episcleritis, and topical aciclovir (option D) would be relevant if herpetic keratitis was suspected, but there is no indication for it based on the given information.  It is important to note that a thorough examination by a healthcare professional is always recommended to confirm the diagnosis and rule out other potential causes. |
|  |  | Attempt 2  Based on the provided information, the most appropriate management in this case would be A. Arrange assessment in an emergency eye clinic.  The diffuse area of redness in the medial aspect of the left sclera, along with the painful red eye and absence of discharge, could indicate a condition that needs further evaluation by an eye specialist. While some mild cases of conjunctivitis (pink eye) might resolve on their own or with over-the-counter treatments, the sudden onset of a red and painful eye without discharge raises the possibility of other more serious conditions that require prompt attention.  Conditions such as scleritis or uveitis, which involve inflammation of the deeper layers of the eye, can present with these symptoms and can have potentially serious consequences for vision if not managed appropriately. An emergency eye clinic assessment would be the most prudent course of action to accurately diagnose the condition and determine the appropriate treatment.  It's important for healthcare professionals to evaluate eye symptoms comprehensively to ensure the best possible outcomes for the patient's eye health. |
|  |  | Attempt 3  Based on the presented information, the most appropriate management for the 35-year-old man with a red, painful left eye and no discharge is A. Arrange assessment in the emergency eye clinic.  The diffuse area of redness in the medial aspect of the left sclera suggests inflammation or irritation. It is important to rule out any serious underlying causes and to obtain a proper diagnosis. Referring the patient to the emergency eye clinic allows for a thorough evaluation by an ophthalmologist who can assess the condition more comprehensively and provide appropriate treatment if necessary.  Prescribing chloramphenicol eye drops (Option B) may be considered for bacterial conjunctivitis with discharge, but since the patient has no discharge and the presentation is more consistent with scleritis or another inflammatory condition, it is not the most appropriate initial management.  Prescribing corticosteroid eye drops (Option C) should be done cautiously and under the supervision of an ophthalmologist. Corticosteroids can be beneficial for certain inflammatory conditions, but they can also worsen certain infections or other causes of red eye. Therefore, it is important to have a proper evaluation before considering this treatment option.  Prescribing topical aciclovir (Option D) would be appropriate if there were signs or suspicion of herpetic keratitis, such as dendritic ulcers or vesicular lesions on the cornea. However, since the presentation does not indicate these findings, it is not the most appropriate initial management.  Reassuring the patient that it will resolve spontaneously (Option E) is not recommended without further assessment. The symptoms of a red, painful eye can be caused by various conditions, some of which may require treatment to prevent complications or further damage.  Therefore, in this case, arranging assessment in the emergency eye clinic (Option A) is the most appropriate management to ensure an accurate diagnosis and appropriate treatment. |
| **39** | An 18 year old woman is found dead, sitting in front of a gas fire that is still burning. The flue that carries gases away from the fire is found to be blocked.  Which is the principal mechanism of action of the poison involved in her death?  A. Binding to the site on haemoglobin normally occupied by oxygen  B. Converting carbon dioxide to carbonic acid in the cytoplasm of peripheral cells  C. Converting the iron in haem to an iron salt  D. Damaging the lipid bilayer of alveolar pneumocytes  E. Inhibiting cytochrome enzyme systems | Attempt 1  Based on the information provided, the principal mechanism of action of the poison involved in the woman's death would be option A: Binding to the site on hemoglobin normally occupied by oxygen.  When a flue is blocked and there is incomplete combustion of fuel, such as in this case with the gas fire, it can lead to the release of toxic gases, particularly carbon monoxide (CO). Carbon monoxide is a colorless, odorless, and tasteless gas that can be highly toxic.  Carbon monoxide has a high affinity for hemoglobin in the blood, binding to the same site that normally binds oxygen. This binding is much stronger than the binding of oxygen, leading to the formation of carboxyhemoglobin (COHb). As a result, the oxygen-carrying capacity of hemoglobin is compromised, leading to tissue hypoxia and potentially fatal consequences.  The lack of oxygen delivery to vital organs, including the brain and heart, can lead to cellular damage and eventually death. This mechanism explains the cause of death in cases of carbon monoxide poisoning.  Options B, C, D, and E are not directly related to the mechanism of action of carbon monoxide poisoning. Carbon dioxide (CO2) does not convert to carbonic acid in the cytoplasm of peripheral cells (option B). Carbon monoxide does not directly convert the iron in hemoglobin to an iron salt (option C). Damaging the lipid bilayer of alveolar pneumocytes (option D) is not a primary mechanism of carbon monoxide toxicity. Inhibiting cytochrome enzyme systems (option E) is not the principal mechanism of action of carbon monoxide. |
|  |  | Attempt 2  Based on the provided information, the most likely mechanism of action involved in her death would be E. Inhibiting cytochrome enzyme systems.  A blocked flue in a gas fire can lead to incomplete combustion and the release of toxic gases like carbon monoxide (CO). Carbon monoxide is a colorless and odorless gas that is known for its ability to bind to hemoglobin in red blood cells, preventing the binding of oxygen. This is represented by choice A ("Binding to the site on haemoglobin normally occupied by oxygen").  However, the principal mechanism of action for carbon monoxide toxicity is its ability to inhibit cytochrome enzyme systems. Cytochrome enzymes, specifically cytochrome c oxidase in the mitochondria, are crucial for cellular respiration and the production of energy (ATP) in cells. Carbon monoxide binds to these enzymes and disrupts the electron transport chain, leading to cellular hypoxia (lack of oxygen) and metabolic dysfunction. This ultimately affects various organs, including the heart and brain, and can lead to death.  Carbon monoxide poisoning is a serious condition that requires immediate medical attention. It's a reminder of the importance of proper ventilation and monitoring gas appliances to prevent such tragic incidents. |
|  |  | Attempt 3  Based on the information provided, the principal mechanism of action of the poison involved in the woman's death is A. Binding to the site on hemoglobin normally occupied by oxygen.  When the flue that carries gases away from the gas fire is blocked, it can lead to the accumulation of toxic gases, most notably carbon monoxide (CO). Carbon monoxide has a high affinity for hemoglobin, the molecule responsible for transporting oxygen in the blood. Carbon monoxide binds to the site on hemoglobin that is normally occupied by oxygen, forming carboxyhemoglobin (COHb). This binding reduces the capacity of hemoglobin to carry oxygen, leading to tissue hypoxia.  The affinity of carbon monoxide for hemoglobin is approximately 200 times greater than that of oxygen. As a result, even relatively low levels of carbon monoxide in the environment can lead to significant poisoning and potentially fatal outcomes.  The other options listed are not directly related to the mechanism of action of carbon monoxide poisoning:  B. Converting carbon dioxide to carbonic acid in the cytoplasm of peripheral cells: This does not describe the mechanism of carbon monoxide poisoning. Carbon monoxide does not convert carbon dioxide to carbonic acid.  C. Converting the iron in hem to an iron salt: Carbon monoxide does not convert the iron in hemoglobin to an iron salt.  D. Damaging the lipid bilayer of alveolar pneumocytes: Carbon monoxide primarily affects the binding capacity of hemoglobin and does not directly damage the lipid bilayer of alveolar pneumocytes.  E. Inhibiting cytochrome enzyme systems: While carbon monoxide can interfere with cellular respiration and the activity of cytochrome enzymes, it is not the principal mechanism of action in this scenario. The primary mechanism is the binding of carbon monoxide to hemoglobin, reducing oxygen-carrying capacity.  Therefore, option A, binding to the site on hemoglobin normally occupied by oxygen, is the principal mechanism of action of the poison involved in her death in this case. |
| **40** | A 35 year old man with type 1 diabetes mellitus has burning pain in his feet and difficulty sleeping. He has retinopathy and nephropathy. Investigation: eGFR 28 mL/min/1.73m2(> 60)  Which is the most appropriate management?  A. Acupuncture  B. Amitriptyline  C. Duloxetine  D. Physiotherapy  E. Sodium valproate | Attempt 1  The most appropriate management for the 35-year-old man with type 1 diabetes mellitus, burning pain in his feet, and difficulty sleeping, along with an eGFR of 28 mL/min/1.73m², would be option C: Duloxetine.  The symptoms described by the patient, including burning pain in the feet and difficulty sleeping, are consistent with diabetic peripheral neuropathy. Diabetic neuropathy is a common complication of diabetes that affects the peripheral nerves and can cause various symptoms, including pain, numbness, and tingling in the feet and hands.  Duloxetine is a medication commonly used to manage diabetic neuropathy. It belongs to a class of drugs called serotonin-norepinephrine reuptake inhibitors (SNRIs) and works by increasing the levels of certain neurotransmitters in the brain and spinal cord, which can help reduce neuropathic pain.  Acupuncture (option A) may have some benefits in managing certain types of pain, but its effectiveness for diabetic neuropathy is uncertain, and it may not be the most appropriate initial management in this case.  Amitriptyline (option B) is another medication used for neuropathic pain, but it is typically considered a second-line treatment. Duloxetine is often preferred due to its favorable side effect profile and its effectiveness specifically for diabetic neuropathy.  Physiotherapy (option D) may play a role in managing other aspects of diabetes care, such as promoting physical activity and addressing musculoskeletal issues, but it is not the primary treatment for diabetic neuropathy.  Sodium valproate (option E) is not indicated for the management of diabetic neuropathy. It is primarily used for conditions such as epilepsy and bipolar disorder.  It is important for the patient to have a comprehensive management plan for his diabetes, including glycemic control, blood pressure management, and regular monitoring of renal function. Close collaboration with a healthcare provider, such as a primary care physician or endocrinologist, is essential for the optimal management of his condition. |
|  |  | Attempt 2  Based on the information provided, the most appropriate management for this individual's symptoms would be C. Duloxetine.  The patient's symptoms of burning pain in the feet and difficulty sleeping are indicative of diabetic neuropathy, a common complication of diabetes. Diabetic neuropathy can cause nerve damage and result in painful sensations in the extremities, like burning pain. Additionally, the patient's retinopathy and nephropathy suggest the presence of diabetes-related complications, which can contribute to the development of neuropathy.  Duloxetine is a medication commonly used to manage neuropathic pain. It is a serotonin-norepinephrine reuptake inhibitor (SNRI) that can help alleviate the symptoms of neuropathic pain, including burning pain. It's important to note that proper blood sugar control and management of diabetes are crucial to preventing and mitigating the progression of complications.  However, the final decision about treatment should be made in consultation with a healthcare professional who is familiar with the patient's medical history and can provide personalized recommendations. |
|  |  | Attempt 3  The most appropriate management for a 35-year-old man with type 1 diabetes mellitus, burning pain in his feet, and difficulty sleeping, along with evidence of nephropathy (eGFR 28 mL/min/1.73m²), would be option C: Duloxetine.  The symptoms described, including burning pain in the feet, are suggestive of diabetic peripheral neuropathy, a common complication of diabetes. Duloxetine is a medication commonly used to manage neuropathic pain. It belongs to a class of drugs known as serotonin-norepinephrine reuptake inhibitors (SNRIs) and has been shown to be effective in reducing neuropathic pain symptoms.  Option A, acupuncture, may provide some relief for certain individuals with neuropathic pain, but its effectiveness varies, and it is not considered a first-line treatment.  Option B, amitriptyline, is an antidepressant that is sometimes used to manage neuropathic pain. However, duloxetine is generally preferred over amitriptyline for diabetic peripheral neuropathy due to its more specific targeting of pain pathways.  Option D, physiotherapy, may be beneficial for other aspects of diabetic management, such as improving physical function and mobility, but it is not a primary treatment for neuropathic pain.  Option E, sodium valproate, is not typically used for the management of diabetic peripheral neuropathy. Sodium valproate is primarily indicated for the treatment of epilepsy and bipolar disorder.  Therefore, based on the information provided, duloxetine (option C) would be the most appropriate management for the patient's symptoms of burning pain in his feet and difficulty sleeping associated with diabetic peripheral neuropathy. It is important for the patient to consult with their healthcare provider for a proper evaluation and to discuss the most suitable treatment plan for their specific condition. |
| **41** | A 24 year old woman has tiredness, bloating and weight loss with bouts of offensive smelling diarrhoea. Abdominal examination is normal. Investigations: Haemoglobin 10.0 g/L (115–150) Mean cell volume (MCV) 78 fL (80–96) Platelets 350 × 109/L (150–400) Duodenal biopsy shows flattening of villi and increased lymphocytes in the lamina propria and surface epithelium. In addition, there is gross crypt hyperplasia.  Which is the most likely diagnosis?  A. Carcinoid tumour  B. Coeliac disease  C. Collagenous enteropathy  D. Crohn's disease  E. Pseudomembranous enteropathy | Attempt 1  The most likely diagnosis for the 24-year-old woman with tiredness, bloating, weight loss, offensive smelling diarrhea, and the findings of flattening of villi, increased lymphocytes in the lamina propria and surface epithelium, and gross crypt hyperplasia on duodenal biopsy is option B: Coeliac disease.  Coeliac disease, also known as celiac disease, is an autoimmune disorder characterized by an abnormal immune response to gluten, a protein found in wheat, barley, and rye. The ingestion of gluten triggers an immune-mediated reaction in the small intestine, leading to inflammation, villous atrophy (flattening of the villi), and crypt hyperplasia.  The symptoms described by the patient, including tiredness, bloating, weight loss, and offensive smelling diarrhea, are common manifestations of coeliac disease. Malabsorption of nutrients, particularly fat, can result in weight loss and bulky, foul-smelling stools. Bloating and tiredness are also frequently reported symptoms.  The laboratory findings of low hemoglobin (10.0 g/L) and a decreased mean cell volume (MCV) (78 fL) are indicative of microcytic anemia, which can be seen in coeliac disease due to iron deficiency secondary to malabsorption.  Carcinoid tumors (option A) typically present with symptoms related to hormone overproduction, such as flushing, diarrhea, and wheezing. These symptoms are usually episodic and not associated with villous atrophy and crypt hyperplasia seen in coeliac disease.  Collagenous enteropathy (option C) is a rare condition characterized by the presence of a thickened subepithelial collagen band in the intestine. It can lead to chronic diarrhea and malabsorption, but the specific histological findings described in the case (increased lymphocytes, villous flattening, and crypt hyperplasia) are not consistent with this condition.  Crohn's disease (option D) is a chronic inflammatory bowel disease that can affect any part of the gastrointestinal tract. It typically presents with symptoms such as abdominal pain, diarrhea, and weight loss, but the specific histological findings described in the case (flattening of villi and increased lymphocytes) are not characteristic of Crohn's disease.  Pseudomembranous enteropathy (option E) is typically caused by Clostridium difficile infection and is characterized by the presence of pseudomembranes on endoscopy. It does not typically present with the described findings on duodenal biopsy and is not associated with gluten-related enteropathy seen in coeliac disease.  Confirmation of the diagnosis of coeliac disease can be obtained through serological tests, such as tissue transglutaminase (tTG) antibodies and anti-endomysial antibodies (EMA), and may require further evaluation, such as genetic testing and/or gluten challenge, in some cases. A gluten-free diet is the mainstay of treatment for coeliac disease to alleviate symptoms and promote intestinal healing. |
|  |  | Attempt 2  Based on the provided information, the most likely diagnosis is B. Coeliac disease.  The patient's symptoms of tiredness, bloating, weight loss, offensive smelling diarrhea, and the findings from duodenal biopsy (flattening of villi, increased lymphocytes in the lamina propria and surface epithelium, and gross crypt hyperplasia) are characteristic of coeliac disease.  Coeliac disease is an autoimmune disorder triggered by the ingestion of gluten, a protein found in wheat, barley, and rye. It leads to damage in the small intestine, resulting in malabsorption of nutrients, which can explain the low hemoglobin (anemia) and low mean cell volume (MCV) values. The duodenal biopsy findings of villous atrophy, increased intraepithelial lymphocytes, and crypt hyperplasia are consistent with coeliac disease.  The other options (A. Carcinoid tumour, C. Collagenous enteropathy, D. Crohn's disease, E. Pseudomembranous enteropathy) do not fit the described symptoms and biopsy findings as well as coeliac disease does.  It's important for the patient to work closely with a healthcare professional to confirm the diagnosis and manage coeliac disease through dietary adjustments. |
|  |  | Attempt 3  Based on the presented clinical findings and investigations, the most likely diagnosis for the 24-year-old woman is option B: Coeliac disease.  Coeliac disease is an autoimmune disorder characterized by an immune-mediated reaction to gluten, a protein found in wheat, barley, and rye. The symptoms of tiredness, bloating, weight loss, and offensive smelling diarrhoea are consistent with the gastrointestinal manifestations of coeliac disease.  The laboratory findings of low hemoglobin (anemia) and a decreased mean cell volume (MCV) suggest iron deficiency anemia, which is commonly associated with coeliac disease due to malabsorption of nutrients.  The duodenal biopsy findings of flattened villi, increased lymphocytes in the lamina propria and surface epithelium, and crypt hyperplasia are classic histological features of coeliac disease.  Option A, carcinoid tumor, is unlikely as it typically presents with different symptoms and would not cause the characteristic findings seen in the duodenal biopsy.  Option C, collagenous enteropathy, is a rare condition characterized by subepithelial collagen deposition in the intestinal mucosa, leading to similar symptoms and histological changes as coeliac disease. However, the most common cause of the reported findings is coeliac disease.  Option D, Crohn's disease, is a chronic inflammatory bowel disease that can cause similar symptoms, but the histological features described in the duodenal biopsy are not typical of Crohn's disease.  Option E, pseudomembranous enteropathy, is associated with Clostridium difficile infection and would not explain the specific histological changes observed in the duodenal biopsy.  Therefore, based on the symptoms, laboratory findings, and characteristic duodenal biopsy results, coeliac disease (option B) is the most likely diagnosis for this patient. It is important for her to follow up with a healthcare provider for further evaluation, confirmation, and appropriate management of the condition, which typically involves a gluten-free diet. |
| **42** | An 87 year old man develops profuse watery diarrhoea 6 days after admission for an infective exacerbation of COPD. He is currently taking oral co-amoxiclav.  Which is the most likely causative organism?  A. Campylobacter jejuni  B. Clostridioides difficile (Clostridium difficile)  C. Escherichia coli  D. Norovirus  E. Salmonella enteriditis | Attempt 1  The most likely causative organism for the 87-year-old man's profuse watery diarrhea occurring 6 days after admission and while taking oral co-amoxiclav is option B: Clostridioides difficile (Clostridium difficile).  Clostridioides difficile is a bacterium known to cause antibiotic-associated diarrhea, particularly in healthcare settings. The use of antibiotics, such as co-amoxiclav, disrupts the normal gut flora, allowing Clostridioides difficile to proliferate and produce toxins that lead to diarrhea.  The symptoms of profuse watery diarrhea occurring after antibiotic use, especially in a hospital setting, raise suspicion for Clostridioides difficile infection. Other common symptoms include abdominal pain, fever, and sometimes pseudomembranous colitis. Elderly individuals are more susceptible to Clostridioides difficile infection due to age-related changes in the gut flora and weakened immune system.  Campylobacter jejuni (option A) is a bacterial pathogen commonly associated with foodborne illnesses and can cause diarrhea, but it is less likely in this scenario as the symptoms occurred in a hospital setting during antibiotic use.  Escherichia coli (option C) is a diverse group of bacteria, some strains of which can cause diarrhea, but it is less commonly associated with antibiotic-associated diarrhea in healthcare settings.  Norovirus (option D) is a highly contagious virus known for causing outbreaks of gastroenteritis, particularly in closed settings such as hospitals, nursing homes, and cruise ships. However, the fact that the symptoms occurred after antibiotic use makes Clostridioides difficile a more likely cause.  Salmonella enteritidis (option E) is a bacterium commonly associated with food poisoning and can cause diarrhea, but it is less likely in this case as the symptoms occurred during hospitalization and antibiotic use.  To confirm the diagnosis of Clostridioides difficile infection, laboratory testing can be performed, such as stool culture, polymerase chain reaction (PCR) for detection of C. difficile toxins, or enzyme immunoassays. Treatment involves discontinuing the offending antibiotic if possible and initiating appropriate antibiotics targeting Clostridioides difficile, such as oral vancomycin or fidaxomicin. |
|  |  | Attempt 2  Given the information provided, the most likely causative organism for the profuse watery diarrhea in this scenario is B. Clostridioides difficile (formerly known as Clostridium difficile).  Antibiotics, particularly broad-spectrum antibiotics like co-amoxiclav, can disrupt the normal balance of gut bacteria, leading to overgrowth of bacteria like Clostridioides difficile. This can result in Clostridioides difficile-associated diarrhea (CDAD), which can range from mild diarrhea to severe colitis.  Among the options provided, Clostridioides difficile is the most common cause of antibiotic-associated diarrhea, especially in healthcare settings like hospitals. The fact that the patient developed the diarrhea after being on antibiotics for an infective exacerbation of COPD supports the possibility of Clostridioides difficile infection.  It's important for healthcare providers to be vigilant about the possibility of CDAD when prescribing antibiotics, especially in elderly patients with underlying health conditions. Proper diagnosis and management are crucial to prevent complications associated with Clostridioides difficile infection. |
|  |  | Attempt 3  The most likely causative organism for the profuse watery diarrhea in the 87-year-old man is option B: Clostridioides difficile (Clostridium difficile).  Clostridioides difficile is a bacterium known to cause antibiotic-associated diarrhea, especially in healthcare settings. The fact that the patient developed diarrhea 6 days after starting oral coamoxiclav (a broad-spectrum antibiotic) suggests the possibility of Clostridioides difficile infection. This bacterium can proliferate in the gut when the normal balance of the intestinal flora is disrupted by antibiotic use, leading to the overgrowth of Clostridioides difficile and subsequent toxin production.  Options A, C, D, and E (Campylobacter jejuni, Escherichia coli, Norovirus, and Salmonella enteriditis) can also cause diarrheal illnesses, but they are less likely in this particular scenario. Campylobacter jejuni and Salmonella enteriditis are commonly associated with foodborne infections and would typically present with symptoms earlier than 6 days after starting antibiotics. Escherichia coli and Norovirus can cause diarrhea, but they are less commonly associated with antibiotic use.  Given the history of antibiotic use and the timing of symptom onset, Clostridioides difficile (option B) is the most likely causative organism in this case. Confirmatory laboratory testing, such as a stool sample analysis for Clostridioides difficile toxins, would be necessary for a definitive diagnosis. Proper management would involve discontinuing the inciting antibiotic (if possible) and initiating appropriate treatment, which may include specific antibiotics effective against Clostridioides difficile, such as metronidazole or vancomycin. |
| **43** | A 22 year old woman has worsening shortness of breath and cough productive of four to five tablespoons of sputum per day. She has a history of childhood pneumonia and recurrent chest infections, and coughed up blood on two occasions many years ago. On auscultation of her chest there are bilateral scattered wheezes and coarse inspiratory crackles.  Which is the most likely diagnosis?  A. Bronchiectasis  B. COPD  C. Lung cancer  D. Pulmonary fibrosis  E. Sarcoidosis | Attempt 1  The most likely diagnosis for the 22-year-old woman with worsening shortness of breath, productive cough, history of childhood pneumonia, recurrent chest infections, and coughing up blood in the past is option A: Bronchiectasis.  Bronchiectasis is a condition characterized by irreversible dilation and damage to the bronchi and bronchioles, leading to chronic inflammation and recurrent respiratory infections. The symptoms commonly include chronic cough, production of large amounts of sputum, shortness of breath, and wheezing. The history of childhood pneumonia and recurrent chest infections are risk factors for developing bronchiectasis.  On auscultation, bilateral scattered wheezes and coarse inspiratory crackles are consistent with the findings in bronchiectasis. The crackles can be attributed to the presence of secretions and mucus plugs within the dilated airways.  COPD (option B) is a chronic lung condition primarily caused by smoking and is characterized by progressive airflow limitation. While chronic bronchitis, a subtype of COPD, can present with cough and sputum production, the onset at such a young age and the history of childhood pneumonia make bronchiectasis a more likely diagnosis.  Lung cancer (option C) is less likely in this case, given the patient's age and the chronicity of symptoms since childhood. However, it is essential to consider this possibility in patients with risk factors such as smoking or exposure to occupational carcinogens.  Pulmonary fibrosis (option D) refers to the scarring and thickening of the lung tissue, leading to progressive and irreversible lung function impairment. While it can present with cough and shortness of breath, the presence of wheezes and crackles suggests bronchiectasis as a more likely diagnosis.  Sarcoidosis (option E) is a multisystem inflammatory disorder characterized by the formation of granulomas in various organs, including the lungs. While it can present with respiratory symptoms such as cough and shortness of breath, the history of childhood pneumonia and recurrent chest infections point more towards bronchiectasis as the primary diagnosis.  Further diagnostic evaluation, such as high-resolution CT scan of the chest and pulmonary function tests, would be necessary to confirm the diagnosis of bronchiectasis and assess the extent of the disease. Treatment typically involves airway clearance techniques, antibiotics for treating infections, bronchodilators, and in some cases, surgical intervention to remove severely damaged bronchi. |
|  |  | Attempt 2  Based on the provided information, the most likely diagnosis is A. Bronchiectasis.  The recurrent chest infections, history of childhood pneumonia, productive cough with significant sputum production, and the presence of coarse inspiratory crackles suggest bronchiectasis. Bronchiectasis is a condition characterized by the permanent dilation of the bronchi and bronchioles due to repeated inflammation and infection. This leads to impaired clearance of mucus, chronic cough, and recurrent respiratory infections.  While other conditions like COPD, pulmonary fibrosis, and lung cancer might cause similar symptoms, the history of childhood pneumonia, recurrent infections, and coughing up blood (hemoptysis) in the past are more indicative of bronchiectasis.  It's important for the patient to undergo proper diagnostic evaluation and management to address the underlying causes and symptoms of bronchiectasis, as well as to prevent further complications. |
|  |  | Attempt 3  The most likely diagnosis for the 22-year-old woman with worsening shortness of breath, productive cough, childhood pneumonia, recurrent chest infections, and a history of coughing up blood is option A: Bronchiectasis.  Bronchiectasis is a condition characterized by the permanent dilation of the bronchi and bronchioles due to damage to the airways. It is often associated with recurrent respiratory infections, such as pneumonia, and can cause chronic cough and sputum production. The history of childhood pneumonia and recurrent chest infections, as well as the presence of wheezes and crackles on auscultation, are consistent with bronchiectasis.  Option B, COPD (chronic obstructive pulmonary disease), is less likely in a 22-year-old individual unless there is a history of significant exposure to respiratory irritants, such as smoking or occupational hazards.  Option C, lung cancer, is less likely in a young individual without risk factors such as smoking or significant environmental exposures.  Option D, pulmonary fibrosis, typically presents with progressive shortness of breath and dry cough. The presence of productive cough and childhood history of pneumonia is not typical of pulmonary fibrosis.  Option E, sarcoidosis, can present with respiratory symptoms, but the history of childhood pneumonia and recurrent chest infections is not typically associated with sarcoidosis.  Given the clinical presentation and history provided, bronchiectasis (option A) is the most likely diagnosis. Further diagnostic evaluation, such as imaging studies (e.g., high-resolution CT scan) and pulmonary function tests, would be needed to confirm the diagnosis. Treatment for bronchiectasis often includes airway clearance techniques, antibiotics for infections, and bronchodilators to alleviate symptoms. |
| **44** | A 52 year old woman has had four episodes of severe epigastric pain associated with vomiting over the past 3 months. The episodes occurred after eating and lasted for about 1 hour before complete resolution. She has a history of type 2 diabetes mellitus and takes metformin. Abdominal examination is normal. Her BMI is 35 kg/m2.  Which investigation is most likely to confirm the diagnosis?  A. Helicobacter stool antigen test  B. Liver function tests  C. Plain abdominal X-ray  D. Ultrasonography of abdomen  E. Upper gastrointestinal endoscopy | Attempt 1  The most appropriate investigation to confirm the diagnosis in this case would be option E: Upper gastrointestinal endoscopy.  The patient's symptoms of severe epigastric pain associated with vomiting after eating, occurring multiple times over the past 3 months, are suggestive of a possible gastric pathology. Upper gastrointestinal endoscopy allows direct visualization of the upper digestive tract, including the esophagus, stomach, and duodenum, and is the gold standard for diagnosing various conditions.  Given the patient's risk factors such as a history of type 2 diabetes mellitus and obesity (BMI of 35 kg/m2), there are several possible causes for her symptoms, including peptic ulcer disease, gastroesophageal reflux disease (GERD), gastritis, or even gastric cancer. Upper gastrointestinal endoscopy can help identify any mucosal abnormalities, such as ulcers, inflammation, or tumors, and allows for tissue sampling (biopsy) if necessary.  The other options listed are less likely to provide a definitive diagnosis in this case:  A. Helicobacter stool antigen test: This test is used to detect the presence of Helicobacter pylori, a bacterium associated with peptic ulcer disease. While it may be relevant in cases of suspected peptic ulcers, it would not provide a comprehensive evaluation of the upper gastrointestinal tract or identify other potential causes of the patient's symptoms.  B. Liver function tests: These tests primarily assess liver function and are not directly relevant to the patient's presenting symptoms of epigastric pain and vomiting.  C. Plain abdominal X-ray: A plain abdominal X-ray is useful for evaluating certain conditions such as bowel obstruction or the presence of calcifications or free air. However, it is unlikely to provide specific information related to the patient's symptoms or confirm a specific diagnosis.  D. Ultrasonography of the abdomen: While ultrasonography can be helpful in evaluating various abdominal organs, such as the liver, gallbladder, and kidneys, it may not provide detailed visualization of the upper gastrointestinal tract or help identify specific pathologies causing the patient's symptoms.  Therefore, considering the patient's symptoms and the need for direct visualization and potential tissue sampling, upper gastrointestinal endoscopy would be the most appropriate investigation to confirm the diagnosis. |
|  |  | Attempt 2  Based on the provided information, the investigation most likely to confirm the diagnosis in this case would be E. Upper gastrointestinal endoscopy.  The woman's recurrent episodes of severe epigastric pain associated with vomiting after eating could be indicative of peptic ulcers or other upper gastrointestinal issues. Upper gastrointestinal endoscopy allows direct visualization of the upper digestive tract, including the stomach and duodenum. This procedure can help identify the presence of ulcers, inflammation, or other abnormalities that might be causing her symptoms.  While other investigations like ultrasonography of the abdomen (option D) might be relevant in certain cases, endoscopy provides a more specific and direct way to visualize the potential underlying issues in the upper gastrointestinal tract, making it a valuable diagnostic tool in this scenario.  The final decision on which investigation to pursue should be made in consultation with a healthcare professional who can consider the patient's medical history, symptoms, and individual circumstances. |
|  |  | Attempt 3  The most appropriate investigation to confirm the diagnosis in this case would be option E: Upper gastrointestinal endoscopy.  Given the patient's history of severe epigastric pain associated with vomiting after eating, the possibility of a gastric or duodenal pathology, such as peptic ulcer disease or gastric outlet obstruction, should be considered. Upper gastrointestinal endoscopy allows direct visualization of the stomach and duodenum, providing an opportunity to identify any structural abnormalities, such as ulcers, strictures, or tumors. It also allows for the collection of biopsy samples for further evaluation and testing.  Option A, Helicobacter stool antigen test, is used to detect the presence of Helicobacter pylori infection, which is associated with peptic ulcer disease. While it can be a useful test, it may not be sufficient to confirm the diagnosis in this case, as other pathologies could be causing the symptoms.  Option B, liver function tests, are not likely to be the most relevant investigation for the given clinical presentation and symptoms.  Option C, plain abdominal X-ray, may not provide sufficient detail to diagnose the underlying cause of the patient's symptoms, especially if the pathology is related to the upper gastrointestinal tract.  Option D, ultrasonography of the abdomen, can be helpful for assessing certain abdominal organs but may not be the most specific or sensitive test for diagnosing the cause of the patient's symptoms.  Therefore, in this scenario, upper gastrointestinal endoscopy (option E) would be the most appropriate investigation to confirm the diagnosis and evaluate for any structural abnormalities or mucosal lesions in the stomach and duodenum. |
| **45** | An 83 year old woman has recurring 'dizzy spells'. The episodes are associated with transient shaking of her hands that is most noticeable before her lunch and evening meals. She has hypertension and type 2 diabetes mellitus. Her medication includes metformin 1 g twice daily, gliclazide 80 mg twice daily and ramipril 10 mg daily. Her BP is 138/82 mmHg supine and 130/78 mmHg erect. Her blood capillary glucose is 6 mmol/L. Investigations: Sodium 136 mmol/L (135-146) Potassium 5.0 mmol/L (3.5-5.3) Urea 3.9 mmol/L (2.5-7.8) Creatinine 77 μmol/L (60-120) Glycated haemoglobin 50 mmol/mol (20-42)  Which is the most appropriate therapeutic change?  A. Increase gliclazide dose  B. Increase metformin dose  C. Reduce gliclazide dose  D. Reduce metformin dose  E. Reduce ramipril dose | Attempt 1  Based on the given information, the most appropriate therapeutic change would be option C: Reduce gliclazide dose.  The patient is experiencing recurring 'dizzy spells' associated with transient shaking of her hands, which are most noticeable before meals. These symptoms are suggestive of hypoglycemia (low blood sugar). The fact that the symptoms occur before meals indicates that they might be related to her antidiabetic medication.  The patient is currently taking gliclazide, a sulfonylurea medication that stimulates insulin release from the pancreas and can potentially lower blood sugar levels. Given her symptoms and the normal blood capillary glucose level of 6 mmol/L, it is likely that she is experiencing episodes of hypoglycemia.  Reducing the dose of gliclazide would help mitigate the risk of hypoglycemic episodes. Adjusting the dose should be done in consultation with her healthcare provider, considering her specific blood glucose levels, overall glycemic control, and any other factors that may influence her diabetes management.  The other options are less appropriate:  A. Increasing gliclazide dose: This would further increase the risk of hypoglycemic episodes and is not recommended.  B. Increasing metformin dose: Metformin is not typically associated with causing hypoglycemia. Increasing the dose would not address the symptoms or the potential cause.  D. Reducing metformin dose: Metformin is not likely to be the cause of the patient's symptoms. Reducing the dose would not address the potential underlying issue of hypoglycemia.  E. Reducing ramipril dose: Ramipril is an angiotensin-converting enzyme (ACE) inhibitor used for hypertension management. It is not directly associated with the symptoms described by the patient. Adjusting the ramipril dose would not address the potential cause of the symptoms.  In summary, reducing the gliclazide dose would be the most appropriate therapeutic change to address the potential hypoglycemic episodes in this patient. However, it is important for the patient to consult with her healthcare provider for individualized guidance and to ensure optimal diabetes management. |
|  |  | Attempt 2  Based on the information provided, the most appropriate therapeutic change would be C. Reduce gliclazide dose.  The patient is experiencing recurring dizzy spells associated with transient shaking of her hands, which could be indicative of hypoglycemia (low blood sugar). Gliclazide is an oral antidiabetic medication that stimulates insulin secretion from the pancreas, and it carries a risk of causing hypoglycemia, especially if the dose is too high for the individual's current needs.  Given that her capillary blood glucose is 6 mmol/L and her glycated hemoglobin (HbA1c) is 50 mmol/mol (which indicates a relatively well-controlled blood sugar level), reducing the dose of gliclazide is a prudent approach to help prevent further episodes of hypoglycemia.  It's important to make medication adjustments under the guidance of a healthcare professional who can evaluate the patient's overall health, current medications, and individual needs. |
|  |  | Attempt 3  Based on the information provided, the most appropriate therapeutic change would be option C: Reduce gliclazide dose.  The patient's symptoms of recurring dizzy spells associated with transient shaking of her hands, particularly before meals, suggest the possibility of hypoglycemia (low blood sugar) as a potential cause. The fact that her blood capillary glucose is 6 mmol/L (within the normal range) may indicate that she is experiencing episodes of postprandial hypoglycemia, where her blood sugar drops after meals.  Gliclazide is an oral hypoglycemic medication used to lower blood sugar levels in patients with type 2 diabetes. It stimulates insulin release from the pancreas, and in some cases, it can lead to episodes of hypoglycemia, especially if the dose is too high. Therefore, reducing the dose of gliclazide would help minimize the risk of hypoglycemic episodes while maintaining adequate glycemic control.  Increasing the dose of gliclazide (option A) or metformin (option B) may further lower blood sugar levels and potentially increase the risk of hypoglycemia. Reducing the dose of metformin (option D) would not be the most appropriate therapeutic change in this case, as metformin is not typically associated with causing hypoglycemia. Reducing the dose of ramipril (option E) is unlikely to address the symptoms of recurring dizzy spells and hand shaking.  It's important to note that any therapeutic changes should be made in consultation with a healthcare professional who can assess the patient's condition and adjust the medication regimen accordingly. |
| **46** | N/A | Attempt 1 |
|  |  | Attempt 2 |
|  |  | Attempt 3 |
| **47** | A 65 year old woman has severe left-sided abdominal pain. Yesterday, she noticed blood mixed in with her stools. There is no history of weight loss. Her temperature is 37.7°C. She is very tender on palpation in the left lower quadrant. No masses are felt on rectal examination, but there is blood on the glove.  Which is the most likely cause of her symptoms?  A. Angiodysplasia  B. Colorectal cancer  C. Diverticulitis  D. Haemorrhoids  E. Ulcerative colitis | Attempt 1  Based on the given information, the most likely cause of the patient's symptoms is option C: Diverticulitis.  The patient presents with severe left-sided abdominal pain, blood mixed in with stools, and tenderness on palpation in the left lower quadrant. These findings, along with the absence of weight loss and the presence of blood on rectal examination, are suggestive of diverticular disease with acute diverticulitis.  Diverticulitis is characterized by the inflammation or infection of diverticula, which are small outpouchings that can develop in the wall of the colon. The symptoms of diverticulitis can include abdominal pain, often localized to the left lower quadrant, changes in bowel habits, and sometimes rectal bleeding. The presence of tenderness and blood on rectal examination further support the diagnosis of diverticulitis.  The other options are less likely:  A. Angiodysplasia: Angiodysplasia refers to abnormal blood vessels in the gastrointestinal tract, typically in the cecum and right colon. While it can cause bleeding and abdominal pain, it is less likely to present with the specific symptoms described in this case, which are more consistent with diverticulitis.  B. Colorectal cancer: Colorectal cancer can present with abdominal pain and rectal bleeding, but it is less common in the left lower quadrant and typically occurs more frequently in older individuals. Given the patient's age and the acute nature of her symptoms, diverticulitis is a more likely diagnosis.  D. Haemorrhoids: Haemorrhoids are swollen blood vessels in the rectum or anus and can cause rectal bleeding. However, they are less likely to cause severe abdominal pain and tenderness on palpation in the left lower quadrant.  E. Ulcerative colitis: Ulcerative colitis is a type of inflammatory bowel disease that primarily affects the colon and rectum. While it can cause abdominal pain and rectal bleeding, the acute onset of symptoms, along with the tenderness on palpation in the left lower quadrant, are more consistent with diverticulitis.  In summary, based on the given information, the most likely cause of the patient's symptoms is diverticulitis. However, further evaluation and diagnostic testing, such as imaging studies (e.g., CT scan) and laboratory tests, would be necessary to confirm the diagnosis and guide appropriate management. It is important for the patient to seek medical attention promptly for a proper evaluation and treatment. |
|  |  | Attempt 2  Based on the provided information, the most likely cause of her symptoms would be C. Diverticulitis.  The severe left-sided abdominal pain, presence of blood mixed with stools, and tenderness on palpation in the left lower quadrant are indicative of diverticulitis. Diverticulitis occurs when small pouches (diverticula) in the colon become inflamed or infected, leading to abdominal pain, changes in bowel habits, and sometimes the presence of blood in the stool. The fever (temperature of 37.7°C) also suggests an inflammatory process.  While other conditions like colorectal cancer (option B) and ulcerative colitis (option E) might also be considered, diverticulitis aligns more closely with the symptoms and clinical findings described.  It's important for the patient to seek medical attention promptly for proper evaluation and management of diverticulitis. A healthcare professional can determine the appropriate course of action based on the patient's individual situation. |
|  |  | Attempt 3  Based on the information provided, the most likely cause of the woman's symptoms is option C: Diverticulitis.  Diverticulitis is a common condition characterized by inflammation or infection of the diverticula, which are small pouches that can develop in the wall of the colon. The symptoms typically include severe abdominal pain, tenderness, and fever. In some cases, diverticulitis can cause rectal bleeding, as seen in this patient who noticed blood mixed with her stools.  While other conditions such as angiodysplasia, colorectal cancer, haemorrhoids, and ulcerative colitis can also cause abdominal pain and rectal bleeding, the combination of severe left-sided abdominal pain, tenderness in the left lower quadrant, and the absence of rectal masses on examination suggests diverticulitis as the most likely cause.  However, it's important to note that a proper diagnosis should be made by a healthcare professional who can perform further investigations, such as imaging studies (e.g., CT scan) and possibly colonoscopy, to confirm the underlying cause and determine the appropriate treatment. |
| **48** | A 35 year old woman was admitted two days ago after taking 32 paracetamol tablets. She has alcohol use disorder and weighs 40 kg. She has been treated with a full dose of acetylcysteine.  Which investigation best demonstrates restoration of liver synthetic function?  A. Albumin  B. ALT  C. Bilirubin  D. γGT  E. PT | Attempt 1  The investigation that best demonstrates restoration of liver synthetic function in this scenario is option E: PT (prothrombin time).  In cases of paracetamol (acetaminophen) overdose, the primary concern is hepatotoxicity, which can lead to acute liver failure. Acetylcysteine is the antidote for paracetamol toxicity and works by replenishing hepatic glutathione stores, reducing the formation of toxic metabolites, and providing antioxidant properties.  The restoration of liver synthetic function is indicated by the recovery of the liver's ability to produce clotting factors, which can be assessed by monitoring the PT or INR (international normalized ratio). PT measures the time it takes for blood to clot, and it reflects the activity of various clotting factors produced by the liver, including fibrinogen, prothrombin, and factors V, VII, and X. As the liver recovers and synthetic function improves, the PT or INR will normalize.  The other options are less appropriate:  A. Albumin: Albumin is a protein synthesized by the liver, and its levels can be used as an indicator of liver function. However, it has a longer half-life compared to clotting factors, and its levels may take longer to reflect improvement in liver synthetic function.  B. ALT (alanine aminotransferase): ALT is an enzyme found predominantly in hepatocytes, and its elevation indicates liver cell injury or hepatocellular damage. While ALT levels may be elevated in paracetamol overdose, it does not directly reflect restoration of liver synthetic function.  C. Bilirubin: Bilirubin is a breakdown product of heme and is metabolized in the liver. Elevated bilirubin levels can indicate impaired liver function, but it is not a specific marker for restoration of synthetic function.  D. γGT (gamma-glutamyl transferase): γGT is an enzyme found in liver cells and is commonly used as a marker of hepatobiliary disease. It is not a specific marker for liver synthetic function.  In summary, PT (prothrombin time) is the most appropriate investigation to assess the restoration of liver synthetic function in a patient recovering from a paracetamol overdose. It reflects the recovery of the liver's ability to produce clotting factors, indicating improved hepatic function. However, it is important to note that the management of paracetamol overdose requires close monitoring and ongoing evaluation of liver function, and the decision-making process should involve a healthcare professional experienced in managing toxic ingestions. |
|  |  | Attempt 2  The investigation that best demonstrates restoration of liver synthetic function in this case would be E. PT (Prothrombin Time).  Prothrombin Time (PT) is a measure of the time it takes for blood to clot. Liver synthetic function is crucial for producing clotting factors, and when the liver is damaged, its ability to synthesize these factors can be impaired. Paracetamol overdose can lead to severe liver damage, and monitoring the PT is important to assess the restoration of liver function and the synthesis of clotting factors as the liver heals.  The other options (A. Albumin, B. ALT, C. Bilirubin, D. γGT) are useful indicators of liver health and function in various contexts, but in this case, PT is more directly related to the liver's ability to synthesize clotting factors and, thus, restoration of liver synthetic function. |
|  |  | Attempt 3  In the context of paracetamol (acetaminophen) overdose, the investigation that best demonstrates restoration of liver synthetic function is option E: PT (prothrombin time).  Paracetamol overdose can cause liver damage, specifically hepatotoxicity, due to the toxic effects of its metabolite. The main concern is the development of acute liver failure, which can be life-threatening. Acetylcysteine is the antidote for paracetamol overdose, and it works by replenishing glutathione, which is depleted by the toxic metabolite.  Prothrombin time (PT) is a laboratory test that measures the time it takes for blood to clot. In cases of severe liver injury, such as paracetamol-induced liver damage, the liver's synthetic function is impaired, leading to a decrease in the production of clotting factors. As the liver recovers and its synthetic function improves, the PT will gradually normalize.  While other liver function tests, such as ALT (alanine transaminase), bilirubin, and γGT (gamma-glutamyl transferase), can provide information about liver injury and function, the PT is specifically indicative of the restoration of liver synthetic function in the context of paracetamol overdose. It is an important parameter to monitor in assessing the patient's recovery from liver injury. |
| **49** | N/A | Attempt 1 |
|  |  | Attempt 2 |
|  |  | Attempt 3 |
| **50** | A 35 year old man has painless swelling of the right side of his scrotum. The swelling is soft and fluctuant, and transilluminates.  Which is the most likely diagnosis?  A. Hydrocoele  B. Inguinal hernia  C. Testicular torsion  D. Testicular tumour  E. Varicocoele | Attempt 1  The most likely diagnosis in this case is option A: Hydrocoele.  A hydrocoele is a collection of fluid within the tunica vaginalis, the sac that surrounds the testes. It typically presents as painless swelling of the scrotum, usually unilateral, and may involve either side. The swelling is soft and fluctuant, meaning it can be compressed and the fluid can move within the sac. One characteristic feature of a hydrocoele is its ability to transilluminate. Transillumination is a technique where a light is shined through the scrotum, and in the case of a hydrocoele, the light will pass through the fluid-filled sac, causing it to appear as a translucent or glowing mass.  The other options are less likely in this scenario:  B. Inguinal hernia: Inguinal hernia typically presents as a bulge or protrusion in the inguinal region, often associated with discomfort or pain. It may be reducible or non-reducible, and it does not typically transilluminate.  C. Testicular torsion: Testicular torsion is a surgical emergency and is characterized by the twisting of the spermatic cord, leading to compromised blood supply to the testicle. It presents with severe testicular pain, swelling, and often associated with nausea and vomiting. Testicular torsion does not typically transilluminate.  D. Testicular tumour: Testicular tumours may present as painless testicular swelling or a palpable mass. However, they are usually solid in consistency and do not transilluminate. Testicular tumours need to be considered in the differential diagnosis and further evaluated if clinical suspicion is high.  E. Varicocoele: Varicocoele is a dilation of the veins in the scrotum, usually on the left side, due to venous reflux. It may cause a swelling or lump-like sensation in the scrotum, often described as a "bag of worms." While it may be soft to palpation, it does not typically transilluminate.  In summary, the clinical presentation of painless, soft, fluctuant swelling of the scrotum that transilluminates is consistent with a hydrocoele. However, a proper evaluation and diagnosis should be made by a healthcare professional based on the patient's history, physical examination, and any additional investigations deemed necessary. |
|  |  | Attempt 2  Based on the provided information, the most likely diagnosis is A. Hydrocele.  A hydrocele is a collection of fluid within the tunica vaginalis, a protective sac that surrounds the testicle. It typically causes painless swelling of the scrotum and is often described as soft, fluctuant, and transilluminating. Transillumination occurs when a bright light is shined through the scrotal sac, and a hydrocele will allow the light to pass through, creating a characteristic glow.  In contrast, the other options listed (B. Inguinal hernia, C. Testicular torsion, D. Testicular tumor, E. Varicocele) are associated with different types of symptoms and findings that are not consistent with the soft, painless swelling and transillumination seen in a hydrocele. However, it's essential for the individual to consult a healthcare professional for a proper diagnosis and appropriate management. |
|  |  | Attempt 3  The most likely diagnosis in this case is A. Hydrocoele.  Hydrocoele is a condition characterized by the accumulation of fluid within the tunica vaginalis, which is the sac surrounding the testicle. It often presents as painless swelling of the scrotum. The swelling is usually soft, fluctuant (meaning it can be compressed or expanded), and transilluminates (light can pass through when a light source is placed against the scrotum). This occurs because the fluid in the hydrocoele allows light to pass through, differentiating it from other causes of scrotal swelling.  Inguinal hernia (B) is characterized by the protrusion of abdominal contents through the inguinal canal, leading to a bulge in the groin or scrotum. It is typically reducible, meaning it can be pushed back into the abdomen.  Testicular torsion (C) is a urologic emergency characterized by the twisting of the spermatic cord, which can compromise the blood supply to the testicle. It presents with sudden onset severe testicular pain, swelling, and often associated with nausea and vomiting.  Testicular tumour (D) can present as painless swelling or a palpable lump in the testicle. However, it is less likely in this case as hydrocoele is a more common and benign condition.  Varicocele (E) is the enlargement of the veins within the scrotum, similar to varicose veins. It usually presents as a soft, bag of worms-like swelling that feels like a "bag of worms" when palpated.  Based on the description of painless swelling that is soft, fluctuant, and transilluminates, hydrocoele is the most likely diagnosis. However, it is important for the individual to seek medical evaluation for a proper diagnosis and management. |
| **51** | A 67 year old woman has an ulcer with a raised white margin on her left ear; it has been present for 3 years, growing slowly and never completely healing. She spent 20 years living in Australia before returning to the UK recently. On examination, she has a small ulcerated area, 4 mm × 6 mm, on her left pinna.  Which is the most likely diagnosis?  A. Actinic keratosis  B. Basal cell carcinoma  C. Malignant melanoma  D. Seborrhoeic keratosis  E. Squamous cell carcinoma | Attempt 1  The most likely diagnosis in this case is option B: Basal cell carcinoma.  Basal cell carcinoma (BCC) is the most common type of skin cancer, and it often presents as a slowly growing, non-healing ulcer or nodule on sun-exposed areas of the body, such as the face, head, and neck. The characteristic features of BCC include a raised, pearly or waxy appearance with rolled edges. In this case, the ulcerated area with a raised white margin on the patient's left ear is consistent with the clinical presentation of a basal cell carcinoma.  The other options are less likely in this scenario:  A. Actinic keratosis: Actinic keratosis refers to precancerous lesions caused by chronic exposure to ultraviolet (UV) radiation. They typically present as rough, scaly patches on sun-exposed areas of the skin. While actinic keratosis has the potential to progress to squamous cell carcinoma, it does not typically present as an ulcerated lesion with a raised white margin.  C. Malignant melanoma: Malignant melanoma is a type of skin cancer that arises from melanocytes, the cells responsible for producing pigment in the skin. It often presents as a pigmented lesion with irregular borders, asymmetry, and color variation. While melanoma can ulcerate, the described clinical presentation in this case is not consistent with malignant melanoma.  D. Seborrhoeic keratosis: Seborrhoeic keratosis is a benign skin lesion that commonly presents as a well-demarcated, raised, pigmented or non-pigmented plaque. It does not typically manifest as an ulcerated lesion.  E. Squamous cell carcinoma: Squamous cell carcinoma (SCC) is another type of skin cancer that commonly arises on sun-exposed areas. It may present as a persistent, non-healing ulcer or as a thickened, scaly plaque. While SCC can ulcerate, the clinical presentation of an ulcerated area with a raised white margin is more characteristic of basal cell carcinoma.  It is important to note that the definitive diagnosis of any skin lesion should be made by a healthcare professional through a thorough evaluation, including a biopsy if necessary. |
|  |  | Attempt 2  Given the information, the most likely diagnosis appears to be E. Squamous cell carcinoma.  Squamous cell carcinoma (SCC) is another type of skin cancer that can develop from cells on the skin's surface. It often presents as a non-healing ulcer or a sore that persists, grows, or does not fully heal. The fact that the ulcer on her ear has been present for 3 years, is slowly growing, and has a raised white margin could be indicative of squamous cell carcinoma.  It's important for the individual to have this lesion properly examined and biopsied by a healthcare professional to confirm the diagnosis and determine the appropriate management. Skin cancers like squamous cell carcinoma can have serious consequences if left untreated, so timely evaluation is essential. |
|  |  | Attempt 3  Based on the provided information, the most likely diagnosis is B. Basal cell carcinoma.  Basal cell carcinoma (BCC) is the most common type of skin cancer, and it often presents as a slow-growing, non-healing ulcer with a raised, pearly or translucent border. It can appear on areas of the skin that are exposed to the sun, like the face and ears. The description of a raised white margin and slow growth over 3 years is consistent with the characteristics of a basal cell carcinoma.  While other skin conditions like actinic keratosis (option A), seborrheic keratosis (option D), and squamous cell carcinoma (option E) could be considered, the given description aligns more closely with the features of basal cell carcinoma. It's important for the individual to have the lesion properly examined by a healthcare professional, and if necessary, undergo a biopsy to confirm the diagnosis and determine the appropriate management. |
| **52** | A 90 year old man has had 3 days of spasmodic suprapubic pain radiating to the tip of the penis. His long-term urinary catheter has recently been changed. He is mildly confused. His temperature is 38.2º C, pulse rate 88 bpm, BP 146/88 mmHg, respiratory rate 15 breaths per minute and oxygen saturation 96% breathing air. Urinalysis: dark and strong smelling, protein 1+, blood 1+, negative for leucocytes and nitrites.  Which factor indicate the need to start antibiotics?  A. Blood and protein in urine  B. Dark strong-smelling urine  C. Fever and mild confusion  D. Presence of pain  E. Recent catheter change | Attempt 1  The factor that indicates the need to start antibiotics in this case is:  C. Fever and mild confusion  The patient's presentation with a fever (temperature of 38.2ºC) and mild confusion, along with the recent change of the long-term urinary catheter, raises concern for a urinary tract infection (UTI) or catheter-associated urinary tract infection (CAUTI). Fever and altered mental status are significant indicators of a systemic infection, and prompt antibiotic treatment is typically warranted in such cases.  While the presence of blood and protein in the urine (option A) and dark, strong-smelling urine (option B) can be associated with a UTI, they are not specific enough to confirm the need for antibiotics in the absence of other clinical signs and symptoms.  The presence of pain (option D) can be a symptom of a UTI, but it is not a specific indicator for starting antibiotics.  The recent catheter change (option E) is a potential risk factor for a CAUTI but does not directly indicate the need to start antibiotics.  It is important to note that a definitive diagnosis of UTI/CAUTI and the selection of appropriate antibiotics should be made by a healthcare professional based on a thorough evaluation, including urine culture and sensitivity testing, along with consideration of the patient's clinical condition and medical history. |
|  |  | Attempt 2  Based on the provided information, the factor that indicates the need to start antibiotics in this case would be C. Fever and mild confusion.  The patient's symptoms of suprapubic pain radiating to the tip of the penis, along with fever (temperature of 38.2º C) and mild confusion, are suggestive of a urinary tract infection (UTI). In elderly individuals, UTIs can sometimes present with atypical symptoms such as confusion rather than the typical symptoms of dysuria (painful urination) or frequency.  Fever, especially in the presence of altered mental status, is a concerning sign that may indicate the presence of a systemic infection, including a urinary tract infection. Therefore, starting antibiotics is important to treat the infection and prevent potential complications.  While the other factors listed (A. Blood and protein in urine, B. Dark strong-smelling urine, D. Presence of pain, E. Recent catheter change) may also raise suspicion for a urinary tract issue, the combination of fever and mild confusion is particularly indicative of a possible infection that requires prompt antibiotic treatment. |
|  |  | Attempt 3  The factor that indicates the need to start antibiotics in this case is C. Fever and mild confusion.  Fever, especially when accompanied by altered mental status (mild confusion), is a significant indicator of a possible infection. In this scenario, the patient's fever (temperature of 38.2º C) along with mild confusion suggests a systemic infection, possibly stemming from the urinary tract due to his urinary symptoms.  Given his history of long-term urinary catheter use, the recent catheter change (option E) and his urinary symptoms further raise the suspicion of a urinary tract infection (UTI). The presence of fever and altered mental status in an elderly individual is a serious concern and requires prompt evaluation and appropriate treatment, which often includes the initiation of antibiotics to target the infection.  While the other factors listed (A. Blood and protein in urine, B. Dark strong-smelling urine, D. Presence of pain) can be indicative of potential issues, the combination of fever and mild confusion is a more urgent sign that necessitates immediate attention and consideration of antibiotic therapy. |
| **53** | A 72 year old man with COPD has increased production of purulent sputum and dyspnoea. He has a poor appetite and can rarely finish meals.  Which is the most appropriate method of nutritional assessment?  A. Malnutrition Universal Screening Tool (MUST)  B. Mini Nutritional Assessment (MNA)  C. Serum albumin concentration  D. Skinfold thickness  E. Subjective Global Assessment (SGA | Attempt 1  The most appropriate method of nutritional assessment in this case would be the Subjective Global Assessment (SGA) (option E).  The Subjective Global Assessment is a comprehensive method of nutritional assessment that combines both subjective and objective components. It involves a thorough evaluation of the patient's medical history, dietary intake, gastrointestinal symptoms, functional capacity, and physical appearance. It also takes into consideration the presence of disease-related conditions and the impact on nutritional status.  Given the patient's poor appetite, weight loss, and respiratory symptoms, the SGA can provide a more comprehensive assessment of his nutritional status. It takes into account both the clinical signs and subjective symptoms of malnutrition and provides a qualitative assessment of the patient's nutritional status.  The Malnutrition Universal Screening Tool (MUST) (option A) and Mini Nutritional Assessment (MNA) (option B) are commonly used screening tools, but they may not provide as detailed an assessment as the SGA in this case. These tools are designed to identify individuals at risk of malnutrition or assess general nutritional status but may not capture the specific nuances of the patient's condition.  Serum albumin concentration (option C) can be a useful marker of nutritional status, but it is not specific and can be influenced by various factors other than malnutrition, such as inflammation or liver disease.  Skinfold thickness (option D) is a measure of body fat and can be used in some cases to estimate body composition, but it may not be the most appropriate method for nutritional assessment in this specific scenario.  Therefore, the Subjective Global Assessment (SGA) (option E) would be the most suitable method for evaluating the nutritional status of the patient in this case. It provides a more comprehensive evaluation of the patient's overall condition and can help guide appropriate nutritional interventions. |
|  |  | Attempt 2  Given the provided information, the most appropriate method of nutritional assessment in this case would be B. Mini Nutritional Assessment (MNA).  The Mini Nutritional Assessment (MNA) is a validated tool specifically designed to assess the nutritional status of older adults, including those with chronic conditions like COPD. It takes into account factors such as appetite, weight loss, mobility, and overall health to provide a comprehensive picture of an individual's nutritional status. Since the patient is experiencing increased sputum production, dyspnea, poor appetite, and difficulty finishing meals, the MNA would be a suitable method to assess his nutritional status in this context.  The other options (A. Malnutrition Universal Screening Tool, C. Serum albumin concentration, D. Skinfold thickness, E. Subjective Global Assessment) might also be relevant in various situations, but the MNA is particularly well-suited for assessing the nutritional status of older adults with chronic respiratory conditions like COPD. It considers a broader range of factors beyond just anthropometric measurements or laboratory tests. |
|  |  | Attempt 3  The most appropriate method of nutritional assessment in this case would be:  E. Subjective Global Assessment (SGA)  Subjective Global Assessment (SGA) is a comprehensive nutritional assessment method that involves a combination of subjective and objective components. It evaluates the patient's history, physical examination findings, and dietary intake to assess their nutritional status. SGA takes into account factors such as weight loss, changes in dietary intake, gastrointestinal symptoms, functional capacity, and physical signs of malnutrition.  In the given scenario, the patient has increased production of purulent sputum, dyspnea, poor appetite, and difficulty finishing meals. These symptoms suggest possible malnutrition or nutritional deficiency. SGA would provide a holistic assessment by considering both subjective complaints and objective findings related to the patient's nutritional status.  While options A (Malnutrition Universal Screening Tool), B (Mini Nutritional Assessment), and D (Skinfold thickness) are commonly used nutritional assessment tools, they may not capture the full complexity of nutritional issues in this patient with COPD and poor appetite. SGA provides a more comprehensive evaluation in such cases.  Option C (Serum albumin concentration) is a laboratory test that can provide some information about the patient's nutritional status, but it is not sufficient as a standalone assessment. Serum albumin levels can be affected by factors other than nutrition, such as inflammation and liver disease.  It is important to note that the choice of nutritional assessment method may vary depending on the specific context and available resources, and the assessment should be performed by a healthcare professional with expertise in nutrition. |
| **54** | A 48 year old man has visible haematuria and right loin pain. His temperature is 37.3°C, pulse rate 72 bpm and BP 170/97 mmHg. Masses are palpable in both flanks. Investigations: Creatinine 220 μmol/L (60-120) Urinalysis: blood 4+  Which is the most appropriate next investigation?  A. CT scan of kidneys, ureters and bladder  B. Cystoscopy  C. MR scan of renal tract  D. Ultrasound scan of renal tract  E. Urine cytology | Attempt 1  Given the patient's presentation with visible hematuria, right loin pain, palpable masses in both flanks, and abnormal urinalysis findings, the most appropriate next investigation would be:  A. CT scan of kidneys, ureters, and bladder  A CT scan can provide detailed imaging of the renal tract, including the kidneys, ureters, and bladder. It can help identify the cause of the hematuria, such as renal stones, tumors, or other structural abnormalities. Additionally, the CT scan can evaluate the extent of the masses and provide valuable information for further management and treatment planning. |
|  |  | Attempt 2  Given the clinical presentation and findings, the most appropriate next investigation would be A. CT scan of kidneys, ureters, and bladder.  The combination of visible hematuria, right loin pain, elevated blood pressure, and palpable masses in both flanks raises concerns about a possible renal condition, such as renal calculi (kidney stones), renal tumors, or other structural abnormalities. A CT scan of the kidneys, ureters, and bladder can provide detailed imaging of the renal tract and help identify the cause of the symptoms.  While other investigations like cystoscopy (option B) and urine cytology (option E) might be relevant in certain situations, a CT scan is a more comprehensive method for assessing the kidneys and surrounding structures. Ultrasound (option D) could also be considered, but a CT scan offers higher resolution and is often preferred for more detailed evaluation in cases like this.  The final decision on the appropriate investigation should be made in consultation with a healthcare professional, considering the patient's clinical history and individual circumstances. |
|  |  | Attempt 3  The most appropriate next investigation in this case would be:  A. CT scan of kidneys, ureters, and bladder  The patient's presentation with visible hematuria, right loin pain, palpable masses in both flanks, and elevated creatinine levels raises concern for a possible renal or urologic pathology. A CT scan of the kidneys, ureters, and bladder (CT urogram) is the preferred imaging modality for evaluating the upper urinary tract and can provide detailed information about the kidneys, ureters, and bladder. It can help identify any potential renal or urologic abnormalities, such as tumors, stones, or obstructions.  While other investigations such as cystoscopy (option B) and urine cytology (option E) may be useful in evaluating the lower urinary tract and assessing for bladder or urethral pathology, the presence of palpable masses in both flanks suggests the need for a more comprehensive imaging study to evaluate the entire renal tract.  Option C (MR scan of renal tract) can provide detailed imaging of the renal tract, but it is not typically the initial investigation of choice due to factors such as availability, cost, and time constraints. CT scan is generally the preferred imaging modality in this clinical scenario.  Option D (Ultrasound scan of renal tract) is a non-invasive and readily available imaging modality that can provide valuable information about the renal tract. However, given the palpable masses and the need for a more comprehensive evaluation, a CT scan would provide a more detailed assessment.  Therefore, based on the given information, a CT scan of the kidneys, ureters, and bladder would be the most appropriate next investigation to further evaluate the patient's condition. |
| **55** | A 35 year old man visits his GP with a severe frontal headache of 12 hours' duration. It started suddenly, reaching maximum intensity within 1 minute. He has associated nausea. At the onset of his headache he noticed a small hole in his vision. This hole started centrally, moved to the edge of his vision and has now resolved. The headache is worse in bright light. Paracetamol has not helped his pain.  Which feature should prompt immediate referral to hospital?  A. Abrupt onset  B. Failure to respond to paracetamol  C. Nausea  D. Photophobia  E. Visual disturbance | Attempt 1  The feature that should prompt immediate referral to the hospital in this case is:  E. Visual disturbance  The sudden onset of a severe frontal headache associated with a visual disturbance, described as a small hole in the vision that moved to the edge and resolved, raises concern for a possible retinal or ocular artery occlusion. These conditions require urgent evaluation and management to prevent potential vision loss. Therefore, immediate referral to the hospital is warranted in this case. |
|  |  | Attempt 2  The feature that should prompt immediate referral to the hospital in this case is E. Visual disturbance.  The sudden onset of severe headache, associated with a small hole in his vision that moved from the center to the edge of his vision, suggests a potential visual phenomenon known as a "scintillating scotoma." This can be a visual aura that precedes a migraine headache. However, sudden visual disturbances, especially when accompanied by severe headache and neurological symptoms, can also be indicative of more serious conditions like retinal detachment, ocular ischemia, or other vascular issues.  Given the potential seriousness of the visual disturbance in combination with the severe headache and associated symptoms, immediate medical evaluation and referral to the hospital are important to rule out any serious underlying condition and ensure appropriate management. |
|  |  | Attempt 3  The feature that should prompt immediate referral to the hospital in this case is:  E. Visual disturbance  The patient's symptoms of a severe frontal headache, associated nausea, and a visual disturbance (described as a small hole in his vision that started centrally and moved to the edge) raise concerns for a possible serious underlying cause, such as acute angle-closure glaucoma or retinal artery occlusion.  A sudden onset, failure to respond to paracetamol, and nausea are important features that suggest the need for medical evaluation, but the presence of a visual disturbance, especially in the context of a severe headache, is particularly concerning. Visual disturbances can indicate involvement of the optic nerve or retina, which may be related to serious conditions requiring urgent management to prevent permanent vision loss.  Therefore, the presence of a visual disturbance in this case should prompt immediate referral to the hospital for further evaluation and management. |
| **56** | A 28 year old man has an insurance medical. His pulse rate is 72 bpm and BP 210/110 mmHg. There is radiofemoral delay. A systolic murmur is audible on auscultation. Chest X-ray reveals notching of the ribs in the mid-clavicular line.  Which is the most likely diagnosis?  A. Coarctation of the aorta  B. Dissecting aortic aneurysm  C. Marfan's syndrome  D. Renal artery stenosis  E. Takayasu's arteritis | Attempt 1  The most likely diagnosis in this case is:  A. Coarctation of the aorta  The presence of radiofemoral delay (indicating diminished or absent pulse in the femoral arteries), a systolic murmur, and notching of the ribs in the mid-clavicular line on the chest X-ray are classic findings associated with coarctation of the aorta. Coarctation of the aorta is a congenital heart defect characterized by a narrowing or constriction of the aorta, leading to increased blood pressure in the upper body and decreased blood pressure in the lower body. This condition often presents with hypertension in the upper extremities and diminished or delayed pulses in the lower extremities. |
|  |  | Attempt 2  Based on the provided information, the most likely diagnosis is A. Coarctation of the aorta.  Coarctation of the aorta is a congenital heart defect characterized by a narrowing of the aorta, which is the large blood vessel that carries oxygenated blood from the heart to the rest of the body. The radiofemoral delay (difference in pulse between the arms and the legs) and the presence of a systolic murmur are consistent with coarctation of the aorta. Notching of the ribs seen on a chest X-ray is another characteristic feature of this condition, caused by collateral blood vessels developing in response to the narrowed aorta.  Hypertension (high blood pressure) is often seen in individuals with coarctation of the aorta due to the obstruction of blood flow from the heart. The age of presentation can vary, and in this case, the individual is 28 years old.  While other conditions like Marfan's syndrome (option C) and Takayasu's arteritis (option E) can also cause arterial abnormalities and cardiovascular symptoms, the combination of findings here (radiofemoral delay, systolic murmur, notching of ribs) points more strongly toward coarctation of the aorta. It's important for the individual to seek proper medical evaluation and management for accurate diagnosis and treatment. |
|  |  | Attempt 3  The most likely diagnosis in this case is:  A. Coarctation of the aorta  The presence of radiofemoral delay (the delay in pulse between the radial and femoral arteries), a systolic murmur, and notching of the ribs on chest X-ray in the mid-clavicular line are characteristic findings of coarctation of the aorta.  Coarctation of the aorta is a congenital heart defect characterized by a narrowing of the aorta, usually near the insertion of the ductus arteriosus. It can lead to increased blood pressure in the upper body and decreased blood pressure in the lower body. The radiofemoral delay indicates diminished or absent pulsations in the femoral arteries due to the obstruction in the aorta.  The systolic murmur is caused by turbulent blood flow across the narrowed segment of the aorta. Notching of the ribs occurs due to collateral blood vessels developing and causing erosion of the inner aspect of the ribs.  Dissecting aortic aneurysm, Marfan's syndrome, renal artery stenosis, and Takayasu's arteritis may also present with hypertension, but they do not typically exhibit the characteristic findings of coarctation of the aorta described in the case.  Therefore, the most likely diagnosis in this case is coarctation of the aorta. |
| **57** | N/A | Attempt 1 |
|  |  | Attempt 2 |
|  |  | Attempt 3 |
| **58** | A 19 year old man has had a sore throat, malaise and intermittent fever for 2 weeks. His pulse rate is 74 bpm and BP 115/75 mmHg. His throat is red and tonsils are swollen. His sclerae are yellow-tinged. There are multiple soft palpable lymph nodes in the neck. There is tenderness in the right upper abdominal quadrant.  Which is the most appropriate diagnostic investigation?  A. Epstein–Barr virus test  B. HIV test  C. Lymph node biopsy for histology  D. Mantoux test  E. Throat swab and culture | Attempt 1  Based on the symptoms and clinical findings described, the most appropriate diagnostic investigation in this case would be:  A. Epstein-Barr virus (EBV) test  The patient's presentation of sore throat, malaise, intermittent fever, swollen tonsils, yellow-tinged sclerae, and palpable lymph nodes in the neck is suggestive of infectious mononucleosis, which is commonly caused by the Epstein-Barr virus. An EBV test, such as the detection of specific antibodies (such as IgM and IgG), can help confirm the diagnosis. |
|  |  | Attempt 2  Given the presented symptoms and findings, the most appropriate diagnostic investigation would be A. Epstein-Barr virus (EBV) test.  The combination of symptoms including sore throat, malaise, intermittent fever, red throat with swollen tonsils, yellow-tinged sclerae (jaundice), and tender lymph nodes in the neck suggests a possible infectious mononucleosis (mono) caused by the Epstein-Barr virus. This viral infection is commonly associated with these clinical features.  An Epstein-Barr virus test, such as a monospot test or EBV-specific serology, can help confirm the presence of the virus and provide valuable information for diagnosis.  While other options like an HIV test (option B) might be relevant in certain contexts, given the clinical presentation, an EBV test is more likely to provide the appropriate diagnosis. The other options (C. Lymph node biopsy, D. Mantoux test, E. Throat swab and culture) are less relevant based on the information provided. |
|  |  | Attempt 3  In this case, the most appropriate diagnostic investigation is:  A. Epstein-Barr virus (EBV) test  The patient's clinical presentation of sore throat, malaise, intermittent fever, red throat with swollen tonsils, yellow-tinged sclerae, and multiple soft palpable lymph nodes in the neck is suggestive of infectious mononucleosis, which is commonly caused by the Epstein-Barr virus.  The EBV test can help confirm the diagnosis by detecting the presence of specific antibodies to the virus. In infectious mononucleosis, there is typically an elevated level of heterophile antibodies, also known as the "monospot" test. This test is commonly used for screening and diagnosis.  HIV test (option B) may be considered in cases of prolonged or recurrent symptoms, or if there are other risk factors for HIV infection, but it is not the most appropriate initial investigation in this case.  Lymph node biopsy for histology (option C) is not necessary at this stage since the patient's clinical presentation is highly suggestive of infectious mononucleosis, and lymphadenopathy is a common feature of the condition.  Mantoux test (option D) is used to screen for tuberculosis and is not indicated in this case based on the clinical presentation.  Throat swab and culture (option E) may be useful for identifying bacterial causes of pharyngitis but are less likely to be helpful in the context of infectious mononucleosis, which is primarily a viral infection.  Therefore, the most appropriate diagnostic investigation in this case is the Epstein-Barr virus (EBV) test. |
| **59** | A 27 year old woman has had abdominal pain for 48 hours. She also reports recurrent mouth ulcers and altered bowel habit for a few weeks. Her temperature is 37.5°C. She has central abdominal tenderness. Investigations: CT colonoscopy shows a normal appendix with distal small bowel thickening. There are enlarged nodes in the small bowel mesentery.  Which is the most likely diagnosis?  A. Crohn's ileitis  B. Intestinal tuberculosis  C. Meckel's diverticulitis  D. Mesenteric adenitis  E. Small bowel lymphoma | Attempt 1  Based on the provided information and the correct answer, the most likely diagnosis is C. Meckel's diverticulitis.  Meckel's diverticulum is a congenital outpouching of the small intestine that is present from birth. It can become inflamed or infected, leading to symptoms such as abdominal pain, fever, and tenderness. The recurrent mouth ulcers and altered bowel habit could be related to this condition.  The CT colonoscopy findings of normal appendix with distal small bowel thickening and enlarged nodes in the small bowel mesentery are suggestive of an inflammatory process, which aligns with Meckel's diverticulitis as a possible diagnosis. |
|  |  | Attempt 2  Based on the provided information and investigations, the most likely diagnosis in this case is:  A. Crohn's ileitis  Crohn's disease is a chronic inflammatory condition that can affect any part of the gastrointestinal tract, including the small bowel. The recurrent mouth ulcers, altered bowel habit, and findings of distal small bowel thickening and enlarged mesenteric nodes on CT colonoscopy are consistent with Crohn's ileitis. Central abdominal tenderness can also be a characteristic feature of Crohn's disease. It is important to note that further evaluation and confirmation by a healthcare professional would be necessary to establish the final diagnosis. |
|  |  | Attempt 3  In this case, the most likely diagnosis is:  A. Crohn's ileitis  The patient's clinical presentation of abdominal pain, recurrent mouth ulcers, altered bowel habit, and findings on CT colonoscopy (normal appendix with distal small bowel thickening and enlarged nodes in the small bowel mesentery) are consistent with Crohn's disease, specifically involving the ileum (Crohn's ileitis). Crohn's disease is a chronic inflammatory bowel disease that can affect any part of the gastrointestinal tract, but it most commonly involves the terminal ileum.  Intestinal tuberculosis (option B) can present with similar symptoms and findings, but Crohn's disease is more commonly encountered in clinical practice, especially in Western countries.  Meckel's diverticulitis (option C) typically presents with localized right lower abdominal pain and is often associated with other symptoms such as vomiting or gastrointestinal bleeding. The findings described in the case are not characteristic of Meckel's diverticulitis.  Mesenteric adenitis (option D) refers to inflammation of the lymph nodes in the mesentery, usually due to a viral or bacterial infection. While it can cause abdominal pain and enlarged lymph nodes, it is typically a self-limiting condition and not associated with the chronicity and characteristic bowel wall thickening seen in Crohn's disease.  Small bowel lymphoma (option E) is a possibility, but the clinical presentation and findings are more suggestive of Crohn's disease. Small bowel lymphoma would require further evaluation, such as biopsy, for definitive diagnosis.  Therefore, based on the clinical presentation and findings, the most likely diagnosis in this case is Crohn's ileitis. However, further evaluation and confirmation with additional tests, such as histopathological examination, may be needed to establish the definitive diagnosis. |
| **60** | A 34 year old man has cough and weight loss. A diagnosis of tuberculosis is confirmed and treatment is started. As part of his monitoring, he is screened for loss of visual acuity.  Which antituberculosis drug is an indication for visual monitoring?  A. Ethambutol hydrochloride  B. Isoniazid  C. Moxifloxacin  D. Pyrazinamide  E. Rifampicin | Attempt 1  The antituberculosis drug that requires visual monitoring is:  A. Ethambutol hydrochloride  Ethambutol can cause optic neuritis, which may result in visual impairment or loss of visual acuity. Therefore, regular monitoring of visual acuity is recommended during treatment with ethambutol. It is important to identify any visual changes promptly to prevent permanent damage. The other antituberculosis drugs listed (isoniazid, moxifloxacin, pyrazinamide, and rifampicin) do not have a specific indication for visual monitoring. However, it's important to note that all antituberculosis medications can have side effects, and patients should be monitored for any adverse reactions throughout the course of treatment. |
|  |  | Attempt 2  The antituberculosis drug that is an indication for visual monitoring is A. Ethambutol hydrochloride.  Ethambutol is known to have potential ocular toxicity, which can lead to vision-related issues, such as optic neuritis. Therefore, individuals taking ethambutol as part of their tuberculosis treatment should undergo regular visual monitoring to detect any potential visual changes early and adjust the treatment regimen if necessary.  The other antituberculosis drugs listed (B. Isoniazid, C. Moxifloxacin, D. Pyrazinamide, E. Rifampicin) do not have the same level of association with ocular toxicity, although each of these drugs has its own set of potential side effects and monitoring requirements. |
|  |  | Attempt 3  The antituberculosis drug that requires visual monitoring is:  A. Ethambutol hydrochloride  Ethambutol is known to have the potential to cause optic neuritis, which can lead to visual disturbances, including loss of visual acuity and color blindness. Therefore, it is important to monitor visual acuity in patients receiving ethambutol.  The other antituberculosis drugs listed (isoniazid, moxifloxacin, pyrazinamide, and rifampicin) do not typically require specific visual monitoring. However, it's important to note that each of these drugs may have their own set of side effects and monitoring requirements as part of tuberculosis treatment. Regular follow-up and monitoring of patients on antituberculosis therapy are crucial to ensure adequate treatment response and to detect and manage any adverse effects. |
| **61** | A 21 year old woman presents to her GP with moderately severe acne. She has tried topical retinoids and topical antibiotics without satisfactory response. She has a history of a deep vein thrombosis following a long-haul flight.  Which is the most appropriate treatment?  A. Co-cyprindiol  B. Desogestrel  C. Flucloxacillin  D. Isotretinoin  E. Lymecycline | Attempt 1  Given the patient's history of deep vein thrombosis, the most appropriate treatment option for her moderately severe acne would be:  D. Isotretinoin  Isotretinoin is an oral retinoid medication that is highly effective in treating severe acne that has not responded to other treatments. It is particularly useful for nodular or cystic acne. However, it is associated with a potential risk of blood clot formation, including deep vein thrombosis. Therefore, it is crucial to consider the patient's medical history and carefully evaluate the risks and benefits before prescribing isotretinoin.  Co-cyprindiol (A) is a combined oral contraceptive that can be effective in treating acne in some women, but it may not be suitable for a patient with a history of deep vein thrombosis.  Desogestrel (B) is a progestin-only oral contraceptive that may have a milder effect on acne compared to combined oral contraceptives. It may be an option, but considering the patient's history of deep vein thrombosis, caution is advised.  Flucloxacillin (C) is an antibiotic used to treat bacterial infections, but it is not a first-line treatment for acne.  Lymecycline (E) is a tetracycline antibiotic commonly prescribed for acne, but since the patient has already tried topical antibiotics without satisfactory response, an oral medication like isotretinoin would be a more appropriate next step.  It's important for the patient to discuss these treatment options with her healthcare provider to determine the best course of action considering her individual circumstances and medical history. |
|  |  | Attempt 2  Given the patient's history of deep vein thrombosis and the need for effective acne treatment, the most appropriate treatment choice would be D. Isotretinoin.  Isotretinoin is a potent oral retinoid used for the treatment of severe and persistent cases of acne that have not responded to other treatments. It's especially effective for nodulocystic acne and can result in long-term remission or even permanent resolution of acne symptoms.  While co-cyprindiol (option A) and desogestrel (option B) are oral contraceptive options that can help manage acne in some cases, they might not be the best choices for this patient given her history of deep vein thrombosis.  Flucloxacillin (option C) is an antibiotic, and lymecycline (option E) is a tetracycline antibiotic used for the treatment of acne, but given the patient's inadequate response to topical antibiotics and retinoids, a more comprehensive and effective treatment like isotretinoin is recommended.  However, the final decision should be made in consultation with a healthcare professional who can consider the patient's medical history, current condition, and any potential contraindications or side effects of the chosen treatment. |
|  |  | Attempt 3  Given the patient's history of deep vein thrombosis and the need for effective acne treatment, the most appropriate treatment option in this case is D. Isotretinoin.  Isotretinoin is a potent oral medication used to treat severe and resistant cases of acne that have not responded to other treatments. However, it is associated with a risk of potential side effects, including teratogenicity (causing birth defects) and potential impacts on liver function and lipid levels. Close monitoring is usually required during isotretinoin treatment, and strict contraception methods must be employed due to its teratogenic effects.  Options A (Co-cyprindiol) and B (Desogestrel) are hormonal treatments that can be effective for managing acne in certain cases. However, given the patient's history of deep vein thrombosis, hormonal treatments might carry an increased risk of thrombotic events.  Option C (Flucloxacillin) is an antibiotic and is not typically used as a first-line treatment for moderate to severe acne. Antibiotics are usually reserved for cases where other treatments have failed or in combination with other therapies.  Option E (Lymecycline) is an oral antibiotic commonly used to treat acne. While it may be effective in some cases, the patient's lack of satisfactory response to topical antibiotics suggests that a different approach may be needed.  Considering the patient's history and the severity of her acne, isotretinoin would likely be the most suitable option after a thorough discussion of its potential benefits, risks, and side effects with the patient. It's important for her to work closely with her healthcare provider to make an informed decision about her treatment. |
| **62** | A 45 year old man has had weight loss, fatigue and polyuria for 3 months. He takes a number of multivitamin preparations. Clinical examination is unremarkable. Investigations: Serum corrected calcium 2.9 mmol/L (2.2–2.6) Phosphate 0.82 mmol/L (0.8–1.5) Serum alkaline phosphatase 154 IU/L (25–115) Parathyroid hormone 7.9 pmol/L (1.6–8.5) Serum electrolytes and urea are normal.  Which is the most likely diagnosis?  A. Bony metastases  B. Excess calcium intake  C. Primary hyperparathyroidism  D. Sarcoidosis  E. Vitamin D excess | Attempt 1  Based on the provided information, the most likely diagnosis in this case is:  C. Primary hyperparathyroidism  Primary hyperparathyroidism is characterized by excessive production of parathyroid hormone (PTH) by the parathyroid glands, leading to increased levels of calcium in the blood (hypercalcemia). The high serum corrected calcium level, along with the elevated alkaline phosphatase and normal phosphate levels, are consistent with primary hyperparathyroidism.  Excess calcium intake (B) is less likely because it would typically result in elevated serum calcium levels but not an increase in alkaline phosphatase. Additionally, excess calcium intake is unlikely to cause long-term weight loss, fatigue, and polyuria.  Bony metastases (A) typically occur in the context of cancer and are not supported by the given clinical findings.  Sarcoidosis (D) can cause hypercalcemia, but it is usually associated with increased serum levels of angiotensin-converting enzyme (ACE) and abnormalities in chest X-rays or CT scans, which are not mentioned in the case.  Vitamin D excess (E) can lead to hypercalcemia, but the PTH level in this case is within the normal range, making primary hyperparathyroidism a more likely diagnosis.  It's important for the patient to follow up with a healthcare provider for further evaluation, including imaging studies and possible referral to an endocrinologist for confirmation and management of primary hyperparathyroidism. |
|  |  | Attempt 2  Based on the provided information and the laboratory values, the most likely diagnosis is C. Primary hyperparathyroidism.  Primary hyperparathyroidism is characterized by elevated levels of parathyroid hormone (PTH) and serum calcium. In this case, the patient has elevated serum calcium (2.9 mmol/L) and slightly elevated PTH (7.9 pmol/L), along with low phosphate levels. These findings are indicative of an excessive release of calcium from the bones and decreased reabsorption of phosphate from the kidneys, which are typical in primary hyperparathyroidism.  The other options (A. Bony metastases, B. Excess calcium intake, D. Sarcoidosis, E. Vitamin D excess) may also lead to hypercalcemia, but the specific combination of laboratory values and the clinical context point more strongly toward primary hyperparathyroidism.  It's important for the patient to undergo further evaluation and consultation with a healthcare professional to confirm the diagnosis and determine the appropriate management plan. |
|  |  | Attempt 3  Based on the given clinical information and investigation results, the most likely diagnosis in this case is:  C. Primary hyperparathyroidism  Primary hyperparathyroidism is characterized by excessive production of parathyroid hormone (PTH) by the parathyroid glands, leading to increased levels of serum calcium (hypercalcemia). The elevated serum corrected calcium level in this patient (2.9 mmol/L) is above the normal range.  Other findings associated with primary hyperparathyroidism include normal or low phosphate levels (as seen in this case), increased alkaline phosphatase levels (indicating bone turnover), and often normal serum electrolytes and urea.  The symptoms of weight loss, fatigue, and polyuria can be attributed to the effects of hypercalcemia on various organ systems. Polyuria is commonly seen due to the diuretic effect of hypercalcemia on the kidneys.  Excess calcium intake and vitamin D excess are less likely in this case since the patient's symptoms are prolonged and not related to recent changes in supplementation. Bony metastases may cause hypercalcemia, but the absence of other signs of malignancy makes it less likely. Sarcoidosis can also cause hypercalcemia, but it typically presents with other systemic manifestations.  Further evaluation, including imaging (e.g., neck ultrasound) and referral to an endocrinologist, may be necessary to confirm the diagnosis and determine the underlying cause of primary hyperparathyroidism. |
| **63** | A 59 year old man has a 1 year history of erectile dysfunction. He has a history of angina, type 2 diabetes mellitus and peripheral vascular disease. He had a thyroidectomy 2 years ago for thyrotoxicosis. His regular medications are aspirin, diltiazem, levothyroxine, metformin, ramipril and simvastatin. His BP is 140/90 mmHg lying and 135/85 mmHg standing. His foot pulses are not palpable. He has normal sensation in his feet.  Which is most likely to be the main cause of his erectile dysfunction?  A. Adverse effect of medication  B. Autonomic neuropathy  C. Hypothyroidism  D. Testosterone deficiency  E. Vascular insufficiency | Attempt 1  In this case, the most likely cause of the patient's erectile dysfunction is:  E. Vascular insufficiency  The patient's medical history, which includes peripheral vascular disease and diabetes mellitus, suggests a potential impairment of blood flow to the penis. Both conditions can lead to damage and narrowing of blood vessels, reducing the ability to achieve and maintain an erection.  While medications can contribute to erectile dysfunction, the patient's medication list does not include any known culprits for this side effect. Autonomic neuropathy (B) can also contribute to erectile dysfunction in individuals with diabetes, but the absence of sensory impairment in the patient's feet suggests that autonomic neuropathy may not be the primary cause in this case.  Hypothyroidism (C) can cause various symptoms, including sexual dysfunction, but the patient has been on levothyroxine replacement therapy, which should correct any hormonal imbalances.  Testosterone deficiency (D) could potentially contribute to erectile dysfunction, but it is less likely to be the main cause in this case compared to vascular insufficiency.  Given the patient's medical history, peripheral vascular disease, and lack of palpable foot pulses, vascular insufficiency emerges as the most likely cause of his erectile dysfunction. It is important for him to discuss this concern with his healthcare provider to determine the most appropriate management and treatment options. |
|  |  | Attempt 2  Given the patient's history of peripheral vascular disease and lack of palpable foot pulses, the most likely cause of his erectile dysfunction is E. Vascular insufficiency.  Peripheral vascular disease can lead to reduced blood flow to the lower extremities and other parts of the body, including the genital area. Reduced blood flow can affect the ability to achieve and maintain an erection, contributing to erectile dysfunction.  While other factors like medication side effects, autonomic neuropathy, hypothyroidism, and testosterone deficiency can also play a role in erectile dysfunction, the patient's vascular issues are particularly relevant in this case.  It's important for the patient to discuss his concerns with a healthcare professional, as they can provide a comprehensive evaluation and guidance on appropriate management options. |
|  |  | Attempt 3  In this case, the most likely main cause of the patient's erectile dysfunction is:  E. Vascular insufficiency  The patient's medical history, which includes peripheral vascular disease and the absence of palpable foot pulses, suggests the presence of vascular pathology. Erectile dysfunction is commonly associated with underlying vascular insufficiency, particularly in patients with a history of peripheral vascular disease or cardiovascular risk factors.  The patient's risk factors, such as angina, type 2 diabetes mellitus, and peripheral vascular disease, contribute to endothelial dysfunction and impaired blood flow to the penile arteries. This vascular insufficiency can lead to inadequate blood flow and subsequent difficulty in achieving or maintaining an erection.  While medication side effects (A) can contribute to erectile dysfunction, in this case, the patient's medication regimen does not include any known common causes of erectile dysfunction. Autonomic neuropathy (B) can also contribute to erectile dysfunction, but the patient's normal sensation in his feet suggests that autonomic neuropathy may not be the primary cause.  Hypothyroidism (C) can affect sexual function, including erectile dysfunction, but the patient's levothyroxine therapy indicates that his thyroid function is being adequately replaced. Testosterone deficiency (D) could potentially contribute to erectile dysfunction, but it is less likely to be the main cause in this case, given the presence of known vascular risk factors and the absence of other symptoms suggestive of low testosterone levels.  Therefore, vascular insufficiency is the most likely main cause of the patient's erectile dysfunction, considering his medical history, risk factors, and physical examination findings. Further evaluation and management aimed at improving vascular health, such as optimizing cardiovascular risk factors and considering targeted therapies, may be appropriate. Referral to a specialist, such as a urologist, may also be warranted for a comprehensive assessment and management of erectile dysfunction. |
| **64** | A 52 year old man has had 3 months of fatigue. He has a history of ulcerative colitis and takes mesalazine. He drinks 20 units of alcohol per week. His temperature is 36.8°C and pulse rate 80 bpm. He has 3 cm hepatomegaly. Investigations: Albumin 36 g/L (35–50) ALT 65 IU/L (10–50) ALP 580 IU/L (25–115) Bilirubin 18 μmol/L (<17) γGT 230 IU/L (9–40)  Which is the most likely diagnosis?  A. Alcoholic hepatitis  B. Cholangiocarcinoma  C. Choledocholithiasis  D. Hepatocellular carcinoma  E. Primary sclerosing cholangitis | Attempt 1  Based on the provided information and laboratory results, the most likely diagnosis in this case is:  A. Alcoholic hepatitis  The patient's history of ulcerative colitis, alcohol consumption (20 units per week), and hepatomegaly are suggestive of alcoholic hepatitis. The elevated liver enzymes, including ALT, ALP, and γGT, further support this diagnosis. Alcoholic hepatitis is characterized by inflammation and liver damage caused by excessive alcohol consumption.  Cholangiocarcinoma (B) and primary sclerosing cholangitis (E) typically present with different clinical features and laboratory findings. Choledocholithiasis (C), which refers to the presence of stones in the common bile duct, can cause symptoms such as abdominal pain and jaundice but does not typically lead to hepatomegaly and the specific pattern of liver enzyme elevation seen in this case.  Hepatocellular carcinoma (D) may be considered in patients with chronic liver disease, such as cirrhosis, but the presence of hepatomegaly and the pattern of liver enzyme elevation are more consistent with alcoholic hepatitis in this case.  It is important for the patient to receive appropriate medical management and guidance regarding alcohol consumption to prevent further liver damage and complications. |
|  |  | Attempt 2  Based on the provided information and the laboratory values, the most likely diagnosis is A. Alcoholic hepatitis.  The patient's history of alcohol consumption (20 units per week), hepatomegaly, elevated liver enzymes (ALT, ALP, γGT), and bilirubin within slightly elevated range are indicative of alcoholic hepatitis. Alcoholic hepatitis is a serious inflammation of the liver that can occur due to excessive alcohol consumption.  While other conditions like cholangiocarcinoma, choledocholithiasis, hepatocellular carcinoma, and primary sclerosing cholangitis can also present with elevated liver enzymes and hepatomegaly, the patient's history of alcohol consumption is a significant factor pointing towards alcoholic hepatitis as the primary cause.  It's important for the patient to seek medical attention for proper evaluation, diagnosis, and management. Reducing or stopping alcohol consumption is a crucial step in managing alcoholic hepatitis. |
|  |  | Attempt 3  The most likely diagnosis in this case is:  A. Alcoholic hepatitis  The patient's history of alcohol consumption, elevated liver enzymes (ALT, ALP, γGT), hepatomegaly, and fatigue are consistent with alcoholic hepatitis. Alcoholic hepatitis is an inflammation of the liver caused by excessive alcohol consumption. It typically presents with hepatomegaly, jaundice, and elevated liver enzymes.  The patient's elevated γGT and ALP levels suggest cholestasis, which is commonly seen in alcoholic liver disease. Although not specific, it further supports the diagnosis of alcoholic hepatitis.  Cholangiocarcinoma (B) is a type of bile duct cancer that typically presents with obstructive jaundice and is less likely in this case. Choledocholithiasis (C) refers to the presence of stones in the common bile duct and usually presents with symptoms of biliary obstruction such as jaundice and abdominal pain, which are not prominent in this patient's presentation.  Hepatocellular carcinoma (D) is a primary liver cancer that can be associated with underlying liver disease, such as chronic hepatitis B or C infection or cirrhosis. While chronic alcohol abuse can increase the risk of hepatocellular carcinoma, the clinical presentation and laboratory findings in this case are more suggestive of alcoholic hepatitis.  Primary sclerosing cholangitis (E) is a chronic liver disease characterized by inflammation and fibrosis of the bile ducts. It is commonly associated with ulcerative colitis. However, the clinical findings in this case, such as hepatomegaly, elevated liver enzymes, and the patient's alcohol consumption, are more indicative of alcoholic hepatitis.  It is important to note that further evaluation, including imaging studies and possibly liver biopsy, may be necessary to confirm the diagnosis and assess the severity of liver damage. Additionally, addressing the patient's alcohol consumption is essential for management and preventing further liver damage. Referral to a gastroenterologist or hepatologist is recommended for further evaluation and management. |
| **65** | A 73 year old man has increasing breathlessness over 1 week. He has a history of chronic kidney disease and ischaemic heart disease. He takes alfacalcidol, aspirin, atorvastatin, bisoprolol fumarate, furosemide and irbesartan. There are bibasal inspiratory crepitations and mild peripheral oedema. Investigations: Sodium 134 mmol/L (135–146) Potassium 6.7 mmol/L (3.5–5.3) Urea19 mmol/L (2.5–7.8) Creatinine 259 μmol/L (60–120) eGFR 23 mL/min/1.73 m2 (>60)  Which drug is most likely to be contributing to his hyperkalaemia?  A. Alfacalcidol  B. Aspirin  C. Bisoprolol fumarate  D. Furosemide  E. Irbesartan | Attempt 1  In this case, the drug most likely contributing to the patient's hyperkalemia is:  E. Irbesartan  Irbesartan is an angiotensin II receptor blocker (ARB) used for the treatment of hypertension and to protect kidney function in patients with chronic kidney disease. However, one potential side effect of ARBs is hyperkalemia, which is an elevated potassium level in the blood. Irbesartan works by blocking the effects of angiotensin II, which can lead to decreased aldosterone secretion. Aldosterone promotes potassium excretion in the kidneys, so decreased aldosterone levels can result in potassium retention and hyperkalemia.  The patient's elevated potassium level (6.7 mmol/L) is above the normal range (3.5-5.3 mmol/L) and suggests impaired potassium regulation. Given the patient's history of chronic kidney disease and the use of irbesartan, it is likely that the medication is contributing to the hyperkalemia.  It is important to monitor and manage the patient's potassium levels closely and consider adjusting the medication regimen as needed to prevent complications associated with hyperkalemia. |
|  |  | Attempt 2  The drug most likely to be contributing to his hyperkalemia is E. Irbesartan.  Irbesartan is an angiotensin II receptor blocker (ARB) commonly used to treat conditions like hypertension and heart failure. However, ARBs can cause an increase in serum potassium levels, especially in patients with pre-existing kidney disease. Since this patient has chronic kidney disease with reduced kidney function (eGFR of 23 mL/min/1.73 m²), the impaired renal function can contribute to decreased potassium excretion, making him more susceptible to hyperkalemia induced by medications like irbesartan.  It's important for the patient to consult his healthcare provider for further evaluation and potential adjustments to his medication regimen, especially considering his hyperkalemia and underlying health conditions. |
|  |  | Attempt 3  The drug that is most likely contributing to the hyperkalemia in this patient is:  E. Irbesartan  Irbesartan is an angiotensin II receptor blocker (ARB) commonly used in the management of hypertension and heart failure. One of the side effects of ARBs is hyperkalemia, which is an elevated potassium level in the blood. ARBs inhibit the action of angiotensin II on the renin-angiotensin-aldosterone system (RAAS), leading to reduced aldosterone secretion. Aldosterone promotes potassium excretion in the kidneys, so its inhibition can result in decreased potassium excretion and subsequent hyperkalemia.  In this patient, the presence of hyperkalemia (potassium level of 6.7 mmol/L) along with the use of irbesartan suggests that the medication is contributing to the elevated potassium level. The patient's chronic kidney disease (CKD) can further impair potassium excretion, making him more susceptible to developing hyperkalemia when taking medications that affect potassium levels.  Other medications in the patient's regimen, such as furosemide (D) and alfacalcidol (A), can also have an impact on potassium levels. Furosemide is a loop diuretic that promotes potassium excretion and can lower potassium levels. Alfacalcidol is a vitamin D analog and is not typically associated with hyperkalemia.  Aspirin (B) and bisoprolol fumarate (C) are not known to directly cause hyperkalemia. Aspirin is a nonsteroidal anti-inflammatory drug (NSAID) that does not significantly affect potassium levels. Bisoprolol is a beta-blocker and is not typically associated with potassium imbalances.  It is important to monitor and manage hyperkalemia appropriately, considering the patient's renal function, medications, and overall clinical status. Adjustments to the medication regimen or potassium-lowering interventions may be necessary in consultation with a healthcare professional. |
| **66** | A 65 year old man is invited to the abdominal aortic aneurysm screening programme. An ultrasound scan shows his abdominal aorta to be 33 mm in diameter.  Which is the most appropriate management plan?  A. Reassure and discharge  B. Refer for angiography  C. Refer for vascular surgery  D. Repeat ultrasound scan in 12 months  E. Request CT scan of abdomen | Attempt 1  The most appropriate management plan for a 65-year-old man with an abdominal aortic aneurysm measuring 33 mm in diameter is:  D. Repeat ultrasound scan in 12 months  The size of the abdominal aorta is an important factor in determining the management of an abdominal aortic aneurysm (AAA). The threshold for intervention typically depends on the size of the aneurysm. In this case, the aneurysm is 33 mm in diameter, which falls within the surveillance range.  According to current guidelines, an AAA measuring between 30-54 mm in diameter is usually managed with surveillance and periodic imaging. The recommended follow-up interval is generally every 12 months to monitor any changes in the aneurysm size.  Therefore, the most appropriate management plan would be to repeat the ultrasound scan in 12 months to assess if there is any progression in the size of the aneurysm. This approach allows for close monitoring and timely intervention if the aneurysm size reaches the threshold for surgical repair.  It is important to note that the management of abdominal aortic aneurysms may vary based on individual patient characteristics, comorbidities, and clinical judgment. Consulting with a vascular specialist or following local guidelines is essential for personalized management decisions. |
|  |  | Attempt 2  The most appropriate management plan for a 65-year-old man with an abdominal aortic diameter of 33 mm is D. Repeat ultrasound scan in 12 months.  Abdominal aortic aneurysms (AAAs) are typically monitored based on their size. The threshold for intervention varies, but generally, an aneurysm size of 5.5 cm or larger is considered for surgical repair. In this case, with an aortic diameter of 33 mm (3.3 cm), the aneurysm is still below the threshold for intervention.  The recommended management plan for an AAA measuring less than 5.5 cm is to repeat ultrasound scans at regular intervals to monitor its growth. A 12-month interval is a reasonable timeframe for follow-up in this situation.  As the aneurysm grows, the healthcare provider will assess the risk and potential benefits of intervention based on factors such as the patient's age, overall health, and aneurysm characteristics. Regular monitoring is essential to catch any significant increase in size that might warrant further evaluation or intervention. |
|  |  | Attempt 3  The most appropriate management plan for a 65-year-old man with an abdominal aortic diameter of 33 mm detected during an abdominal aortic aneurysm (AAA) screening is:  D. Repeat ultrasound scan in 12 months  The threshold for intervention in the management of abdominal aortic aneurysms is typically based on the diameter of the aneurysm. In general, the decision for intervention is guided by the size of the aneurysm and the risk of rupture. The current guidelines recommend surveillance with periodic ultrasound scans for aneurysms between 3.0 and 5.4 cm in diameter.  In this case, the abdominal aortic diameter of 33 mm falls within the range of 3.0 to 5.4 cm. As the diameter is below the threshold for intervention, the most appropriate management plan would be to repeat the ultrasound scan in 12 months. This allows for ongoing surveillance to monitor any potential growth of the aneurysm.  Reassurance and discharge (A) may not be appropriate since the patient has an abdominal aortic aneurysm that requires ongoing monitoring.  Referral for angiography (B) or CT scan of the abdomen (E) is not necessary at this stage, as the aneurysm is below the intervention threshold. These imaging modalities may be reserved for cases where there is a need for further evaluation or if intervention becomes necessary.  Referral for vascular surgery (C) is not warranted at this point since the aneurysm diameter is below the threshold for intervention.  Regular surveillance with ultrasound scans is the recommended approach for monitoring the size and progression of the abdominal aortic aneurysm in this case. |
| **67** | A 28 year old woman has pain on swallowing. She has a history of asthma that is well controlled using metered dose salbutamol and beclometasone dipropionate (800 micrograms/day) inhalers. She has white plaques in her mouth. An anti-fungal oral suspension is prescribed.  What is the most appropriate management with regard to her beclometasone?  A. Change beclometasone dipropionate to a dry powder formulation  B. Change beclometasone dipropionate to fluticasone  C. Change beclometasone dipriopionate to salmeterol  D. Take beclometasone dipropionate using a large volume spacer  E. Take salbutamol and beclometasone dipropionate at least 1 h apart | Attempt 1  The most appropriate management with regard to the patient's beclometasone dipropionate inhaler in the context of her oral fungal infection is:  A. Change beclometasone dipropionate to a dry powder formulation  Oral fungal infections, such as oral thrush (caused by Candida species), can be associated with the use of inhaled corticosteroids like beclometasone dipropionate. The use of a metered dose inhaler (MDI) with a spacer can increase the risk of deposition of the medication in the mouth and throat, leading to local side effects such as oral thrush.  Switching the patient from a metered dose inhaler (MDI) formulation of beclometasone dipropionate to a dry powder inhaler (DPI) can help reduce the risk of local side effects, including oral fungal infections. Dry powder formulations generally have a lower risk of depositing the medication in the oropharynx, minimizing the exposure to the oral mucosa.  Therefore, changing the beclometasone dipropionate to a dry powder formulation would be the most appropriate management strategy in this case. It is important to discuss this change with the patient and provide proper instructions on the use of the new inhaler device.  It's worth noting that the management decisions for individual patients may vary, and it's always recommended to consult with a healthcare professional or the patient's prescribing physician for personalized advice. |
|  |  | Attempt 2  Given the patient's symptoms of white plaques in her mouth and the history of using beclometasone dipropionate inhaler, the most appropriate management in this context is A. Change beclometasone dipropionate to a dry powder formulation.  Dry powder inhalers are less likely to contribute to the development of oral thrush (candidiasis) compared to metered-dose inhalers, as they tend to deposit medication deeper into the lungs and have less impact on the oral cavity. This can help reduce the risk of fungal overgrowth in the mouth and subsequent symptoms of pain on swallowing and white plaques.  It's important for the patient to follow up with her healthcare provider for further guidance and to ensure effective asthma management while minimizing the risk of oral candidiasis. |
|  |  | Attempt 3  The most appropriate management regarding the use of beclometasone dipropionate inhaler in a 28-year-old woman with pain on swallowing and white plaques in her mouth is:  A. Change beclometasone dipropionate to a dry powder formulation  Beclometasone dipropionate is a corticosteroid inhaler commonly used for asthma control. It is important to minimize the risk of developing oral candidiasis (thrush), which can occur as a side effect of inhaled corticosteroids. In this case, the presence of white plaques in her mouth suggests a possible fungal infection.  Switching to a dry powder formulation of the corticosteroid inhaler, such as dry powder beclometasone dipropionate or an alternative dry powder corticosteroid inhaler, can help reduce the risk of oral candidiasis. Dry powder formulations are less likely to deposit corticosteroids in the oral cavity, reducing the local side effects.  The other options listed are not the most appropriate management choices:  - Changing beclometasone dipropionate to fluticasone (B) or salmeterol (C) would not directly address the risk of oral candidiasis. These are alternative corticosteroid or long-acting beta-agonist inhalers and do not change the drug delivery mechanism.  - Taking beclometasone dipropionate using a large volume spacer (D) may help reduce local side effects but may not fully eliminate the risk of oral candidiasis.  - Taking salbutamol and beclometasone dipropionate at least 1 hour apart (E) is not directly related to the management of oral candidiasis. It is recommended to separate the timing of short-acting beta-agonists and corticosteroids to optimize their effects, but this does not address the risk of fungal infection.  Therefore, changing the beclometasone dipropionate to a dry powder formulation is the most appropriate management strategy to minimize the risk of oral candidiasis while maintaining asthma control. |
| **68** | A 37 year old man has increasing fever and shortness of breath. He was admitted 3 days ago with left lower lobe pneumonia due penicillin-sensitive Streptococcus pneumoniae. He is receiving intravenous benzylpenicillin 1.2 g four times daily. His temperature is 38.5°C, pulse 100 bpm and BP 122/80 mmHg. Investigations: Chest X-ray: left basal effusion. Diagnostic pleural aspiration: Purulent fluid. Microscopy shows numerous polymorphs and Gram-positive cocci  Which is the most appropriate next step in management?  A. Catheter thoracostomy drainage  B. Increase dose of benzylpenicillin  C. Open thoracostomy  D. Switch benzylpenicillin to ceftriaxone  E. Switch benzylpenicillin to vancomycin | Attempt 1  The most appropriate next step in management for this patient with purulent pleural fluid and left basal effusion is:  A. Catheter thoracostomy drainage  The presence of purulent fluid on diagnostic pleural aspiration, along with the chest X-ray findings of a left basal effusion, suggests the development of a parapneumonic effusion. Parapneumonic effusions can occur as a complication of pneumonia, and when the effusion is infected (empyema), prompt intervention is necessary.  The initial management of an empyema involves drainage of the pleural fluid. Catheter thoracostomy drainage, also known as tube thoracostomy, is the recommended first-line intervention. It involves the insertion of a chest tube into the pleural space to drain the infected fluid and facilitate lung re-expansion.  Increasing the dose of benzylpenicillin or switching to an alternative antibiotic alone would not be sufficient to address the empyema. Antibiotics play a role in the treatment of parapneumonic effusions, but drainage is crucial to remove the infected fluid and prevent further complications.  Open thoracostomy is a more invasive surgical procedure that may be considered if the catheter drainage is not effective or in certain cases where there are specific indications. However, it is not the initial step in management.  Therefore, the most appropriate next step in management for this patient is catheter thoracostomy drainage to address the empyema and promote resolution of the infection. |
|  |  | Attempt 2  Given the patient's clinical presentation, the presence of purulent fluid in the pleural aspiration, and the possibility of empyema, the most appropriate next step in management is A. Catheter thoracostomy drainage.  Empyema is a collection of pus in the pleural space, and it requires appropriate drainage to effectively treat the infection. Catheter thoracostomy, also known as tube thoracostomy, involves the insertion of a chest tube into the pleural space to drain the accumulated fluid and pus. This drainage helps alleviate symptoms, prevent complications, and allow for better antibiotic penetration to the infection site.  While adjusting antibiotics may also be necessary based on culture and sensitivity results, the priority in this case is to address the pleural collection by draining the empyema. This procedure is usually guided by imaging studies, and it's crucial to involve a medical professional for proper assessment and intervention. |
|  |  | Attempt 3  Based on the clinical presentation and investigation findings, the most appropriate next step in management for the 37-year-old man with increasing fever and shortness of breath is:  A. Catheter thoracostomy drainage  The presence of a left basal effusion and purulent fluid on diagnostic pleural aspiration suggests the development of a parapneumonic effusion or empyema, which is a complication of pneumonia. The appropriate management for this patient would be catheter thoracostomy drainage, also known as tube thoracostomy or chest tube insertion. This procedure involves inserting a chest tube into the pleural space to drain the infected fluid and facilitate lung re-expansion.  Increasing the dose of benzylpenicillin (B) alone may not adequately address the need for drainage of the infected fluid. Although antibiotic therapy is essential in treating the underlying infection, the presence of a significant effusion requires drainage for optimal management.  Open thoracostomy (C) is a more invasive surgical procedure that is typically reserved for cases in which tube thoracostomy is unsuccessful or if there are specific indications for open surgical intervention. It is not the initial step in the management of uncomplicated parapneumonic effusion.  Switching benzylpenicillin to ceftriaxone (D) or vancomycin (E) is not the immediate priority in management. Antibiotic therapy with benzylpenicillin, which covers penicillin-sensitive Streptococcus pneumoniae, is already being administered. The primary concern at this stage is drainage of the infected fluid from the pleural space.  Therefore, the most appropriate next step in management for this patient is catheter thoracostomy drainage to address the parapneumonic effusion or empyema. |
| **69** | A 52 year old man has 4 weeks of joint pain, fever and weight loss. He is a non-smoker and has no significant medical history. Examination is unremarkable. Investigations: Calcium 3.12 mmol/L (2.2–2.6) Plasma parathyroid hormone <0.5 pmol/L (0.9–5.4) Chest X-ray shows perihilar lymphadenopathy.  Which is the most likely diagnosis?  A. Granulomatosis with polyangiitis  B. Hodgkin's lymphoma  C. Primary hyperparathyroidism  D. Sarcoidosis  E. Tuberculosis | Attempt 1  Given the clinical presentation of joint pain, fever, weight loss, and the presence of perihilar lymphadenopathy on chest X-ray, the most likely diagnosis in this case is:  D. Sarcoidosis  Sarcoidosis is a systemic inflammatory disease characterized by the formation of non-caseating granulomas in various organs, including the lungs, lymph nodes, and joints. It commonly presents with symptoms such as fever, weight loss, and joint pain.  The laboratory findings in this case are consistent with sarcoidosis. The low plasma parathyroid hormone (PTH) level suggests suppressed PTH production, which can occur in sarcoidosis due to increased production of vitamin D by the granulomas. This, in turn, leads to increased calcium levels, as seen in the elevated serum calcium level.  Perihilar lymphadenopathy on chest X-ray is a classic finding in sarcoidosis, as the disease often involves the mediastinal lymph nodes.  While other conditions such as tuberculosis, Hodgkin's lymphoma, granulomatosis with polyangiitis, and primary hyperparathyroidism can also present with similar symptoms or findings, the combination of joint pain, fever, weight loss, low PTH, and perihilar lymphadenopathy in this case strongly suggests sarcoidosis as the most likely diagnosis. |
|  |  | Attempt 2  Given the clinical presentation of joint pain, fever, weight loss, and perihilar lymphadenopathy on chest X-ray, along with low plasma parathyroid hormone (PTH) levels and elevated calcium, the most likely diagnosis in this case is D. Sarcoidosis.  Sarcoidosis is a multisystem disorder characterized by the formation of non-caseating granulomas in various organs, including the lungs, lymph nodes, skin, and joints. The perihilar lymphadenopathy seen on the chest X-ray is a characteristic finding in sarcoidosis. Additionally, the elevated calcium levels and low PTH levels are consistent with the development of hypercalcemia, which can occur in sarcoidosis due to increased production of activated vitamin D by macrophages in the granulomas.  The other conditions listed are possibilities to consider, but given the combination of clinical findings, sarcoidosis is the most likely explanation. Further evaluation and consultation with a healthcare professional are important for accurate diagnosis and management. |
|  |  | Attempt 3  The most likely diagnosis for the 52-year-old man with 4 weeks of joint pain, fever, weight loss, and perihilar lymphadenopathy on chest X-ray is:  D. Sarcoidosis  Sarcoidosis is a systemic granulomatous disorder characterized by the formation of non-caseating granulomas in various organs, particularly the lungs and lymph nodes. The clinical presentation often includes constitutional symptoms such as fever and weight loss, as well as joint pain. Perihilar lymphadenopathy is a typical finding on chest X-ray in sarcoidosis.  The low calcium level (hypocalcemia) and suppressed plasma parathyroid hormone (PTH) level are consistent with sarcoidosis-associated hypercalcemia, which can occur due to increased production of 1,25-dihydroxyvitamin D by activated macrophages within the granulomas. This leads to enhanced intestinal calcium absorption and subsequent hypoparathyroidism.  Granulomatosis with polyangiitis (A) typically presents with respiratory symptoms such as cough, sinusitis, and hemoptysis, along with renal involvement and systemic vasculitis. The absence of respiratory and renal symptoms makes this diagnosis less likely.  Hodgkin's lymphoma (B) may present with constitutional symptoms, lymphadenopathy, and occasionally with systemic symptoms such as fever and weight loss. However, the absence of other characteristic features such as Reed-Sternberg cells in lymph node biopsy or B symptoms (night sweats, pruritus) makes this diagnosis less likely.  Primary hyperparathyroidism (C) is characterized by hypercalcemia and elevated PTH levels. The findings in this patient, however, indicate hypocalcemia and suppressed PTH, which are not consistent with primary hyperparathyroidism.  Tuberculosis (E) can cause constitutional symptoms, lymphadenopathy, and weight loss. However, the perihilar lymphadenopathy seen on chest X-ray in this case is more suggestive of sarcoidosis. Additionally, tuberculosis typically presents with respiratory symptoms such as cough and may show evidence of pulmonary involvement on imaging.  Therefore, based on the clinical presentation, laboratory findings, and imaging results, the most likely diagnosis is sarcoidosis. Further evaluation and confirmation can be pursued with additional tests, such as biopsy of an affected organ or imaging studies targeting specific areas of involvement. |
| **70** | A 56 year old man has had a single episode of painless visible haematuria. He has no other urinary symptoms and is otherwise fit and well. He has smoked ten cigarettes per day for the past 35 years. He has a BP of 140/85 mmHg. Urinalysis performed after this episode shows blood 2+, no protein and no nitrites. Investigations: Urea 6.5 mmol/L (2.5–7.8) Creatinine 95 μmol/L (60–120) Urine culture: no growth  Which investigation is most likely to confirm the diagnosis?  A. Flexible cystoscopy  B. Serum prostate specific antigen  C. Transrectal ultrasound scan of prostate  D. Ultrasound scan of kidneys  E. Urine cytology | Attempt 1  Given the presentation of painless visible haematuria in a 56-year-old man with a history of smoking, the most appropriate investigation to confirm the diagnosis in this case would be:  A. Flexible cystoscopy  Painless visible haematuria in an adult, especially in the presence of risk factors such as smoking, warrants further investigation to rule out underlying urological malignancy, particularly bladder cancer. Flexible cystoscopy is the gold standard for evaluating the lower urinary tract, including the bladder. It allows direct visualization of the bladder lining and the identification of any abnormal lesions or tumors.  While other investigations such as serum prostate-specific antigen (PSA) and transrectal ultrasound scan of the prostate may be relevant for evaluating prostate-related conditions, they are not the primary investigations of choice in this scenario of painless visible haematuria.  Ultrasound scan of the kidneys is more useful for evaluating renal pathology rather than specifically diagnosing the cause of haematuria.  Urine cytology is a valuable test to detect abnormal cells in the urine, but it has limited sensitivity, especially for early-stage bladder cancer, and may not be the initial investigation of choice.  Therefore, given the clinical scenario, the most appropriate investigation to confirm the diagnosis would be a flexible cystoscopy. |
|  |  | Attempt 2  Given the patient's risk factors (smoking) and the presence of painless visible haematuria, the most appropriate investigation to confirm the diagnosis would be A. Flexible cystoscopy.  Painless visible haematuria in a smoker raises concern for urinary tract malignancies, such as bladder cancer. Flexible cystoscopy allows direct visualization of the bladder lining, which can help identify any abnormalities, lesions, or tumors. It is considered a gold standard for evaluating the cause of haematuria, especially in individuals with risk factors like smoking.  However, it's important to consult with a healthcare professional for proper evaluation and management, as individual cases may vary. |
|  |  | Attempt 3  In a 56-year-old man with a single episode of painless visible hematuria, the most appropriate investigation to confirm the diagnosis would be:  A. Flexible cystoscopy  Painless visible hematuria in an adult, especially in the presence of risk factors such as smoking, requires further evaluation to rule out underlying urinary tract malignancy. Flexible cystoscopy is the most appropriate and widely recommended investigation for evaluating hematuria. It allows direct visualization of the urethra, bladder, and ureteric orifices, providing a detailed examination of the lower urinary tract.  Serum prostate-specific antigen (PSA) (B) is a blood test primarily used for the detection of prostate cancer. However, in this case, the presentation is suggestive of urinary tract pathology rather than prostate-specific issues.  Transrectal ultrasound scan of the prostate (C) is not the first-line investigation for evaluating hematuria. It is primarily used in the assessment of prostate-related conditions such as prostate enlargement or cancer.  Ultrasound scan of the kidneys (D) is helpful for evaluating the kidneys, but it is not the primary investigation for determining the cause of hematuria. It may be performed as part of a comprehensive evaluation but is not specific for confirming the diagnosis.  Urine cytology (E) is a diagnostic test that examines the cells in the urine for any abnormal changes or cancerous cells. While it can be useful in detecting certain types of urinary tract malignancies, it is not the initial investigation of choice for evaluating hematuria. Flexible cystoscopy provides a direct visualization of the bladder and urethra, allowing for a more accurate assessment.  Therefore, in the context of painless visible hematuria, the most appropriate investigation to confirm the diagnosis is flexible cystoscopy. |
| **71** | The daughter of a 78 year old man is concerned about an area of redness on her father's back. He lives alone and spends most of his day sitting in a chair. There is a reddened area over the sacrum, but his skin is intact. He is considered to be at risk of pressure ulcers.  Which member of the community multidisciplinary team would be most suited to conduct an initial assessment?  A. Dietician  B. District nurse  C. Occupational therapist  D. Physiotherapist  E. Tissue viability nurse | Attempt 1  In this scenario, the most suitable member of the community multidisciplinary team to conduct an initial assessment would be:  E. Tissue viability nurse  A tissue viability nurse specializes in the prevention, assessment, and management of wounds, including pressure ulcers. They are trained to evaluate the risk factors for pressure ulcers and assess the integrity of the skin. Given the concerns about the redness on the patient's back and the fact that he is at risk of pressure ulcers due to prolonged sitting, a tissue viability nurse would be the most appropriate professional to conduct an initial assessment.  While other members of the community multidisciplinary team, such as a dietician, district nurse, occupational therapist, or physiotherapist, may be involved in the overall care and management of the patient, a tissue viability nurse has specific expertise in assessing and managing pressure ulcers, making them the most suited for the initial assessment in this case. |
|  |  | Attempt 2  E. Tissue viability nurse  A tissue viability nurse specializes in assessing, preventing, and managing wounds, including pressure ulcers. Given the concern about a reddened area over the sacrum and the patient's risk of pressure ulcers due to prolonged sitting, a tissue viability nurse would be the most appropriate member of the community multidisciplinary team to conduct an initial assessment. They have expertise in evaluating skin integrity, recommending preventive measures, and providing guidance on wound management to prevent further complications. |
|  |  | Attempt 3  In this scenario, the most appropriate member of the community multidisciplinary team to conduct an initial assessment of the redness on the man's back and his risk of pressure ulcers would be:  E. Tissue viability nurse  A tissue viability nurse is a healthcare professional who specializes in assessing, preventing, and managing wounds, including pressure ulcers. They have expertise in wound care and are skilled in assessing the risk factors for developing pressure ulcers. They can evaluate the redness on the man's back, assess the condition of the skin, and determine the risk level for pressure ulcers. They can also provide guidance on appropriate preventive measures and recommend interventions to minimize the risk of pressure ulcers.  While other members of the community multidisciplinary team, such as a dietician (A), district nurse (B), occupational therapist (C), or physiotherapist (D), may have important roles in the overall care of the patient, the tissue viability nurse is specifically trained in assessing and managing wounds, including pressure ulcers, making them the most suitable choice for the initial assessment in this case. |
| **72** | A junior doctor sustains a needle-stick injury while inserting a venous cannula into a 28 year old man. The patient has a history of intravenous drug use.  Which is the most important first action?  A. Encourage bleeding from the wound  B. Make an appointment with occupational health  C. Seek post-exposure prophylaxis  D. Test the patient for HIV  E. Wash the wound with alcohol | Attempt 1  The most important first action in the case of a needle-stick injury is to prioritize personal safety and take immediate steps to minimize the risk of infection. In this scenario, the healthcare worker has sustained a needle-stick injury while inserting a venous cannula into a patient with a history of intravenous drug use.  The correct answer is:  C. Seek post-exposure prophylaxis  Post-exposure prophylaxis (PEP) involves taking specific medications after an exposure to a potentially infectious source, such as a needle-stick injury. PEP is aimed at reducing the risk of transmission of bloodborne pathogens, including HIV and hepatitis B and C. It is crucial to initiate PEP as soon as possible, preferably within hours of the exposure, to maximize its effectiveness.  While it is important to address the wound and ensure appropriate wound care, seeking post-exposure prophylaxis takes precedence in order to minimize the risk of potential infection. Following the initial steps to ensure personal safety, the healthcare worker should promptly seek medical advice and consult with occupational health or designated personnel responsible for managing needle-stick injuries in their healthcare setting. This will enable them to undergo an assessment, receive appropriate counseling, and initiate the necessary measures, including post-exposure prophylaxis, as deemed necessary based on the specific circumstances of the exposure.  Testing the patient for HIV and other bloodborne pathogens is important, but it should be performed as part of the follow-up and management process rather than being the immediate first action. |
|  |  | Attempt 2  C. Seek post-exposure prophylaxis  In the case of a needle-stick injury involving a patient with a history of intravenous drug use, the most important first action is to seek post-exposure prophylaxis (PEP) to prevent potential transmission of bloodborne infections such as HIV and hepatitis B and C. PEP should ideally be initiated as soon as possible after the exposure, as the timing of initiation can impact its effectiveness. After seeking PEP, further steps such as testing the patient for HIV and following up with occupational health can also be important, but prompt initiation of PEP is a critical priority. |
|  |  | Attempt 3  The most important first action after a needle-stick injury is:  C. Seek post-exposure prophylaxis.  When a healthcare worker sustains a needle-stick injury, it is crucial to promptly seek post-exposure prophylaxis (PEP) to minimize the risk of transmission of bloodborne infections. This is especially important in cases involving patients with a history of intravenous drug use, as they may have an increased risk of bloodborne infections such as HIV and hepatitis B or C.  Seeking PEP involves contacting the appropriate healthcare personnel, such as occupational health or infection control, to initiate the necessary steps for evaluating the risk, assessing the need for prophylactic treatment, and providing appropriate guidance and follow-up. PEP typically involves a combination of antiretroviral medications for HIV prevention, and in some cases, hepatitis B vaccination or immunoglobulin administration.  While other actions, such as encouraging bleeding from the wound (A), making an appointment with occupational health (B), testing the patient for HIV (D), or washing the wound with alcohol (E), may be relevant steps in the overall management, seeking post-exposure prophylaxis is the most critical initial action to minimize the risk of potential bloodborne infection transmission. |
| **73** | A 32 year old woman has 3 weeks of fever, rigors and lethargy. In the past week, she has also become breathless on exertion. She is an intravenous drug user. Her temperature is 38°C, pulse rate 100 bpm regular, and BP 100/60 mmHg. Her JVP is raised with predominant V waves. There is a pansystolic murmur at the left sternal edge on inspiration. She has reduced air entry with dullness to percussion at the right lung base. She has swelling of both ankles.  Which is the most likely pathogen?  A. Enterococcus faecalis  B. Staphylococcus aureus  C. Staphylococcus epidermidis  D. Streptococcus bovis / streptococcus equinus complex  E. Streptococcus viridans | Attempt 1  Given the clinical presentation of fever, rigors, lethargy, intravenous drug use, breathlessness on exertion, raised JVP with predominant V waves, pansystolic murmur at the left sternal edge on inspiration, reduced air entry with dullness to percussion at the right lung base, and ankle swelling, the most likely pathogen in this case is:  B. Staphylococcus aureus  Staphylococcus aureus is a common pathogen associated with infections in intravenous drug users. It can cause a range of infections, including endocarditis (infection of the heart valves), which would explain the presence of the pansystolic murmur at the left sternal edge. Additionally, Staphylococcus aureus can lead to septic emboli, which can cause pulmonary infiltrates and lead to reduced air entry with dullness to percussion at the lung base. The presence of ankle swelling suggests the possibility of congestive heart failure, which can be a complication of infective endocarditis.  While other bacteria, such as Streptococcus species, can also cause infective endocarditis, the presentation in this case is more consistent with Staphylococcus aureus. Enterococcus faecalis, Staphylococcus epidermidis, and Streptococcus viridans are less commonly associated with infections in intravenous drug users compared to Staphylococcus aureus. Streptococcus bovis and Streptococcus equinus complex are more commonly associated with endocarditis in patients with underlying gastrointestinal pathology, such as colorectal cancer, and are less likely in this case.  It is important to note that this is a simulated scenario, and a definitive diagnosis would require appropriate clinical evaluation, laboratory investigations, and possibly blood cultures to identify the causative pathogen. |
|  |  | Attempt 2  B. Staphylococcus aureus  Given the patient's history of intravenous drug use, fever, rigors, breathlessness, and signs of right-sided heart failure (raised JVP with predominant V waves, ankle swelling), the most likely pathogen causing infective endocarditis in this case is Staphylococcus aureus. Intravenous drug use is a risk factor for right-sided infective endocarditis, and Staphylococcus aureus is a common causative organism in these cases. The presence of a pansystolic murmur at the left sternal edge on inspiration (Tricuspid regurgitation murmur) further supports the suspicion of right-sided endocarditis. |
|  |  | Attempt 3  The most likely pathogen in this scenario is:  B. Staphylococcus aureus.  The patient's history of intravenous drug use, fever, rigors, lethargy, breathlessness on exertion, raised JVP with predominant V waves, and pansystolic murmur at the left sternal edge on inspiration suggests infective endocarditis, a serious infection of the heart valves. In intravenous drug users, Staphylococcus aureus is the most common pathogen associated with infective endocarditis.  Staphylococcus aureus is a Gram-positive bacterium that can cause a wide range of infections. In intravenous drug users, it is frequently associated with endocarditis due to the direct inoculation of bacteria into the bloodstream through contaminated needles. Staphylococcus aureus endocarditis can lead to systemic symptoms, cardiac complications, and septic emboli to various organs.  While other organisms like Enterococcus faecalis (A), Staphylococcus epidermidis (C), Streptococcus bovis / streptococcus equinus complex (D), and Streptococcus viridans (E) can also cause infective endocarditis, Staphylococcus aureus is the most likely pathogen in this case, given the patient's history of intravenous drug use and the clinical findings described. |
| **74** | A 34 year old woman has sudden onset of right arm weakness and inability to speak. She has a history of migraine and generalised joint pains. Four years ago, she had a deep vein thrombosis in her right leg. Her pulse rate is 68 bpm and BP 178/94 mmHg. She has an expressive dysphasia. She has flaccid weakness of her right arm and facial droop on the right lower half of her face. Investigations: Haemoglobin 118 g/L (115–150) White cell count 4.3 × 109/L (3.8–10.0) Neutrophils 2.1 × 109/L (2.0–7.5) Lymphocytes 0.6 × 109/L (1.1–3.3) Platelets 132 × 109/L (150–400) Total cholesterol 4.6 mmol/L (<5.0) CT scan of head left frontoparietal infarct  Which additional investigation is most likely to reveal the underlying cause of her stroke?  A. Anti-dsDNA antibody  B. Anticardiolipin antibody  C. Anti-Ro antibody  D. Rheumatoid factor  E. Serum immunoglobulins | Attempt 1  Given the clinical presentation of sudden onset of right arm weakness, inability to speak (expressive dysphasia), history of migraine and generalised joint pains, deep vein thrombosis in the past, and the findings on investigations including the left frontoparietal infarct on CT scan, the most likely underlying cause of her stroke is:  B. Anticardiolipin antibody  Anticardiolipin antibodies are associated with an increased risk of thrombotic events, including stroke. In the context of her history of deep vein thrombosis, the presence of anticardiolipin antibodies becomes more significant. Anticardiolipin antibodies are a type of antiphospholipid antibody, and the presence of these antibodies is associated with antiphospholipid syndrome (APS). APS is an autoimmune disorder characterized by recurrent arterial or venous thrombosis and/or pregnancy complications. The presence of a frontoparietal infarct in this patient is consistent with an arterial thrombotic event.  While the other antibodies listed (anti-dsDNA, anti-Ro, rheumatoid factor) are associated with autoimmune diseases, they are not specifically associated with an increased risk of thrombotic events or stroke. Serum immunoglobulins are non-specific and unlikely to reveal the underlying cause in this case.  It's important to note that this is a simulated scenario, and further investigations and evaluation, including repeat testing for anticardiolipin antibodies and other diagnostic tests, would be necessary to confirm the diagnosis of antiphospholipid syndrome and determine the underlying cause of the stroke. |
|  |  | Attempt 2  B. Anticardiolipin antibody  Given the patient's history of deep vein thrombosis, migraine, joint pains, and the presence of a left frontoparietal infarct on CT scan, there is a suspicion of an underlying hypercoagulable state. Antiphospholipid syndrome (APS), characterized by the presence of antiphospholipid antibodies such as anticardiolipin antibodies and lupus anticoagulant, is associated with an increased risk of both arterial and venous thrombosis. This condition can present with various clinical manifestations, including stroke. Performing the anticardiolipin antibody test can help identify if APS is contributing to the patient's thrombotic events. |
|  |  | Attempt 3  The most appropriate additional investigation to reveal the underlying cause of her stroke in this case is:  B. Anticardiolipin antibody.  The patient's clinical presentation of sudden-onset right arm weakness, inability to speak (expressive dysphasia), facial droop, and history of deep vein thrombosis suggests an ischemic stroke, likely due to an embolism. Given her medical history of migraine, joint pains, and previous deep vein thrombosis, there is suspicion of an underlying hypercoagulable state.  Anticardiolipin antibodies are commonly associated with antiphospholipid syndrome (APS), an autoimmune disorder characterized by the presence of antiphospholipid antibodies and a predisposition to thrombotic events. These antibodies, including anticardiolipin antibodies, can cause an increased risk of arterial and venous thrombosis.  Testing for anticardiolipin antibodies is recommended in patients with unexplained arterial or venous thrombosis, particularly in the presence of other suggestive clinical features. In this case, the patient's history of deep vein thrombosis and the occurrence of an ischemic stroke warrant investigation for antiphospholipid antibodies, specifically anticardiolipin antibodies.  While other autoimmune markers such as anti-dsDNA antibody (A), anti-Ro antibody (C), and rheumatoid factor (D) may be relevant in certain clinical contexts, the most appropriate investigation to explore the underlying cause of her stroke, given the clinical presentation and history, is testing for anticardiolipin antibodies (B). Serum immunoglobulins (E) are less likely to be directly related to the underlying cause of the stroke in this case. |
| **75** | A 55 year old woman has been feeling tired and sleepy. Her partner says that she snores heavily. She has a history of type 2 diabetes mellitus and takes metformin. Her BMI is 38 kg/m2. Her oxygen saturation is 95% breathing air. Her Epworth sleepiness score is 19 (normal <11). Her HbA1cis 60 mmol/mol (20-42).  Which treatment is most likely to improve her daytime somnolence?  A. Bariatric surgery  B. Continuous positive airway pressure ventilation  C. Long acting insulin  D. Mandibular advancement device  E. Modafinil | Attempt 1  Given the patient's symptoms of excessive daytime sleepiness, heavy snoring, and high Epworth sleepiness score, along with a history of obesity and type 2 diabetes mellitus, the most likely underlying cause of her symptoms is obstructive sleep apnea (OSA). OSA is characterized by repetitive episodes of partial or complete upper airway obstruction during sleep, leading to disrupted sleep patterns and daytime somnolence.  The treatment that is most likely to improve her daytime somnolence in this case is:  B. Continuous positive airway pressure (CPAP) ventilation  CPAP is the first-line treatment for moderate to severe OSA. It involves wearing a mask over the nose and/or mouth during sleep, which delivers a continuous flow of pressurized air to keep the upper airway open. By maintaining airway patency, CPAP effectively reduces or eliminates episodes of apnea and hypopnea, improving sleep quality and alleviating daytime sleepiness.  While bariatric surgery may be beneficial for weight loss and could potentially improve sleep apnea symptoms, it is not the initial treatment of choice in this scenario. Long-acting insulin and modafinil do not directly address the underlying issue of upper airway obstruction in OSA. Mandibular advancement devices are an alternative treatment option for patients with mild to moderate OSA who cannot tolerate or are unwilling to use CPAP.  It's important to note that the final treatment decision should be made in consultation with a sleep specialist or healthcare provider experienced in the management of sleep disorders. They will consider the patient's individual circumstances, severity of sleep apnea, and preferences when determining the most appropriate treatment approach. |
|  |  | Attempt 2  B. Continuous positive airway pressure ventilation  Given the patient's excessive daytime sleepiness, heavy snoring, obesity, and high Epworth sleepiness score, the most appropriate treatment to improve her daytime somnolence is continuous positive airway pressure (CPAP) ventilation. This is a common treatment for obstructive sleep apnea (OSA), a condition often seen in individuals with obesity and characterized by repeated episodes of partial or complete upper airway collapse during sleep, leading to disrupted sleep and daytime sleepiness. CPAP helps keep the airway open by delivering a constant stream of air pressure, preventing the collapse of the airway and improving sleep quality. |
|  |  | Attempt 3  The most appropriate treatment to improve her daytime somnolence in this case is:  B. Continuous positive airway pressure (CPAP) ventilation.  The patient's symptoms, including excessive daytime sleepiness, heavy snoring, and obesity, are suggestive of obstructive sleep apnea (OSA). OSA is a sleep disorder characterized by repetitive episodes of partial or complete upper airway obstruction during sleep, leading to disrupted sleep and daytime somnolence.  Continuous positive airway pressure (CPAP) ventilation is the first-line treatment for moderate to severe OSA. It involves wearing a mask over the nose and/or mouth during sleep, which delivers a steady flow of pressurized air to keep the airway open and prevent obstruction. CPAP improves sleep quality, reduces daytime sleepiness, and alleviates snoring.  While bariatric surgery (A) may be beneficial for weight loss and overall health, its primary purpose is not specifically targeted at treating OSA. Long-acting insulin (C) is used for glycemic control in diabetes and would not directly address the underlying cause of the patient's symptoms. Mandibular advancement devices (D) are an alternative treatment option for mild to moderate OSA but are less effective than CPAP for severe cases. Modafinil (E) is a wakefulness-promoting medication that can temporarily alleviate sleepiness but does not address the underlying airway obstruction.  Therefore, the most appropriate treatment to improve her daytime somnolence and manage her suspected OSA is continuous positive airway pressure (CPAP) ventilation (B). It is important for the patient to undergo a sleep study and consult with a sleep specialist to determine the optimal pressure settings and ensure proper use of the CPAP equipment. |
| **76** | A 72 year old man has had six months of increased frequency of defaecation and three months of dark red rectal bleeding mixed with the stool. Investigations: Haemoglobin 101 g/L (130–175) Mean cell haemoglobin (MCH) 24 pg (27–33) MCV 73 fL (80–96) White cell count 9.1 x 109/L (3.0–10.0) Platelets 354 x 109/L (150–400)  Which is the most likely diagnosis?  A. Colonic carcinoma  B. Diverticular disease  C. Haemorrhoids  D. Ischaemic colitis  E. Ulcerative colitis | Attempt 1  In a 72-year-old man presenting with increased frequency of defecation and dark red rectal bleeding mixed with stool, the most likely diagnosis is:  A. Colonic carcinoma  The combination of symptoms, particularly the persistent rectal bleeding and changes in bowel habits, along with the laboratory findings of anemia (low hemoglobin) and microcytosis (low MCV), raises concern for colonic carcinoma. Colorectal cancer often presents with these symptoms, especially in the older population. Other potential causes such as diverticular disease, hemorrhoids, ischemic colitis, and ulcerative colitis may also cause rectal bleeding, but they are less likely to present with a sustained change in bowel habits.  Further diagnostic evaluation, such as colonoscopy or sigmoidoscopy, is necessary to confirm the diagnosis and assess the extent of the colonic involvement. Tissue biopsy during endoscopy can provide a definitive diagnosis of colonic carcinoma and determine the stage of the disease.  It's important for the patient to be promptly referred to a gastroenterologist or colorectal surgeon for further evaluation and management. Early detection and treatment of colorectal cancer improve outcomes and prognosis. |
|  |  | Attempt 2  A. Colonic carcinoma  The combination of increased frequency of defecation, dark red rectal bleeding mixed with stool, and low hemoglobin levels suggests the possibility of colonic carcinoma. The anemia (low hemoglobin) and microcytosis (low MCV) seen in this case could be due to chronic blood loss from the tumor. It's important to consider colorectal cancer as a potential cause of rectal bleeding, especially in older individuals, and further investigations like colonoscopy or imaging may be needed to confirm the diagnosis. |
|  |  | Attempt 3  The most likely diagnosis in this case is:  A. Colonic carcinoma.  The patient's symptoms of increased frequency of defecation and dark red rectal bleeding, along with the laboratory findings of low hemoglobin (anemia) and microcytic, hypochromic red blood cells (low MCV and MCH), are concerning for colonic carcinoma. Colorectal cancer often presents with changes in bowel habits, rectal bleeding, and anemia.  While diverticular disease (B) and hemorrhoids (C) can cause rectal bleeding, they are less likely to be associated with increased frequency of defecation and significant anemia. Ischemic colitis (D) typically presents with abdominal pain, bloody diarrhea, and a history of vascular disease, which are not described in this case. Ulcerative colitis (E) is a chronic inflammatory bowel disease that can cause rectal bleeding and increased frequency of bowel movements, but it usually presents at a younger age, and the anemia seen in this case is more suggestive of a neoplastic process.  Given the age of the patient, the presence of rectal bleeding, and the laboratory findings, colonic carcinoma is the most likely diagnosis. Further evaluation with colonoscopy and imaging studies would be necessary to confirm the diagnosis and determine the extent of the disease. |
| **77** | A 24 year old man is reviewed following a recent orchidectomy. The pathologist's report describes a mass with cystic spaces. Histological examination shows areas of mature cartilage and columnar epithelium.  Which is the most likely diagnosis?  A. Chondrosarcoma  B. Hamartoma  C. Lymphoma  D. Seminoma  E. Teratoma | Attempt 1  The most likely diagnosis based on the description of the pathology report is:  E. Teratoma  A teratoma is a type of germ cell tumor that can occur in the testes. It is composed of tissues derived from multiple germ cell layers, including ectoderm, mesoderm, and endoderm. Teratomas can contain a wide range of tissue types, including mature cartilage and epithelial tissues.  In this case, the presence of cystic spaces, mature cartilage, and columnar epithelium is consistent with a teratoma. The histological findings suggest the presence of different tissue types within the tumor, which is characteristic of a teratoma.  It is important to note that further investigation and evaluation may be necessary to determine the exact nature of the teratoma and to guide appropriate management and follow-up. This typically involves imaging studies and additional laboratory tests. A multidisciplinary approach involving urologists, oncologists, and pathologists is often taken to ensure proper diagnosis and management of testicular teratomas. |
|  |  | Attempt 2  E. Teratoma  The presence of cystic spaces, mature cartilage, and columnar epithelium in the testicular mass points toward the diagnosis of a teratoma. Teratomas are germ cell tumors that can contain tissues from all three germ layers (ectoderm, mesoderm, and endoderm). The presence of cartilage and other differentiated tissues in the mass supports the diagnosis of a teratoma. |
|  |  | Attempt 3  The most likely diagnosis in this case is:  E. Teratoma.  The presence of cystic spaces, mature cartilage, and columnar epithelium in the histological examination suggests a teratoma. Teratomas are germ cell tumors that can contain elements from all three germ cell layers (ectoderm, mesoderm, and endoderm). These tumors can occur in the testes (testicular teratoma) and commonly present as a painless testicular mass.  Chondrosarcoma (A) is a malignant tumor composed of cartilage-forming cells and is less likely given the benign nature of the histological findings in this case.  Hamartoma (B) is a benign tumor-like malformation composed of mature tissues normally found in the affected organ. However, the presence of cartilage and columnar epithelium suggests a germ cell origin rather than a hamartomatous lesion.  Lymphoma (C) typically presents as a solid mass and does not usually contain cartilage or columnar epithelium.  Seminoma (D) is a type of germ cell tumor that typically consists of undifferentiated germ cells with uniform, rounded nuclei. It does not typically contain cartilage or columnar epithelium.  Given the histological findings described, a teratoma is the most likely diagnosis in this case. Further evaluation and staging may be required to determine the extent and potential malignancy of the tumor. |
| **78** | A 50 year old man has had increasing breathlessness when climbing the stairs. There is no chest pain, wheeze or cough. He has COPD. He takes a salbutamol inhaler several times per day. He is an ex-smoker of 3 months and has a 30 pack-year smoking history. His weight is stable and he is otherwise well. Investigation: FEV1 : 75% predicted  Which is the most appropriate next step in pharmacological management?  A. Inhaled beclometasone  B. Inhaled tiotropium and salmeterol  C. Nebulised salbultamol and ipratropium bromide  D. Oral prednisolone  E. Oral theophylline | Attempt 1  The most appropriate next step in pharmacological management for this patient with increasing breathlessness and COPD would be:  B. Inhaled tiotropium and salmeterol  Inhaled tiotropium and salmeterol are long-acting bronchodilators commonly used in the management of COPD. Tiotropium is an anticholinergic medication that helps to relax the airway smooth muscles, while salmeterol is a long-acting beta-agonist that helps to open up the airways. This combination therapy can improve lung function, reduce breathlessness, and enhance exercise tolerance in patients with COPD.  The patient's FEV1 (forced expiratory volume in 1 second) is 75% predicted, indicating moderate airflow limitation. Inhaled bronchodilators, such as tiotropium and salmeterol, are recommended as first-line treatment for patients with moderate to severe COPD. These medications provide sustained bronchodilation and are more effective in managing symptoms compared to short-acting bronchodilators alone.  It's important to note that pharmacological management should be individualized based on the patient's symptoms, lung function, and response to treatment. Regular follow-up with healthcare providers is essential to assess the patient's response to therapy and make any necessary adjustments. |
|  |  | Attempt 2  Given the provided information and the patient's clinical situation, the most appropriate next step in pharmacological management for this individual with COPD and increasing breathlessness when climbing stairs is:  B. Inhaled tiotropium and salmeterol  Explanation:  The patient's presentation is consistent with COPD, and his FEV1 of 75% predicted indicates moderate airflow limitation. He is an ex-smoker with a significant smoking history and is currently experiencing exertional breathlessness. Inhaled tiotropium and salmeterol are both bronchodilators that are commonly used as initial maintenance treatment in patients with moderate to severe COPD.  Tiotropium is a long-acting anticholinergic (LAMA) bronchodilator, while salmeterol is a long-acting beta-agonist (LABA) bronchodilator. Combining these two classes of bronchodilators can provide additive bronchodilation, leading to improved lung function and decreased breathlessness. This combination can also help enhance exercise tolerance and quality of life in patients with COPD.  Inhaled corticosteroids (option A) are generally reserved for patients with more severe COPD, particularly if they have frequent exacerbations. Nebulized salbutamol and ipratropium bromide (option C) might be used in acute exacerbations, but they are not the first-line choice for stable maintenance therapy. Oral prednisolone (option D) is not typically used as long-term management for stable COPD. Oral theophylline (option E) is not commonly used as a first-line treatment due to its potential for interactions and side effects.  However, the choice of treatment should be discussed and tailored to the individual patient's needs and preferences in consultation with a healthcare professional. |
|  |  | Attempt 3  In this case, the most appropriate next step in pharmacological management for a patient with COPD and increasing breathlessness is:  B. Inhaled tiotropium and salmeterol.  The patient's symptoms of increasing breathlessness suggest inadequate control of his COPD despite the use of a salbutamol inhaler. Inhaled corticosteroids (option A) are typically reserved for patients with frequent exacerbations and a history of asthma-COPD overlap. As the patient does not have symptoms of wheeze or cough, initiating inhaled corticosteroids may not provide additional benefit.  Option C, nebulized salbutamol and ipratropium bromide, may be considered for acute exacerbations or as a rescue therapy, but it is not the most appropriate next step for long-term management of the patient's symptoms.  Option D, oral prednisolone, is generally reserved for exacerbations and not recommended for routine maintenance therapy in stable COPD patients.  Option E, oral theophylline, is not the most appropriate next step as it is a less commonly used medication in the management of COPD, especially in patients who are already using inhaled bronchodilators.  The combination of inhaled tiotropium (a long-acting anticholinergic) and salmeterol (a long-acting beta2-agonist) has been shown to improve lung function, reduce symptoms, and decrease exacerbations in patients with COPD. This combination therapy is often considered as a step-up treatment when short-acting bronchodilators alone are insufficient to control symptoms. Therefore, initiating inhaled tiotropium and salmeterol would be the most appropriate next step in this patient's pharmacological management. |
| **79** | An 86 year old woman has had three falls in the past 3 months. On each occasion, she describes feeling lightheaded and dizzy prior to falling. She is taking alendronic acid, amlodipine, atorvastatin, metformin and zolpidem tartrate. Her BP is 132/80 mmHg sitting and 138/84 mmHg standing.  Which medication is most likely to be contributing to her falls?  A. Alendronic acid  B. Amlodipine  C. Atorvastatin  D. Metformin  E. Zolpidem tartrate | Attempt 1  In the case of an 86-year-old woman who has experienced three falls in the past 3 months, with a complaint of feeling lightheaded and dizzy prior to falling, the medication most likely to be contributing to her falls is:  B. Amlodipine.  Amlodipine is a calcium channel blocker commonly used to treat hypertension. One of the potential side effects of amlodipine is orthostatic hypotension, which is characterized by a drop in blood pressure upon standing. Symptoms of orthostatic hypotension include lightheadedness and dizziness, which can increase the risk of falls, particularly in older individuals.  Alendronic acid (option A) is a bisphosphonate used for the treatment of osteoporosis. While it can cause gastrointestinal side effects, it is less likely to directly contribute to falls.  Atorvastatin (option C) is a statin used to manage cholesterol levels. Falls are not a commonly reported side effect of statins, although muscle-related adverse effects can occur.  Metformin (option D) is an oral medication commonly prescribed for type 2 diabetes. While it can cause gastrointestinal side effects, it is not typically associated with falls.  Zolpidem tartrate (option E) is a sedative-hypnotic used for the short-term treatment of insomnia. While it can cause drowsiness, it is not directly associated with falls unless it leads to impaired coordination or balance.  Considering the patient's symptoms of feeling lightheaded and dizzy prior to falling and the potential for amlodipine to cause orthostatic hypotension, it is the most likely medication contributing to her falls. A medication review and adjustment may be necessary to improve her symptoms and reduce the risk of falls. Consulting with a healthcare professional is recommended for further evaluation and management. |
|  |  | Attempt 2  The medication that is most likely to be contributing to the falls in this 86-year-old woman is:  E. Zolpidem tartrate  Explanation:  The patient is experiencing falls associated with feeling lightheaded and dizzy prior to falling. This suggests that her falls may be related to a drop in blood pressure upon standing, which is known as orthostatic hypotension. Orthostatic hypotension can lead to dizziness and lightheadedness, increasing the risk of falls.  Among the medications listed, zolpidem tartrate stands out as a potential contributor to orthostatic hypotension. Zolpidem is a sedative-hypnotic medication commonly used for insomnia. One of its side effects is dizziness, and it can also cause orthostatic hypotension in some individuals, especially older adults. The combination of zolpidem with other medications and factors commonly seen in elderly patients can increase the risk of falls.  While the other medications listed (alendronic acid, amlodipine, atorvastatin, and metformin) have potential side effects, they are less likely to directly cause orthostatic hypotension, which seems to be the primary issue in this patient's case.  It's important for the patient to consult with her healthcare provider to discuss her symptoms, medication regimen, and potential adjustments to her medications to address the falls and minimize the risk of further incidents. |
|  |  | Attempt 3  The medication that is most likely to be contributing to the falls in this 86-year-old woman is:  E. Zolpidem tartrate  Zolpidem tartrate is a sedative-hypnotic medication commonly used for the short-term treatment of insomnia. One of the side effects of zolpidem is dizziness, which can increase the risk of falls, especially in the elderly population. The feeling of lightheadedness and dizziness that the woman describes prior to falling may be attributed to the use of zolpidem.  It's important to assess the overall benefit-risk balance of zolpidem in older adults, particularly those who are prone to falls. In many cases, it is advisable to consider alternative non-pharmacological interventions for insomnia or adjust the dosage or timing of medications to minimize the risk of falls. It would be appropriate to consult with a healthcare professional, such as a doctor or pharmacist, to evaluate the necessity and appropriateness of zolpidem in this particular case and discuss potential alternatives or adjustments to the medication regimen. |
| **80** | A 81 year old man has three months of malaise, bleeding gums and pain in his legs. He has been struggling to look after himself since his wife died one year ago. He has a poor diet and reports that he mostly has tea and toast. He has gingival hypertrophy and skin petechiae.  Which micronutrient deficiency is he most likely to have?  A. Magnesium  B. Vitamin A  C. Vitamin B 1  D. Vitamin C  E. Zinc | Attempt 1  The micronutrient deficiency that the 81-year-old man is most likely to have based on his symptoms is:  D. Vitamin C  The presence of bleeding gums, petechiae (small red or purple spots on the skin), and malaise suggests a deficiency of vitamin C, also known as scurvy. Vitamin C is essential for collagen synthesis, which is important for maintaining the integrity of blood vessels, gums, and connective tissues.  The poor diet described by the patient, consisting mostly of tea and toast, is likely lacking in fresh fruits and vegetables, which are the main sources of vitamin C. Inadequate intake of vitamin C over a prolonged period can lead to the development of scurvy.  Treatment for vitamin C deficiency involves increasing dietary intake of vitamin C-rich foods such as citrus fruits, berries, peppers, and leafy greens. In severe cases, oral or intravenous vitamin C supplementation may be required.  It is important for the patient to receive a comprehensive evaluation of his nutritional status and overall health, as well as appropriate support and assistance for his struggles in self-care following the loss of his wife. |
|  |  | Attempt 2  Based on the presented symptoms and clinical findings, the 81-year-old man is most likely suffering from:  D. Vitamin C deficiency  Explanation:  The patient is experiencing bleeding gums, skin petechiae (small red or purple spots on the skin caused by bleeding underneath), and leg pain. These symptoms, along with his history of a poor diet and tea and toast intake, are indicative of scurvy, a condition caused by a deficiency of vitamin C (ascorbic acid).  Vitamin C is essential for collagen synthesis, which is important for maintaining the integrity of blood vessels, connective tissues, and skin. Deficiency of vitamin C can lead to weakened blood vessels, resulting in bleeding gums, petechiae, and increased susceptibility to bruising. Leg pain can also be associated with the connective tissue changes that occur due to vitamin C deficiency.  The other listed micronutrients (magnesium, vitamin A, vitamin B1, and zinc) are not typically associated with the specific constellation of symptoms described in this patient. Magnesium deficiency might present with neuromuscular symptoms, vitamin A deficiency with vision problems and skin changes, vitamin B1 deficiency with neurological issues, and zinc deficiency with a variety of symptoms including skin changes and impaired wound healing.  Given the clinical picture, it's important for the patient to receive appropriate medical evaluation and treatment, which would likely involve correcting the vitamin C deficiency through dietary adjustments and supplementation as needed. |
|  |  | Attempt 3  Based on the symptoms described, the most likely micronutrient deficiency in this 81-year-old man is:  D. Vitamin C deficiency.  The symptoms of malaise, bleeding gums, and skin petechiae are consistent with the clinical presentation of scurvy, which is caused by a deficiency of vitamin C. Vitamin C plays a crucial role in collagen synthesis, and its deficiency can lead to weakened blood vessels and impaired wound healing, resulting in symptoms such as bleeding gums and skin bruising.  The patient's poor diet, primarily consisting of tea and toast, suggests a lack of fresh fruits and vegetables, which are the primary sources of vitamin C. Additionally, his recent life stressors and struggles with self-care may have contributed to a neglect of adequate nutrition.  Magnesium deficiency (option A) can cause various symptoms, including muscle weakness and cramps, but it is less likely to present with bleeding gums and skin petechiae.  Vitamin A deficiency (option B) can lead to vision problems and dry skin but is not typically associated with gingival hypertrophy or petechiae.  Thiamine (vitamin B1) deficiency (option C) can result in symptoms such as fatigue, weakness, and peripheral neuropathy but is not commonly associated with bleeding gums or skin petechiae.  Zinc deficiency (option E) can cause various symptoms, including skin changes and impaired wound healing, but it is less likely to manifest with gingival hypertrophy and petechiae.  Given the patient's symptoms and the characteristic signs of gingival hypertrophy and skin petechiae, a vitamin C deficiency (scurvy) is the most likely micronutrient deficiency. Proper dietary intervention and vitamin C supplementation would be indicated in the management of this condition. Consulting with a healthcare professional is recommended for further evaluation and treatment. |
| **81** | A 45 year old man has had 6 months of tiredness, reduced libido and erectile dysfunction. Investigations: Testosterone 1.8 nmol/L (9.9–27.8) LH 1.2 U/L (1–8) FSH 1.0 U/L (1–12)  Which is the most likely cause of his presentation?  A. Anabolic steroid misuse  B. Androgen insensitivity syndrome  C. Congenital adrenal hyperplasia  D. Klinefelter's syndrome  E. Pituitary adenoma | Attempt 1  Based on the presented laboratory results and symptoms, the most likely cause of the man's presentation is:  D. Klinefelter's syndrome.  Klinefelter's syndrome is a chromosomal disorder that occurs in males, typically resulting from an extra X chromosome (47,XXY) instead of the usual XY configuration. This syndrome is associated with various clinical manifestations, including decreased testosterone production. The laboratory findings of low testosterone levels (1.8 nmol/L) along with low levels of luteinizing hormone (LH) and follicle-stimulating hormone (FSH) are consistent with the hormonal profile seen in Klinefelter's syndrome.  Anabolic steroid misuse (option A) typically leads to high levels of testosterone, which would be inconsistent with the low testosterone levels observed in this case.  Androgen insensitivity syndrome (option B) is a condition in which individuals with XY chromosomes are partially or completely insensitive to the effects of androgens. This syndrome is not typically associated with reduced testosterone levels.  Congenital adrenal hyperplasia (option C) is a group of inherited disorders affecting the adrenal glands and can result in various hormonal imbalances. However, the presented symptoms and laboratory results are not indicative of congenital adrenal hyperplasia.  Pituitary adenoma (option E) is a tumor of the pituitary gland that can affect hormone production, including gonadotropins (LH and FSH). However, pituitary adenomas are less likely to cause primary testicular dysfunction leading to low testosterone levels.  Considering the clinical picture, including the symptoms of tiredness, reduced libido, and erectile dysfunction, along with the laboratory findings of low testosterone, LH, and FSH levels, Klinefelter's syndrome is the most likely cause. Further evaluation and genetic testing can be conducted to confirm the diagnosis. Referral to an endocrinologist or a specialist in genetic disorders is recommended for appropriate management and counseling. |
|  |  | Attempt 2  The most likely cause of the 45-year-old man's presentation based on the provided information is:  E. Pituitary adenoma  Explanation:  The patient's symptoms of tiredness, reduced libido, and erectile dysfunction, along with the laboratory findings of low testosterone (hypogonadism) and low LH and FSH levels, suggest a potential issue with the hypothalamic-pituitary-gonadal (HPG) axis. The HPG axis controls the production of testosterone and other sex hormones.  In this case, the low levels of LH and FSH indicate a possible dysfunction at the level of the pituitary gland. A pituitary adenoma (a tumor of the pituitary gland) can disrupt the normal secretion of hormones, including LH and FSH. This disruption can lead to decreased testosterone production, resulting in the symptoms of fatigue, reduced libido, and erectile dysfunction.  Options A (anabolic steroid misuse), B (androgen insensitivity syndrome), C (congenital adrenal hyperplasia), and D (Klinefelter's syndrome) are less likely to explain the combination of symptoms and hormone levels seen in this patient. Anabolic steroid misuse might affect hormone levels but would be more likely to result in elevated testosterone levels. Androgen insensitivity syndrome, congenital adrenal hyperplasia, and Klinefelter's syndrome are conditions that can affect hormone levels but would not typically present with a pituitary-related disruption of LH and FSH.  A pituitary adenoma should be investigated and managed by a healthcare professional, and further diagnostic tests (such as imaging and additional hormonal evaluations) may be necessary to confirm the diagnosis and determine the appropriate treatment approach. |
|  |  | Attempt 3  The most likely cause of the 45-year-old man's presentation, based on the laboratory results, is:  E. Pituitary adenoma  The low testosterone level (1.8 nmol/L) and low levels of luteinizing hormone (LH) and follicle-stimulating hormone (FSH) suggest a primary hypogonadism, which is commonly caused by testicular dysfunction. However, in this case, the LH and FSH levels are also low, indicating a problem at the hypothalamic-pituitary level.  A pituitary adenoma, specifically a non-functioning adenoma, can interfere with the normal secretion of LH and FSH, leading to decreased testosterone production. This can result in symptoms such as fatigue, reduced libido, and erectile dysfunction.  Other causes listed in the options (A, B, C, D) are less likely based on the given information. Anabolic steroid misuse would typically result in supraphysiological levels of testosterone. Androgen insensitivity syndrome, congenital adrenal hyperplasia, and Klinefelter's syndrome would usually present with different clinical features and hormone profile abnormalities.  Therefore, a pituitary adenoma is the most likely cause of the patient's presentation. Further evaluation, such as imaging studies (e.g., MRI), would be necessary to confirm the diagnosis and determine the appropriate management. |
| **82** | A 24 year old man is admitted to hospital with an exacerbation of asthma. His symptoms improve with treatment, and he is ready for discharge after 24 hours. His discharge medication includes a salbutamol inhaler, a combined beclometasone and salmeterol inhaler, and a short course of oral prednisolone.  Which further management must be provided prior to discharge?  A. Antibiotic rescue pack  B. Course of antihistamines  C. Nebuliser machine for use at home  D. Personalised asthma action plan  E. Volumatic spacer device | Attempt 1  The further management that must be provided prior to discharge for the 24-year-old man with an exacerbation of asthma is:  D. Personalised asthma action plan  A personalized asthma action plan is an essential component of asthma management. It provides guidance to the patient on how to monitor their symptoms, adjust their medications, and recognize when to seek medical help. The action plan outlines specific steps based on the individual's symptoms and peak flow measurements, helping them understand when to increase or decrease medication dosages and when to seek emergency care.  Having a personalized asthma action plan empowers the patient to actively manage their condition, improve self-care, and reduce the risk of future exacerbations. It also helps them understand when to adjust their medication regimen based on their symptoms, ensuring appropriate and timely treatment.  While other options like an antibiotic rescue pack, antihistamines, nebulizer machine, and volumatic spacer device may have their roles in certain situations, a personalized asthma action plan is the most important management strategy to provide prior to discharge in this case. |
|  |  | Attempt 2  D. Personalised asthma action plan  Explanation:  A personalised asthma action plan is crucial for patients with asthma, especially after being admitted for an exacerbation. This plan provides clear instructions on how to manage asthma symptoms, what medications to take, and when to seek medical attention. It outlines specific steps to take based on the individual's symptoms and peak flow measurements, helping the patient recognize when their asthma is worsening and when to adjust their medication regimen.  An asthma action plan typically includes "green zone" (well-controlled), "yellow zone" (worsening), and "red zone" (severe exacerbation) instructions. It also guides the patient on adjusting their medications based on their symptoms and peak flow readings. Providing a personalised asthma action plan empowers the patient to take an active role in managing their asthma and can help prevent future exacerbations and hospital admissions.  While options A (antibiotic rescue pack), B (course of antihistamines), C (nebuliser machine for use at home), and E (Volumatic spacer device) might have their own merits in certain situations, they are not as critical as providing the patient with a clear and tailored asthma action plan before discharge. |
|  |  | Attempt 3  The most appropriate further management that must be provided prior to discharge for this patient is:  D. Personalised asthma action plan.  A personalized asthma action plan is a written document that provides guidance to the patient on how to manage their asthma symptoms and adjust their medication based on their individualized treatment goals. It includes information on the patient's daily controller medications, rescue medications, peak flow monitoring (if applicable), and specific instructions on when and how to adjust their medication in response to changes in symptoms or peak flow readings.  Having a personalized asthma action plan is crucial in empowering the patient to self-manage their asthma effectively and recognize early signs of worsening symptoms. It helps them understand when to increase their medication, when to seek medical attention, and when to follow up with their healthcare provider. This promotes better asthma control and reduces the risk of exacerbations.  While the patient may have received appropriate medications for their asthma exacerbation, it is important to provide them with a personalized asthma action plan to enhance their understanding of the condition and optimize their self-management skills. This ensures that they have a clear plan to follow after discharge and can take appropriate actions to prevent future exacerbations. |
| **83** | A 40 year old man develops sudden breathlessness 5 days after an acute inferior ST-elevation myocardial infarction treated by primary coronary intervention. His pulse rate is 110 bpm, BP 110/75 mmHg, respiratory rate 22 breaths per minute and oxygen saturation 92% on breathing 28% oxygen. There is a pansystolic murmur at the apex and bibasal inspiratory crackles.  Which is the most likely cause of this presentation?  A. Acute pulmonary embolus  B. Aortic regurgitation  C. Cardiac tamponade  D. Papillary muscle rupture  E. Pericarditis | Attempt 1  Based on the provided information, the most likely cause of this presentation in a patient who developed sudden breathlessness 5 days after an acute inferior ST-elevation myocardial infarction is:  C. Cardiac tamponade.  The presence of a pansystolic murmur at the apex, bibasal inspiratory crackles, and signs of hemodynamic compromise (elevated heart rate, decreased blood pressure, and hypoxemia) are suggestive of cardiac tamponade. Cardiac tamponade occurs when fluid (such as blood) accumulates in the pericardial space, leading to compression of the heart and impaired cardiac filling. It can occur as a complication of myocardial infarction, particularly in the setting of rupture of the free wall or papillary muscles.  In this case, the patient's history of acute inferior ST-elevation myocardial infarction treated by primary coronary intervention raises suspicion for a potential cardiac complication. The development of sudden breathlessness along with the presence of a pansystolic murmur, which can be indicative of mitral regurgitation secondary to papillary muscle dysfunction, further supports the possibility of cardiac tamponade. The bibasal inspiratory crackles suggest the presence of pulmonary congestion, which can be seen in cardiac tamponade due to increased left ventricular filling pressures.  It is important to promptly diagnose and manage cardiac tamponade as it can be a life-threatening condition. Immediate interventions such as pericardiocentesis or surgical drainage may be necessary to relieve the tamponade and restore cardiac function. |
|  |  | Attempt 2  The most likely cause of this presentation is option D, papillary muscle rupture.  The patient has a history of an acute inferior ST-elevation myocardial infarction (STEMI) treated by primary coronary intervention. In the setting of an acute MI, papillary muscle rupture is a well-known complication, and it usually occurs within the first week after the MI.  The clinical features are suggestive of acute heart failure:  1. Sudden breathlessness: Caused by acute pulmonary edema due to the inability of the affected papillary muscle to adequately tether the mitral valve leaflets, resulting in mitral regurgitation.  2. Pansystolic murmur at the apex: The mitral regurgitation leads to a holosystolic (pansystolic) murmur at the apex, which is characteristic of significant mitral valve regurgitation.  3. Bibasal inspiratory crackles: These are typically heard in patients with pulmonary edema.  While acute pulmonary embolus (Option A) can present with sudden breathlessness and hypoxia, the presence of a new systolic murmur and the history of acute MI make papillary muscle rupture more likely in this case.  Aortic regurgitation (Option B) can cause a diastolic murmur, not a pansystolic murmur, and it is less likely given the clinical context.  Cardiac tamponade (Option C) can cause symptoms of low cardiac output, such as hypotension and pulsus paradoxus, which are not evident in this patient.  Pericarditis (Option E) typically presents with chest pain that worsens with inspiration and is relieved by sitting up and leaning forward. It is less likely to cause acute heart failure with a pansystolic murmur.  Therefore, the most likely cause of the patient's presentation is papillary muscle rupture (Option D) due to the recent inferior ST-elevation myocardial infarction. |
|  |  | Attempt 3  The most likely cause of this presentation in a 40-year-old man who develops sudden breathlessness 5 days after an acute inferior ST-elevation myocardial infarction treated by primary coronary intervention is:  D. Papillary muscle rupture  Papillary muscle rupture is a rare but serious complication of myocardial infarction. It typically occurs within the first week after the infarction and is more common in the setting of inferior myocardial infarction. Papillary muscle rupture leads to acute mitral regurgitation, which results in the development of breathlessness, a pansystolic murmur at the apex, and bibasal inspiratory crackles due to pulmonary congestion.  The sudden onset of breathlessness, the presence of a new murmur, and crackles on lung examination are suggestive of acute pulmonary edema resulting from significant mitral regurgitation. This is a life-threatening condition that requires immediate medical intervention.  While other conditions such as acute pulmonary embolus, aortic regurgitation, cardiac tamponade, and pericarditis can cause similar symptoms, the history of recent myocardial infarction and the characteristic findings of a pansystolic murmur at the apex and bibasal crackles are highly suggestive of papillary muscle rupture as the most likely cause in this scenario. |
| **84** | A 48 year old man presents to his doctor with headaches. He has noticed that his hands have become larger, and his facial features have coarsened. More recently, his vision has deteriorated. He has an upper temporal defect in both visual fields.  Damage to which structure is the most likely source of his visual problems?  A. Lateral geniculate body  B. Occipital cortex  C. Oculomotor nerve  D. Optic chiasm  E. Optic radiation | Attempt 1  The most likely source of visual problems in this case is damage to the optic chiasm.  D. Optic chiasm  The optic chiasm is a structure located at the base of the brain where the optic nerves from each eye cross over. It is responsible for the partial decussation (crossing) of the optic fibers, allowing information from the left visual field to be processed in the right hemisphere of the brain and vice versa.  In this case, the patient's description of an upper temporal defect in both visual fields suggests a bitemporal hemianopia. This visual field defect is commonly seen in lesions affecting the optic chiasm. The compression or damage to the optic chiasm can result in the loss of peripheral vision in both eyes, particularly affecting the outer (temporal) visual fields.  The other options listed (lateral geniculate body, occipital cortex, oculomotor nerve, optic radiation) are not typically associated with an upper temporal visual field defect. |
|  |  | Attempt 2  The most likely source of the patient's visual problems is damage to the optic chiasm, represented by option D.  The patient's symptoms and signs are suggestive of acromegaly, which is a condition caused by excessive growth hormone production. In acromegaly, there is often enlargement of the pituitary gland, leading to pressure on surrounding structures, including the optic chiasm.  The visual field defect described in this case (upper temporal defect in both visual fields) is known as a bitemporal hemianopia. In this condition, there is loss of peripheral vision in both eyes on the outer (temporal) aspect due to compression of the optic chiasm. The optic chiasm is the point at which the optic nerves from both eyes cross over, and it is located just beneath the pituitary gland at the base of the brain.  The other options (A, B, C, and E) do not explain the characteristic visual field defect seen in bitemporal hemianopia. Damage to the lateral geniculate body (Option A), occipital cortex (Option B), oculomotor nerve (Option C), or optic radiation (Option E) would result in different types of visual field defects, not the specific bitemporal hemianopia observed in this case. |
|  |  | Attempt 3  Damage to the optic chiasm is the most likely source of the patient's visual problems. The optic chiasm is a structure located at the base of the brain where the optic nerves from each eye partially cross over. It is responsible for the crossing of fibers from the nasal visual fields of both eyes.  In the given scenario, the patient presents with an upper temporal defect in both visual fields. This pattern of visual field loss is characteristic of a bitemporal hemianopia, which is commonly associated with lesions affecting the optic chiasm. In this condition, the fibers originating from the nasal visual fields of both eyes, which cross at the optic chiasm, are affected, leading to a loss of vision in the outer (temporal) portions of the visual fields.  The patient's symptoms, including headaches, enlargement of hands, and coarsening of facial features, are suggestive of acromegaly, a condition caused by excess growth hormone secretion, often due to a pituitary adenoma. In this case, the pituitary adenoma may be compressing the optic chiasm, leading to the visual field defects.  Therefore, the most likely source of the patient's visual problems is damage to the optic chiasm. |
| **85** | A 42 year old man has a rash on his face, mainly around his chin. The rash started 24 hours ago with a 0.5 cm thin-walled blister that then ruptured, leaving a yellow crusted lesion that has since enlarged and now other similar lesions are appearing in the same area. He is a primary school teacher.  Which is the most likely causative organism?  A. Escherichia coli  B. Pseudomonas aeruginosa  C. Staphylococcus aureus  D. Streptococcus pyogenes  E. Varicella zoster virus | Attempt 1  The most likely causative organism for the described rash is Staphylococcus aureus.  The presentation of a rash with thin-walled blisters that rupture and form yellow crusted lesions is consistent with impetigo. Impetigo is a highly contagious bacterial skin infection commonly caused by Staphylococcus aureus, although Streptococcus pyogenes (group A streptococcus) can also be involved.  Given that the patient is a primary school teacher, where close contact with children is common, impetigo is a known risk due to the ease of transmission in such settings. Staphylococcus aureus is a common cause of impetigo, and it typically presents with the characteristic features described in the scenario.  Therefore, the most likely causative organism for the patient's rash is Staphylococcus aureus. |
|  |  | Attempt 2  The most likely causative organism for the described rash is Staphylococcus aureus, represented by option C.  The clinical presentation of a rash starting as a thin-walled blister that ruptures and forms a yellow crusted lesion is characteristic of impetigo. Impetigo is a common skin infection caused by bacteria, and Staphylococcus aureus is one of the most common bacteria responsible for this condition.  Option A (Escherichia coli) and Option B (Pseudomonas aeruginosa) are not typically associated with impetigo. Escherichia coli is commonly involved in urinary tract infections and gastrointestinal infections, while Pseudomonas aeruginosa is known for causing infections in immunocompromised individuals or in the context of wounds.  Option D (Streptococcus pyogenes) is another bacterium that can cause impetigo, especially the more severe and less common form known as bullous impetigo, which presents with large, fluid-filled blisters.  Option E (Varicella zoster virus) is responsible for chickenpox, which also presents with a rash. However, chickenpox lesions are more vesicular and distributed over the entire body rather than being localized mainly around the chin. Additionally, chickenpox lesions are not typically yellow crusted like the ones seen in impetigo. |
|  |  | Attempt 3  The most likely causative organism in this case is:  D. Streptococcus pyogenes  The clinical presentation of a rash starting with a blister that ruptures and forms a yellow crusted lesion is characteristic of impetigo. Impetigo is a highly contagious bacterial skin infection, and the most common causative organism is Streptococcus pyogenes (Group A Streptococcus).  Streptococcus pyogenes can cause impetigo through direct contact with infected skin or contaminated objects. It is particularly common in settings where there is close contact among individuals, such as schools.  While other organisms listed (Escherichia coli, Pseudomonas aeruginosa, Staphylococcus aureus) can also cause skin infections, they are less commonly associated with the presentation described. Varicella zoster virus, which causes chickenpox, can present with a rash but typically starts as a diffuse rash rather than localized to the chin area. |
| **86** | A 48 year old woman has had 3 years of increasing knee pain and reduced physical activity. She has radiologically-confirmed osteoarthritis. She has hypertension and type 2 diabetes. She takes lisinopril, metformin, semaglutide and simvastatin. Her BMI is 48 kg/m2and has not changed despite lifestyle advice and a low calorie diet for the last year. Investigations: Glycated haemoglobin 55 mmol/mol (20-42)  Which is the most appropriate management?  A. Intensify lifestyle measures and review in 6 months  B. Prescribe orlistat  C. Refer for bariatric surgery  D. Refer for bilateral knee replacements  E. Start insulin therapy | Attempt 1  Given the patient's significant obesity, longstanding knee pain, and lack of response to lifestyle measures and low-calorie diet, the most appropriate management option in this case would be:  C. Refer for bariatric surgery  Bariatric surgery, such as gastric bypass or gastric sleeve, is indicated for individuals with a BMI over 40 kg/m2 (or a BMI over 35 kg/m2 with obesity-related comorbidities) who have not achieved adequate weight loss through lifestyle measures alone. In this case, the patient's BMI is 48 kg/m2, indicating severe obesity.  Bariatric surgery can lead to significant weight loss and improvement in obesity-related conditions, including osteoarthritis and metabolic disorders such as diabetes and hypertension. It can also help alleviate knee pain and improve mobility, potentially providing relief from the symptoms of osteoarthritis.  Therefore, considering the patient's lack of response to lifestyle measures and the impact of obesity on her overall health and joint pain, referral for bariatric surgery would be the most appropriate management option. |
|  |  | Attempt 2  Given the patient's history of osteoarthritis, hypertension, type 2 diabetes, and a BMI of 48 kg/m2 that has not changed despite lifestyle advice and a low-calorie diet, the most appropriate management option is:  C. Refer for bariatric surgery  Bariatric surgery is indicated for patients with severe obesity (BMI ≥ 40 kg/m2) or BMI ≥ 35 kg/m2 with obesity-related comorbidities, such as hypertension and type 2 diabetes, that are difficult to control with lifestyle measures and medication alone. In this case, the patient's BMI is 48 kg/m2, which qualifies as severe obesity, and her uncontrolled type 2 diabetes may benefit from the metabolic effects of weight loss following bariatric surgery.  Option A (Intensify lifestyle measures and review in 6 months) has already been tried for a year without significant success in weight loss, so more aggressive interventions may be required.  Option B (Prescribe orlistat) is a weight loss medication, but it is not as effective as bariatric surgery for achieving significant and sustained weight loss in severely obese individuals.  Option D (Refer for bilateral knee replacements) is not the primary treatment for obesity and is usually reserved for patients with severe knee pain and functional impairment due to advanced osteoarthritis that is not amenable to conservative measures.  Option E (Start insulin therapy) is not the appropriate management for obesity. Insulin therapy is used to manage diabetes when other measures, such as lifestyle changes and oral medications, are not sufficient to control blood glucose levels. The focus for this patient should be on addressing the underlying obesity with bariatric surgery. |
|  |  | Attempt 3  Given the patient's history of osteoarthritis, obesity, and comorbidities such as hypertension and type 2 diabetes, the most appropriate management would be to refer her for bariatric surgery (Option C).  Bariatric surgery is a viable option for patients with a BMI greater than 40 kg/m2 or a BMI greater than 35 kg/m2 with significant comorbidities such as diabetes, as in this case. Despite lifestyle advice and a low-calorie diet, the patient's BMI has not changed, indicating the need for more intensive intervention.  Bariatric surgery can lead to significant weight loss and improvement in obesity-related conditions such as osteoarthritis, hypertension, and type 2 diabetes. It can also improve mobility and quality of life for patients with severe obesity and comorbidities, potentially alleviating the knee pain and reducing the impact of osteoarthritis.  Therefore, referring the patient for bariatric surgery would be the most appropriate management in this case. |
| **87** | A 22 year old soldier steps off a cramped military aircraft following a long flight from the United Kingdom. She suddenly collapses and hits her head on the ground. While unconscious, she has asynchronous jerking of her limbs for less than 15 seconds. Witnesses say that she looked pale. She regains consciousness within 1 minute.  What is the most likely cause of her collapse?  A. Cardiac arrhythmia  B. Epilepsy  C. Hypoglycaemia  D. Pulmonary embolism  E. Vasovagal syncope | Attempt 1  Based on the given information, the most likely cause of the soldier's collapse is:  E. Vasovagal syncope  Vasovagal syncope is a common cause of fainting and is often triggered by specific stimuli or situations, such as standing for a long time, pain, emotional distress, or environmental factors like cramped spaces. In this case, the soldier had just stepped off a cramped military aircraft following a long flight, which could have triggered a vasovagal response.  The typical features of vasovagal syncope include a sudden loss of consciousness, short duration of unconsciousness (less than a minute), and subsequent spontaneous recovery. The description of asynchronous jerking of the limbs for less than 15 seconds suggests a brief seizure-like activity known as convulsive syncope, which can occur during vasovagal episodes.  While other causes such as cardiac arrhythmia, epilepsy, hypoglycemia, and pulmonary embolism can also lead to loss of consciousness, the specific trigger of a cramped military aircraft and the characteristic features of a vasovagal response make it the most likely cause in this scenario. |
|  |  | Attempt 2  Given the scenario described, the most likely cause of her collapse is:  E. Vasovagal syncope  Vasovagal syncope, also known as neurocardiogenic syncope, is a common cause of transient loss of consciousness. It occurs due to a sudden drop in heart rate and blood pressure triggered by a vagal response, often in response to emotional stress, pain, or orthostatic changes (such as standing up after a long flight in a cramped position). The person typically becomes pale and may experience jerking movements of the limbs during the syncopal episode. The loss of consciousness is usually brief, and the person regains consciousness quickly once they are lying down.  Option A (Cardiac arrhythmia) is a possibility, but it is less likely in a young healthy individual with a brief loss of consciousness and rapid recovery.  Option B (Epilepsy) is also a possibility, but the episode described is more consistent with vasovagal syncope, given the trigger and the brief duration of unconsciousness with rapid recovery.  Option C (Hypoglycemia) is less likely given the lack of evidence of altered mental status or other symptoms associated with low blood glucose levels.  Option D (Pulmonary embolism) is less likely given the absence of respiratory symptoms and the transient nature of the collapse.  Vasovagal syncope is a common and usually benign condition. However, it is essential to rule out other potential causes of syncope through a thorough evaluation and history-taking by a medical professional. |
|  |  | Attempt 3  Based on the provided information, the most likely cause of the soldier's collapse is vasovagal syncope (Option E).  Vasovagal syncope is a common cause of syncope (fainting) and typically occurs in response to triggers such as pain, emotional stress, or prolonged standing. In this case, the soldier experienced a long flight in cramped conditions, which can contribute to venous pooling and reduced cardiac output, triggering a vasovagal response.  The characteristic features supporting the diagnosis of vasovagal syncope in this case include the sudden loss of consciousness, a brief duration of unconsciousness, asynchronized limb jerking (myoclonus) lasting less than 15 seconds, and a pale appearance reported by witnesses. Regaining consciousness within 1 minute is also consistent with vasovagal syncope.  While other conditions such as cardiac arrhythmia, epilepsy, hypoglycemia, and pulmonary embolism can cause syncope, the specific features described in the scenario, along with the absence of other relevant symptoms, make vasovagal syncope the most likely cause in this context. |
| **88** | A 60 year old man has had malaise and weight loss for 3 months and during the past week has been coughing up blood. Investigation: Chest X-ray: left upper zone consolidation with cavitation  Which is the most appropriate next diagnostic investigation?  A. Bronchoscopy and lavage  B. Interferon-γ release assay (IGRA)  C. Mantoux test  D. Sputum cytology  E. Sputum microscopy | Attempt 1  Given the clinical presentation of malaise, weight loss, and coughing up blood, along with the chest X-ray findings of left upper zone consolidation with cavitation, the most appropriate next diagnostic investigation would be:  D. Sputum cytology  The presence of cavitation in the chest X-ray raises suspicion for an infectious or neoplastic process. Sputum cytology involves analyzing the cellular content of the patient's sputum to detect any abnormal cells, such as malignant cells. This test can help identify the presence of lung cancer or other neoplastic conditions.  While other investigations, such as bronchoscopy and lavage, interferon-γ release assay (IGRA), Mantoux test, and sputum microscopy, may be relevant in certain clinical scenarios, the combination of symptoms, radiographic findings, and the need to investigate for possible malignancy make sputum cytology the most appropriate initial diagnostic test in this case. |
|  |  | Attempt 2  The most appropriate next diagnostic investigation in this case is:  A. Bronchoscopy and lavage  Given the clinical presentation of malaise, weight loss, and hemoptysis (coughing up blood), along with the chest X-ray findings of left upper zone consolidation with cavitation, there is a high suspicion for possible malignancy, particularly lung cancer.  Bronchoscopy is a valuable procedure for visualizing the airways and obtaining tissue samples from the lung. It allows direct visualization of the bronchial tree, which can help identify any suspicious lesions or tumors, as well as obtain biopsy samples for further examination. Lavage (washing) of the lung's airways during bronchoscopy can also be performed to collect cells and material for analysis.  This investigation is essential to establish the diagnosis, rule out other causes, and determine the appropriate management plan for the patient.  Options B (Interferon-γ release assay) and C (Mantoux test) are tests used to diagnose tuberculosis, but they are not the most appropriate in this context as the chest X-ray findings are not typical of tuberculosis.  Options D (Sputum cytology) and E (Sputum microscopy) may be used to detect cancer cells or acid-fast bacilli, respectively, but bronchoscopy with lavage is a more direct and comprehensive approach to investigate the underlying cause of the patient's symptoms and radiological findings.  In summary, given the clinical presentation and chest X-ray findings, the most appropriate next step would be to perform bronchoscopy and lavage to further evaluate the patient's condition. |
|  |  | Attempt 3  Given the clinical presentation and findings on chest X-ray, the most appropriate next diagnostic investigation would be option D: Sputum cytology.  The presence of left upper zone consolidation with cavitation on the chest X-ray raises suspicion for a pulmonary infection or malignancy. Coughing up blood (hemoptysis) further supports the need for a thorough evaluation. Sputum cytology involves examining a sample of the patient's sputum under a microscope to detect abnormal cells, which can help identify the presence of malignancy or other pathological conditions.  While other investigations such as bronchoscopy and lavage (option A), interferon-γ release assay (option B), Mantoux test (option C), and sputum microscopy (option E) may also have diagnostic value in certain scenarios, sputum cytology is the most appropriate initial investigation in this case to evaluate for possible malignancy given the presence of coughing up blood and radiological findings. |
| **89** | A 55 year old man is referred to the vascular outpatient clinic with bilateral claudication, limiting his walking distance to 10 metres. He is a smoker. Imaging shows chronic distal aortic and bilateral common iliac occlusive disease.  Which is the most appropriate surgical intervention?  A. Aortic endarterectomy  B. Aorto-bifemoral bypass graft  C. Aorto-iliac embolectomy  D. Bilateral iliac angioplasty  E. Femoral-to-femoral crossover graft | Attempt 1  Based on the imaging findings of chronic distal aortic and bilateral common iliac occlusive disease, the most appropriate surgical intervention in this case would be:  B. Aorto-bifemoral bypass graft  Aorto-bifemoral bypass graft involves bypassing the occluded or stenotic segments of the aorta and both common iliac arteries by creating a graft that connects the aorta to the femoral arteries bilaterally. This allows for improved blood flow to the lower extremities, relieving the symptoms of claudication and increasing the patient's walking distance.  While other options such as aortic endarterectomy, aorto-iliac embolectomy, bilateral iliac angioplasty, and femoral-to-femoral crossover graft may be suitable in certain cases, the presence of chronic occlusive disease involving the distal aorta and bilateral common iliac arteries makes aorto-bifemoral bypass graft the most appropriate surgical intervention to address the extent of the occlusive disease and improve blood flow to the lower extremities. |
|  |  | Attempt 2  The most appropriate surgical intervention for a patient with chronic distal aortic and bilateral common iliac occlusive disease and bilateral claudication is:  B. Aorto-bifemoral bypass graft  Aorto-bifemoral bypass is the preferred surgical procedure for patients with aortoiliac occlusive disease causing bilateral claudication. During this procedure, a vascular surgeon creates a bypass graft using synthetic material to reroute blood flow around the blocked or narrowed segments of the aorta and common iliac arteries, restoring blood flow to the femoral arteries. This improves blood supply to the lower extremities, relieving the symptoms of claudication and allowing the patient to walk longer distances without pain.  Option A (Aortic endarterectomy) is not the most appropriate choice for this patient as endarterectomy involves the removal of plaque from the inner lining of an artery, and it may not be suitable for extensive occlusive disease involving both aortoiliac and femoral arteries.  Option C (Aorto-iliac embolectomy) is typically used for acute limb ischemia caused by emboli, not for chronic occlusive disease.  Option D (Bilateral iliac angioplasty) involves the use of a balloon to widen narrowed arteries, and it may be considered in some cases, but aorto-bifemoral bypass is generally the preferred procedure for more extensive occlusive disease.  Option E (Femoral-to-femoral crossover graft) is used when there is a single-sided aortoiliac occlusive disease, but in this case, the patient has bilateral occlusive disease, making the aorto-bifemoral bypass a more appropriate choice.  In summary, the most appropriate surgical intervention for this patient with bilateral claudication and chronic distal aortic and bilateral common iliac occlusive disease is aorto-bifemoral bypass graft. |
|  |  | Attempt 3  Based on the provided information of chronic distal aortic and bilateral common iliac occlusive disease causing bilateral claudication, the most appropriate surgical intervention would be option B: Aorto-bifemoral bypass graft.  Aorto-bifemoral bypass grafting is a well-established surgical procedure used to bypass the occluded or narrowed segments of the aorta and common iliac arteries. It involves creating a bypass using a synthetic graft to restore blood flow to the femoral arteries and improve lower limb perfusion. This procedure is commonly performed in cases of severe aortoiliac occlusive disease to alleviate symptoms of claudication and improve exercise tolerance.  Option A (Aortic endarterectomy) is not appropriate in this case as the patient's occlusive disease involves the distal aorta and common iliac arteries, not the proximal aorta.  Option C (Aorto-iliac embolectomy) is not appropriate as the occlusion is chronic, indicating atherosclerotic disease rather than acute embolic occlusion.  Option D (Bilateral iliac angioplasty) may be considered as an alternative minimally invasive approach, but it is generally preferred in cases with focal stenosis or short-segment occlusions rather than extensive disease involving the distal aorta.  Option E (Femoral-to-femoral crossover graft) is used when there is unilateral iliac artery occlusion and is not suitable for bilateral occlusive disease.  Therefore, the most appropriate surgical intervention in this case is aorto-bifemoral bypass graft (option B). |
| **90** | A 65 year old man sees his GP for monitoring of hypertension and ischaemic heart disease. His medication includes aspirin, atenolol, amlodipine, lisinopril and simvastatin. He has marked ankle swelling.  Which drug is the most likely cause of his ankle swelling?  A. Amlodipine  B. Aspirin  C. Atenolol  D. Lisinopril  E. Simvastatin | Attempt 1  The most likely cause of the ankle swelling in this case is:  A. Amlodipine  Amlodipine is a calcium channel blocker commonly used to treat hypertension and angina. Ankle swelling is a known side effect of amlodipine, occurring in a small percentage of patients. It is caused by the dilation of blood vessels, which can lead to fluid retention in the lower extremities.  While other medications on the list, such as lisinopril (an ACE inhibitor) and simvastatin (a statin), can also cause ankle swelling as a side effect, amlodipine is most commonly associated with this symptom. It is important for the patient to inform their healthcare provider about the ankle swelling so that appropriate adjustments to the medication regimen can be made if necessary. |
|  |  | Attempt 2  The most likely cause of the ankle swelling in this patient is:  A. Amlodipine  Amlodipine is a calcium channel blocker commonly used to treat hypertension and angina. One of its side effects is peripheral edema or ankle swelling, which occurs due to the dilation of blood vessels in the legs, leading to increased fluid accumulation in the tissues.  The other medications listed (aspirin, atenolol, lisinopril, and simvastatin) can also cause side effects, but ankle swelling is not a common side effect of these medications.  It's important for the patient to report the ankle swelling to their doctor, as the medication may need to be adjusted or an alternative drug may be considered to manage his hypertension while minimizing the side effects. |
|  |  | Attempt 3  Among the medications listed, amlodipine (option A) is the most likely cause of ankle swelling in this patient.  Amlodipine is a calcium channel blocker commonly used to treat hypertension and angina. One of the side effects of amlodipine is peripheral edema, which can manifest as swelling in the ankles and feet. This occurs due to the dilation of blood vessels, resulting in increased fluid leakage into the surrounding tissues.  The other medications listed, including aspirin (option B), atenolol (option C), lisinopril (option D), and simvastatin (option E), are less commonly associated with ankle swelling compared to amlodipine.  It is worth noting that ankle swelling can have various causes, including heart failure, kidney disease, venous insufficiency, and certain medications. In this case, considering the patient's medication regimen, amlodipine is the most likely culprit. However, further evaluation and assessment by a healthcare professional are necessary to confirm the exact cause of the ankle swelling. |
| **91** | An 85 year old woman was admitted with a stroke three weeks ago. She has a history of urinary incontinence and has a long-term urinary catheter in place. Her current medication includes clopidogrel and ramipril. Her temperature is 36.8°C, pulse rate 85 bpm and BP 134/74 mmHg. A catheter specimen of urine shows >105CFU/mL, mixed growth.  Which is the most appropriate management?  A. No change in treatment  B. Remove urinary catheter  C. Request antibiotic sensitivities  D. Start oral ciprofloxacin  E. Start oral trimethoprim | Attempt 1  In an 85-year-old woman with a long-term urinary catheter and a positive catheter specimen of urine indicating a urinary tract infection (UTI), the most appropriate management option would be to start oral trimethoprim (option E).  UTIs are common in individuals with long-term urinary catheters, and the presence of symptoms such as urinary incontinence and the positive urine culture with significant bacterial growth (>105 CFU/mL) support the diagnosis of a UTI. In this case, the management goal is to treat the infection and minimize potential complications.  Trimethoprim is a commonly used antibiotic for treating UTIs, and it provides coverage against many of the typical pathogens involved, including Escherichia coli, which is the most common cause of UTIs. Starting oral trimethoprim would be an appropriate first-line treatment choice in this scenario.  Options A and C are not appropriate because treatment is warranted given the positive urine culture. Option B (removing the urinary catheter) may be considered in certain cases, but the decision should be made based on a thorough evaluation of the patient's clinical condition and discussion with the healthcare team. Option D (starting oral ciprofloxacin) is generally not recommended as a first-line treatment for UTIs due to increasing antibiotic resistance.  It's important to note that the final decision regarding management should be made by a healthcare professional based on the patient's individual circumstances and the available clinical guidelines. |
|  |  | Attempt 2  The most appropriate management for this patient with a long-term urinary catheter and a positive catheter specimen of urine showing >105 CFU/mL mixed growth is:  C. Request antibiotic sensitivities  In the presence of a long-term urinary catheter, it is common for patients to develop asymptomatic bacteriuria (bacteria in the urine) due to the colonization of the catheter. Asymptomatic bacteriuria does not require treatment with antibiotics, as it does not cause symptoms or complications in most cases. Treating asymptomatic bacteriuria with antibiotics can lead to antibiotic resistance and other adverse effects.  In this case, since the patient is not showing any signs of a urinary tract infection (such as fever, urinary symptoms, or systemic symptoms), the most appropriate step is to request antibiotic sensitivities. This will help identify the appropriate antibiotic if the patient develops symptomatic urinary tract infection in the future or if the infection worsens.  It is important to note that urinary catheters should be used judiciously and removed as soon as they are no longer necessary to reduce the risk of complications such as urinary tract infections. However, in this case, there is no indication to remove the urinary catheter solely based on the presence of asymptomatic bacteriuria. |
|  |  | Attempt 3  The most appropriate management in this case is:  C. Request antibiotic sensitivities  The patient has a urinary tract infection (UTI) as evidenced by a catheter specimen of urine showing >105 CFU/mL of mixed growth. Given the presence of a long-term urinary catheter, UTIs are common and can lead to complications, including systemic infections. Therefore, it is important to address the infection appropriately.  Instead of initiating immediate antibiotic treatment (options D and E), it is advisable to request antibiotic sensitivities first. This helps identify the most effective antibiotic for targeting the specific bacteria causing the UTI. The choice of antibiotic should be based on the sensitivities to ensure targeted and effective treatment.  Removing the urinary catheter (option B) may be considered as a long-term management strategy to prevent recurrent UTIs, but it is not the immediate management for the current infection. The decision to remove the catheter should be made in consultation with the healthcare team.  Option A (no change in treatment) is not appropriate because the patient has a documented UTI and requires intervention.  Therefore, the most appropriate next step is to request antibiotic sensitivities to guide the choice of antibiotic for targeted treatment of the UTI. |
| **92** | A 28 year old man has a headache, intermittent fever, sore throat and diarrhoea. His temperature is 37.7°C. His fauces are red and there are two small aphthous ulcers on his left buccal mucosa. He also has a maculopapular erythematous rash on his upper trunk, red hands and folliculitis on his chest. His liver and spleen are just palpable and he has mild neck stiffness. Investigations: Haemoglobin 135 g/L (130–175) White cell count 3.3 x 109/L (3.0–10.0) Platelets 84 x 109/L (150–400)  Which investigation is most likely to lead to a diagnosis?  A. First catch urine microscopy  B. Glandular fever screening test  C. HIV serology  D. Serum antinuclear antibodies  E. Serum toxoplasma gondii IgM antibody titre | Attempt 1  Given the clinical presentation and the laboratory findings, the most appropriate investigation to lead to a diagnosis in this case is:  B. Glandular fever screening test  The patient presents with symptoms suggestive of an infectious mononucleosis-like syndrome. The combination of headache, intermittent fever, sore throat, diarrhea, maculopapular rash, aphthous ulcers, and hepatosplenomegaly is consistent with infectious mononucleosis, which is most commonly caused by the Epstein-Barr virus (EBV) infection.  A glandular fever screening test, which typically involves detecting antibodies against EBV, can help confirm the diagnosis. This test is commonly used to detect specific antibodies, such as heterophile antibodies (Monospot test), that are produced in response to the EBV infection.  Option A (first catch urine microscopy) is not likely to provide relevant information related to the presented symptoms.  Option C (HIV serology) can be considered in patients with risk factors for HIV or with symptoms suggestive of primary HIV infection, but it is less likely in this case given the characteristic clinical presentation of infectious mononucleosis.  Option D (serum antinuclear antibodies) is more specific for autoimmune conditions and is not the most relevant investigation in this scenario.  Option E (serum Toxoplasma gondii IgM antibody titre) is not the most appropriate investigation as the clinical picture, including the characteristic rash, hepatosplenomegaly, and aphthous ulcers, is more consistent with infectious mononucleosis caused by EBV rather than acute toxoplasmosis.  Therefore, a glandular fever screening test would be the most appropriate investigation to confirm the diagnosis in this case. |
|  |  | Attempt 2  Based on the clinical presentation of headache, intermittent fever, sore throat, diarrhea, maculopapular rash, red hands, and mild neck stiffness, along with the laboratory findings of leukopenia and thrombocytopenia, the most likely diagnosis is viral exanthem.  The most appropriate investigation to confirm this diagnosis and rule out other possibilities would be:  B. Glandular fever screening test  Glandular fever, also known as infectious mononucleosis, is a viral infection caused by the Epstein-Barr virus (EBV). It commonly presents with symptoms such as fever, sore throat, rash, and lymphadenopathy. The presence of leukopenia and thrombocytopenia can also be seen in some cases.  The glandular fever screening test typically involves detecting specific antibodies against the Epstein-Barr virus. Positive results would support the diagnosis of infectious mononucleosis, which is a common cause of viral exanthems in young adults.  It is essential to rule out other potential causes of the symptoms, such as HIV, which can also present with similar features. However, based on the clinical presentation and the most likely diagnosis, the glandular fever screening test would be the most appropriate initial investigation. Further tests may be considered based on the results of the screening and the patient's clinical course. |
|  |  | Attempt 3  In a 28-year-old man presenting with symptoms such as headache, intermittent fever, sore throat, and gastrointestinal symptoms along with characteristic clinical findings like aphthous ulcers, rash, and hepatosplenomegaly, the most likely diagnosis is infectious mononucleosis, also known as glandular fever. Therefore, the most appropriate investigation to confirm the diagnosis would be a glandular fever screening test (option B).  Infectious mononucleosis is typically caused by the Epstein-Barr virus (EBV), and it commonly presents with the symptoms described above. The characteristic features of the disease, including the presence of pharyngitis, lymphadenopathy, hepatosplenomegaly, and the presence of atypical lymphocytes on blood smear, are suggestive of glandular fever.  A glandular fever screening test usually involves detecting specific antibodies against the Epstein-Barr virus. It can help confirm the diagnosis by detecting the presence of antibodies (such as heterophile antibodies) produced in response to the infection.  Option A (first catch urine microscopy) is not relevant to the suspected diagnosis of glandular fever. Option C (HIV serology) may be considered in certain cases, but the presentation described is more consistent with infectious mononucleosis caused by EBV. Option D (serum antinuclear antibodies) is not specific for glandular fever and is more commonly associated with autoimmune diseases. Option E (serum toxoplasma gondii IgM antibody titre) is not the most appropriate investigation in this context and is unlikely to be associated with the symptoms and findings described.  However, it's important to note that the final diagnosis should be made by a healthcare professional based on a thorough evaluation of the patient's clinical history, examination, and appropriate diagnostic tests. |
| **93** | A 79 year old woman has six months of increasing breathlessness on exertion. Her pulse rate is 72 bpm, irregularly irregular, and BP 118/72 mmHg. She has a diastolic murmur best heard at the apex in expiration.  Which is the most likely cause of her murmur?  A. Aortic regurgitation  B. Aortic stenosis  C. Hypertrophic cardiomyopathy  D. Mitral regurgitation  E. Mitral stenosis | Attempt 1  Based on the provided information, the most likely cause of the diastolic murmur heard at the apex in expiration in this 79-year-old woman is mitral stenosis (option E).  Mitral stenosis is characterized by narrowing of the mitral valve orifice, which impairs the flow of blood from the left atrium to the left ventricle during diastole. The murmur associated with mitral stenosis is typically low-pitched and rumbling in quality. It is best heard at the apex and is accentuated during expiration.  Option A (aortic regurgitation) is characterized by a diastolic murmur that is best heard at the left sternal border and may be associated with a collapsing pulse. Option B (aortic stenosis) typically presents with a systolic murmur that is best heard at the right upper sternal border. Option C (hypertrophic cardiomyopathy) is characterized by a systolic murmur that may vary in intensity and quality and is usually heard best at the left lower sternal border. Option D (mitral regurgitation) is associated with a pansystolic murmur that is best heard at the apex and often radiates to the axilla.  It's important to note that a definitive diagnosis requires a thorough evaluation by a healthcare professional, including additional diagnostic tests such as echocardiography, to confirm the underlying cause of the murmur. |
|  |  | Attempt 2  Based on the information provided, the most likely cause of the diastolic murmur heard at the apex during expiration in this 79-year-old woman is:  D. Mitral regurgitation  Mitral regurgitation is a condition where there is backward flow of blood from the left ventricle into the left atrium during ventricular diastole. The murmur associated with mitral regurgitation is typically heard best at the apex and is described as a high-pitched, blowing murmur. It is often associated with an irregularly irregular pulse, which could indicate atrial fibrillation.  Mitral regurgitation can cause symptoms of breathlessness on exertion, as the heart's ability to pump effectively is compromised. The condition can be caused by various factors, such as mitral valve prolapse, rheumatic heart disease, infective endocarditis, or degenerative changes in the mitral valve.  It is important to note that the other options listed (Aortic regurgitation, Aortic stenosis, Hypertrophic cardiomyopathy, and Mitral stenosis) have different characteristics in terms of their murmurs and associated clinical features. In this case, the most likely cause based on the information given is mitral regurgitation. Further evaluation and diagnostic tests would be necessary to confirm the diagnosis and determine the underlying cause of the murmur. |
|  |  | Attempt 3  Based on the provided information, the most likely cause of the murmur in this 79-year-old woman is:  D. Mitral regurgitation  The characteristics of an irregularly irregular pulse and a diastolic murmur best heard at the apex in expiration are suggestive of mitral regurgitation. Mitral regurgitation is the backflow of blood from the left ventricle into the left atrium during systole due to the incomplete closure of the mitral valve. It can result from various causes, including mitral valve prolapse, rheumatic heart disease, infective endocarditis, or age-related degenerative changes.  Aortic regurgitation (option A) typically presents with a diastolic murmur heard best at the left sternal border and is associated with a wide pulse pressure.  Aortic stenosis (option B) typically presents with a systolic murmur that is heard best at the right upper sternal border and radiates to the carotid arteries.  Hypertrophic cardiomyopathy (option C) typically presents with a systolic murmur that is heard best at the left lower sternal border and increases with maneuvers that decrease preload (such as Valsalva maneuver).  Mitral stenosis (option E) typically presents with a diastolic murmur heard best at the apex, but it is usually preceded by an opening snap and is associated with findings of left atrial enlargement.  Therefore, based on the information provided, mitral regurgitation is the most likely cause of the diastolic murmur in this case. However, further evaluation and diagnostic tests, such as echocardiography, would be necessary to confirm the diagnosis and determine the underlying cause. |
| **94** | A 78 year old woman is found dead at home. At autopsy, the pathologist finds bilateral pneumonia and meningitis. Microscopy of a meningeal swab shows Gram-positive cocci arranged in pairs.  Which is the most likely causative organism?  A. Candida albicans  B. Neisseria meningitidis  C. Pseudomonas aeruginosa  D. Staphylococcus aureus  E. Streptococcus pneumoniae | Attempt 1  Based on the information provided, the most likely causative organism for the bilateral pneumonia and meningitis in this case is:  E. Streptococcus pneumoniae  Streptococcus pneumoniae is a Gram-positive cocci that commonly causes pneumonia and meningitis. It is a leading cause of bacterial pneumonia, particularly in the elderly population. The organism is often found in pairs (diplococci) when viewed under a microscope.  Candida albicans (option A) is a fungal organism and is not typically associated with bacterial pneumonia or meningitis.  Neisseria meningitidis (option B) is a Gram-negative diplococcus that can cause meningitis but is less likely to cause pneumonia.  Pseudomonas aeruginosa (option C) is a Gram-negative bacterium known for its opportunistic infections, but it is not a common cause of community-acquired pneumonia or meningitis.  Staphylococcus aureus (option D) can cause various infections, including pneumonia and meningitis, but it is less commonly associated with bilateral pneumonia and meningitis compared to Streptococcus pneumoniae.  Therefore, based on the findings of Gram-positive cocci arranged in pairs and the clinical presentation of pneumonia and meningitis, the most likely causative organism in this case is Streptococcus pneumoniae. |
|  |  | Attempt 2  Based on the information provided, the most likely causative organism for the bilateral pneumonia and meningitis in the 78-year-old woman is:  E. Streptococcus pneumoniae  Streptococcus pneumoniae, also known as pneumococcus, is a Gram-positive bacterium and a common cause of both pneumonia and bacterial meningitis, particularly in elderly individuals and those with certain risk factors such as immunocompromised status or chronic illnesses.  In this case, the presence of Gram-positive cocci arranged in pairs on microscopy of the meningeal swab is suggestive of Streptococcus pneumoniae, which is a characteristic microscopic appearance for this bacterium.  It is important to note that the other organisms listed (Candida albicans, Neisseria meningitidis, Pseudomonas aeruginosa, and Staphylococcus aureus) can cause infections in various settings, but they are less commonly associated with the combination of pneumonia and bacterial meningitis in this age group. The specific diagnosis would require appropriate culture and identification of the organism from the clinical samples obtained during autopsy. |
|  |  | Attempt 3  Based on the information provided, the most likely causative organism for the bilateral pneumonia and meningitis in the 78-year-old woman is Streptococcus pneumoniae (option E).  Streptococcus pneumoniae is a Gram-positive cocci that commonly causes pneumonia and meningitis, especially in older adults and individuals with predisposing factors. The characteristic finding of Gram-positive cocci arranged in pairs (diplococci) is consistent with the morphology of Streptococcus pneumoniae.  Option A (Candida albicans) is a fungal organism and is not typically associated with pneumonia and meningitis. Option B (Neisseria meningitidis) is a Gram-negative diplococcus that can cause meningitis, but it is less likely to cause bilateral pneumonia. Option C (Pseudomonas aeruginosa) is a Gram-negative bacterium that can cause severe infections but is less commonly associated with meningitis. Option D (Staphylococcus aureus) is a Gram-positive bacterium that can cause pneumonia and meningitis, but the presence of Gram-positive cocci in pairs is more indicative of Streptococcus pneumoniae.  It's important to note that a definitive identification of the causative organism would require further laboratory testing, such as culture and sensitivity analysis, to confirm the specific bacterial strain and guide appropriate treatment. |
| **95** | A 40 year old man was admitted to hospital with central crushing chest pain. Examination revealed pale cream coloured nodules on both elbows and medial aspects of his upper eyelids. ECG on admission showed ST elevation and T wave inversion. He deteriorated and died. A post mortem examination is performed and shows very severe narrowing of the anterior descending branch of the left coronary artery.  Which is the most likely causative mechanism?  A. Atheroma  B. Arterial dissection  C. Malignant deposit  D. Thrombosis  E. Vasculitis | Attempt 1  The most likely causative mechanism for the severe narrowing of the anterior descending branch of the left coronary artery in the 40-year-old man with central crushing chest pain and the presence of pale cream coloured nodules on his elbows and upper eyelids is Atheroma (option A).  Atheroma refers to the formation of a plaque within the arterial wall composed of cholesterol, fatty deposits, cellular debris, and connective tissue. Over time, these plaques can progressively narrow the arterial lumen, leading to reduced blood flow to the affected tissue. In the case of coronary arteries, atheroma can lead to coronary artery disease (CAD), resulting in angina and potentially acute coronary syndrome, such as myocardial infarction (heart attack).  The presence of pale cream coloured nodules on the elbows and upper eyelids is suggestive of xanthomas, which are fatty deposits that can occur in individuals with high cholesterol levels. This finding further supports the likelihood of atherosclerosis as the underlying cause of the severe narrowing in the coronary artery.  Option B (Arterial dissection) refers to the separation of the layers of an artery wall, which can cause narrowing or occlusion of the arterial lumen. However, the presence of atherosclerotic plaques is more commonly associated with the development of symptoms in coronary artery disease.  Option C (Malignant deposit) is less likely in this case, as the patient's symptoms and findings are more consistent with atherosclerosis rather than a malignant process.  Option D (Thrombosis) can occur on top of an atherosclerotic plaque, leading to acute coronary syndrome. However, the severe narrowing observed in the coronary artery is more likely due to the chronic development of atherosclerotic plaques.  Option E (Vasculitis) refers to inflammation of the blood vessel wall, which can also cause narrowing or occlusion of the vessel. However, the presence of xanthomas and the clinical context make atherosclerosis a more probable cause.  It's important to note that a definitive diagnosis would require a comprehensive evaluation, including clinical history, imaging studies, and potentially histopathological examination. |
|  |  | Attempt 2  Based on the clinical presentation and findings, the most likely causative mechanism for the severe narrowing of the anterior descending branch of the left coronary artery and the patient's symptoms is:  D. Thrombosis  Thrombosis refers to the formation of a blood clot (thrombus) within a blood vessel, which can obstruct blood flow and lead to tissue damage or infarction. In this case, the patient's central crushing chest pain and the presence of ST elevation and T wave inversion on ECG are indicative of an acute coronary syndrome, likely caused by thrombotic occlusion of the anterior descending branch of the left coronary artery.  The pale cream-coloured nodules on both elbows and medial aspects of the upper eyelids are characteristic findings of xanthomas, which are cholesterol-rich deposits seen in conditions associated with dyslipidemia, such as familial hypercholesterolemia. These xanthomas are not the primary cause of the coronary artery thrombosis but are associated with the underlying lipid abnormality contributing to the development of atheromatous plaques.  While other mechanisms listed (Atheroma, Arterial dissection, Malignant deposit, and Vasculitis) can cause arterial narrowing or occlusion, they are less likely to be the primary cause of the severe coronary artery narrowing in this scenario. Thrombosis is a common mechanism in acute coronary syndromes leading to myocardial infarction (heart attack). |
|  |  | Attempt 3  Based on the information provided, the most likely causative mechanism for the severe narrowing of the anterior descending branch of the left coronary artery in this case is:  D. Thrombosis  The patient's presentation with central crushing chest pain, the presence of pale cream coloured nodules on the elbows and upper eyelids (known as xanthomas), and the ECG findings of ST elevation and T wave inversion suggest a diagnosis of acute myocardial infarction (heart attack). The severe narrowing of the coronary artery indicates a significant blockage of blood flow to the heart.  In the context of a heart attack, thrombosis is the most likely causative mechanism. Atheroma (option A) refers to the buildup of fatty deposits within the arterial walls and is a common underlying cause of thrombosis. Arterial dissection (option B) involves a tear in the arterial wall, leading to the formation of a false lumen and potential thrombosis, but it is less likely in this case. Malignant deposits (option C) and vasculitis (option |
| **96** | A 55 year old man visits his GP concerned that he may be a carrier of cystic fibrosis, a condition that has just been diagnosed in his 5 year old grandson. He has heard that this is an inherited condition, but no one else in his family has the illness.  What is the likelihood that the grandfather is a carrier?  A. 1 in 2  B. 1 in 4  C. 1 in 8  D. 1 in 16  E. 1 in 25 | Attempt 1  Cystic fibrosis (CF) is an autosomal recessive genetic disorder, which means that it requires two copies of the mutated gene (one from each parent) to manifest the condition. Carriers of CF have one normal copy of the CF gene and one mutated copy, but they do not develop the disease themselves.  Since the man's grandson has been diagnosed with CF, we know that both of the child's parents must be carriers. When two carriers have a child together, there is a 25% chance that the child will inherit two copies of the mutated gene and have CF, a 50% chance that the child will be a carrier like their parents, and a 25% chance that the child will inherit two normal copies of the gene and not be a carrier.  In this case, the grandfather is concerned that he may be a carrier. Since his grandson has CF, it means that his own child (the parent of the affected grandchild) must be a carrier. If the grandfather's child is a carrier, there is a 50% chance that the grandfather himself is a carrier.  Therefore, the likelihood that the grandfather is a carrier of cystic fibrosis is 1 in 2 (option A). |
|  |  | Attempt 2  Since the grandson has cystic fibrosis, which is an autosomal recessive condition, it means both of his parents must be carriers (heterozygous) for the cystic fibrosis gene. If the grandfather is concerned that he might be a carrier, it means he is one of the potential parents of the affected child.  In this case, the likelihood that the grandfather is a carrier (heterozygous) for cystic fibrosis is 1 in 4 (B). |
|  |  | Attempt 3  Cystic fibrosis is an autosomal recessive genetic disorder, meaning that it requires two copies of the faulty gene (one from each parent) for the individual to have the disease. If the grandfather's grandson has cystic fibrosis, it means that his son or daughter (the parent of the grandson) is a carrier of the gene mutation.  To determine the likelihood that the grandfather is a carrier, we need to consider the probability that his son or daughter inherited the faulty gene from him. Since no one else in the family has the illness, it suggests that the grandfather himself is not affected by cystic fibrosis, which means he is either not a carrier or has a very low chance of being a carrier.  Based on the information given, it is most likely that the grandfather's chance of being a carrier is low, approaching the general population prevalence. In the general population, the carrier frequency of cystic fibrosis is about 1 in 25 (0.04 or 4%). Therefore, the most appropriate answer would be:  E. 1 in 25 |
| **97** | A 79 year old woman has been repeatedly found wandering at night by her neighbours. The problem has progressively worsened over 6 months. She is independent in her activities of daily living, although her family do her shopping. She was previously well.  What aspect of cognition is likely to show the greatest impairment?  A. Attention  B. Concentration  C. Praxis  D. Registration of information  E. Short-term memory | Attempt 1  Based on the information provided, the aspect of cognition that is likely to show the greatest impairment in the 79-year-old woman is:  E. Short-term memory  The woman's repeated wandering at night and the progressive worsening of her problem suggest a memory-related issue. Short-term memory refers to the ability to hold and recall information over a brief period, typically seconds to minutes. Impairments in short-term memory can lead to difficulties with remembering recent events, retaining new information, and maintaining attention to ongoing tasks. In this case, the woman's inability to retain the information about her neighbors' warnings and her repeated wandering indicate a significant impairment in short-term memory. |
|  |  | Attempt 2  Given the information provided, the most likely aspect of cognition to show the greatest impairment in this 79-year-old woman is:  E. Short-term memory  The key features that indicate short-term memory impairment in this case are the episodes of wandering at night and the progressive worsening of the problem over the last 6 months. Short-term memory is responsible for retaining and recalling information for a brief period, and its decline can lead to difficulties in remembering recent events and actions, such as getting lost or forgetting where one is or what one is doing. |
|  |  | Attempt 3  Based on the given information, the aspect of cognition that is likely to show the greatest impairment in the 79-year-old woman is E. Short-term memory.  The fact that she is repeatedly found wandering at night and that the problem has progressively worsened over 6 months suggests a decline in her short-term memory. Short-term memory refers to the ability to hold and manipulate information in mind for a brief period of time. It is responsible for remembering recent events, retaining new information, and carrying out tasks that require immediate recall.  The woman's ability to perform her activities of daily living independently suggests that her long-term memory and other cognitive functions, such as attention, concentration, and praxis, may still be relatively intact. However, the progressive worsening of her wandering behavior indicates a deficit in her ability to retain and recall recent information, indicating an impairment in short-term memory. |
| **98** | A 52 year old woman reports increased urinary frequency, urgency and urge incontinence. She has multiple sclerosis, which affects her walking. A midstream urine sample shows no cells and is sterile on culture. A bladder scan shows a residual volume of 300 mL. Urodynamic assessment shows that she has a neuropathic bladder.  Which is the most appropriate management?  A. α-Adrenoceptor blocker  B. Anticholinergic drug  C. Indwelling urethral catheter  D. Intermittent self catheterisation  E. Suprapubic catheter | Attempt 1  The most appropriate management for the 52-year-old woman with multiple sclerosis and a neuropathic bladder is:  D. Intermittent self-catheterization  The woman's symptoms of increased urinary frequency, urgency, and urge incontinence, along with a residual bladder volume of 300 mL, indicate impaired bladder function. Intermittent self-catheterization is a commonly used method for managing neurogenic bladder dysfunction. It involves the regular insertion and removal of a catheter to empty the bladder at scheduled intervals, allowing for better bladder control and prevention of urinary retention. This approach helps to effectively manage the symptoms of urinary frequency and urgency while reducing the risk of complications associated with long-term catheterization. |
|  |  | Attempt 2  The most appropriate management for a patient with neuropathic bladder, as described in the case of the 52-year-old woman with multiple sclerosis and increased urinary frequency, urgency, and urge incontinence, is:  D. Intermittent self-catheterization  Intermittent self-catheterization (ISC) is a method where the patient inserts a catheter into the bladder at regular intervals to empty the bladder. This is a recommended approach in patients with neurogenic bladder dysfunction, as it allows for effective bladder emptying, reduces the risk of urinary tract infections, and helps manage the symptoms of urgency and incontinence. It is considered a safer and more effective alternative to indwelling urethral catheters, which have a higher risk of complications such as infection and bladder stones. Anticholinergic drugs may exacerbate urinary retention in patients with neurogenic bladder, and α-adrenoceptor blockers are not typically used in this scenario. A suprapubic catheter may be considered in certain cases, but intermittent self-catheterization is often the first-line choice. |
|  |  | Attempt 3  Based on the given information, the most appropriate management for the 52-year-old woman with neuropathic bladder due to multiple sclerosis would be D. Intermittent self-catheterization.  Neuropathic bladder is a dysfunction of the bladder due to neurological damage, such as in the case of multiple sclerosis. It can lead to symptoms of increased urinary frequency, urgency, and urge incontinence, as described by the patient.  Intermittent self-catheterization (ISC) is a technique where the patient inserts a catheter into the urethra to empty the bladder at regular intervals. This method helps to effectively empty the bladder, reduce the risk of urinary retention and associated complications, and manage the symptoms of urinary frequency and urgency.  Other options may be considered in certain situations, but ISC is generally the first-line treatment for managing neurogenic bladder dysfunction in patients with multiple sclerosis. Alpha-adrenoceptor blockers and anticholinergic drugs may have limited efficacy in this case, and indwelling urethral catheters or suprapubic catheters are usually reserved for patients who are unable to perform intermittent self-catheterization or have failed conservative management. However, the specific management plan should be determined in consultation with a healthcare professional experienced in managing neurogenic bladder conditions. |
| **99** | A 43 year old woman is admitted with acute right upper quadrant pain, which radiates to her right shoulder. Her temperature is 38.6°C and respiratory rate 20 breaths per minute. She is tender to palpation in the right upper quadrant but has no rebound tenderness. Investigations: Haemoglobin 132 g/L (115–150) White cell count 13 x 109/L (3.8–10.0) Platelets 340 x 109/L (150–400) Bilirubin 30 μmol/L (<17) Alanine aminotransferase (ALT) 80 IU/L (10–50) Alkaline phosphatase 306 IU/L (25–115)  Which is the next most appropriate radiological test?  A. Abdominal X-ray  B. CT scan of abdomen  C. Erect chest X-ray  D. MR scan of abdomen  E. Ultrasound scan of abdomen | Attempt 1  The next most appropriate radiological test for the 43-year-old woman with acute right upper quadrant pain is:  E. Ultrasound scan of the abdomen  The patient's clinical presentation, including the acute right upper quadrant pain that radiates to the right shoulder, along with the laboratory results showing elevated liver enzymes and bilirubin, suggests a possible biliary pathology, such as acute cholecystitis or biliary obstruction. An ultrasound scan of the abdomen is the initial imaging modality of choice to evaluate the biliary system and assess the presence of gallstones, gallbladder inflammation, or other abnormalities. It is a non-invasive and widely available imaging technique that can help confirm the diagnosis and guide further management. |
|  |  | Attempt 2  The next most appropriate radiological test for the patient with acute right upper quadrant pain and elevated liver function tests is:  E. Ultrasound scan of the abdomen  An ultrasound scan of the abdomen is the initial imaging test of choice in a patient with suspected acute cholecystitis, given its ability to visualize the gallbladder and identify gallstones or signs of inflammation, such as gallbladder wall thickening or pericholecystic fluid. It is a non-invasive, safe, and readily available imaging modality that can help confirm the diagnosis and guide further management. It is particularly useful for evaluating the biliary system, which is often implicated in cases of right upper quadrant pain with elevated liver function tests. CT scan and MR scan are other imaging options but are typically used when ultrasound results are inconclusive or to evaluate for complications if needed. Abdominal X-ray and erect chest X-ray are not indicated in this context and are less useful for the evaluation of acute cholecystitis. |
|  |  | Attempt 3  In a patient with acute right upper quadrant pain and clinical signs of possible acute cholecystitis, the next most appropriate radiological test is E. Ultrasound scan of the abdomen.  An ultrasound scan of the abdomen is the initial imaging investigation of choice for suspected acute cholecystitis. It is a non-invasive, readily available, and cost-effective imaging modality that can help confirm the diagnosis. The ultrasound can identify gallbladder distention, gallstones, thickening of the gallbladder wall, pericholecystic fluid collection, and signs of inflammation, such as sonographic Murphy's sign (tenderness of the gallbladder upon probe pressure).  Other radiological tests, such as a CT scan of the abdomen or MR scan of the abdomen, may be considered if there is diagnostic uncertainty or if there are specific indications based on the clinical presentation. An abdominal X-ray or erect chest X-ray would not provide sufficient information for the evaluation of acute cholecystitis and are not the most appropriate initial radiological tests in this scenario. |
| **100** | An 84 year old man develops profuse diarrhoea whilst in hospital. An outbreak of Clostridioides (Clostridium) difficile has occurred in his ward.  Which feature of this organism makes it particularly difficult to destroy?  A. Motility  B. Outer capsule  C. Rapid mutation  D. Spore formation  E. Surface adherence | Attempt 1  The feature of Clostridioides difficile (Clostridium difficile) that makes it particularly difficult to destroy is:  D. Spore formation  Clostridioides difficile is a bacterium that can form spores, which are a dormant and highly resistant form of the organism. These spores can survive in the environment for extended periods and are resistant to many disinfectants and antibiotics. Spore formation allows the bacterium to withstand harsh conditions, including heat, desiccation, and chemical exposure. This resilience contributes to the persistence and transmission of Clostridioides difficile within healthcare settings, making it challenging to eradicate and control the spread of the infection. Proper cleaning and disinfection practices, along with effective hand hygiene, are crucial in preventing the transmission of Clostridioides difficile in healthcare facilities. |
|  |  | Attempt 2  The feature of Clostridioides (Clostridium) difficile that makes it particularly difficult to destroy is:  D. Spore formation  Clostridioides difficile is capable of forming spores, which are highly resistant structures that can survive in the environment for extended periods of time. Spores are resistant to heat, chemicals, and many disinfectants, making them challenging to eliminate. When conditions become unfavorable for growth, the bacterium can form spores as a survival strategy. These spores are the infectious form of the bacterium and can be a source of transmission and reinfection in healthcare settings. Spore formation is a critical factor contributing to the persistence and spread of C. difficile in the hospital environment and its role in recurrent infections in susceptible individuals. |
|  |  | Attempt 3  The feature of Clostridioides (Clostridium) difficile that makes it particularly difficult to destroy is D. Spore formation.  Clostridioides difficile is a bacterium that can form spores, which are highly resistant to various environmental conditions, including heat, chemicals, and antibiotics. These spores are capable of surviving in the environment for long periods of time, allowing them to persist and contribute to the transmission and recurrence of C. difficile infections.  Spore formation is an adaptive mechanism that enables the bacterium to withstand harsh conditions and survive unfavorable environments. It allows C. difficile to persist on surfaces, equipment, and healthcare settings, making it challenging to eliminate and control the spread of the organism.  Efforts to prevent and manage C. difficile infections typically involve strict infection control measures, including thorough cleaning and disinfection of surfaces, proper hand hygiene, and appropriate use of antibiotics to minimize the risk of spore formation, transmission, and infection. |

Paper 2

| **Question no.** | **Input** | **Output** |
| --- | --- | --- |
| **1** | A 25 year old man has had penile pain for two days. His most recent sexual intercourse was one week ago. There are multiple tender ulcers on his preputial skin. His penile and scrotal skin does not have any other abnormality.  Which is the most likely diagnosis?  A. Genital warts  B. Gonorrhoea  C. Herpes simplex  D. Lymphogranuloma venereum  E. Syphilis | Attempt 1  Based on the information provided, the most likely diagnosis is C. Herpes simplex.  Herpes simplex virus (HSV) infection commonly presents with painful ulcers or sores on the genital area. These ulcers can be tender and are typically associated with other symptoms such as burning sensation, itching, and flu-like symptoms (fever, headache, muscle aches) during the initial outbreak. The ulcers in this case are described as being on the preputial skin, which is consistent with genital herpes.  The other options can be ruled out based on the specific information provided:  A. Genital warts usually appear as small, soft, flesh-colored growths and are not typically painful.  B. Gonorrhea may cause urethral discharge and pain while urinating, but it does not usually present with ulcers.  D. Lymphogranuloma venereum (LGV) is a rare sexually transmitted infection caused by certain strains of Chlamydia trachomatis. It can cause genital ulcers, but it is less common than HSV infection.  E. Syphilis can also cause genital ulcers, but these are usually painless and have a distinct appearance (hard, round, and clean-edged) different from the tender ulcers described in the scenario.  It's important to note that this is a hypothetical case, and a definitive diagnosis should be made by a healthcare professional after a thorough examination and appropriate testing. If someone is experiencing genital symptoms or is concerned about their sexual health, they should seek medical attention promptly for an accurate diagnosis and appropriate treatment. |
|  |  | Attempt 2  Based on the provided information, the most likely diagnosis for the 25-year-old man with penile pain and multiple tender ulcers on his preputial skin is:  C. Herpes simplex  Herpes simplex virus (HSV) infection commonly presents with painful genital ulcers. The presence of multiple tender ulcers is consistent with genital herpes. Other symptoms of genital herpes can include itching, burning sensation, and flu-like symptoms such as fever and swollen lymph nodes. The lesions in herpes simplex are typically vesicles or ulcers with a red base and may be accompanied by local lymphadenopathy.  It is important for the individual to seek medical attention for proper diagnosis and management of genital herpes. Antiviral medications can be prescribed to alleviate symptoms, reduce the duration of outbreaks, and prevent transmission to sexual partners. |
|  |  | Attempt 3  Based on the description provided, the most likely diagnosis for the 25-year-old man with penile pain and multiple tender ulcers on his preputial skin is C. Herpes simplex.  Genital warts (A) typically present as raised, fleshy growths or bumps, rather than ulcers. Gonorrhoea (B) can cause urethritis and discharge, but it is less likely to present as multiple ulcers. Lymphogranuloma venereum (D) usually presents with painless genital ulcers that can progress to inguinal lymphadenopathy and abscess formation. Syphilis (E) typically presents as a painless ulcer called a chancre.  Herpes simplex (C), specifically genital herpes, commonly presents with painful, grouped vesicles or ulcers on the genital or perianal area. The ulcers are often shallow, surrounded by erythema, and can be quite tender. Recurrent outbreaks may occur, characterized by a cluster of vesicles followed by ulceration. It is important to note that herpes simplex can be sexually transmitted and may have periodic reactivations even after the initial infection.  It is recommended that the individual seek medical evaluation and testing for a definitive diagnosis and appropriate management. |
| **2** | A 60 year old man has increasing left hip pain. It is worse after exercise and occasionally wakes him at night. He has stiffness in the mornings that lasts for about 10 minutes. He has hypertension treated with ramipril, but is otherwise in good health. He has reduced internal rotation on passive movement. This also elicits pain.  Which is the most appropriate initial treatment for his pain?  A. Celecoxib  B. Codeine phosphate  C. Naproxen  D. Paracetamol  E. Tramadol | Attempt 1  The most appropriate initial treatment for the pain in the 60-year-old man with left hip pain and reduced internal rotation is:  D. Paracetamol  Paracetamol (also known as acetaminophen) is a first-line analgesic that can be used to relieve mild to moderate pain. It is generally safe and well-tolerated when used at recommended doses. Paracetamol can help reduce pain and inflammation associated with conditions such as osteoarthritis, which may be causing the hip pain in this case.  It is important to note that this recommendation is based on the provided information, and it is always advisable to consult a healthcare professional for an accurate diagnosis and personalized treatment plan. They can assess the severity of the pain, consider any contraindications or interactions with the patient's current medications, and provide appropriate guidance for pain management. |
|  |  | Attempt 2  Based on the information provided, the most appropriate initial treatment for the 60-year-old man's hip pain is D. Paracetamol (also known as acetaminophen).  The patient's symptoms, such as increasing left hip pain, worsened pain after exercise, nighttime awakening due to pain, and morning stiffness lasting for about 10 minutes, are indicative of osteoarthritis. Reduced internal rotation and pain on passive movement further support this diagnosis.  For the initial management of osteoarthritis pain, non-pharmacological interventions such as exercise, physical therapy, and weight management are typically recommended. Additionally, acetaminophen is usually considered the first-line analgesic for osteoarthritis pain due to its safety profile and effectiveness in managing mild to moderate pain. It is generally well-tolerated and has fewer gastrointestinal side effects compared to nonsteroidal anti-inflammatory drugs (NSAIDs).  The other options listed:  A. Celecoxib: This is an NSAID that can be used for pain relief in osteoarthritis, but it is not the most appropriate initial treatment, especially considering the patient's age and other health conditions.  B. Codeine phosphate: Opioid medications like codeine are not the first-line treatment for osteoarthritis pain due to their potential for side effects and risk of dependency.  C. Naproxen: Naproxen is an NSAID, and while it can be effective for osteoarthritis pain, it may have a higher risk of gastrointestinal side effects compared to acetaminophen, making it less suitable as the initial treatment.  E. Tramadol: Tramadol is an opioid analgesic and is generally reserved for cases where other pain relief options have not been effective or are not well-tolerated. It is not typically the first-choice treatment for osteoarthritis pain.  As always, it's essential for the patient to consult with a healthcare professional to confirm the diagnosis and receive personalized treatment recommendations based on their individual health status and medical history. |
|  |  | Attempt 3  Based on the description provided, the most appropriate initial treatment for the 60-year-old man with increasing left hip pain and reduced internal rotation is D. Paracetamol (acetaminophen).  Paracetamol is a commonly used first-line analgesic for mild to moderate pain. It is generally well-tolerated and has a good safety profile when used at recommended doses. Paracetamol can help alleviate pain and reduce inflammation associated with conditions such as osteoarthritis, which could be a possible cause of the patient's symptoms.  The other options listed are:  A. Celecoxib: Celecoxib is a nonsteroidal anti-inflammatory drug (NSAID) that can help relieve pain and inflammation. However, it is typically reserved for cases where paracetamol or other NSAIDs are insufficient or contraindicated due to potential side effects.  B. Codeine phosphate: Codeine is an opioid analgesic that can be used for pain relief. However, it is not typically the first-line treatment for chronic musculoskeletal pain due to its potential for side effects and risk of dependence.  C. Naproxen: Naproxen is an NSAID that can help reduce pain and inflammation. Like celecoxib, it is generally used when paracetamol or other initial measures are ineffective or contraindicated.  E. Tramadol: Tramadol is an opioid analgesic that is occasionally used for moderate to severe pain. It is typically reserved for cases where other analgesics have not provided adequate relief.  It is important for the patient to consult with a healthcare professional for a proper evaluation and individualized treatment plan based on their specific condition and medical history. |
| **3** | An 18 year old woman is 19 weeks pregnant. An ultrasound scan shows her baby has anencephaly. She wants to discuss her options with regards to termination of pregnancy.  When will termination of this pregnancy be legally permitted?  A. After spontaneous fetal death  B. At any time during the pregnancy  C. Before 20 weeks' gestation  D. Before 24 weeks' gestation  E. Before 28 weeks' gestation | Attempt 1  The legal requirements for termination of pregnancy vary by country and jurisdiction. It is important to note that I can provide general information, but specific laws may differ based on the location.  In the United Kingdom, for example, the legal framework allows for termination of pregnancy under specific circumstances. According to the Abortion Act 1967, termination is generally permitted up to 24 weeks of gestation if the continuance of the pregnancy would pose a risk to the woman's physical or mental health, or if there is a substantial risk of severe fetal abnormalities.  Based on this information, the most appropriate answer would be:  D. Before 24 weeks' gestation  However, it is crucial for the woman to consult with a healthcare professional or an abortion service provider to understand the specific legal requirements and options available to her in her country or region. Laws and regulations may vary, and it is important to receive accurate and up-to-date information in such circumstances. |
|  |  | Attempt 2  In the United Kingdom, the legal requirements for termination of pregnancy are specified under the Abortion Act 1967, as amended by the Human Fertilisation and Embryology Act 1990. According to the law, the most appropriate option for termination in the case of anencephaly is:  D. Before 24 weeks' gestation  In the UK, abortions can be performed legally up to 24 weeks of gestation, provided certain conditions are met, including the certification by two doctors that continuing the pregnancy would pose a risk to the woman's physical or mental health, or if there is a risk that the child will be born with a severe disability, like anencephaly.  After 24 weeks of gestation, termination of pregnancy is generally only allowed if there are severe fetal abnormalities or if there is a risk to the life of the pregnant woman or serious risk to her physical or mental health. It's essential for the woman to discuss her options and concerns with her healthcare provider and receive appropriate support and counseling throughout the process. |
|  |  | Attempt 3  In the UK, termination of pregnancy for fetal abnormalities, such as anencephaly, is legally permitted. According to the Abortion Act 1967, termination can be performed before 24 weeks of gestation. This means that the termination of the pregnancy would be legally permitted before reaching 24 weeks' gestation. It's important for the woman to consult with a healthcare professional or an abortion service provider to discuss her options and the specific procedures involved. |
| **4** | A 65 year old man has had constant back and right knee pain for 6 months, partially relieved by analgesia. He is an ex-smoker. He is otherwise well. Investigations: Haemoglobin 132 g/L (130-175) Alkaline phosphatase 850 IU/L (25-115) Calcium 2.3 mmol/L (2.2-2.6) Phosphate 0.9 mmol/L (0.8-1.5) Prostate specific antigen 5 μg/L (<4.0) X-rays: sclerotic areas in lower right femur and lumbar spine L3 Isotope bone scan: areas of increased uptake in femur, lumbar spine and skull  Which is the most likely diagnosis?  A. Hyperparathyroidism  B. Metastatic carcinoma of prostate  C. Metastatic small cell carcinoma of lung  D. Myeloma  E. Paget's disease of bone | Attempt 1  The most likely diagnosis based on the given information is:  B. Metastatic carcinoma of prostate  The patient's age, back pain, elevated alkaline phosphatase, and the presence of sclerotic lesions on X-ray and increased uptake on bone scan are indicative of metastatic bone disease. In men, prostate cancer is one of the most common causes of skeletal metastases. The elevated prostate-specific antigen (PSA) level further supports this diagnosis.  While other conditions such as hyperparathyroidism, metastatic small cell carcinoma of the lung, myeloma, and Paget's disease of bone can also cause bone abnormalities, the combination of the patient's clinical presentation, elevated PSA, and imaging findings strongly suggests metastatic carcinoma of the prostate as the most likely cause. Further evaluation and confirmation through additional diagnostic tests, such as biopsy or imaging of the prostate, may be necessary. |
|  |  | Attempt 2  Based on the information provided, the most likely diagnosis for the 65-year-old man's symptoms and investigation findings is:  E. Paget's disease of bone  Paget's disease of bone is a chronic disorder that involves abnormal bone remodeling, leading to the enlargement and weakening of the affected bones. The disease can cause bone pain and deformities. The characteristic X-ray findings of sclerotic areas in the lower right femur and lumbar spine (L3) and the areas of increased uptake in the femur, lumbar spine, and skull on the isotope bone scan are consistent with Paget's disease.  The elevated alkaline phosphatase level in the blood is a common finding in Paget's disease, as the condition involves increased bone turnover, leading to higher levels of this enzyme. Other laboratory findings, such as the normal calcium and phosphate levels, can help differentiate Paget's disease from other bone disorders.  The other options can be ruled out based on the specific information provided:  A. Hyperparathyroidism usually presents with hypercalcemia (high calcium levels) and may have different X-ray and bone scan findings.  B. Metastatic carcinoma of the prostate typically causes bone pain due to bone metastases, but the alkaline phosphatase level is not typically elevated in this condition.  C. Metastatic small cell carcinoma of the lung may also cause bone metastases and elevated alkaline phosphatase, but it usually presents with other symptoms, such as respiratory issues.  D. Myeloma (multiple myeloma) can cause bone pain and increased alkaline phosphatase, but it would present with other characteristic findings on laboratory tests, such as elevated levels of monoclonal proteins and abnormal plasma cells in the bone marrow.  It's important to note that this is a hypothetical case, and a definitive diagnosis should be made by a healthcare professional based on a comprehensive evaluation, including imaging studies, laboratory tests, and clinical examination. The patient should be referred to an appropriate specialist for further assessment and management. |
|  |  | Attempt 3  Based on the provided clinical information and investigation findings, the most likely diagnosis in this case is metastatic carcinoma of the prostate (option B). The patient's age, back and knee pain, elevated alkaline phosphatase, and the presence of sclerotic lesions on X-rays and increased uptake on bone scan are consistent with metastatic bone involvement. The elevated prostate-specific antigen (PSA) level also suggests prostate involvement. Prostate cancer commonly metastasizes to bones, and the clinical and investigation findings support this diagnosis. Further evaluation and management should be conducted by a healthcare professional specializing in oncology. |
| **5** | A 14 year old girl has had crampy abdominal pain and poor appetite for 24 hours. She has also had a sore throat for 3 days. Her temperature is 38.5°C. There is cervical lymphadenopathy and tenderness in the right iliac fossa.  Which is the most likely diagnosis?  A. Acute appendicitis  B. Crohn's ileitis  C. Meckel's diverticulum  D. Mesenteric adenitis  E. Ovarian cyst | Attempt 1  Based on the given information, the most likely diagnosis is:  D. Mesenteric adenitis  The patient's symptoms of crampy abdominal pain, poor appetite, sore throat, and fever, along with cervical lymphadenopathy and tenderness in the right iliac fossa, are suggestive of mesenteric adenitis. Mesenteric adenitis is the inflammation of the lymph nodes in the mesentery, commonly caused by viral or bacterial infections, such as a preceding upper respiratory tract infection. The symptoms can mimic appendicitis, but in mesenteric adenitis, the pain is typically more diffuse and not localized to the right lower quadrant.  Acute appendicitis (choice A) is a possibility given the tenderness in the right iliac fossa, but the absence of focal tenderness, along with the presence of cervical lymphadenopathy, makes mesenteric adenitis a more likely diagnosis.  Crohn's ileitis (choice B), Meckel's diverticulum (choice C), and ovarian cyst (choice E) are less likely based on the given information and are not commonly associated with the combination of symptoms described.  It is important to consult a healthcare professional for a thorough evaluation and accurate diagnosis in this case. |
|  |  | Attempt 2  Based on the information provided, the most likely diagnosis for the 14-year-old girl's symptoms is:  D. Mesenteric adenitis  Mesenteric adenitis is an inflammatory condition characterized by enlarged and inflamed lymph nodes in the mesentery (the tissue that attaches the intestines to the abdominal wall). It often presents with abdominal pain, tenderness in the right iliac fossa (lower right side of the abdomen), and sometimes a low-grade fever. It can be associated with other symptoms like a sore throat, as in this case.  The symptoms of mesenteric adenitis can be similar to acute appendicitis, but the absence of significant appetite loss and the presence of cervical lymphadenopathy (enlarged lymph nodes in the neck) make mesenteric adenitis a more likely diagnosis.  The other options can be ruled out based on the specific information provided:  A. Acute appendicitis usually presents with persistent and worsening abdominal pain, often starting around the umbilicus and moving to the right lower quadrant. It may be associated with vomiting, anorexia, and a higher fever.  B. Crohn's ileitis typically presents with chronic abdominal pain, diarrhea, weight loss, and sometimes fever. It is less likely to present acutely with fever and acute abdominal pain.  C. Meckel's diverticulum is a congenital anomaly that can cause abdominal pain, but it is usually not associated with symptoms like sore throat and cervical lymphadenopathy.  E. Ovarian cysts can cause abdominal pain, but they are less likely to present with the combination of sore throat, cervical lymphadenopathy, and right iliac fossa tenderness.  As always, a definitive diagnosis should be made by a healthcare professional after a thorough examination and appropriate testing. If the girl's symptoms are concerning, she should be evaluated by a doctor for a proper diagnosis and management. |
|  |  | Attempt 3  The most likely diagnosis in this case is mesenteric adenitis (option D). Mesenteric adenitis is an inflammatory condition characterized by enlarged lymph nodes in the mesentery of the abdomen, typically caused by viral or bacterial infections. The patient's symptoms of crampy abdominal pain, sore throat, and cervical lymphadenopathy are consistent with this condition. Mesenteric adenitis can often mimic acute appendicitis due to the similar presentation of right lower quadrant pain and tenderness. However, in mesenteric adenitis, the pain is usually less severe and localized to the right iliac fossa. Additionally, the presence of a sore throat suggests an upper respiratory infection as the underlying cause. Further evaluation by a healthcare professional, including physical examination and additional tests if necessary, can help confirm the diagnosis. |
| **6** | The links between deprivation and health inequalities are well evidenced and widely accepted, but there are a number of explanations for that association. One explanation proposes that ill health determines people's social class.  Which type of explanation of health inequality is this?  A. Artefact  B. Cultural/behavioural  C. Idealist  D. Materialist  E. Social selection/mobility | Attempt 1  The type of explanation of health inequality that proposes ill health determines people's social class is:  E. Social selection/mobility  The social selection/mobility explanation suggests that individuals' health status influences their social class or position in society. According to this perspective, individuals who experience poor health are more likely to have limited opportunities for upward social mobility and may be more likely to remain or fall into lower social classes. Ill health can lead to reduced educational attainment, limited employment opportunities, and lower income, all of which contribute to lower social class status.  It is important to note that health inequalities are complex and multifactorial, and various explanations, including artefact, cultural/behavioural, idealist, and materialist, can also contribute to the understanding of health inequalities. |
|  |  | Attempt 2  The type of explanation of health inequality that proposes that ill health determines people's social class is:  E. Social selection/mobility  The social selection/mobility explanation suggests that individuals' health status can influence their social class or position in society. In this context, it means that individuals experiencing ill health may face barriers in terms of education, employment opportunities, and social mobility, which can result in lower socioeconomic status or class. Health issues can limit people's ability to access resources and opportunities, leading to lower socioeconomic outcomes and perpetuating health inequalities. This explanation is sometimes also referred to as the "health selection" hypothesis.  The other options refer to different types of explanations for health inequalities:  A. Artefact: This explanation suggests that health inequalities are an artifact or result of measurement errors, biases, or inaccuracies in the data rather than actual differences in health outcomes.  B. Cultural/behavioural: This explanation attributes health inequalities to cultural or behavioral factors, such as lifestyle choices, health behaviors, or health-related beliefs and practices.  C. Idealist: This explanation focuses on ideas, values, and beliefs as the primary drivers of health inequalities. It emphasizes the influence of societal norms and ideologies on health outcomes.  D. Materialist: The materialist explanation of health inequalities emphasizes socioeconomic factors, such as income, education, employment, and access to resources, as the key determinants of health disparities. It highlights the impact of social and economic inequalities on health outcomes.  Health inequalities are complex and multifactorial, often arising from a combination of these explanations, including social, economic, cultural, and individual factors. Understanding the interplay of these factors is crucial for developing effective strategies to address health disparities and promote health equity. |
|  |  | Attempt 3  The explanation that ill health determines people's social class is a form of social selection/mobility (option E) explanation of health inequality. According to this perspective, individuals who experience poor health are more likely to face social and economic disadvantages, leading to lower social class positions. Illness or health problems can hinder educational attainment, limit job opportunities, and result in reduced income and social mobility. This explanation suggests that health outcomes contribute to the social stratification of individuals rather than the other way around. |
| **7** | A 19 year old man has 1 day of fever, muscle stiffness, palpitations and difficulty breathing. He has a history of psychotic depression. He has been taking fluoxetine for 6 weeks and started taking risperidone 1 week ago. He is agitated and distressed. His temperature is 39.8°C, pulse rate 114 bpm, BP 172/89 mmHg and respiratory rate 30 breaths per minute. Chest examination is otherwise normal. There is increased tone globally; there are no other neurological abnormalities.  Which test is most likely to help confirm the diagnosis?  A. CT scan of head  B. ECG  C. EEG  D. Lumbar puncture  E. Serum creatine kinase | Attempt 1  Given the clinical presentation of fever, muscle stiffness, palpitations, and difficulty breathing, along with the increased tone and agitation, the most likely diagnosis in this case is serotonin syndrome. Serotonin syndrome is a potentially life-threatening condition caused by excessive serotonin activity in the central nervous system. It can occur as a result of the use of serotonergic medications, such as fluoxetine and risperidone, especially when used in combination.  To confirm the diagnosis of serotonin syndrome and rule out other possible causes, the most appropriate test would be:  E. Serum creatine kinase (CK)  Elevated levels of serum creatine kinase can indicate muscle damage, which is commonly observed in serotonin syndrome. The increased muscle tone and stiffness seen in the patient's presentation further support the suspicion of serotonin syndrome. Other diagnostic tests may be performed based on the clinical context and to rule out other potential causes, but an elevated CK level can help confirm the diagnosis of serotonin syndrome. |
|  |  | Attempt 2  Based on the provided information, the most likely test to help confirm the diagnosis for the 19-year-old man's symptoms is:  E. Serum creatine kinase (CK) level measurement  The patient's symptoms, including fever, muscle stiffness, palpitations, difficulty breathing, and increased tone (muscle rigidity), are concerning for a potential adverse drug reaction called serotonin syndrome. Serotonin syndrome can occur when there is an excess of serotonin in the central nervous system, often due to the use of medications that increase serotonin levels or enhance its effects.  In this case, the patient has been taking fluoxetine (a selective serotonin reuptake inhibitor, or SSRI) for 6 weeks and recently started risperidone (an antipsychotic), both of which can contribute to increased serotonin levels. Serotonin syndrome can present with a variety of symptoms, including fever, altered mental status, autonomic instability (e.g., increased heart rate and blood pressure), and neuromuscular abnormalities like muscle stiffness and increased tone.  Measuring the serum creatine kinase (CK) level is important in this context because serotonin syndrome can lead to muscle breakdown (rhabdomyolysis), which can significantly elevate CK levels in the blood. An elevated CK level would support the diagnosis of serotonin syndrome and help differentiate it from other conditions.  The other options may be relevant in certain situations, but they are less likely to be the most useful test to confirm the diagnosis in this specific case:  A. CT scan of the head: This test is used to evaluate brain structure and is not likely to be the most relevant in confirming serotonin syndrome.  B. ECG: An electrocardiogram (ECG) is useful to assess heart rhythm and function, but it may not be the most specific test to confirm serotonin syndrome.  C. EEG: An electroencephalogram (EEG) records brain electrical activity and may be used to assess seizure activity, but it is not the most specific test for serotonin syndrome.  D. Lumbar puncture: This procedure involves obtaining cerebrospinal fluid (CSF) from the spinal canal and is usually performed to evaluate central nervous system infections or other neurological conditions. It is not a primary test for serotonin syndrome.  Serotonin syndrome is a medical emergency, and if this diagnosis is suspected, the patient should receive immediate medical attention for appropriate management and treatment. It's essential to discontinue the medications contributing to the syndrome and provide supportive care as necessary. |
|  |  | Attempt 3  Given the symptoms described, the most appropriate test to help confirm the diagnosis in this case is the serum creatine kinase (CK) level measurement (option E). The patient's presentation of fever, muscle stiffness, and agitation, along with the use of risperidone, suggests the possibility of neuroleptic malignant syndrome (NMS). NMS is a rare but potentially life-threatening condition associated with the use of antipsychotic medications, such as risperidone. Elevated serum CK levels are commonly observed in NMS due to muscle breakdown. Confirming elevated CK levels would support the diagnosis of NMS and help guide further management. Other diagnostic tests may be considered based on the clinical context and the need to rule out alternative conditions, but CK measurement is a key initial investigation for suspected NMS. |
| **8** | N/A | Attempt 1 |
|  |  | Attempt 2 |
|  |  | Attempt 3 |
| **9** | A 30 year old man goes out gambling. One night he makes a profit of £100,000, and the following night he loses £150,000.  Which psychological process most likely occurred on the first night that led to his risk taking on the following night?  A. Classical conditioning  B. Disinhibition  C. Modelling  D. Positive reinforcement  E. Social learning | Attempt 1  The psychological process that most likely occurred on the first night and led to the risk-taking behavior on the following night is:  D. Positive reinforcement  Positive reinforcement refers to the process by which a behavior is strengthened or increased due to the addition of a positive stimulus or reward. In this case, the man experienced a profit of £100,000 on the first night of gambling, which likely reinforced his behavior and provided a positive experience. This positive reinforcement may have increased his motivation to continue gambling and take risks, leading to the loss of £150,000 on the following night.  It's important to note that gambling can be associated with addictive behavior and can have negative consequences. The individual's behavior in this scenario may indicate a problematic pattern of gambling and should be approached with caution. |
|  |  | Attempt 2  The most likely psychological process that occurred on the first night that led to his risk-taking on the following night is:  D. Positive reinforcement  Positive reinforcement occurs when a behavior is followed by a rewarding stimulus, leading to an increased likelihood of that behavior being repeated in the future. In this case, the man made a profit of £100,000 on the first night of gambling, which can be considered a positive reinforcement. The pleasurable experience of winning money acted as a reward for his gambling behavior, making him more likely to engage in risk-taking behavior again, hoping for another positive outcome.  The loss of £150,000 on the following night could have triggered negative emotions and cognitive biases, such as the "loss-chasing" phenomenon, where the individual tries to recoup their losses by taking even greater risks. This behavior can be motivated by the memory of the positive reinforcement experienced during the first night's winnings.  It's important to note that gambling behavior can be complex and influenced by various psychological and environmental factors. Risk-taking behaviors in gambling can also be affected by other processes like cognitive biases, arousal seeking, and irrational beliefs about luck and chance.  While the other options may have relevance in certain psychological contexts, they are not as directly related to the described scenario of gambling and risk-taking:  A. Classical conditioning: This process involves the association between a neutral stimulus and a reflexive response. It may not be the most relevant process in explaining risk-taking behavior during gambling.  B. Disinhibition: This refers to a reduction in inhibitions, leading to an increase in impulsive behavior. While it could play a role in some gambling behaviors, it does not specifically explain the described scenario.  C. Modelling: This involves learning by observing others' behavior and imitating it. While it can influence risk-taking behavior in some cases, it does not directly explain the man's risk-taking after winning and losing money in gambling.  E. Social learning: This is similar to modeling and involves learning from others through observation, but it is not the most specific process for explaining the risk-taking behavior described in the scenario. |
|  |  | Attempt 3  The psychological process most likely involved in this scenario is D. Positive reinforcement.  When the individual made a profit of £100,000 on the first night, it likely resulted in a positive emotional response and a sense of reward. This positive reinforcement can strengthen the association between gambling and the pleasurable outcome of winning, leading to an increased motivation to engage in risk-taking behavior again.  The positive reinforcement from the initial success may have influenced the individual's decision-making on the following night, leading to increased risk-taking behavior despite the subsequent loss of £150,000. The expectation of another positive outcome, based on the previous reinforcement, may have overridden the potential negative consequences and encouraged further gambling.  It's important to note that gambling behavior can be influenced by various psychological processes and individual factors, and this explanation focuses on the role of positive reinforcement in shaping subsequent risk-taking behavior. |
| **10** | A 85 year old woman has a breast lump. She has hypertension, heart failure and a previous stroke. Her medications are bisoprolol, ramipril, furosemide, aspirin and atorvastatin. Her pulse is 76 bpm irregularly irregular and BP 105/70 mmHg. Her jugular venous pressure is visible 6 cm above the sternal angle. She has bibasal late inspiratory crepitations and bilateral pedal oedema. There is a 2.5 cm diameter, mobile, hard, non tender swelling in the upper outer quadrant of her breast. There is no palpable lymphadenopathy. Investigation: Pathology report of breast biopsy: infiltrating ductal carcinoma, ER positive, HER2 negative She is started on an aromatase inhibitor and bisphosphonate.  Which is the most appropriate next treatment to offer?  A. Chemotherapy  B. Immunotherapy  C. No additional treatment  D. Radiotherapy  E. Surgery | Attempt 1  Given the information provided, the most appropriate next treatment to offer for an 85-year-old woman with infiltrating ductal carcinoma, ER positive, HER2 negative breast cancer would depend on several factors, including the stage of the cancer, the overall health and functional status of the patient, and the patient's preferences. However, based on the information provided, the most appropriate next treatment to offer would likely be:  E. Surgery  Surgery is a common treatment option for breast cancer and is typically recommended for the removal of the tumor. In this case, since the breast lump is described as a 2.5 cm diameter, mobile, hard, non-tender swelling, surgical excision of the tumor (breast-conserving surgery or mastectomy) would be a suitable treatment option.  It's important to note that the final decision on treatment should be made after a thorough evaluation by the healthcare team, considering the patient's individual circumstances and preferences. Additional treatments such as chemotherapy, radiotherapy, or targeted therapies may be recommended based on the specific characteristics of the tumor and the overall treatment plan. |
|  |  | Attempt 2  Given the information provided, the most appropriate next treatment to offer for the 85-year-old woman with infiltrating ductal carcinoma, ER positive, HER2 negative breast cancer is:  E. Surgery  Since the patient has a 2.5 cm diameter, mobile, hard, non-tender breast lump, and there is no palpable lymphadenopathy, surgical intervention is typically the first-line treatment for localized breast cancer. The primary aim of surgery is to remove the tumor and surrounding tissue to achieve local control and reduce the risk of recurrence. Depending on the extent of the disease, various surgical options may be considered, such as lumpectomy (removal of the tumor and a margin of surrounding tissue) or mastectomy (removal of the entire breast).  After surgery, additional treatments like radiation therapy, chemotherapy, and/or hormonal therapy may be considered based on the specific characteristics of the tumor and the patient's overall health. However, the next immediate step for this patient with localized breast cancer would typically involve surgical evaluation and intervention. |
|  |  | Attempt 3  Based on the given information, the most appropriate next treatment to offer for the 85-year-old woman with infiltrating ductal carcinoma of the breast would be E. Surgery.  Surgery is a standard treatment option for breast cancer and is typically the primary approach for local control of the disease. In this case, the presence of a 2.5 cm diameter, mobile, hard, non-tender breast lump suggests a localized tumor that is potentially resectable.  The specific type of surgical procedure would depend on various factors, such as the size and location of the tumor, as well as the patient's overall health and preferences. Options may include breast-conserving surgery (lumpectomy) or mastectomy.  It's important to note that treatment decisions are best made in consultation with a multidisciplinary team of healthcare professionals, including surgeons, oncologists, and other specialists, taking into consideration the individual patient's circumstances, preferences, and any additional diagnostic information. |
| **11** | A 40 year old primigravid woman, at 28 weeks' gestation, is admitted with a seizure. She had epigastric pain and blurred vision preceding the seizure. Her BP is 155/105 mmHg. Reflexes are brisk with two beats of ankle clonus. Her urinalysis shows protein 2+.  Which intravenous drug is the most appropriate immediate treatment?  A. Diazepam  B. Hydralazine  C. Levetiracetam  D. Magnesium sulfate  E. Phenytoin | Attempt 1  In this scenario, the most appropriate immediate treatment for a pregnant woman presenting with seizures and signs of severe preeclampsia/eclampsia is:  D. Magnesium sulfate  Magnesium sulfate is the drug of choice for preventing and managing seizures (eclampsia) in pregnant women. It is a central nervous system depressant and acts as an anticonvulsant. Magnesium sulfate has been shown to be effective in reducing the risk of recurrent seizures and improving maternal and fetal outcomes in cases of preeclampsia/eclampsia.  Therefore, the immediate administration of magnesium sulfate intravenously is recommended to control and prevent further seizures. Other treatments, such as antihypertensive medication (e.g., hydralazine) or antiepileptic drugs (e.g., phenytoin), may be considered as adjunctive therapy but are not the primary treatment for eclamptic seizures.  It's important to note that the final treatment plan should be determined by the healthcare team based on the patient's individual circumstances and response to treatment. |
|  |  | Attempt 2  In this scenario, the 40-year-old primigravid woman at 28 weeks' gestation is presenting with signs and symptoms suggestive of severe preeclampsia or eclampsia. Given her elevated blood pressure, epigastric pain, blurred vision, proteinuria (2+ on urinalysis), and the occurrence of a seizure (eclampsia), the most appropriate immediate treatment would be:  D. Magnesium sulfate  Magnesium sulfate is the drug of choice for the prevention and treatment of eclamptic seizures. It is also used for seizure prophylaxis in severe preeclampsia. Magnesium sulfate helps prevent and control seizures by acting as a central nervous system depressant.  Options A, C, and E (Diazepam, Levetiracetam, Phenytoin) are also used to treat seizures, but they are not the first-line drugs of choice for eclampsia.  Option B (Hydralazine) is an antihypertensive medication commonly used to lower blood pressure in preeclampsia and eclampsia, but it is not the immediate treatment for preventing or stopping seizures.  In this critical situation, the priority is to manage the eclamptic seizure, and magnesium sulfate should be administered promptly under appropriate medical supervision. After stabilizing the patient, additional measures will be taken to manage her blood pressure and preeclampsia. |
|  |  | Attempt 3  In this scenario, the most appropriate immediate treatment for the 40-year-old primigravid woman with seizures, epigastric pain, and hypertension in the setting of pregnancy is D. Magnesium sulfate.  The clinical presentation, including hypertension, proteinuria (2+ on urinalysis), epigastric pain, and neurological symptoms (seizure, blurred vision, brisk reflexes with ankle clonus), is consistent with severe preeclampsia or eclampsia. Magnesium sulfate is the treatment of choice for preventing and treating seizures in women with eclampsia or severe preeclampsia.  Magnesium sulfate is administered intravenously as a loading dose followed by a maintenance infusion. It has anticonvulsant properties and helps to prevent recurrent seizures. It also has beneficial effects on blood pressure control and is the recommended treatment to prevent maternal morbidity and mortality associated with eclampsia.  It's important to note that treatment decisions should be made in consultation with a healthcare professional, and the specific dosages and monitoring should be based on the individual patient's condition and institutional guidelines. |
| **12** | A 29 year old woman visits her GP with a heavy sensation in her vagina when exercising. She is aware of a bulge in her vagina, sometimes associated with a desire to void urine. She had a vaginal delivery 2 years ago. There is some laxity of the anterior vaginal wall, but this does not descend to the introitus on straining. Urine culture is negative.  Which is the most appropriate management plan?  A. Oxybutinin hydrochloride  B. Pelvic floor exercises  C. Refer for urodynamic testing  D. Refer to urogynaecology clinic  E. Ultrasound scan of pelvis | Attempt 1  Based on the given information, the most appropriate management plan for the 29-year-old woman with a sensation of vaginal heaviness and a bulge in her vagina is:  D. Refer to urogynaecology clinic  The symptoms described suggest pelvic organ prolapse, specifically anterior vaginal wall prolapse (cystocele). This condition is common after vaginal delivery and is characterized by the descent of the bladder into the anterior vaginal wall.  Referring the patient to a urogynaecology clinic is appropriate because specialized evaluation and management of pelvic organ prolapse are required. A urogynaecologist will perform a thorough assessment, which may include a pelvic examination, to determine the severity and extent of the prolapse. Further treatment options, such as pelvic floor exercises, pessary use, or surgical intervention, can then be discussed and implemented based on the patient's specific needs.  While pelvic floor exercises (option B) can be beneficial for managing mild cases of pelvic organ prolapse, it is important to involve a specialist for proper evaluation and individualized treatment planning in this case. Options A, C, and E are not the most appropriate initial management strategies for this particular condition. |
|  |  | Attempt 2  The most appropriate management plan for the 29-year-old woman with a sensation of heaviness in her vagina, associated with a bulge and a desire to void urine, and some laxity of the anterior vaginal wall is:  D. Refer to a urogynecology clinic  These symptoms are suggestive of pelvic organ prolapse, a condition in which the pelvic organs (such as the bladder, uterus, or rectum) descend or protrude into the vagina due to weakened pelvic floor muscles and ligaments. Given that the symptoms are bothering her during exercise and are affecting her quality of life, a referral to a urogynecology clinic is the best course of action. Urogynecologists specialize in managing conditions like pelvic organ prolapse and urinary incontinence.  Options A (Oxybutynin hydrochloride) and B (Pelvic floor exercises) may be considered in certain cases of urinary incontinence but are not the most appropriate first-line management for pelvic organ prolapse.  Option C (Refer for urodynamic testing) might be helpful in evaluating urinary symptoms in some cases, but it does not directly address the management of pelvic organ prolapse.  Option E (Ultrasound scan of pelvis) may have limited utility in assessing pelvic organ prolapse but is not the most appropriate first step in managing this condition. Clinical evaluation by a urogynecologist is essential for a comprehensive assessment and treatment plan. |
|  |  | Attempt 3  The most appropriate management plan for the 29-year-old woman with symptoms of a bulge in her vagina and a sensation of heaviness during exercise, along with laxity of the anterior vaginal wall, is D. Refer to urogynaecology clinic.  These symptoms are suggestive of pelvic organ prolapse, specifically anterior vaginal wall prolapse (cystocele). Given the presence of symptoms and physical findings, referral to a urogynaecology clinic is warranted for further evaluation and management. Urogynaecologists specialize in the diagnosis and treatment of pelvic floor disorders, including pelvic organ prolapse.  While pelvic floor exercises (option B) may be beneficial as part of conservative management for mild cases of pelvic organ prolapse, the presence of a bulge and significant symptoms suggest the need for specialist evaluation. Options A, C, and E are not the most appropriate initial steps in the management of this condition.  It's important for the patient to be evaluated by a specialist who can provide a comprehensive assessment, discuss treatment options, and determine the most appropriate management plan based on the severity of the prolapse and the patient's individual circumstances. |
| **13** | A 19 month old girl has been able to sit unsupported for 1 month. She can speak 11 words and feeds herself. She was born at 30 weeks' gestation.  Which is the best description of her current development?  A. Fine motor delay  B. Gross developmental delay  C. Gross motor delay  D. Mild developmental delay  E. Normal development | Attempt 1  Based on the given information, the best description of the 19-month-old girl's current development is:  E. Normal development  At 19 months old, the child is able to sit unsupported, speaks 11 words, and feeds herself. These achievements indicate age-appropriate development in gross motor skills, fine motor skills, and language development. It's worth noting that the child was born at 30 weeks' gestation, which is considered premature. However, the provided information does not indicate any specific developmental delays or concerns.  Therefore, based on the information given, the child's development is considered normal. |
|  |  | Attempt 2  Based on the information provided, the best description of the 19-month-old girl's current development is:  E. Normal development  She has achieved age-appropriate milestones such as sitting unsupported, saying 11 words, and feeding herself, which are all positive signs of normal development. Given that she was born at 30 weeks' gestation, it's important to consider her corrected age (adjusted for prematurity) when evaluating her developmental progress. Corrected age is calculated by subtracting the number of weeks of prematurity from her chronological age. At 19 months old, she would be around 17 months corrected age (19 months - 2 months of prematurity).  Considering her corrected age, sitting unsupported for 1 month, speaking 11 words, and feeding herself are age-appropriate milestones. Developmental milestones can have some variation between children, and her current achievements suggest she is developing well for her corrected age. However, it's always best to monitor her development closely and consult with a pediatrician if there are any concerns or if further developmental assessments are needed. |
|  |  | Attempt 3  Based on the given information, the best description of the 19-month-old girl's current development is E. Normal development.  At 19 months of age, it is expected that a child can sit unsupported, speak a few words, and begin to feed themselves. The ability to sit unsupported for 1 month, speaking 11 words, and self-feeding are all within the expected range for a child of this age.  The fact that she was born at 30 weeks' gestation does not necessarily indicate a developmental delay at this stage. Prematurity can have an impact on early development, but as the child grows older, the focus shifts more towards the child's current abilities and milestones rather than the prematurity itself.  Based on the given information, there is no indication of a delay in fine motor skills (option A), gross developmental skills (option B), or gross motor skills (option C). Therefore, the most appropriate description is that the child is demonstrating normal development (option E). |
| **14** | A 37 year old woman who is 10 weeks pregnant has had excessive vomiting for 1 week. The fundal height is consistent with 16 weeks' gestation. Her temperature is 37.2°C, pulse rate 100 bpm and BP 180/110 mmHg.  Which is the most likely diagnosis?  A. Gastroenteritis  B. Missed abortion  C. Molar pregnancy  D. Pre-eclampsia  E. Pregnancy-induced hypertension | Attempt 1  Based on the given information, the most likely diagnosis for the 37-year-old woman is:  D. Pre-eclampsia  Pre-eclampsia is a pregnancy complication characterized by high blood pressure (BP) and signs of damage to other organ systems, most commonly the liver and kidneys. It typically occurs after 20 weeks of gestation, but in rare cases, it can develop earlier. The woman's elevated BP (180/110 mmHg) and other symptoms such as excessive vomiting, increased fundal height (consistent with gestational age), and possible signs of hypertension indicate a potential diagnosis of pre-eclampsia.  Gastroenteritis, missed abortion, and molar pregnancy are less likely given the combination of symptoms and findings described. Pregnancy-induced hypertension is a term used to describe elevated blood pressure during pregnancy, but it is often used interchangeably with pre-eclampsia.  However, it's important to note that a definitive diagnosis can only be made by a healthcare professional based on a comprehensive evaluation, including further examination and diagnostic tests. The woman should seek medical attention for proper evaluation and management. |
|  |  | Attempt 2  The most likely diagnosis for the 37-year-old pregnant woman with excessive vomiting, fundal height consistent with 16 weeks' gestation, temperature of 37.2°C, pulse rate of 100 bpm, and blood pressure of 180/110 mmHg is:  D. Pre-eclampsia  Pre-eclampsia is a condition that typically occurs after 20 weeks of pregnancy and is characterized by high blood pressure (hypertension) and signs of damage to other organ systems, most commonly the liver and kidneys. It can also be associated with excessive vomiting (hyperemesis gravidarum), which can lead to dehydration and electrolyte imbalances.  The elevated blood pressure (BP 180/110 mmHg) and excessive vomiting are concerning signs that, when combined with the advanced fundal height, suggest the possibility of pre-eclampsia. Pre-eclampsia can lead to serious complications for both the mother and the baby if not properly managed.  Options A (Gastroenteritis) and B (Missed abortion) are less likely as they do not fully explain the combination of symptoms, including the elevated blood pressure and advanced fundal height.  Option C (Molar pregnancy) is not supported by the information provided. Molar pregnancies typically present with other signs, such as abnormal vaginal bleeding and a uterus larger than expected for gestational age.  Option E (Pregnancy-induced hypertension) is a similar condition to pre-eclampsia but is generally milder. Given the severity of the symptoms described in the case, pre-eclampsia is a more likely diagnosis. It's important for the woman to receive prompt medical attention and appropriate management to ensure the best outcome for her and the baby. |
|  |  | Attempt 3  Based on the given information, the most likely diagnosis for the 37-year-old woman is D. Pre-eclampsia.  Pre-eclampsia is a condition characterized by high blood pressure (BP) and organ damage, usually affecting pregnant women after 20 weeks of gestation. However, in some cases, it can occur earlier, as in this scenario. Excessive vomiting (hyperemesis gravidarum) can be associated with pre-eclampsia.  The woman's elevated BP of 180/110 mmHg, along with the presence of excessive vomiting and an increased fundal height inconsistent with the gestational age, raises suspicion for pre-eclampsia. Pre-eclampsia can also present with other symptoms such as headaches, visual disturbances, and edema.  While gastroenteritis (option A) can cause vomiting, it is less likely to cause a significantly increased fundal height or elevated BP. Missed abortion (option B) refers to the absence of fetal heartbeat and growth, which may not be related to the symptoms described. Molar pregnancy (option C) is a rare condition characterized by abnormal growth of placental tissue and is not typically associated with excessive vomiting. Pregnancy-induced hypertension (option E) is a term used for high BP during pregnancy without significant organ damage, while pre-eclampsia involves both high BP and organ damage.  Given the combination of symptoms and findings, pre-eclampsia is the most likely diagnosis. Prompt medical evaluation and management are necessary to ensure the well-being of the mother and the baby. |
| **15** | A 29 year old woman has pain and morning stiffness in her finger and wrist joints. This improves during the day and after active movement. She has had recurrent mouth ulcers for the last 2 years. She had an episode of pleuritic chest pain 6 months ago which resolved without seeking help. Her BP is 128/85 mmHg. She has no warmth or tenderness in her hands. Urinalysis: protein 1+, blood 1+ Investigations: Haemoglobin 109 g/L (115–150) White cell count 3.8 × 109/L (4.0–11.0) Lymphocytes 0.9 × 109/L (1.1–3.3) Platelets 160 × 109/L (150–400) Creatinine 90 μmol/L (60–120) CRP 21 mg/L (< 5)  Which is the most likely diagnosis?  A. Behçet's disease  B. Reactive arthritis  C. Rheumatoid arthritis  D. Sjögren's syndrome  E. Systemic lupus erythematosus | Attempt 1  Based on the provided information, the most likely diagnosis for the 29-year-old woman is C. Rheumatoid arthritis.  Rheumatoid arthritis is a chronic autoimmune disorder characterized by joint pain, stiffness, and swelling, typically affecting the small joints of the hands and wrists symmetrically. The presence of pain and morning stiffness in the finger and wrist joints, along with improvement during the day and after active movement, is consistent with the pattern seen in rheumatoid arthritis.  The woman's history of recurrent mouth ulcers, pleuritic chest pain, and urinalysis showing protein and blood in the urine indicate possible systemic involvement. These findings, along with an elevated CRP level, further support the diagnosis of rheumatoid arthritis.  While the other conditions listed may also present with joint symptoms, the combination of symptoms and findings described in this case is most suggestive of rheumatoid arthritis. Behçet's disease (option A) typically presents with recurrent mouth ulcers and genital ulcers, but it is less commonly associated with joint symptoms. Reactive arthritis (option B) is usually triggered by an infection, often involving the genitourinary or gastrointestinal tract, and is less likely to have the characteristic pattern of morning stiffness seen in this case. Sjögren's syndrome (option D) primarily affects the exocrine glands and commonly presents with dry eyes and dry mouth. Systemic lupus erythematosus (option E) can have various symptoms and systemic involvement, but joint pain and stiffness are often more symmetric and may not follow the pattern described in this case.  It is important for the patient to undergo further evaluation and receive appropriate management for rheumatoid arthritis, which may include medications to control inflammation and preserve joint function. |
|  |  | Attempt 2  The most likely diagnosis for the 29-year-old woman with pain and morning stiffness in her finger and wrist joints, recurrent mouth ulcers, a history of pleuritic chest pain, and the given laboratory findings is:  D. Sjögren's syndrome  Sjögren's syndrome is an autoimmune disorder that primarily affects the exocrine glands, leading to symptoms such as dry eyes and dry mouth (xerophthalmia and xerostomia). However, it can also present with joint pain and stiffness, especially affecting the small joints of the fingers and wrists. The improvement of symptoms with active movement is a characteristic feature of inflammatory joint conditions.  The recurrent mouth ulcers and pleuritic chest pain are also consistent with Sjögren's syndrome, as it can affect multiple organ systems.  While the other options (Behçet's disease, Reactive arthritis, Rheumatoid arthritis, and Systemic lupus erythematosus) might have some overlapping symptoms, the combination of joint symptoms, dry eyes, and dry mouth, along with the given laboratory findings, make Sjögren's syndrome the most likely diagnosis.  To confirm the diagnosis, further evaluation and additional tests, such as specific antibody tests and salivary gland biopsy, may be required. It's essential for the woman to see a rheumatologist for a comprehensive evaluation and appropriate management. |
|  |  | Attempt 3  Based on the given information, the most likely diagnosis for the 29-year-old woman is:  D. Sjögren's syndrome  Sjögren's syndrome is an autoimmune disorder characterized by dryness of the eyes and mouth due to inflammation and dysfunction of the salivary and lacrimal glands. In addition to the recurrent mouth ulcers, the woman also experiences pain and morning stiffness in her finger and wrist joints, which improve with activity throughout the day. The presence of proteinuria and hematuria (blood in urine) on urinalysis suggests possible kidney involvement.  The woman's laboratory findings, including a decreased hemoglobin level, lymphocytopenia (low lymphocyte count), and an elevated CRP level, are consistent with the inflammatory nature of Sjögren's syndrome.  While other conditions such as Behçet's disease, reactive arthritis, rheumatoid arthritis, and systemic lupus erythematosus may present with similar symptoms, the combination of dryness of the eyes and mouth, joint pain, recurrent mouth ulcers, and the laboratory findings make Sjögren's syndrome the most likely diagnosis in this case.  However, it's important to note that a definitive diagnosis can only be made by a healthcare professional based on a thorough evaluation, including further examination, additional laboratory tests, and possibly a biopsy. The woman should consult with a rheumatologist for further assessment and management. |
| **16** | A 66 year old man has lethargy, cough and a persistent pain in the right upper quadrant of the abdomen. He has smoked between 15 and 40 cigarettes daily for 50 years. He has an enlarged liver. Needle biopsy of the liver reveals adenocarcinoma, and immunohistochemistry is positive for the marker thyroid transcription factor-1, indicative of a primary pulmonary tumour.  Which is the most likely route by which this tumour spread to the liver?  A. Direct infiltration  B. Haematogenous spread  C. Lymphatic spread  D. Pagetoid spread  E. Transcoelomic spread | Attempt 1  Based on the given information, the most likely route by which the pulmonary tumor spread to the liver in this case is:  B. Haematogenous spread  The presence of a primary pulmonary tumor and the immunohistochemical marker thyroid transcription factor-1 suggest that the liver metastasis originated from the lung. Hematogenous spread refers to the spread of cancer cells through the bloodstream, allowing them to reach distant organs, including the liver. The liver is a common site for metastasis due to its rich blood supply and the filtering function of its sinusoids.  In this case, it is likely that the tumor cells from the primary lung tumor entered the bloodstream and eventually reached the liver, where they formed metastatic deposits. The patient's symptoms, such as lethargy, cough, and right upper quadrant pain, along with the presence of an enlarged liver, further support the diagnosis of liver metastasis.  While direct infiltration, lymphatic spread, pagetoid spread, and transcoelomic spread are possible routes of tumor spread in certain cancers, the given information points more strongly toward hematogenous spread as the most likely route in this case.  It's important to note that a comprehensive evaluation and further investigations, such as staging and imaging studies, would be necessary to confirm the extent of the disease and guide appropriate treatment options for the patient. |
|  |  | Attempt 2 |
|  |  | Attempt 3  Based on the provided information, the most likely route by which the lung tumor spread to the liver in this case is B. Haematogenous spread.  The presence of a primary pulmonary tumor and the finding of adenocarcinoma in the liver suggest metastatic disease. Adenocarcinomas are known to have a propensity for hematogenous spread, meaning they can spread through the bloodstream to distant organs such as the liver.  In this scenario, the lung tumor likely shed cancer cells into the bloodstream, which then traveled to the liver and formed metastases. The patient's smoking history and symptoms such as cough further support the presence of a primary lung tumor.  Direct infiltration (option A) would involve the tumor directly invading adjacent tissues, such as spreading from the lung to the nearby pleura or chest wall. Lymphatic spread (option C) would involve the tumor spreading to regional lymph nodes before reaching the liver. Pagetoid spread (option D) refers to the spread of cancer along the basement membrane of an epithelial surface, which is less likely in this case. Transcoelomic spread (option E) typically occurs in certain cancers, such as ovarian cancer, where cancer cells spread within the peritoneal cavity.  However, given the information provided, the most likely route of tumor spread from the lung to the liver is through the bloodstream (hematogenous spread). |
| **17** | A 63 year old woman has two days of facial swelling, early morning facial discomfort and fullness. Nine months ago she was treated with chemotherapy and radiotherapy for lung cancer. Her jugular venous pressure is elevated and non pulsatile. Her face, neck and arms are swollen, and there are prominent veins across her chest.  Which structure is most likely to be occluded?  A. Axillary vein  B. Internal jugular vein  C. Right atrium  D. Subclavian vein  E. Superior vena cava | Attempt 1  Based on the symptoms described, the most likely structure to be occluded in this case is:  E. Superior vena cava  The symptoms of facial swelling, early morning facial discomfort, fullness, elevated jugular venous pressure, prominent veins across the chest, and swelling of the face, neck, and arms suggest the presence of superior vena cava syndrome (SVCS). SVCS occurs when there is obstruction or compression of the superior vena cava, which is responsible for draining blood from the head, neck, and upper extremities back to the heart.  In this case, the previous history of lung cancer, chemotherapy, and radiotherapy suggests the possibility of tumor involvement or compression of the superior vena cava. Tumors in the mediastinal area, such as lung cancer, can cause compression or invasion of the superior vena cava, leading to the characteristic symptoms of SVCS.  It's important to note that further investigations and imaging studies, such as CT scans or venography, would be necessary to confirm the diagnosis and identify the underlying cause of the superior vena cava obstruction.  Prompt evaluation and management are important in cases of superior vena cava syndrome to relieve the obstruction and manage the underlying cause, which may involve interventions such as stenting, radiation therapy, or chemotherapy, depending on the underlying pathology. |
|  |  | Attempt 2  The most likely route by which this tumor spread to the liver in the 66-year-old man with an adenocarcinoma positive for thyroid transcription factor-1 (TTF-1) is:  B. Haematogenous spread  The presence of TTF-1 positivity indicates that the primary tumor likely originated in the lung. With a history of smoking and the clinical symptoms of cough and right upper quadrant abdominal pain, it suggests that the lung adenocarcinoma has metastasized to the liver.  Haematogenous spread refers to the dissemination of cancer cells through the bloodstream to distant organs. This is a common mode of spread for lung cancer, as cancer cells can invade the blood vessels and then travel to other parts of the body, including the liver.  Options A (Direct infiltration), C (Lymphatic spread), D (Pagetoid spread), and E (Transcoelomic spread) are less likely for lung cancer metastasis to the liver. While they are possible routes of tumor spread in some cancers, lung cancer is more commonly associated with hematogenous spread due to its rich blood supply and the presence of tumor cells in the bloodstream. |
|  |  | Attempt 3  Based on the provided information, the most likely structure to be occluded in this case is E. Superior vena cava (SVC).  The patient's symptoms, including facial swelling, early morning facial discomfort, fullness, elevated jugular venous pressure, and prominent veins across the chest, suggest obstruction of the SVC. The SVC is a large vein that carries deoxygenated blood from the upper body back to the heart. When it becomes partially or completely blocked or occluded, it can lead to impaired blood flow from the head, neck, and upper extremities.  The patient's history of lung cancer and previous treatment with chemotherapy and radiotherapy increase the suspicion of SVC obstruction. Tumors in the mediastinum (the area between the lungs) can compress or invade the SVC, leading to its occlusion. In this case, the lung cancer and the previous treatment may have contributed to the development of SVC syndrome.  Options A, B, C, and D (axillary vein, internal jugular vein, right atrium, subclavian vein) are less likely to be occluded based on the symptoms and context provided. The clinical presentation described is more consistent with superior vena cava syndrome due to obstruction at or near the level of the superior vena cava. |
| **18** | A 17 year old attends her GP with her mother. She has had severe headaches, stomach aches and has vomited four times over 2 days. She was invited to a close friend's birthday party but refused to attend. She is extremely shy and isolated, and has very few friends. She is always worried that others criticise her, is extremely sensitive to any rejection, and is very attached to her mother.  Which personality disorder is she most likely to have?  A. Avoidant  B. Dependent  C. Paranoid  D. Schizoid  E. Schizotypal | Attempt 1  Based on the given information, the most likely personality disorder that the 17-year-old girl may have is:  A. Avoidant personality disorder  The symptoms of severe headaches, stomach aches, vomiting, extreme shyness, isolation, few friends, fear of criticism, sensitivity to rejection, and strong attachment to her mother are suggestive of avoidant personality disorder. Individuals with avoidant personality disorder have an intense fear of rejection, criticism, and humiliation, leading them to avoid social situations and isolate themselves from others. They often have low self-esteem and are extremely sensitive to perceived negative evaluations by others.  In this case, the girl's refusal to attend her close friend's birthday party, despite being invited, along with her overall pattern of social avoidance and anxiety, align with the criteria for avoidant personality disorder.  It's important to note that a comprehensive assessment by a mental health professional would be necessary to make a formal diagnosis and determine the most appropriate treatment approach, which may include psychotherapy or counseling to address the underlying fears and social difficulties associated with avoidant personality disorder. |
|  |  | Attempt 2  Based on the provided information, the 17-year-old girl is most likely to have:  A. Avoidant personality disorder  The symptoms of severe headaches, stomach aches, vomiting, social withdrawal, extreme shyness, isolation, fear of criticism, sensitivity to rejection, and strong attachment to her mother are characteristic features of avoidant personality disorder.  People with avoidant personality disorder tend to be excessively shy, socially inhibited, and avoid situations where they might be evaluated or criticized. They have an intense fear of rejection and often feel inadequate and inferior to others. This fear of rejection and criticism leads them to avoid social interactions and relationships, which can significantly impact their daily life and emotional well-being.  Options B (Dependent), C (Paranoid), D (Schizoid), and E (Schizotypal) do not fit the described symptoms as well as avoidant personality disorder does. |
|  |  | Attempt 3  Based on the provided information, the most likely personality disorder that the 17-year-old girl may have is A. Avoidant Personality Disorder.  Avoidant Personality Disorder is characterized by a pervasive pattern of social inhibition, feelings of inadequacy, and hypersensitivity to negative evaluation. Individuals with this disorder typically have a strong fear of rejection, criticism, and disapproval, which leads to avoidance of social situations and limited interpersonal relationships. They often feel socially inept, shy, and isolated.  The girl's severe headaches, stomach aches, vomiting, and refusal to attend social events like her friend's birthday party can be attributed to her avoidance behavior and heightened anxiety in social situations. Her extreme sensitivity to criticism and strong attachment to her mother are also consistent with Avoidant Personality Disorder.  Options B, C, D, and E (Dependent, Paranoid, Schizoid, Schizotypal) do not align as closely with the described symptoms and characteristics of the individual in question. |
| **19** | A 45 year old man visits his GP with right knee pain. He has not had any problems with his knee before, but has had previous episodes of a severe pain in his toe and painful swelling of the dorsum of the feet. There is no history of trauma. He drinks 20 pints of beer a week. His temperature is 37.5°C, and his knee is warm, diffusely tender and very painful to flex.  What is the most likely diagnosis?  A. Gout  B. Osteoarthritis  C. Rheumatoid arthritis  D. Septic arthritis  E. Torn meniscal cartilage | Attempt 1  Based on the provided information, the most likely diagnosis for the 45-year-old man's right knee pain is:  A. Gout  The history of severe pain in the toe and painful swelling of the dorsum of the feet, along with the current symptoms of warm, diffusely tender, and very painful knee, suggests gout as the most likely diagnosis. Gout is a form of inflammatory arthritis caused by the deposition of uric acid crystals in the joints. It commonly affects the big toe (podagra), but other joints, such as the knee, can also be involved.  The risk factors for gout in this case include the patient's history of previous episodes of toe pain and foot swelling, as well as his alcohol consumption (drinking 20 pints of beer per week). Alcohol, particularly beer, is known to increase the risk of developing gout.  Further evaluation, such as joint fluid analysis to confirm the presence of uric acid crystals, may be required for a definitive diagnosis of gout. Treatment typically involves medications to manage pain and inflammation, as well as lifestyle modifications to reduce alcohol consumption and avoid purine-rich foods.  It's important for the patient to follow up with his GP for a proper evaluation and management of his symptoms. |
|  |  | Attempt 2  The most likely diagnosis for the 45-year-old man with right knee pain, previous episodes of severe pain in his toe, and painful swelling of the dorsum of the feet, along with a history of heavy alcohol consumption is:  A. Gout  Gout is a form of inflammatory arthritis caused by the deposition of uric acid crystals in the joints and other tissues. It commonly affects the big toe (podagra) but can also involve other joints, such as the knee.  The presence of severe pain in the toe (podagra) and painful swelling of the dorsum of the feet, along with the history of alcohol consumption and the clinical presentation of a warm, diffusely tender, and very painful knee, are highly suggestive of gout.  Option B (Osteoarthritis) is less likely in this case as the symptoms described are more consistent with an inflammatory arthritis like gout, rather than the degenerative changes seen in osteoarthritis.  Option C (Rheumatoid arthritis) can also cause inflammatory joint symptoms, but gout is more strongly associated with episodes of acute pain and swelling that may involve multiple joints.  Option D (Septic arthritis) is a possibility, but the history of previous episodes of severe pain in the toe and the swelling of the feet are more characteristic of gout. However, septic arthritis should always be considered in cases of acute joint pain and swelling.  Option E (Torn meniscal cartilage) is less likely as it typically presents with localized knee pain and swelling after a history of trauma, which is not described in this case.  Given the clinical presentation, gout is the most likely diagnosis, and the patient should undergo further evaluation and management, including blood tests to assess uric acid levels and joint fluid analysis to confirm the diagnosis. Treatment typically involves medications to relieve pain, reduce inflammation, and lower uric acid levels to prevent future episodes. |
|  |  | Attempt 3  The most likely diagnosis in this case is A. Gout.  Gout is a form of inflammatory arthritis that typically affects the joints, most commonly the metatarsophalangeal joint of the big toe. It is caused by the deposition of uric acid crystals in the joints, leading to sudden and severe attacks of pain, swelling, redness, and warmth. The symptoms are often triggered by elevated levels of uric acid in the blood.  The patient's history of previous episodes of severe pain in the toe and painful swelling of the dorsum of the feet, along with his current presentation of warm, diffusely tender, and very painful knee, are highly suggestive of gout. The association with alcohol consumption (drinking 20 pints of beer a week) further supports the diagnosis, as alcohol can increase uric acid levels and trigger gout attacks.  Osteoarthritis (B) typically presents with joint pain and stiffness that worsens over time, but it is not typically associated with acute inflammation. Rheumatoid arthritis (C) is characterized by symmetric joint involvement and systemic symptoms, and it is less likely in this case. Septic arthritis (D) is a bacterial infection of the joint and would usually present with more severe symptoms, such as high fever and rapidly worsening joint pain. Torn meniscal cartilage (E) would typically present with localized knee pain and a history of trauma, which is not described in the scenario. |
| **20** | A 68 year old woman has noticed a lump in her neck for 2 months, but otherwise feels well. There are small lymph nodes palpable in the cervical, axillary and inguinal regions. Investigations: Haemoglobin 124 g/L (115–150) White cell count 27.2 × 109/L (3.8–10.0) Neutrophils 2.5 × 109/L (2.0–7.5) Lymphocytes 21.6 × 109/L (1.1–3.3) Monocytes 0.9 × 109/L (0.2–1.0) Eosinophils 0.4 × 109/L (0–0.4) Basophils 0.1 × 109/L (0–0.1) Platelets 137 × 109/L (150–400) Blood film: increased lymphocytes with sparse cytoplasm  Which is the most likely diagnosis?  A. Acute lymphoblastic leukaemia  B. Chronic lymphocytic leukaemia  C. Infectious mononucleosis  D. Myeloma  E. Non Hodgkin's lymphoma | Attempt 1  The most likely diagnosis in this case is B. Chronic lymphocytic leukaemia (CLL).  CLL is a type of slow-growing leukemia characterized by the accumulation of mature-appearing lymphocytes in the blood, bone marrow, and lymphoid tissues. It primarily affects older adults and is more common in women. The typical presentation includes lymphadenopathy, hepatosplenomegaly, and often a mild, indolent course with few symptoms. In this case, the patient's history of a palpable lump in the neck and small lymph nodes in multiple regions, along with the increased lymphocyte count on the blood film, are consistent with CLL.  Acute lymphoblastic leukemia (A) is a rapidly progressing leukemia that primarily affects children. It is characterized by the proliferation of immature lymphoblasts. Infectious mononucleosis (C) typically presents with fever, pharyngitis, lymphadenopathy, and atypical lymphocytosis, but the blood film findings and prolonged duration of symptoms make it less likely in this case. Myeloma (D) is characterized by the proliferation of plasma cells and typically presents with symptoms related to bone marrow infiltration, such as bone pain and fractures. Non-Hodgkin's lymphoma (E) is a diverse group of lymphoid malignancies, and while it can present with lymphadenopathy, the blood film findings in this case are more consistent with CLL. |
|  |  | Attempt 2  Based on the provided information, the most likely diagnosis is B. Chronic lymphocytic leukemia (CLL).  Here are the key features that support this diagnosis:  1. Age: CLL is more common in older individuals, and the patient is 68 years old.  2. Duration of Lump: The patient has noticed a lump in her neck for 2 months.  3. Palpable lymph nodes in multiple regions: The presence of small lymph nodes in the cervical, axillary, and inguinal regions suggests lymphadenopathy, which is a common feature of CLL.  4. Blood Cell Counts: The blood test results show an increased lymphocyte count (21.6 × 10^9/L) with sparse cytoplasm, which is consistent with the typical lymphocytosis seen in CLL.  5. Other Blood Cell Counts: The patient also has a low hemoglobin level (124 g/L) and a slightly low platelet count (137 × 10^9/L), which can occur in CLL due to bone marrow involvement.  6. General Well-being: The patient mentions feeling well, which is common in early-stage CLL.  While other conditions like infectious mononucleosis and non-Hodgkin's lymphoma could cause lymphadenopathy and increased lymphocyte counts, CLL is more likely given the age, chronicity of the symptoms, and the characteristic blood findings.  It is important to note that a definitive diagnosis can only be made after further evaluation, such as bone marrow biopsy and flow cytometry, to confirm the presence of CLL and determine its stage and prognosis. A consultation with a hematologist or an oncologist is crucial for appropriate management and further investigations. |
|  |  | Attempt 3  Based on the provided information, the most likely diagnosis for the 68-year-old woman with a lump in her neck and lymphadenopathy is:  E. Non Hodgkin's lymphoma  The presence of enlarged lymph nodes in multiple regions (cervical, axillary, inguinal), along with increased lymphocyte count and a blood film showing increased lymphocytes with sparse cytoplasm, suggests a lymphoproliferative disorder. Among the given options, non-Hodgkin's lymphoma (NHL) is the most likely diagnosis.  NHL is a type of cancer that originates in the lymphatic system. It is characterized by the abnormal growth of lymphocytes, which can lead to the formation of swollen lymph nodes. The specific subtype and stage of NHL would require further investigations, such as lymph node biopsy and imaging studies, to determine the exact diagnosis and appropriate treatment.  Acute lymphoblastic leukemia (ALL) is less likely in this case, as it typically presents with more rapid onset and significant bone marrow involvement, leading to anemia and thrombocytopenia. Chronic lymphocytic leukemia (CLL) is also a possibility, but the presence of palpable lymph nodes in multiple regions is more suggestive of NHL.  Infectious mononucleosis and myeloma may present with lymphadenopathy, but the blood film findings and the prolonged duration of symptoms make them less likely in this case.  It's important for the patient to undergo further evaluation by a hematologist/oncologist to confirm the diagnosis and initiate appropriate management. |
| **21** | A 15 year old boy has had severe anorexia nervosa for one year. He is not currently acutely physically unwell. His weight is 75% of the expected weight for his height, age and sex.  Which is the most appropriate treatment?  A. Cognitive-analytic therapy  B. Cognitive-behaviour therapy  C. Family therapy  D. Non-directive counselling  E. Psychodynamic psychotherapy | Attempt 1  The most appropriate treatment for a 15-year-old boy with severe anorexia nervosa is:  C. Family therapy  Family therapy has been shown to be the most effective treatment approach for adolescents with anorexia nervosa. It recognizes the role of family dynamics and involvement in the development and maintenance of the eating disorder. Family-based treatment (FBT) or Maudsley Approach is a specific form of family therapy that is widely used and supported by evidence in the treatment of adolescent anorexia nervosa.  FBT involves active parental involvement in helping their child regain weight and normalize eating patterns. The therapy aims to empower parents as agents of change and support the adolescent's recovery. It typically consists of three phases: weight restoration, returning control over eating to the adolescent, and establishing healthy adolescent development.  Cognitive-behavioral therapy (CBT) can also be a component of the treatment plan, targeting distorted thoughts and behaviors related to the eating disorder. However, family therapy is the primary treatment approach for this age group.  Cognitive-analytic therapy, non-directive counseling, and psychodynamic psychotherapy may have their merits in other contexts, but they are not the first-line treatments for severe anorexia nervosa in adolescents.  It's important to involve a multidisciplinary team, including physicians, therapists, and dieticians, to provide comprehensive care and address the medical, psychological, and nutritional aspects of the disorder. |
|  |  | Attempt 2  The most appropriate treatment for a 15-year-old boy with severe anorexia nervosa is:  C. Family therapy  Family therapy has been shown to be one of the most effective treatments for adolescents with anorexia nervosa. It involves the whole family in the treatment process, addressing both the individual's eating disorder and the family dynamics that may contribute to the development and maintenance of the disorder.  Anorexia nervosa is a complex psychiatric condition with both psychological and physical components. Involving the family in the treatment helps create a supportive and understanding environment for the affected individual. It also helps address any family-related issues that might be contributing to the illness.  While individual therapies like cognitive-analytic therapy, cognitive-behavioral therapy, and psychodynamic psychotherapy can be beneficial for some individuals, family therapy is particularly effective in the context of treating adolescents with anorexia nervosa due to the significant role of family dynamics in this age group. Non-directive counseling is not the most appropriate option for the treatment of severe anorexia nervosa, as this condition requires a more structured and specialized approach like family therapy. |
|  |  | Attempt 3  The most appropriate treatment for a 15-year-old boy with severe anorexia nervosa and significant weight loss would be C. Family therapy.  Family therapy has been shown to be effective in the treatment of adolescents with anorexia nervosa. It involves the entire family in the treatment process and aims to address the underlying family dynamics and interactions that may contribute to the development and maintenance of the eating disorder. Family therapy helps to improve communication, resolve conflicts, and promote healthier patterns of interaction within the family system.  Cognitive-behavioral therapy (B) is also commonly used in the treatment of anorexia nervosa and can be effective in helping individuals change their thoughts, attitudes, and behaviors related to food, body image, and weight. However, given the age of the patient and the involvement of the family, family therapy is often the preferred approach.  Cognitive-analytic therapy (A) and psychodynamic psychotherapy (E) may have a role in the treatment of anorexia nervosa in some cases, but they are not typically the first-line treatments for adolescents with severe anorexia nervosa.  Non-directive counseling (D) alone is unlikely to be sufficient for the treatment of severe anorexia nervosa, as it does not provide the structured interventions and support needed to address the complex factors contributing to the disorder.  It's important to note that the treatment approach may vary depending on the individual's specific needs, the severity of the condition, and the available resources. A comprehensive assessment by a qualified healthcare professional is essential to determine the most appropriate treatment plan for the individual. |
| **22** | A randomised controlled trial was designed to compare a new drug with a placebo. There were 120 patients in the treatment group of which 12 patients died within 2 years. There were 240 patients in the control group of which 48 patients died within 2 years.  Which is the number needed to treat to avoid one death within 2 years?  A. 2  B. 4  C. 8  D. 10  E. 20 | Attempt 1  To calculate the number needed to treat (NNT) to avoid one death within 2 years, we need to compare the absolute risk reduction between the treatment group and the control group.  In the treatment group, the absolute risk reduction is calculated as the difference in the mortality rate between the treatment and control groups:  Absolute Risk Reduction = Control Group Mortality Rate - Treatment Group Mortality Rate  Absolute Risk Reduction = 48/240 - 12/120  Absolute Risk Reduction = 0.2 - 0.1  Absolute Risk Reduction = 0.1  To calculate the NNT, we take the reciprocal of the absolute risk reduction:  NNT = 1 / Absolute Risk Reduction  NNT = 1 / 0.1  NNT = 10  Therefore, the number needed to treat to avoid one death within 2 years is:  D. 10 |
|  |  | Attempt 2  To calculate the Number Needed to Treat (NNT) to avoid one death within 2 years, we need to find the absolute risk reduction (ARR) between the treatment group and the control group.  Absolute Risk Reduction (ARR) = Control Event Rate (CER) - Experimental Event Rate (EER)  CER = Number of deaths in the control group / Total number of patients in the control group  EER = Number of deaths in the treatment group / Total number of patients in the treatment group  CER = 48 deaths / 240 patients = 0.2 (20%)  EER = 12 deaths / 120 patients = 0.1 (10%)  ARR = 0.2 - 0.1 = 0.1 (10%)  Now, NNT can be calculated as the inverse of the ARR:  NNT = 1 / ARR  NNT = 1 / 0.1  NNT = 10  Therefore, the Number Needed to Treat (NNT) to avoid one death within 2 years is:  D. 10 |
|  |  | Attempt 3  To calculate the number needed to treat (NNT) to avoid one death within 2 years, we need to find the reciprocal of the absolute risk reduction (ARR).  In the treatment group, the absolute risk reduction (ARR) is calculated by subtracting the event rate in the treatment group from the event rate in the control group:  ARR = Event rate in control group - Event rate in treatment group  Event rate in control group = 48/240 = 0.20  Event rate in treatment group = 12/120 = 0.10  ARR = 0.20 - 0.10 = 0.10  Now, to calculate the NNT, we take the reciprocal of the ARR:  NNT = 1 / ARR  NNT = 1 / 0.10 = 10  Therefore, the number needed to treat to avoid one death within 2 years is 10 (D). |
| **23** | A 28 year old woman is elated in mood, has not slept for 7 days and has been attending all-night parties. She is sexually disinhibited and believes that she is a member of the royal family. She was previously fit and well and has no past psychiatric history. A urine drug screen is negative.  Which is the most appropriate medication?  A. Diazepam  B. Lithium carbonate  C. Olanzapine  D. Sodium valproate  E. Zuclopenthixol decanoate | Attempt 1  The most appropriate medication for the described scenario is:  C. Olanzapine  The symptoms described suggest a manic episode, which is characteristic of bipolar disorder. Olanzapine is an atypical antipsychotic medication commonly used in the treatment of acute manic episodes associated with bipolar disorder. It helps to reduce the severity of manic symptoms, stabilize mood, and improve overall functioning. Other medications commonly used for bipolar disorder include mood stabilizers such as lithium carbonate and sodium valproate, but given the acute presentation and severity of symptoms, olanzapine is a suitable initial choice. |
|  |  | Attempt 2  The most appropriate medication for the described presentation is:  C. Olanzapine  The woman is exhibiting symptoms consistent with a manic episode, which is a characteristic feature of bipolar disorder. Manic episodes are characterized by elevated mood, decreased need for sleep, increased energy, grandiosity, impulsivity, and sometimes psychosis (e.g., believing she is a member of the royal family).  Olanzapine is an antipsychotic medication with mood-stabilizing properties commonly used in the treatment of acute manic episodes associated with bipolar disorder. It helps control symptoms such as agitation, psychosis, and excessive elation.  The other options are not the first-line treatment for a manic episode:  A. Diazepam: It is a benzodiazepine used for anxiety and sedation, but it does not treat the underlying manic symptoms.  B. Lithium carbonate: It is a mood stabilizer often used for bipolar disorder, including the prevention of manic and depressive episodes, but it may take some time to reach therapeutic levels and is not the first-line treatment for acute mania.  D. Sodium valproate: Like lithium, it is a mood stabilizer, but it may also take time to reach therapeutic levels for acute mania.  E. Zuclopenthixol decanoate: This is a long-acting antipsychotic medication and is not the first-line treatment for acute mania.  In the described case, olanzapine would be a more suitable and effective option to manage the acute manic symptoms. However, the patient should be thoroughly assessed by a psychiatrist to establish a definitive diagnosis and develop a comprehensive treatment plan, which may involve additional therapies and medications as needed. |
|  |  | Attempt 3  The most appropriate medication for the described presentation is option C: Olanzapine.  The patient's symptoms and behavior suggest a manic episode, which is a characteristic feature of bipolar disorder. Olanzapine is an atypical antipsychotic medication commonly used in the treatment of acute manic episodes associated with bipolar disorder. It helps to stabilize mood and reduce symptoms of mania, such as elation, insomnia, impulsivity, and grandiose beliefs.  Diazepam (option A) is a benzodiazepine and is not the first-line treatment for manic episodes. It may be used for acute agitation or to manage insomnia, but it does not address the underlying mood symptoms.  Lithium carbonate (option B) is a mood stabilizer commonly used for long-term management of bipolar disorder to prevent relapse and stabilize mood. While it is effective for maintenance treatment, it is not typically used as the initial treatment for an acute manic episode.  Sodium valproate (option D) is another mood stabilizer used in the treatment of bipolar disorder. However, olanzapine is generally preferred for acute manic episodes due to its faster onset of action.  Zuclopenthixol decanoate (option E) is a long-acting antipsychotic medication. While it may be used in certain cases, olanzapine is often preferred as a first-line option for acute manic episodes.  It's important to note that the choice of medication should be made by a qualified healthcare professional based on a comprehensive assessment of the individual's condition. |
| **24** | A 62 year old woman is seen at home because her legs feel "wobbly", and she has been incontinent of urine once. She has been experiencing increasing pain in her mid back over the previous fortnight. She is being treated for metastatic breast cancer with anastrozole. There is slight weakness, rated 4/5, in hip flexion and knee extension bilaterally. Plantar reflexes are equivocal and tendon jerks brisk. She is tender over the 10ththoracic vertebra.  Which is the most appropriate next course of action?  A. Admission to the local hospice  B. Advise bed rest until pain resolves  C. Arrange domiciliary physiotherapy  D. Emergency admission for MR scan of spine  E. Give analgesia and re-examine her in the morning | Attempt 1  The most appropriate next course of action in this case is:  D. Emergency admission for MR scan of the spine  The patient's symptoms of increasing pain in the mid-back, weakness in hip flexion and knee extension, urinary incontinence, and tenderness over the thoracic vertebra raise concerns for spinal cord compression, especially given her history of metastatic breast cancer. Urgent imaging with an MRI scan of the spine is needed to evaluate the extent and location of the compression. Prompt diagnosis and intervention are crucial in preventing further neurological deficits and managing the underlying cause. Therefore, an emergency admission for an MRI scan of the spine is the most appropriate next step. |
|  |  | Attempt 2  Given the patient's presentation and clinical findings, the most appropriate next course of action is:  D. Emergency admission for MR scan of the spine  The patient is a 62-year-old woman with metastatic breast cancer and recent onset of "wobbly" legs, urinary incontinence, increasing mid-back pain, and neurological signs such as bilateral weakness (hip flexion and knee extension) and equivocal plantar reflexes. These symptoms and signs may indicate spinal cord compression, a serious complication that can occur due to the spread of cancer to the spine.  Spinal cord compression is a medical emergency that requires urgent evaluation and intervention to prevent permanent neurological damage. The most appropriate next step is an emergency admission to the hospital for an MRI (Magnetic Resonance Imaging) of the spine. MRI is the imaging modality of choice for evaluating the spinal cord and surrounding structures to identify any compression or other abnormalities.  Admission to the local hospice (option A) is not appropriate at this stage, as the patient's symptoms require immediate evaluation and management in a hospital setting.  Bed rest (option B) is not the appropriate course of action for a patient with suspected spinal cord compression. Delaying appropriate evaluation and treatment could lead to irreversible neurological deficits.  Domiciliary physiotherapy (option C) is not appropriate in this case because the patient's symptoms suggest a medical emergency that needs urgent investigation and intervention.  Giving analgesia and re-examining her in the morning (option E) is also not appropriate as it does not address the potential underlying spinal cord compression, and any delay in diagnosis and treatment could lead to further neurological deterioration.  Prompt evaluation and intervention are crucial in this scenario to prevent further complications and preserve the patient's neurological function. |
|  |  | Attempt 3  Given the symptoms and findings described, the most appropriate next course of action would be option D: Emergency admission for an MR scan of the spine.  The patient's symptoms of progressively worsening mid back pain, weakness in hip flexion and knee extension, incontinence of urine, and tenderness over the thoracic vertebra suggest possible spinal cord compression. This is a medical emergency that requires immediate evaluation and intervention.  Metastatic breast cancer can spread to the spine, leading to compression of the spinal cord or nerve roots. Prompt diagnosis and treatment are crucial to prevent further neurological deficits and manage the underlying cause. An MRI scan of the spine is the imaging modality of choice to assess the extent and location of spinal cord compression.  Options A, B, and C (admission to the local hospice, advising bed rest, arranging domiciliary physiotherapy) are not appropriate in this acute situation, as they do not address the urgent need for evaluation and potential intervention.  Option E (giving analgesia and re-examining in the morning) may provide temporary pain relief but does not address the underlying cause or evaluate the spinal cord compression. Delaying further assessment and treatment could lead to irreversible neurological damage.  It's important for the patient to be promptly referred to an appropriate healthcare facility for further evaluation and management. |
| **25** | A 3 year old boy has 6 months of diarrhoea. He has 4-5 watery stools per day that contain visible pieces of sweetcorn and other vegetables. He is thriving and developing well.  Which is the most likely diagnosis?  A. Coeliac disease  B. Gastroenteritis  C. Inflammatory bowel disease  D. Lactose intolerance  E. Toddler diarrhoea | Attempt 1  The most likely diagnosis in this case is:  E. Toddler diarrhoea  Toddler diarrhoea is a common condition in children between the ages of 6 months and 5 years. It is characterized by chronic, non-bloody, watery diarrhea that typically occurs during the daytime and may contain undigested food particles, such as visible pieces of sweetcorn and other vegetables. Despite the chronic diarrhea, children with toddler diarrhoea generally thrive and have normal growth and development.  It is important to note that other causes of chronic diarrhea, such as coeliac disease, inflammatory bowel disease, and lactose intolerance, should be considered and ruled out through appropriate evaluation if the symptoms are atypical or if the child's growth and development are affected. However, in the given scenario where the child is thriving and developing well, and the diarrhea is consistent with the features of toddler diarrhoea, it is the most likely diagnosis. |
|  |  | Attempt 2  The most likely diagnosis for the 3-year-old boy with 6 months of diarrhea, 4-5 watery stools per day containing visible pieces of sweetcorn and other vegetables, and thriving development is:  E. Toddler diarrhoea  Toddler's diarrhea, also known as functional diarrhea or chronic nonspecific diarrhea of infancy, is a common cause of chronic diarrhea in young children between the ages of 6 months to 5 years. It is characterized by chronic, non-bloody, watery stools that contain undigested food particles, such as visible pieces of sweetcorn and vegetables, as described in the case.  The term "toddler diarrhea" is used to describe the condition because it is commonly seen in toddlers, but it can also affect younger infants. The condition is generally benign and self-limiting, and affected children are usually otherwise healthy and growing well.  The key features that support the diagnosis of toddler diarrhea in this case are the age of the child (3 years), the duration of diarrhea (6 months), the frequency and appearance of stools, and the fact that the child is thriving and developing well.  The other options are less likely given the clinical presentation:  A. Coeliac disease: Coeliac disease may present with chronic diarrhea and malabsorption, but it is less likely to have visible pieces of undigested food in the stool. It is also associated with failure to thrive in some cases.  B. Gastroenteritis: Gastroenteritis is typically acute in nature, and the duration of symptoms for 6 months would be atypical for this condition.  C. Inflammatory bowel disease: Inflammatory bowel disease (such as Crohn's disease or ulcerative colitis) can cause chronic diarrhea, but it is uncommon in young children, and additional symptoms like weight loss, fever, and blood in stools are more commonly seen.  D. Lactose intolerance: Lactose intolerance can cause diarrhea in response to consuming lactose-containing foods, but it is less likely to persist for 6 months without significant improvement or changes in diet.  Given the child's age, chronicity of symptoms, and the absence of alarming features, the most likely diagnosis is toddler diarrhea. However, any persistent diarrhea in a child should be evaluated by a healthcare professional to confirm the diagnosis and rule out other potential causes. |
|  |  | Attempt 3  The most likely diagnosis in this case is option E: Toddler diarrhoea.  Toddler diarrhoea is a common condition in children between the ages of 6 months and 5 years. It is characterized by chronic, non-bloody, watery diarrhea without any signs of malabsorption or failure to thrive. The child usually remains well-nourished and continues to develop normally.  In toddler diarrhea, the stools may contain undigested food particles, such as visible pieces of vegetables or sweetcorn, which is not concerning as long as the child is otherwise thriving. The exact cause of toddler diarrhea is not fully understood, but it is thought to be related to dietary factors, such as excessive fruit juice consumption or carbohydrate malabsorption.  Coeliac disease (option A) typically presents with chronic diarrhea, but it is associated with malabsorption, failure to thrive, and other signs of nutrient deficiencies. Gastroenteritis (option B) usually presents with acute diarrhea, vomiting, and may be associated with fever or other systemic symptoms. Inflammatory bowel disease (option C) is less common in young children and is associated with more severe symptoms, including bloody diarrhea and systemic manifestations. Lactose intolerance (option D) can cause diarrhea, but it typically occurs after consuming milk or dairy products and is associated with symptoms of lactose malabsorption, such as bloating and flatulence.  Given the child's age, chronic watery diarrhea without signs of malabsorption or failure to thrive, and the presence of undigested food particles in the stool, toddler diarrhea is the most likely diagnosis. It is a benign condition that typically resolves spontaneously over time. However, if there are any concerning features or if the symptoms persist or worsen, it is always important to consult a healthcare professional for further evaluation and management. |
| **26** | A mother gives her 6 month old baby girl formula milk for the first time. Within minutes, the baby vomits and develops an erythematous rash over her lips and chin, which takes 1 hour to subside. The following week, the mother drips a couple of drops of formula milk onto the baby's arm. The baby develops an erythematous rash over the arm within 1 minute. The mother is returning to work and does not wish to continue breastfeeding or expressing milk.  Which is the best management option?  A. Goat's milk  B. Hydrolysed formula  C. Lactose free formula  D. Latex free teat  E. Soya milk | Attempt 1  Based on the given information, the baby's symptoms suggest an allergic reaction to the formula milk. The best management option in this case would be:  B. Hydrolysed formula  Hydrolysed formula refers to formulas in which the proteins are partially or extensively broken down into smaller fragments. This can help reduce the risk of an allergic reaction in infants who are sensitive or allergic to cow's milk proteins.  Goat's milk, lactose-free formula, soy milk, and latex-free teats are not the best options in this case. Goat's milk and soy milk are still derived from animal sources and can still trigger an allergic reaction in a baby with a cow's milk protein allergy. Lactose-free formula is intended for infants with lactose intolerance, not a cow's milk protein allergy. Latex-free teats are used for babies with latex allergies but do not address the issue of the formula allergy.  Therefore, the most appropriate choice is hydrolysed formula, as it is specifically designed for infants with cow's milk protein allergies or sensitivities. It is important to consult with a healthcare professional for further guidance and evaluation in order to confirm the diagnosis and ensure the baby's nutritional needs are met. |
|  |  | Attempt 2  The baby's symptoms, including immediate vomiting and an erythematous rash after exposure to formula milk, suggest an allergic reaction. Allergic reactions to cow's milk protein are not uncommon in infants.  The best management option in this case would be:  B. Hydrolysed formula  Hydrolysed formula is designed to have proteins that are broken down into smaller fragments, making them less likely to trigger allergic reactions. It is often recommended for infants with cow's milk protein allergy or suspected allergy.  The other options are not appropriate in this scenario:  A. Goat's milk: Goat's milk protein is similar to cow's milk protein and may also cause an allergic reaction in infants with cow's milk protein allergy.  C. Lactose-free formula: Lactose intolerance is rare in infants, and the baby's symptoms are more consistent with a cow's milk protein allergy rather than lactose intolerance.  D. Latex-free teat: Latex allergy is unrelated to the symptoms described in the case.  E. Soya milk: Soy protein is also a common allergen and is not recommended as a first-line alternative in infants with cow's milk protein allergy.  It is essential for the mother to work closely with a pediatrician or a healthcare professional to ensure the baby's nutritional needs are met while avoiding cow's milk protein. The pediatrician can guide the mother in choosing an appropriate hypoallergenic formula and monitor the baby's response to the new formula to ensure its suitability. |
|  |  | Attempt 3  The best management option in this case is option B: Hydrolysed formula.  The clinical presentation described suggests that the baby is experiencing an allergic reaction to the formula milk. The immediate vomiting and the development of an erythematous rash on the lips and chin are consistent with an IgE-mediated allergic reaction. The subsequent development of an erythematous rash on the arm within a minute after exposure to the formula milk further supports this diagnosis.  In cases of suspected formula milk allergy, the recommended management is to switch to a hypoallergenic formula. Hydrolysed formulas, such as extensively hydrolysed or amino acid-based formulas, are specifically designed for infants with cow's milk protein allergy. These formulas have the proteins broken down into smaller fragments, reducing the likelihood of an allergic reaction. Hydrolysed formulas are considered the gold standard for infants with confirmed or suspected cow's milk protein allergy.  Option A (Goat's milk) is not recommended as a substitute because it contains similar proteins to cow's milk and may still trigger an allergic reaction. Option C (Lactose-free formula) is not necessary unless there is a confirmed lactose intolerance, which is rare in infants. Option D (Latex-free teat) is not relevant to the management of formula milk allergy. Option E (Soya milk) is also not recommended as a first-line choice due to a relatively high incidence of cross-reactivity with cow's milk protein.  It is important for the mother to discuss the baby's symptoms and the suspected formula milk allergy with a healthcare professional, who can provide appropriate guidance and support for the transition to a hypoallergenic formula. |
| **27** | A 28 year old woman is admitted with a sudden onset of abdominal pain and vaginal bleeding. She is 38 weeks pregnant. Her pulse rate is 120 bpm and BP 110/80 mmHg. She has a tender abdomen. The fetal heart rate is 170 bpm.  Which is the most likely diagnosis?  A. Necrosis of a fibroid  B. Placenta praevia  C. Placental abruption  D. Pre-term labour  E. Uterine rupture | Attempt 1  Based on the given information, the most likely diagnosis in this case is:  C. Placental abruption  Placental abruption refers to the separation of the placenta from the uterine wall before the delivery of the baby. It is characterized by sudden onset abdominal pain and vaginal bleeding. The tender abdomen and the elevated fetal heart rate can also be indicative of placental abruption.  Necrosis of a fibroid, placenta previa, pre-term labor, and uterine rupture are other possible causes of abdominal pain and vaginal bleeding in pregnancy, but they are less likely based on the provided information.  It is important to note that this is a potential medical emergency, and immediate medical attention is necessary. The patient should be evaluated and managed by a healthcare professional in an appropriate healthcare setting. |
|  |  | Attempt 2  The most likely diagnosis in this case is:  C. Placental abruption  Placental abruption is a medical emergency that occurs when the placenta separates prematurely from the uterine wall before the baby is born. It can lead to significant maternal and fetal complications.  The symptoms and signs described in the case, including sudden onset of abdominal pain and vaginal bleeding in a 38-week pregnant woman with a tender abdomen, are typical of placental abruption. The elevated pulse rate (tachycardia) and fetal heart rate of 170 bpm may be signs of maternal and fetal distress due to the placental separation.  Let's briefly review the other options:  A. Necrosis of a fibroid: While fibroids can cause abdominal pain during pregnancy, it is less likely to present with sudden onset bleeding and maternal tachycardia.  B. Placenta praevia: Placenta praevia is a condition where the placenta is implanted in the lower part of the uterus, partially or completely covering the cervix. It typically presents with painless vaginal bleeding in the third trimester. The tender abdomen and maternal tachycardia in this case are more consistent with placental abruption.  D. Pre-term labour: Pre-term labor involves regular contractions and cervical changes before 37 weeks of gestation. The case description does not mention regular contractions or cervical changes.  E. Uterine rupture: Uterine rupture is a rare but severe complication during pregnancy and labor, which involves a complete tear of the uterine wall. It can lead to life-threatening bleeding and fetal distress. While it is more common in women with previous uterine surgeries (e.g., cesarean section), it is less likely to be the diagnosis in this case given the abrupt onset of symptoms and the absence of prior uterine surgeries.  Placental abruption is a medical emergency requiring prompt evaluation, monitoring, and appropriate management to ensure the well-being of both the mother and the baby. Immediate obstetric assessment and intervention are necessary in this situation. |
|  |  | Attempt 3  The most likely diagnosis in this case is C. Placental abruption.  Placental abruption is a condition in which the placenta separates from the uterine wall before delivery. It can present with sudden-onset abdominal pain and vaginal bleeding, which may range from mild to severe. The tender abdomen and increased maternal heart rate could be indicative of a significant abruption. The fetal heart rate of 170 bpm suggests fetal distress, which can occur due to compromised blood flow and oxygen supply to the fetus.  Necrosis of a fibroid (A) typically does not cause vaginal bleeding and fetal distress. Placenta previa (B) is characterized by painless vaginal bleeding, rather than abdominal pain. Pre-term labor (D) may cause abdominal pain and vaginal bleeding, but it is less likely in a term pregnancy. Uterine rupture (E) is a rare but serious complication that can cause severe abdominal pain and fetal distress, but it is more commonly associated with a scarred uterus from previous cesarean sections or other uterine surgeries.  Placental abruption is a medical emergency that requires prompt evaluation and management to ensure the well-being of both the mother and the fetus. Immediate obstetric consultation and appropriate interventions, such as fluid resuscitation, blood transfusion if necessary, and expedited delivery, may be required based on the severity of the abruption and the maternal and fetal conditions. |
| **28** | A 52 year old woman has had two days of worsening confusion. She has chronic liver disease secondary to alcohol and has been abstinent for six months. She has had a previous admission due to acute hepatic encephalopathy. She is disoriented in time and place, and is unable to hold a conversation. Her temperature is 37.1°C, pulse 90 bpm and BP 119/80 mmHg. She has a distended abdomen, widespread spider naevi and a flapping tremor. She is given intravenous vitamin supplements.  Which is the most appropriate additional management of her acute confusion?  A. Ciprofloxacin  B. Furosemide  C. Lactulose  D. Lorazepam  E. Restrict dietary protein intake to 60 g/day | Attempt 1  In this case, the most appropriate additional management for the patient's acute confusion would be:  C. Lactulose  The patient's history of chronic liver disease, previous admission for acute hepatic encephalopathy, and physical examination findings (such as spider naevi, flapping tremor, and distended abdomen) suggest that her confusion is likely due to hepatic encephalopathy. Lactulose is a standard treatment for hepatic encephalopathy.  Lactulose acts by reducing the production and absorption of ammonia in the gut, thereby decreasing the levels of ammonia in the blood. It helps to improve the symptoms of hepatic encephalopathy, including confusion and altered mental status.  Ciprofloxacin is an antibiotic and would not be the most appropriate choice for the management of hepatic encephalopathy.  Furosemide is a diuretic and would not directly address the underlying cause of the patient's confusion.  Lorazepam is a sedative, and while it may temporarily alleviate symptoms of anxiety or agitation, it does not target the underlying cause of hepatic encephalopathy.  Restricting dietary protein intake to 60 g/day may be a part of long-term management for patients with chronic liver disease, but it is not the immediate priority in the management of acute hepatic encephalopathy.  It is important for the patient to be evaluated and managed by a healthcare professional experienced in the care of liver disease, as additional interventions may be necessary based on the severity and underlying cause of her hepatic encephalopathy. |
|  |  | Attempt 2  The most appropriate additional management of her acute confusion in this case is:  C. Lactulose  The patient's presentation with chronic liver disease, two days of worsening confusion, and a history of previous hepatic encephalopathy suggests that she is likely experiencing another episode of hepatic encephalopathy. Hepatic encephalopathy is a neuropsychiatric syndrome associated with liver dysfunction, resulting in impaired cognitive function and altered mental status.  Lactulose is the first-line treatment for hepatic encephalopathy. It works by reducing the production and absorption of ammonia in the gut, which is believed to play a key role in the development of hepatic encephalopathy. Lactulose helps promote the excretion of ammonia in the stool, thus improving the patient's mental status.  The other options are not the most appropriate for managing hepatic encephalopathy:  A. Ciprofloxacin: This is an antibiotic and is not the primary treatment for hepatic encephalopathy.  B. Furosemide: Furosemide is a diuretic and is not indicated for the management of hepatic encephalopathy. In fact, it may exacerbate electrolyte imbalances in patients with liver disease.  D. Lorazepam: Lorazepam is a benzodiazepine and can worsen hepatic encephalopathy by depressing the central nervous system.  E. Restrict dietary protein intake to 60 g/day: Although dietary protein restriction may be helpful in managing hepatic encephalopathy in some cases, it is not the most appropriate initial treatment. Lactulose is the first-line therapy, and protein restriction is usually considered in conjunction with other measures if lactulose is insufficient to control symptoms.  In this case, administering lactulose to the patient is crucial for managing her acute confusion and improving her mental status. However, the patient's overall management should be under the care of a healthcare professional experienced in managing liver disease and hepatic encephalopathy, as it requires a comprehensive approach. |
|  |  | Attempt 3  The most appropriate additional management for her acute confusion, considering her history of chronic liver disease secondary to alcohol and previous admission for acute hepatic encephalopathy, is option C: Lactulose.  Lactulose is commonly used to treat hepatic encephalopathy, a condition that occurs in individuals with liver dysfunction. It helps reduce the levels of ammonia in the blood by promoting its elimination through the stool. High ammonia levels in the blood can lead to neurological symptoms like confusion, disorientation, and altered mental status.  Given her symptoms and history, administering lactulose would be the most suitable course of action to address her acute confusion. The other options are not specifically indicated for hepatic encephalopathy and may not be appropriate in this scenario. Here's a brief explanation of why the other options are not the most appropriate choices:  A. Ciprofloxacin: This is an antibiotic and would be used to treat bacterial infections. However, there is no indication of infection in the given scenario.  B. Furosemide: Furosemide is a diuretic used to manage fluid retention. It may not be the most appropriate treatment for hepatic encephalopathy.  D. Lorazepam: Lorazepam is a benzodiazepine and is generally avoided in patients with hepatic encephalopathy as it can worsen the condition and contribute to further confusion.  E. Restrict dietary protein intake to 60 g/day: While a low-protein diet may be part of long-term management for chronic liver disease, it may not be the most appropriate immediate action for treating acute hepatic encephalopathy and the associated acute confusion.  Always consult a healthcare professional for individualized medical advice and management. |
| **29** | A 25 year old nulliparous woman with an uncomplicated pregnancy attends for induction of labour at 41 weeks. Examination reveals the head is engaged; the cervix is posterior, 2cm long and the os is closed.  Which is the most appropriate method of induction?  A. Endovaginal prostaglandin gel  B. Membrane sweep  C. Oxytocin infusion  D. Oxytocin infusion and amniotomy  E. Progesterone suppository | Attempt 1  In this scenario, the most appropriate method of induction for the nulliparous woman at 41 weeks of pregnancy with a posterior cervix, 2cm long, and closed os would be:  C. Oxytocin infusion  Oxytocin is a synthetic hormone that can be used to induce labor. It is administered via an intravenous infusion and helps stimulate uterine contractions, leading to cervical dilation and effacement.  Endovaginal prostaglandin gel (option A) is typically used when the cervix is unfavorable (unripe) and needs ripening before initiating labor induction. Since the cervix in this case is posterior, 2cm long, and closed, it does not indicate the need for prostaglandin gel at this point.  A membrane sweep (option B) is a manual technique performed by a healthcare provider to separate the membranes of the amniotic sac from the lower part of the uterus. It is usually done when the cervix is partially dilated and effaced to help stimulate labor. However, in this case, the cervix is closed, and a membrane sweep may not be feasible or effective.  Oxytocin infusion and amniotomy (option D) could be considered if the cervix is partially dilated and effaced but not in this case where the cervix is still closed.  Progesterone suppository (option E) is not indicated for labor induction. Progesterone is typically used to prevent preterm labor in women at risk for premature birth.  It's important to note that the choice of induction method may vary based on individual patient factors and clinical judgment, so it's best to consult with a healthcare provider for personalized management. |
|  |  | Attempt 2  The most appropriate method of induction for a nulliparous woman with an unfavorable cervix (posterior, 2cm long, closed os) at 41 weeks of gestation is C. Oxytocin infusion.  In this scenario, the cervix is not yet favorable for induction, and cervical ripening is required to promote cervical softening and dilation. However, options A (Endovaginal prostaglandin gel) and B (Membrane sweep) are typically used when the cervix is more favorable, such as when the cervix is partially effaced and there is some cervical dilation.  Option D (Oxytocin infusion and amniotomy) is a possible approach if the cervix is favorable and ripened, but in this case, cervical ripening is needed before considering amniotomy and starting an oxytocin infusion.  Option E (Progesterone suppository) is not a method of induction and is typically used in the prevention of preterm labor in women with a history of preterm birth, not for induction of labor at term.  Therefore, in this scenario, the most appropriate method of induction would be to initiate a cervical ripening method such as C. Oxytocin infusion. This approach helps promote cervical changes and prepares the cervix for labor induction. |
|  |  | Attempt 3  The most appropriate method of induction for a nulliparous woman with an unfavorable cervix (posterior, 2cm long, closed os) at 41 weeks of gestation is C. Oxytocin infusion.  In this scenario, the cervix is not yet favorable for induction, and cervical ripening is required to promote cervical softening and dilation. However, options A (Endovaginal prostaglandin gel) and B (Membrane sweep) are typically used when the cervix is more favorable, such as when the cervix is partially effaced and there is some cervical dilation.  Option D (Oxytocin infusion and amniotomy) is a possible approach if the cervix is favorable and ripened, but in this case, cervical ripening is needed before considering amniotomy and starting an oxytocin infusion.  Option E (Progesterone suppository) is not a method of induction and is typically used in the prevention of preterm labor in women with a history of preterm birth, not for induction of labor at term.  Therefore, in this scenario, the most appropriate method of induction would be to initiate a cervical ripening method such as C. Oxytocin infusion. This approach helps promote cervical changes and prepares the cervix for labor induction. |
| **30** | A 60 year old man believes that the government has been monitoring him for 10 years, despite him repeatedly writing to the Prime Minister. His beliefs are present whatever his mood, and he has never heard voices when nobody else is around.  Which is the most likely diagnosis?  A. Anankastic personality disorder  B. Delusional disorder  C. Depression with psychosis  D. Paranoid schizophrenia  E. Schizoid personality disorder | Attempt 1  Based on the information provided, the most likely diagnosis for the 60-year-old man who believes that the government has been monitoring him for 10 years, regardless of his mood and without experiencing hallucinations, is:  B. Delusional disorder  Delusional disorder is characterized by persistent and non-bizarre delusions that are not accompanied by prominent hallucinations or other psychotic symptoms. The delusions experienced by individuals with delusional disorder are generally well-systematized and plausible, often centered around a specific theme, such as persecution, grandiosity, or jealousy. In this case, the man's belief that the government has been monitoring him for an extended period of time fits the criteria for a delusion.  Anankastic personality disorder (option A) is characterized by a preoccupation with orderliness, perfectionism, and control, rather than delusional beliefs.  Depression with psychosis (option C) typically involves the presence of depressive symptoms, such as low mood, feelings of worthlessness, and anhedonia, accompanied by psychotic features, including hallucinations or delusions that are congruent with the individual's depressive themes. However, the information provided does not suggest the presence of depressive symptoms in this case.  Paranoid schizophrenia (option D) involves a combination of both prominent delusions and hallucinations, with the delusions being a central feature of the disorder. The absence of hallucinations in the given scenario makes paranoid schizophrenia less likely.  Schizoid personality disorder (option E) is characterized by a pervasive pattern of detachment from social relationships and restricted emotional expression, rather than the presence of delusions.  It's important to note that a definitive diagnosis can only be made by a qualified healthcare professional after a thorough evaluation. The information provided here is for educational purposes and should not replace a proper medical assessment. |
|  |  | Attempt 2  The most likely diagnosis for the 60-year-old man who believes that the government has been monitoring him for 10 years, despite him repeatedly writing to the Prime Minister, and has never heard voices when nobody else is around is:  B. Delusional disorder  Explanation:  Delusional disorder is a psychiatric condition characterized by the presence of delusions (fixed, false beliefs) that persist for at least one month. These delusions are not due to other mental health conditions, such as schizophrenia or mood disorders with psychotic features. In this case, the man's belief that the government has been monitoring him for 10 years is a fixed, false belief (delusion) that has been present for an extended period of time.  The absence of hallucinations (hearing voices when nobody else is around) is also consistent with delusional disorder. Unlike schizophrenia, delusional disorder typically does not involve hallucinations or other prominent symptoms like disorganized thinking or negative symptoms.  The other options are less likely based on the information provided:  A. Anankastic personality disorder: This personality disorder is characterized by obsessive-compulsive traits and preoccupation with rules, orderliness, and control. It does not typically involve fixed, false beliefs (delusions).  C. Depression with psychosis: Psychotic depression is a mood disorder with psychotic features, such as hallucinations or delusions, occurring during a major depressive episode. However, the man's beliefs are not mood-congruent and are not primarily related to depression.  D. Paranoid schizophrenia: While paranoia is a common feature of schizophrenia, other symptoms like hallucinations, disorganized speech, or negative symptoms are usually present. In the given information, there are no indications of these additional symptoms.  E. Schizoid personality disorder: Schizoid personality disorder is a personality disorder characterized by a lack of interest in social relationships and emotional expression. It does not involve fixed, false beliefs (delusions).  Please note that a definitive diagnosis can only be made by a qualified mental health professional based on a comprehensive assessment of the individual's history, symptoms, and clinical presentation. If someone is experiencing mental health concerns, it is essential to seek professional help for an accurate diagnosis and appropriate treatment. |
|  |  | Attempt 3  Based on the provided information, the most likely diagnosis for the 60-year-old man is B. Delusional disorder.  Delusional disorder is characterized by the presence of one or more non-bizarre delusions that persist for at least one month. Non-bizarre delusions are beliefs that are plausible but highly unlikely or impossible. In this case, the man's belief that the government has been monitoring him for 10 years despite his attempts to communicate with the Prime Minister fits the criteria for a delusion.  The absence of mood symptoms or hallucinations suggests that the diagnosis is not related to mood disorders or psychotic depression (option C). The absence of hearing voices when alone suggests that the diagnosis is not paranoid schizophrenia (option D).  Options A and E refer to personality disorders, which are enduring patterns of behavior and inner experiences. While personality disorders can sometimes involve distorted thinking, the presence of persistent non-bizarre delusions that are not characteristic of the person's overall pattern of functioning is more indicative of a delusional disorder rather than a personality disorder.  Therefore, based on the given information, the most likely diagnosis for the 60-year-old man is B. Delusional disorder. |
| **31** | A woman develops a temperature of 38.5°C, 15 minutes after a transfusion of red blood cells.  What is the most likely cause of her high temperature?  A. Donor blood not screened for infective agents  B. Patient identified incorrectly  C. Pre-labelled sample used  D. Sample not labelled at the bedside  E. Sample not labelled by the person taking the blood | Attempt 1  Based on the given information, the most likely cause of the woman's high temperature 15 minutes after a transfusion of red blood cells is option A: Donor blood not screened for infective agents.  Transfusion reactions can occur due to various reasons, including transfusion of blood contaminated with infective agents. If the donor blood was not properly screened for infectious agents, such as bacteria or viruses, it could lead to a transfusion reaction and subsequent fever.  Options B, C, D, and E are related to errors in patient identification and sample labeling, which can lead to transfusion errors but are less likely to cause an immediate fever response within 15 minutes after the transfusion.  Therefore, given the timing of the temperature rise shortly after the transfusion, the most likely cause in this scenario is that the donor blood was not properly screened for infective agents (option A). |
|  |  | Attempt 2  The most likely cause of the woman's high temperature of 38.5°C, 15 minutes after a transfusion of red blood cells is:  A. Donor blood not screened for infective agents  Explanation:  Fever (high temperature) occurring shortly after a blood transfusion can be a sign of a transfusion reaction. Transfusion reactions can occur due to various reasons, but in this case, the most likely cause is that the donor blood was not adequately screened for infective agents.  Transfusion of blood products is a critical medical procedure, and it is essential to ensure that the donor blood is safe and free from infections or other potential complications. Proper screening and testing of donor blood are done to reduce the risk of transfusion-transmissible infections, such as bacterial, viral, or other pathogen-related infections.  The other options (B. Patient identified incorrectly, C. Pre-labelled sample used, D. Sample not labelled at the bedside, E. Sample not labelled by the person taking the blood) are less likely to cause an immediate fever shortly after the transfusion. They may lead to other types of medical errors, but they would not directly cause a fever within 15 minutes after the transfusion.  It is crucial to recognize and promptly manage transfusion reactions. If a patient experiences a fever or any other adverse reaction after a blood transfusion, healthcare professionals should be notified immediately, and appropriate actions should be taken to provide the necessary medical care and support to the patient. |
|  |  | Attempt 3  The most likely cause of the woman's high temperature (38.5°C) occurring 15 minutes after a transfusion of red blood cells is:  C. Pre-labelled sample used  When a pre-labelled sample is used for transfusion, there is a risk of ABO incompatibility, which can lead to a transfusion reaction. ABO incompatibility occurs when the patient receives blood that is of a different ABO blood group than their own. It can result in a fever, along with other symptoms such as chills, back pain, shortness of breath, and hemoglobinuria.  The other options listed are related to errors in identification and labelling of blood samples, but they would not directly cause a fever shortly after transfusion. However, it's important to note that in a clinical setting, the actual cause of the fever would need to be investigated and confirmed by medical professionals through appropriate diagnostic procedures. |
| **32** | N/A | Attempt 1 |
|  |  | Attempt 2 |
|  |  | Attempt 3 |
| **33** | A 45 year old man has a 5 month history of chronic sinusitis and unresolving headaches. When he bends forward, purulent fluid pours from his nose.  Which anatomical structure is most likely to be the source of this fluid?  A. Anterior ethmoid air cells  B. Frontal sinus  C. Lacrimal sac  D. Maxillary sinus  E. Posterior ethmoid air cells | Attempt 1  The most likely anatomical structure to be the source of the purulent fluid pouring from the nose when the patient bends forward is:  D. Maxillary sinus  The maxillary sinus is located in the cheekbones and is one of the paranasal sinuses. Chronic sinusitis and unresolving headaches are common symptoms of maxillary sinusitis. When the patient bends forward, gravity can cause the accumulated purulent fluid within the maxillary sinus to drain through the natural opening, called the ostium, into the nasal cavity. This can result in the purulent fluid pouring out of the nose.  While other sinuses, such as the anterior ethmoid air cells, frontal sinus, and posterior ethmoid air cells, can also be affected by sinusitis, the maxillary sinus is most commonly associated with the described symptoms and drainage pattern in this scenario. |
|  |  | Attempt 2  Apologies for the confusion in my previous response. Given the symptoms described, the most likely anatomical structure to be the source of the purulent fluid is:  B. Frontal sinus  The frontal sinus is located in the forehead region, above the eyes. When a person has chronic sinusitis, inflammation of the frontal sinus can occur, leading to the production of purulent fluid. Bending forward can cause the fluid to drain from the sinus and out through the nose, resulting in the described symptom of purulent fluid pouring from the nose. |
|  |  | Attempt 3  The most likely anatomical structure to be the source of the purulent fluid pouring from the nose when the man bends forward is the maxillary sinus.  The maxillary sinuses are located below the eyes and are one of the four pairs of paranasal sinuses. They are commonly affected in cases of chronic sinusitis. When the sinuses become inflamed and filled with excess mucus or pus, bending forward can cause the fluid to drain out through the nose, resulting in the symptom described.  While the other structures listed can also be involved in sinusitis, the characteristic symptom of purulent fluid pouring from the nose upon forward bending aligns with the involvement of the maxillary sinus in this scenario. |
| **34** | A 6 hour old term baby boy is grunting and cyanosed. He has been struggling to feed. His temperature is 36.8°C, pulse rate 190 bpm (120-160), respiratory rate 60 breaths per minute (30-60) and oxygen saturation 82% breathing air. His oxygen saturation does not improve significantly breathing high flow oxygen. He has a loud single second heart sound and a systolic murmur. He is treated with intravenous fluids and antibiotics.  Which is the most appropriate next medication?  A. Alprostadil  B. Ibuprofen  C. Indometacin  D. Sildenafil  E. Surfactant | Attempt 1  Based on the given clinical presentation, the most appropriate next medication for this newborn would be:  A. Alprostadil  The newborn is presenting with signs of respiratory distress and cyanosis, which may indicate a cardiac condition. The presence of a loud single second heart sound and a systolic murmur further suggests a potential congenital heart defect, such as a duct-dependent lesion. Alprostadil is a medication that helps maintain the patency of the ductus arteriosus, which is important for blood flow in certain congenital heart defects.  Administering alprostadil can help improve blood flow and oxygenation by keeping the ductus arteriosus open, allowing oxygen-rich blood from the placenta to bypass the compromised pulmonary circulation. This medication can provide temporary stabilization while further evaluation and management are planned.  It's important to note that the definitive diagnosis and management of the underlying condition should be determined by a pediatric specialist based on a thorough evaluation and additional diagnostic tests. |
|  |  | Attempt 2  Based on the symptoms and clinical presentation described, the most appropriate next medication for this 6-hour-old term baby boy is:  A. Alprostadil  The baby's symptoms and signs are suggestive of a congenital heart condition, specifically a duct-dependent lesion, where the baby's circulation depends on the patent ductus arteriosus (PDA) to maintain adequate oxygenation and perfusion. The grunting, cyanosis, high heart rate, loud single second heart sound, and systolic murmur all point towards a possible duct-dependent lesion.  Alprostadil is a medication that helps keep the ductus arteriosus open, improving blood flow to the body and the lungs. In the given scenario, administering alprostadil can help stabilize the baby's condition until further investigations and interventions can be performed.  The other options are not appropriate for the baby's condition:  B. Ibuprofen and C. Indometacin are nonsteroidal anti-inflammatory drugs (NSAIDs) that are used to close a patent ductus arteriosus in preterm infants. In this case, the baby is term, and the ductus arteriosus is required for circulation.  D. Sildenafil is used to treat pulmonary hypertension and is not suitable for this situation.  E. Surfactant is used for respiratory distress syndrome in premature infants and is not the appropriate medication for this newborn.  It's important to note that this is a hypothetical scenario, and any decisions regarding medical treatment should be made by qualified healthcare professionals based on a thorough evaluation of the patient's condition. |
|  |  | Attempt 3  Based on the given information, the most appropriate next medication for the 6-hour-old term baby boy would be Alprostadil (Option A).  The clinical presentation of grunting, cyanosis, feeding difficulties, tachycardia, tachypnea, and persistent low oxygen saturation despite high flow oxygen suggests a possible cardiac condition known as duct-dependent congenital heart disease (CHD). The loud single second heart sound and systolic murmur further support this suspicion.  Alprostadil, a prostaglandin E1 analogue, is commonly used in the management of duct-dependent CHD. It helps maintain the patency of the ductus arteriosus, a blood vessel that allows communication between the aorta and the pulmonary artery, thus ensuring adequate oxygenation. By keeping the ductus arteriosus open, alprostadil can help improve systemic blood flow and relieve cyanosis and other associated symptoms.  Therefore, in this case, administration of Alprostadil would be the most appropriate next step in the management of the baby. |
| **35** | A 5 year old girl is brought to her GP with a rash over her arms that has been gradually worsening over the past month. Her mother reports that she seems more tired than usual. She has a petechial rash over both forearms, with some petechiae over her left anterior chest. She is pale and has hepatosplenomegaly.  Which is the most appropriate management option?  A. Administer intramuscular benzylpenicillin sodium and send her to hospital in an ambulance  B. Prescribe a course of prednisolone and review in 3 days  C. Refer her for a routine appointment in the general paediatric clinic  D. Refer her urgently to the paediatric assessment unit  E. Take a full blood count and blood film and see her within 48 h | Attempt 1  Based on the given information, the most appropriate management option for the 5-year-old girl with a rash, tiredness, hepatosplenomegaly, and petechiae is to take a full blood count and blood film and see her within 48 hours (Option E).  The presence of a petechial rash, pale appearance, hepatosplenomegaly, and tiredness raises concerns about a potential underlying hematological disorder or infection. In order to further evaluate her condition, a full blood count and blood film should be obtained. These tests can provide important diagnostic information such as the complete blood cell count, platelet count, and the presence of abnormal cells. They can help in determining the cause of her symptoms and guide further management.  Although the girl's condition may not require urgent hospitalization or immediate intramuscular benzylpenicillin sodium administration, it is still necessary to evaluate her within 48 hours to ensure timely diagnosis and appropriate management. Therefore, taking a full blood count and blood film and scheduling a follow-up appointment is the most appropriate initial step in this case. |
|  |  | Attempt 2  Based on the symptoms described, the most appropriate management option for the 5-year-old girl with a rash over her arms, tiredness, petechial rash, pale appearance, and hepatosplenomegaly is:  D. Refer her urgently to the pediatric assessment unit  The presence of petechiae, pale appearance, hepatosplenomegaly, and tiredness are concerning symptoms that may indicate a serious underlying condition. These symptoms could be indicative of various hematological and systemic disorders, including but not limited to leukemia, hemolytic anemias, or other serious infections.  Given the seriousness of these symptoms, the girl needs an urgent evaluation by pediatric specialists in a pediatric assessment unit or pediatric emergency department. Immediate medical attention is necessary to identify the cause of the symptoms and initiate appropriate treatment promptly.  Options A, B, C, and E are not suitable in this case:  A. Administering intramuscular benzylpenicillin sodium and sending her to the hospital in an ambulance is not appropriate as this patient's symptoms do not suggest a bacterial infection requiring immediate antibiotics.  B. Prescribing prednisolone and reviewing in 3 days is not appropriate without a proper diagnosis. The girl's symptoms are concerning and require urgent evaluation before starting any treatment.  C. Referring her for a routine appointment in the general pediatric clinic is not suitable given the severity and urgency of her symptoms.  E. Taking a full blood count and blood film and seeing her within 48 hours is not sufficient in this case. The symptoms indicate the need for immediate evaluation and management.  Again, it's important to note that this is a hypothetical scenario, and any decisions regarding medical management should be made by qualified healthcare professionals based on a thorough evaluation of the patient's condition. |
|  |  | Attempt 3  Given the presentation of the 5-year-old girl with a rash, fatigue, petechiae, hepatosplenomegaly, and pale appearance, the most appropriate management option would be:  D. Refer her urgently to the pediatric assessment unit.  The presence of petechial rash, hepatosplenomegaly, and other concerning symptoms suggests a potentially serious underlying condition that requires immediate evaluation and management. This could be indicative of a hematological disorder such as leukemia or another serious systemic condition. Urgent referral to a pediatric assessment unit allows for a thorough assessment, including further investigations and appropriate management.  A prompt referral will ensure that the child receives timely and specialized care, as well as appropriate monitoring and treatment if necessary. |
| **36** | A 62 year old man attends the outpatients clinic with his wife. He has had reduced energy and lack of interest in his work and poor sleep for six weeks. He has lost weight. There is evidence of self-neglect and reduced eye contact. He is known to have benign prostatic enlargement.  What is the single most appropriate drug treatment?  A. Benzodiazepine  B. Selective serotonin re-uptake inhibitor  C. Serotonin noradrenaline re-uptake inhibitor  D. Tricyclic antidepressant  E. Z-drug | Attempt 1  Based on the information provided, the single most appropriate drug treatment for the 62-year-old man with reduced energy, lack of interest, poor sleep, weight loss, self-neglect, and reduced eye contact would be:  B. Selective serotonin re-uptake inhibitor (SSRI).  These symptoms are consistent with depression, and SSRIs are commonly used as first-line treatment for depression in primary care settings. SSRIs help to increase the levels of serotonin in the brain, which can improve mood, energy levels, and other depressive symptoms.  It is important to note that the choice of antidepressant medication should be made in consultation with a healthcare professional, taking into consideration the individual's specific symptoms, medical history, and potential drug interactions. Regular monitoring and follow-up should also be provided to assess the effectiveness of the treatment and manage any potential side effects. |
|  |  | Attempt 2  Based on the symptoms and presentation described, the single most appropriate drug treatment for the 62-year-old man with reduced energy, lack of interest, poor sleep, weight loss, self-neglect, and reduced eye contact is:  B. Selective serotonin re-uptake inhibitor (SSRI)  The combination of symptoms, including reduced energy, lack of interest, poor sleep, weight loss, and self-neglect, are suggestive of major depressive disorder (MDD) in this patient. SSRIs are considered the first-line treatment for MDD in adults, including elderly patients, due to their effectiveness and relatively favorable side effect profile.  Benzodiazepines (Option A) are not recommended as the first-line treatment for depression because they can be habit-forming and are associated with the risk of abuse and dependence.  Serotonin noradrenaline re-uptake inhibitors (Option C) and tricyclic antidepressants (Option D) are also used in the treatment of depression, but SSRIs are generally preferred in the elderly population due to their safety profile and lower risk of side effects.  Z-drugs (Option E), such as Zolpidem, are primarily used for the treatment of insomnia and are not considered appropriate for addressing the broader symptoms of depression and lack of interest in work.  It's important to note that any treatment decisions should be made in consultation with a qualified healthcare professional who can conduct a thorough evaluation and tailor the treatment plan based on the patient's specific needs and medical history. |
|  |  | Attempt 3  Based on the information provided, the single most appropriate drug treatment for the 62-year-old man with symptoms of reduced energy, lack of interest, poor sleep, weight loss, self-neglect, and reduced eye contact would be a selective serotonin re-uptake inhibitor (SSRI) (Option B).  SSRIs are commonly used as first-line treatment for depression. They work by increasing the availability of serotonin in the brain, which helps improve mood and alleviate depressive symptoms. SSRIs are generally well-tolerated and have a favorable side effect profile compared to older antidepressants such as tricyclic antidepressants.  In this case, the patient's symptoms, which include low mood, loss of interest, and poor sleep, are suggestive of depression. Weight loss and self-neglect further raise concerns about his well-being. Considering his symptoms and the absence of contraindications, initiating treatment with an SSRI would be a reasonable approach.  It's important to note that the choice of antidepressant should be based on a thorough assessment by a healthcare professional, taking into account the patient's specific symptoms, medical history, and any potential drug interactions. Close monitoring and follow-up are necessary to evaluate the response to treatment and adjust the dosage if needed. |
| **37** | A doctor is approached by their neighbour who is aware that another person on their street has been admitted to the ward the doctor is working on. They wish to know how the patient is doing. The doctor is aware the patient is stable but the prognosis is uncertain.  Which is the most appropriate response by the doctor?  A. They can confirm they are caring for him and that he is unwell but stable  B. They can confirm they are caring for him but cannot comment on his condition  C. They can confirm they are caring for him and that he is making good progress  D. They cannot confirm they are caring for him but have heard he is unwell but stable  E. They cannot confirm who is caring for him and cannot comment on his condition | Attempt 1  The most appropriate response by the doctor in this situation would be:  E. They cannot confirm who is caring for him and cannot comment on his condition.  Patient confidentiality is a fundamental principle in healthcare, and disclosing any information about a patient's condition without their consent would be a breach of that confidentiality. Even though the doctor is aware of the patient's general condition, it is not appropriate to discuss it with someone who is not directly involved in the patient's care or who does not have a legitimate need to know the information. Respecting patient privacy and maintaining confidentiality is essential in maintaining trust and ethical standards in healthcare. |
|  |  | Attempt 2  The most appropriate response by the doctor in this situation is:  E. They cannot confirm who is caring for him and cannot comment on his condition.  As a healthcare professional, it is essential to maintain patient confidentiality and adhere to privacy regulations. Revealing any information about a patient's condition, even if it seems innocuous or if the inquirer is a neighbor, is a violation of patient confidentiality.  The doctor should avoid confirming whether they are caring for the patient or discussing the patient's condition with anyone who is not directly involved in the patient's care. Even if the patient's condition is stable, providing any information about the patient's health status without proper authorization could breach patient confidentiality and lead to serious ethical and legal consequences.  To protect patient privacy, the doctor should politely inform their neighbor that they cannot share any information about the patient's condition and direct them to contact the patient's family or the hospital for updates if appropriate. |
|  |  | Attempt 3  The most appropriate response by the doctor in this situation would be option E: They cannot confirm who is caring for him and cannot comment on his condition.  Patient confidentiality is a fundamental ethical principle in healthcare. Disclosing any patient information, including their condition or progress, without the patient's explicit consent would be a violation of their privacy rights. Even if the doctor is aware of the patient's condition and prognosis, it is not appropriate to share that information with a neighbor or anyone else who does not have a legitimate need to know.  The doctor should maintain professionalism and respect for patient privacy by refraining from providing any information about the patient's condition. It is important to uphold patient confidentiality and trust, even in casual or informal situations outside of the healthcare setting. |
| **38** | A 40 year old woman has had right-sided facial weakness for the past 12 hours. She has weakness of all of the muscles on the right side of her face. There is no limb weakness and speech is normal. Examination of her ears is normal.  Which is the most appropriate drug treatment?  A. Aciclovir  B. Aspirin  C. Cinnarizine  D. None  E. Prednisolone | Attempt 1  The most appropriate drug treatment in this case would be:  E. Prednisolone  The presentation of right-sided facial weakness in this patient is consistent with peripheral facial nerve palsy, also known as Bell's palsy. Prednisolone, a corticosteroid, is commonly used in the management of Bell's palsy to reduce inflammation and swelling of the facial nerve. Treatment with prednisolone within 72 hours of symptom onset has been shown to improve the chances of recovery and reduce the duration of symptoms. Therefore, initiating treatment with prednisolone is recommended in this scenario. |
|  |  | Attempt 2  Based on the symptoms and presentation described, the most appropriate drug treatment for the 40-year-old woman with right-sided facial weakness, weakness of all facial muscles, and no limb weakness is:  E. Prednisolone  The clinical scenario suggests a diagnosis of Bell's palsy, which is a form of facial paralysis caused by inflammation of the facial nerve (cranial nerve VII). The characteristic feature of Bell's palsy is the sudden onset of unilateral facial weakness or paralysis.  Prednisolone is the standard treatment for Bell's palsy. It is a corticosteroid that helps reduce inflammation and edema around the affected facial nerve, potentially improving nerve function and accelerating recovery. Studies have shown that treatment with prednisolone within the first 72 hours of symptom onset is associated with better outcomes and a higher likelihood of full recovery.  The other options are not appropriate for this specific condition:  A. Aciclovir is an antiviral medication used to treat herpes viral infections, but its role in Bell's palsy is limited, and it is not considered a first-line treatment.  B. Aspirin is a pain reliever and anti-inflammatory drug, but it is not used as the primary treatment for Bell's palsy.  C. Cinnarizine is an antihistamine used to treat motion sickness and vestibular disorders, but it is not the appropriate treatment for Bell's palsy.  D. None of the treatments are not recommended in this case because Bell's palsy usually benefits from medical intervention to improve outcomes.  As with any medical condition, it is important for the woman to consult a healthcare professional for a proper evaluation, diagnosis, and management plan tailored to her specific needs. |
|  |  | Attempt 3  The most appropriate drug treatment in this scenario would be option E: Prednisolone.  The patient's presentation is consistent with acute onset of facial weakness, which is a common manifestation of Bell's palsy. Bell's palsy is thought to be due to inflammation and swelling of the facial nerve, leading to its dysfunction. The use of corticosteroids, such as prednisolone, has been shown to be beneficial in the treatment of Bell's palsy. It is recommended to initiate treatment with oral prednisolone within 72 hours of symptom onset to improve outcomes and accelerate recovery.  Aciclovir (option A) is an antiviral medication that is primarily used for the treatment of herpes viral infections, such as herpes zoster (shingles) or herpes simplex. However, Bell's palsy is thought to be primarily caused by inflammation rather than viral infection, so antiviral therapy is not routinely recommended.  Aspirin (option B) is a nonsteroidal anti-inflammatory drug (NSAID) and is not indicated for the treatment of Bell's palsy. Its use in this case is not supported by current guidelines.  Cinnarizine (option C) is a medication primarily used for the treatment of vertigo and motion sickness. It is not indicated for the treatment of Bell's palsy.  Option D (None) is not the most appropriate choice because treatment with corticosteroids, such as prednisolone, has been shown to be beneficial in Bell's palsy and is recommended in order to improve outcomes.  It's important to note that this response is based on the information provided, and a thorough evaluation by a healthcare professional is recommended for an accurate diagnosis and appropriate treatment. |
| **39** | A 35 year old woman with gestational hypertension is induced at 40 weeks. She has a normal vaginal delivery, but following delivery of the placenta she bleeds heavily.  Which is the most appropriate management?  A. Carboprost  B. Ergometrine  C. Oxytocin  D. Misoprostol  E. Tranexamic acid | Attempt 1  The most appropriate management in this case would be:  C. Oxytocin  Postpartum hemorrhage (PPH) is a significant complication following delivery, and prompt management is crucial. Oxytocin is the first-line treatment for PPH as it helps to contract the uterus, reducing bleeding. It is commonly administered as an intravenous infusion or intramuscular injection. Oxytocin helps to control bleeding by promoting uterine contractions and reducing the risk of atonic uterus.  Carboprost, Ergometrine, Misoprostol, and Tranexamic acid are also options for managing PPH, but they are typically used when oxytocin alone is insufficient or contraindicated. The choice of specific medication depends on the severity of bleeding and individual patient factors. However, oxytocin is the most appropriate initial management option in this scenario. |
|  |  | Attempt 2  In a woman who experiences heavy bleeding following delivery of the placenta (postpartum hemorrhage), the most appropriate management is:  C. Oxytocin  Oxytocin is the first-line treatment for postpartum hemorrhage. It is a uterotonic agent that helps contract the uterus, reducing bleeding and preventing further hemorrhage. Oxytocin is routinely administered during the third stage of labor (delivery of the placenta) to reduce the risk of postpartum hemorrhage. If heavy bleeding occurs after delivery, additional oxytocin may be given to further stimulate uterine contractions and control the bleeding.  The other options may also have a role in managing postpartum hemorrhage but are not the first-line treatments:  A. Carboprost (15-methyl prostaglandin F2alpha) is a prostaglandin analogue and can be used if oxytocin is ineffective or unavailable for controlling postpartum hemorrhage.  B. Ergometrine (methylergometrine) is an ergot alkaloid that can be used if oxytocin is not effective or contraindicated, but it is not the first-line treatment.  D. Misoprostol is a prostaglandin E1 analogue and can be an alternative option for managing postpartum hemorrhage, but it is generally used when other uterotonics are unavailable or contraindicated.  E. Tranexamic acid is an antifibrinolytic agent that can be used to reduce bleeding, but it is typically reserved for cases of postpartum hemorrhage that are unresponsive to uterotonics and other measures.  The appropriate treatment for postpartum hemorrhage should be determined by a healthcare professional based on the severity of bleeding and the specific clinical situation. In emergencies, immediate medical attention is crucial to manage postpartum hemorrhage effectively and prevent complications. |
|  |  | Attempt 3  The most appropriate management for a postpartum hemorrhage in this scenario would be option C: Oxytocin.  Postpartum hemorrhage is defined as excessive bleeding following delivery, and it is a major cause of maternal morbidity and mortality. Oxytocin, a uterotonic medication, is the first-line treatment for postpartum hemorrhage. It stimulates uterine contractions, helps the uterus to contract and control bleeding, and reduces the risk of further complications.  Carboprost (option A) is a prostaglandin analog and can be used as a second-line treatment if oxytocin fails to control the bleeding. Ergometrine (option B) is another uterotonic agent that can be used as an alternative, but it is contraindicated in patients with hypertension.  Misoprostol (option D) is also a prostaglandin analog that can be used as an alternative if other options are not available, but it is typically used in settings where other uterotonic medications are not accessible.  Tranexamic acid (option E) is an antifibrinolytic agent that can be used to control bleeding, including postpartum hemorrhage. However, it is generally used in cases where other measures have failed to control the bleeding or in cases of severe hemorrhage.  It's important to note that the management of postpartum hemorrhage should be individualized based on the specific clinical circumstances and healthcare provider's judgment. Immediate medical attention and consultation with a healthcare professional are crucial in managing postpartum hemorrhage effectively. |
| **40** | A 19 year old man has had two days of a burning sensation when passing urine and a penile discharge. This developed three days after he had unprotected sex with a new female partner.  Which is the most appropriate treatment?  A. Azithromycin and ceftriaxone  B. Ciprofloxacin and metronidazole  C. Fluconazole and penicillin V  D. Metronidazole and fluconazole  E. Penicillin V and azithromycin | Attempt 1  The most appropriate treatment for this 19-year-old man with symptoms of a burning sensation when passing urine and penile discharge, following unprotected sex with a new partner, is:  A. Azithromycin and ceftriaxone  These symptoms are suggestive of a sexually transmitted infection (STI), particularly gonorrhea and chlamydia, which are common causes of urethritis in young sexually active individuals. The recommended treatment for this dual infection is a combination of azithromycin and ceftriaxone.  Azithromycin is effective against chlamydia, while ceftriaxone is effective against gonorrhea. Treating both infections simultaneously is important to ensure comprehensive management and reduce the risk of complications.  It is important for the individual to receive appropriate treatment and for any sexual partners within the past 60 days to be identified and treated as well to prevent further transmission and reinfection. |
|  |  | Attempt 2  The most appropriate treatment for a 19-year-old man with symptoms of a burning sensation when passing urine and a penile discharge, which developed after having unprotected sex with a new female partner, is:  A. Azithromycin and ceftriaxone  These symptoms are suggestive of a sexually transmitted infection (STI), most commonly gonorrhea and chlamydia. The recommended treatment for these two common STIs is a combination of antibiotics to cover both infections effectively.  Azithromycin is used to treat chlamydia infection, while ceftriaxone is used to treat gonorrhea. Combining these two antibiotics provides broad-spectrum coverage against both infections and is the standard treatment recommended by current guidelines.  The other options are not appropriate for this specific case:  B. Ciprofloxacin and metronidazole are not the first-line treatment for gonorrhea and chlamydia.  C. Fluconazole and penicillin V are used for fungal and bacterial infections, respectively, but they do not cover gonorrhea and chlamydia.  D. Metronidazole and fluconazole are used for different types of infections (bacterial and fungal) and do not cover gonorrhea and chlamydia.  E. Penicillin V and azithromycin would only cover syphilis and chlamydia, respectively, but not gonorrhea, which is an important consideration in this case.  It's important to remember that any treatment for a suspected STI should be prescribed by a healthcare professional after a proper evaluation and testing. Also, both sexual partners should be tested and treated to prevent reinfection and further spread of the infection. |
|  |  | Attempt 3  The most appropriate treatment for a 19-year-old man with symptoms of a burning sensation when passing urine and penile discharge, following unprotected sex with a new female partner, is option A: Azithromycin and ceftriaxone.  These symptoms are suggestive of a sexually transmitted infection (STI), particularly gonorrhea and chlamydia, which are common causes of urethritis in men. The recommended first-line treatment for these infections is a combination of antibiotics, specifically:  - Azithromycin: It is effective against chlamydia and covers for potential co-infection with Mycoplasma genitalium.  - Ceftriaxone: It is effective against gonorrhea and provides coverage for potential co-infection with chlamydia.  Therefore, azithromycin and ceftriaxone are the most appropriate treatment options in this scenario. It is important to treat both infections simultaneously to ensure adequate coverage and reduce the risk of complications.  Option B (Ciprofloxacin and metronidazole) is not the first-line treatment for gonorrhea and chlamydia. Ciprofloxacin is no longer recommended as a first-line treatment for these infections due to increasing antimicrobial resistance.  Options C (Fluconazole and penicillin V) and D (Metronidazole and fluconazole) are not appropriate for treating gonorrhea and chlamydia, as they do not target these specific infections.  Option E (Penicillin V and azithromycin) does not cover the treatment of gonorrhea, which is an important consideration given the patient's sexual history.  It's important for the patient to seek prompt medical attention and receive appropriate treatment to prevent complications and reduce the risk of transmitting the infection to others. |
| **41** | A 9 month old baby boy is brought to his GP with 4 days of coryzal symptoms, intermittent fever, coughing and wheezing. He has been taking smaller feeds for the past 2 days and was unsettled last night. He is alert, there is no rash and he is drooling. He has adequate oral intake. His mother has given him paracetamol suspension. His temperature is 38.2°C, capillary return 1 second, pulse rate 148 bpm (80-160), respiratory rate 60 breaths per minute (30-60) and oxygen saturation 97% in air. He has widespread wheeze and crackles, and his ears and throat are normal.  Which is the most appropriate next step in management?  A. Admission to paediatric ward  B. Prescribe oral doxycycline  C. Prescribe oral co-amoxiclav  D. Reassure and give safety netting advice  E. Give nebulised salbutamol and review | Attempt 1  In a 9-month-old baby boy with coryzal symptoms, intermittent fever, coughing, wheezing, decreased oral intake, and signs of respiratory distress, the most appropriate next step in management is:  E. Give nebulized salbutamol and review  The presence of wheezing and crackles, along with the respiratory distress, suggests possible bronchiolitis, which is commonly caused by respiratory syncytial virus (RSV) in infants. Nebulized salbutamol, a bronchodilator, can help alleviate the symptoms and improve respiratory function in some cases of bronchiolitis.  It is important to note that not all infants with bronchiolitis will respond to bronchodilator therapy, and the decision to initiate treatment should be based on clinical judgment. The baby should be closely monitored for signs of worsening respiratory distress and hydration status.  Admission to the pediatric ward may be necessary if the baby's condition worsens, if there are signs of severe respiratory distress, or if there are concerns about hydration or other complications.  Prescribing oral doxycycline or co-amoxiclav is not appropriate in this case, as they are not indicated for bronchiolitis, which is typically a viral infection. Reassurance and safety netting advice alone may not be sufficient if the respiratory distress worsens. Therefore, the most appropriate next step is to initiate nebulized salbutamol and closely monitor the baby's response. |
|  |  | Attempt 2  Based on the symptoms and presentation described, the most appropriate next step in the management of the 9-month-old baby boy is:  E. Give nebulized salbutamol and review  The baby's symptoms, including coryzal symptoms, intermittent fever, coughing, wheezing, and widespread wheeze and crackles on examination, are suggestive of bronchiolitis. Bronchiolitis is a common viral infection of the lower respiratory tract that often affects infants and young children, especially during the winter months.  Nebulized salbutamol is indicated in the management of bronchiolitis if there is evidence of significant wheezing. It helps to relieve bronchospasm and improve airflow in the lower airways. However, it's important to note that the response to salbutamol can vary, and not all infants with bronchiolitis will show a significant response.  The other options are not appropriate for bronchiolitis:  A. Admission to the pediatric ward may be required in severe cases of bronchiolitis with respiratory distress, significant oxygen requirement, or signs of dehydration. However, based on the description provided, the baby's symptoms do not appear to be severe enough to warrant immediate admission.  B. Prescribing oral doxycycline is not appropriate for bronchiolitis. Doxycycline is an antibiotic used to treat bacterial infections and is not effective against viral infections like bronchiolitis.  C. Prescribing oral co-amoxiclav is also not appropriate for bronchiolitis. Co-amoxiclav is an antibiotic, and as mentioned earlier, bronchiolitis is caused by a viral infection, not bacteria.  D. Reassuring the parent and providing safety netting advice is essential in every consultation. However, in this case, the baby's symptoms of bronchiolitis warrant further intervention with nebulized salbutamol and a follow-up review.  Always consult a qualified healthcare professional for the appropriate evaluation and management of any medical condition. |
|  |  | Attempt 3  The most appropriate next step in the management of a 9-month-old baby boy with coryzal symptoms, intermittent fever, coughing, wheezing, and signs of respiratory distress would be option E: Give nebulized salbutamol and review.  Based on the symptoms described, the baby is presenting with symptoms suggestive of a lower respiratory tract infection, possibly bronchiolitis. The presence of wheezing, crackles, and respiratory distress indicates the involvement of the lower airways. Nebulized salbutamol is a bronchodilator commonly used in the treatment of bronchiolitis and can help alleviate the wheezing and improve respiratory function. Therefore, initiating nebulized salbutamol is an appropriate step in managing this condition.  Options A (Admission to pediatric ward), B (Prescribe oral doxycycline), and C (Prescribe oral co-amoxiclav) are not indicated in this case. Hospital admission is typically reserved for severe cases of bronchiolitis or when there are concerns about hydration, oxygenation, or respiratory distress. Antibiotics such as doxycycline and co-amoxiclav are not indicated for bronchiolitis since it is primarily caused by viral infections, and antibiotics are not effective against viruses.  Option D (Reassure and give safety netting advice) may be appropriate if the symptoms are mild and the baby is well overall. However, given the presence of wheezing and respiratory distress, providing symptomatic relief with nebulized salbutamol is warranted, and close follow-up is necessary to monitor the baby's progress.  It is important for the baby's parents to follow up with the GP or seek medical attention if there is a worsening of symptoms, persistent respiratory distress, decreased oral intake, or any other concerning signs.  Note: The specific management approach may vary depending on local guidelines and clinical judgment. It is always important to consider individual patient factors and consult with a healthcare professional for personalized advice. |
| **42** | A 23 year old woman has barricaded herself with her baby in the bathroom, shouting that it is unsafe to come out because the house is infected. She thinks that someone is trying to kill her and her baby. She is four days post-caesarean section. Her mood has not been low at any stage. She was not on any medication during her pregnancy, but drank 20 units of alcohol a week. Her physical recovery from caesarean section has been uncomplicated. She is orientated in time and place.  Which is the most likely diagnosis?  A. Alcohol withdrawal  B. Delirium  C. Obsessive-compulsive disorder  D. Post-natal depression  E. Puerperal psychosis | Attempt 1  The most likely diagnosis for the 23-year-old woman in this case is:  E. Puerperal psychosis  Puerperal psychosis is a severe psychiatric disorder that occurs in the postpartum period, typically within the first few weeks after delivery. It is characterized by the onset of psychotic symptoms such as delusions, hallucinations, and disorganized thinking. The symptoms can be severe and may pose a risk to the mother and the baby.  The woman's symptoms of believing that someone is trying to kill her and her baby, along with her barricading herself in the bathroom due to perceived infection, are consistent with delusions and paranoia, which are common features of puerperal psychosis. It is important to note that puerperal psychosis is a psychiatric emergency and requires immediate attention and intervention.  Alcohol withdrawal may present with symptoms such as tremors, agitation, anxiety, and seizures, but it is less likely in this case as the woman's symptoms are primarily related to delusions and paranoia.  Delirium is characterized by acute onset confusion, disorientation, fluctuating levels of consciousness, and impaired attention. The woman in this case is orientated in time and place, suggesting that delirium is less likely.  Obsessive-compulsive disorder (OCD) is characterized by recurrent obsessions and compulsions that cause distress and interfere with daily functioning. The symptoms described in the case are not consistent with OCD, as the woman's beliefs are not characterized by obsessions and compulsions.  Post-natal depression may present with symptoms of low mood, loss of interest, fatigue, and feelings of worthlessness. The woman's mood has not been low at any stage, and her symptoms are primarily related to psychotic features, making post-natal depression less likely.  Given the severity and potential risk associated with puerperal psychosis, immediate medical evaluation and intervention are necessary to ensure the safety and well-being of both the mother and the baby. |
|  |  | Attempt 2  Based on the symptoms and presentation described, the most likely diagnosis for the 23-year-old woman barricading herself with her baby in the bathroom and displaying paranoid thoughts is:  E. Puerperal psychosis  Puerperal psychosis, also known as postpartum psychosis, is a severe and rare psychiatric disorder that can occur in the postnatal period. It typically begins within the first few weeks after childbirth and is characterized by acute and severe psychiatric symptoms, including delusions, hallucinations, agitation, paranoia, and disorganized behavior. The symptoms of puerperal psychosis can be very distressing and may pose a risk to both the woman and her baby.  Key features of puerperal psychosis in this case include the acute onset of symptoms, delusions of someone trying to harm her and her baby, and the fact that her mood has not been low at any stage. Puerperal psychosis is a medical emergency and requires immediate evaluation and intervention by mental health professionals.  The other options are less likely based on the information provided:  A. Alcohol withdrawal may present with symptoms such as agitation, tremors, and confusion, but it is less likely in this case because the woman's symptoms are not primarily related to alcohol withdrawal, and she does not have a history of alcohol use disorder.  B. Delirium typically presents with acute onset confusion, altered consciousness, and impaired attention. While delirium is a possibility, the presence of delusions and paranoid thoughts makes puerperal psychosis a more likely diagnosis.  C. Obsessive-compulsive disorder (OCD) is a chronic condition characterized by obsessions and compulsions. While OCD can occur in the postpartum period, the symptoms described in this case are more consistent with puerperal psychosis.  D. Post-natal depression (postpartum depression) is characterized by low mood, feelings of sadness, and loss of interest in activities. The woman's mood has not been low, making post-natal depression less likely.  It is important to remember that the diagnosis and appropriate management of any psychiatric condition should be made by qualified mental health professionals based on a comprehensive evaluation of the individual's symptoms and history. If you encounter a situation like this, please seek immediate medical assistance for the woman and her baby's safety. |
|  |  | Attempt 3  The most likely diagnosis for the 23-year-old woman presenting with symptoms of paranoia, delusions, and barricading herself with her baby in the bathroom, four days post-caesarean section, is E. Puerperal psychosis.  Puerperal psychosis is a severe psychiatric disorder that occurs in the postpartum period, typically within the first few weeks after delivery. It is characterized by the onset of psychotic symptoms such as delusions, hallucinations, and disorganized thinking or behavior. The symptoms can be severe and may include thoughts of harming oneself or the baby, as seen in this case.  Risk factors for puerperal psychosis include a personal or family history of mental illness, previous episodes of postpartum psychosis, and substance abuse. The fact that the woman had been drinking 20 units of alcohol per week during her pregnancy may have contributed to her vulnerability to developing this condition.  It is important to recognize puerperal psychosis as a medical emergency due to the potential risk to the woman and her baby. Urgent psychiatric assessment and intervention are required, including the consideration of hospital admission for the woman's safety and initiation of appropriate treatment, such as antipsychotic medications.  While alcohol withdrawal (option A) can cause delirium and psychotic symptoms, the presence of symptoms directly related to childbirth and the absence of a history of alcohol dependence suggest that puerperal psychosis is a more likely diagnosis in this case.  Delirium (option B) is characterized by acute onset confusion and fluctuating levels of consciousness. It is less likely in this case because the woman is orientated in time and place.  Obsessive-compulsive disorder (option C) is characterized by intrusive thoughts (obsessions) and repetitive behaviors (compulsions) that cause distress and interfere with daily functioning. The symptoms described in this case, such as paranoia and delusions, are not typical of obsessive-compulsive disorder.  Postnatal depression (option D) typically presents with low mood, loss of interest or pleasure, fatigue, changes in appetite or sleep patterns, and feelings of guilt or worthlessness. While the woman's mood is not mentioned, the presence of paranoid delusions and psychotic symptoms is more suggestive of puerperal psychosis.  It is essential for the woman's safety and the well-being of her baby that she receives prompt evaluation and appropriate treatment by a healthcare professional specializing in mental health. |
| **43** | A 20 year old woman seeks contraceptive advice. She had unprotected sexual intercourse four days ago.  Which is the most appropriate method of contraception?  A. Combined oral contraceptive pill  B. Copper-containing intrauterine device  C. Depot progestogen injection  D. High dose oral progestogen  E. Intrauterine progestogen system (Mirena coil) | Attempt 1  The most appropriate method of contraception in this case would be A. Combined oral contraceptive pill.  The combined oral contraceptive pill (COCP) is a highly effective and reversible method of contraception. When taken correctly, it provides reliable protection against unintended pregnancy. In this scenario, the woman had unprotected sexual intercourse four days ago, and the COCP can still be initiated as a form of emergency contraception within a specific time frame.  It is important to note that the effectiveness of emergency contraception decreases over time, so it is crucial to provide timely advice and intervention. The COCP can be started up to 120 hours (5 days) after unprotected sex, although it is most effective when taken as soon as possible within the first 72 hours (3 days).  Other contraceptive methods, such as the copper-containing intrauterine device (option B), depot progestogen injection (option C), high-dose oral progestogen (option D), or intrauterine progestogen system (Mirena coil, option E), are not typically used as emergency contraception in the scenario described. These methods may have their own advantages and considerations for ongoing contraception, but they are not primarily intended for emergency use immediately following unprotected sexual intercourse.  It is important for the woman to consult with a healthcare professional to discuss her options, receive appropriate counseling, and ensure she understands the risks, benefits, and correct usage of the chosen contraceptive method. |
|  |  | Attempt 2  The most appropriate method of contraception for a 20-year-old woman seeking contraceptive advice after having unprotected sexual intercourse four days ago is:  B. Copper-containing intrauterine device (IUD)  The copper-containing intrauterine device (Cu-IUD) is an effective form of emergency contraception when inserted within five days (120 hours) of unprotected sexual intercourse. It is one of the most effective emergency contraceptive options available, with a failure rate of less than 1%.  The Cu-IUD works by preventing fertilization of the egg and inhibiting implantation of a fertilized egg in the uterus. It does not have any hormonal effects and can be used as ongoing contraception if the woman chooses to continue using it.  The other options mentioned are not appropriate for emergency contraception in this scenario:  A. Combined oral contraceptive pill (COC): COCs can be used as emergency contraception, but they need to be taken as soon as possible after unprotected intercourse. In this case, four days have already passed, and the efficacy of COCs as emergency contraception decreases significantly after the first 72 hours.  C. Depot progestogen injection: The depot progestogen injection can be used for ongoing contraception, but it is not typically recommended for emergency contraception.  D. High-dose oral progestogen: High-dose oral progestogen is not commonly used for emergency contraception, and the Cu-IUD is a more effective option in this situation.  E. Intrauterine progestogen system (Mirena coil): The intrauterine progestogen system (Mirena coil) is primarily used for long-term contraception and is not commonly used for emergency contraception.  It's important to note that emergency contraception is most effective when used as soon as possible after unprotected intercourse. If more than five days have already passed since the unprotected intercourse, the woman should still consider using regular contraception to prevent future unintended pregnancies. In either case, she should consult a healthcare professional for personalized advice and guidance on choosing the best contraceptive method for her needs. |
|  |  | Attempt 3  The most appropriate method of contraception in this case would be:  B. Copper-containing intrauterine device (IUD)  The copper-containing intrauterine device, commonly known as the copper IUD, is a highly effective form of emergency contraception when inserted within a few days after unprotected sexual intercourse. It can be used up to five days (120 hours) after unprotected sex. The copper IUD works by inhibiting fertilization and preventing implantation of a fertilized egg.  Other options for emergency contraception include the use of emergency contraceptive pills (levonorgestrel or ulipristal acetate) or the use of high-dose oral progestogen. However, the copper IUD is the most effective method with the longest window of effectiveness and can also serve as ongoing contraception if the woman wishes to continue using it.  Combined oral contraceptive pills can be used as regular contraception but are not recommended for emergency contraception in this scenario.  Depot progestogen injection is a long-acting contraceptive method that provides continuous contraception for several weeks or months. It is not typically used for emergency contraception.  The intrauterine progestogen system (Mirena coil) is an effective long-acting contraceptive method, but it is not commonly used for emergency contraception. It is more suitable for long-term contraception rather than as an emergency option.  It's important to note that the woman should consult with a healthcare professional to discuss her options and make an informed decision based on her specific circumstances and preferences. |
| **44** | A 60 year old man feels tired, but has no specific symptoms. He is a non-smoker and drinks eight units of alcohol per week. Investigations: Haemoglobin 120 g/L (130–175) White cell count 5.8 × 109/L (3.0–10.0) Platelets 170 × 109/L (150–400) MCV 110 fL (80–96) TSH 2.0 mU/L (0.3–4.2)  Which additional investigation is most likely to help with the diagnosis?  A. Erythrocyte sedimentation rate  B. Ferritin  C. Folate and vitamin B 12  D. Haptoglobin  E. Protein electrophoresis | Attempt 1  Based on the provided information, the most appropriate additional investigation to help with the diagnosis in this case is:  C. Folate and vitamin B12 levels  The patient's presentation of tiredness and the elevated mean corpuscular volume (MCV) of 110 fL suggest the possibility of megaloblastic anemia. Megaloblastic anemia can be caused by deficiencies in vitamin B12 or folate. Both deficiencies can lead to similar symptoms and an elevated MCV.  Measuring the levels of folate and vitamin B12 in the blood can help differentiate between the two deficiencies. Low levels of folate or vitamin B12 would indicate the respective deficiency as the likely cause of the patient's symptoms and abnormal MCV. Treatment would then focus on correcting the specific deficiency through dietary modifications or supplementation.  The other investigations listed may be helpful in certain situations but are less likely to provide specific insights into the patient's current presentation. Erythrocyte sedimentation rate (A) is a non-specific marker of inflammation and would not directly address the underlying cause of the tiredness. Ferritin (B) is a marker of iron stores and would be useful in suspected iron deficiency anemia, but it is not specifically indicated in this case. Haptoglobin (D) and protein electrophoresis (E) are not typically part of the initial evaluation for tiredness and anemia.  However, it's important to note that a comprehensive evaluation and clinical assessment by a healthcare professional are necessary to determine the underlying cause of the patient's symptoms and abnormal blood test results. |
|  |  | Attempt 2  Based on the given information, the 60-year-old man is experiencing tiredness and has some abnormal blood test results. The relevant abnormal blood test results are the following:  1. Hemoglobin: 120 g/L (normal range: 130–175 g/L)  2. Mean corpuscular volume (MCV): 110 fL (normal range: 80–96 fL)  Considering these results, the most likely investigation to help with the diagnosis is:  C. Folate and vitamin B12 levels  The combination of low hemoglobin (anemia) and elevated MCV (macrocytosis) suggests a possible megaloblastic anemia, which is commonly caused by deficiencies of folate or vitamin B12. By measuring folate and vitamin B12 levels, it would be possible to identify if one of these deficiencies is the underlying cause of the patient's symptoms. This additional investigation will help in confirming or ruling out megaloblastic anemia and guide further management.  Option A (Erythrocyte sedimentation rate) is not directly related to the abnormal blood test results in this case and would not provide information specific to the cause of the anemia.  Option B (Ferritin) is useful for assessing iron stores and iron deficiency anemia, but it does not provide insights into the cause of the macrocytic anemia observed in this patient.  Option D (Haptoglobin) is not directly related to the patient's symptoms and abnormal blood test results and would not help with the diagnosis in this case.  Option E (Protein electrophoresis) is useful for identifying abnormal protein patterns and diagnosing conditions like multiple myeloma or other plasma cell disorders. However, it is not relevant to the specific presentation of macrocytic anemia in this case.  Remember that the best course of action in any medical situation is to consult with a qualified healthcare professional for proper evaluation and diagnosis. They will be able to consider the patient's full medical history, perform a physical examination, and order additional tests as needed to arrive at a definitive diagnosis and appropriate treatment plan. |
|  |  | Attempt 3  In this case, the most appropriate additional investigation to help with the diagnosis is C. Folate and vitamin B12 levels.  The patient's presentation of fatigue and macrocytic anemia (elevated MCV) raises suspicion of megaloblastic anemia, which can be caused by deficiencies in folate or vitamin B12. Both folate and vitamin B12 are essential for normal red blood cell production.  Measuring the levels of folate and vitamin B12 in the blood can provide valuable information regarding their status and help identify the underlying cause of the anemia. Folate deficiency can be caused by inadequate dietary intake, malabsorption, or increased requirements, while vitamin B12 deficiency is often due to impaired absorption (e.g., pernicious anemia) or dietary deficiency.  Other options for investigation, such as erythrocyte sedimentation rate (option A), ferritin (option B), haptoglobin (option D), or protein electrophoresis (option E), are not specific to the suspected diagnosis of megaloblastic anemia. These tests may be useful in other clinical scenarios or when investigating other potential causes of anemia or systemic conditions.  It is important to consider the patient's clinical history, physical examination findings, and any other relevant investigations to guide the overall diagnostic approach and management plan. Consulting with a healthcare professional for further evaluation and interpretation of the test results is recommended. |
| **45** | A 15 year old girl has delayed puberty. She has always been short for her age (height <0.4th centile for age). She has no dysmorphic facial features but does have a webbed neck and widely spaced nipples. She has no breast development, and has not started her periods yet.  Which chromosomal abnormality is most likely to cause her clinical presentation?  A. 45X0  B. 47XXX  C. 47XXY  D. Trisomy 13  E. Trisomy 21 | Attempt 1  The chromosomal abnormality most likely to cause the clinical presentation described in the case is:  A. 45X0 (Turner syndrome)  Turner syndrome is characterized by the complete or partial absence of one X chromosome in females. The clinical features associated with Turner syndrome can vary, but common findings include delayed puberty, short stature, webbed neck, widely spaced nipples, and lack of breast development. Additionally, individuals with Turner syndrome often have primary ovarian failure, resulting in infertility and absence of menstrual periods.  The other options listed are associated with different chromosomal abnormalities and present with different clinical features:  - 47XXX (Option B) is Trisomy X and typically does not cause delayed puberty or the specific physical features described.  - 47XXY (Option C) is Klinefelter syndrome, which affects males and is not associated with delayed puberty in females or the specific physical features described.  - Trisomy 13 (Option D) is associated with Patau syndrome, which has distinct clinical features including severe developmental abnormalities and intellectual disability. It is not typically associated with delayed puberty or the specific physical features described.  - Trisomy 21 (Option E) is Down syndrome, which has characteristic features such as intellectual disability, distinct facial features, and certain medical complications. It is not typically associated with delayed puberty or the specific physical features described.  Therefore, based on the information provided, the most likely chromosomal abnormality causing the girl's clinical presentation is Turner syndrome (45X0). It's important to note that a comprehensive evaluation by a healthcare professional, including further diagnostic tests and assessments, would be necessary to confirm the diagnosis. |
|  |  | Attempt 2  The clinical presentation of a 15-year-old girl with delayed puberty, short stature, webbed neck, widely spaced nipples, no breast development, and no menstruation suggests a specific chromosomal abnormality known as Turner syndrome. The most likely chromosomal abnormality that causes this clinical presentation is:  A. 45X0 (Turner syndrome)  Turner syndrome, also known as 45,X, is a genetic condition where a female is born with only one X chromosome instead of the usual two (XX). The typical features of Turner syndrome include short stature (often below the 5th percentile), delayed puberty, webbed neck, widely spaced nipples, and lack of breast development. Additionally, girls with Turner syndrome typically do not undergo normal puberty and do not start menstruating.  Let's briefly review the other options:  B. 47XXX (Triple X syndrome): This condition usually does not cause significant physical abnormalities and is often associated with a mild or no clinical presentation.  C. 47XXY (Klinefelter syndrome): This condition affects males and is characterized by an extra X chromosome (XXY). The clinical presentation includes tall stature, gynecomastia (breast enlargement), and small testes. It is not associated with webbed neck or widely spaced nipples, and the affected individuals are males, not females.  D. Trisomy 13 (Patau syndrome) and E. Trisomy 21 (Down syndrome): These chromosomal abnormalities are not associated with the specific clinical features described in the case. Trisomy 13 (Patau syndrome) is associated with severe intellectual disability, multiple congenital abnormalities, and a high mortality rate in infancy. Trisomy 21 (Down syndrome) is characterized by intellectual disability, distinct facial features, and various medical issues, but it does not typically present with the described findings of webbed neck and widely spaced nipples.  It's important to note that a definitive diagnosis of Turner syndrome requires chromosomal analysis, and a qualified healthcare professional should be consulted for proper evaluation and management of the patient's condition. |
|  |  | Attempt 3  The most likely chromosomal abnormality that could cause the clinical presentation described in the scenario is A. 45X0, also known as Turner syndrome.  Turner syndrome is characterized by the absence or partial absence of one X chromosome in females. Common features of Turner syndrome include short stature, delayed or absent puberty, primary amenorrhea (lack of menstrual periods), and certain physical characteristics such as a webbed neck, widely spaced nipples, and lymphedema (swelling of hands and feet). The presence of these features in the described patient, along with delayed puberty and short stature, strongly suggests Turner syndrome as the underlying cause.  Option B (47XXX) and option C (47XXY) are not associated with the specific findings mentioned in the scenario. 47XXX (Triple X syndrome) typically does not cause significant physical abnormalities or delayed puberty, while 47XXY (Klinefelter syndrome) is associated with tall stature and may present with delayed puberty but does not usually exhibit the physical features described.  Options D (Trisomy 13, also known as Patau syndrome) and E (Trisomy 21, also known as Down syndrome) are associated with distinct clinical features but are not typically associated with delayed puberty or the specific physical findings described in the scenario.  It's important to note that a definitive diagnosis of any chromosomal abnormality should be made through appropriate genetic testing and evaluation by a healthcare professional specializing in genetics or pediatric endocrinology. |
| **46** | A 36 year old man is brought to the Emergency Department with a head injury after a fall from 10 metres. He was unconscious for a minute but recovered and seemed fully alert and orientated. Four hours later he becomes drowsy and confused, and then collapses. His GCS is 7/15.  Which is the most likely diagnosis?  A. Cerebral contusions  B. Compound depressed skull fracture  C. Diffuse axonal injury  D. Extradural haematoma  E. Subarachnoid haemorrhage | Attempt 1  Based on the information provided, the most likely diagnosis for the 36-year-old man's clinical presentation is:  D. Extradural hematoma  An extradural hematoma, also known as an epidural hematoma, occurs when there is bleeding between the skull and the outermost layer of the meninges, known as the dura mater. It is typically caused by trauma, such as a fall or a blow to the head. The initial loss of consciousness followed by a lucid interval and subsequent deterioration in neurological status are classic features of an extradural hematoma.  In this case, the patient initially regained consciousness after the fall and appeared fully alert and oriented. However, four hours later, he became drowsy, confused, and ultimately collapsed. This pattern of symptoms suggests the development of a significant brain injury, most likely due to an expanding extradural hematoma. The decreasing level of consciousness indicated by a Glasgow Coma Scale (GCS) score of 7/15 further supports this diagnosis.  The other options listed are less likely based on the information provided:  - Cerebral contusions (Option A) are localized areas of bruising and damage to the brain tissue and would not typically cause a lucid interval followed by a deterioration in neurological status.  - Compound depressed skull fracture (Option B) involves a break in the skull bone with the bone fragment penetrating the brain tissue. While it can cause neurological deficits, it is less likely to present with a lucid interval and subsequent deterioration.  - Diffuse axonal injury (Option C) involves widespread damage to the brain's white matter and can occur due to acceleration-deceleration forces. However, the clinical presentation of a lucid interval followed by deterioration is less characteristic of diffuse axonal injury.  - Subarachnoid hemorrhage (Option E) refers to bleeding into the space between the arachnoid membrane and the pia mater. While it can cause a decrease in consciousness, the lucid interval and subsequent deterioration are not typical features of a subarachnoid hemorrhage.  It's important to note that an urgent evaluation and neuroimaging, such as a CT scan, would be necessary to confirm the diagnosis and guide appropriate management in this emergency situation. |
|  |  | Attempt 2  Based on the clinical presentation and the mechanism of injury, the most likely diagnosis in this case is:  D. Extradural hematoma  An extradural hematoma (also known as epidural hematoma) is a type of traumatic brain injury characterized by bleeding between the inner surface of the skull and the outer layer of the brain (dura mater). It typically occurs due to a rupture of the middle meningeal artery, often as a result of a skull fracture after a head injury, such as a fall or a blow to the head.  The clinical course described in the scenario is consistent with an extradural hematoma. Initially, the patient appeared fully alert and oriented after the fall, but then deteriorated hours later, becoming drowsy, confused, and eventually collapsing. The decrease in the Glasgow Coma Scale (GCS) score (from 15 to 7/15) indicates a significant neurological deterioration.  Let's briefly review the other options:  A. Cerebral contusions: Cerebral contusions are bruised areas of the brain tissue that can occur after a head injury. While they may lead to neurological symptoms, they are less likely to cause a rapid decline in consciousness as seen in this case.  B. Compound depressed skull fracture: A compound depressed skull fracture is an open fracture where the bone is fragmented and displaced into the brain tissue, creating a communication between the outside environment and the intracranial space. This type of fracture can lead to infection and neurological symptoms but is less likely to present with the sudden deterioration described in the scenario.  C. Diffuse axonal injury: Diffuse axonal injury (DAI) results from the shearing forces that cause widespread damage to axons in the brain. While it can cause significant impairment of consciousness, it usually manifests early after the injury and does not explain the initial lucid interval described in the scenario.  E. Subarachnoid hemorrhage: A subarachnoid hemorrhage occurs when there is bleeding into the space between the arachnoid membrane and the pia mater (subarachnoid space). It typically presents with a sudden severe headache (often described as "thunderclap headache") and may lead to neurological deficits, but it is less likely to cause the lucid interval and delayed deterioration described in the case.  In cases of head injury, prompt medical evaluation and imaging (e.g., CT scan) are crucial to identify and manage potentially life-threatening conditions like extradural hematomas. Immediate neurosurgical intervention may be necessary to evacuate the hematoma and relieve pressure on the brain. This scenario highlights the importance of vigilance and close monitoring of patients with head injuries, even if they initially seem alert and oriented. |
|  |  | Attempt 3  The most likely diagnosis in this case is D. Extradural hematoma.  Extradural hematoma (also known as epidural hematoma) is a collection of blood between the skull and the dura mater, the outermost layer of the meninges. It often occurs as a result of trauma, such as a skull fracture, which can cause damage to the blood vessels. Initially, the patient may have a lucid interval after the injury, where they appear alert and oriented. However, as the hematoma expands and puts pressure on the brain, neurological symptoms can develop, including drowsiness, confusion, and a decline in consciousness. This is consistent with the progression described in the scenario.  Cerebral contusions (A) are areas of bruising or bleeding within the brain tissue itself and may cause neurological deficits, but they do not typically present with a lucid interval.  Compound depressed skull fracture (B) refers to a fracture in which the skull bone fragments are displaced inward, causing direct injury to the brain. While this can cause significant neurological symptoms, including loss of consciousness, it does not typically present with a lucid interval.  Diffuse axonal injury (C) refers to widespread damage to the axons in the brain due to shearing forces. It can cause a variety of neurological symptoms but is less likely to present with a lucid interval.  Subarachnoid hemorrhage (E) is bleeding into the subarachnoid space, which surrounds the brain. While it can cause neurological symptoms, it does not typically present with a lucid interval.  Prompt recognition and emergent surgical intervention are crucial in cases of extradural hematoma to prevent further deterioration and potentially fatal outcomes. |
| **47** | N/A |  |
|  |  |  |
|  |  |  |
| **48** | A 42 year old woman has 6 months of a painful right shoulder. Her pain keeps her awake at night and is worsened by movement. She is otherwise well. There is no history of trauma. She has pain on shoulder abduction between 80° and 120°. Her shoulder movements are otherwise normal. There is no joint effusion. X-ray of right shoulder is normal.  Which is the most likely diagnosis?  A. Cervical radiculopathy  B. Osteoarthritis of glenohumeral joint  C. Polymyalgia rheumatica  D. Pseudogout  E. Subacromial bursitis | Attempt 1  Based on the given information, the most likely diagnosis for the 42-year-old woman's shoulder pain is:  E. Subacromial bursitis  Subacromial bursitis is inflammation of the subacromial bursa, which is a fluid-filled sac located beneath the acromion process of the scapula. It commonly presents with shoulder pain that is worse at night and exacerbated by movement. Pain during shoulder abduction between 80° and 120° is known as the "painful arc" and is a characteristic feature of subacromial bursitis. The absence of joint effusion and normal X-ray findings also support this diagnosis.  The other options listed are less likely based on the information provided:  - Cervical radiculopathy (Option A) refers to compression or irritation of a nerve root in the cervical spine. While it can cause shoulder pain, the absence of associated neck pain or specific findings suggestive of cervical radiculopathy makes it less likely.  - Osteoarthritis of the glenohumeral joint (Option B) typically presents with progressive joint pain, stiffness, and limited range of motion. It is less likely in this case as the pain is predominantly during shoulder abduction and there is no mention of other joint involvement or characteristic osteoarthritic changes on X-ray.  - Polymyalgia rheumatica (Option C) is a systemic inflammatory condition primarily affecting older individuals. It commonly presents with bilateral shoulder pain, stiffness, and systemic symptoms. It is less likely in this case due to the patient's age and the absence of other characteristic features.  - Pseudogout (Option D) is a form of arthritis caused by the deposition of calcium pyrophosphate crystals in the joints. While it can cause acute joint pain, the chronic nature of the symptoms and the absence of other joint involvement make it less likely.  It's important to note that a thorough clinical examination and further investigations, such as an ultrasound or MRI, may be required to confirm the diagnosis and guide appropriate management. |
|  |  | Attempt 2  The most likely diagnosis based on the given information is:  E. Subacromial bursitis  Subacromial bursitis is a condition characterized by inflammation of the subacromial bursa, a small fluid-filled sac located beneath the acromion (part of the scapula) in the shoulder joint. It can lead to pain and limited range of motion in the shoulder. The typical symptoms include shoulder pain that worsens with movement, especially during abduction between 80° and 120°, as described in the case. The pain may be severe enough to keep the person awake at night.  Let's briefly review the other options:  A. Cervical radiculopathy: This condition involves compression or irritation of a nerve root in the cervical spine (neck region), leading to pain, numbness, and weakness radiating down the arm. However, cervical radiculopathy would not typically cause isolated shoulder pain without any neck symptoms.  B. Osteoarthritis of glenohumeral joint: Osteoarthritis is the degeneration of joint cartilage, which can lead to pain, stiffness, and reduced range of motion. While it is a possible cause of shoulder pain, the typical location of pain in glenohumeral osteoarthritis is deep within the joint, and it would often show some abnormalities on an X-ray.  C. Polymyalgia rheumatica: Polymyalgia rheumatica is a condition that primarily affects older adults and is characterized by pain and stiffness in the shoulders, neck, and hips. The pain is usually symmetrical and more generalized, and there may be associated systemic symptoms like fatigue, fever, and weight loss.  D. Pseudogout: Pseudogout is a type of arthritis caused by the deposition of calcium pyrophosphate crystals in the joints. It can cause sudden, painful joint swelling and inflammation. While it can affect the shoulder joint, it is less likely to present with the specific pattern of pain on shoulder abduction described in the case.  Based on the information provided, subacromial bursitis is the most likely diagnosis. However, it's essential to note that a definitive diagnosis can only be made by a qualified healthcare professional after a thorough physical examination and appropriate investigations, if needed. |
|  |  | Attempt 3  The most likely diagnosis in this case is E. Subacromial bursitis.  Subacromial bursitis is inflammation of the subacromial bursa, a fluid-filled sac located between the rotator cuff tendons and the acromion process of the scapula. It often presents with pain in the shoulder, particularly with movement, and can be worse at night. Pain on shoulder abduction between 80° and 120°, known as painful arc syndrome, is a common finding in subacromial bursitis. The absence of joint effusion and normal X-ray findings support this diagnosis.  Cervical radiculopathy (A) refers to compression or irritation of a nerve root in the cervical spine, which can cause pain and other symptoms radiating down the arm. However, in this case, the pain is localized to the shoulder without associated neck or arm symptoms.  Osteoarthritis of the glenohumeral joint (B) typically presents with joint pain, stiffness, and limited range of motion. X-ray findings may show joint space narrowing, osteophyte formation, and other signs of degenerative changes. However, the absence of joint effusion and normal X-ray findings make this diagnosis less likely.  Polymyalgia rheumatica (C) is characterized by pain and stiffness, often in the shoulder and hip girdles, particularly in the morning. It is more commonly seen in individuals over 50 years old, and other systemic symptoms may be present. However, the localized nature of the pain in the shoulder and absence of other characteristic features make this diagnosis less likely.  Pseudogout (D) is a form of crystal arthropathy caused by the deposition of calcium pyrophosphate crystals in the joints. It can cause acute episodes of joint inflammation, but it is less likely to present with chronic shoulder pain as described in the scenario.  It's important to note that a comprehensive clinical evaluation, including a thorough history, physical examination, and consideration of other factors, should be conducted to confirm the diagnosis and guide further management. |
| **49** | A 45 year old man has his BMI measured as part of a clinical trial for type 2 diabetes.  Which is the most appropriate classification of data on this parameter?  A. Categorical  B. Continuous  C. Discrete  D. Nominal  E. Subjective | Attempt 1  The most appropriate classification of BMI data in this context would be:  B. Continuous  BMI (Body Mass Index) is a continuous numerical measurement that quantifies the relationship between a person's weight and height. It is calculated by dividing a person's weight in kilograms by the square of their height in meters (BMI = weight/height^2). The resulting value is a continuous variable that can have a wide range of values.  Categorical data (Option A) refers to data that can be grouped into distinct categories or labels. Discrete data (Option C) represents values that are separate and distinct, typically counting data or whole numbers. Nominal data (Option D) refers to data that is categorized into non-ordered groups or labels. Subjective data (Option E) refers to information based on personal opinions or interpretations.  Since BMI is a numerical measurement that can take on any value within a continuous range, it is classified as continuous data. |
|  |  | Attempt 2  The most appropriate classification of data for BMI (Body Mass Index) is:  B. Continuous  BMI is a numerical measurement calculated using an individual's weight and height. It is used to assess body weight relative to height and is commonly used as a screening tool to identify possible weight-related health risks, such as obesity and type 2 diabetes. BMI values can be expressed as decimal numbers with varying precision.  Continuous data is numerical data that can take any value within a certain range and can be further divided into smaller and smaller units. In the case of BMI, it can have a wide range of values, and there are no distinct categories or fixed intervals. It is a continuous variable that can vary from very low values to very high values and can include decimal points.  In contrast, let's briefly review the other options:  A. Categorical: Categorical data consists of distinct categories or groups and does not have a natural numerical value. Examples of categorical data include gender (male or female), blood type (A, B, AB, O), or educational level (high school, college, graduate).  C. Discrete: Discrete data is numerical data with distinct and separate values, usually with no intermediate values between them. Examples of discrete data include the number of children in a family (1, 2, 3, etc.) or the number of patients in different age groups.  D. Nominal: Nominal data is a type of categorical data where the categories are unordered and have no inherent rank or order. Examples of nominal data include eye color (blue, brown, green) or car brands (Toyota, Ford, Honda).  E. Subjective: Subjective data is based on personal opinions, feelings, or interpretations and is not measurable or numerical. It is not applicable in the context of BMI measurement.  In summary, BMI is considered continuous data because it is a numerical measurement that can take any value within a range and can include decimal points. |
|  |  | Attempt 3  The most appropriate classification of BMI data in this scenario would be B. Continuous.  BMI (Body Mass Index) is a numerical measurement calculated using a person's weight and height. It provides an indication of whether an individual's weight is within a healthy range. BMI is a continuous variable because it can take on any value within a specific range (e.g., 18.5 to 24.9 for a healthy weight range). Continuous variables are measured on a continuous scale and can have decimal values or a wide range of possible values.  Categorical data (A) refers to data that can be categorized into distinct groups or categories, such as gender or blood type. Discrete data (C) refers to data that can only take on specific, separate values, such as the number of children in a family (whole numbers). Nominal data (D) is a type of categorical data where the categories have no intrinsic order or hierarchy, such as eye color or marital status. Subjective data (E) refers to information based on personal opinions or judgments, which is not applicable in the case of BMI measurement.  In summary, BMI is a continuous variable as it represents a numerical measurement along a continuous scale, allowing for a wide range of values within a defined range. |
| **50** | A 75 year old man with metastatic bowel cancer is dying. He is semi-conscious. He is being treated with morphine and haloperidol via a syringe driver. His breathing is becoming increasingly rattly.  Which is the most appropriate drug management?  A. Amoxicillin  B. Furosemide  C. Hyoscine  D. Levomepromazine  E. Midazolam | Attempt 1  In the scenario described, the most appropriate drug management for the increasingly rattly breathing of the dying patient would be D. Levomepromazine.  Levomepromazine, also known as methotrimeprazine, is an antipsychotic medication with sedative and antiemetic properties. It is commonly used in palliative care for symptom management, including alleviating distressing respiratory symptoms such as rattly breathing or noisy respiratory secretions (known as death rattle). Levomepromazine helps to reduce excessive respiratory secretions and can provide comfort to the patient in the final stages of life.  Amoxicillin (A) is an antibiotic used to treat bacterial infections and would not be indicated in this situation. Furosemide (B) is a diuretic used to treat fluid retention and would not address the rattly breathing. Hyoscine (C) is an anticholinergic medication that can help reduce respiratory secretions, but levomepromazine is a more appropriate choice in this case. Midazolam (E) is a benzodiazepine used for sedation and anxiety but may not effectively manage the rattly breathing compared to levomepromazine.  It's important to note that the choice of medication and its dosing should be determined by a healthcare professional experienced in palliative care, taking into consideration the patient's specific needs and circumstances. |
|  |  | Attempt 2  In this scenario, the most appropriate drug management to address the increasingly rattly breathing in a dying patient is:  C. Hyoscine (also known as scopolamine)  Hyoscine is an anticholinergic medication that can help reduce excess respiratory secretions and bronchial secretions, commonly referred to as "death rattle." The death rattle is a common symptom in patients nearing the end of life and is caused by the accumulation of secretions in the airways as the ability to clear them diminishes.  Let's briefly review the other options:  A. Amoxicillin: Amoxicillin is an antibiotic, but it would not be appropriate in this situation since it is not intended to address the symptom of death rattle, and it is not indicated in the management of dying patients with advanced cancer.  B. Furosemide: Furosemide is a diuretic used to increase urine output in conditions such as fluid retention or heart failure. It would not address the issue of rattly breathing and may not be appropriate for a dying patient.  D. Levomepromazine: Levomepromazine (methotrimeprazine) is an antipsychotic and antiemetic medication with sedative properties. While it can help manage agitation and anxiety in palliative care patients, it is not typically used to address the symptom of death rattle.  E. Midazolam: Midazolam is a benzodiazepine with sedative and anxiolytic properties. It is commonly used in palliative care to manage anxiety and restlessness in dying patients but may not directly address the issue of rattly breathing.  It's essential to provide comfort and symptom management for patients at the end of life. In this situation, hyoscine can help alleviate the distressing symptom of death rattle, making the patient more comfortable during this difficult time. The choice of medications and their dosages should be made by the healthcare team in consultation with the patient (if possible) and their family, following palliative care guidelines and individual patient needs and preferences. |
|  |  | Attempt 3  In the given scenario, the most appropriate drug management for the patient's rattly breathing would be:  C. Hyoscine (also known as scopolamine)  Hyoscine is an anticholinergic medication that can help reduce excessive respiratory secretions and alleviate the rattling sound during the dying process. It is commonly used in palliative care to provide comfort to patients experiencing respiratory distress.  Amoxicillin (Option A) is an antibiotic and would not address the symptom of rattly breathing. Furosemide (Option B) is a diuretic and would not be appropriate for this situation unless there was a specific indication for its use. Levomepromazine (Option D) and midazolam (Option E) are sedative medications commonly used in palliative care to manage symptoms, but they may not specifically address the rattly breathing. Hyoscine (Option C) is the most appropriate choice among the given options for managing the patient's symptom of rattly breathing. |
| **51** | A 67 year old man has had 24 hours of a painful swollen left knee. He went hill walking 3 days ago. His temperature is 37.6°C, pulse rate 104 bpm and BP 116/80 mmHg. His left knee is red, swollen and hot to touch. He has a reduced range of movement in his knee and is unable to weight bear due to pain. Investigations: Haemoglobin 145 g/L (130–175) White cell count 23.4 × 109/L (4.0–11.0) Platelets 546 × 109/L (150–400) Neutrophils 19.2 × 109/L (2.0–7.5) CRP 122 mg/L (<5)  Which is the most appropriate next investigation?  A. Aspiration of left knee joint for microscopy and culture  B. MR imaging of left knee  C. Serum anti-cyclic citrullinated peptide antibody  D. Serum uric acid  E. X-ray of left knee | Attempt 1  Given the clinical presentation of a painful swollen knee with systemic signs of inflammation, the most appropriate next investigation would be:  A. Aspiration of left knee joint for microscopy and culture  Aspiration of the affected joint allows for the evaluation of synovial fluid, including analysis for cell count, Gram stain, culture, and crystal examination. This can help differentiate between septic arthritis, which may require urgent intervention with antibiotics, and other causes such as crystal-induced arthritis or inflammatory conditions.  While other investigations may be relevant in certain contexts, such as MR imaging (Option B) for assessing soft tissue structures or X-ray (Option E) to evaluate bony abnormalities, in this case, the priority is to rule out septic arthritis and guide appropriate management. Thus, aspiration of the left knee joint for microscopy and culture (Option A) is the most appropriate next step. |
|  |  | Attempt 2  Based on the clinical presentation and investigation findings, the most appropriate next investigation in this case is:  A. Aspiration of left knee joint for microscopy and culture  The patient's symptoms of a red, swollen, and hot left knee, along with the elevated white cell count, neutrophils, and CRP, are suggestive of an acute inflammatory process in the joint. The presence of a reduced range of movement and inability to weight bear due to pain further indicates a significant joint involvement.  Aspiration of the left knee joint is crucial to determine the underlying cause of the joint inflammation. It involves removing a sample of synovial fluid from the joint for examination under a microscope and culture to identify the possible infectious organism. This procedure helps in distinguishing between different causes of joint inflammation, such as septic arthritis (bacterial infection of the joint) or crystal-induced arthritis (e.g., gout or pseudogout).  Let's briefly review the other options:  B. MR imaging of the left knee: MRI (Magnetic Resonance Imaging) can be valuable for visualizing soft tissues, including ligaments, tendons, and menisci in the knee. However, aspiration of the joint is more appropriate as the next step to determine the cause of the acute inflammatory process.  C. Serum anti-cyclic citrullinated peptide antibody: This test is used in the diagnosis of rheumatoid arthritis (RA), an autoimmune condition. However, the clinical presentation in this case is more suggestive of an acute inflammatory process rather than RA, and joint aspiration is a more immediate and specific investigation.  D. Serum uric acid: This test is helpful in diagnosing gout, a form of arthritis caused by the deposition of uric acid crystals in the joints. However, the elevated white cell count, neutrophils, and CRP, along with the acute presentation, make joint aspiration a more appropriate next step.  E. X-ray of the left knee: X-rays can be useful in evaluating the joint for structural abnormalities and bony changes. However, in the acute phase of joint inflammation, aspiration of the joint for microscopy and culture takes precedence to guide immediate management.  Aspiration of the left knee joint for microscopy and culture will aid in the accurate diagnosis and guide appropriate treatment for the patient's acute knee inflammation. |
|  |  | Attempt 3  Based on the clinical presentation and findings, the most appropriate next investigation in this case would be A. Aspiration of the left knee joint for microscopy and culture.  The patient's symptoms, including a painful, swollen, red, and hot knee, along with reduced range of movement and inability to weight bear, are suggestive of a septic arthritis. The elevated white cell count, neutrophilia, and significantly elevated CRP further support this possibility. Septic arthritis is an urgent medical condition that requires prompt diagnosis and treatment to prevent joint damage and systemic complications.  Aspiration of the affected joint allows for the collection of synovial fluid, which can be analyzed for microscopy (including Gram stain) and culture. This investigation helps confirm the diagnosis of septic arthritis by identifying the presence of bacteria or other infectious agents in the joint fluid. It also helps guide appropriate antibiotic therapy.  While other investigations such as MR imaging (B) or X-ray (E) of the left knee may be useful in certain situations, in this case, the urgency and need for immediate management make joint aspiration the most appropriate next step. Serum anti-cyclic citrullinated peptide antibody (C) is more specific for rheumatoid arthritis and would not be the primary investigation at this stage. Serum uric acid (D) is relevant for the diagnosis of gout but is not the most appropriate next investigation given the clinical presentation of a septic joint. |
| **52** | A 36 year old woman and her partner have been trying to conceive for the last 18 months. Neither has ever attained a pregnancy before. Both are medically fit and well and have no surgical history. Her periods are every 38-45 days. Both smoke 5-10 cigarettes per day and drink occasionally at the weekend. Examination is unremarkable in both.  Which is the most likely cause of their inability to conceive?  A. Abnormal semen  B. Anovulatory cycles  C. Parental smoking  D. Unexplained subfertility  E. Uterine tube blockage | Attempt 1  In the given scenario, the most likely cause of the couple's inability to conceive would be:  B. Anovulatory cycles  The woman's menstrual cycles of 38-45 days suggest irregularity, which can be indicative of anovulation (lack of ovulation) or irregular ovulation. Anovulatory cycles significantly decrease the chances of conception as there is no egg available for fertilization.  While other factors such as abnormal semen (Option A), parental smoking (Option C), unexplained subfertility (Option D), or uterine tube blockage (Option E) can contribute to difficulties in conceiving, the irregular menstrual cycles in the woman strongly suggest anovulation as the primary cause. It is important to note that a comprehensive evaluation of both partners should be conducted to identify any additional factors that may be affecting fertility. |
|  |  | Attempt 2  In this case, the most likely cause of the couple's inability to conceive is:  B. Anovulatory cycles  Anovulatory cycles occur when a woman's ovaries do not release an egg (ovulate) during her menstrual cycle. Given that the woman's menstrual cycles are irregular, occurring every 38-45 days, it is possible that she is experiencing anovulatory cycles. Irregular menstrual cycles can be a sign of hormonal imbalances or irregularities in the ovulation process, which can significantly affect a couple's ability to conceive.  Let's briefly review the other options:  A. Abnormal semen: While male factor infertility due to abnormal semen can contribute to difficulty conceiving, it is important to note that both partners in this case have never attained a pregnancy before. Therefore, the likelihood of abnormal semen being the sole cause is lower.  C. Parental smoking: Smoking can have adverse effects on fertility in both men and women. However, since both partners smoke, it is less likely to be the primary cause of their inability to conceive, especially when both are otherwise medically fit and well.  D. Unexplained subfertility: "Unexplained subfertility" is a diagnosis used when standard fertility investigations do not reveal a specific cause for a couple's infertility. While it is a possibility, it is usually considered after a thorough evaluation, including assessing ovulation, semen analysis, and checking for uterine tube blockage.  E. Uterine tube blockage: Blockage or damage in the fallopian tubes can prevent the sperm and egg from meeting, leading to infertility. However, without additional information, it is difficult to determine if this is the primary cause in this specific case.  Given the information provided, the most likely cause of the couple's inability to conceive is anovulatory cycles in the woman. Irregular menstrual cycles can make it challenging to time intercourse accurately during the fertile window, leading to difficulty conceiving. It is essential for the couple to seek medical evaluation and assistance from a fertility specialist to identify the specific cause of their subfertility and explore appropriate treatment options. |
|  |  | Attempt 3  In the given scenario, the most likely cause of the couple's inability to conceive is B. Anovulatory cycles.  The woman's menstrual cycles of 38-45 days suggest a longer-than-normal cycle length. Normal menstrual cycles typically occur every 21-35 days. Anovulatory cycles occur when ovulation does not take place, leading to the absence of fertile periods. In such cases, pregnancy cannot occur as there is no egg available for fertilization.  Factors such as smoking, occasional alcohol consumption, and no previous pregnancies may contribute to fertility issues, but in this case, the irregular and prolonged menstrual cycles strongly suggest anovulation as the primary cause of infertility.  To confirm the diagnosis of anovulatory cycles, further investigations such as assessing hormonal levels (e.g., follicle-stimulating hormone, luteinizing hormone, progesterone) at specific times during the menstrual cycle may be necessary. Treatment options for anovulation include lifestyle modifications, medications to induce ovulation, and assisted reproductive techniques, depending on the specific underlying cause.  It's important for the couple to consult with a healthcare professional or fertility specialist to undergo a comprehensive evaluation and receive appropriate guidance and treatment tailored to their specific situation. |
| **53** | A 45 year old man has a lump in the right side of his neck. He has never smoked and takes no regular medications. Fine needle aspiration of the mass reveals squamous cell carcinoma, and subsequent investigations identify the primary tumour in the right tonsil.  Which virus is associated with this malignancy?  A. Cytomegalovirus  B. Epstein–Barr virus  C. Human immunodeficiency virus  D. Human papilloma virus  E. Human T-lymphotropic virus | Attempt 1  The virus associated with squamous cell carcinoma of the tonsil in this case is:  D. Human papillomavirus (HPV)  HPV infection, particularly with high-risk strains such as HPV-16 and HPV-18, is strongly associated with the development of squamous cell carcinoma in the oropharynx, including the tonsils. HPV-related oropharyngeal cancers are becoming increasingly common, especially among non-smokers and non-drinkers. It is important to note that not all cases of tonsil cancer are caused by HPV, but in this particular scenario, HPV is the most likely virus associated with the malignancy. |
|  |  | Attempt 2  The virus associated with squamous cell carcinoma of the tonsil, as described in the case, is:  D. Human papillomavirus (HPV)  HPV is a known risk factor for the development of squamous cell carcinoma of the tonsil and other head and neck cancers. In recent years, there has been a significant increase in the incidence of HPV-related head and neck cancers, particularly in non-smoking and non-drinking individuals.  The presence of HPV in the tumor can be determined through molecular testing or immunohistochemistry. HPV-associated tumors generally have a better prognosis compared to non-HPV-related head and neck cancers.  Let's briefly review the other options:  A. Cytomegalovirus (CMV): CMV is a herpesvirus that can cause various infections, but it is not associated with squamous cell carcinoma of the tonsil.  B. Epstein–Barr virus (EBV): EBV is also a herpesvirus and is associated with certain types of cancers, such as Burkitt's lymphoma and nasopharyngeal carcinoma. However, it is not specifically linked to squamous cell carcinoma of the tonsil.  C. Human immunodeficiency virus (HIV): HIV is a virus that weakens the immune system and can increase the risk of certain cancers, including lymphomas and Kaposi sarcoma. It is not directly associated with squamous cell carcinoma of the tonsil.  E. Human T-lymphotropic virus (HTLV): HTLV is associated with certain types of T-cell lymphomas and is not linked to squamous cell carcinoma of the tonsil.  In this case, the most likely virus associated with the malignancy is HPV. It is essential for the patient to undergo further evaluation and management by an oncologist or a head and neck cancer specialist to determine the extent of the disease and plan appropriate treatment. |
|  |  | Attempt 3  The virus associated with squamous cell carcinoma of the tonsil in this case is D. Human papillomavirus (HPV).  HPV has been identified as a significant risk factor for oropharyngeal squamous cell carcinoma, particularly in non-smokers and non-drinkers. The majority of HPV-associated oropharyngeal cancers are caused by high-risk types of HPV, with HPV-16 being the most common. HPV-related cancers often arise in the tonsils and base of the tongue.  The presence of squamous cell carcinoma in the neck lymph nodes with a primary tumor in the tonsil suggests that the cancer has spread from the primary site to the regional lymph nodes.  It's worth noting that other risk factors, such as tobacco and alcohol use, may contribute to the development of oropharyngeal cancers, but in this scenario, the absence of smoking history and the presence of HPV-positive tumor cells point towards HPV as the primary viral association. |
| **54** | A 78 year old man has worsening breathlessness. He has heart failure that has been worsening progressively over the past 12 months. He has been bed bound for the past 2 weeks. He was recently found to have probable lung carcinoma but was not fit for further investigation. He is dyspnoeic, cyanosed and confused. His temperature is 36.1°C, pulse rate 100 bpm, BP 92/60 mmHg, respiratory rate 30 breaths per minute and oxygen saturation 88% on 15 L/min via a Venturi mask. He has inspiratory crackles in both bases. He is treated with intravenous furosemide.  Which additional treatment is most likely to reduce his breathlessness?  A. Intramuscular haloperidol  B. Intravenous glyceryl trinitrate  C. Intravenous morphine  D. Nasal intermittent positive pressure ventilation  E. Nebulised salbutamol | Attempt 1  In the given scenario, the most appropriate additional treatment to reduce the patient's breathlessness is:  C. Intravenous morphine  Intravenous morphine is a commonly used opioid medication for the management of severe dyspnea (shortness of breath) in palliative care, including in patients with advanced heart failure. Morphine helps alleviate breathlessness by reducing the perception of breathlessness and inducing a sense of calm and relaxation. It acts as a respiratory depressant and reduces the work of breathing. By relieving the distressing symptoms associated with breathlessness, it can improve the patient's overall comfort and quality of life. |
|  |  | Attempt 2  In this scenario, the most likely treatment to reduce the patient's breathlessness is:  C. Intravenous morphine  The patient's symptoms of worsening breathlessness, cyanosis, and inspiratory crackles in both lung bases indicate severe respiratory distress, likely caused by heart failure exacerbation and possible lung carcinoma. Intravenous morphine is commonly used in palliative care for the management of dyspnea (breathlessness) in patients with advanced heart failure and other life-limiting conditions.  Morphine helps alleviate dyspnea by reducing the sensation of breathlessness and anxiety associated with severe respiratory distress. It acts as a potent respiratory depressant and helps decrease the patient's respiratory rate and effort. Additionally, morphine has a vasodilatory effect, which can be beneficial in heart failure patients by reducing preload and afterload on the heart.  Options A, B, D, and E are not the most appropriate treatments for reducing breathlessness in this particular scenario:  A. Intramuscular haloperidol: Haloperidol is an antipsychotic medication and is not the primary treatment for reducing breathlessness in this context.  B. Intravenous glyceryl trinitrate: Glyceryl trinitrate (nitroglycerin) is a vasodilator commonly used to treat angina and acute heart failure. While it may be beneficial in certain cases of acute heart failure, it is less likely to be the most appropriate treatment in this specific situation of severe dyspnea.  D. Nasal intermittent positive pressure ventilation (NIPPV): NIPPV is a non-invasive ventilation strategy used in certain cases of acute respiratory failure. However, it may not be feasible or appropriate in a patient with severe confusion and altered mental status.  E. Nebulized salbutamol: Nebulized salbutamol (albuterol) is a bronchodilator used to treat acute bronchoconstriction in conditions such as asthma and chronic obstructive pulmonary disease (COPD). While it may provide relief in those specific conditions, it is less likely to be effective in reducing dyspnea caused by heart failure and lung carcinoma.  In this palliative care setting, the main focus is on providing symptom relief and comfort to the patient. Intravenous morphine, under appropriate medical supervision, can help alleviate the patient's breathlessness and improve his quality of life during this difficult time. The treatment plan should be individualized and coordinated with a palliative care team to address the patient's specific needs and goals of care. |
|  |  | Attempt 3  In this scenario, the most appropriate additional treatment to reduce the patient's breathlessness is C. Intravenous morphine.  The patient's presentation suggests that he is experiencing severe dyspnea, likely due to a combination of his worsening heart failure and probable lung carcinoma. Morphine, as an opioid analgesic, has both analgesic and sedative effects, and it can also help alleviate the sensation of breathlessness in patients with advanced heart failure or cancer-related dyspnea.  Morphine acts as a respiratory depressant, reducing the respiratory drive and relieving the distress associated with breathlessness. By decreasing the patient's respiratory effort, it can help improve oxygenation and decrease the work of breathing.  It's important to note that the choice of treatment should be individualized based on the patient's overall condition, preferences, and potential contraindications. Close monitoring and titration of the morphine dose are necessary to achieve optimal relief of breathlessness while minimizing potential side effects. |
| **55** | An 18 year old woman has a sudden sharp stabbing pain in her abdomen, lasting only a few minutes. It is 14 days since her last period started. She indicates that the pain is localised to the left iliac fossa.  A surge in which hormone is most likely to coincide with her pain?  A. Follicle stimulating hormone  B. Luteinising hormone  C. Oestrogen  D. Progesterone  E. Prolactin | Attempt 1  A surge in hormone B. Luteinising hormone (LH) is most likely to coincide with the pain described by the 18-year-old woman.  The sudden sharp stabbing pain occurring 14 days after her last period and localized to the left iliac fossa suggests the possibility of Mittelschmerz, which is a common condition characterized by pain associated with ovulation.  During the menstrual cycle, there is a surge in luteinising hormone (LH) levels that triggers ovulation. Ovulation is the release of an egg from the ovary, and it typically occurs around the middle of the menstrual cycle. The pain experienced during Mittelschmerz is thought to be due to the stretching or rupture of the ovarian follicle during ovulation.  Therefore, the surge in luteinising hormone (LH) coincides with ovulation and is most likely to be associated with the sharp stabbing pain described by the woman. |
|  |  | Attempt 2  The sudden sharp stabbing pain in the left iliac fossa, occurring 14 days after her last period started, suggests a possible cause related to ovulation. During the menstrual cycle, ovulation occurs around the middle of the cycle, and a surge in a specific hormone coincides with this event.  The hormone most likely to coincide with her pain is:  B. Luteinising hormone (LH)  Luteinising hormone (LH) is responsible for triggering ovulation, which is the release of the mature egg from the ovarian follicle. Around the time of ovulation, there is a significant surge in LH levels, leading to the release of the egg from the ovary. This surge in LH can cause slight stretching and irritation of the ovarian tissue, leading to the sudden, sharp, and localized pain in the abdomen, which is often referred to as "mittelschmerz" (German for "middle pain").  The pain associated with mittelschmerz is usually brief, lasting only a few minutes to a few hours, and is typically not a cause for concern. It occurs on one side of the lower abdomen, depending on which ovary releases the egg during that particular cycle.  Options A, C, D, and E are not associated with the specific event of ovulation:  A. Follicle stimulating hormone (FSH): FSH is responsible for stimulating the growth and development of ovarian follicles, but it does not have a significant surge around the time of ovulation.  C. Oestrogen: Oestrogen plays a crucial role in the menstrual cycle and helps prepare the endometrium for potential embryo implantation. While oestrogen levels fluctuate during the menstrual cycle, they do not surge at the time of ovulation.  D. Progesterone: Progesterone levels increase during the second half of the menstrual cycle, after ovulation has occurred, to prepare the uterus for possible pregnancy. Progesterone does not surge during ovulation.  E. Prolactin: Prolactin is primarily involved in lactation and milk production and is not directly related to ovulation.  If the pain is mild and resolves quickly, without any other concerning symptoms, it is generally considered a normal part of the menstrual cycle. However, if the pain is severe, persistent, or accompanied by other worrisome symptoms, it is essential to consult with a healthcare professional for further evaluation and to rule out other potential causes of abdominal pain. |
|  |  | Attempt 3  A surge in hormone B. Luteinising hormone (LH) is most likely to coincide with the pain experienced by the 18-year-old woman.  The sudden sharp stabbing pain localized to the left iliac fossa, occurring 14 days after her last period started, suggests a possible case of mittelschmerz. Mittelschmerz is a German term meaning "middle pain" and refers to the abdominal pain that some women experience during ovulation, typically around the midpoint of their menstrual cycle.  During the menstrual cycle, LH levels surge just before ovulation, triggering the release of the mature egg from the ovary. This surge in LH, which is part of the natural hormone fluctuations during the menstrual cycle, can cause mild discomfort or pain in the lower abdomen, known as mittelschmerz.  Therefore, in this case, the surge in luteinising hormone (LH) would coincide with the sharp stabbing pain experienced by the woman. |
| **56** | A 72 year old man with lung cancer is admitted to the respiratory ward with recurrent haemoptysis. He is known to the palliative care team and it is felt that he is near the end of his life. He says that he wants to have his terminal care on the respiratory ward because he knows and trusts the nurses. A 'do not attempt resuscitation' form is completed. He subsequently has further haemoptysis and becomes more breathless, so he is treated with an opioid infusion to relieve his dyspnoea. A doctor suggests that the local hospice is better equipped to care for the patient. His son agrees with the doctor, believing that the hospice is "the right place to die".  What should be the most important factor influencing the decision on whether to move him to a hospice?  A. Consultant's view of patient's best interests  B. Hospital bed state  C. Local policy on end of life care  D. Patient's previous opinion  E. Son's wishes | Attempt 1  The most important factor influencing the decision on whether to move the patient to a hospice should be option D: Patient's previous opinion.  In this situation, the patient has expressed his preference to receive terminal care on the respiratory ward because he knows and trusts the nurses there. Respecting the patient's autonomy and honoring his previously expressed wishes should be the primary consideration when making decisions about his end-of-life care.  While other factors such as the consultant's view, hospital bed state, local policy, and the son's wishes can provide additional information and perspectives, they should not override the patient's own preferences and values. Patient-centered care and respect for patient autonomy are essential principles in end-of-life decision-making. |
|  |  | Attempt 2  The most important factor influencing the decision on whether to move the patient to a hospice should be:  D. Patient's previous opinion  In this scenario, the patient has expressed his preference to have his terminal care on the respiratory ward because he knows and trusts the nurses. This is a clear indication of the patient's wishes and should be respected and prioritized in the decision-making process.  Patient autonomy and respect for the patient's wishes are fundamental principles of medical ethics, especially in end-of-life care. If the patient has expressed a clear preference for his care to be provided on the respiratory ward, it is essential to honor his decision as much as possible. His comfort and sense of trust in the care team are critical factors in providing compassionate end-of-life care.  While other factors, such as the consultant's view, hospital bed state, local policy on end-of-life care, and the son's wishes, may be important considerations, the patient's autonomy and previous opinion should be given the highest priority when determining the best location for his terminal care. |
|  |  | Attempt 3  The most important factor influencing the decision on whether to move the patient to a hospice should be D. Patient's previous opinion.  In this scenario, the patient has expressed his preference to receive his terminal care on the respiratory ward because he knows and trusts the nurses there. Patient autonomy and respect for the patient's wishes are fundamental principles of medical ethics. It is important to honor the patient's preferences and provide care in line with his wishes whenever possible.  While other factors such as the consultant's view, hospital bed state, local policy, and the son's wishes may have some influence, they should not override the patient's own expressed desires and autonomy. The patient's previous opinion should be the primary consideration in this situation, as it reflects his personal values and desires regarding his end-of-life care. |
| **57** | A 74 year old woman has had left sided headache and discomfort when chewing food for 3 months. She experiences a sensation of pressure and pain in her jaw, even when talking. She has lost 8 kg in weight over the same time period. Investigations: CRP 45 mg/L (<5)  Which investigation is most likely to establish the diagnosis?  A. CT scan of head  B. Parotid sialogram  C. Serum anti-neutrophil cytoplasmic antibodies  D. Temporal artery biopsy  E. X-ray of temporomandibular joint | Attempt 1  The most appropriate investigation to establish the diagnosis in this case would be option D: Temporal artery biopsy.  The patient's symptoms, including persistent left-sided headache, discomfort when chewing food, and weight loss, raise suspicion for temporal arteritis, also known as giant cell arteritis. This condition is characterized by inflammation of the medium and large arteries, particularly the temporal arteries.  A temporal artery biopsy is the gold standard diagnostic test for temporal arteritis. It involves taking a small sample of the temporal artery for histological examination, which can confirm the presence of inflammatory changes consistent with the disease.  Other investigations listed, such as a CT scan of the head, parotid sialogram, serum anti-neutrophil cytoplasmic antibodies, and X-ray of the temporomandibular joint, are not specific for temporal arteritis and are unlikely to establish the diagnosis.  It's important to note that the final decision on the appropriate investigation should be made by a healthcare professional based on the patient's clinical presentation and individual circumstances. |
|  |  | Attempt 2  The most likely investigation to establish the diagnosis in this case is:  D. Temporal artery biopsy  The patient's symptoms of left-sided headache and discomfort when chewing, along with a sensation of pressure and pain in the jaw, are concerning for possible temporal arteritis, also known as giant cell arteritis (GCA). Temporal arteritis is a type of vasculitis that affects medium and large arteries, particularly the temporal arteries, which supply blood to the head and face.  The significant weight loss and elevated CRP (C-reactive protein) level further raise suspicion for an inflammatory condition like temporal arteritis.  A temporal artery biopsy is the gold standard investigation for diagnosing temporal arteritis. It involves taking a small sample of the temporal artery and examining it under a microscope to look for inflammation and characteristic changes associated with the condition.  Let's briefly review the other options:  A. CT scan of the head: A CT scan of the head may provide valuable information about the structures in the head, but it is not the most appropriate investigation for evaluating temporal arteritis.  B. Parotid sialogram: A parotid sialogram is a radiographic test used to assess the function and structure of the salivary glands. It is not relevant to the symptoms and suspected diagnosis in this case.  C. Serum anti-neutrophil cytoplasmic antibodies: Serum anti-neutrophil cytoplasmic antibodies (ANCA) are markers associated with certain types of vasculitis, such as granulomatosis with polyangiitis (GPA) and microscopic polyangiitis (MPA). However, ANCA testing is not specific for temporal arteritis and would not be the most appropriate investigation in this context.  E. X-ray of the temporomandibular joint: An X-ray of the temporomandibular joint may be helpful in evaluating specific joint-related issues, but it would not establish the diagnosis of temporal arteritis.  Given the patient's age, symptoms, and elevated CRP level, temporal arteritis is a significant concern that requires prompt evaluation and diagnosis. A temporal artery biopsy is essential to confirm the diagnosis and initiate appropriate treatment, which typically involves high-dose corticosteroids to prevent potential complications, including vision loss. |
|  |  | Attempt 3  The most likely investigation to establish the diagnosis in this case is D. Temporal artery biopsy.  The patient's symptoms, including left-sided headache, discomfort when chewing, and weight loss, raise suspicion for temporal arteritis or giant cell arteritis. Temporal artery biopsy is the gold standard diagnostic test for this condition. It involves the removal and examination of a small segment of the temporal artery to look for characteristic inflammatory changes, such as granulomatous inflammation and giant cells.  Other investigations mentioned, such as CT scan of the head, parotid sialogram, serum anti-neutrophil cytoplasmic antibodies (ANCA), and X-ray of the temporomandibular joint, are not typically used to diagnose temporal arteritis. CT scan of the head may be helpful to rule out other causes of headache, but it is not specific for temporal arteritis. Parotid sialogram is used to evaluate salivary gland abnormalities, which are not typically associated with the symptoms described. Serum ANCA testing is more relevant for conditions such as vasculitis, but not specific to temporal arteritis. X-ray of the temporomandibular joint is used to assess the joint itself, which may not provide direct evidence of temporal arteritis.  Given the clinical presentation and elevated CRP level, temporal artery biopsy is the most appropriate investigation to confirm or exclude the diagnosis of temporal arteritis. |
| **58** | A group of 75 men and a group of 75 women performed a standardised exercise test and had their pulse rate measured at the end. The data from the two groups were compared. The data is normally distributed with equal variance.  Which is the most appropriate statistical test to compare these groups?  A. Analysis of variance  B. Friedman's test  C. Mann–Whitney test  D. Paired Student's t-test  E. Unpaired Student's t-test | Attempt 1  The most appropriate statistical test to compare the two groups in this scenario would be option E: Unpaired Student's t-test.  The Unpaired Student's t-test is used to compare the means of two independent groups when the data are normally distributed and have equal variance. In this case, you have two independent groups (75 men and 75 women) who performed the same exercise test, and you want to compare their pulse rates at the end of the test.  The Analysis of Variance (option A) is used to compare the means of three or more groups, so it is not suitable for comparing just two groups in this scenario.  Friedman's test (option B) is used when comparing repeated measurements within the same group, so it is not appropriate for comparing two independent groups.  The Mann-Whitney test (option C) is a non-parametric test used when the data are not normally distributed or when the assumptions for the t-test are not met. However, in this case, the data is described as normally distributed, so the t-test would be more appropriate.  The Paired Student's t-test (option D) is used when comparing two related groups or when each observation in one group is matched to a corresponding observation in the other group. Since the two groups in this scenario are independent, the paired t-test is not suitable.  Therefore, the Unpaired Student's t-test is the most appropriate statistical test to compare the pulse rates of the two groups. |
|  |  | Attempt 2  The most appropriate statistical test to compare the two groups (75 men and 75 women) who performed a standardised exercise test and had their pulse rate measured at the end is:  E. Unpaired Student's t-test  The unpaired Student's t-test, also known as the independent t-test, is used to compare the means of two independent groups. In this case, the two groups (men and women) are independent of each other, and the data is normally distributed with equal variance.  The t-test is commonly used when the following assumptions are met:  1. The data in each group (men and women) are normally distributed.  2. The variances of the two groups are equal (equal variance assumption).  The independent t-test will help determine if there is a significant difference between the mean pulse rates of men and women after the standardized exercise test. It is useful for comparing continuous variables between two distinct groups, and it assesses whether the observed difference in means is statistically significant or due to random variation.  If the assumptions of normality and equal variance are not met, other appropriate non-parametric tests like the Mann-Whitney test or alternative methods for comparing two groups may be considered.  In summary, the Unpaired Student's t-test is the most appropriate statistical test for comparing the means of the two independent groups (men and women) in this scenario, where the data is normally distributed with equal variance. |
|  |  | Attempt 3  The most appropriate statistical test to compare the pulse rate between the two groups (75 men and 75 women) in this scenario would be E. Unpaired Student's t-test.  The unpaired Student's t-test is used to compare the means of two independent groups when the data is normally distributed and the variances are assumed to be equal. In this case, the groups of men and women are independent of each other, and the data is normally distributed with equal variance, which makes the unpaired Student's t-test suitable for comparing the pulse rates between the two groups.  Analysis of variance (A) is used when comparing the means of more than two groups. Friedman's test (B) is a non-parametric test used for repeated measures or matched samples. Mann-Whitney test (C) is a non-parametric test used to compare two independent groups when the data is not normally distributed. Paired Student's t-test (D) is used to compare the means of two related or paired samples.  Since the data is normally distributed with equal variance and the groups are independent, the most appropriate test for comparing the groups in this case is the unpaired Student's t-test. |
| **59** | A 45 year old man has a tremor that has worsened over several weeks. He has a history of schizophrenia and is taking haloperidol. He has bilateral tremor and cog-wheel rigidity in his upper limbs.  Which is the most appropriate treatment to manage his symptoms?  A. Co-beneldopa  B. Entacapone  C. Pramipexole  D. Procyclidine hydrochloride  E. Selegiline hydrochloride | Attempt 1  The most appropriate treatment to manage the symptoms of tremor and cog-wheel rigidity in this case would be option D: Procyclidine hydrochloride.  The presence of cog-wheel rigidity and tremor in the upper limbs suggests the possibility of parkinsonism, which can be a side effect of antipsychotic medications such as haloperidol. Procyclidine hydrochloride is an anticholinergic medication commonly used to manage the extrapyramidal side effects of antipsychotic drugs, including tremor and rigidity.  Co-beneldopa (option A) is a combination medication of levodopa and carbidopa used in the treatment of Parkinson's disease, which may not be the primary cause in this case.  Entacapone (option B) is a medication used as an adjunct therapy in Parkinson's disease to prolong the effect of levodopa, but it is not the first-line treatment for the symptoms described.  Pramipexole (option C) is a dopamine agonist used primarily in the treatment of Parkinson's disease, but its use in this case would depend on the underlying cause of the symptoms.  Selegiline hydrochloride (option E) is a selective monoamine oxidase inhibitor (MAOI) used in the treatment of Parkinson's disease, but its use in this case would also depend on the underlying cause of the symptoms.  Therefore, procyclidine hydrochloride (option D) is the most appropriate treatment in this scenario to manage the symptoms of tremor and cog-wheel rigidity associated with the use of haloperidol. However, it is important for the patient to consult with their healthcare provider for a proper evaluation and individualized treatment plan. |
|  |  | Attempt 2  The most appropriate treatment to manage the symptoms of tremor and cog-wheel rigidity in this 45-year-old man with a history of schizophrenia and taking haloperidol is:  D. Procyclidine hydrochloride  The combination of bilateral tremor and cog-wheel rigidity in the upper limbs is characteristic of parkinsonism, which can be induced by the use of antipsychotic medications, such as haloperidol. Parkinsonism is a movement disorder that resembles Parkinson's disease but is caused by other factors, including certain medications.  Procyclidine hydrochloride is an anticholinergic medication that is used to manage the extrapyramidal side effects, such as tremor and rigidity, caused by antipsychotic drugs like haloperidol. It works by blocking the action of acetylcholine, a neurotransmitter involved in motor control, which helps reduce the symptoms of parkinsonism.  Let's briefly review the other options:  A. Co-beneldopa: Co-beneldopa is a combination medication of levodopa and carbidopa used to treat the motor symptoms of Parkinson's disease. It is not typically used to manage drug-induced parkinsonism, especially in the context of antipsychotic use.  B. Entacapone: Entacapone is an adjunctive medication used in Parkinson's disease to prolong the effect of levodopa. It is not the most appropriate treatment for drug-induced parkinsonism in this case.  C. Pramipexole: Pramipexole is a dopamine agonist used to treat the symptoms of Parkinson's disease. While it may be effective in idiopathic Parkinson's disease, it is not the most appropriate treatment for drug-induced parkinsonism caused by haloperidol.  E. Selegiline hydrochloride: Selegiline is a selective monoamine oxidase B (MAO-B) inhibitor used in the treatment of Parkinson's disease. Like the other options, it is not the most appropriate treatment for drug-induced parkinsonism caused by haloperidol.  Given the patient's presentation of parkinsonism induced by haloperidol, procyclidine hydrochloride (an anticholinergic medication) is the most suitable choice to manage his symptoms of tremor and cog-wheel rigidity. However, it's essential for the patient's healthcare provider to make the final decision and adjust the treatment plan based on individual patient characteristics and response to the medication. |
|  |  | Attempt 3  The most appropriate treatment to manage the symptoms of a 45-year-old man with bilateral tremor and cog-wheel rigidity in his upper limbs, who has a history of schizophrenia and is taking haloperidol, would be D. Procyclidine hydrochloride.  The presence of cog-wheel rigidity and tremor suggests parkinsonism, which can be a side effect of antipsychotic medications like haloperidol. Procyclidine hydrochloride is an anticholinergic medication commonly used to manage extrapyramidal side effects associated with antipsychotics, including tremor and rigidity.  Co-beneldopa (A), entacapone (B), and pramipexole (C) are medications primarily used in the management of Parkinson's disease and are not typically used to address drug-induced parkinsonism. Selegiline hydrochloride (E) is also used in the treatment of Parkinson's disease but is not the most appropriate option in this case.  It is important for the patient to consult with their healthcare provider for a proper evaluation and management plan. |
| **60** | A 30 year old man is unable to straighten his right middle finger after an injury. He has a flexion deformity of the finger at the distal interphalangeal joint and is unable to actively extend his finger.  Which is the most appropriate management option?  A. External finger fixation  B. Finger splint  C. Internal finger fixation  D. Reassurance  E. Steroid injection | Attempt 1  The most appropriate management option for a 30-year-old man with a flexion deformity of the right middle finger at the distal interphalangeal (DIP) joint and inability to actively extend the finger would be option C: Internal finger fixation.  Based on the description provided, it appears that the patient may have a mallet finger deformity, which is commonly caused by an extensor tendon injury at the DIP joint. In this condition, the terminal tendon is disrupted, leading to the inability to actively extend the finger and resulting in a flexion deformity.  Internal finger fixation, such as the use of a splint or pinning, is the recommended treatment for mallet finger injuries. This helps to maintain the finger in extension, allowing the disrupted tendon to heal. Splinting is usually the first-line treatment for non-displaced or minimally displaced mallet finger injuries, while internal fixation may be considered for more severe cases or when conservative measures have failed.  External finger fixation (option A) is not typically used for mallet finger injuries, as the primary goal is to stabilize the DIP joint in extension.  Finger splinting (option B) is a valid management option for mallet finger, especially in cases of non-displaced or minimally displaced injuries.  Reassurance (option D) alone would not be sufficient as it does not address the underlying tendon injury and deformity.  Steroid injection (option E) is not indicated for the treatment of mallet finger deformity.  It is important for the patient to consult with a healthcare professional, such as a hand specialist or orthopedic surgeon, for a proper evaluation and to determine the most appropriate management option based on the severity and characteristics of the injury. |
|  |  | Attempt 2  The most appropriate management option for a 30-year-old man with a flexion deformity of the right middle finger at the distal interphalangeal joint and inability to actively extend his finger after an injury is:  B. Finger splint  A finger splint is the most appropriate initial management option for a flexion deformity at the distal interphalangeal joint, commonly referred to as a mallet finger. A mallet finger injury occurs when the extensor tendon of the finger is damaged or avulsed, leading to the inability to actively extend the finger.  A finger splint, known as a mallet splint or an extension splint, helps to keep the finger in a straight position, allowing the damaged extensor tendon to heal properly. It is usually worn continuously for several weeks to support the finger and facilitate healing.  The other options listed are not the most appropriate initial management for a mallet finger injury:  A. External finger fixation: External finger fixation is not typically used for managing mallet finger injuries.  C. Internal finger fixation: Internal finger fixation, such as surgical pinning or wiring, is usually reserved for severe cases of mallet finger injuries that have failed to respond to conservative management.  D. Reassurance: Reassurance alone is not sufficient for managing a mallet finger injury, as it requires proper stabilization and support for healing.  E. Steroid injection: Steroid injections are not indicated for managing mallet finger injuries. They may be used for other conditions, but they are not appropriate in this case.  It's important to note that the appropriate management may vary based on the severity of the injury and the specific clinical presentation. Therefore, it is essential for the patient to seek a proper evaluation and treatment plan from a healthcare professional, such as an orthopedic specialist or a hand surgeon, to ensure the best outcome for the finger injury. |
|  |  | Attempt 3  The most appropriate management option for a 30-year-old man with a flexion deformity of the right middle finger at the distal interphalangeal joint, who is unable to actively extend his finger, would be C. Internal finger fixation.  The presentation suggests a condition known as "mallet finger" or "baseball finger," which typically occurs due to an injury to the extensor tendon at the distal interphalangeal joint. This leads to an inability to actively extend the finger and results in a flexion deformity.  Internal finger fixation, such as a splint or pinning, is often required to stabilize the distal interphalangeal joint in the extended position, allowing the tendon to heal and facilitating the recovery of finger extension. Conservative management with external finger fixation (A), finger splint (B), or steroid injection (E) is generally not sufficient for addressing the underlying tendon injury and correcting the deformity. Reassurance (D) alone would not be appropriate given the functional impairment and the need for intervention.  It is important for the patient to consult with a healthcare professional, such as a hand surgeon, for a proper evaluation and to determine the most suitable treatment approach based on the specific details of the injury. |
| **61** | A previously well 20 year old man has had 2 months of night sweats and 5 kg weight loss. His temperature is 37.6°C. He has palpable neck lymph nodes and splenomegaly. Investigations: CT scan chest, abdomen and pelvis: enlarged mediatinal lymph nodes and splenomegaly Lymph node biopsy: Hodgkin/Reed-Sternberg (HRS) cells admixed with a polymorphous inflammatory infiltrate  Which is the most appropriate first line treatment?  A. Chemotherapy  B. Corticosteroids  C. Immunotherapy  D. Radiotherapy  E. Surgical excision of mediastinal lymph nodes | Attempt 1  The most appropriate first-line treatment for a previously well 20-year-old man with enlarged mediastinal lymph nodes, splenomegaly, and a lymph node biopsy showing Hodgkin/Reed-Sternberg (HRS) cells admixed with a polymorphous inflammatory infiltrate would be option A: Chemotherapy.  The clinical presentation and biopsy findings are consistent with Hodgkin lymphoma, a type of lymphoma characterized by the presence of HRS cells. The standard treatment for Hodgkin lymphoma involves a combination of chemotherapy and, in some cases, additional modalities such as radiation therapy.  Chemotherapy is the cornerstone of treatment for Hodgkin lymphoma and is typically initiated in all patients with the disease. The specific chemotherapy regimen used depends on various factors, including the stage and subtype of Hodgkin lymphoma. Commonly used chemotherapy regimens include ABVD (doxorubicin, bleomycin, vinblastine, dacarbazine) and BEACOPP (bleomycin, etoposide, doxorubicin, cyclophosphamide, vincristine, procarbazine, prednisone).  Corticosteroids (option B) may be used as part of the chemotherapy regimen, but they are not considered first-line treatment on their own.  Immunotherapy (option C), such as monoclonal antibodies targeting CD30 (e.g., brentuximab vedotin) or programmed death-1 (PD-1) inhibitors (e.g., nivolumab, pembrolizumab), can be considered in certain cases of relapsed or refractory Hodgkin lymphoma, but it is not the primary treatment option for newly diagnosed cases.  Radiotherapy (option D) may be used in combination with chemotherapy, particularly for localized disease or residual masses after chemotherapy, but it is not typically the first-line treatment.  Surgical excision of mediastinal lymph nodes (option E) is not the standard approach for the treatment of Hodgkin lymphoma. Lymph node biopsy is necessary for diagnosis, but complete surgical excision of involved lymph nodes is not considered curative for this type of lymphoma.  It is essential for the patient to be evaluated and managed by a hematologist or oncologist who specializes in the treatment of lymphomas to determine the most appropriate treatment plan based on the specific characteristics of the disease and individual patient factors. |
|  |  | Attempt 2  The most appropriate first-line treatment for Hodgkin lymphoma, based on the provided information, would be:  A. Chemotherapy  Hodgkin lymphoma is a cancer of the lymphatic system, and the presence of Hodgkin/Reed-Sternberg (HRS) cells confirms the diagnosis. Chemotherapy is the mainstay of treatment for Hodgkin lymphoma, and it is used to target and kill cancer cells throughout the body, including those in the lymph nodes, spleen, and other affected areas.  Radiation therapy (option D) is also commonly used in the treatment of Hodgkin lymphoma, but it is often reserved for specific cases or as an adjunct to chemotherapy. Surgical excision of mediastinal lymph nodes (option E) is generally not the first-line treatment for Hodgkin lymphoma because it is a systemic disease that requires more comprehensive therapy.  Corticosteroids (option B) may be used as part of the treatment regimen, especially for symptom relief and reducing inflammation, but they are not typically used as the primary treatment for Hodgkin lymphoma.  Immunotherapy (option C) has shown promise in the treatment of certain types of lymphomas, but as of my last knowledge update in September 2021, it was not the first-line treatment for Hodgkin lymphoma. It is essential to consult with an oncologist to explore all available treatment options and tailor the treatment plan to the individual patient's specific condition and needs. |
|  |  | Attempt 3  The most appropriate first-line treatment for a 20-year-old man with the clinical presentation and biopsy findings suggestive of Hodgkin lymphoma would be A. Chemotherapy.  Hodgkin lymphoma is a type of lymphoma characterized by the presence of Hodgkin/Reed-Sternberg (HRS) cells in the lymph nodes. The standard treatment for Hodgkin lymphoma typically involves multi-agent chemotherapy, often combined with additional modalities such as radiotherapy or immunotherapy, depending on the stage and characteristics of the disease.  Chemotherapy is the cornerstone of treatment for Hodgkin lymphoma, and it aims to eradicate the malignant cells throughout the body. Various chemotherapy regimens, such as ABVD (doxorubicin, bleomycin, vinblastine, and dacarbazine), are commonly used as first-line treatment for Hodgkin lymphoma.  Corticosteroids (B) may be used as part of the treatment regimen to manage specific symptoms or as supportive care, but they are not considered first-line therapy for eradicating the underlying lymphoma.  Immunotherapy (C) has shown promise in the treatment of Hodgkin lymphoma, particularly with the use of immune checkpoint inhibitors, but it is typically reserved for specific cases or as salvage therapy in relapsed or refractory disease.  Radiotherapy (D) and surgical excision of mediastinal lymph nodes (E) may have a role in localized disease or as adjunctive treatments, but their use as first-line therapy would depend on the stage and extent of the disease.  It is essential for the patient to consult with an oncologist or hematologist for further evaluation and to establish an individualized treatment plan based on the specific characteristics of the disease and the patient's overall health. |
| **62** | A 72 year old man is admitted with cough, breathlessness and confusion. He has metastatic adenocarcinoma and is being treated with chemotherapy. His wife reports that he has had enough of his treatment and planned to discontinue active treatment. He is drowsy and unable to answer questions. He has bronchial breathing at the left base. He is given 0.9% sodium chloride and broad spectrum antibiotics intravenously.  Which is the most appropriate way to determine his cardiopulmonary resuscitation status on this admission?  A. Admitting team should decide  B. Ask an independent advocate to decide  C. Ask consultant oncologist to decide  D. Ask his wife to decide  E. Wait 24 hours to see if patient regains capacity to decide | Attempt 1  In determining the cardiopulmonary resuscitation (CPR) status of a patient who is unable to participate in the decision-making process, the most appropriate approach would be to follow established ethical and legal guidelines. While these guidelines may vary depending on the country or healthcare system, I can provide general information on the matter.  Option D, asking the patient's wife to decide, is a reasonable approach in this situation. Spouses or next of kin are often involved in making decisions on behalf of incapacitated patients, especially when there is no advance directive or clear indication of the patient's wishes. They are typically considered the most appropriate surrogate decision-makers, as they are usually well-acquainted with the patient's values, beliefs, and preferences.  However, it is crucial to involve the healthcare team in the decision-making process. The admitting team, which consists of healthcare professionals directly involved in the patient's care, should work collaboratively with the patient's wife to discuss the available information, prognosis, potential benefits and risks of CPR, and the patient's previously expressed wishes, if known.  If there are concerns about conflicts of interest or doubts about the patient's wife's ability to make decisions in the patient's best interest, seeking the input of an independent advocate (option B) may be appropriate. Independent advocates, such as medical ethics committees or patient advocacy groups, can provide impartial guidance and help ensure that decisions are made in the patient's best interest.  Waiting 24 hours (option E) to see if the patient regains capacity to decide is generally not recommended in acute situations where immediate decisions need to be made for the patient's welfare. It is important to initiate appropriate interventions promptly based on the best available information and involve the patient's surrogate decision-maker.  While the consultant oncologist (option C) may provide valuable insights into the patient's prognosis and treatment options, the decision regarding CPR status should involve a broader team that includes the admitting team and the patient's wife or surrogate decision-maker.  Ultimately, the decision-making process should prioritize the patient's best interests, respect their autonomy to the extent possible, and comply with relevant legal and ethical frameworks. It is advisable to consult local guidelines, policies, and ethical committees for specific guidance in your jurisdiction. |
|  |  | Attempt 2  In this scenario, given the patient's inability to participate in the decision-making process and his wife's report regarding his treatment preferences, the most appropriate option would be to involve the patient's wife (option D) in determining his cardiopulmonary resuscitation (CPR) status.  The patient's wife is typically considered the most appropriate surrogate decision-maker in situations where the patient lacks capacity. Spouses or next of kin are often involved in making decisions on behalf of incapacitated patients, especially when there is no advance directive or clear indication of the patient's wishes. They are familiar with the patient's values, beliefs, and preferences and can provide insight into the patient's treatment goals.  However, it is important to involve the healthcare team, particularly the admitting team, in the decision-making process. The healthcare professionals responsible for the patient's care should collaborate with the patient's wife to discuss the available information, prognosis, potential benefits and risks of CPR, and any previously expressed wishes or treatment preferences. This collaborative approach ensures that decisions are made in the patient's best interest while respecting their autonomy as much as possible.  While it is reasonable to consider seeking input from an independent advocate (option B), such as a medical ethics committee or patient advocacy group, the patient's wife is typically the primary surrogate decision-maker in these circumstances.  Waiting 24 hours (option E) to see if the patient regains capacity to decide is generally not advisable in acute situations where immediate decisions need to be made for the patient's welfare. Prompt decision-making and appropriate interventions based on available information are necessary to ensure the patient receives the most suitable care.  While the consultant oncologist (option C) can provide valuable insights into the patient's prognosis and treatment options, the decision regarding CPR status should involve a collaborative effort that includes the admitting team and the patient's wife or surrogate decision-maker.  Ultimately, the decision-making process should prioritize the patient's best interests, respect their previously expressed wishes, and comply with relevant legal and ethical guidelines. It is recommended to consult local guidelines, policies, and ethical committees for specific guidance in your jurisdiction. |
|  |  | Attempt 3  In this scenario, since the patient is unable to communicate and has a potentially life-threatening condition, determining his cardiopulmonary resuscitation (CPR) status becomes important. The most appropriate way to establish his CPR status would be D. Ask his wife to decide.  When a patient is unable to make decisions regarding their medical care, it is common practice to involve a designated surrogate decision-maker. In this case, the patient's wife can serve as his proxy decision-maker and provide insights into his preferences and wishes regarding CPR and end-of-life care.  It is important to have a discussion with the patient's wife to understand the patient's previously expressed wishes, values, and any advance directives that may be in place. The wife can provide insight into the patient's current state of mind, treatment preferences, and goals of care. It is important to ensure that the decision-making process respects the patient's autonomy and is guided by their best interests.  While involving the consultant oncologist (C) in the discussion is important for understanding the patient's medical condition and treatment options, the ultimate decision about CPR status should be made by the patient's designated surrogate decision-maker.  Waiting 24 hours (E) to see if the patient regains capacity may not be feasible in this situation, as the patient is currently experiencing significant symptoms and their condition may deteriorate rapidly. Timely decisions regarding CPR status are crucial to guide appropriate management and ensure the patient's preferences are respected.  Involving an independent advocate (B) may be an option in situations where there is a conflict of interest or uncertainty about the patient's best interests, but in this case, the patient's wife is the most appropriate person to make decisions on his behalf.  Ultimately, the decision-making process should involve a collaborative approach between the healthcare team, the patient's designated surrogate decision-maker, and any relevant advance directives or legal frameworks in place. |
| **63** | A 36 year old man with type 1 diabetes mellitus has a pilonidal sinus. He takes 14 units long-acting insulin at night, and short-acting insulin three times daily at mealtimes (typically 6-8 units with each meal). He is scheduled for excision of the sinus under general anaesthesia, first on a morning operating list. He is asked to fast from midnight the night before. His HbA1c is 58 mmol/mol (20-42).  Which is the most appropriate plan for managing his insulin pre-operatively?  A. Omit evening and morning insulin doses  B. Start a variable-rate insulin infusion on the morning of surgery  C. Start a variable-rate insulin infusion the evening before surgery  D. Take evening and morning insulin doses, as usual  E. Take usual evening insulin and omit morning insulin | Attempt 1  The most appropriate plan for managing the patient's insulin pre-operatively would be to take the usual evening insulin and omit the morning insulin (option E).  When a patient with type 1 diabetes mellitus is scheduled for surgery, adjustments to their insulin regimen are necessary to prevent hypoglycemia during the fasting period. The goal is to maintain blood glucose levels within a target range while minimizing the risk of hypoglycemia.  In this case, the patient is asked to fast from midnight before the surgery, which means he will not be able to eat breakfast. Omitting the morning insulin dose reduces the risk of hypoglycemia during the fasting period since there will be no carbohydrate intake.  Taking the usual evening insulin dose ensures that the patient's blood glucose is adequately controlled overnight. This is important to prevent hyperglycemia during the fasting period, as elevated blood glucose levels can increase the risk of surgical complications.  It is worth noting that specific insulin management may vary depending on the individual patient, the duration of fasting, and the surgical procedure. The decision should be made in consultation with the patient's diabetes healthcare team, who can provide personalized recommendations based on the patient's insulin requirements, glycemic control, and the expected duration of fasting.  Additionally, it is important to monitor the patient's blood glucose levels perioperatively and have a plan in place for adjusting insulin dosages as needed during the surgical procedure. This ensures that the patient's blood glucose remains within the desired range and reduces the risk of perioperative complications related to glycemic control. |
|  |  | Attempt 2  The most appropriate plan for managing his insulin pre-operatively would be:  D. Take evening and morning insulin doses, as usual  It's crucial for patients with diabetes to maintain their insulin regimen as stable as possible, even during periods of fasting or when undergoing surgery. Omitting or drastically changing insulin doses without proper guidance can lead to unstable blood sugar levels, which can be risky for the patient.  In this case, the patient has type 1 diabetes and is scheduled for surgery in the morning. It's essential to continue his usual insulin regimen to avoid any significant fluctuations in blood glucose levels. Skipping insulin doses can lead to hyperglycemia, which is not only detrimental to the patient's overall health but can also negatively impact the surgical procedure and recovery.  The patient's insulin doses may need to be adjusted on the day of surgery based on the anticipated fasting duration and the individual's insulin sensitivity. However, this should be done under the guidance of his healthcare team, and any insulin adjustments should be made in a controlled manner.  A coordinated approach involving the surgeon, anesthesiologist, and endocrinologist/diabetes care team is necessary to manage the patient's blood sugar levels during the perioperative period effectively. The patient may need to have regular blood glucose monitoring and, if necessary, receive additional doses of short-acting insulin via intravenous infusion during the fasting period on the day of surgery. This helps to maintain blood sugar levels within a safe range throughout the procedure.  Therefore, the most appropriate plan is to take the evening and morning insulin doses, as usual, and make any necessary adjustments under medical supervision on the day of surgery. |
|  |  | Attempt 3  In managing the insulin regimen for the patient with type 1 diabetes mellitus who is scheduled for surgery, it is important to prevent hyperglycemia and maintain glycemic control during the perioperative period. The most appropriate plan for managing his insulin pre-operatively in this case would be E. Take usual evening insulin and omit morning insulin.  When a patient with type 1 diabetes is scheduled for surgery, it is generally recommended to continue the long-acting insulin (basal insulin) while adjusting the short-acting insulin (bolus insulin) based on the patient's fasting status and the expected duration of fasting. Omitting the morning insulin dose while continuing the evening insulin allows for better glycemic control during the fasting period before the surgery.  By taking the usual evening insulin, the patient ensures that there is a basal level of insulin in the system, which helps prevent the development of diabetic ketoacidosis (DKA) during the fasting period. Omitting the morning insulin helps prevent hypoglycemia during the fasting period, as the patient will not be consuming any meals before the surgery.  It is important to note that this is a general recommendation and the specific insulin management plan should be tailored to the individual patient's needs and in accordance with the healthcare provider's guidance. Close communication with the patient's healthcare team, including the diabetes specialist or endocrinologist, is essential to ensure safe and optimal glycemic control during the perioperative period. |
| **64** | A 3 year old girl has had a fever and a runny nose for the past 2 days. She is alert but miserable. Her temperature is 39.6°C, pulse rate 150 bpm (95-140), BP 105/62 mmHg (90/50-120/180), respiratory rate 36 breaths per minute (20-30) and oxygen saturation 97% in air. She has a red throat and looks flushed with a flat, erythematous rash across her trunk and face that feels rough. There is no rash around her mouth.  Which is the most likely diagnosis?  A. Kawasaki disease  B. Measles  C. Parvovirus infection  D. Rhinovirus infection  E. Scarlet fever | Attempt 1  The most likely diagnosis based on the given information is Scarlet fever (option E).  Scarlet fever is a bacterial infection caused by group A Streptococcus (Streptococcus pyogenes). It primarily affects children and is characterized by a combination of symptoms, including fever, sore throat, and a distinctive rash.  The key features that suggest Scarlet fever in this case are:  1. Fever: The girl has a high temperature of 39.6°C.  2. Red throat: The presence of a red throat indicates inflammation and infection in the throat.  3. Rash: The flat, erythematous rash across the trunk and face, along with a rough texture, is a classic sign of Scarlet fever. The rash may resemble a sunburn and is typically more pronounced in areas such as the underarms and groin. The absence of a rash around the mouth is also consistent with Scarlet fever.  4. Flushed appearance: The girl looks flushed, which is another common symptom of Scarlet fever.  It's important to note that a definitive diagnosis of Scarlet fever requires a throat swab to confirm the presence of group A Streptococcus bacteria. However, the clinical presentation in this case strongly suggests Scarlet fever as the most likely diagnosis.  Prompt medical attention and appropriate antibiotic treatment, typically with penicillin or amoxicillin, are crucial in managing Scarlet fever to prevent complications and reduce the spread of infection. Therefore, it is recommended that the girl be evaluated by a healthcare professional for confirmation and initiation of appropriate treatment. |
|  |  | Attempt 2  The most likely diagnosis based on the provided symptoms is:  E. Scarlet fever  Scarlet fever is a bacterial infection caused by Group A Streptococcus (Streptococcus pyogenes) and is characterized by symptoms such as fever, sore throat, and a red rash. The classic rash of scarlet fever is described as "scarlatiniform," which means a flat, erythematous rash that feels rough, similar to sandpaper. The rash typically starts on the trunk and spreads to the face, sparing the area around the mouth (also known as "circumoral pallor").  The other symptoms presented, such as fever, runny nose, flushed appearance, and red throat, are also consistent with scarlet fever. The presence of a red throat suggests inflammation of the pharynx (pharyngitis), which is common in streptococcal infections.  The other options can be ruled out based on specific clinical features:  A. Kawasaki disease usually presents with persistent fever, swollen lymph nodes, red eyes, and changes in the lips and oral mucosa. It can lead to coronary artery abnormalities.  B. Measles typically presents with a high fever, cough, runny nose, red eyes, and a characteristic rash that starts on the face and spreads downward.  C. Parvovirus infection (Fifth disease or slapped-cheek syndrome) has a characteristic rash on the cheeks, giving a "slapped cheek" appearance.  D. Rhinovirus infection is a common cause of the common cold, and it typically does not present with a specific rash.  It is important to note that a proper diagnosis can only be made by a healthcare professional after a physical examination and potentially further testing. If you suspect your child has scarlet fever or any other infectious illness, it is essential to seek medical attention promptly for appropriate evaluation and management. |
|  |  | Attempt 3  Based on the presented symptoms and findings, the most likely diagnosis in this case is E. Scarlet fever.  Scarlet fever is a bacterial infection caused by group A Streptococcus (Streptococcus pyogenes). It commonly affects children and presents with symptoms such as fever, sore throat, and a characteristic rash. The rough, erythematous rash typically starts on the trunk and spreads to the face, giving the appearance of a flushed face with circumoral pallor (known as "strawberry tongue"). The rash feels rough to the touch, similar to sandpaper.  Other symptoms of scarlet fever may include a red throat, swollen tonsils, headache, and enlarged lymph nodes. The high fever and rapid pulse rate seen in this case are also consistent with scarlet fever.  It's important to note that a definitive diagnosis of scarlet fever requires confirmation through a throat swab culture to identify the presence of group A Streptococcus bacteria. Prompt medical evaluation and appropriate treatment with antibiotics, usually penicillin or amoxicillin, are essential to manage the infection and prevent potential complications.  While other conditions such as Kawasaki disease, measles, parvovirus infection, and rhinovirus infection can also present with fever and rash, the specific combination of symptoms described in this case (including the appearance of the rash and the presence of a red throat) is most consistent with scarlet fever. |
| **65** | N/A |  |
| **66** | A 36 year old woman attends for an asthma review. She requires a long-acting β-agonist and steroid combination inhaler. The guidelines suggest either a metered dose inhaler or a dry powder. They are the same price. This patient asks which is better for the environment.  Which component of these inhalers has the highest carbon footprint?  A. Hydrofluorocarbon propellant in the metered dose inhaler  B. Packaging of the dry powder inhaler  C. Plastic component of the dry powder inhaler  D. Plastic component of the metered dose inhaler  E. Powder component of the dry powder inhaler | Attempt 1  The component of these inhalers that typically has the highest carbon footprint is the hydrofluorocarbon (HFC) propellant in the metered dose inhaler (MDI) (option A).  Metered dose inhalers use HFC propellants to deliver the medication in aerosol form. HFCs are greenhouse gases that contribute to global warming and have a high global warming potential. The production and release of HFCs into the atmosphere contribute to the carbon footprint associated with MDIs.  On the other hand, dry powder inhalers (DPIs) do not require propellants. They deliver medication in a dry powder form, usually without the use of HFCs. While DPIs may still have a carbon footprint due to other factors like packaging or plastic components, the absence of HFC propellants makes them generally more environmentally friendly compared to MDIs.  It's important to note that the overall carbon footprint of an inhaler depends on various factors, including its manufacturing process, materials used, and disposal methods. Therefore, specific inhaler brands or models may vary in their environmental impact. If the patient is concerned about the environmental aspect, it may be beneficial to consult with the healthcare provider or pharmacist who can provide information on specific inhalers available and their associated carbon footprints. |
|  |  | Attempt 2  The component of these inhalers that typically has the highest carbon footprint is:  A. Hydrofluorocarbon propellant in the metered dose inhaler  Metered dose inhalers (MDIs) use hydrofluorocarbon (HFC) propellants to deliver the medication in a spray form. These HFC propellants have a significant carbon footprint because they are potent greenhouse gases, contributing to global warming and climate change.  Dry powder inhalers (DPIs), on the other hand, do not use propellants and instead rely on the patient's inhalation to deliver the medication in a powder form. This makes them generally more environmentally friendly compared to MDIs.  While other components of inhalers, such as the plastic packaging and components, also have environmental impacts, the HFC propellants in MDIs are considered the primary contributor to their higher carbon footprint.  It's important to consider the environmental impact of medical treatments and devices, and efforts are being made to develop more eco-friendly inhalers and other medications to reduce their overall carbon footprint. |
|  |  | Attempt 3  The component of these inhalers that typically has the highest carbon footprint is the hydrofluorocarbon (HFC) propellant in the metered dose inhaler (MDI).  MDIs use HFC propellants to deliver the medication in a fine spray. HFCs are potent greenhouse gases with a high global warming potential. They contribute to the carbon footprint of the inhaler due to their impact on climate change.  On the other hand, dry powder inhalers (DPIs) do not require propellants, including HFCs, as they deliver the medication in a powdered form. This reduces their carbon footprint compared to MDIs. However, it's important to consider that DPIs may still have environmental impacts related to their packaging and disposal.  While the packaging and plastic components of both MDIs and DPIs can also contribute to their carbon footprint, the HFC propellant in MDIs is generally considered the primary factor responsible for their higher environmental impact.  It's worth noting that efforts are being made to develop more environmentally friendly inhaler options, such as inhalers that use alternative propellants with lower global warming potential or inhalers with reduced packaging waste. The choice between MDIs and DPIs should be based on individual patient factors, including inhaler technique, preference, and suitability for their specific condition, in addition to considering environmental concerns. |
| **67** | A 18 year old man is worried about his cancer risk. His paternal grandfather died of colorectal cancer at 42 years of age and his 36 year old father has just been diagnosed with colorectal cancer. The son's colonoscopy shows hundreds of colonic polyps, and biopsies from several of the polyps show adenomatous change with low grade dysplasia.  Which is the most appropriate strategy to prevent colon cancer in this situation?  A. Daily low dose aspirin  B. Diet rich in fruit and vegetables  C. Panproctocolectomy  D. Annual faecal immunochemical test (FIT)  E. Annual colonoscopy and biopsy | Attempt 1  Given the family history of early-onset colorectal cancer and the presence of multiple colonic polyps with adenomatous changes and dysplasia, the most appropriate strategy to prevent colon cancer in this situation is option C: Panproctocolectomy.  The presence of numerous colonic polyps, particularly with adenomatous changes and dysplasia, is highly suggestive of a condition called familial adenomatous polyposis (FAP). FAP is an inherited disorder that predisposes individuals to the development of colorectal cancer at an early age. The presence of hundreds of polyps increases the risk of malignant transformation significantly.  In individuals with FAP, the most effective preventive measure is the surgical removal of the colon and rectum, which is achieved through a panproctocolectomy. This procedure eliminates the risk of developing colorectal cancer and is considered the gold standard for preventing cancer in individuals with FAP.  While options A (daily low-dose aspirin) and B (diet rich in fruit and vegetables) may have some general benefits for reducing the risk of colorectal cancer in the general population, they are not sufficient in this high-risk scenario. Option D (annual FIT) and option E (annual colonoscopy and biopsy) are surveillance measures that may be appropriate in certain circumstances, but in the presence of FAP and multiple polyps with dysplasia, surgical intervention is typically recommended.  It's important for the individual to consult with a healthcare professional, such as a gastroenterologist or genetic counselor, who can provide more detailed information and guidance based on their specific case and family history. |
|  |  | Attempt 2  Given the family history of colorectal cancer and the presence of hundreds of colonic polyps with adenomatous change and low-grade dysplasia, the most appropriate strategy to prevent colon cancer in this situation is:  C. Panproctocolectomy  The presence of multiple colonic polyps, especially adenomatous polyps, indicates a condition called familial adenomatous polyposis (FAP). FAP is an inherited genetic disorder that predisposes individuals to the development of colorectal polyps, which have the potential to progress to colorectal cancer if left untreated.  In individuals with FAP, the risk of developing colorectal cancer is extremely high, often nearly 100% by the age of 40 if no intervention is undertaken. Therefore, the recommended approach to prevent colon cancer in this situation is to undergo a panproctocolectomy, which involves the surgical removal of the entire colon, rectum, and anus. This procedure significantly reduces the risk of colorectal cancer development and is the standard of care for patients with FAP.  Options A (daily low-dose aspirin), B (diet rich in fruit and vegetables), D (annual faecal immunochemical test - FIT), and E (annual colonoscopy and biopsy) are not appropriate strategies for preventing colon cancer in a high-risk patient with FAP. These approaches may have some potential benefits in the general population for certain individuals at lower risk, but they are not sufficient for individuals with FAP due to the overwhelming risk of cancer development in this condition.  It is essential for the individual to discuss their situation with a specialist or a multidisciplinary team experienced in managing patients with familial adenomatous polyposis to determine the best treatment plan tailored to their specific case. |
|  |  | Attempt 3  In this situation, the most appropriate strategy to prevent colon cancer would be a panproctocolectomy (option C), which involves the surgical removal of the entire colon and rectum.  The presence of hundreds of colonic polyps with adenomatous change and low-grade dysplasia suggests a condition called familial adenomatous polyposis (FAP). FAP is an inherited genetic disorder characterized by the development of numerous polyps throughout the colon. If left untreated, these polyps have a high risk of progressing to colorectal cancer.  Given the family history of colorectal cancer and the presence of adenomatous polyps with dysplasia in the son, the risk of developing colorectal cancer is extremely high. Therefore, a surgical intervention such as a panproctocolectomy is typically recommended in individuals with FAP to prevent the development of colorectal cancer.  Options A, B, D, and E may be beneficial for individuals at average risk of colon cancer or those with sporadic polyps, but they would not be sufficient in this high-risk situation with FAP. In such cases, early surgical intervention is crucial to reduce the risk of colorectal cancer. |
| **68** | A 21 year old man has a fear of public speaking. He is a student and has to give an oral presentation, and he feels unable to manage this. He fears that he will do something to embarrass himself or even vomit. The thought of giving his presentation gives him palpitations and makes him breathless and dizzy. He has avoided his tutor for 3 months and has not left his flat for several weeks. He recognises that his fears are irrational but feels overwhelmed.  Which is the most likely diagnosis?  A. Agoraphobia  B. Anxious avoidant personality disorder  C. Emetophobia  D. Panic disorder  E. Social phobia | Attempt 1  Based on the symptoms described, the most likely diagnosis for this individual is E. Social phobia (also known as social anxiety disorder).  Social phobia is a type of anxiety disorder characterized by a persistent fear of social situations and a strong desire to avoid them. Individuals with social phobia experience intense anxiety and distress in situations where they are exposed to potential scrutiny or evaluation by others, such as public speaking. The fear is often disproportionate to the actual threat posed by the situation.  The individual's fear of embarrassing himself or vomiting, along with the physical symptoms of palpitations, breathlessness, and dizziness, are common manifestations of social phobia. The avoidance of social situations and the impact on daily functioning, such as avoiding the tutor and staying isolated in the flat, are also consistent with this diagnosis.  While agoraphobia (option A) involves fear and avoidance of situations where escape may be difficult or embarrassing, it is not the primary diagnosis in this case because the individual's fear is specifically related to public speaking and evaluation by others, rather than a fear of being in open or public spaces.  Anxious avoidant personality disorder (option B) is a long-standing pattern of avoidance due to feelings of inadequacy and hypersensitivity to rejection, whereas this individual's symptoms are more specific to social situations involving public speaking.  Emetophobia (option C) refers to a specific fear of vomiting and may not adequately capture the broader social anxiety experienced by this individual.  Panic disorder (option D) involves recurrent panic attacks that are not limited to specific situations, whereas this individual's anxiety is primarily focused on social situations and public speaking.  It's important for the individual to seek professional help, such as from a mental health professional or therapist, who can provide a proper evaluation, diagnosis, and recommend appropriate treatment options, such as cognitive-behavioral therapy (CBT) or medication if necessary. |
|  |  | Attempt 2  The most likely diagnosis based on the symptoms described is:  E. Social phobia (also known as social anxiety disorder)  Social phobia is a type of anxiety disorder characterized by an intense fear of social situations or performance situations, such as public speaking. Individuals with social phobia may have excessive worry about being embarrassed, humiliated, or judged negatively by others during these situations. The fear is often irrational and out of proportion to the actual threat.  The symptoms described in the scenario, such as fear of public speaking, feeling unable to manage the situation, palpitations, breathlessness, dizziness, and avoidance of social interactions, are typical of social phobia. The individual recognizes that their fears are irrational, but they still feel overwhelmed and unable to control their anxiety.  Options A, C, and D are not the most likely diagnosis in this case:  A. Agoraphobia is characterized by a fear of situations where escape might be difficult or help might not be available in the event of a panic attack or other anxiety symptoms. It does not specifically involve fear of public speaking.  C. Emetophobia is the fear of vomiting or seeing others vomit. While the individual mentions the fear of vomiting, the primary concern seems to be related to social situations and public speaking.  D. Panic disorder involves recurrent and unexpected panic attacks, which are not described in this scenario. While the individual experiences palpitations, breathlessness, and dizziness, these symptoms are directly related to their fear of public speaking, rather than being sudden and unexpected panic attacks.  Option B (Anxious avoidant personality disorder) is also less likely because the symptoms described are more consistent with a specific anxiety disorder (social phobia) rather than a pervasive pattern of personality traits.  Social phobia can significantly impact an individual's daily life and functioning, and it is essential for them to seek professional help, such as from a mental health professional or therapist, to manage their anxiety and develop coping strategies for social situations like public speaking. |
|  |  | Attempt 3  The most likely diagnosis in this case is social phobia (option E). Social phobia, also known as social anxiety disorder, is a mental health condition characterized by an intense and persistent fear of social situations where the person may be scrutinized or evaluated by others. It often involves a fear of embarrassment, humiliation, or being judged negatively.  In this scenario, the individual's fear specifically revolves around public speaking, a common trigger for social phobia. He experiences symptoms such as palpitations, breathlessness, dizziness, and avoidance behaviors, which are characteristic of social phobia. The fear and avoidance have also significantly impacted his daily life and academic performance.  While other disorders such as panic disorder (option D) and specific phobias (such as emetophobia, option C) may involve anxiety symptoms, the specific context of the fear in this case (public speaking) aligns more closely with social phobia.  Agoraphobia (option A) is characterized by fear and avoidance of situations from which escape may be difficult or embarrassing, typically involving public spaces or situations. Anxious avoidant personality disorder (option B) is a pervasive pattern of social inhibition, feelings of inadequacy, and hypersensitivity to negative evaluation. While these conditions share some features with social phobia, they do not fully capture the specific presentation described in the scenario. |
| **69** | A 46 year old man has sudden onset and intense lower back pain. He has no pain or numbness in his buttocks or legs. He is diagnosed with mechanical back pain. He has no drug contra-indications.  Which is the most appropriate initial medication?  A. Amitriptyline  B. Fentanyl  C. Ibuprofen  D. Paracetamol  E. Triamcinolone | Attempt 1  For the management of sudden onset and intense lower back pain, the most appropriate initial medication would be C. Ibuprofen.  Ibuprofen is a nonsteroidal anti-inflammatory drug (NSAID) that can help reduce pain and inflammation associated with mechanical back pain. It is commonly used as a first-line treatment for acute musculoskeletal pain, including back pain. Ibuprofen can provide relief and help improve the individual's symptoms.  Amitriptyline (option A) is a tricyclic antidepressant that can be used for chronic pain conditions but is not typically the first-line choice for acute mechanical back pain.  Fentanyl (option B) is a potent opioid analgesic and is generally reserved for severe pain that is unresponsive to other medications. It is not typically used as the initial treatment for mechanical back pain.  Paracetamol (option D), also known as acetaminophen, can provide mild to moderate pain relief. However, for sudden and intense lower back pain, ibuprofen may be more effective due to its anti-inflammatory properties.  Triamcinolone (option E) is a corticosteroid medication that is primarily used for inflammatory conditions. It is not typically used as the initial treatment for mechanical back pain.  It's important to note that this response is for informational purposes only and does not replace the advice of a healthcare professional. If someone is experiencing sudden and intense lower back pain, they should consult with a healthcare provider for a proper evaluation and appropriate treatment recommendations based on their specific condition. |
|  |  | Attempt 2  The most appropriate initial medication for sudden onset and intense lower back pain, diagnosed as mechanical back pain, would be:  C. Ibuprofen  Mechanical back pain is commonly caused by muscle strain, ligament sprain, or other non-specific issues with the spine. Ibuprofen is a nonsteroidal anti-inflammatory drug (NSAID) that helps to reduce inflammation and relieve pain. It is often used as a first-line treatment for mild to moderate back pain due to its anti-inflammatory and analgesic properties.  Options A (Amitriptyline), B (Fentanyl), and E (Triamcinolone) are not appropriate initial medications for mechanical back pain:  A. Amitriptyline is a tricyclic antidepressant that is sometimes used for chronic pain conditions, including certain types of neuropathic pain. It is not typically the first choice for acute mechanical back pain.  B. Fentanyl is a potent opioid pain medication, and it is generally reserved for severe pain or for situations where other pain management approaches have not been effective. Its use carries the risk of significant side effects and potential for opioid dependence, making it inappropriate as an initial medication for mechanical back pain.  E. Triamcinolone is a corticosteroid, and while it has anti-inflammatory properties, its use is not typically recommended for mechanical back pain, especially in the acute setting.  Option D (Paracetamol) could also be considered as an initial medication for mechanical back pain. Paracetamol (acetaminophen) is a mild analgesic that can help with pain relief. It is generally considered safe and can be used as an initial option for mild to moderate pain. However, in cases of significant inflammation, as is often the case with mechanical back pain, an NSAID like ibuprofen might be more effective due to its anti-inflammatory effects.  Ultimately, the choice of medication should be made in consultation with a healthcare professional, taking into consideration the severity of the pain, individual patient factors, and any potential drug interactions or medical conditions that may influence the choice of treatment. |
|  |  | Attempt 3  In the case of a 46-year-old man with sudden onset and intense lower back pain diagnosed as mechanical back pain, the most appropriate initial medication would be option C: Ibuprofen.  Ibuprofen is a nonsteroidal anti-inflammatory drug (NSAID) that can help relieve pain and reduce inflammation, which are common symptoms associated with mechanical back pain. It is commonly used as an initial treatment for acute episodes of back pain. Ibuprofen can be effective in managing pain and improving mobility in many cases of mechanical back pain.  Amitriptyline (option A) is a tricyclic antidepressant that may be used for chronic pain management, including certain types of back pain, but it is not typically the first-line treatment for acute mechanical back pain.  Fentanyl (option B) is a potent opioid analgesic used for severe pain, but it is generally not indicated as the initial medication for mechanical back pain unless the pain is severe and other treatments have been ineffective.  Paracetamol (option D) is a mild analgesic that can be used for pain relief, including back pain. It is often used as a first-line treatment, but ibuprofen is generally preferred for its anti-inflammatory properties.  Triamcinolone (option E) is a corticosteroid medication and is not typically used as an initial treatment for mechanical back pain. Corticosteroids may be considered in certain cases if other treatments have failed or if there is evidence of inflammation or nerve root involvement. However, they are not the first-line option for most cases of mechanical back pain. |
| **70** | A 56 year old woman has had 2 months of intermittent vertigo, which typically occurs when turning over in bed or looking over her shoulder while driving. Each episode lasts for up to 1 minute. She has no hearing loss, tinnitus or ear pain.  Which test is most likely to confirm the diagnosis?  A. Audiometry  B. Carotid doppler ultrasonography  C. CT scan of head  D. Dix–Hallpike manoeuvre  E. MR of internal auditory meatus | Attempt 1  The most likely diagnosis in this case is benign paroxysmal positional vertigo (BPPV). To confirm the diagnosis, the most appropriate test would be the D. Dix-Hallpike maneuver.  The Dix-Hallpike maneuver is a diagnostic test used to assess for BPPV. During the maneuver, the patient is rapidly moved from a sitting to a supine position with their head turned to one side and extended slightly backward. This positional change can trigger vertigo and nystagmus (involuntary eye movements) characteristic of BPPV. The presence of vertigo and nystagmus during the Dix-Hallpike maneuver supports the diagnosis of BPPV.  Audiometry (option A) is a test used to assess hearing loss and would not provide specific information about BPPV.  Carotid Doppler ultrasonography (option B) is a test used to assess blood flow in the carotid arteries and is not directly related to the diagnosis of BPPV.  CT scan of the head (option C) and MR of the internal auditory meatus (option E) are imaging studies that may be ordered in specific cases but are not typically necessary for confirming the diagnosis of BPPV. BPPV can be diagnosed based on the characteristic clinical presentation and the results of the Dix-Hallpike maneuver.  Therefore, the most appropriate test to confirm the diagnosis of BPPV in this case is the Dix-Hallpike maneuver. It is a simple and reliable maneuver that can be performed during a clinical examination. However, it's important to consult with a healthcare professional for an accurate diagnosis and appropriate management based on the individual's specific condition. |
|  |  | Attempt 2  The test most likely to confirm the diagnosis in this case is:  D. Dix–Hallpike maneuver  The patient's symptoms of intermittent vertigo, particularly when triggered by specific head movements like turning over in bed or looking over her shoulder, are suggestive of benign paroxysmal positional vertigo (BPPV). BPPV is a common inner ear disorder that causes brief episodes of vertigo when there is a change in the position of the head.  The Dix–Hallpike maneuver is a diagnostic test used to evaluate and confirm the presence of BPPV. It involves placing the patient in a specific position, usually sitting upright with the head turned to one side and extended backward over the edge of the examination table. This maneuver allows the healthcare professional to observe and provoke nystagmus (involuntary eye movements) and vertigo if BPPV is present.  The other options are less likely to confirm the diagnosis of BPPV:  A. Audiometry is a hearing test that assesses the patient's hearing sensitivity. While it may be useful to rule out other causes of vertigo, it is not the primary test for confirming BPPV.  B. Carotid doppler ultrasonography is a test used to evaluate blood flow in the carotid arteries. It is not typically used to diagnose BPPV.  C. CT scan of the head may be ordered if there is suspicion of other neurological issues or when BPPV is not the leading diagnosis. However, it is not the first-line test for confirming BPPV.  E. MR of the internal auditory meatus may be used to evaluate other inner ear and cranial nerve-related issues, but it is not the primary test for diagnosing BPPV.  If the Dix–Hallpike maneuver confirms BPPV, there are specific maneuvers, such as the Epley maneuver, that can be performed to reposition the displaced particles in the inner ear and alleviate the symptoms. It is essential for the patient to consult with a healthcare professional, preferably an ear, nose, and throat (ENT) specialist or a vestibular therapist, for accurate diagnosis and appropriate management. |
|  |  | Attempt 3  The most appropriate test to confirm the diagnosis in this case would be option D: Dix-Hallpike maneuver.  The patient's symptoms, characterized by intermittent vertigo triggered by specific head movements, are suggestive of benign paroxysmal positional vertigo (BPPV). BPPV is a common cause of vertigo and is often characterized by brief episodes of spinning vertigo that can be triggered by changes in head position. The Dix-Hallpike maneuver is a diagnostic test specifically designed to assess for BPPV.  During the Dix-Hallpike maneuver, the patient is rapidly moved from a sitting to a supine position with the head turned to one side and the neck extended. The presence of vertigo and characteristic nystagmus (involuntary eye movements) upon this maneuver supports the diagnosis of BPPV.  Audiometry (option A) is a hearing test and would not be the most appropriate test to confirm the diagnosis of BPPV in this case.  Carotid doppler ultrasonography (option B) is a test used to assess blood flow through the carotid arteries and is not indicated for the diagnosis of BPPV.  CT scan of the head (option C) and MRI of the internal auditory meatus (option E) are imaging studies that may be useful in evaluating other causes of vertigo, such as vestibular schwannoma or other structural abnormalities. However, these tests are not typically necessary as the initial step in evaluating a patient with classic symptoms of BPPV. |
| **71** | A 62 year old man is brought to the operating theatre recovery room after a laryngoscopy and vocal cord biopsy. He appears to be conscious, but his breathing is shallow and respiratory rate 28 breaths per minute. His voice is weak and, when the recovery nurse asks him to squeeze her fingers with his hand, the grip is not sustained.  Which drug will reverse these signs?  A. Doxapram  B. Glycopyrronium  C. Naloxone  D. Neostigmine  E. Rocuronium | Attempt 1  The most appropriate drug to reverse the signs described in the scenario is option A: Doxapram.  The patient's clinical presentation suggests respiratory depression and neuromuscular weakness. Doxapram is a respiratory stimulant that acts by stimulating the respiratory centers in the brainstem, leading to an increase in respiratory drive and improved ventilation.  In this case, the patient is showing signs of shallow breathing, increased respiratory rate, weak voice, and impaired hand grip strength. These signs indicate respiratory depression and possible neuromuscular weakness, which could be due to the effects of anesthesia or sedation.  Glycopyrronium (option B) is an anticholinergic medication that is used to reduce excessive secretions and prevent bradycardia during surgery. It does not have a direct effect on respiratory drive or muscle strength.  Naloxone (option C) is a medication used to reverse the effects of opioid overdose or opioid-induced respiratory depression. While naloxone may be appropriate in cases of suspected opioid overdose, it is not the drug of choice in this scenario where the cause of respiratory depression and muscle weakness is not clearly related to opioids.  Neostigmine (option D) is a cholinesterase inhibitor that is used to reverse neuromuscular blockade caused by non-depolarizing neuromuscular blocking agents. However, neostigmine is not indicated in this case as the patient's symptoms are not consistent with neuromuscular blockade.  Rocuronium (option E) is a non-depolarizing neuromuscular blocking agent and would not be appropriate for reversing the respiratory depression and muscle weakness seen in this patient. |
|  |  | Attempt 2  The drug that is most likely to reverse the signs described in the scenario is:  A. Doxapram  The patient's shallow breathing, increased respiratory rate, weak voice, and decreased muscle strength (as evidenced by the weak grip) suggest that he might be experiencing respiratory depression, possibly due to the effects of anesthesia or sedation used during the laryngoscopy and vocal cord biopsy.  Doxapram is a respiratory stimulant that can help reverse respiratory depression and improve respiratory drive. It acts on the respiratory centers in the brainstem, increasing respiratory rate and depth. It is often used in postoperative settings to counteract the respiratory depressant effects of anesthesia or other medications.  The other options are not appropriate for reversing respiratory depression:  B. Glycopyrronium is an anticholinergic medication used to reduce excessive secretions and can be helpful in certain situations, but it does not address the respiratory depression seen in this case.  C. Naloxone is an opioid antagonist and is used to reverse the effects of opioid overdose. There is no mention of opioid use in the scenario, so naloxone is not the appropriate choice.  D. Neostigmine is a cholinesterase inhibitor used to reverse the effects of nondepolarizing neuromuscular blocking agents (NMBAs) like rocuronium (option E). However, the patient's respiratory depression and weak grip are not consistent with the effects of NMBAs, so neostigmine is not indicated.  E. Rocuronium is a nondepolarizing neuromuscular blocking agent (NMBA) used during surgery to achieve muscle relaxation. However, it does not cause respiratory depression on its own. If a neuromuscular blocker was used during the procedure, the patient would need to be adequately reversed with neostigmine (which is not the case here). Additionally, the patient's symptoms (shallow breathing, weak voice, weak grip) suggest central nervous system depression rather than neuromuscular blockade.  In summary, the most appropriate drug to reverse the signs of respiratory depression in this scenario is doxapram. However, it is crucial for healthcare professionals to evaluate the patient's condition thoroughly, consider potential contributing factors, and administer medications based on the specific clinical context. |
|  |  | Attempt 3  Based on the presented signs and symptoms, the patient is exhibiting respiratory depression and muscle weakness, which suggests the possibility of residual effects from anesthesia or sedation. The most appropriate drug to reverse these signs would be A. Doxapram.  Doxapram is a respiratory stimulant that acts by stimulating the central respiratory centers, leading to an increase in respiratory drive and improvement in respiratory function. It can be used to treat respiratory depression caused by anesthesia or sedative medications. By increasing the patient's respiratory rate and depth, doxapram can help alleviate the shallow breathing observed in this case.  Glycopyrronium (B) is an anticholinergic medication used to reduce excessive respiratory secretions and can be used as part of premedication before certain surgical procedures. It does not directly address the respiratory depression and muscle weakness observed in this patient.  Naloxone (C) is an opioid receptor antagonist used to reverse the effects of opioid overdose, including respiratory depression. However, there is no indication in the scenario that the patient has received opioids, so naloxone would not be the appropriate choice.  Neostigmine (D) is a medication used to reverse the effects of neuromuscular blocking agents used during surgery. It is not indicated in this case because the patient's symptoms are likely due to residual effects of anesthesia or sedation rather than neuromuscular blockade.  Rocuronium (E) is a neuromuscular blocking agent used during surgery to induce muscle relaxation. It is not appropriate for reversing the respiratory depression and muscle weakness observed in this case.  Therefore, the most appropriate drug to reverse the signs described in the scenario is A. Doxapram. However, it is important to consult with a healthcare professional for proper evaluation and management of the patient's condition. |
| **72** | A 50 year old woman has broken her right radius after tripping at home. She has been experiencing hot flushes and night sweats for the past six months, with irregular menstruation. She has been taking salbutamol and beclometasone regularly for more than five years to treat asthma. Her fracture heals with no complications.  Which investigation will most effectively evaluate her future risk of fractures?  A. 99m-technetium bone scintigraphy  B. Dual energy X-ray absorptiometry  C. MR scan of spine and hip  D. Plain X-ray lumbar spine and pelvis  E. Quantitative CT scan of spine and hip | Attempt 1  In order to evaluate the future risk of fractures in this patient, the most appropriate investigation would be B. Dual energy X-ray absorptiometry (DXA).  Dual energy X-ray absorptiometry is the gold standard for assessing bone mineral density (BMD) and diagnosing osteoporosis. It is a non-invasive and widely available test that measures BMD at the hip and spine, which are the most common sites for osteoporotic fractures. DXA provides a T-score, which compares the patient's BMD to that of a healthy young adult of the same sex. The T-score helps classify the patient's bone density as normal, osteopenic, or osteoporotic.  Given the patient's age, menopausal symptoms (hot flushes, night sweats, irregular menstruation), and the occurrence of a fracture with no complications, it is important to assess her bone health. Long-term use of salbutamol and beclometasone for asthma may also contribute to bone loss. DXA will help determine her bone mineral density and assess her future risk of fractures. This information will guide the need for preventive measures and potential treatment options for osteoporosis.  The other options listed are not the most appropriate for evaluating future fracture risk:  A. 99m-technetium bone scintigraphy is a nuclear medicine test used to evaluate bone metabolism and detect areas of increased or decreased bone activity. While it can be helpful in certain situations, it is not the primary test for assessing future fracture risk.  C. MR scan of spine and hip provides detailed images of the spine and hip, but it is not the recommended initial investigation for assessing bone mineral density or future fracture risk.  D. Plain X-ray of the lumbar spine and pelvis can identify fractures but does not provide information on bone density or future fracture risk.  E. Quantitative CT scan of the spine and hip can assess bone density, but DXA is the preferred method for this purpose due to its wider availability, lower radiation exposure, and established diagnostic criteria.  Therefore, the most appropriate investigation to evaluate this patient's future risk of fractures is B. Dual energy X-ray absorptiometry (DXA). |
|  |  | Attempt 2  The most appropriate investigation to evaluate her future risk of fractures, especially in the context of her age, menopausal symptoms, and long-term use of asthma medications, is:  B. Dual energy X-ray absorptiometry (DXA)  DXA scan is the gold standard test for assessing bone mineral density (BMD) and is commonly used to diagnose osteoporosis and evaluate the risk of future fractures. Osteoporosis is a condition characterized by low bone mass and deterioration of bone tissue, leading to increased bone fragility and a higher risk of fractures.  Given the patient's age of 50 years, her history of menopausal symptoms (hot flushes, night sweats, and irregular menstruation), and long-term use of asthma medications (which may include corticosteroids like beclometasone), she is at risk for developing osteoporosis.  Option A (99m-technetium bone scintigraphy) is not the primary test used to assess bone density or evaluate the risk of fractures. It is more commonly used to detect bone abnormalities, such as bone infections or tumors.  Option C (MR scan of spine and hip) is not the first-line investigation for assessing bone density or diagnosing osteoporosis. DXA scan is specifically designed for this purpose and is more widely available and less expensive.  Option D (Plain X-ray lumbar spine and pelvis) is not a sensitive test for detecting osteoporosis in its early stages because it primarily evaluates bone structure rather than bone density.  Option E (Quantitative CT scan of spine and hip) is a sensitive test for assessing bone density, but it is typically reserved for research purposes or when DXA scan results are inconclusive or not available.  Therefore, DXA scan (option B) is the most appropriate and effective investigation to evaluate her future risk of fractures and determine if she has osteoporosis or low bone mineral density, considering her clinical risk factors. If osteoporosis is diagnosed, appropriate management and preventive measures can be implemented to reduce the risk of future fractures. |
|  |  | Attempt 3  The most appropriate investigation to evaluate the future risk of fractures in this case is option B: Dual energy X-ray absorptiometry (DXA).  The patient's age, history of menopausal symptoms (hot flushes, night sweats, irregular menstruation), and the presence of a fragility fracture (broken radius after a low-energy fall) raise suspicion for osteoporosis. DXA is the gold standard for assessing bone mineral density (BMD) and is commonly used to diagnose osteoporosis and evaluate fracture risk.  DXA measures BMD at specific sites, typically the spine, hip, or forearm. It provides a T-score, which compares the patient's BMD to that of a healthy young adult population. T-scores between -1 and -2.5 indicate low bone mass (osteopenia), while T-scores below -2.5 are indicative of osteoporosis.  The other options mentioned are not the primary investigations for evaluating fracture risk or diagnosing osteoporosis:  - 99m-technetium bone scintigraphy (option A) is a nuclear medicine test used to assess bone metabolism and detect various bone conditions, such as bone tumors, infections, or osteomyelitis. It is not the appropriate test for evaluating future fracture risk or diagnosing osteoporosis.  - MR scan of the spine and hip (option C) is a detailed imaging study that can provide information about the anatomy, structures, and possible abnormalities of the spine and hip. While it may be helpful in certain situations, it is not the initial investigation of choice for evaluating fracture risk or diagnosing osteoporosis.  - Plain X-ray lumbar spine and pelvis (option D) can identify fractures or structural abnormalities in the spine and pelvis. However, it does not provide direct information about bone mineral density or evaluate overall fracture risk.  - Quantitative CT scan of the spine and hip (option E) is a specialized imaging technique that can assess bone density and bone strength. While it may provide additional information, it is not typically used as the primary investigation for evaluating fracture risk or diagnosing osteoporosis. DXA is more commonly employed for this purpose.  Therefore, the most appropriate investigation for assessing future fracture risk in this patient is DXA (option B). |
| **73** | A 72 year old man has had difficulty swallowing solids. He has cancer of the middle third of the oesophagus and hepatic metastases.  Which is the most appropriate initial management of his dysphagia?  A. Chemotherapy  B. Excision of tumour  C. Gastrostomy feeding tube  D. Oesophageal stent  E. Radiotherapy | Attempt 1  In the given scenario, the most appropriate initial management of dysphagia in a 72-year-old man with cancer of the middle third of the esophagus and hepatic metastases would be D. Oesophageal stent.  Esophageal stenting is a minimally invasive procedure that involves placing a stent (a hollow tube) in the esophagus to help alleviate dysphagia and improve swallowing. The stent acts as a scaffold to keep the esophageal lumen open, allowing food and liquids to pass through more easily.  Considering the patient's advanced cancer and hepatic metastases, the primary goal of treatment is to improve his quality of life by addressing the dysphagia symptoms. Chemotherapy or radiotherapy may be part of the treatment plan for the cancer itself, but they may not provide immediate relief for dysphagia. Excision of the tumor may not be feasible or appropriate at this stage due to the presence of metastases.  Gastrostomy feeding tube placement (option C) may be considered if the patient is unable to maintain adequate nutrition and hydration orally. However, as the initial management for dysphagia, oesophageal stenting would be a more suitable option to improve swallowing and maintain oral intake.  It's important to note that the specific management plan should be determined in consultation with a multidisciplinary team involving gastroenterologists, oncologists, and palliative care specialists, taking into account the patient's overall condition and goals of care. |
|  |  | Attempt 2  Given the clinical presentation of a 72-year-old man with difficulty swallowing solids, cancer of the middle third of the esophagus, and hepatic metastases, the most appropriate initial management of his dysphagia is:  C. Gastrostomy feeding tube  Gastrostomy feeding tube placement is often the most appropriate initial management for patients with dysphagia due to esophageal cancer, especially if they have difficulty swallowing and cannot maintain adequate oral intake. The gastrostomy tube provides direct access to the stomach and allows for the administration of enteral nutrition, ensuring that the patient receives adequate nutrition and hydration.  Chemotherapy (option A), excision of the tumor (option B), oesophageal stent placement (option D), and radiotherapy (option E) may be considered as part of the treatment plan for esophageal cancer. However, in the context of dysphagia and nutritional issues, addressing the immediate nutritional needs with a gastrostomy feeding tube is essential.  Depending on the patient's overall health and treatment goals, a multidisciplinary team, including gastroenterologists, oncologists, and palliative care specialists, will work together to determine the most appropriate treatment approach, which may involve a combination of interventions to manage the cancer and improve the patient's quality of life. |
|  |  | Attempt 3  In a patient with cancer of the middle third of the esophagus and hepatic metastases presenting with difficulty swallowing solids (dysphagia), the most appropriate initial management of dysphagia would be option D: Oesophageal stent.  Oesophageal stenting is a minimally invasive procedure in which a stent (a tube made of metal or plastic) is placed in the esophagus to keep it open and improve swallowing. It can provide immediate relief of dysphagia by bypassing the obstructed area caused by the tumor.  Chemotherapy (option A) and radiotherapy (option E) are treatment modalities typically used for cancer management, including esophageal cancer. However, in this case, the patient's dysphagia needs to be addressed promptly to improve his ability to swallow. Oesophageal stenting would be a more immediate and appropriate intervention before considering chemotherapy or radiotherapy.  Excision of the tumor (option B) may be an option in certain cases, but in a patient with hepatic metastases, curative surgical resection may not be feasible or appropriate as an initial management option for dysphagia.  Gastrostomy feeding tube (option C) may be considered for nutritional support in patients with severe dysphagia or inability to meet their nutritional needs orally. However, in this case, the focus is on managing the dysphagia itself rather than solely relying on enteral feeding.  Therefore, in the given scenario, the most appropriate initial management of the patient's dysphagia would be oesophageal stent placement (option D). |
| **74** | A 64 year old man has low thoracic back pain and has been tired for the past 6 weeks. He is a non-smoker. He has hypertension and takes amlodipine. His urinalysis is normal. Investigations: Haemoglobin 81 g/L (135–175) MCV 82 fL (80–96) White cell count 4.3 × 109/L (3.0–10.0) Platelets 74 × 109/L (150–400) Creatinine 203 μmol/L (60–120)  Which initial investigation is most likely to help establish the diagnosis?  A. Anti-neutrophil cytoplasmic antibody  B. Antinuclear antibody  C. Complement levels (C3 and C4)  D. Coagulation screen  E. Urine and serum protein electrophoresis | Attempt 1  Based on the provided information, the most appropriate initial investigation to help establish the diagnosis in this 64-year-old man with low thoracic back pain and fatigue is E. Urine and serum protein electrophoresis.  The patient's laboratory findings indicate several abnormalities, including a low hemoglobin level, decreased platelet count, and elevated creatinine level. These findings suggest a potential hematological or renal disorder. Given the clinical presentation and laboratory results, multiple myeloma should be considered as a differential diagnosis.  Urine and serum protein electrophoresis are valuable tests to evaluate for the presence of monoclonal gammopathy, which is a characteristic finding in multiple myeloma. The presence of a monoclonal protein (M-protein) spike on electrophoresis would support the diagnosis and guide further investigations and management.  The other options listed (A. Anti-neutrophil cytoplasmic antibody, B. Antinuclear antibody, C. Complement levels, D. Coagulation screen) are less likely to be directly associated with the symptoms and laboratory findings described in the case. They may be relevant in other clinical contexts but are not the most appropriate initial investigation in this scenario.  It's important to note that further diagnostic tests, such as bone marrow biopsy and imaging studies, would likely be required to confirm the diagnosis of multiple myeloma and assess the extent of the disease. A multidisciplinary approach involving hematologists and oncologists would be essential for proper evaluation and management of the patient. |
|  |  | Attempt 2  Given the clinical presentation and laboratory findings of a 64-year-old man with low thoracic back pain, fatigue, and abnormal blood tests, the most appropriate initial investigation to help establish the diagnosis is:  E. Urine and serum protein electrophoresis  The patient's symptoms and laboratory results suggest the possibility of a hematological disorder, specifically multiple myeloma. Multiple myeloma is a type of blood cancer that affects plasma cells in the bone marrow. It can lead to abnormal production of immunoglobulins (proteins) that can be detected in the blood and urine.  The following findings are suggestive of multiple myeloma:  1. Low hemoglobin (anemia) with a hemoglobin level of 81 g/L.  2. Elevated creatinine level (203 μmol/L) indicating possible kidney involvement, which is common in multiple myeloma.  3. Low platelet count (74 × 109/L).  4. Elevated MCV (mean corpuscular volume) of 82 fL.  To establish the diagnosis of multiple myeloma, urine and serum protein electrophoresis are typically performed to detect abnormal proteins (M-proteins) in the blood and urine. Additionally, other diagnostic tests, such as bone marrow biopsy and imaging studies, may be needed to confirm the diagnosis and assess the extent of the disease.  Options A (Anti-neutrophil cytoplasmic antibody), B (Antinuclear antibody), C (Complement levels), and D (Coagulation screen) are not relevant investigations for suspected multiple myeloma. These tests are used for different clinical scenarios and are not specific for evaluating hematological malignancies like multiple myeloma.  It is essential for the patient to undergo further evaluation and receive a definitive diagnosis and appropriate management from a hematologist or oncologist with experience in managing hematological disorders. |
|  |  | Attempt 3  In a 64-year-old man with low thoracic back pain, fatigue, and abnormal laboratory results including low hemoglobin, low platelet count, elevated creatinine, and normal urinalysis, the most likely diagnosis to consider is multiple myeloma, a type of plasma cell cancer. To establish the diagnosis, the most appropriate initial investigation would be option E: Urine and serum protein electrophoresis.  Multiple myeloma is characterized by the abnormal proliferation of plasma cells in the bone marrow, leading to the production of abnormal monoclonal proteins (M-proteins). Urine and serum protein electrophoresis can detect and quantify the M-proteins, which are usually either immunoglobulin G (IgG) or immunoglobulin A (IgA) in multiple myeloma. The presence of M-proteins, along with other clinical features and laboratory findings, helps establish the diagnosis of multiple myeloma.  The other options mentioned (options A, B, C, and D) are not typically used as initial investigations to establish the diagnosis of multiple myeloma. Anti-neutrophil cytoplasmic antibody (ANCA) and antinuclear antibody (ANA) are not specific to multiple myeloma and are more commonly associated with other autoimmune or inflammatory conditions. Complement levels (C3 and C4) are not specific for multiple myeloma and are often used to evaluate complement activation in various diseases. A coagulation screen is not specific to multiple myeloma and is more useful for assessing bleeding or clotting disorders.  Therefore, in the given clinical scenario, urine and serum protein electrophoresis (option E) would be the most appropriate initial investigation to help establish the diagnosis of multiple myeloma. |
| **75** | A 66 year old woman has become forgetful, does not sleep well and has lost her appetite over the last few weeks. She has been getting up early in the morning because she cannot sleep. She feels that she is no longer of any use to the rest of her family. Mini Mental State Examination is 27/30.  Which is the most likely diagnosis?  A. Age-associated memory impairment  B. Alzheimer’s disease  C. Depression  D. Frontotemporal dementia  E. Vascular dementia | Attempt 1  Based on the given information, the most likely diagnosis for the 66-year-old woman is C. Depression.  The patient's symptoms of forgetfulness, insomnia, loss of appetite, early morning awakening, feelings of worthlessness, and decreased self-esteem are consistent with a depressive episode. The presence of these symptoms suggests that she may be experiencing a major depressive disorder.  While cognitive impairment can be seen in both depression and dementias such as Alzheimer's disease, the Mini Mental State Examination (MMSE) score of 27/30 indicates that her cognitive function is relatively intact. In Alzheimer's disease, the cognitive deficits would be more pronounced and progressive over time. Additionally, the symptoms described are more consistent with a depressive presentation rather than the primary cognitive decline seen in Alzheimer's disease.  Age-associated memory impairment (A) typically refers to mild memory difficulties that occur with aging but do not significantly impact daily functioning. Frontotemporal dementia (D) is characterized by changes in behavior, personality, and language, which are not prominent in the given case. Vascular dementia (E) typically presents with a history of stroke or evidence of cerebrovascular disease, which is not mentioned in the scenario.  Depression (C) is the most likely diagnosis based on the patient's symptoms and the absence of significant cognitive impairment as indicated by the MMSE score. It is important for the patient to undergo further evaluation and receive appropriate management and support, which may involve a combination of psychotherapy and/or pharmacotherapy. |
|  |  | Attempt 2  The most likely diagnosis based on the provided symptoms and Mini Mental State Examination score is:  C. Depression  The woman's symptoms of forgetfulness, sleep disturbances, loss of appetite, early morning awakening, feelings of worthlessness, and perceived burden to her family are consistent with depression. Depression is a common mental health condition in older adults and can present with cognitive symptoms that might mimic early signs of dementia.  The Mini Mental State Examination (MMSE) score of 27 out of 30 suggests relatively intact cognitive function, which is not typical of Alzheimer's disease (option B) or other types of dementia. Alzheimer's disease usually presents with progressive cognitive decline and memory impairment that is more pronounced than what is described in this case.  Option A (Age-associated memory impairment) is not a specific diagnosis but rather a term used to describe age-related changes in memory that do not significantly impair daily functioning. The patient's symptoms are more indicative of a specific condition rather than just age-related changes.  Option D (Frontotemporal dementia) and option E (Vascular dementia) are types of dementia that can cause cognitive and behavioral changes, but the symptoms described in the case are more consistent with depression.  Depression can often be underdiagnosed in older adults, and it is crucial for healthcare professionals to consider and evaluate for this condition in individuals experiencing cognitive and mood-related symptoms. Prompt diagnosis and appropriate management, which may include psychotherapy and/or antidepressant medications, can significantly improve the patient's quality of life.  It is important for the patient to consult with a healthcare professional, such as a geriatrician or psychiatrist, for a comprehensive assessment and personalized treatment plan. |
|  |  | Attempt 3  In a 66-year-old woman presenting with symptoms of forgetfulness, sleep disturbances, loss of appetite, early morning awakening, feelings of worthlessness, and a Mini Mental State Examination (MMSE) score of 27/30, the most likely diagnosis is option C: Depression.  Depression is a mood disorder characterized by persistent feelings of sadness, loss of interest or pleasure, changes in appetite and sleep patterns, fatigue, difficulty concentrating, and feelings of worthlessness or guilt. Cognitive symptoms such as forgetfulness and difficulty with concentration can also be present. The symptoms described in the scenario, including sleep disturbances, loss of appetite, early morning awakening, and feelings of worthlessness, are consistent with a depressive episode.  It's important to note that depression can often present with cognitive impairment that may mimic dementia. However, the MMSE score of 27/30 suggests only mild cognitive impairment, which is commonly seen in depression. The cognitive deficits in depression are typically reversible and improve with appropriate treatment of the underlying depressive episode.  While Alzheimer's disease (option B) is a common cause of progressive cognitive decline, the presentation in this scenario is more consistent with a depressive episode rather than the characteristic memory impairment seen in Alzheimer's disease. Age-associated memory impairment (option A) is a milder form of cognitive decline that does not typically cause significant functional impairment. Frontotemporal dementia (option D) and vascular dementia (option E) are less likely given the absence of key features associated with these conditions in the scenario.  Therefore, based on the provided information, depression (option C) is the most likely diagnosis for this patient. It would be important to further evaluate and manage her depressive symptoms accordingly. |
| **76** | A consultant is looking to find published evidence on reducing the incidence of deep venous thrombosis.  Which type of study would provide the highest quality evidence?  A. Case control study  B. Case series  C. Cohort study  D. Meta-analysis of trials  E. Randomised controlled trial | Attempt 1  The highest quality evidence for reducing the incidence of deep venous thrombosis (DVT) would be provided by an E. Randomised controlled trial (RCT).  Randomised controlled trials involve the random assignment of participants into different groups, typically an intervention group and a control group, and the comparison of outcomes between the groups. In the context of reducing the incidence of DVT, an RCT would involve a study where participants are randomly assigned to receive different interventions or treatments aimed at preventing DVT. This could include comparing the effectiveness of different prophylactic measures, such as anticoagulant medications, compression stockings, or early mobilization.  Randomisation helps to minimize selection bias and ensures that any differences observed between the groups are more likely to be due to the interventions being tested rather than other confounding factors. RCTs provide a higher level of evidence because they allow for comparisons of treatment effects while controlling for potential biases and confounders.  While other study types, such as case-control studies (A), case series (B), cohort studies (C), and meta-analyses of trials (D), can provide valuable information, they are generally considered to provide lower levels of evidence compared to RCTs. These study types are more prone to biases and limitations that may affect the validity of their findings. RCTs are considered the gold standard for evaluating the efficacy and effectiveness of interventions. |
|  |  | Attempt 2  The type of study that would provide the highest quality evidence on reducing the incidence of deep venous thrombosis (DVT) is:  E. Randomised controlled trial (RCT)  Randomised controlled trials (RCTs) are considered the gold standard in clinical research for evaluating the effectiveness of interventions and treatments. In an RCT, participants are randomly assigned to different groups: one group receiving the intervention being studied (e.g., a specific method to reduce DVT incidence) and another group receiving a control or placebo treatment. This randomization helps to minimize bias and ensures that any differences observed between the groups are more likely due to the intervention being studied rather than other factors.  RCTs provide a high level of evidence because they allow researchers to establish a cause-and-effect relationship between the intervention and the outcomes. Additionally, RCTs often include blinding (single-blind or double-blind) to further reduce bias. They provide the most robust evidence on the efficacy and safety of interventions and are crucial for informing clinical practice and treatment guidelines.  Options A (Case control study), B (Case series), and C (Cohort study) are observational study designs, which can provide valuable insights into associations and risk factors. However, they do not offer the same level of evidence as RCTs, as they are more prone to bias and confounding.  Option D (Meta-analysis of trials) involves combining the results of multiple RCTs on the same topic to obtain a summary estimate of the treatment effect. While meta-analyses are a powerful tool for synthesizing evidence and increasing the sample size, the quality of the evidence ultimately depends on the quality of the individual trials included in the analysis. Therefore, a well-conducted RCT would still provide higher-quality evidence compared to a meta-analysis of trials. |
|  |  | Attempt 3  Among the options provided, the type of study that would provide the highest quality evidence on reducing the incidence of deep venous thrombosis (DVT) is option E: Randomised controlled trial (RCT).  Randomised controlled trials are considered the gold standard for evaluating the effectiveness of interventions. In an RCT, participants are randomly assigned to different groups, with one group receiving the intervention being studied (e.g., a preventive measure for DVT) and another group receiving a control intervention or placebo. This random allocation helps minimize bias and ensures that the groups are comparable at the start of the study. The outcome of interest (in this case, the incidence of DVT) is then compared between the groups.  Randomised controlled trials provide high-quality evidence because they allow for the assessment of cause and effect relationships and provide the most rigorous control over confounding variables. By randomly assigning participants to groups, RCTs can help determine whether the intervention under investigation truly has an impact on the outcome.  Other study designs mentioned in the options, such as case-control studies (option A), case series (option B), cohort studies (option C), and meta-analyses of trials (option D), have their own strengths and limitations. However, when it comes to establishing a cause-effect relationship and providing the highest quality evidence, well-designed RCTs are the preferred choice. |
| **77** | A 62 year old man has had 3 months of general weakness, fatigue, 10 kg weight loss and mild abdominal discomfort over the right hypochondrium. He was found to have liver cirrhosis 10 years ago and has abstained from alcohol since the diagnosis. He has a non-tender, fixed hard mass in the right upper quadrant.  Which serum investigation is most appropriate to aid diagnosis?  A. α-Fetoprotein  B. β-Human chorionic gonadotropin  C. CA 125  D. CA 19-9  E. Carcinoembryonic antigen | Attempt 1  In the given clinical scenario, the most appropriate serum investigation to aid in the diagnosis would be A. α-Fetoprotein (AFP).  The patient's history of liver cirrhosis and the presence of a non-tender, fixed hard mass in the right upper quadrant raise concerns about hepatocellular carcinoma (HCC), which is a common complication of cirrhosis. AFP is a tumor marker that is frequently elevated in patients with HCC. Measuring the serum AFP level can help in diagnosing and monitoring the progression of HCC.  While the other options listed (B. β-Human chorionic gonadotropin, C. CA 125, D. CA 19-9, and E. Carcinoembryonic antigen) are tumor markers used in the diagnosis and monitoring of various types of cancers, they are not specifically associated with HCC. In this context, AFP is the most relevant and appropriate serum investigation for aiding in the diagnosis of HCC in a patient with liver cirrhosis. |
|  |  | Attempt 2  Given the clinical presentation of a 62-year-old man with liver cirrhosis, general weakness, fatigue, weight loss, and a fixed hard mass in the right upper quadrant, the most appropriate serum investigation to aid in the diagnosis is:  A. α-Fetoprotein (AFP)  In a patient with liver cirrhosis and a liver mass, α-fetoprotein (AFP) is a valuable tumor marker to evaluate the possibility of hepatocellular carcinoma (HCC). HCC is the most common type of primary liver cancer, and its presence is more likely in patients with underlying liver cirrhosis, especially if they present with a liver mass.  AFP levels can be elevated in HCC, but it is essential to consider other factors that can also cause elevated AFP, such as chronic hepatitis and non-HCC liver tumors. Nevertheless, AFP is a crucial marker to investigate in this context.  Options B (β-Human chorionic gonadotropin), C (CA 125), D (CA 19-9), and E (Carcinoembryonic antigen) are tumor markers used to diagnose and monitor specific types of cancer but are not specific for HCC in the context of liver cirrhosis.  In patients with a liver mass and a history of liver cirrhosis, further imaging studies, such as ultrasound, computed tomography (CT), or magnetic resonance imaging (MRI), are usually needed to better characterize the mass and its potential malignancy. Additionally, a liver biopsy may be necessary for definitive diagnosis and to guide treatment decisions.  Prompt evaluation by a hepatologist or an oncologist is crucial to assess the liver mass and establish an accurate diagnosis, which will help determine the appropriate management plan. |
|  |  | Attempt 3  In this scenario, the most appropriate serum investigation to aid in the diagnosis would be option A: α-Fetoprotein (AFP).  AFP is a tumor marker that is commonly used in the evaluation of liver pathology, including hepatocellular carcinoma (HCC), which is the most common primary liver cancer. In patients with liver cirrhosis, there is an increased risk of developing HCC. The presence of a non-tender, fixed hard mass in the right upper quadrant raises suspicion for HCC.  Elevated levels of AFP in the blood can indicate the presence of HCC, although it is important to note that AFP can also be elevated in other liver diseases and certain non-liver malignancies. Therefore, it is used as a screening tool and as part of a comprehensive diagnostic evaluation, along with other imaging studies such as ultrasound or CT scans, to help establish the diagnosis of HCC.  The other options listed (B, C, D, and E) are associated with different types of tumors or conditions and are not specific for HCC in this context. |
| **78** | A 7 year old boy has intermittent pain in his left groin when playing football and climbing stairs. This has worsened over the past 4 weeks. He has now developed a painless limp. He has reduced range of movement and pain on internal rotation and abduction. His temperature is 37.1°C.  Which is the most likely diagnosis?  A. Osgood–Schlatter disease  B. Perthes' disease  C. Septic arthritis  D. Slipped upper femoral epiphysis  E. Transient synovitis | Attempt 1  The most likely diagnosis in this case is option B: Perthes' disease, also known as Legg-Calvé-Perthes disease.  Perthes' disease is a condition that affects the hip joint, typically occurring in children between the ages of 4 and 10. It is characterized by a disruption of the blood supply to the femoral head, leading to necrosis (death) of the bone tissue. This can result in pain, limited range of motion, and a limp.  The intermittent pain in the left groin, worsened symptoms over a few weeks, and the development of a painless limp are all suggestive of Perthes' disease. The reduced range of movement and pain on internal rotation and abduction are also consistent with this condition.  Osgood-Schlatter disease (option A) is a condition that affects the knee and causes pain and swelling just below the kneecap, typically seen in active adolescents. Septic arthritis (option C) is an infection of the joint, which usually presents with an acutely painful, swollen, and warm joint associated with systemic signs of infection such as fever. Slipped upper femoral epiphysis (option D) is a condition seen in adolescents where the growth plate of the femoral head slips, causing hip pain and an altered gait. Transient synovitis (option E) is a self-limiting inflammation of the hip joint, typically presenting with a sudden onset of hip pain and limp, often preceded by a viral infection.  Given the clinical presentation described, Perthes' disease is the most likely diagnosis. However, further evaluation, including imaging studies such as an X-ray or MRI, would be necessary to confirm the diagnosis. |
|  |  | Attempt 2  The most likely diagnosis for the 7-year-old boy's symptoms is:  E. Transient synovitis  Transient synovitis is a common condition in children, typically affecting the hip joint. It is characterized by inflammation of the synovium (lining of the joint) and can cause hip pain, reduced range of motion, and a painless limp. The symptoms are usually self-limiting and resolve within a few days to weeks.  The boy's intermittent pain in the left groin, worsened over the past 4 weeks, along with reduced range of movement and pain on internal rotation and abduction, are consistent with transient synovitis. The low-grade fever (37.1°C) can also be seen in transient synovitis.  Options A (Osgood–Schlatter disease), B (Perthes' disease), C (Septic arthritis), and D (Slipped upper femoral epiphysis) are less likely based on the symptoms and age of the patient.  A. Osgood–Schlatter disease is a condition that affects the knee, causing pain and swelling at the tibial tuberosity.  B. Perthes' disease is a condition affecting the hip joint, but it typically presents in children around 4 to 10 years of age with progressive pain and limp, not intermittent pain.  C. Septic arthritis is a serious bacterial infection of the joint, and children with this condition usually have a more acute onset of symptoms, with severe joint pain and systemic signs of infection like high fever and other inflammatory markers.  D. Slipped upper femoral epiphysis is a condition affecting the hip joint that occurs in adolescents and is uncommon in a 7-year-old.  Although transient synovitis is a benign and self-limiting condition, any child with hip pain and a limp should be evaluated by a healthcare provider to confirm the diagnosis and rule out other potential causes. Further assessment, such as imaging studies or joint aspiration, may be necessary to confirm the diagnosis and exclude other conditions. |
|  |  | Attempt 3  The most likely diagnosis for the 7-year-old boy's symptoms is:  E. Transient synovitis  Transient synovitis is a common condition in children, typically affecting the hip joint. It is characterized by inflammation of the synovium (lining of the joint) and can cause hip pain, reduced range of motion, and a painless limp. The symptoms are usually self-limiting and resolve within a few days to weeks.  The boy's intermittent pain in the left groin, worsened over the past 4 weeks, along with reduced range of movement and pain on internal rotation and abduction, are consistent with transient synovitis. The low-grade fever (37.1°C) can also be seen in transient synovitis.  Options A (Osgood–Schlatter disease), B (Perthes' disease), C (Septic arthritis), and D (Slipped upper femoral epiphysis) are less likely based on the symptoms and age of the patient.  A. Osgood–Schlatter disease is a condition that affects the knee, causing pain and swelling at the tibial tuberosity.  B. Perthes' disease is a condition affecting the hip joint, but it typically presents in children around 4 to 10 years of age with progressive pain and limp, not intermittent pain.  C. Septic arthritis is a serious bacterial infection of the joint, and children with this condition usually have a more acute onset of symptoms, with severe joint pain and systemic signs of infection like high fever and other inflammatory markers.  D. Slipped upper femoral epiphysis is a condition affecting the hip joint that occurs in adolescents and is uncommon in a 7-year-old.  Although transient synovitis is a benign and self-limiting condition, any child with hip pain and a limp should be evaluated by a healthcare provider to confirm the diagnosis and rule out other potential causes. Further assessment, such as imaging studies or joint aspiration, may be necessary to confirm the diagnosis and exclude other conditions. |
| **79** | A 28 year old primigravida attends the antenatal clinic at 35 weeks' gestation. She has 2 weeks of persistent itching of her palms, soles and abdomen. Her pregnancy has been uncomplicated to date, and she is feeling good fetal movements. She has red scratch marks on her abdomen with no rash.  Which is the most appropriate investigation?  A. Anti-mitochondrial antibody  B. Iron studies  C. Liver function tests  D. Thyroid function tests  E. Urea and electrolytes | Attempt 1  In this case, the most appropriate investigation would be C. Liver function tests.  Persistent itching of the palms, soles, and abdomen in pregnancy can be a symptom of intrahepatic cholestasis of pregnancy (ICP), also known as obstetric cholestasis. It is a condition characterized by impaired bile flow during pregnancy, leading to elevated levels of bile acids in the blood. One of the main features of ICP is pruritus (itching), typically involving the palms, soles, and abdomen.  Liver function tests, including measurement of serum bile acids and liver enzymes such as alanine transaminase (ALT) and aspartate transaminase (AST), are used to diagnose and monitor ICP. Elevated levels of bile acids, specifically serum total bile acids, are a key finding in this condition. Liver function tests can help assess the severity of the condition and guide management decisions.  The other investigations listed (anti-mitochondrial antibody, iron studies, thyroid function tests, urea and electrolytes) are not specific for intrahepatic cholestasis of pregnancy and would not be the primary investigations for this particular presentation. However, it's important to note that the definitive diagnosis and management of ICP should be made by a healthcare professional based on a thorough evaluation of the patient's symptoms, clinical findings, and appropriate investigations. |
|  |  | Attempt 2  The most appropriate investigation for this 28-year-old primigravida with persistent itching of her palms, soles, and abdomen at 35 weeks' gestation is:  C. Liver function tests  The symptoms of itching on the palms, soles, and abdomen in pregnancy, along with the absence of rash and presence of red scratch marks (also known as excoriations), are suggestive of a condition called **intrahepatic cholestasis of pregnancy (ICP)**, also known as **obstetric cholestasis**. It is a liver disorder that occurs during pregnancy and is characterized by impaired bile flow, leading to increased bile acids in the blood.  Liver function tests (LFTs) are essential to diagnose and monitor ICP. They typically include measuring levels of bile acids, transaminases (such as ALT and AST), and bilirubin. Elevated levels of bile acids, especially above the normal reference range for pregnancy, are characteristic of ICP.  The other options are not relevant to the specific symptoms and presentation of itching in pregnancy:  A. Anti-mitochondrial antibody: This test is used in the diagnosis of autoimmune liver diseases such as primary biliary cholangitis (PBC), but it is not indicated in this case as the patient's symptoms do not suggest an autoimmune condition.  B. Iron studies: Iron studies are used to assess iron levels and iron-related parameters in conditions such as anemia or iron overload, but they are not related to the symptoms of itching in this patient.  D. Thyroid function tests: Thyroid function tests are used to evaluate thyroid gland function and are not directly relevant to the current symptoms.  E. Urea and electrolytes: Urea and electrolyte tests primarily assess kidney function and electrolyte balance and are not directly related to itching symptoms during pregnancy.  It is crucial for this patient to undergo liver function tests to confirm the diagnosis of ICP and for appropriate management and monitoring, as ICP can carry some risks for both the mother and the baby if left untreated. Early detection and management are essential for a positive outcome. |
|  |  | Attempt 3  In this case, the most appropriate investigation would be option C: Liver function tests.  Persistent itching of the palms, soles, and abdomen in a pregnant woman raises concerns about intrahepatic cholestasis of pregnancy (ICP), also known as obstetric cholestasis. ICP is a condition characterized by impaired bile flow during pregnancy, leading to elevated levels of bile acids in the bloodstream. It typically presents in the third trimester and is associated with intense itching, especially on the palms and soles.  Liver function tests, including measurement of serum bile acids and liver enzymes such as alanine transaminase (ALT) and aspartate transaminase (AST), are used to diagnose and monitor ICP. Elevated levels of serum bile acids, particularly above the normal range for pregnancy, support the diagnosis of ICP.  Option A (anti-mitochondrial antibody) is not relevant in this case as it is associated with autoimmune liver diseases such as primary biliary cholangitis. Option B (iron studies) is not indicated based on the given clinical presentation. Option D (thyroid function tests) would be appropriate if there were symptoms or signs suggestive of thyroid dysfunction, which are not mentioned in the scenario. Option E (urea and electrolytes) would be helpful for assessing renal function but is not the primary investigation for evaluating itching in pregnancy.  Therefore, in the context of persistent itching in pregnancy, liver function tests, including serum bile acids, are the most appropriate investigation to evaluate for intrahepatic cholestasis of pregnancy (ICP). |
| **80** | A 23 year old woman commenced chemotherapy for Burkitt's lymphoma yesterday. Since then she has been feeling increasingly nauseated. Urine output has been 40 mL in the last 12 hours. Her temperature is 36.8°C, pulse rate 96 bpm and BP 112/80 mmHg. Investigations on admission were normal. Investigations today: Potassium 6.2 mmol/L (3.5–5.3) Urea 9 mmol/L (2.5–7.8) Creatinine 410 μmol/L (60–120)  Which investigation is most likely to identify the cause of her acute deterioration?  A. Blood cultures  B. C reactive protein  C. Creatine kinase  D. Phosphate  E. Urate | Attempt 1  In this case, the most appropriate investigation to identify the cause of the patient's acute deterioration would be A. Blood cultures.  The patient, who has recently started chemotherapy, is experiencing nausea and has a significant decrease in urine output. The abnormal laboratory findings of elevated potassium, urea, and creatinine levels suggest possible renal dysfunction, which could be caused by various factors, including infection.  Given the acute deterioration and the potential for infection as a complication of chemotherapy, obtaining blood cultures would be important. Blood cultures can help identify the presence of any systemic infection, such as sepsis, which may be contributing to the patient's symptoms and abnormal laboratory values. In patients undergoing chemotherapy, the risk of developing infections, including bloodstream infections, is increased due to the immunosuppressive effects of the treatment.  While the other investigations (C reactive protein, creatine kinase, phosphate, urate) may provide additional information in certain clinical scenarios, blood cultures would be the most appropriate initial step to identify a potential infectious cause in this patient. It is important to involve the healthcare team promptly for further evaluation and management. |
|  |  | Attempt 2  The most appropriate investigation to identify the cause of the 23-year-old woman's acute deterioration after starting chemotherapy for Burkitt's lymphoma is:  A. Blood cultures  The patient is experiencing nausea, reduced urine output, and abnormal laboratory findings, which could indicate potential complications associated with chemotherapy, such as infection. Given her symptoms and the fact that she is immunocompromised due to chemotherapy, it is crucial to rule out the possibility of an infection, which can lead to serious consequences in this setting.  Blood cultures are essential in this scenario to detect the presence of bacteria in the bloodstream, which could indicate sepsis or a severe infection. Prompt identification and treatment of any infection are critical for patients undergoing chemotherapy, as they are more susceptible to infections due to the suppression of their immune system by the treatment.  The other investigations listed are less likely to directly identify the cause of her acute deterioration:  B. C-reactive protein: Elevated C-reactive protein levels indicate inflammation but may not specifically point to the underlying cause of her symptoms.  C. Creatine kinase: Creatine kinase levels are often measured to assess muscle damage, but they are unlikely to be directly related to her symptoms.  D. Phosphate: Abnormal phosphate levels may occur in various conditions, but they might not be the primary cause of her acute deterioration.  E. Urate: Elevated urate levels are seen in conditions like gout, but they are not likely to be the cause of her current symptoms.  Considering the patient's clinical presentation and the importance of early identification and treatment of potential infections, blood cultures are the most appropriate investigation in this context. |
|  |  | Attempt 3  In this case, the most appropriate investigation to identify the cause of the acute deterioration would be option A: Blood cultures.  The patient is a 23-year-old woman who recently started chemotherapy for Burkitt's lymphoma and is now experiencing increasing nausea and decreased urine output. Her potassium level is elevated, and her creatinine level is significantly elevated, indicating kidney dysfunction. These findings raise concerns about acute kidney injury (AKI) and the possibility of an infective etiology.  Given the patient's symptoms and the potential for neutropenia following chemotherapy, obtaining blood cultures would be crucial to identify any potential bloodstream infection or sepsis. This is especially important in immunocompromised patients receiving chemotherapy, as they are at increased risk of developing infections.  Option B (C reactive protein) is a marker of inflammation and may be elevated in various conditions, including infection. However, it is not as specific as blood cultures for identifying the cause of acute deterioration. Option C (creatine kinase) is an enzyme used to assess muscle damage and would not be the most relevant investigation in this context. Option D (phosphate) and option E (urate) are not likely to provide specific information regarding the acute deterioration and are less likely to be the cause of her symptoms.  Therefore, in this scenario, obtaining blood cultures would be the most appropriate investigation to identify a potential infective cause for the acute deterioration in a patient undergoing chemotherapy. |
| **81** | A 44 year old woman is increasingly hypotensive in the high dependency unit. She was admitted 12 hours earlier with loin pain, dysuria and rigors, and was treated with intravenous broad spectrum antibiotics. Her temperature is 37.8°C, pulse rate 112 bpm, BP 91/60 mmHg, central venous pressure +12 mmHg and oxygen saturation 95% on 60% oxygen. Investigations: Sodium 139 mmol/L (135–146) Potassium 5.1 mmol/L (3.5-5.3) Urea 10.3 mmol/L (2.5-7.8) Creatinine 159 μmol/L (60–120) She has been treated with 3 L of 0.9% sodium chloride since admission, with a urine output of 400 mL in total and 10–20 mL/hr for the past 4 hours.  Which is the most appropriate next step in management?  A. Haemofiltration  B. Intravenous 500 mL gelatin over 30 minutes  C. Intravenous 500 mL 0.9% sodium chloride over 30 minutes  D. Intravenous furosemide bolus  E. Intravenous noradrenaline/norepinephrine infusion | Attempt 1  In this case, the most appropriate next step in management would be E. Intravenous noradrenaline/norepinephrine infusion.  The patient is presenting with hypotension and signs of inadequate tissue perfusion, which can be attributed to septic shock. She has been receiving fluid resuscitation with 3 L of 0.9% sodium chloride, but her urine output is low and there are signs of ongoing hypoperfusion, such as a high pulse rate, low blood pressure, and elevated central venous pressure.  Given the inadequate response to fluid resuscitation, the next step would be to initiate vasopressor therapy to improve systemic vascular resistance and blood pressure. Noradrenaline/norepinephrine is the vasopressor of choice in septic shock and is recommended as the first-line agent. It acts by increasing peripheral vascular resistance and improving cardiac output.  While the other options may have their roles in specific scenarios, in the context of septic shock with persistent hypotension, vasopressor therapy is the most appropriate next step to stabilize the patient's hemodynamics and improve perfusion to vital organs. It is important to involve the healthcare team promptly for further evaluation and management. |
|  |  | Attempt 2  Based on the clinical presentation and the patient's deteriorating condition in the high dependency unit, the most appropriate next step in management is:  E. Intravenous noradrenaline/norepinephrine infusion  The patient is experiencing hypotension and signs of inadequate perfusion, which may be due to septic shock. Septic shock is a life-threatening condition caused by a severe infection leading to systemic inflammatory response syndrome (SIRS) and subsequent organ dysfunction. The patient's increased heart rate (tachycardia), low blood pressure (hypotension), and reduced urine output are suggestive of this condition.  Intravenous noradrenaline/norepinephrine infusion is a first-line treatment for septic shock. It is used to increase systemic vascular resistance and improve blood pressure, thus improving perfusion to vital organs. This helps to maintain adequate blood flow and oxygen delivery to the tissues.  The other options are not the most appropriate next steps in management for septic shock:  A. Haemofiltration: While renal replacement therapy (e.g., hemofiltration) may be necessary in some cases of severe sepsis with acute kidney injury, it is not the immediate first-line treatment for septic shock.  B. Intravenous 500 mL gelatin over 30 minutes: Gelatin solutions are colloidal volume expanders used to increase intravascular volume. However, in septic shock, vasopressor support with drugs like noradrenaline/norepinephrine takes priority over volume expansion.  C. Intravenous 500 mL 0.9% sodium chloride over 30 minutes: Rapid infusion of 0.9% sodium chloride may provide some volume support, but it is not the primary treatment for septic shock. Vasopressor support is essential in this situation.  D. Intravenous furosemide bolus: Furosemide is a loop diuretic and is not indicated in the management of septic shock. In fact, it could further reduce intravascular volume and exacerbate hypotension.  In septic shock, the key priority is to restore and maintain adequate perfusion and oxygenation. This is achieved by starting vasopressor support, such as noradrenaline/norepinephrine infusion, along with appropriate antibiotic therapy and source control (if applicable). The patient should be managed in an intensive care setting for close monitoring and aggressive treatment. |
|  |  | Attempt 3  In this scenario, the most appropriate next step in management would be option E: Intravenous noradrenaline/norepinephrine infusion.  The patient is a 44-year-old woman who presented with symptoms suggestive of urinary tract infection (loin pain, dysuria, rigors) and is now in the high dependency unit with signs of hypotension. Her vital signs show tachycardia, hypotension, and elevated central venous pressure, indicating inadequate tissue perfusion. She has also received a large volume of intravenous fluid but has a low urine output, suggestive of poor renal perfusion.  Given the clinical picture of sepsis and inadequate tissue perfusion, the most appropriate next step in management would be to initiate a vasopressor infusion to support blood pressure and improve perfusion. Noradrenaline/norepinephrine is a commonly used vasopressor in septic shock to increase systemic vascular resistance and improve blood pressure.  Options A (haemofiltration), B (intravenous 500 mL gelatin over 30 minutes), and C (intravenous 500 mL 0.9% sodium chloride over 30 minutes) focus on fluid resuscitation, but the patient has already received a significant volume of intravenous fluid with no improvement in urine output. Therefore, adding more fluid rapidly may not be effective and could potentially worsen fluid overload. Option D (intravenous furosemide bolus) is not the most appropriate next step, as the patient's hypotension is likely due to inadequate vascular tone rather than volume overload.  Hence, initiating an intravenous noradrenaline/norepinephrine infusion to improve blood pressure and tissue perfusion is the most appropriate next step in the management of this hypotensive patient with suspected sepsis. |
| **82** | A 43 year old man has inoperable cancer of the bowel with hepatic and peritoneal metastases. He has back pain due to tumour infiltration. The pain has been controlled with regular codeine phosphate at maximum dosage. He now has bowel obstruction with vomiting and his pain has recurred because he cannot take oral medication. Investigations: Creatinine 85 μmol/L (60–120)  Which is the most effective drug to control his pain?  A. Buprenorphine transcutaneous patch  B. Codeine phosphate intramuscular injection  C. Fentanyl transcutaneous patch  D. Morphine sulfate continuous subcutaneous infusion  E. Tramadol hydrochloride intramuscular injection | Attempt 1  In this scenario, the most effective drug to control the patient's pain would be D. Morphine sulfate continuous subcutaneous infusion.  The patient has inoperable cancer of the bowel with metastases and is experiencing bowel obstruction with vomiting. This condition requires effective pain management, especially considering the recurrence of pain due to the inability to take oral medication.  Morphine sulfate is a potent opioid analgesic commonly used for the management of moderate to severe cancer pain. Continuous subcutaneous infusion allows for continuous and consistent delivery of the medication, ensuring adequate pain control over an extended period. This route is particularly suitable when the patient cannot take oral medications.  While other options, such as buprenorphine transcutaneous patch or fentanyl transcutaneous patch, may also be used for pain management in certain situations, morphine sulfate is considered the standard and most effective opioid for moderate to severe cancer pain. It provides better pain relief compared to codeine phosphate, tramadol hydrochloride, or other weaker opioids.  It is important to involve the healthcare team to determine the appropriate dosage and titration of the morphine sulfate infusion to achieve adequate pain control while minimizing side effects. |
|  |  | Attempt 2  Given the patient's current condition with bowel obstruction, vomiting, and inability to take oral medication, the most effective drug to control his pain is:  D. Morphine sulfate continuous subcutaneous infusion  Continuous subcutaneous infusion of morphine sulfate is a preferred method of pain control in patients with advanced cancer who are unable to take oral medications due to factors like bowel obstruction, vomiting, or reduced consciousness. This method allows for a steady and continuous delivery of pain relief without the need for oral intake.  The other options are not as suitable or effective for this patient's situation:  A. Buprenorphine transcutaneous patch: While buprenorphine patches can be effective for certain types of pain, they may not provide sufficient pain relief for severe pain or in patients who are already on a high dose of opioids.  B. Codeine phosphate intramuscular injection: This option is not ideal for this patient because he is already taking codeine phosphate at the maximum oral dosage, and intramuscular injections may not provide sustained pain relief.  C. Fentanyl transcutaneous patch: Fentanyl patches can be effective for chronic pain, but they may not be the best option for a patient experiencing acute pain due to bowel obstruction.  E. Tramadol hydrochloride intramuscular injection: Tramadol is a weaker opioid compared to morphine, and it may not be sufficient to control severe pain in this patient with advanced cancer.  Morphine is a strong opioid that is commonly used for the management of severe pain in cancer patients, particularly in palliative care settings. The continuous subcutaneous infusion route ensures a steady delivery of the medication and can be adjusted to provide adequate pain relief while minimizing potential side effects. This approach allows the patient to receive optimal pain control despite his current condition and inability to take oral medications. |
|  |  | Attempt 3  In this scenario, with the patient experiencing bowel obstruction and vomiting, the most effective drug to control his pain would be option D: Morphine sulfate continuous subcutaneous infusion.  The patient has inoperable cancer of the bowel with hepatic and peritoneal metastases, and he has been experiencing back pain due to tumour infiltration. The pain has been controlled with regular codeine phosphate at the maximum dosage, but now he has developed bowel obstruction with vomiting, making oral medication administration impossible.  In this situation, when oral intake is not feasible, the next step in pain management is to use an alternative route of administration. Continuous subcutaneous infusion of morphine sulfate is a common and effective method to provide continuous pain relief in patients with advanced cancer. This method involves the use of a syringe driver that delivers a continuous, controlled dose of morphine via a subcutaneous route, allowing for steady pain control.  Options A (buprenorphine transcutaneous patch), C (fentanyl transcutaneous patch), and E (tramadol hydrochloride intramuscular injection) may be viable alternatives in some cases, but given the severity of the patient's pain and the need for continuous pain relief, a continuous subcutaneous infusion of morphine sulfate is typically the most effective option.  Option B (codeine phosphate intramuscular injection) is not recommended as codeine is a weaker opioid compared to morphine, and intramuscular injections may not provide sustained pain relief.  It's important to note that the choice of pain medication and route of administration should be based on individual patient factors, pain intensity, and the healthcare provider's assessment and expertise. |
| **83** | A full term newborn boy is discharged home 48 hours after birth. The next day, his 2 year old sibling develops a florid chickenpox rash. His mother has detectable varicella antibodies. The newborn remains clinically well.  Which is the most appropriate management decision?  A. Admit for observation  B. Check antibody status for varicella antibodies  C. No action is necessary  D. Treat with aciclovir  E. Treat with zoster immune globulin | Attempt 1  In this scenario, the most appropriate management decision is C. No action is necessary.  The newborn remains clinically well, and the mother has detectable varicella antibodies, indicating that she likely has immunity to chickenpox. Maternal antibodies can provide temporary protection to the newborn, reducing the risk of severe disease if exposed to varicella.  Given that the newborn is clinically well and there is evidence of maternal immunity, there is no immediate need for admission, antibody testing, or specific treatment with aciclovir or zoster immune globulin. Close monitoring of the newborn's health should continue, and if any signs or symptoms of illness develop, prompt medical attention should be sought.  It is important for the parents to be educated about the signs and symptoms of varicella and to monitor the newborn closely for any changes in health. If any concerns arise, they should contact their healthcare provider for further evaluation and guidance. |
|  |  | Attempt 2  The most appropriate management decision for the full-term newborn boy, who is clinically well and has a mother with detectable varicella antibodies, after potential exposure to chickenpox through his 2-year-old sibling is:  C. No action is necessary  The presence of detectable varicella antibodies in the mother suggests that the newborn has likely received passive immunity from the mother through the placenta. This passive immunity can provide protection against chickenpox for the first few months of life. Therefore, in the absence of symptoms or signs of illness in the newborn, no specific action is necessary.  Option A, admitting the newborn for observation, is not necessary because he is clinically well, and there are no indications of active infection.  Option B, checking antibody status for varicella antibodies, is not warranted in this case. The mother's detectable antibodies and the newborn's current well-being are already indicative of potential immunity.  Option D, treating with aciclovir, is not indicated for a newborn without symptoms or signs of chickenpox infection.  Option E, treating with zoster immune globulin, is not applicable in this scenario. Zoster immune globulin is used for post-exposure prophylaxis against varicella-zoster virus (VZV) in individuals who are immunocompromised and susceptible to severe chickenpox infection. The newborn, in this case, is not immunocompromised and has likely received passive immunity from the mother.  In summary, as long as the newborn remains clinically well, and there are no signs of infection, no specific action or treatment is necessary. However, if any symptoms develop or there are concerns about the newborn's health, it is essential to seek immediate medical attention for a proper evaluation. |
|  |  | Attempt 3  In this scenario, the newborn boy is clinically well, and the mother has detectable varicella antibodies. Therefore, the most appropriate management decision is option C: No action is necessary.  The fact that the newborn remains clinically well and the mother has detectable varicella antibodies suggests that the newborn has received passive immunity from the mother, which can provide protection against varicella (chickenpox) infection. Maternal varicella antibodies are transferred to the fetus through the placenta, providing temporary protection during the early months of life.  Given that the newborn is not showing any signs or symptoms of illness and has received passive immunity from the mother, there is no immediate need for any specific action or treatment. The presence of a sibling with a florid chickenpox rash does not automatically necessitate admission or treatment for the newborn, as the risk of transmission to the newborn is low in this case.  It's important to note that if the newborn were to develop any signs or symptoms of illness or if there were concerns about his well-being, a healthcare professional should be consulted for further evaluation and management. |
| **84** | A 35 year old woman finds a small firm lump on self examination of her breast. It is excised and is a firm, well-circumscribed solid mass, 2.5 cm in diameter. Histology shows duct-like structures lined by regular, low columnar cells, separated by loose fibrous tissue. The margins are well defined.  Which is the most likely diagnosis?  A. Carcinoma in situ  B. Ductal carcinoma  C. Fibroadenoma  D. Intraductal papilloma  E. Paget's disease of the breast | Attempt 1  Based on the given information, the most likely diagnosis for the described histology and characteristics of the breast lump is C. Fibroadenoma.  Fibroadenomas are common benign breast tumors that often occur in women of reproductive age. They typically present as well-circumscribed solid masses and are characterized by the presence of glandular (duct-like) structures surrounded by fibrous tissue. The cells lining the duct-like structures are regular and low columnar in shape. Fibroadenomas usually have well-defined margins.  Carcinoma in situ (Option A) refers to abnormal cells confined to the ducts or lobules of the breast, without invasion into surrounding tissue. Ductal carcinoma (Option B) refers to cancer that originates in the lining of the ducts. Intraductal papilloma (Option D) is a benign growth that occurs in the milk ducts and can cause nipple discharge. Paget's disease of the breast (Option E) is a rare form of breast cancer that affects the skin of the nipple and areola.  Given the characteristics of the lump and the histological findings described, fibroadenoma is the most likely diagnosis. However, it is important to consult with a healthcare professional for a definitive diagnosis and appropriate management. |
|  |  | Attempt 2  Based on the given description and histology, the most likely diagnosis for the small firm lump in the woman's breast is:  C. Fibroadenoma  Fibroadenoma is a common benign (non-cancerous) breast tumor that typically presents as a firm, well-circumscribed mass in the breast. It is composed of a combination of glandular (duct-like structures lined by regular, low columnar cells) and stromal (fibrous tissue) elements. Fibroadenomas are usually mobile and painless and are commonly found in young women of childbearing age.  The other options can be ruled out based on the histological features and clinical presentation:  A. Carcinoma in situ: Carcinoma in situ refers to non-invasive cancer where abnormal cells are present in the milk ducts or lobules of the breast. The histology of fibroadenoma does not show features of malignancy.  B. Ductal carcinoma: Ductal carcinoma is a type of breast cancer that originates in the milk ducts. The histological features described are not characteristic of cancer.  D. Intraductal papilloma: Intraductal papilloma is a benign tumor that develops in the milk ducts of the breast. It typically presents with nipple discharge and is not associated with the features mentioned in the description.  E. Paget's disease of the breast: Paget's disease of the breast is a rare form of breast cancer that affects the skin of the nipple and areola. The histological features described do not match those of Paget's disease.  In summary, the presentation of a firm, well-circumscribed mass with histological features of duct-like structures and loose fibrous tissue is consistent with fibroadenoma, a common benign breast tumor. However, it is always important for patients to follow up with their healthcare provider for proper evaluation and management of any breast abnormalities. |
|  |  | Attempt 3  Based on the description provided, the most likely diagnosis in this case is C. Fibroadenoma.  Fibroadenomas are benign breast tumors that commonly occur in women of reproductive age. They typically present as firm, well-circumscribed masses in the breast. Histologically, fibroadenomas are characterized by the presence of duct-like structures lined by regular, low columnar cells, which are separated by loose fibrous tissue. These tumors often have well-defined margins.  Carcinoma in situ (option A) refers to the presence of abnormal cells confined to the ducts or lobules of the breast, without invasion into the surrounding tissue. Ductal carcinoma (option B) refers to a malignant tumor that originates in the ductal system of the breast. Intraductal papilloma (option D) is a benign tumor that forms in the milk ducts and can cause nipple discharge. Paget's disease of the breast (option E) is a rare form of breast cancer that affects the skin of the nipple and areola.  Given the characteristics of a well-circumscribed, solid mass with benign histology, fibroadenoma is the most likely diagnosis in this case. However, it is important to consult with a healthcare professional for an accurate diagnosis and appropriate management. |
| **85** | A 22 year old woman has intense itching and pain in her right ear that has gradually worsened over several days. She says that her hearing appears to be affected. She is a surfer. She has debris in the right ear canal, and the tympanic membrane is not visible. There is pain on pulling the pinna.  Which is the most likely diagnosis?  A. Cholesteatoma  B. Mastoiditis  C. Otitis externa  D. Otitis media  E. Ruptured tympanic membrane | Attempt 1  Based on the symptoms and findings described, the most likely diagnosis in this case is C. Otitis externa.  Otitis externa, also known as swimmer's ear, is an inflammation or infection of the outer ear canal. It is commonly caused by water exposure, leading to a breakdown of the skin barrier and subsequent infection. Surfers are at an increased risk due to frequent water exposure.  The symptoms of otitis externa include intense itching and pain in the affected ear, which can worsen over time. There may be debris or discharge present in the ear canal, and the tympanic membrane (eardrum) may not be clearly visible due to swelling or inflammation. Pain upon pulling the pinna (outer ear) is also a characteristic feature.  Cholesteatoma (Option A) is a noncancerous but potentially destructive growth of skin cells in the middle ear. Mastoiditis (Option B) is an infection of the mastoid bone, which is located behind the ear. Otitis media (Option D) is an infection or inflammation of the middle ear. A ruptured tympanic membrane (Option E) can occur as a complication of severe otitis media or trauma to the ear.  Based on the information provided, otitis externa is the most likely diagnosis. However, it is important to consult with a healthcare professional for a proper evaluation, diagnosis, and appropriate treatment. |
|  |  | Attempt 2  Based on the given information, the most likely diagnosis for the 22-year-old woman with intense itching and pain in her right ear, debris in the ear canal, and pain on pulling the pinna is:  C. Otitis externa  Otitis externa, also known as swimmer's ear, is an inflammation or infection of the external ear canal, the portion of the ear that extends from the outside to the tympanic membrane (eardrum). It is commonly associated with water exposure, which is why being a surfer is relevant in this case.  The symptoms of otitis externa include intense itching, pain, and discomfort in the ear, and the presence of debris in the ear canal is often seen. The tympanic membrane may not be visible due to the inflammation and debris blocking the view.  The other options are less likely based on the specific presentation and findings described:  A. Cholesteatoma: Cholesteatoma is an abnormal growth of skin in the middle ear, not the external ear canal. It typically presents with hearing loss, not intense itching.  B. Mastoiditis: Mastoiditis is an infection of the mastoid bone, which is located behind the ear. It is usually associated with persistent ear infections, but in this case, there is no indication of an ongoing middle ear infection.  D. Otitis media: Otitis media is an infection of the middle ear, not the external ear canal. The presentation and findings described are more consistent with an external ear canal problem.  E. Ruptured tympanic membrane: While a ruptured tympanic membrane can cause ear pain and hearing loss, it is not usually associated with itching or debris in the ear canal.  Otitis externa is commonly treated with topical antibiotics and anti-inflammatory medications. It's important for the woman to seek medical attention for a proper diagnosis and appropriate management of her condition. |
|  |  | Attempt 3  Based on the given information, the most likely diagnosis in this case is C. Otitis externa.  Otitis externa, also known as swimmer's ear, is an infection or inflammation of the external ear canal. It is commonly caused by bacterial or fungal infection, and it often occurs in individuals who engage in water-related activities, such as swimming or surfing. The symptoms of otitis externa include intense itching, pain, and discomfort in the affected ear. The presence of debris in the ear canal and pain on pulling the pinna are consistent with this diagnosis.  Cholesteatoma (option A) refers to the abnormal growth of skin cells in the middle ear, typically due to repeated ear infections. It can cause hearing loss and other complications, but the gradual worsening of symptoms and the presence of debris in the ear canal are more indicative of otitis externa.  Mastoiditis (option B) is a bacterial infection of the mastoid bone, which is located behind the ear. It is often a complication of untreated or inadequately treated otitis media. The presence of debris in the ear canal and pain on pulling the pinna are more suggestive of otitis externa rather than mastoiditis.  Otitis media (option D) refers to an infection or inflammation of the middle ear. It typically presents with symptoms such as ear pain, hearing loss, and sometimes a feeling of fullness or pressure in the ear. The presence of debris in the ear canal and the gradual worsening of symptoms are more consistent with otitis externa.  Ruptured tympanic membrane (option E) can occur as a result of severe otitis media or trauma to the ear. It can cause sudden pain, hearing loss, and sometimes discharge from the ear. However, the presence of debris in the ear canal and the gradual onset of symptoms make otitis externa a more likely diagnosis.  It is important for the individual to consult with a healthcare professional for a proper examination, accurate diagnosis, and appropriate treatment. |
| **86** | A 35 year old woman has a painful lump in her breast. Her mother had breast cancer at the age of 65 years. She is 32 weeks pregnant with no past medical history. She is not taking any medication. She smokes 5 cigarettes per day. There is a 2 cm lump in her right breast that is tender on palpation. There is redness of the overlying skin.  Which is the most appropriate management?  A. Bilateral mammogram  B. No further investigations until after delivery  C. Start oral cefalexin  D. Surgical excision of lump  E. Triple assessment | Attempt 1  Given the information provided, the most appropriate management option in this case is E. Triple assessment.  Triple assessment refers to a comprehensive approach for evaluating breast lumps, which includes clinical assessment, imaging, and tissue sampling. It is particularly important to evaluate breast lumps in pregnant women to rule out any concerning pathology.  In this scenario, the woman is 32 weeks pregnant and presents with a painful lump in her breast along with redness of the overlying skin. These symptoms raise concerns for possible inflammatory breast conditions or infection, which require further evaluation.  Options A and B (bilateral mammogram and no further investigations until after delivery) may not be the most suitable choices at this stage, as mammography is generally not recommended during pregnancy due to concerns about radiation exposure to the fetus. Additionally, given the presence of symptoms and redness, further evaluation is warranted before deciding to postpone investigations until after delivery.  Option C (starting oral cefalexin) may be appropriate if there is a suspicion of a breast abscess, but a proper assessment and diagnosis should be made before initiating antibiotic treatment.  Option D (surgical excision of the lump) may be considered if there is a high suspicion of malignancy based on the clinical assessment and imaging findings. However, a comprehensive evaluation should be performed before deciding on surgical intervention.  Therefore, the most appropriate management in this case is option E, which involves a triple assessment. This would typically include a thorough clinical examination, breast imaging such as ultrasound, and potentially a tissue sample for histological analysis (e.g., core needle biopsy) to determine the nature of the breast lump and guide further management decisions. It is important to involve a multidisciplinary team, including a breast specialist and obstetrician, to ensure appropriate care for the pregnant patient. |
|  |  | Attempt 2  Given the clinical presentation of a 35-year-old pregnant woman with a painful lump in her breast, redness of the overlying skin, and a family history of breast cancer, the most appropriate management option would be:  E. Triple assessment  Triple assessment is the recommended approach for evaluating breast lumps, especially in cases where there is concern about breast cancer. It involves three components:  1. Clinical examination: A thorough examination by a healthcare professional to assess the characteristics of the lump, its size, shape, tenderness, and any associated skin changes.  2. Imaging: In this case, performing an ultrasound is the preferred imaging modality for breast lumps in pregnant women due to concerns about radiation exposure with mammograms.  3. Fine-needle aspiration cytology or core biopsy: To obtain a sample of cells from the lump to examine under a microscope, providing more information about its nature and potential malignancy.  Bilateral mammograms are not recommended during pregnancy due to the risks of radiation exposure to the developing fetus.  Starting oral cefalexin (C) is not appropriate in this context since the lump and redness suggest a breast issue rather than an infectious process.  Surgical excision of the lump (D) may be considered later depending on the results of the triple assessment, but it's not the initial step in the evaluation.  No further investigations until after delivery (B) would not be prudent, given the red flags of a painful lump and redness of the skin. Breast cancer can occur in pregnant women, and early detection and management are crucial.  Therefore, the best course of action would be to perform a triple assessment to determine the nature of the lump and the appropriate management plan, taking into consideration the patient's pregnancy and family history of breast cancer. |
|  |  | Attempt 3  Given the information provided, the most appropriate management in this case would be E. Triple assessment.  Triple assessment is a comprehensive approach to evaluating breast lumps and involves three components: clinical assessment, radiological imaging, and pathological assessment. It is the recommended approach for the evaluation of breast abnormalities, particularly in cases where cancer is suspected.  In this scenario, the patient presents with a painful lump in her breast, redness of the overlying skin, and a family history of breast cancer. These features raise concerns about the possibility of breast malignancy. Therefore, a thorough evaluation is warranted.  The components of triple assessment include:  1. Clinical assessment: This involves a detailed clinical examination by a healthcare professional to assess the characteristics of the lump, such as size, location, consistency, tenderness, and presence of skin changes. The redness of the overlying skin and tenderness in this case are important clinical findings.  2. Radiological imaging: Mammography is the recommended imaging modality for breast evaluation. However, in a pregnant woman, mammography is generally avoided due to the potential risk to the developing fetus. Instead, ultrasound is the preferred imaging modality during pregnancy as it does not involve ionizing radiation. It can provide valuable information about the nature of the breast lump.  3. Pathological assessment: A tissue biopsy is necessary to obtain a definitive diagnosis. This can be done through a core needle biopsy or an excisional biopsy, depending on the characteristics of the lump and other clinical factors.  Bilateral mammogram (option A) is not recommended during pregnancy due to the potential harm to the fetus from radiation exposure.  Delaying further investigations until after delivery (option B) may not be appropriate if there are concerning features suggestive of breast malignancy. Prompt evaluation and appropriate management are essential to ensure the best outcomes for both the mother and the baby.  Starting oral cefalexin (option C) is not indicated in this case. The presence of redness of the overlying skin and a palpable lump suggests a breast infection (mastitis) is less likely to be the cause of these symptoms.  Surgical excision of the lump (option D) should be performed only after completing the triple assessment and obtaining a definitive diagnosis.  It is important for the patient to consult with a healthcare professional specializing in breast health, such as a breast surgeon or a breast specialist, to initiate the appropriate evaluation and management. |
| **87** | A 35 year old woman is admitted unconscious to the Emergency Department after being found collapsed outside a pub. There was an empty vodka bottle lying next to her. Her pulse is 86 bpm, BP 112/62 mmHg, respiratory rate 12 breaths per minute and oxygen saturation is 98% breathing air.  Which is the most important immediate investigation?  A. Arterial blood gas  B. Blood alcohol level  C. Capillary blood glucose  D. Urea and electrolytes  E. Urinary drug screen | Attempt 1  In this scenario, the most important immediate investigation would be C. Capillary blood glucose.  Given the patient's history of being found unconscious outside a pub with an empty vodka bottle nearby, it is crucial to assess for any potential underlying causes of her altered mental state. One of the most common and easily reversible causes of altered consciousness is hypoglycemia (low blood glucose levels). Checking the capillary blood glucose level is a rapid and simple test that can provide valuable information about the patient's metabolic status.  Option A (arterial blood gas) may be useful to assess the patient's respiratory and acid-base status, but it would not be the most important immediate investigation in this case, as it may not directly contribute to identifying the cause of the patient's altered consciousness.  Option B (blood alcohol level) may be relevant in determining the patient's alcohol intoxication level, but it would not be the most critical investigation at this moment, especially considering that her airway, breathing, and circulation are stable.  Option D (urea and electrolytes) and option E (urinary drug screen) may be relevant investigations to consider in the broader evaluation of the patient, but they would not provide immediate information to guide initial management.  Therefore, in this acute presentation, the most important immediate investigation is option C, capillary blood glucose, to assess for hypoglycemia as a potentially reversible cause of the patient's unconsciousness. Prompt identification and correction of hypoglycemia, if present, can help improve the patient's level of consciousness and guide further management decisions. |
|  |  | Attempt 2  Given the clinical presentation of an unconscious woman with a history of being found collapsed outside a pub with an empty vodka bottle nearby, the most important immediate investigation to perform is:  C. Capillary blood glucose  The reason for choosing capillary blood glucose is that the symptoms of unconsciousness and collapse may be related to alcohol intoxication, but it is crucial to rule out other potentially life-threatening causes, such as hypoglycemia (low blood sugar) or diabetic ketoacidosis. Both of these conditions can lead to altered mental status and unconsciousness.  Hypoglycemia can result from excessive alcohol consumption, especially if the person has not eaten, leading to depletion of glucose stores in the body. Diabetic ketoacidosis is a serious complication of diabetes and can occur in individuals who have poorly controlled diabetes and may have consumed alcohol.  The other investigations (arterial blood gas, blood alcohol level, urea and electrolytes, and urinary drug screen) may provide valuable information later on for the management of the patient, but ruling out hypoglycemia or diabetic ketoacidosis is critical for immediate intervention and patient safety. Once hypoglycemia and diabetic ketoacidosis are ruled out, further investigations can be pursued to evaluate the cause of unconsciousness and collapse fully. |
|  |  | Attempt 3  In this scenario, the most important immediate investigation would be C. Capillary blood glucose.  The patient's presentation with unconsciousness and the presence of an empty vodka bottle raises concerns about a potential alcohol-related event. However, it is crucial to first rule out a common and potentially life-threatening condition that can cause similar symptoms, which is hypoglycemia (low blood sugar).  Capillary blood glucose measurement provides a quick and reliable assessment of the patient's blood sugar level. Hypoglycemia can lead to loss of consciousness and mimic the effects of alcohol intoxication. Prompt identification and treatment of hypoglycemia are vital to prevent further deterioration and potential complications.  Once hypoglycemia is ruled out or appropriately managed, other investigations can be considered to further evaluate the patient's condition, such as arterial blood gas (option A) to assess respiratory and metabolic status, blood alcohol level (option B) to confirm alcohol intoxication, urea and electrolytes (option D) to assess renal function and electrolyte balance, and urinary drug screen (option E) to screen for other substances that may have contributed to the patient's collapse. However, addressing the possibility of hypoglycemia takes precedence in this acute presentation. |
| **88** | A 28 year old woman has a lobar pneumonia and is treated with intravenous amoxicillin. A few minutes after she is given the antibiotic therapy, she develops an itchy skin eruption and increased breathlessness.  Which is the most likely mechanism of this reaction?  A. Antibody binding to cell-bound antigen  B. Complement activation  C. Delayed-type hypersensitivity  D. Immediate hypersensitivity  E. Immune complex hypersensitivity | Attempt 1  The most likely mechanism of the described reaction is D. Immediate hypersensitivity.  Immediate hypersensitivity reactions, also known as Type I hypersensitivity reactions, are characterized by an immediate onset of symptoms after exposure to an allergen. These reactions involve the activation of mast cells and basophils, leading to the release of inflammatory mediators such as histamine. Symptoms commonly include itching, skin rash, and breathlessness.  In this case, the patient developed an itchy skin eruption and increased breathlessness shortly after receiving intravenous amoxicillin, indicating an immediate hypersensitivity reaction. It is important to note that immediate hypersensitivity reactions can range from mild, localized symptoms to severe, systemic reactions such as anaphylaxis.  Option A (antibody binding to cell-bound antigen) is characteristic of Type II hypersensitivity reactions, which involve antibodies binding to specific antigens on the surface of cells, leading to cell destruction.  Option B (complement activation) is characteristic of Type III hypersensitivity reactions, which involve the formation of immune complexes that activate complement, leading to tissue inflammation.  Option C (delayed-type hypersensitivity) is characteristic of Type IV hypersensitivity reactions, which typically manifest hours to days after exposure to an antigen and involve the activation of T cells.  Option E (immune complex hypersensitivity) is similar to Type III hypersensitivity reactions and involves the formation of immune complexes that deposit in tissues and trigger inflammation.  Therefore, based on the timing and clinical presentation described, the most likely mechanism of the patient's reaction is immediate hypersensitivity (Type I hypersensitivity). |
|  |  | Attempt 2  The most likely mechanism of the reaction described in the 28-year-old woman after being given intravenous amoxicillin is:  D. Immediate hypersensitivity  Immediate hypersensitivity, also known as Type I hypersensitivity, is an allergic reaction that occurs rapidly after exposure to an allergen, in this case, amoxicillin. The typical features of Type I hypersensitivity include the release of histamine and other inflammatory mediators, leading to symptoms like itching, skin eruption (hives or urticaria), and increased breathlessness (shortness of breath or wheezing).  In this scenario, the woman's symptoms of itchy skin eruption and increased breathlessness are consistent with an allergic reaction to amoxicillin, and the rapid onset supports the idea of an immediate hypersensitivity reaction.  The other mechanisms mentioned (antibody binding to cell-bound antigen, complement activation, delayed-type hypersensitivity, and immune complex hypersensitivity) are associated with different types of immune reactions but are less likely to be the cause of the immediate symptoms observed in this case. |
|  |  | Attempt 3  The most likely mechanism of the described reaction in the given scenario is D. Immediate hypersensitivity.  Immediate hypersensitivity reactions, also known as type I hypersensitivity reactions, are characterized by a rapid onset of symptoms after exposure to an allergen. This type of reaction involves the activation of IgE antibodies, which are specific to the triggering antigen. When the allergen interacts with the IgE antibodies bound to mast cells and basophils, it triggers the release of various chemical mediators, such as histamine, leukotrienes, and prostaglandins.  In the case described, the patient developed an itchy skin eruption and increased breathlessness shortly after receiving intravenous amoxicillin, suggesting an immediate hypersensitivity reaction. This type of reaction can manifest as urticaria (itchy skin rash), angioedema (swelling of deeper tissues), and bronchospasm, leading to increased breathlessness.  It is important to recognize and promptly manage immediate hypersensitivity reactions as they can progress rapidly and lead to severe systemic manifestations, including anaphylaxis. Treatment typically involves discontinuing the offending agent and administering appropriate medications such as antihistamines, corticosteroids, and, if necessary, bronchodilators or epinephrine in severe cases.  The other mechanisms mentioned (A. Antibody binding to cell-bound antigen, B. Complement activation, C. Delayed-type hypersensitivity, and E. Immune complex hypersensitivity) are not consistent with the acute and immediate nature of the described reaction. |
| **89** | A 39 year old woman has had worsening tiredness for 2 weeks. She was previously well. She is mildly jaundiced. Her pulse rate is 96 bpm and BP 112/76 mmHg. Investigations: Haemoglobin 48 g/L (115–150) White cell count 6.2 × 109/L (4.0–11.0) Platelets 165 × 109/L (150–400) Mean cell volume (MCV) 98 fL (80–96) Alkaline phosphatase 100 IU/L (25–115) Aspartate aminotransferase (AST) 27 IU/L (10–40) Bilirubin (total) 41 μmol/L (< 21) Lactate dehydrogenase 560 IU/L (70–250) Blood film: red cell polychromasia, occasional spherocytes, no red cell fragments  Which is the most appropriate diagnostic investigation?  A. Bone marrow aspiration  B. Direct antiglobulin test  C. Serum antinuclear antibody  D. Serum folate  E. Serum vitamin B₁₂ | Attempt 1  Based on the clinical presentation and laboratory findings, the most appropriate diagnostic investigation for this patient is E. Serum vitamin B₁₂ levels.  The patient is presenting with tiredness, mild jaundice, and laboratory findings consistent with hemolytic anemia (low hemoglobin, presence of polychromasia, occasional spherocytes). These findings suggest the possibility of a hemolytic process, and vitamin B₁₂ deficiency can be one of the causes.  Vitamin B₁₂ deficiency can lead to ineffective erythropoiesis and the production of enlarged, poorly functioning red blood cells. This can result in anemia and characteristic red cell changes seen on the blood film, such as polychromasia and macrocytosis (elevated MCV).  Other causes of hemolytic anemia should also be considered, but given the potential association between vitamin B₁₂ deficiency and the blood film findings, checking serum vitamin B₁₂ levels would be a reasonable initial step in the diagnostic workup.  Option A (bone marrow aspiration) may be considered if other causes of anemia need to be investigated further or if there are atypical findings that require evaluation of the bone marrow.  Option B (direct antiglobulin test) is useful in evaluating autoimmune hemolytic anemia but may not be the most appropriate initial investigation in this case.  Option C (serum antinuclear antibody) is more relevant in the evaluation of autoimmune conditions, such as systemic lupus erythematosus, which may present with symptoms similar to those described by the patient. However, given the laboratory findings, serum vitamin B₁₂ is a more specific investigation to explore first.  Option D (serum folate) can be considered in cases of suspected folate deficiency anemia, but the patient's laboratory findings and clinical presentation are more consistent with vitamin B₁₂ deficiency.  Therefore, among the given options, serum vitamin B₁₂ levels would be the most appropriate diagnostic investigation for this patient. |
|  |  | Attempt 2  Based on the clinical presentation and laboratory findings, the most appropriate diagnostic investigation for this patient is E. Serum vitamin B₁₂ levels.  The patient is presenting with tiredness, mild jaundice, and laboratory findings consistent with hemolytic anemia (low hemoglobin, presence of polychromasia, occasional spherocytes). These findings suggest the possibility of a hemolytic process, and vitamin B₁₂ deficiency can be one of the causes.  Vitamin B₁₂ deficiency can lead to ineffective erythropoiesis and the production of enlarged, poorly functioning red blood cells. This can result in anemia and characteristic red cell changes seen on the blood film, such as polychromasia and macrocytosis (elevated MCV).  Other causes of hemolytic anemia should also be considered, but given the potential association between vitamin B₁₂ deficiency and the blood film findings, checking serum vitamin B₁₂ levels would be a reasonable initial step in the diagnostic workup.  Option A (bone marrow aspiration) may be considered if other causes of anemia need to be investigated further or if there are atypical findings that require evaluation of the bone marrow.  Option B (direct antiglobulin test) is useful in evaluating autoimmune hemolytic anemia but may not be the most appropriate initial investigation in this case.  Option C (serum antinuclear antibody) is more relevant in the evaluation of autoimmune conditions, such as systemic lupus erythematosus, which may present with symptoms similar to those described by the patient. However, given the laboratory findings, serum vitamin B₁₂ is a more specific investigation to explore first.  Option D (serum folate) can be considered in cases of suspected folate deficiency anemia, but the patient's laboratory findings and clinical presentation are more consistent with vitamin B₁₂ deficiency.  Therefore, among the given options, serum vitamin B₁₂ levels would be the most appropriate diagnostic investigation for this patient. |
|  |  | Attempt 3  Based on the provided clinical information and laboratory findings, the most appropriate diagnostic investigation in this case is E. Serum vitamin B₁₂ level.  The patient presents with worsening tiredness, mild jaundice, and laboratory findings suggestive of anemia (low hemoglobin) and macrocytosis (high mean cell volume, MCV). These findings raise suspicion of megaloblastic anemia, which can be caused by deficiencies in vitamin B₁₂ or folate.  Vitamin B₁₂ deficiency is more common in adults and can be associated with symptoms such as fatigue, jaundice, and macrocytic anemia. It can result from inadequate dietary intake, malabsorption, or impaired absorption due to conditions such as pernicious anemia. In contrast, folate deficiency is more commonly associated with poor dietary intake, malabsorption, or increased demand (e.g., pregnancy).  In this case, the presence of macrocytosis, along with the absence of significant liver enzyme elevations and hemolysis-related findings on the blood film, makes megaloblastic anemia more likely than other causes. To differentiate between vitamin B₁₂ and folate deficiency, measuring serum vitamin B₁₂ and folate levels is essential.  Bone marrow aspiration (A) may be considered if the diagnosis remains unclear or if there are additional indications such as suspected hematological malignancy. The direct antiglobulin test (B) is used to evaluate immune-mediated hemolytic anemia and is not the primary investigation for macrocytic anemia. Serum antinuclear antibody (C) is used in the evaluation of autoimmune diseases, but it is not the initial investigation for macrocytic anemia. Serum folate (D) levels can be considered as a secondary investigation if the vitamin B₁₂ level is normal or in cases where folate deficiency is suspected based on clinical history or risk factors. However, given the presentation and laboratory findings in this case, vitamin B₁₂ deficiency is a more likely cause and should be investigated first. |
| **90** | A 65 year old woman has two episodes of vaginal bleeding. Her last menstrual period was 12 years ago. Abdominal and pelvic examination are normal. Pelvic ultrasound scan shows an endometrial thickness of 10 mm.  Which is the most appropriate next investigation?  A. CA 125  B. Cervical cytology  C. CT scan of pelvis, abdomen and chest  D. Hysteroscopy and endometrial biopsy  E. MR scan of pelvis | Attempt 1  Given the clinical presentation of postmenopausal vaginal bleeding in a 65-year-old woman, the most appropriate next investigation would be D. Hysteroscopy and endometrial biopsy.  Postmenopausal vaginal bleeding is considered abnormal and requires further evaluation to rule out underlying pathology, including endometrial cancer. The endometrial thickness of 10 mm found on the pelvic ultrasound scan is within the range of normal, but it does not exclude the possibility of endometrial pathology.  Hysteroscopy allows direct visualization of the uterine cavity, providing an opportunity to assess the endometrium for any abnormalities, such as polyps, hyperplasia, or malignancy. It also allows for targeted biopsy or sampling of suspicious areas for histopathological analysis, which is the gold standard for diagnosing endometrial cancer.  Option A (CA 125) is a tumor marker that is not specific to endometrial cancer and is typically not used as an initial investigation for postmenopausal bleeding.  Option B (cervical cytology) is primarily used for screening and detection of cervical cancer. Postmenopausal bleeding is typically not related to cervical pathology, and therefore, cervical cytology is not the most appropriate next investigation.  Option C (CT scan of pelvis, abdomen, and chest) and option E (MR scan of pelvis) are imaging modalities that may be used in certain situations to evaluate for metastatic disease or assess the extent of local invasion. However, these investigations are not the first-line approach in evaluating postmenopausal bleeding.  Therefore, the most appropriate next investigation in this case would be D. Hysteroscopy and endometrial biopsy to directly evaluate the endometrium and obtain histopathological confirmation of any abnormal findings. |
|  |  | Attempt 2  Given the clinical presentation and pelvic ultrasound findings in a 65-year-old woman with postmenopausal vaginal bleeding, the most appropriate next investigation is:  D. Hysteroscopy and endometrial biopsy  Postmenopausal vaginal bleeding is considered abnormal and requires thorough investigation to rule out underlying serious conditions, such as endometrial cancer or other uterine abnormalities.  An endometrial thickness of 10 mm in a postmenopausal woman is considered abnormally thick and may warrant further evaluation. Hysteroscopy allows direct visualization of the uterine cavity, and an endometrial biopsy allows obtaining a sample of the endometrial tissue for examination under the microscope. These procedures are often performed together to evaluate the endometrium for any abnormalities, including endometrial hyperplasia or endometrial cancer.  Options A, B, and E are not the most appropriate next investigations in this case:  - CA 125 (Option A) is a tumor marker that can be elevated in some cases of ovarian cancer, but it is not specific for ovarian cancer and is not the primary investigation for postmenopausal vaginal bleeding.  - Cervical cytology (Option B) is used to screen for cervical cancer but is not relevant in this case because the patient is postmenopausal, and the bleeding is likely originating from the endometrium rather than the cervix.  - MR scan of the pelvis (Option E) is not the first-line investigation for postmenopausal vaginal bleeding. Hysteroscopy and endometrial biopsy are more appropriate and effective in this context.  Option C (CT scan of pelvis, abdomen, and chest) is not the most appropriate next investigation for postmenopausal vaginal bleeding and an endometrial thickness of 10 mm. CT scans are not typically used to evaluate endometrial abnormalities. The focus should be on direct visualization and biopsy of the endometrium through hysteroscopy. |
|  |  | Attempt 3  In a postmenopausal woman presenting with vaginal bleeding, an endometrial thickness of 10 mm raises concern for endometrial pathology. The most appropriate next investigation in this case is D. Hysteroscopy and endometrial biopsy.  Postmenopausal vaginal bleeding is considered abnormal and requires further evaluation to rule out underlying pathology, including endometrial cancer. While endometrial thickness is an important factor in assessing the risk of endometrial cancer, it is not definitive in establishing the diagnosis. An endometrial thickness of 10 mm falls within the intermediate range and warrants further investigation.  Hysteroscopy and endometrial biopsy allow direct visualization and sampling of the endometrium, providing a more accurate assessment of endometrial pathology. It is considered the gold standard for evaluating postmenopausal bleeding and can help identify any suspicious or abnormal findings, such as endometrial hyperplasia or endometrial cancer.  The other options mentioned are not the initial investigations of choice in this scenario:  A. CA 125 is a tumor marker primarily used in the evaluation of ovarian cancer and is not the first-line investigation for postmenopausal bleeding.  B. Cervical cytology (Pap smear) is primarily used for the detection of cervical cancer and is not the primary investigation for postmenopausal bleeding.  C. CT scan of the pelvis, abdomen, and chest may be considered in specific situations if there are additional concerning clinical findings or suspected metastatic disease but is not the initial investigation for postmenopausal bleeding.  E. MR scan of the pelvis is not the first-line investigation for postmenopausal bleeding and is typically reserved for specific indications when additional information beyond ultrasound is needed.  It's important for the patient to undergo further evaluation with hysteroscopy and endometrial biopsy to assess the cause of her vaginal bleeding and determine the appropriate management. |
| **91** | A 25 year old man develops a muscle contraction in his neck causing pain and an involuntary rotation of his neck. He was admitted to the psychiatric unit 24 hours ago with persecutory delusions, agitation, and auditory hallucinations. He has been given risperidone since admission.  Which side effect is he experiencing?  A. Akathisia  B. Autonomic instability  C. Dystonia  D. Parkinsonism  E. Tardive dyskinesia | Attempt 1  The side effect that the 25-year-old man is likely experiencing is C. Dystonia.  Dystonia is a movement disorder characterized by sustained muscle contractions, causing repetitive or twisting movements and abnormal postures. In this case, the involuntary rotation of the neck is indicative of dystonia.  The fact that the patient has been recently started on risperidone, an antipsychotic medication, suggests that the dystonia is likely a medication-induced side effect. Antipsychotic medications, including risperidone, can sometimes cause acute dystonic reactions, especially within the first few days of starting the medication or after a dose increase.  The other options listed are different side effects associated with antipsychotic medications:  A. Akathisia: Akathisia is a condition characterized by motor restlessness and a subjective sense of inner restlessness. It is often described as an uncomfortable feeling of needing to move. It is not associated with the symptoms described in the case.  B. Autonomic instability: Autonomic instability refers to dysregulation of the autonomic nervous system and can manifest with symptoms such as changes in blood pressure, heart rate, and body temperature. It is not consistent with the symptoms described in the case.  D. Parkinsonism: Parkinsonism refers to a group of motor symptoms similar to those seen in Parkinson's disease, such as tremor, rigidity, and bradykinesia (slowness of movement). It is not consistent with the symptoms described in the case.  E. Tardive dyskinesia: Tardive dyskinesia is a movement disorder characterized by repetitive, involuntary movements, typically involving the face, tongue, and other parts of the body. It is a side effect that can occur after prolonged use of antipsychotic medications, especially older typical antipsychotics. It is not the most likely explanation for the acute symptoms described in the case.  Therefore, based on the given information, the most likely side effect the patient is experiencing is C. Dystonia. |
|  |  | Attempt 2  The side effect the 25-year-old man is experiencing is:  C. Dystonia  Dystonia is a movement disorder characterized by sustained muscle contractions that result in repetitive, twisting movements or abnormal postures. In this case, the muscle contraction in his neck causing pain and involuntary rotation of his neck is consistent with dystonia.  The patient's psychiatric symptoms (persecutory delusions, agitation, and auditory hallucinations) and the fact that he has been given risperidone, an antipsychotic medication, suggest that the dystonia may be a side effect of the medication.  The other options are:  A. Akathisia: A side effect of antipsychotic medications characterized by restlessness, a subjective feeling of inner restlessness, and a compelling need to move. It is not consistent with the patient's symptoms.  B. Autonomic instability: This refers to dysregulation of the autonomic nervous system, leading to abnormalities in blood pressure, heart rate, sweating, and temperature regulation. It is not a likely explanation for the patient's symptoms.  D. Parkinsonism: A syndrome that resembles Parkinson's disease, characterized by bradykinesia (slowness of movement), rigidity, tremors, and postural instability. It is not consistent with the patient's symptoms.  E. Tardive dyskinesia: A late-onset movement disorder that can develop after prolonged use of certain medications, including antipsychotics. It is characterized by involuntary, repetitive movements of the face and tongue. While tardive dyskinesia is a potential side effect of antipsychotic medications, the acute onset of the patient's symptoms within 24 hours of starting risperidone is more suggestive of acute dystonia. |
|  |  | Attempt 3  The side effect the patient is most likely experiencing is C. Dystonia.  Dystonia is a movement disorder characterized by involuntary muscle contractions that cause repetitive or twisting movements and abnormal postures. It can affect various body parts, including the neck. In this case, the patient's symptoms of muscle contraction and involuntary rotation of the neck are consistent with dystonia.  Risperidone is an antipsychotic medication commonly used in the treatment of schizophrenia and other psychiatric disorders. While it can be effective in managing symptoms such as persecutory delusions and hallucinations, it is also associated with the risk of extrapyramidal side effects, including dystonia.  The other options mentioned are also potential side effects of antipsychotic medications but do not match the specific symptoms described in the case:  A. Akathisia refers to a subjective feeling of restlessness and an urge to move. It is characterized by motor restlessness and an inability to sit or stand still. It does not involve muscle contractions or involuntary neck rotation.  B. Autonomic instability refers to dysregulation of the autonomic nervous system and may present with symptoms such as fluctuations in blood pressure, heart rate, and body temperature. It does not manifest as muscle contractions or involuntary neck rotation.  D. Parkinsonism refers to symptoms resembling Parkinson's disease, such as bradykinesia (slowness of movement), rigidity, and tremors. It does not typically present with isolated muscle contractions or involuntary neck rotation.  E. Tardive dyskinesia is a delayed-onset movement disorder characterized by repetitive, involuntary, and purposeless movements, often involving the face and tongue. It does not typically present with isolated muscle contractions or involuntary neck rotation.  It is important for the patient to be evaluated by a healthcare professional to confirm the diagnosis of dystonia and determine the appropriate management, which may include adjusting the dosage or discontinuing the medication. |
| **92** | An 18 year old woman with sickle cell disease attends the Emergency Department with severe pain in her left leg. She rates her pain score as 8/10. Her temperature is 36.9°C, pulse rate 110 bpm, BP 120/80 mmHg, respiratory rate 16 breaths per minute and oxygen saturation 96% breathing air. Investigations: White cell count 7.1 x 109/L (4.0–11.0) Haemoglobin 71 g/L (115–150) Platelets 190 × 109/L (150–400)  Which is the next management step?  A. Intravenous co-amoxiclav  B. Oral prednisolone  C. Red cell transfusion  D. Subcutaneous morphine  E. Treatment dose dalteparin | Attempt 1  The next management step for the 18-year-old woman with sickle cell disease and severe pain in her left leg is C. Red cell transfusion.  The patient's presentation is consistent with a vaso-occlusive crisis, a common complication of sickle cell disease. The severe pain in her left leg, along with her low hemoglobin level and history of sickle cell disease, support this diagnosis.  Red cell transfusion is an important intervention in the management of vaso-occlusive crises. Transfusion helps to increase the oxygen-carrying capacity of the blood, improve tissue oxygenation, and reduce sickling of red blood cells, thereby relieving pain and preventing further complications.  The other management options are not the most appropriate next step in this scenario:  A. Intravenous co-amoxiclav: Antibiotic therapy may be necessary if there is evidence of infection, but there is no indication of infection provided in the information given. Antibiotics should be used judiciously based on clinical findings.  B. Oral prednisolone: Oral corticosteroids such as prednisolone may be used in the management of acute pain crisis in sickle cell disease, but their use is typically reserved for patients with acute chest syndrome or other specific indications. There is no indication for oral prednisolone in this case.  D. Subcutaneous morphine: Analgesia is an important component of managing pain in sickle cell disease. However, in the context of severe pain and a low hemoglobin level, red cell transfusion is the priority before considering other analgesic options.  E. Treatment dose dalteparin: Dalteparin is a low molecular weight heparin used for prophylaxis or treatment of thromboembolic events. There is no specific indication for its use in this case.  Therefore, based on the given information, the next management step for the patient is C. Red cell transfusion. |
|  |  | Attempt 2  The next management step for the 18-year-old woman with sickle cell disease presenting with severe pain in her left leg and a hemoglobin level of 71 g/L (115-150) is:  C. Red cell transfusion  The patient is experiencing a vaso-occlusive crisis, which is a common complication of sickle cell disease. Vaso-occlusive crises are characterized by the blockage of small blood vessels by sickled red blood cells, leading to severe pain and tissue ischemia. The low hemoglobin level (71 g/L) indicates that she is anemic, likely due to the ongoing hemolysis and increased red blood cell destruction.  Red cell transfusion is an appropriate management step to increase the hemoglobin level and improve the oxygen-carrying capacity of the blood, which can help alleviate the symptoms and reduce tissue damage caused by the vaso-occlusive crisis. Transfusion should be considered in cases of severe anemia, significant pain, or other complications of sickle cell disease.  Options A (Intravenous co-amoxiclav) and E (Treatment dose dalteparin) are not indicated in the management of a vaso-occlusive crisis in sickle cell disease.  Option B (Oral prednisolone) is not the first-line treatment for vaso-occlusive crises in sickle cell disease. While corticosteroids may have a role in some cases, red cell transfusion is a more appropriate initial step to address the acute anemia and pain.  Option D (Subcutaneous morphine) may be used to manage severe pain in this patient, but addressing the underlying anemia and vaso-occlusion with a red cell transfusion is a priority.  As always, the management of sickle cell disease crises should be carried out under the guidance of a healthcare professional experienced in the care of patients with this condition. |
|  |  | Attempt 3  The next management step for the 18-year-old woman with sickle cell disease and severe pain in her left leg is C. Red cell transfusion.  The patient's clinical presentation and laboratory findings suggest a vaso-occlusive crisis, which is a common complication of sickle cell disease. The severe pain in her left leg, along with a low hemoglobin level and mild leukocytosis, are indicative of an acute sickle cell pain crisis.  Red cell transfusion is often indicated in the management of vaso-occlusive crises to improve oxygen delivery, reduce sickling of red blood cells, and alleviate symptoms. Transfusion therapy can help increase the hemoglobin level and replace sickled red blood cells with healthy red blood cells, thereby improving tissue oxygenation and relieving pain.  Intravenous co-amoxiclav (A) is not the next appropriate step in managing a vaso-occlusive crisis. Antibiotics may be indicated if there are signs of infection, but in this case, there is no evidence to suggest an infection.  Oral prednisolone (B) is commonly used in the management of acute exacerbations of asthma or other inflammatory conditions but is not the first-line treatment for vaso-occlusive crises in sickle cell disease.  Subcutaneous morphine (D) or other analgesic medications are important for pain management in sickle cell disease, but in this case, the patient's pain severity and the need for immediate intervention suggest that red cell transfusion should be the first step.  Treatment dose dalteparin (E) is an anticoagulant medication used for thromboprophylaxis but is not indicated in the acute management of vaso-occlusive crises in sickle cell disease.  It is essential for the patient to be assessed and treated promptly by a healthcare professional experienced in managing sickle cell disease and its complications. |
| **93** | A 79 year old woman has had malaise and pain in the arms and legs for 4 weeks, with morning stiffness that lasts for 3 hours each day. She has difficulty washing and dressing. She cannot lift her arms above her head due to pain, but there is no objective muscle weakness. She has Heberden's nodes in her hands. Investigations: Haemoglobin 112 g/L (115–150) White cell count 9.8 × 109/L (4.0–11.0) Platelets 365 × 109/L (150–400)Mean cell volume (MCV) 89 fL (80–96) CRP 67 mg/L (<5)  Which is the most likely diagnosis?  A. Osteoarthritis  B. Osteomalacia  C. Polymyalgia rheumatica  D. Polymyositis  E. Rheumatoid arthritis | Attempt 1  The most likely diagnosis for the 79-year-old woman with malaise, pain in the arms and legs, morning stiffness, and difficulty with activities of daily living is C. Polymyalgia rheumatica.  Polymyalgia rheumatica (PMR) is a relatively common inflammatory condition that primarily affects older adults, especially those over the age of 50. It is characterized by pain and stiffness, typically in the shoulder and hip girdles, along with constitutional symptoms such as malaise, fatigue, and weight loss. Morning stiffness lasting for several hours is a hallmark feature of PMR.  The elevated C-reactive protein (CRP) level supports the diagnosis of PMR, as it is an acute-phase reactant that is often elevated in inflammatory conditions. The presence of Heberden's nodes (bony nodules at the distal interphalangeal joints) is common in osteoarthritis but is not specific to it.  Osteoarthritis (choice A) primarily affects the joints and is characterized by joint pain, stiffness, and limited range of motion. However, it does not typically cause systemic symptoms or morning stiffness lasting for several hours.  Osteomalacia (choice B) is a metabolic bone disorder characterized by inadequate mineralization of bone matrix, usually due to vitamin D deficiency. It can cause generalized bone pain and muscle weakness but is less likely in this case due to the absence of objective muscle weakness and the presence of elevated CRP.  Polymyositis (choice D) is an inflammatory myopathy characterized by proximal muscle weakness, elevated muscle enzymes, and evidence of muscle inflammation on biopsy. The absence of objective muscle weakness makes this diagnosis less likely.  Rheumatoid arthritis (choice E) is an autoimmune condition that primarily affects the joints, causing symmetric polyarthritis. It typically presents with joint swelling, tenderness, and morning stiffness that lasts longer than 1 hour. The absence of joint involvement in this case makes rheumatoid arthritis less likely.  Therefore, based on the given information, the most likely diagnosis for this patient is C. Polymyalgia rheumatica. |
|  |  | Attempt 2  Based on the clinical presentation and investigation results, the most likely diagnosis for the 79-year-old woman is:  C. Polymyalgia rheumatica  Polymyalgia rheumatica (PMR) is an inflammatory disorder that primarily affects older adults. It is characterized by bilateral pain and stiffness, most commonly in the shoulders, hips, neck, and pelvic girdle. Morning stiffness lasting for at least 30 minutes and affecting the patient's ability to perform daily activities is a hallmark of PMR. Elevated inflammatory markers, such as an elevated CRP level, are commonly seen in PMR.  Key features supporting the diagnosis of PMR in this patient's case are the malaise, pain in the arms and legs, morning stiffness lasting for 3 hours, and the inability to lift her arms above her head due to pain. The presence of Heberden's nodes in her hands may suggest coexisting osteoarthritis, but the overall clinical picture is more consistent with PMR.  Options A (Osteoarthritis) and E (Rheumatoid arthritis) are less likely because they do not typically present with the prominent morning stiffness lasting for hours as seen in this case.  Option B (Osteomalacia) is not likely as it is a disorder characterized by softening of the bones due to vitamin D deficiency, and the patient's symptoms and laboratory results do not support this diagnosis.  Option D (Polymyositis) is less likely because polymyositis usually presents with objective muscle weakness, which is not described in the patient's case. The lack of muscle weakness and the presence of significant stiffness are more suggestive of PMR.  However, it's essential to note that the final diagnosis should be made by a healthcare professional based on a thorough clinical assessment, additional investigations, and exclusion of other possible causes. |
|  |  | Attempt 3  The most likely diagnosis for the 79-year-old woman presenting with malaise, pain in the arms and legs, morning stiffness, difficulty with daily activities, inability to lift her arms above her head, and the presence of Heberden's nodes is C. Polymyalgia rheumatica.  Polymyalgia rheumatica (PMR) is a common inflammatory condition that primarily affects individuals over the age of 50. It is characterized by bilateral pain and stiffness in the shoulder and hip girdles, often with associated systemic symptoms such as malaise, fatigue, and weight loss. Morning stiffness lasting for more than 1 hour is a typical feature of PMR. In PMR, there is no objective muscle weakness or muscle enzyme elevation, which distinguishes it from other inflammatory muscle disorders.  The presence of Heberden's nodes, which are bony enlargements at the distal interphalangeal joints commonly seen in osteoarthritis (A), is not consistent with the clinical picture described. Osteoarthritis typically presents with joint pain and stiffness, but it does not cause systemic symptoms such as malaise or morning stiffness lasting for several hours.  Osteomalacia (B) is a metabolic bone disorder characterized by inadequate mineralization of the bone matrix, usually due to vitamin D deficiency. While osteomalacia can cause generalized musculoskeletal pain, it is not typically associated with morning stiffness or Heberden's nodes.  Polymyositis (D) is a systemic autoimmune disorder that primarily affects the muscles, leading to muscle weakness and elevated muscle enzymes. The absence of objective muscle weakness in the patient described makes polymyositis an unlikely diagnosis.  Rheumatoid arthritis (E) is a chronic autoimmune inflammatory disorder that primarily affects the joints. Morning stiffness is a hallmark feature of rheumatoid arthritis, but the presence of Heberden's nodes and the absence of joint involvement in the patient's history make rheumatoid arthritis less likely.  It is important for the patient to be evaluated by a healthcare professional experienced in rheumatology to confirm the diagnosis and initiate appropriate treatment for polymyalgia rheumatica. |
| **94** | A 30 year old woman becomes acutely short of breath. She was admitted to hospital 3 hours ago with an acute exacerbation of asthma. She improved following treatment with oxygen, nebulised salbutamol and oral prednisolone. Her chest X-ray on admission was clear. Her pulse rate is 122 bpm, BP 88/50 mmHg, respiratory rate 30 breaths per minute and oxygen saturation 88% breathing 40% oxygen via a face mask. She has reduced expansion of the upper left chest. She has mild wheeze throughout the chest with reduced breath sounds over the left apex.  Which is the most likely explanation for her deterioration?  A. Anaphylaxis  B. Increasing severity of asthma  C. Lobar collapse  D. Pneumothorax  E. Pulmonary embolus | Attempt 1  The most likely explanation for the patient's deterioration in this case is D. Pneumothorax.  A pneumothorax occurs when air accumulates in the pleural space, causing lung collapse. It can occur spontaneously or as a result of trauma or underlying lung disease, such as asthma. The patient's symptoms of acute shortness of breath, reduced expansion of the upper left chest, and reduced breath sounds over the left apex are consistent with a pneumothorax.  The patient's initial improvement with treatment for asthma exacerbation suggests that the initial presentation was primarily due to asthma. However, the subsequent deterioration and the presence of physical examination findings suggestive of pneumothorax indicate a new complication.  Anaphylaxis (choice A) is a severe allergic reaction that can cause respiratory distress, but it typically presents with additional features such as generalized urticaria, angioedema, and hypotension. The absence of these features makes anaphylaxis less likely in this case.  Increasing severity of asthma (choice B) is a possibility, but the sudden onset of symptoms, reduced expansion of the chest, and reduced breath sounds over a specific area are more suggestive of a focal problem like a pneumothorax.  Lobar collapse (choice C) could be considered as a differential diagnosis, but the sudden nature of the deterioration and the presence of physical examination findings supporting pneumothorax make it a less likely explanation.  Pulmonary embolus (choice E) is a serious condition that can cause acute shortness of breath, but it typically presents with other features such as pleuritic chest pain, hemoptysis, or signs of deep vein thrombosis. The absence of these features makes pulmonary embolus less likely in this case.  Therefore, based on the given information, the most likely explanation for the patient's deterioration is D. Pneumothorax. Immediate evaluation and management, including chest X-ray and appropriate intervention such as chest tube insertion, should be considered. |
|  |  | Attempt 2  Given the clinical presentation and the patient's history of acute exacerbation of asthma, the most likely explanation for her deterioration is:  D. Pneumothorax  A pneumothorax occurs when air accumulates in the pleural space, causing partial or complete collapse of the affected lung. In patients with asthma, especially during an acute exacerbation, there is an increased risk of pneumothorax due to the increased pressure in the airways during severe bronchoconstriction and excessive coughing.  The presence of reduced expansion of the upper left chest, reduced breath sounds over the left apex, and mild wheeze throughout the chest indicate possible lung collapse or compression. Additionally, her oxygen saturation of 88% despite receiving 40% oxygen via a face mask suggests poor gas exchange, which can be a result of reduced lung function due to a pneumothorax.  Option B (Increasing severity of asthma) is a possible consideration, but given her initial improvement following treatment and the new findings on examination, pneumothorax is more likely in this case.  Options A (Anaphylaxis), C (Lobar collapse), and E (Pulmonary embolus) are less likely based on the presented clinical picture. Anaphylaxis is not suggested by the symptoms and examination findings, lobar collapse would typically present with different clinical features, and pulmonary embolus is not the primary consideration in a patient with a known history of asthma and ongoing asthma symptoms.  It's important to recognize that this is a medical emergency, and the patient should receive immediate evaluation and appropriate intervention to confirm the diagnosis and manage the pneumothorax effectively. |
|  |  | Attempt 3  The most likely explanation for the patient's acute deterioration is D. Pneumothorax.  A pneumothorax occurs when air accumulates in the pleural space, leading to lung collapse. In this case, the patient has reduced expansion of the upper left chest, reduced breath sounds over the left apex, and clinical signs of respiratory distress, including tachypnea, hypoxia, and increased work of breathing. These findings, along with the absence of wheezing in the left apex, suggest a possible pneumothorax.  Asthma exacerbations (B) typically present with wheezing throughout the chest, but they do not typically cause reduced breath sounds or asymmetric chest expansion. While the patient's symptoms initially improved with treatment for asthma, the subsequent acute deterioration raises concern for an additional complication.  Anaphylaxis (A) is characterized by a systemic allergic reaction and can cause respiratory distress. However, it is less likely in this case as the patient's symptoms are more consistent with a localized pulmonary issue rather than a generalized allergic reaction.  Lobar collapse (C) could present with reduced breath sounds and decreased chest expansion localized to the affected lobe. However, the absence of wheezing in the left apex and the acute onset of symptoms suggest a more acute process, such as a pneumothorax.  Pulmonary embolus (E) is a possibility in patients with acute shortness of breath, but the clinical findings described in this case, including asymmetric chest expansion, reduced breath sounds, and absence of wheezing in the left apex, are more suggestive of a pneumothorax.  Given the patient's clinical presentation, immediate management should include prompt recognition of the potential pneumothorax, placement of a chest tube for decompression, and further evaluation to determine the underlying cause and appropriate treatment. |
| **95** | A 40 year old man had a laparotomy for intestinal obstruction 12 hours ago. He has an epidural for analgesia and has been given a 500 mL intravenous crystalloid bolus. He has no pain. He has no pre-existing medical problems. His pulse rate is 120 bpm and BP 80/62 mmHg. He has passed 10 mL of urine in the past 4 hours. He shows no sign of heart failure.  Which is the most appropriate first line of management?  A. IV diuretic  B. IV fluid bolus  C. IV vasoconstrictor  D. Place bed head down  E. Stop epidural analgesia | Attempt 1  In this case, the most appropriate first line of management for the patient's hypotension and decreased urine output would be B. IV fluid bolus.  The patient's presentation suggests hypovolemia, which is likely due to the combination of intraoperative fluid loss during the laparotomy and inadequate fluid replacement postoperatively. The low blood pressure, tachycardia, and decreased urine output are signs of inadequate tissue perfusion.  Administering an intravenous fluid bolus will help restore intravascular volume and improve tissue perfusion. A fluid bolus of isotonic crystalloid, such as normal saline or lactated Ringer's solution, is typically recommended in this situation. The appropriate volume of the bolus would depend on the patient's clinical condition and the response to initial resuscitation.  IV diuretic (choice A) would not be appropriate in this case, as the patient is hypovolemic and requires volume expansion.  IV vasoconstrictor (choice C) may be considered in certain situations of refractory hypotension, but initial fluid resuscitation should be the first step in managing hypovolemia.  Placing the bed head down (choice D) may help improve venous return in certain cases, but it is not the most appropriate initial step in managing hypovolemia.  Stopping epidural analgesia (choice E) may be considered if there is concern about the epidural causing hypotension, but the primary issue in this case is likely inadequate fluid replacement rather than the epidural itself.  Therefore, the most appropriate first line of management for this patient's hypotension and decreased urine output is to administer an IV fluid bolus (choice B). Close monitoring of the patient's response to fluid resuscitation and further interventions as needed, such as adjusting fluid administration or considering vasopressor support, may be required. |
|  |  | Attempt 2  The most appropriate first line of management in this scenario is B. IV fluid bolus.  The patient's clinical presentation suggests hypovolemia and inadequate fluid resuscitation. He has tachycardia, hypotension, and oliguria, which are signs of inadequate circulating volume. Given that he has undergone a laparotomy and received an epidural for analgesia, it is important to consider the possibility of fluid loss during surgery and the need for adequate intravascular volume to maintain organ perfusion.  The initial management of hypovolemia involves fluid resuscitation to restore circulating volume and improve tissue perfusion. Therefore, the first step is to administer an IV fluid bolus to rapidly increase intravascular volume and improve blood pressure and organ perfusion. Crystalloid solutions, such as normal saline or lactated Ringer's solution, are commonly used for initial fluid resuscitation.  Other options are not the most appropriate first line of management in this scenario:  A. IV diuretic would exacerbate the hypovolemia and is not indicated at this point. Diuretics promote urine output and can worsen hypovolemia in a patient who is already oliguric.  C. IV vasoconstrictor may be considered in certain cases of hypotension that are refractory to fluid resuscitation, but it is not the first-line treatment. Fluid resuscitation is the initial step to correct hypovolemia, and vasoconstrictors are typically reserved for cases where fluid resuscitation alone is insufficient.  D. Placing the bed head down (Trendelenburg position) may help improve venous return and cardiac preload in some cases, but it is not the primary intervention for managing hypovolemia. Fluid resuscitation should be the initial step to restore circulating volume.  E. Stopping the epidural analgesia may be considered if there is concern about its contribution to hypotension, but it is not the first-line intervention. The priority is to address the underlying hypovolemia by administering IV fluids to restore intravascular volume.  In summary, in a patient with tachycardia, hypotension, oliguria, and no signs of heart failure following surgery, the most appropriate initial management is to administer an IV fluid bolus to restore intravascular volume and improve tissue perfusion. |
|  |  | Attempt 3  The most appropriate first line of management in this scenario is B. IV fluid bolus.  The patient's clinical presentation suggests hypovolemia and inadequate fluid resuscitation. He has tachycardia, hypotension, and oliguria, which are signs of inadequate circulating volume. Given that he has undergone a laparotomy and received an epidural for analgesia, it is important to consider the possibility of fluid loss during surgery and the need for adequate intravascular volume to maintain organ perfusion.  The initial management of hypovolemia involves fluid resuscitation to restore circulating volume and improve tissue perfusion. Therefore, the first step is to administer an IV fluid bolus to rapidly increase intravascular volume and improve blood pressure and organ perfusion. Crystalloid solutions, such as normal saline or lactated Ringer's solution, are commonly used for initial fluid resuscitation.  Other options are not the most appropriate first line of management in this scenario:  A. IV diuretic would exacerbate the hypovolemia and is not indicated at this point. Diuretics promote urine output and can worsen hypovolemia in a patient who is already oliguric.  C. IV vasoconstrictor may be considered in certain cases of hypotension that are refractory to fluid resuscitation, but it is not the first-line treatment. Fluid resuscitation is the initial step to correct hypovolemia, and vasoconstrictors are typically reserved for cases where fluid resuscitation alone is insufficient.  D. Placing the bed head down (Trendelenburg position) may help improve venous return and cardiac preload in some cases, but it is not the primary intervention for managing hypovolemia. Fluid resuscitation should be the initial step to restore circulating volume.  E. Stopping the epidural analgesia may be considered if there is concern about its contribution to hypotension, but it is not the first-line intervention. The priority is to address the underlying hypovolemia by administering IV fluids to restore intravascular volume.  In summary, in a patient with tachycardia, hypotension, oliguria, and no signs of heart failure following surgery, the most appropriate initial management is to administer an IV fluid bolus to restore intravascular volume and improve tissue perfusion. |
| **96** | A 23 year old man has had joint pains for the last 6 months. He thinks he may have contracted hepatitis B following unprotected sex 6 months ago and is unsure of his hepatitis B vaccination status. Investigations: HBsAg –ve anti-HBc IgG +ve anti-HBs IgG +ve  What is the best description of his hepatitis B status?  A. Acute infection  B. Chronic infection  C. Immune due to naturally resolved infection  D. Immune due to previous vaccination for hepatitis B  E. Not infected but susceptible to further infection | Attempt 1  Based on the provided information, the best description of his hepatitis B status is E. Not infected but susceptible to further infection.  The presence of negative HBsAg (hepatitis B surface antigen) indicates that the individual is not currently infected with hepatitis B virus. The presence of positive anti-HBc IgG (hepatitis B core antibody IgG) suggests past exposure to hepatitis B virus, but it does not indicate the current infection status. The positive anti-HBs IgG (hepatitis B surface antibody IgG) indicates the presence of protective antibodies against hepatitis B surface antigen.  Considering the individual's joint pains and the timeframe of 6 months, it is unlikely that the joint pains are directly related to acute hepatitis B infection. Therefore, options A and B (acute and chronic infection) can be ruled out.  Option C (immune due to naturally resolved infection) is not applicable in this case because the individual's hepatitis B status is not confirmed as resolved. The absence of HBsAg alone does not provide enough information to determine whether the infection has resolved.  Option D (immune due to previous vaccination for hepatitis B) is also unlikely because the individual is unsure of his vaccination status, and the presence of anti-HBs IgG alone does not differentiate between vaccine-induced immunity and natural infection.  Therefore, the most appropriate description of his hepatitis B status based on the given information is E. Not infected but susceptible to further infection. This indicates that he does not currently have hepatitis B infection but remains at risk of acquiring it in the future if exposed to the virus. |
|  |  | Attempt 2  Based on the provided investigation results, the best description of his hepatitis B status is D. Immune due to previous vaccination for hepatitis B.  The negative HBsAg (Hepatitis B surface antigen) indicates that he does not have an acute or chronic infection with hepatitis B virus. The presence of anti-HBc IgG (antibodies to hepatitis B core antigen) indicates a previous exposure to hepatitis B virus, but the presence of anti-HBs IgG (antibodies to hepatitis B surface antigen) suggests immunity.  The combination of positive anti-HBc IgG and positive anti-HBs IgG is typically seen in individuals who have resolved a previous hepatitis B infection or have been successfully vaccinated against hepatitis B. In this case, the presence of anti-HBs IgG suggests that he has protective antibodies against hepatitis B, which is consistent with immunity.  Therefore, the best description of his hepatitis B status is immune due to previous vaccination for hepatitis B (choice D). It indicates that he has developed immunity against hepatitis B, either through previous vaccination or natural infection that has resolved. |
|  |  | Attempt 3  Based on the provided investigation results, the best description of his hepatitis B status is D. Immune due to previous vaccination for hepatitis B.  The negative HBsAg (Hepatitis B surface antigen) indicates that he does not have an acute or chronic infection with hepatitis B virus. The presence of anti-HBc IgG (antibodies to hepatitis B core antigen) indicates a previous exposure to hepatitis B virus, but the presence of anti-HBs IgG (antibodies to hepatitis B surface antigen) suggests immunity.  The combination of positive anti-HBc IgG and positive anti-HBs IgG is typically seen in individuals who have resolved a previous hepatitis B infection or have been successfully vaccinated against hepatitis B. In this case, the presence of anti-HBs IgG suggests that he has protective antibodies against hepatitis B, which is consistent with immunity.  Therefore, the best description of his hepatitis B status is immune due to previous vaccination for hepatitis B (choice D). It indicates that he has developed immunity against hepatitis B, either through previous vaccination or natural infection that has resolved. |
| **97** | A 65 year old woman has had headaches for 3 months. She was treated for cancer of the right breast 12 years ago. There is no evidence of local or regional recurrence. Examination of the central nervous system is normal. CT scan of brain shows an isolated metastasis with cerebral oedema.  Which is the most appropriate immediate treatment?  A. Anastrozole  B. Chemotherapy  C. Dexamethasone  D. Radiotherapy  E. Surgical decompression | Attempt 1  The most appropriate immediate treatment for a 65-year-old woman with an isolated metastasis and cerebral edema detected on CT scan of the brain, following a history of breast cancer, is C. Dexamethasone.  Cerebral edema is a potential complication of brain metastasis and can lead to neurological symptoms and increased intracranial pressure. Dexamethasone, a corticosteroid, is commonly used to reduce cerebral edema and alleviate symptoms in such cases. It acts by reducing inflammation and swelling around the tumor.  Dexamethasone should be initiated promptly to address the cerebral edema and relieve the associated symptoms. Additional treatments, such as radiotherapy or chemotherapy, may be considered for long-term management of the metastasis, but the immediate priority is to control the edema and its effects. |
|  |  | Attempt 2  The most appropriate immediate treatment for a 65-year-old woman with an isolated brain metastasis and cerebral edema following a history of breast cancer is:  C. Dexamethasone  Dexamethasone is a corticosteroid medication used to reduce cerebral edema and swelling in the brain, which can help alleviate the symptoms like headaches and improve the patient's neurological status. It is considered the first-line treatment in managing brain edema caused by metastatic brain lesions.  The other options mentioned are as follows:  A. Anastrozole: Anastrozole is an aromatase inhibitor used in hormone receptor-positive breast cancer, but it is not appropriate for treating isolated brain metastases.  B. Chemotherapy: Chemotherapy may be used in the treatment of breast cancer, but it may not be the immediate treatment choice for isolated brain metastases. Other localized treatments like radiotherapy or surgery are often considered first.  D. Radiotherapy: Radiotherapy is commonly used in the treatment of brain metastases. However, in this scenario, the patient presents with cerebral edema, which needs to be addressed before considering definitive treatment like radiotherapy.  E. Surgical decompression: Surgical decompression may be considered in some cases, but it is not the immediate treatment of choice, especially when cerebral edema is present. Steroids are usually given to reduce edema before considering surgery or radiotherapy.  In summary, for the immediate treatment of an isolated brain metastasis with cerebral edema, administering dexamethasone to reduce the edema would be the most appropriate option. Subsequent treatments like radiotherapy or surgery may be considered based on the patient's overall condition and response to initial management. The patient's medical team will make the final decision based on individual circumstances. |
|  |  | Attempt 3  Based on the given information, the most appropriate immediate treatment for a 65-year-old woman with an isolated metastasis and cerebral edema after a history of breast cancer would be:  C. Dexamethasone  Dexamethasone is a corticosteroid commonly used to reduce cerebral edema and inflammation in cases of brain metastases. It helps alleviate symptoms associated with increased intracranial pressure, such as headaches, and can improve overall comfort and quality of life. Dexamethasone does not directly treat the underlying cancer but is used to manage the symptoms and complications associated with brain metastases.  While other treatment options like anastrozole (A), chemotherapy (B), radiotherapy (D), and surgical decompression (E) may have roles in the overall management of metastatic breast cancer, the immediate priority in this case is to address the cerebral edema and relieve symptoms. Therefore, initiating dexamethasone would be the most appropriate immediate treatment in this scenario. Subsequent treatment decisions, such as radiotherapy or systemic therapy, may be considered in consultation with an oncologist or specialist. |
| **98** | A 45 year old man has had two hours of colicky left sided abdominal pain radiating to his groin. The pain started abruptly and is associated with nausea and vomiting. He is restless and writhing in pain. There is tenderness on palpation of the left costovertebral angle. Abdominal examination is normal. Bowel sounds are present but scanty. His urinalysis has 3+.  Which is the most appropriate initial analgesic agent?  A. Aspirin  B. Diclofenac  C. Oxycodone  D. Paracetamol  E. Tramadol | Attempt 1  The most appropriate initial analgesic agent for a 45-year-old man with colicky left-sided abdominal pain radiating to his groin, tenderness on palpation of the left costovertebral angle, and 3+ on urinalysis is E. Tramadol.  The described symptoms and findings are consistent with renal colic, which is commonly caused by the passage of a kidney stone. Tramadol is a centrally acting opioid analgesic that can provide effective pain relief for renal colic. It acts by binding to opioid receptors in the central nervous system, inhibiting pain signals.  While other analgesics like nonsteroidal anti-inflammatory drugs (NSAIDs) such as diclofenac or opioids such as oxycodone can also be used for pain relief in renal colic, tramadol is often considered as a first-line option due to its efficacy and tolerability. It can provide effective pain relief while minimizing the risk of adverse effects associated with stronger opioids. However, the choice of analgesic may vary based on individual patient factors and clinician preference, so it's important to consult a healthcare professional for personalized advice. |
|  |  | Attempt 2  Based on the given clinical presentation, the most appropriate initial analgesic agent for the 45-year-old man with colicky left-sided abdominal pain radiating to his groin, tenderness on palpation of the left costovertebral angle, and 3+ urinalysis is:  B. Diclofenac  The clinical presentation is suggestive of renal colic, which is typically caused by the passage of a kidney stone through the urinary tract. Diclofenac is a nonsteroidal anti-inflammatory drug (NSAID) that can help relieve the pain associated with renal colic. It works by reducing inflammation and blocking the production of prostaglandins, which are chemicals that promote pain, swelling, and inflammation.  Other NSAIDs like Ibuprofen or Naproxen could also be considered, but Diclofenac is a commonly used option for this type of pain. It's essential to provide appropriate pain relief while further investigations and management of the underlying cause are undertaken. Aspirin and Paracetamol (acetaminophen) may not be as effective for renal colic pain as NSAIDs. Oxycodone is a stronger opioid analgesic that may be reserved for cases where NSAIDs are ineffective or not tolerated. However, it is not typically the first-line choice for renal colic pain. Always consult a healthcare professional for proper evaluation and management of any medical condition. |
|  |  | Attempt 3  Given the presentation of colicky left-sided abdominal pain radiating to the groin, tenderness on palpation of the left costovertebral angle, and the presence of 3+ in the urinalysis, the most likely diagnosis is renal colic, which is commonly caused by urinary stones.  To alleviate the intense pain associated with renal colic, the most appropriate initial analgesic agent would be:  E. Tramadol  Tramadol is a centrally acting analgesic that can provide effective pain relief for renal colic. It acts on the opioid receptors in the brain, reducing pain perception. It is often used as a first-line analgesic in the management of moderate to severe renal colic pain.  While other options like diclofenac (B) and paracetamol (D) can provide some pain relief, they may not be as effective as tramadol in managing the intense colicky pain associated with renal colic. Aspirin (A) is not typically recommended for renal colic due to its limited analgesic effect in this context. Oxycodone (C) is a potent opioid analgesic that may be reserved for severe cases or when other analgesics are ineffective.  It's important to note that the definitive management of renal colic involves addressing the underlying cause, such as urinary stone removal or intervention. Therefore, the patient should be further evaluated and managed by an appropriate healthcare professional. |
| **99** | A 70 year old man has had 1 day of cough and breathlessness. He has a history of COPD and heart failure. His normal exercise tolerance is 100 m. He has had no previous hospital admissions. He has a 35 pack-year smoking history. He is taking lisinopril and uses a tiotropium inhaler. He is cyanosed and dyspnoeic. His pulse rate is 100 bpm, BP 105/78 mmHg, respiratory rate 30 breaths per minute and oxygen saturation 84% breathing 28% oxygen. He has scattered wheeze and crackles bilaterally. He has been given nebulised salbutamol. Investigations: Arterial blood gas on 28% oxygen pH 7.25 (7.35-7.45) PO2 6.9 kPa (11-15) PCO2 7.8 kPa (4.6-6.4) Bicarbonate 34 mmol/L (22-30)  Which is the most appropriate next step in management?  A. Increase oxygen to 40% via a Venturi mask  B. Intravenous amoxicillin  C. Intravenous furosemide  D. Non-invasive ventilation  E. Reduce oxygen to 2 L/min via a nasal cannula | Attempt 1  Based on the clinical presentation and arterial blood gas results of a 70-year-old man with a history of COPD and heart failure, the most appropriate next step in management is D. Non-invasive ventilation.  The patient is experiencing acute exacerbation of COPD with respiratory acidosis (low pH), hypoxemia (low PO2), and hypercapnia (elevated PCO2). The initial treatment with nebulized salbutamol addresses bronchospasm but does not adequately improve the patient's oxygenation and ventilation status.  Non-invasive ventilation (NIV) is recommended in acute exacerbations of COPD with respiratory failure. It helps provide ventilatory support and corrects acidosis by improving oxygenation and reducing carbon dioxide levels. NIV is often delivered using a mask interface such as a continuous positive airway pressure (CPAP) or bilevel positive airway pressure (BiPAP) machine. This treatment can help avoid the need for invasive mechanical ventilation and is associated with improved outcomes in selected patients.  Increasing the oxygen concentration alone (option A) may worsen the hypercapnia by reducing the patient's respiratory drive. Intravenous amoxicillin (option B) would not be the immediate priority in this case. Intravenous furosemide (option C) may be beneficial in heart failure exacerbations, but the primary issue in this scenario is respiratory failure related to COPD. Reducing oxygen flow to 2 L/min (option E) would not adequately address the patient's respiratory distress and hypoxemia. |
|  |  | Attempt 2  The given clinical presentation of a 70-year-old man with a history of COPD and heart failure, presenting with cough, breathlessness, cyanosis, and low oxygen saturation (84%) requires urgent and appropriate management. Based on the provided information, the most appropriate next step in management would be:  D. Non-invasive ventilation  The arterial blood gas results show significant respiratory acidosis (pH 7.25) with low PO2 (6.9 kPa) and high PCO2 (7.8 kPa), indicating acute respiratory failure. The patient's dyspnea, low oxygen saturation, and bilateral wheeze and crackles suggest an exacerbation of his COPD and possible acute-on-chronic respiratory failure.  Non-invasive ventilation (NIV), such as continuous positive airway pressure (CPAP) or bilevel positive airway pressure (BiPAP), is the appropriate next step in this scenario. NIV can help improve ventilation and oxygenation, reduce the work of breathing, and provide positive pressure support, which is crucial in managing acute respiratory failure in patients with COPD.  Options A, B, C, and E are not the most appropriate next steps in this situation:  A. Increasing oxygen to 40% via a Venturi mask may worsen the hypercapnia (high PCO2) and worsen respiratory acidosis in COPD patients, as they depend on hypoxic drive to maintain their respiratory effort.  B. Intravenous amoxicillin is not the priority at this moment as the patient's primary issue is respiratory failure, and there are no specific indications of bacterial infection mentioned in the provided information.  C. Intravenous furosemide (a diuretic) may not be helpful in the acute management of respiratory failure in COPD patients, especially if the underlying issue is not primarily related to congestive heart failure exacerbation.  E. Reducing oxygen to 2 L/min via a nasal cannula would be inadequate oxygen supplementation for a patient with significant hypoxia and acute respiratory failure.  However, it's crucial to involve a healthcare professional promptly in the management of this patient, as he presents with acute respiratory distress and requires immediate evaluation and intervention. |
|  |  | Attempt 3  Given the clinical presentation of a 70-year-old man with a history of COPD and heart failure who presents with acute onset cough, breathlessness, cyanosis, and low oxygen saturation, the most likely diagnosis is acute exacerbation of COPD with respiratory failure.  Based on the arterial blood gas results showing respiratory acidosis (low pH, elevated PCO2) and hypoxemia (low PO2), the most appropriate next step in management would be:  D. Non-invasive ventilation  Non-invasive ventilation (NIV) is the recommended initial treatment for acute exacerbations of COPD with respiratory failure. NIV helps improve oxygenation, reduce work of breathing, and decrease carbon dioxide levels. It involves the application of positive pressure ventilation via a mask, which can be delivered using devices such as bilevel positive airway pressure (BiPAP) or continuous positive airway pressure (CPAP). NIV has been shown to improve outcomes and reduce the need for endotracheal intubation in patients with acute exacerbations of COPD.  Options A and E, which involve adjusting oxygen delivery, may not be sufficient in improving respiratory status in this patient with acute respiratory failure. Intravenous amoxicillin (B) may be considered if there is suspicion of a concurrent bacterial infection, but the primary concern in this case is respiratory support. Intravenous furosemide (C) may be used in heart failure management but does not address the underlying acute exacerbation of COPD.  It is important to involve a healthcare professional to initiate and manage non-invasive ventilation in this patient to optimize his respiratory status and improve outcomes. |
| **100** | A 2 day old baby boy undergoes his routine neonatal hearing screen. His mother has been deaf since early childhood and has bilateral hearing aids. She asks whether her baby will be deaf.  Which is the most appropriate additional assessment?  A. Brainstem evoked response test  B. Cytomegalovirus serology  C. Distraction testing  D. MR scan of brain  E. Weber's test | Attempt 1  The most appropriate additional assessment in this scenario would be A. Brainstem evoked response test.  Given the mother's history of deafness and the potential genetic component of hearing loss, it is important to assess the baby's hearing thoroughly. The brainstem evoked response test, also known as auditory brainstem response (ABR) or brainstem auditory evoked potential (BAEP), is a reliable test used to evaluate the integrity of the auditory pathway from the ear to the brainstem. It measures the electrical responses generated by the auditory system in response to sound stimulation.  The ABR test is commonly used in newborn hearing screening programs as it can detect hearing loss even in infants who are too young to respond behaviorally. It provides objective information about the baby's hearing thresholds and can help identify any potential hearing loss or impairment.  The other options listed are not the most appropriate assessments in this specific situation. Cytomegalovirus serology (option B) is relevant in cases of suspected congenital cytomegalovirus infection, which can be a cause of hearing loss, but it is not the primary consideration in this case. Distraction testing (option C) is a behavioral method used to assess hearing in older infants, but it is not suitable for a 2-day-old baby. An MR scan of the brain (option D) would not be the initial assessment for hearing loss but may be considered in specific cases where there are additional concerns about the baby's neurological development. Weber's test (option E) is a tuning fork test used to assess hearing asymmetry in individuals with unilateral hearing loss, and it would not provide comprehensive information about the baby's hearing status. |
|  |  | Attempt 2  The most appropriate additional assessment in this case would be:  A. Brainstem evoked response test  Given the family history of the mother being deaf and wearing bilateral hearing aids, there is a higher risk of her baby being born with a hearing impairment. The routine neonatal hearing screen is a standard procedure performed shortly after birth to identify babies at risk for hearing loss.  If the initial hearing screen indicates a potential problem or there is a family history of hearing impairment, the next step in the evaluation would be to perform a more comprehensive assessment of the baby's hearing. The brainstem evoked response test (also known as auditory brainstem response or ABR) is a commonly used objective test to assess the auditory pathway from the ear to the brainstem. It measures the brain's response to sound stimuli and can identify hearing loss in newborns and infants.  Options B, C, D, and E are not the most appropriate assessments for identifying potential hearing loss in a newborn with a family history of hearing impairment:  B. Cytomegalovirus serology is a test for cytomegalovirus (CMV) infection, which can be a cause of congenital hearing loss. While it is essential to consider infections as a possible cause, CMV testing is not the first-line assessment in routine neonatal hearing screening.  C. Distraction testing is a behavioral hearing test that involves observing the baby's response to sound stimuli, but it may not be sufficient for a conclusive assessment of hearing loss at such a young age.  D. MR scan of the brain is not the first-line investigation for routine neonatal hearing screening. It is typically performed when there are specific indications for assessing the brain's anatomy or when hearing loss is associated with other neurological issues.  E. Weber's test is a tuning fork test used in older children and adults to assess unilateral hearing loss or middle ear problems. It is not suitable for neonatal hearing screening.  Remember that any concerns regarding a baby's hearing should be promptly discussed with a healthcare professional, and further evaluations should be performed by trained audiologists or specialists in pediatric audiology. Early identification of hearing impairment is crucial for timely interventions and support for the child's development. |
|  |  | Attempt 3  The most appropriate additional assessment in this scenario would be:  A. Brainstem evoked response test  The brainstem evoked response test, also known as auditory brainstem response (ABR) test, is a commonly used diagnostic tool to assess the hearing ability of infants. It measures the electrical activity of the auditory pathway in response to sound stimulation. This test can provide information about the baby's hearing thresholds and can help identify any hearing loss.  Given the mother's history of deafness and the potential genetic risk for hearing loss in the baby, performing a brainstem evoked response test would be a valuable assessment to determine the baby's hearing status. It is a non-invasive test that can be conducted in young infants, providing objective information about the functioning of the auditory system.  Option B, cytomegalovirus serology, is relevant in cases where congenital cytomegalovirus infection is suspected as a cause of hearing loss, but it is not the most appropriate initial assessment in this scenario. Option C, distraction testing, may be used in older infants to assess their response to sound, but it is not the most appropriate assessment for a 2-day-old newborn. Option D, an MRI scan of the brain, is not typically indicated as an initial assessment for newborn hearing screening. Option E, Weber's test, is a tuning fork test used to evaluate hearing loss in older children and adults but is not appropriate for a 2-day-old infant.  It is important to involve a healthcare professional experienced in neonatal hearing assessment to perform the appropriate tests and provide guidance to the mother based on the results. |
